# Evolutionary and Functional Conservation of Topologically Associating Domain Boundaries in Rice Genomes

**DOI:** 10.3390/ijms27208979

**Published:** 2026-10-10

**Authors:** Owen Geyman, Amina Kurbidaeva

**Affiliations:** 1 Center for Genomics and Systems Biology, Department of Biology, New York University, 12 Waverly Place, New York, NY 10003, USA; 2 The Mastery Learning System, Avenues The World School, 259 10th Avenue, New York, NY 10001, USA; 3 Department of Systems Biology, Columbia University, New York, NY 10032, USA

**Keywords:** TAD boundaries, TCBF, 3D genomics, rice, genome architecture, comparative genomics, evolutionary genomics

## Abstract

Traditional evolutionary analysis has relied primarily on one-dimensional (1D) sequencing data, but recent advances in three-dimensional (3D) genomics allow for deeper investigation of how genome architecture shapes evolutionary divergence. In this study, we analyze conserved and species-specific topologically associating domain (TAD) boundaries across multiple *Oryza* species to uncover both structural and functional evolutionary insights. Here, we characterize boundary conservation at different levels, from highly conserved to species-unique. We apply TCBF, a relatively novel and underutilized tool for TAD boundary conservation analysis, alongside a suite of complementary analyses, including gene ontology, RNA-seq, sequence conservation, and epigenetic profiling. Our approach not only validates canonical evolutionary relationships reflected in sequence data but also novel patterns unique to 3D genome organization. Most notably, our study uncovers strong associations between highly conserved TAD boundaries and RNA metabolism-related genes, as well as other genetic and epigenetic signatures, and a contrasting absence in minimally conserved boundaries. To our knowledge, we created the first phylogenetic reconstruction derived from topological domain data in plants, which demonstrated a strong similarity between 3D and 1D genomic evolutionary relationships. Overall, our work presents an approach that integrates the 3D genome structure into evolutionary genomics, addressing a critical and underexplored dimension of comparative plant genomics.

## 1. Introduction

Three-dimensional (3D) genomics aim to understand the structure, function, and heterogeneity of genomes by analyzing factors that shape higher-order chromatin organization. Nuclear DNA is compacted into chromatin, a complex of DNA and histone proteins, which undergo selective, location-specific activation to direct transcriptional activity. Cell-type-specific chromatin states, shaped by and shaping dynamic transcription, have become a powerful framework for mechanistic and translational research, with growing focus on the three-dimensional organization of the genome. 3D genomics aims to expand our understanding of genomic organizations beyond traditional one-dimensional (1D) sequence-driven research by recognizing that all genetic material has spatial properties. In recent years, the field of 3D genomics has expanded rapidly, with an increasing amount of research performed in an interspecies and comparative context [1], following the adoption and modernization of spatial genomic assays, including Chromosome Conformation Capture (3C), such as Hi-C [2] and Micro-C [3], ChIP-seq (Chromatin Immunoprecipitation Sequencing) [4], and ChromEMT [5].

Advancements in 3D genomics have, and will continue to, catalyze significant developments across scientific, biomedical, and agricultural disciplines. These include the identification of enhancer–promoter loops that govern transcriptional activity and reflect dynamic chromatin state and compartmentalization [6], and the discovery of topologically associating domains (TADs) and their role in core gene-regulatory architecture [7,8]. TADs, ubiquitous 3D-genomic domains pervasive across genomic landscapes, facilitate frequent self-interactions between DNA sequences [7,8]. These domains, typically ranging from kilobase- to megabase-sized regions, depending on the species, serve as organizational units that constrain interactions between genes and coordinated cis-regulatory elements, and help elucidate observations of linearly distant genetic interactions, which, in reality, are spatially proximal [7,8]. Each TAD is flanked by two TAD boundaries, which are genomic fragments that act as demarcators of interaction activity, preventing inter-TAD regulatory communication networks and enhancing intra-TAD chromatin contacts [9]. In mammalian cells, TAD boundaries are defined by the interaction between cohesin and CTCF, two proteins consequently vital for the formation of 3D genomic topologies [6,9]. Yet, it has been widely shown that CTCF, or any functional homolog, is absent in plants, albeit with the presence of various cohesin sub-units [10,11,12,13,14,15,16]. As such, mechanisms aiding in TAD boundary formation remain elusive in plants, and while potential boundary proteins have been identified, none have been strongly confirmed [11,12,17]. Current research is both focused on the formation of these boundaries, and on the transcriptional and epigenetic profiles of inter/intra-TAD regions in plants to better understand their phenotypic effects [11,12,17].

In addition to TADs’ critical role in maintaining both genomic organization and stability through pervasive transcriptional regulation across organisms [18,19], TADs and their boundaries are currently being investigated as a tool to understand evolutionary relationships more thoroughly [20,21,22]. Similar to mammalian species, the extent of TAD conservation appears to vary considerably across plant species. Only 8.23% of foxtail millet TADs were conserved in sorghum, with similarly low conservation reported between sorghum and maize [23]. At the species level, approximately 25% of TADs were conserved between *Brassica rapa* and *Brassica oleracea* [24], whereas 40–48% were conserved between two poplar species [25]. However, differences in Hi-C map resolution and criteria used to define TAD conservation across these studies limit direct comparisons and preclude a general estimate of the extent of TAD conservation across plants. Such investigations are vital as they allow for the understanding of natural mechanisms of the adaptations of certain plant species to environmental changes, enabling researchers to record, manipulate, and replicate specific molecular, cellular, and anatomical biomarkers associated with beneficial phenotypes for plant improvement. Some target traits for improvement include crop yield, drought and cold tolerance, plant vigor, flowering time, pest and herbicide resistance, and nutritional content [26]. Initial studies in plant species, based on limited yet persuading evidence from Arabidopsis [27,28,29], suggested that plant species lack TADs entirely; this assumption was disproven to apply to all plants after strong evidence of TAD presence in numerous other plant species arose [19,23,30,31,32], although the original conclusion still holds for Arabidopsis [33]. Recent findings [19] dictate clear 3D-genomic-based evolutionary relationships between certain rice species and suggest key correlations between functional characteristics, such as structural variations, histone modifications, and epigenetic profiles, and conserved TADs. Studies also indicate varying epigenetic signatures near or on plant TAD boundaries [19,34], and a scarcity of chromatin looping in TAD boundary domains, but rather between CpG islands [23], all of which are akin to conventional findings associated with mammalian TAD boundaries [20]. While 3D-genomic-based research has concentrated largely on the mammalian/drosophila genome and has recently begun advancing into plant research, applications in plants and non-mammalian organisms remain limited, leaving the 3D architecture of numerous genomes comparatively understudied due to limited integration of advanced spatial genomic methods.

Our study aims to characterize the functional and structural landscape of TAD boundaries across multiple *Oryza* genomes, to compare their interspecific conservation, and to provide insight into evolutionary relationships among rice species through the lens of 3D genome architecture. Previous research has investigated TAD conservation across various plant species, with several studies reporting moderate levels of interspecific conservation [19,23,24,25,30,31]; however, these efforts often rely on disparate methodologies, distinct organisms, and inconsistent definitions of what constitutes a conserved TAD. Only a few studies [19,35] have tentatively explored plant evolutionary patterns shaped by TAD boundary abundance, but failed to distinguish levels of TAD boundary conservation, focusing primarily on the TADs themselves. Collectively, these limitations prevent prior work from generating clear, inference-driven conclusions about evolutionary interrelationships or from establishing a coherent, 3D-genomics-based evolutionary framework for plant species. Here, we analyze TAD boundary conservation across multiple Asian domesticated rice (*Oryza sativa*) landraces, including Azucena (tropical japonica), Nipponbare (temperate japonica), and IR64 (indica), and two closely related wild species, *O. rufipogon* and *O. meridionalis*. While both are wild species, *O. rufipogon* retains greater geographic (Asian) and genetic similarity to the domesticated cultivars, having diverged by approximately 0.30 Mya, whereas *O. meridionalis* is uniquely Australasian and diverged considerably earlier, at approximately 2.41 Mya [36]. We apply a novel and underutilized tool for boundary conservation detection that can be applied to the nuclear genome of any species with accurate genomic information [35], and unprecedentedly, allows for the direct comparison of TAD boundaries interspecifically without the need for any coordinate-based genomic conversions (liftOver). LiftOver-based methods, including the study conducted by Kurbidaeva et al. 2025 [19], define conserved TADs and TAD boundaries based on the translation of their coordinates between species with the liftOver tool. This tool relies on the pairwise genome alignments between the species, which experiences inaccuracies stemming from complex structural variants and ambiguous mapping. TCBF incorporates synteny block analysis and graph clustering into its pipeline, increasing accuracy of interspecies comparisons of TAD boundaries. In addition, the approach used by Kurbidaeva et al. 2025 [19] first determines conservation at the TAD level and subsequently defines fixed-width boundary regions flanking those of conserved TADs, instead of analyzing the TAD boundary sequences directly. Thus, conserved boundaries are often used as proxies for conserved TADs, although this may introduce computational artifacts that do not fully reflect biological reality. Results from the tool were then used for further qualitative and quantitative analyses, including those involving structural, functional, and evolutionary objectives (Figure S1). We then synthesized our findings to generate a clear, unprecedented 3D-genomic-based phylogenetic tree that reflects the evolutionary trajectories shaped by 3D genome architecture in our selected wild and domesticated rice cultivars, and used it for comparison with canonical one-dimensional evolutionary reconstructions (Figure S1).

## 2. Results

### 2.1. Interspecific Conservation of Rice TAD Boundaries

**Figure 1:**
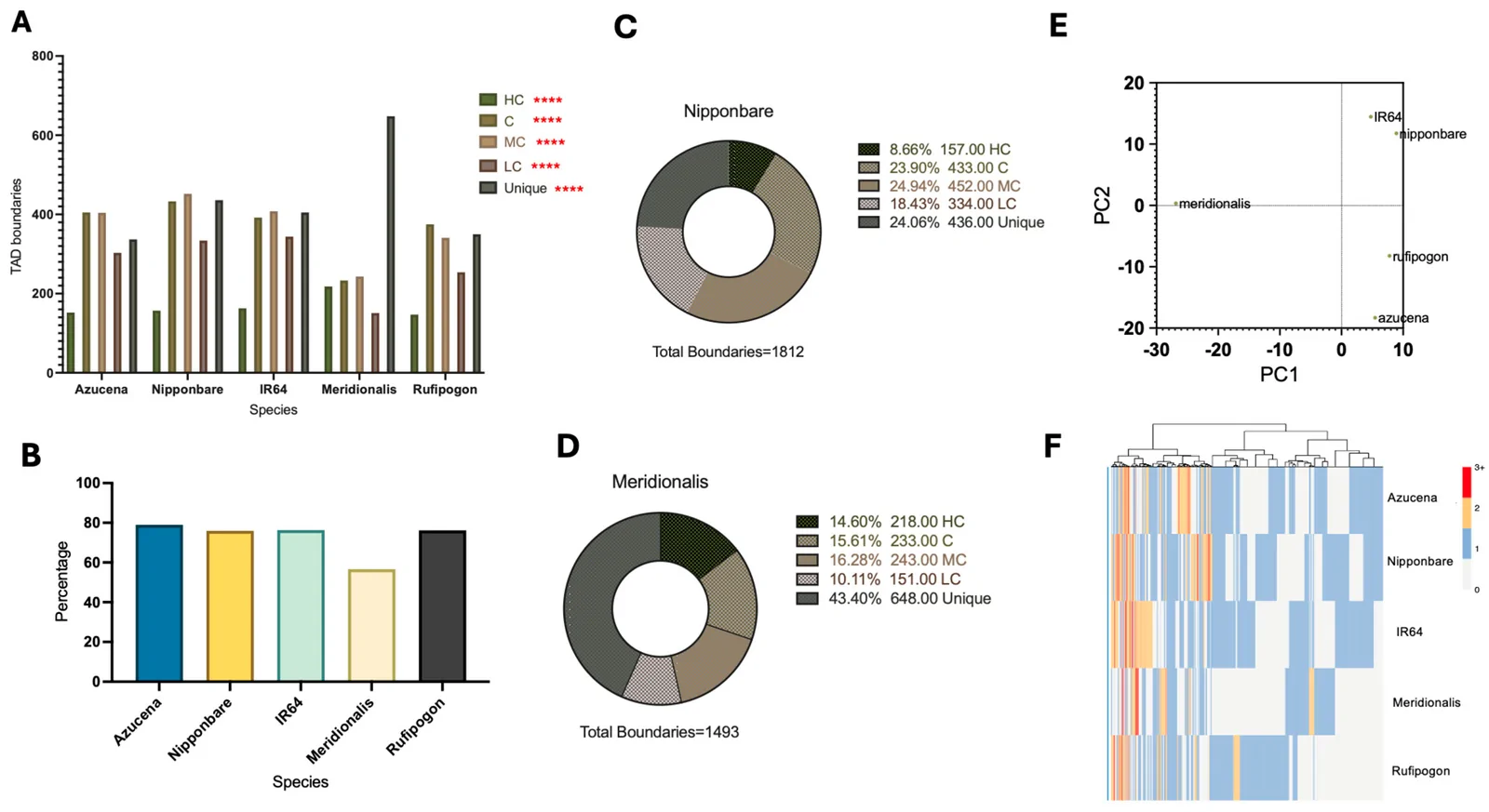
(**A**) Quantity of TAD boundaries stratified across selected rice species and conservation levels. Significance of Fisher’s exact tests depicted as **** *p* < 0.0001. (**B**) Percentage of Nipponbare TAD boundaries conserved by level against total boundaries present in the Nipponbare genome. Each conservation level is represented by a unique color. (**C**) Percentage of *O. meridionalis* TAD boundaries conserved by level against total boundaries present in the *O. meridionalis* genome. (**D**) Principal component analysis (PCA) of species-specific TAD boundary conservation group profiles. Each point represents a species. The first two principal components (PC1 and PC2) are shown, collectively explaining ~50% of the total variance. (**E**) Bar plot depicting the proportion of total conserved boundaries (accumulation of HC, C, MC, and LC) to total boundaries for each species. (**F**) Heatmap of phylogenomic profiles across selected rice species. Hierarchical clustering was performed based on species-specific distributions of TAD boundary conservation groups. Color intensity represents the number of TAD boundaries within each group for each species. Statistical significance was determined by Kruskal–Wallis testing (*p* < 0.0001). Categorical pairwise significance (determined by FDR-adjusted post-hoc Dunn’s multiple comparison tests) is reported in-text.

The TCBF tool was used to identify conserved TAD boundary domains across five selected wild and domesticated *O. sativa* genomes and to classify boundaries according to their degree of conservation (Figure 1A). Across all genomes, conserved (C) and moderately conserved (MC) boundaries represented the most abundant conservation classes, with both particularly enriched in the Nipponbare reference genome and differing by no more than approximately ±50 boundaries among Azucena, IR64, and *O. rufipogon*. Unique boundaries were similarly abundant across these four genomes, with C, MC, and Unique boundary counts consistently exceeding 200. In contrast, HC boundaries were substantially less abundant than the other non-unique conservation categories in all genomes except *O. meridionalis*, while LC boundaries consistently represented either the least or second least abundant conservation class. Across multiple metrics, *O. meridionalis* exhibited a markedly distinct conservation profile compared to the other cultivars. Relative to the Nipponbare reference genome, both HC and Unique boundaries were enriched, with Unique boundaries increasing by 19.32% of the total TAD boundary landscape (Figure 1C,D). Correspondingly, *O. meridionalis* possessed the lowest overall proportion of conserved TAD boundaries among all analyzed genomes, whereas Azucena displayed the highest level of conservation by a slight margin (Figure 1B). Differences in conservation category distributions were statistically significant across all five genomes (Figure 1A). Principal component analysis (PCA) further highlighted the distinct genomic architecture of *O. meridionalis* (Figure 1E). On PC1, *O. meridionalis* formed a pronounced outlier, whereas the remaining genomes clustered closely together. In contrast, PC2 separated the genomes into two distinct groups, with Nipponbare clustering with IR64 and Azucena clustering with *O. rufipogon*, while *O. meridionalis* occupied an intermediate position almost precisely between the two clusters. A three-dimensional PCA plot incorporating PC3 demonstrated that the apparent clustering along PC2 was sensitive to the inclusion of additional principal-component variation, with Nipponbare shifting toward *O. rufipogon* and Azucena toward IR64, while *O. meridionalis* retained an almost unchanged PC2 position (Figure S3). None of the PCA axes demonstrate Nipponbare–Azucena clustering. Heatmap clustering further emphasized the statistically significant divergence of the *O. meridionalis* genome (*p* < 2.2 × 10^−16^), revealing two extensive regions lacking conserved TAD boundaries within the TCBF conservation matrix, indicative of localized depletion of 3D genome conservation (Figure 1F). A comparable, though less pronounced, region was also observed in *O. rufipogon* at corresponding genomic positions. In contrast, Nipponbare and Azucena exhibited a more continuous distribution of conserved boundaries throughout their genomes, with relatively few regions devoid of conservation. Our results indicate highly statistically significant (*p* < 0.0001) relationships between *O. meridionalis* and all other rice cultivars, as well as between *O. rufipogon* and Nipponbare and Azucena. The data from Figure 1 support an evolutionary and statistically significant divergence in 3D genome conservation in the *O. meridionalis* genome, supported by the quantity and clustering of both conserved and unique TAD boundaries.

### 2.2. TCBF Performance and TAD Boundary Group Creation

**Figure 2:**
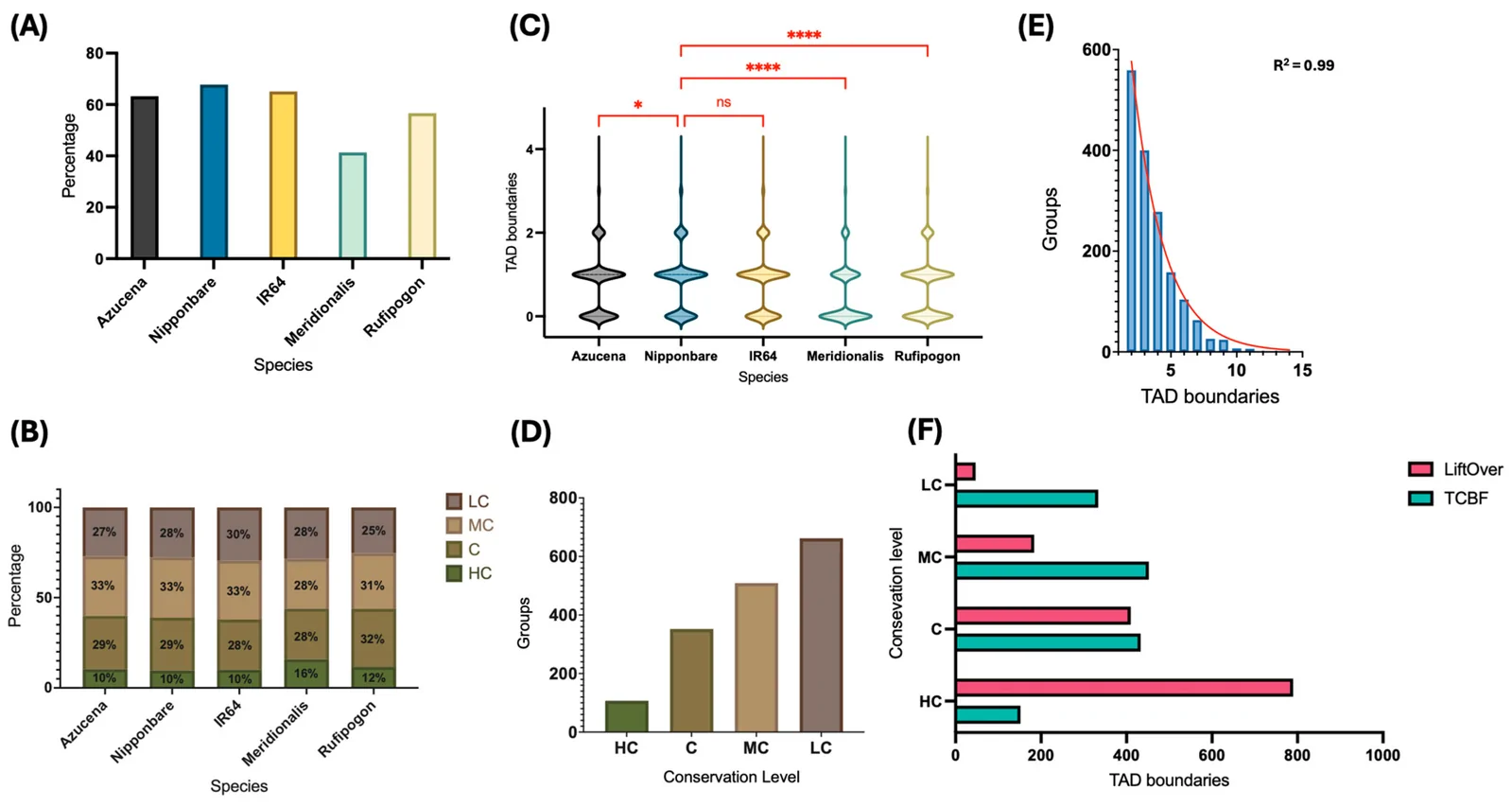
(**A**) Species’ presence across all TAD boundary groups represented by the proportion of TAD boundary groups containing the given species to total TAD boundary groups (n = 1630). (**B**) Conservation-level breakdown (by percentage) of TAD boundary groups containing a given species. (**C**) Violin plots showing the distribution of the number of TAD boundaries across TAD boundary groups per species. Significance of pairwise Welch’s *t*-tests depicted as ns *p* ≥ 0.05; * *p* < 0.05; **** *p* < 0.0001. Statistical significance was assessed using pairwise Welch’s *t*-tests comparing each species with Nipponbare (reference). (**D**) Bar plot depicting the quantity of TAD boundary groups associated with each conservation category (excluding Unique). (**E**) Histogram of total sizes of TAD boundary groups, based on the total accumulation of TAD boundaries present in the group across all species. A non-linear regression was created (plotted in R) to show statistical fit of data (R^2^ = 0.99). (**F**) Comparison of TAD boundary data between tool leveraged here (TCBF) and the canonical method (LiftOver) depicted by a horizontal bar plot representing the total quantity of TAD boundaries per conservation category output between methods.

The TCBF algorithm identifies evolutionarily conserved TAD boundary groups by clustering homologous TAD boundaries across the selected genomes according to their genomic positions and conservation. Across all 1630 identified TAD boundary groups, no species contributed more than three TAD boundaries to a single group, with the vast majority of groups containing only a single boundary per species rather than multiple conserved boundaries, indicating that interspecific conservation is overwhelmingly represented by one-to-one boundary relationships rather than complex multiplexed structures (Figure 2C). Species representation across TAD boundary groups was non-uniform and broadly reflected the evolutionary patterns observed previously. *O. meridionalis* was represented in fewer than 50% of all TAD boundary groups, the lowest proportion among the five analyzed genomes, whereas the Nipponbare reference genome was represented in nearly 70% of all groups (Figure 2A). Among the boundary groups in which each species was present, *O. meridionalis* exhibited a greater proportion of HC groups (16%) than the remaining genomes (10–12%), consistent with its distinct conservation landscape identified in the preceding analyses (Figure 2B). In contrast, the relative distributions of the remaining conservation categories were broadly similar among the other genomes. The internal composition of TAD boundary groups also differed statistically significantly for *O. meridionalis*. Groups lacking *O. meridionalis* boundaries were substantially more prevalent, whereas groups containing one or more *O. meridionalis* boundaries occurred less frequently, representing the opposite trend observed for the remaining four genomes (Figure 2C). The overall structure of TAD boundary groups followed highly consistent trends. The number of boundary groups decreased progressively with the increasing conservation level, demonstrating an inverse relationship between group abundance and conservation strength (Figure 2D). Likewise, the distribution of cumulative TAD boundary counts per group was strongly right-skewed (Figure 2E). Finally, TCBF-derived boundary counts were compared with those reported by Kurbidaeva et al. 2025 [19], which were generated using a LiftOver-based approach. TCBF identified greater numbers of LC-, MC-, and C-classified boundaries, whereas the LiftOver methodology identified substantially higher numbers of C and HC boundaries (Figure 2F). Although the complete boundary sets showed 100% genomic overlap, with a minimum overlap threshold set to 1 bp (unpublished data), conservation-category-specific concordance between TCBF and the previous LiftOver-based approach was substantially lower (Table S4). These differences likely reflect the distinct units of conservation assessed by the two frameworks: TCBF directly compares TAD boundaries across species, whereas the previous approach derives boundary classifications from the conservation status of their associated TADs. Figure 2, overall, denotes evolutionary trends effectively analogous to those aforementioned, highlighting that boundary-specific relationships are indeed apparent in both species- and conservation level-specific analyses, and demonstrating *O. meridionalis* as a divergent *Oryza* cultivar in 3D genomic terms.

### 2.3. Chromosomal Distribution of TAD Boundary Conservation

**Figure 3:**
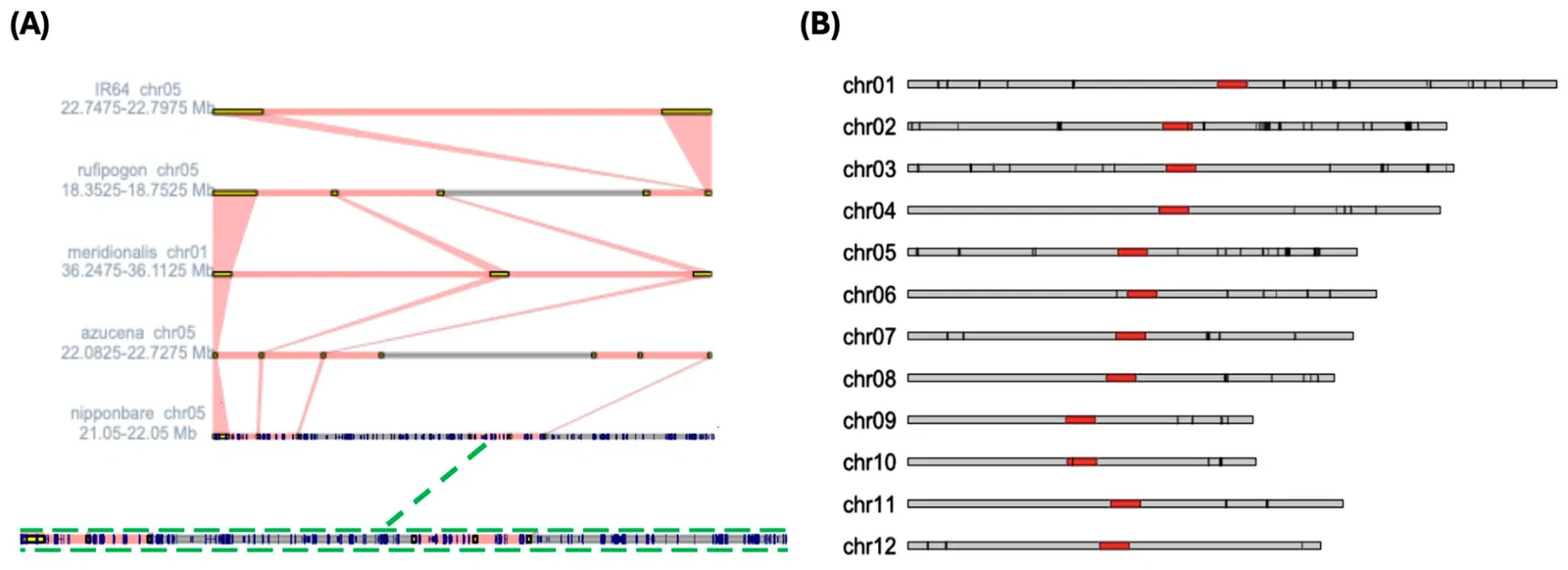
(**A**) Synteny relationship mapping of conserved TAD boundaries across respective 1 MB regions in each selected genome. Genes located within the displayed Nipponbare region are overlaid as blue segments. (**B**) Ideogram visualizing HC TAD boundary chromosomal localization for the Nipponbare genome. Black bars represent highly conserved TAD boundaries. Red regions denote centromeric sections of each chromosome.

Alongside quantitative analyses based on relative conservation scores, we visualized TAD boundary conservation within chromosomal context at both local and genome-wide scales. Synteny mapping, as described by He et al. 2023 [35], enables detailed visualization of cross-species relationships within a 1-Mb genomic window but is not readily suited to genome-wide comparisons. Within the selected region, TAD boundaries present on the *O. meridionalis* track are frequently aligned with boundaries across multiple other genomes, visually recapitulating our definition of highly conserved (HC) TAD boundaries (Figure 3A). This pattern is consistent with the previously observed enrichment of HC boundaries in *O. meridionalis*. Boundaries in *O. meridionalis* also appeared broader than those in the other rice genomes within this window; however, this localized visualization is insufficient to support conclusions regarding genome-wide differences in TAD boundary size. The gene map overlaid on the Nipponbare track further revealed an apparent scarcity of genes within conserved TAD boundaries despite substantial gene density across the surrounding 1-Mb region, suggesting a potential inverse relationship between highly conserved TAD boundary localization and gene distribution (Figure 3A). We next examined whether TAD boundary conservation was associated with chromosomal position at the genome-wide scale. Although no clear differences in HC boundary localization were observed across broad chromosomal regions, such as chromosome arms or telomeric regions, HC TAD boundaries were notably depleted from centromeric regions across the 12 Nipponbare chromosomes (Figure 3B). The same pattern, albeit less evident, was present when examining the chromosomal localization of all conserved Nipponbare boundaries, not only those of the highest conservation (Figure S4). HC boundary density also appeared to vary with chromosome identity, with a partial trend across chromosome number (Figure 3B). Together, these patterns suggest that TAD boundary conservation may reflect the functional maintenance of genomic architecture.

### 2.4. Functional Significance of Conserved TAD Boundaries

**Figure 4:**
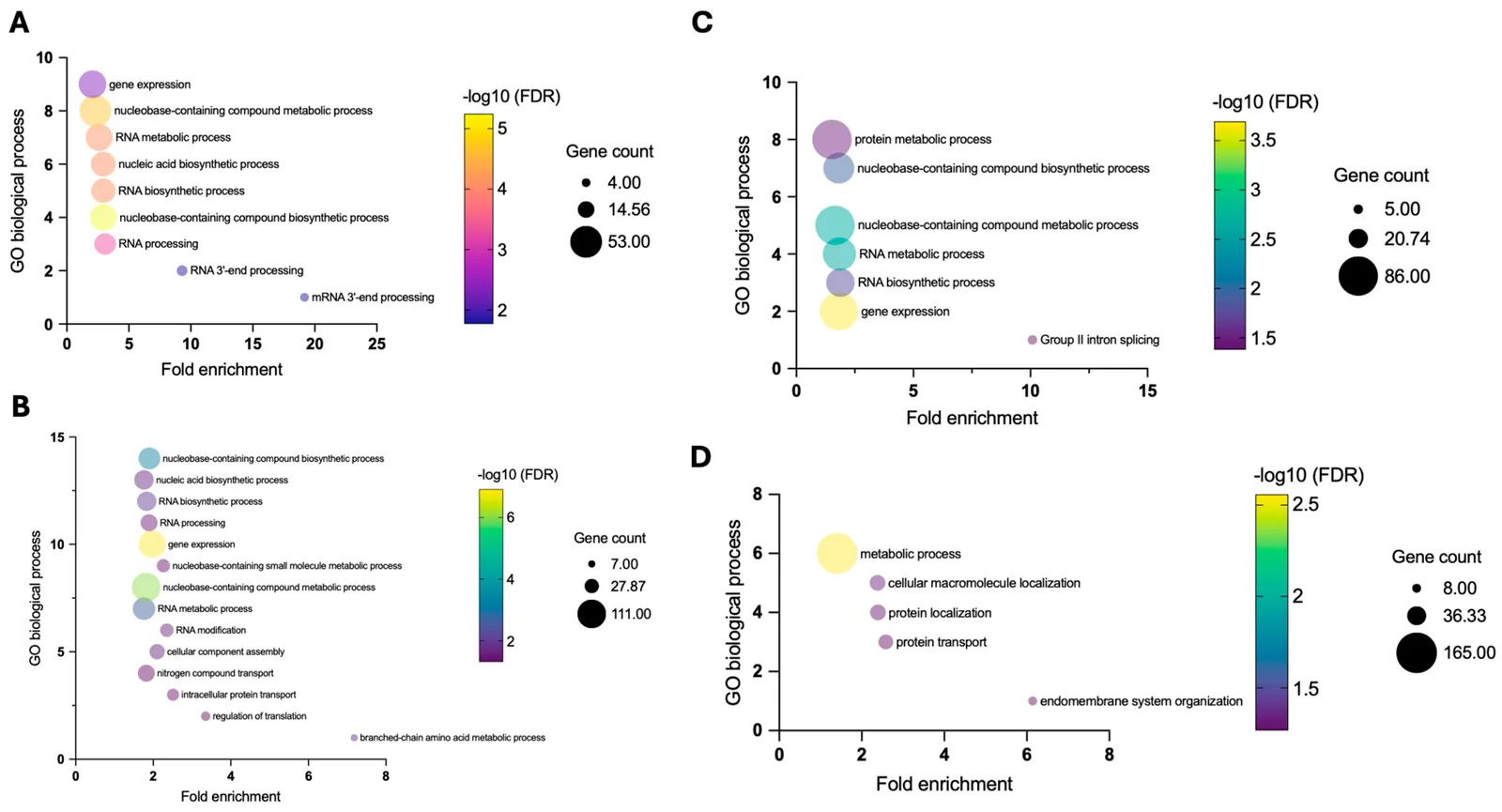
Gene Ontology (GO) biological process (BP) enrichment analysis from PANTHER of genes associated with (**A**) highly conserved, (**B**) conserved, (**C**) moderately conserved, and (**D**) least conserved TAD boundaries in the Nipponbare reference genome. Fold enrichment (observed proportion/expected proportion) is plotted for significantly enriched GO terms, ordered from lowest to highest fold enrichment. Bubble color represents the −log_10_-transformed FDR-adjusted *p*-value, while bubble size corresponds to the number of genes associated with each GO term.

To better understand the functional dynamics associated with TAD boundary conservation across rice species, we examined whether specific biological processes were enriched in conserved boundaries. We first performed Gene Ontology (GO) enrichment analysis with PANTHER in the Nipponbare genome for genes overlapping with TAD boundaries across conservation categories (Figure 4). Our results revealed a striking relationship between boundary conservation and the enrichment of RNA metabolism-associated genes. HC boundaries exhibited nine enriched GO Biological Process (BP) terms (Figure 4A), all of which were related to RNA metabolism, processing, or gene expression, whereas LC boundaries exhibited only four enriched GO-BP terms, none of which were associated with RNA-related processes (Figure 4D). C and MC boundaries displayed intermediate patterns, forming a progressive decline in RNA-associated enrichment with decreasing boundary conservation (Figure 4B,C). This pattern provides evidence of a functional association between evolutionary TAD boundary conservation and RNA metabolism-associated gene enrichment. Fold enrichment was particularly pronounced among GO terms associated with HC boundaries, reaching nearly 20-fold for mRNA 3′-end processing, with enrichment strength similarly declining across conservation categories. Notably, neither the total number of enriched GO terms nor the cumulative gene counts represented across enriched GO terms followed a similar conservation-dependent pattern; rather, the observed trend was specific to RNA-associated processes (Table S3). Additional GO enrichment of genes present within all conserved TAD boundaries revealed similar conservation patterns of RNA-associated terms in PANTHER, as well as g:Profiler and clusterProfiler (Figures S5 and S6).

**Figure 5:**
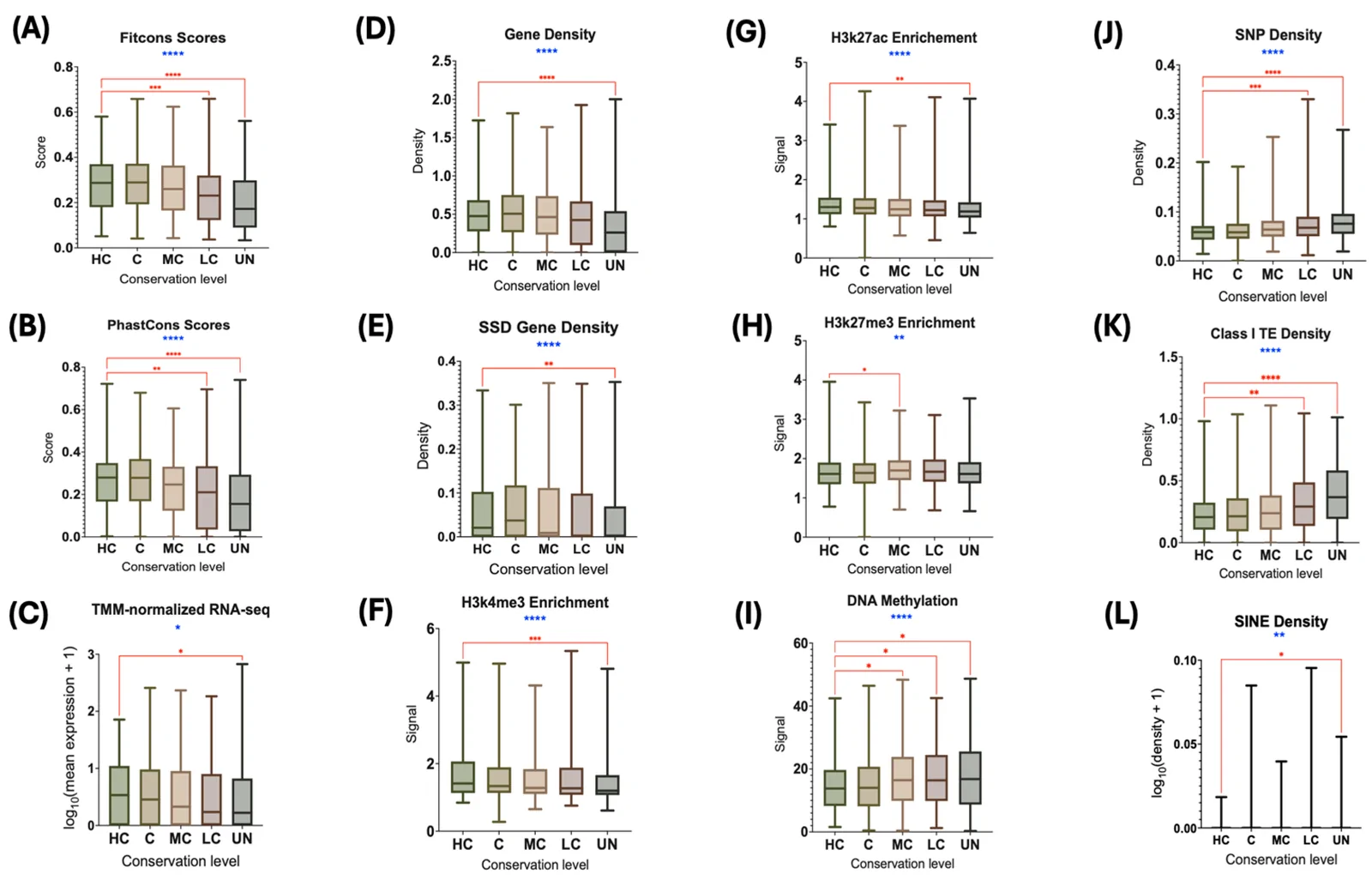
Category 1 analysis. Functional, structural, and epigenetic features measured across TAD boundaries in all conservation categories of the Nipponbare reference genome: (**A**) Fitcons score; (**B**) PhastCons score; (**C**) gene expression (TMM-normalized RNA-seq); (**D**) gene density; (**E**) small-scale duplication (SSD) density; (**F**) histone H3 lysine 4 trimethylation (H3K4me3) enrichment; (**G**) histone H3 lysine 27 acetylation (H3K27ac) enrichment; (**H**) histone H3 lysine 27 trimethylation (H3K27me3) enrichment; (**I**) DNA methylation; (**J**) SNP density; (**K**) retrotransposon (Class I transposon) density; and (**L**) short interspersed nuclear element (SINE) density. Overall statistical significance was assessed using the Kruskal–Wallis test (blue asterisks), followed by FDR-adjusted post-hoc Dunn’s multiple comparisons test between the highly conserved (HC) category and each remaining conservation category (red bars and asterisks). Only statistically significant pairwise comparisons are shown. Significance is depicted as * *p* < 0.05; ** *p* < 0.01; *** *p* < 0.001; **** *p* < 0.0001.

**Figure 6:**
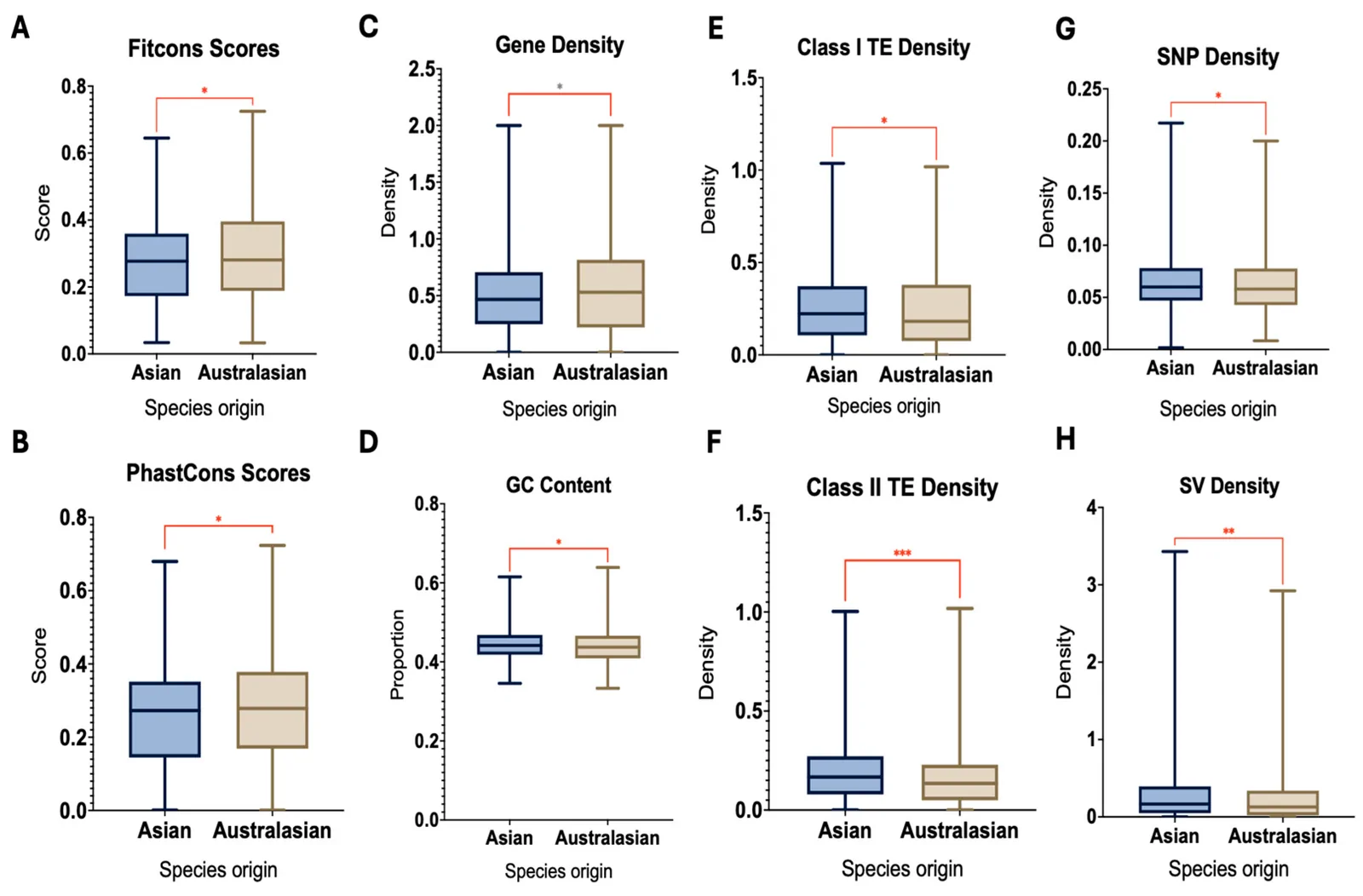
Category 2 analysis. Functional, structural, and epigenetic features measured across TAD boundaries between Asian (Nipponbare, Azucena, IR64, *O. rufipogon*) and Australasian (*O. meridionalis*) rice genomes: (**A**) Fitcons score; (**B**) PhastCons score; (**C**) gene density; (**D**) GC content; (**E**) retrotransposon (class I transposon) density; (**F**) DNA transposon (class 2 transposon) density; (**G**) SNP density; and (**H**) SV density. Statistical significance of Wilcoxon’s rank sum tests (red bars and asterisks) is depicted as * *p* < 0.05; ** *p* < 0.01; *** *p* < 0.001.

We next examined more than 20 genomic and epigenomic features across the Nipponbare genome and quantified their signals within TAD boundary regions of varying conservation levels and geographic origins (Figure 5 and Figure 6). Twelve features differed statistically significantly across conservation categories (Figure 5). Several features exhibited broad conservation-dependent patterns. Fitcons scores, PhastCons scores, gene density, gene expression, H3K27ac enrichment, and H3K4me3 enrichment (Figure 5A–D,F,G) generally exhibited higher values among more highly conserved boundaries and lower values among LC and UN boundaries, although the strength and consistency of this pattern varied across metrics. Fitcons and PhastCons scores showed particularly pronounced decreases toward lower conservation categories, while gene density and gene expression similarly exhibited lower median values in UN boundaries. H3K27ac and H3K4me3 enrichment displayed more modest differences across categories. Conversely, DNA methylation, SNP density, and Class I transposable element density (Figure 5I–K) generally increased toward lower conservation categories, with the highest median values observed among LC or UN boundaries. H3K27me3 enrichment (Figure 5H) was elevated in intermediate-to-low conservation categories, particularly MC and LC, but did not exhibit a consistent monotonic relationship with conservation. SSD gene density and SINE density also differed significantly across conservation categories (Figure 5E,L), although neither exhibited a clear monotonic relationship with conservation. Pairwise comparisons most frequently identified significant differences between HC boundaries and LC or UN boundaries. Together, these results demonstrate that multiple genomic and epigenomic features vary systematically across TAD boundary conservation categories.

Due to the previously observed result that *O. meridionalis* is 3D-genomically distinct from the other rice species studied here, we next aimed to juxtapose boundaries conserved across all rice species except *O. meridionalis* with boundaries unique to *O. meridionalis* across functional metrics. This allowed us to examine whether functional evolution was associated with species origin, as *O. meridionalis* is the sole Australasian rice species included in this study (Figure 6). Analyzing the same functional metrics, only eight were found to be statistically significant. Fitcons scores, PhastCons scores, and gene density (Figure 6A–C) were among the few metrics with higher values in *O. meridionalis* than in the Asian rice species, whereas retrotransposon (Class I TE), DNA transposon (Class II TE), SNP, and SV densities were higher in the Asian rice species than in the Australasian species (Figure 6E–H). GC content, although its median was slightly higher in the Asian species, was largely indistinguishable between groups but nevertheless exhibited a statistically significant difference (Figure 6D). These results demonstrate that multiple genomic features vary significantly with the evolutionary origin of TAD boundaries.

### 2.5. Phylogenetic and 3D-Genomic Relationships Among Rice Species

**Figure 7:**
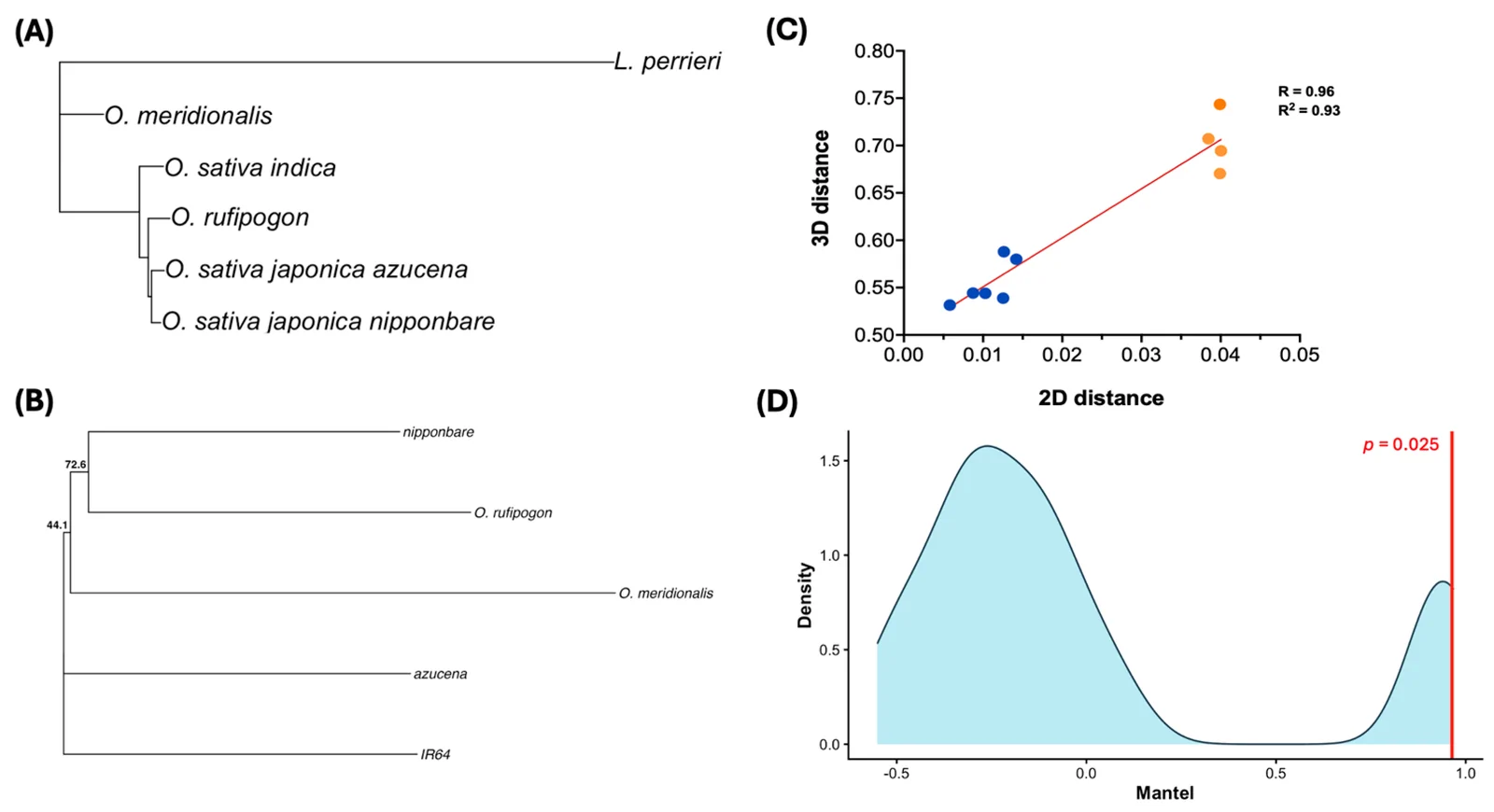
(**A**) Phylogenetic tree of the selected rice genomes constructed using genome-wide nucleotide similarity estimated from MASH distances. The wild relative of *Oryza sativa*, *Leersia perrieri* (*L. perrieri*), was included as the outgroup. Bootstrap analysis was performed to assess support for the inferred phylogenetic topology. (**B**) Phylogenetic tree of the selected rice genomes constructed using Jaccard distances as a proxy for three-dimensional genomic distance based on TAD boundary presence/absence similarity. Bootstrap analysis was performed with 1000 replicates (n = 5). The percentages 72.6% and 44.1% are the corresponding support values for the Nipponbare–*O. Rufipogon* and *O. meridionalis* clades, respectively. (**C**) Correlation between evolutionary distances derived from panel (**A**) and three-dimensional genomic distances derived from panel (**B**) for all pairwise species comparisons (n = 10). Orange data points represent pairwise comparisions including *O. meridionalis* on at least one axis, where blue data points map pairwise comparisons that do not include *O. meridionalis*. Linear regression analysis was performed and graphed (red line) to assess statistical significance (R = 0.96, R^2^ = 0.93). (**D**) Mantel test permutation density plot comparing pairwise evolutionary and three-dimensional genomic distance matrices (Panel (**C**)). The vertical red line indicates the observed Pearson correlation coefficient (r = 0.96), while the corresponding *p*-value (*p* = 0.025) is reported on the plot.

To better understand the evolutionary relationships between *Oryza* genomes derived from TAD boundary conservation, we used phylogenetic and statistical approaches to compare 1D- and 3D-genomic relationships (Figure 7). A 1D genomic phylogenetic tree representing canonical evolutionary relationships among rice species, with *L. perrieri* as the outgroup, was constructed using genome-wide nucleotide sequence similarity (Figure 7A). As shown, *O. meridionalis* was positioned on a separate clade from the other rice species, while Azucena and Nipponbare exhibited the greatest genetic similarity, followed by *O. rufipogon* and IR64. Using TAD boundary conservation data, we constructed a phylogenetic tree from an adapted Jaccard distance matrix (see Materials and Methods) to visualize evolutionary relationships based on 3D-genomic architecture (Figure 7B). As expected, *O. meridionalis* remained the most divergent species, branching earliest from the root, while the remaining four species separated into two pairs. Interestingly, Nipponbare clustered with *O. rufipogon*, whereas Azucena clustered with IR64, a topology that did not fully recapitulate previously observed evolutionary relationships [36,37]. Despite these topological differences, pairwise evolutionary and TAD boundary distances exhibited a strong positive relationship (R = 0.96, R^2^ = 0.93; Figure 7C). A Mantel test using Pearson correlation further supported this relationship, yielding a statistically significant Mantel correlation (r = 0.96, *p* = 0.025; Figure 7D). The permutation distribution was largely centered on negative correlation values, with its largest peak at approximately −0.25, whereas the observed Mantel statistic of 0.96 fell at the upper extreme of the distribution. Together, these phylogenetic and statistical results demonstrate a strong association between evolutionary divergence at the nucleotide-sequence level and divergence in TAD boundary organization.

## 3. Discussion

Our study aimed to assess the evolutionary divergence of five rice cultivars, three domesticated and two wild, through analysis of the functional, structural, and evolutionary conservation of their 3D genomic architecture, specifically TAD boundaries. Previous studies have pursued similar aims in mapping the 3D genome architecture of common rice cultivars, often examining pheno- and genotypic effects resulting from targeted mutagenesis [19,34,38,39,40,41]. Only Kurbidaeva et al. 2025 [19] has specifically incorporated a prominent evolutionary lens, an analytical focus on TAD boundaries as a genomic biomarker, or comprehensive statistical treatment of 3D genomic temporal trajectories, all of which we sought to accomplish here.

Our findings indicate a broadly similar TAD boundary conservation landscape among rice cultivars with similar 1D evolutionary histories, particularly Nipponbare, Azucena, and IR64 (indica), whereas the two wild landraces, *O. rufipogon* and, especially, *O. meridionalis*, show pronounced dissimilarities. *O. meridionalis* exhibited a marked decrease in conserved TAD boundary quantity, structural composition, and chromosomal distribution, accompanied by a larger amount of unique boundaries. Notably, *O. meridionalis* also reported the highest proportion of HC boundaries of any genome studied. Unique *O. meridionalis* TAD boundaries, representing the Australasian lineage, showed higher median FitCons and PhastCons scores than boundaries conserved among the four Asian cultivars. Together, we interpret these results as evidence for elevated retention of ancestral-like TAD boundary features in O. meridionalis, consistent with its position as the sister lineage to the other AA-genome *Oryza* species analyzed and its early divergence during the rapid radiation of the AA clade [36,37]. This retention of ancestral genomic characteristics may account for both its elevated HC boundary count and higher evolutionary conservation scores, despite an overall lower rate of cross-species boundary sharing. Our results also reveal a positive relationship between the TAD boundary conservation level and RNA metabolism-associated gene content in Nipponbare, suggesting that boundaries are conserved because they are functionally important: conserved boundaries disproportionately harbor housekeeping genes essential to growth, survival, and reproduction. This interpretation is reinforced by the inverse trend in mutagenesis-associated and heterochromatin-associated markers, including DNA methylation (which reduces chromatin accessibility), SNP density, repressive histone modifications, and TE density, all of which decline with an increasing conservation level, while evolutionary constraint scores, gene density, and gene expression track conservation levels progress in the same direction as the GO enrichment signal. Finally, while the 1D- and 3D-genomic phylogenetic topologies differ visually, their statistical correlation is strong, suggesting that 3D genomic evolution, modeled here as TAD boundary conservation, can act as a meaningful predictor of, or complement to, conventional nucleotide- or gene-based evolutionary metrics.

Our functional characterization of conserved TAD boundaries in Nipponbare closely parallels findings from Kurbidaeva et al. 2025 [19], the source study for our Micro-C data, which similarly reported that rice TAD boundaries show high transcriptional activity, low methylation levels, low transposable element content, and increased gene density. Our conservation-stratified analysis extends this observation by showing that these functional signatures are not uniform across all TAD boundaries, but instead scale directly with the degree of cross-species conservation, a distinction not resolved in the original study. Our finding that 3D-genomic distance closely tracks 1D sequence divergence is also consistent with Kurbidaeva et al.’s broader conclusion that global 3D genome evolution closely tracks sequence evolution in *Oryza*, lending independent support to that claim using a distinct boundary-conservation-based method (TCBF) rather than a direct topological comparison [19,35].

Our results also parallel informative differences and recent findings in mammalian systems. A comparative Hi-C/ChIP-seq study across four primate and four rodent species found that only 14% of human TAD boundaries were shared across all eight species (termed “ultraconserved”), while 15% were species-specific; ultraconserved boundaries showed stronger insulation strength, CTCF binding, and enrichment of older retrotransposons relative to species-specific boundaries [20]. The direction of the TE relationship in that study is worth flagging against our own: whereas we found that overall Class I TE density declines with increasing conservation level, the mammalian study associated ultraconserved boundaries with enrichment for specifically old, decayed retrotransposons rather than high overall TE burden. Together, this suggests a shared underlying principle: conserved boundaries tend to accumulate ancient, “domesticated” repetitive elements rather than young, potentially destabilizing ones, even though rice lacks the CTCF-based mechanism believed to anchor mammalian TAD boundaries. This raises the possibility that structurally analogous evolutionary pressures act on TAD boundaries across kingdoms via convergent, rather than homologous, mechanisms. Additionally, the histone modifications we found most strongly associated with conservation level (H3K4me3, H3K27me3) match the two markers that the TCBF developers themselves identified, using a deep neural network, as the key predictive features of TAD boundary formation [42], reinforcing that our conservation signal likely detects a biologically grounded phenomenon rather than a methodological artifact.

To our knowledge, this study represents the first application of a dedicated, tiered TAD boundary conservation framework (HC/C/MC/LC/Unique) to a multi-species plant system, and the first construction of a phylogenetic tree derived purely from TAD data in any plant genus. While prior work, including Kurbidaeva et al. 2025 [19], has established that rice TADs are functional genomic units and that global 3D genome divergence tracks sequence divergence, no previous plant study has stratified boundaries by conservation depth and tested each stratum against independent functional, epigenetic, and evolutionary metrics simultaneously. This tiered approach allowed us to detect a conservation-dependent functional gradient that a binary conserved/unconserved framework would likely have obscured, and to identify the distinct evolutionary signature of *O. meridionalis* with a level of resolution not previously available for wild *Oryza* relatives. Beyond its immediate findings, this work offers a template for using 3D genome architecture as a tractable, generalizable biomarker in comparative and evolutionary plant genomics, one that does not require species-specific CTCF-based frameworks, given its apparent absence in plants [20,43,44,45]. Because TAD boundaries in our analysis were preferentially associated with housekeeping and RNA-processing gene content, boundary conservation profiling may offer breeders a complementary lens for identifying regulatory regions under strong purifying selection, which are less likely to tolerate disruption during trait introgression or genome editing. As 3D genomic technologies become more accessible and cost-effective, this conservation-tiered framework could be extended to additional wild rice relatives or other domesticated crop lineages to help distinguish functionally constrained regulatory architecture from more evolutionarily flexible regions of the genome.

Several limitations warrant consideration. GO enrichment analysis was restricted to the Nipponbare reference genome because it is the only genome in this study with sufficiently complete, cross-validated functional annotation, drawing on RAP-DB, Oryzabase, and g:Profiler’s GMT resources, to construct a reliable background gene set; the remaining four genomes lack comparably mature annotation, and enrichment analysis performed against an incomplete or inconsistent background risks producing unreliable or spurious results. Future work incorporating improved annotation for Azucena, IR64, *O. rufipogon*, and *O. meridionalis* would allow this functional analysis to be extended across all five genomes. Second, our TCBF-derived conservation calls diverged substantially from those generated by the liftOver-based approach used in Kurbidaeva et al. 2025 [19], with TCBF identifying more LC/MC boundaries and liftOver identifying more C/HC boundaries. Because the two methods differ fundamentally in how they anchor cross-species homology (synteny-and-sequence-based clustering versus direct coordinate lift-over), only raw boundary counts were directly comparable in this study, and conservation-category assignments should not be treated as interchangeable across methods. Additionally, our 3D-genomic phylogeny was constructed solely from presence/absence (Jaccard) data across five taxa. Given the small number of taxa and the binary nature of the data, the resulting bootstrap support values should be interpreted cautiously. While the inferred relationships are broadly consistent with other analyses presented in this study, the phylogeny should be considered supportive rather than definitive evidence of evolutionary relationships. Future methodological work extending 3D-genomic conservation-based distance matrices from non-binarized data, implying that higher boundary content indicates increased evolutionary retention, would strengthen confidence in 3D-genomic phylogenetic topologies of this kind. Finally, a tool that correspondingly assesses cross-species TAD conservation, rather than their boundaries, would further strengthen evolutionary patterns and conclusions determined here and other studies with similar goals.

## 4. Materials and Methods

### 4.1. Primary Data Sourcing

Micro-C chromatin conformation datasets for the five *Oryza* genomes were retrieved from NCBI (GEO accession: PRJNA1121389) [19]. TADs were analyzed using HiCExplorer (v.3.7.2) through the established Dovetail Genomics (Scotts Valley, CA, USA) pipeline [46]. Full experimental procedures and associated metadata are described in the work of Kurbidaeva et al. 2025 [19]. Genome FASTA assemblies, gene annotation files (in GFF format), and gene structure files (in GTF format) for Azucena, IR64, *O. meridionalis*, and *O. rufipogon* were obtained from Ensembl Plants (v114), while the Nipponbare (reference genome) sequence (Os-Nipponbare-Reference-IRGSP-1.0), gene annotation file, and gene structure file were downloaded from RAP-DB.

### 4.2. Conserved TAD Boundary Detection

To quantify and analyze interspecific TAD boundary conservation across our five rice genomes, we employed a nascent, command-line-based bioinformatic tool, TCBF (Topologically associating domain Conservative Boundary Finder), following the protocols established by He et al. 2023 [35]. TCBF requires three input files per species for pre-processing: a FASTA sequence, a gene annotation file, and corresponding TAD coordinates. Coordinates may be obtained from any 3C-based assay; however, Hi-C or an equivalent, high-throughput method is preferred for higher-resolution boundary detection. TCBF allows user-defined TAD boundary minimum lengths to reduce spurious alignments, which allows the tool to remove and filter any TAD boundary not meeting the allocated size, which we set at 5000 base pairs (bp) to match the resolution of the original Micro-C dataset [19,35]. An adapted version of the TCBF workflow is available schematically (Figure S2). Output from TCBF provides conserved TAD boundaries across our five species, divided into specific conservation groups, which are arranged sequentially by orthogroup size (2 ≤ *n* ≤ 14), where *n* reflects the total number of conserved boundaries present in a conserved group (Table S1). Boundary coordinates are provided concomitantly (Table S2). Conservation categories are determined based on the number of species present in a given TAD boundary group: (highly conserved (HC) = 5 species, conserved (C) = 4 species, moderately conserved (MC) = 3 species, and least conserved (LC) = 2 species). Conservation categories apply to both the group itself, and the boundaries associated with it. Unique (UN) boundaries were defined as those present in a species’ genome yet not featured in any TAD boundary group. Conserved boundary data were plotted on a heatmap based on code provided by He et al. 2023 [35]; statistical significance was confirmed by Kruskal–Wallis test. Statistical significance for all other visualizations of TAD boundary conservation data obtained from TCBF was determined by chi-squared tests of independence, Fisher’s exact tests, or pairwise Welch’s *t*-tests.

### 4.3. Boundary Synteny and Ideogram Generation

We used the TCBF tool for collinear synteny plot generation based on input FASTA sequences and output unique and conserved TAD boundary coordinates. We visualized a 1 Mb sector of chromosome 5. The Nipponbare gene map (Assembly accession ID: GCF_001433935.1) from the UCSC Genome Browser [47] was overlaid onto the synteny track.

The R package *karyoploteR* was utilized for ideogram generation, as well as manually mapped highly conserved TAD boundary coordinates represented as small black regions. Centromeric regions were incorporated into the plot using previously reported coordinates [48,49].

### 4.4. GO Enrichment

We aimed to assess whether TAD boundaries, characterized by their conservation level, are differentially enriched for various functional categories. To maximize robustness, we employed three Gene Ontology (GO) tools: g:Profiler [50], clusterProfiler [51], and pantherDB [52]. All enrichment analyses were only performed for the reference genome (Nipponbare), but were performed across all conservation levels. Across all three platforms, we ran enrichment analyses for three ontologies: biological process (BP), molecular function (MF), and cellular component (CC). BEDTools was used to identify genes located within conserved TAD boundary regions based on the Nipponbare GFF file and conserved TAD boundary coordinates, which were already segregated into conservation levels. For g:Profiler, we downloaded and implemented the R package *gprofiler2*, in which we utilized the g:GOST function (with Benjamini–Hochberg FDR correction) for enrichment analysis and the gostplot function for Manhattan plot generation. For clusterProfiler, we utilized the enrichGO function (with Benjamini–Hochberg FDR correction) for our GO enrichment analyses. For pantherDB, we performed Fisher’s Exact PANTHER overrepresentation tests (with FDR correction) for each of our GO enrichment analyses and plotted using custom code. For all three GO platforms, we iterated two background gene sets: the total dataset of genes featured in all Nipponbare TAD boundaries and the total dataset of genes in the entire Nipponbare genome; both datasets were generated using BEDTools. We determined that the latter yielded more robust and statistically sound results compared to the former due to the increased sample size (*n* ≈ 33,000, *n* ≈ 10,500) and as such, should be used for all downstream analyses to maximize statistical rigor. Our background gene set was compiled of data from three sources: the Nipponbare GFF file from RAP-DB, a Nipponbare GFF file from Oryzabase, and the *Oryza sativa* japonica GMT file from g:Profiler; any duplicate genes across sources were removed. Enriched GO terms were filtered for redundancy and/or cellular specificity in all visualizations. The raw GO results are also provided for our PANTHER-based analyses (Table S3).

### 4.5. Functional Profiling

Functional genomics datasets for the Nipponbare genome, generated by the Purugganan Lab and used for downstream computational analyses, included: RNAPII mapping (PRO-seq), DNA methylation, Fitcons scoring, PhastCons scoring, histone modifications [H3K27ac, H3K27me3, H3K4me3], gene density, GC content, SNP density, SV breakpoint density, transposable element (TE) densities (including all retrotransposons, LTR TEs [Ty3-gypsy, Ty1-copia], non-LTR TEs [LINES, SINES], and DNA transposons), small-scale duplication (SSD) genes, whole-genome duplication (WGD) genes, and gene expression (RNA-seq) [19,53,54,55]. ATAC-seq data was extracted from NCBI (GEO accession: GSE144564) [56]. Functional analyses were performed for two different categorical frameworks: conservation levels (Category 1) and geographic origin (Category 2). For Category 1, we used pybedtools v.0.9.1, a Python wrapper for BEDTools [57] to analyze the enrichment of genetic and epigenetic features between TAD-associated conservation groups, to determine if any differential functional units exist between boundary conservation levels (including unique boundary conditions). Category 2 was designed to foreground the pronounced divergence of *O. meridionalis* by contrasting boundaries unique to *O. meridionalis* against boundaries conserved across all three *O. sativa* cultivars and *O. rufipogon*, which are more closely related at the 1D sequence level and originate from Asian, compared to the Australasian background of *O. meridionalis* [58]. Because all functional profiles are referenced to the Nipponbare coordinates, we used UCSC’s liftOver to map non-Nipponbare boundaries. We obtained chain files for pairs of repeat-masked genomes from Kurbidaeva et al. 2025 [19], which were generated using custom scripts based on the UCSC pipeline [47]. We then lifted over the genomic coordinates of our conserved Azucena, IR64, and *O. rufipogon,* and unique *O. meridionalis* TAD boundaries in Category 2 to the target (Nipponbare) genome with the liftOver tool. The minimum base overlap ratio was set at 0.20 [19], and percentages of successfully mapped and unmapped TAD boundaries across cultivars are provided (Table S5). For Category 1, Kruskal–Wallis tests were performed to determine overall statistical significance for each profile; metrics with significant *p*-values were then subject to FDR-adjusted Dunn’s multiple comparisons tests. For Category 2, Wilcoxon’s rank sum tests were performed for all pairwise comparisons between the groups, and metrics with insignificant *p*-values were excluded from further analyses.

### 4.6. Phylogeny and Multidimensional Evolutionary Comparisons

We generated phylogenetic trajectories using both one-dimensional sequence information and 3D chromatin architecture, allowing us to assess whether evolutionary distances inferred from genome structure align with those derived from the nucleotide sequence. For the sequence-based analysis, *Mash*, a command-line tool for a fast whole-genome comparison, was used to compute pairwise distances across all genomes [59]. Mash distances were computed using default parameters (k = 21, s = 1000), which estimate whole-genome similarity via MinHash-derived k-mer overlaps. The resulting 5 × 5 distance matrix was then used with the *ape* package in R to construct a neighbor-joining (NJ) phylogenetic tree.

To complement this, we sought to generate the first 3D genomic phylogeny, constructed entirely from chromatin topological information. TAD boundary conservation data from TCBF were binarized to indicate the presence (1) or absence (0) of conserved boundaries across genomes; this analysis disregarded the presence of multiple boundaries belonging to one species in a given conserved TAD boundary group, meaning that any boundary count greater than 0 was equally converted to a digit of 1 in the binary table. Pairwise Jaccard similarities (J) were calculated using the *vegan* package and subsequently converted to Jaccard distances (1 − J). A custom implementation using *ape* enabled NJ tree construction directly from this Jaccard-derived distance matrix. A resampling-based stability analysis was initially performed; however, with only five taxa, the permutation space was limited to 5! = 120 unique arrangements. This restricted the number of independent resamples available for robust support estimation and produced insufficiently stable branch-support values. To statistically compare the evolutionary patterns derived from the 1D and 3D analyses, a Mantel test using Pearson correlation was performed with the *vegan* package in R. In addition, both a density-versus-Mantel correlation plot and a Pearson correlation scatter plot were generated to visualize the relationship between the two distance matrices.

### 4.7. Statistical Analysis

All statistical analyses were performed in base R (v.4.5.1). Non-parametric statistical tests were used where appropriate, and significance was defined as *p* ≤ 0.05 unless otherwise noted.

### 4.8. Code Availability

Custom code used for data processing (TCBF) and analysis, statistical testing, and visualization in this study is publicly available on GitHub at ogeyman/TCBF_TAD-boundaries_rice_project (https://github.com/ogeyman/TCBF_TAD-boundaries_rice_project, accessed on 1 October 2026).

## 5. Conclusions

Here, we present the first three-dimensional genomic phylogeny constructed from TAD boundary conservation data, establishing a novel framework for evolutionary inference in plants that operate independently of, yet are strongly correlated with, canonical nucleotide-based approaches (Figure 7). This concordance validates 3D genome architecture as a biologically meaningful axis of evolutionary divergence, while the topological differences we observed between the 1D- and 3D-derived trees, most notably the discordant clustering of Nipponbare, *O. rufipogon*, Azucena, and IR64, suggest that TAD boundary evolution may capture regulatory or structural signal not fully explained by sequence divergence alone. *O. meridionalis* emerged as a consistent outlier across every level of analysis: it possessed the lowest proportion of conserved boundaries, the highest fraction of unique boundaries, and formed a distinct cluster in both PCA and phylogenetic reconstructions, reinforcing its status as the most 3D-genomically divergent species in our dataset and underscoring TAD architecture as a marker of deeper evolutionary separation between Asian and Australasian rice lineages. Beyond structural conservation, we identify a clear functional gradient: highly conserved TAD boundaries are consistently and specifically enriched for RNA metabolism and processing genes, with this enrichment declining in a stepwise fashion through conserved, moderately conserved, and least conserved categories. This pattern, paired with the parallel decline in FitCons and PhastCons scores, active histone markers, and gene density alongside conservation strength, suggests that evolutionary constraint on TAD boundaries is not incidental but reflects functional pressure to preserve regulatory architecture surrounding a specific, biologically coherent gene set. Collectively, these findings demonstrate that TAD boundary conservation is a tractable, biologically informative, and evolutionarily interpretable feature of plant genomes, one that can be systematically compared across species using accessible computational tools such as TCBF. As spatial genomic datasets become increasingly available for non-model and agriculturally important species, the framework established here offers a template for incorporating three-dimensional genome architecture into comparative and applied plant genomics, with particular relevance for identifying conserved regulatory elements that might otherwise be overlooked by sequence-based approaches alone.

## Data Availability

The publicly available datasets analyzed in this study are accessible through the repositories and accession numbers specified in the Materials and Methods section. The primary TAD boundary conservation datasets generated in this study and used for downstream analyses are provided as Supplementary Material. Additional processed data are available from the corresponding author.

## Supplementary Materials

The following supporting information can be downloaded at https://www.mdpi.com/article/10.3390/ijms27208979/s1.
