# Supplementary material for: Evolutionary and Functional Conservation of Topologically Associating Domain Boundaries in Rice Genomes
Source: Int J Mol Sci. 2026 Oct 10;27(20):8979. doi: 10.3390/ijms27208979 (PMC13704375; doi:10.3390/ijms27208979)
Supplement: Supplementary file 1 [file ijms-27-08979-s001.zip › Sup_Figures.pdf]

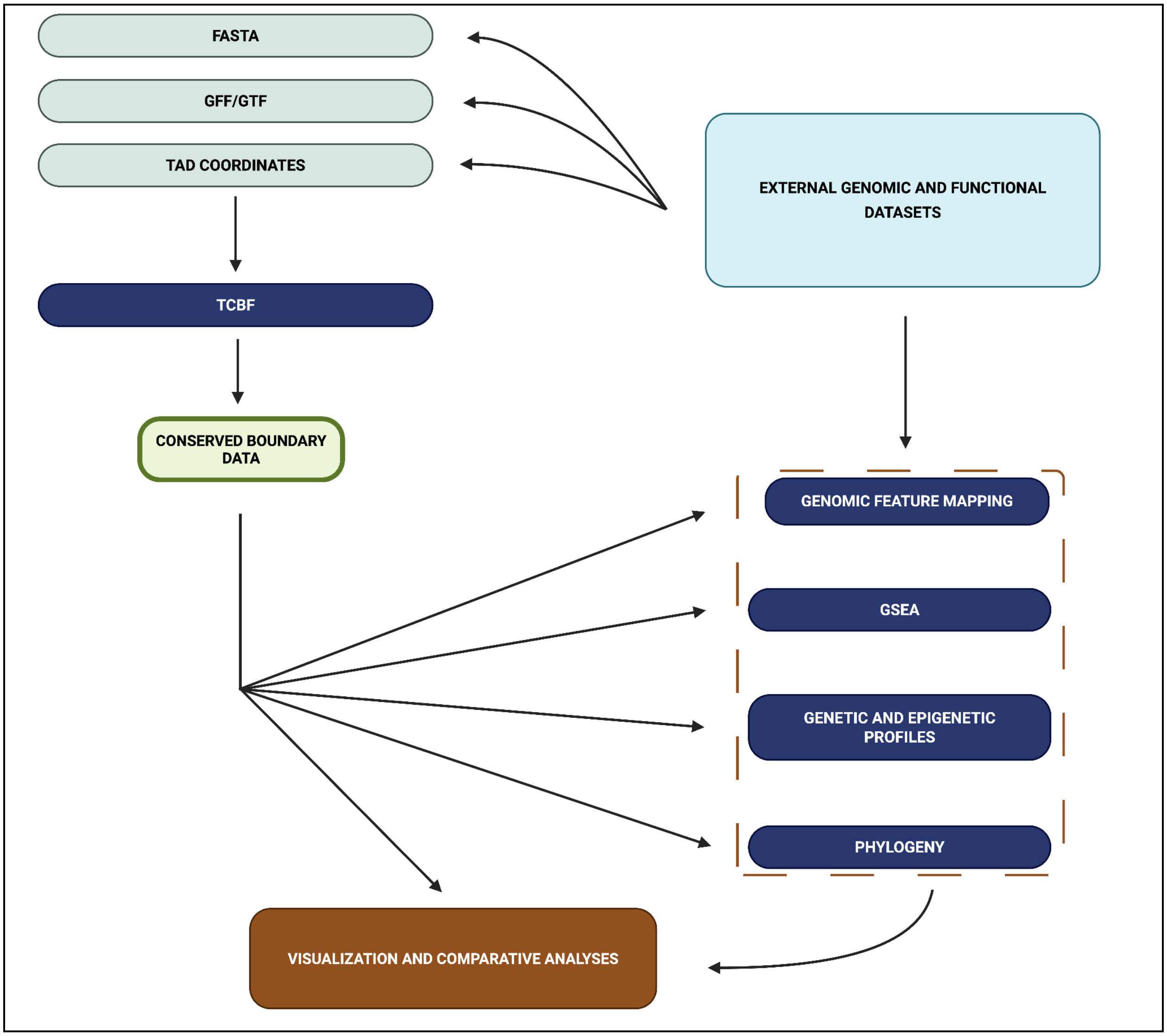

Supplemental Figure S1: Schematic overview of the study workflow. Additional technical details are provided in the Methods.

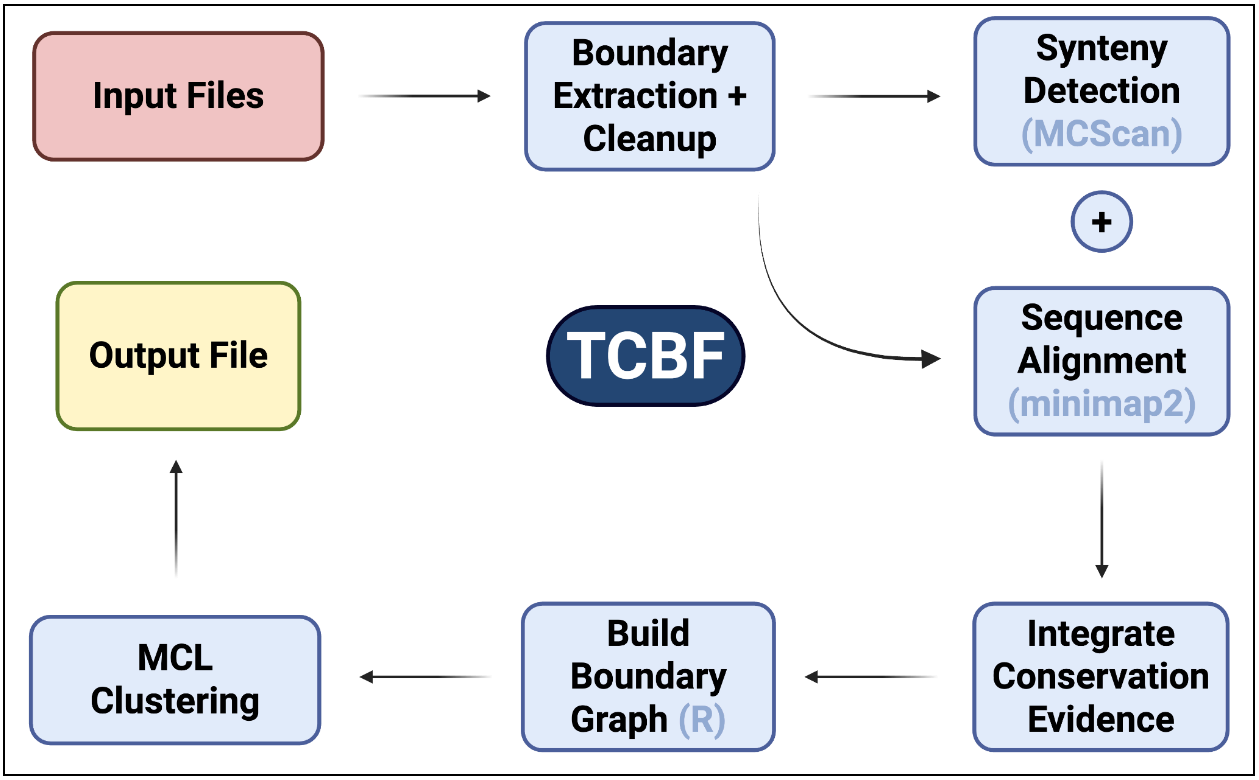

Supplemental Figure S2: Adapted schematic of the Topologically Associating Domain Conservative Boundary Finder (TCBF) workflow, based on He et al. 2022 [35].

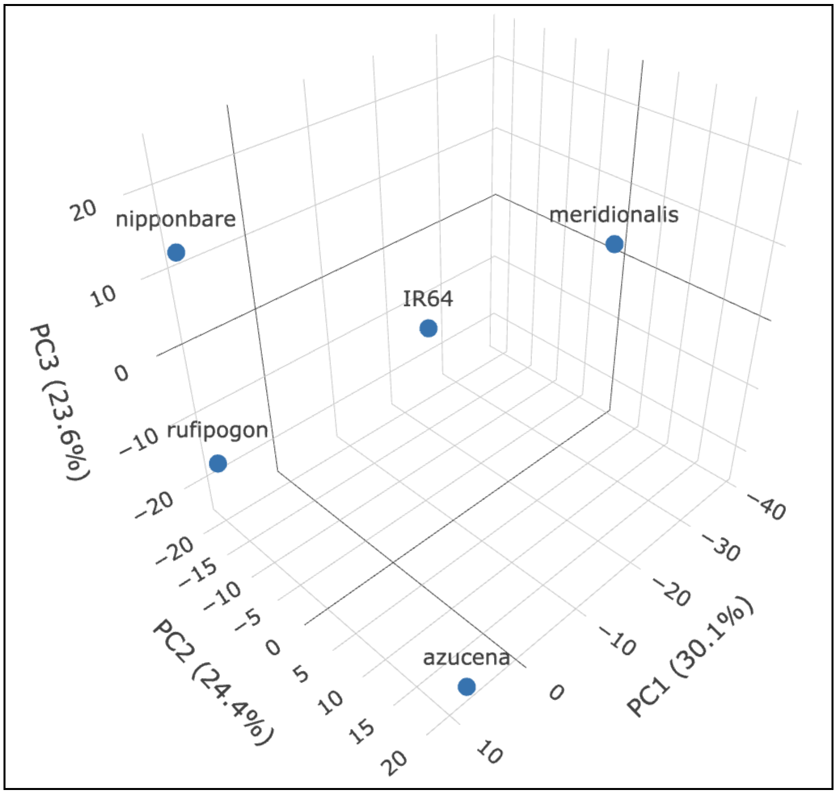

Supplemental Figure S3: Three-dimensional principal component analysis (PCA) of interspecific TAD boundary-group conservation data. The plot was generated using the R package *plotly*.

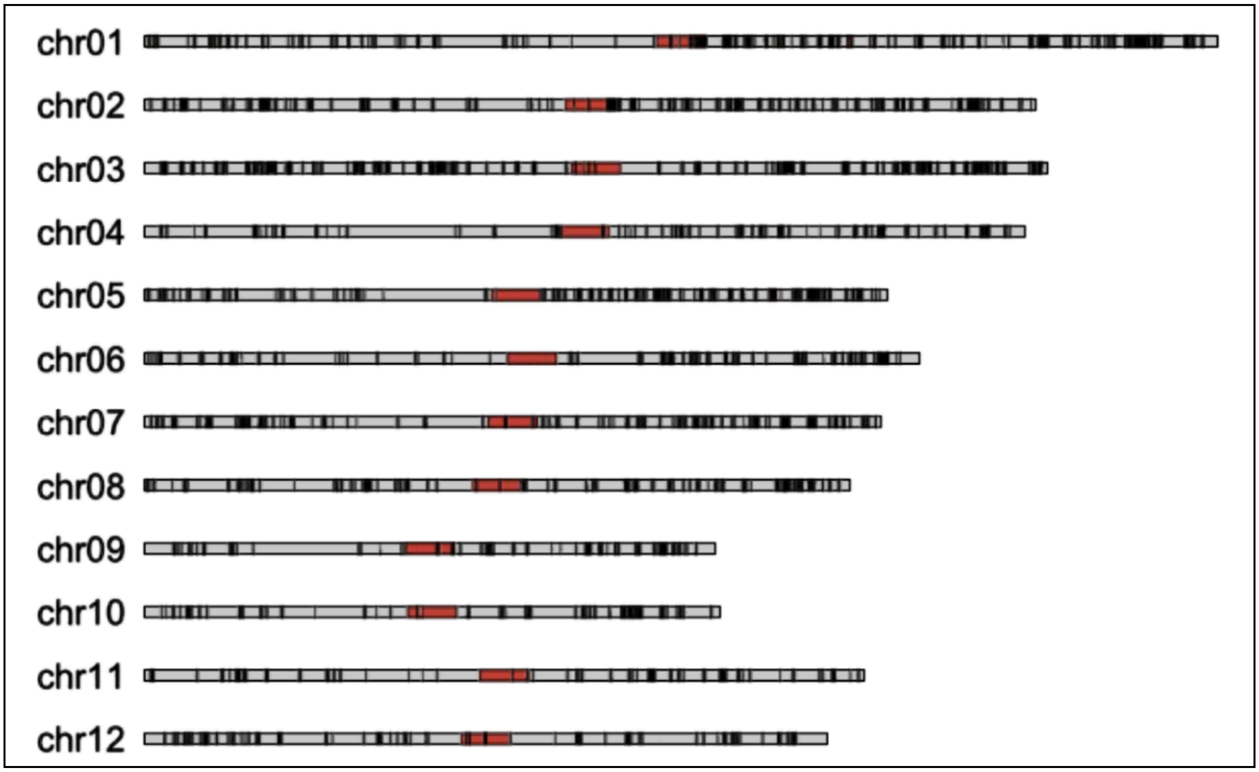

Supplemental Figure S4: Ideogram showing the chromosomal localization of all conserved TAD boundaries in the Nipponbare genome. Black bars represent conserved TAD boundaries, and red regions denote centromeric regions of each chromosome.

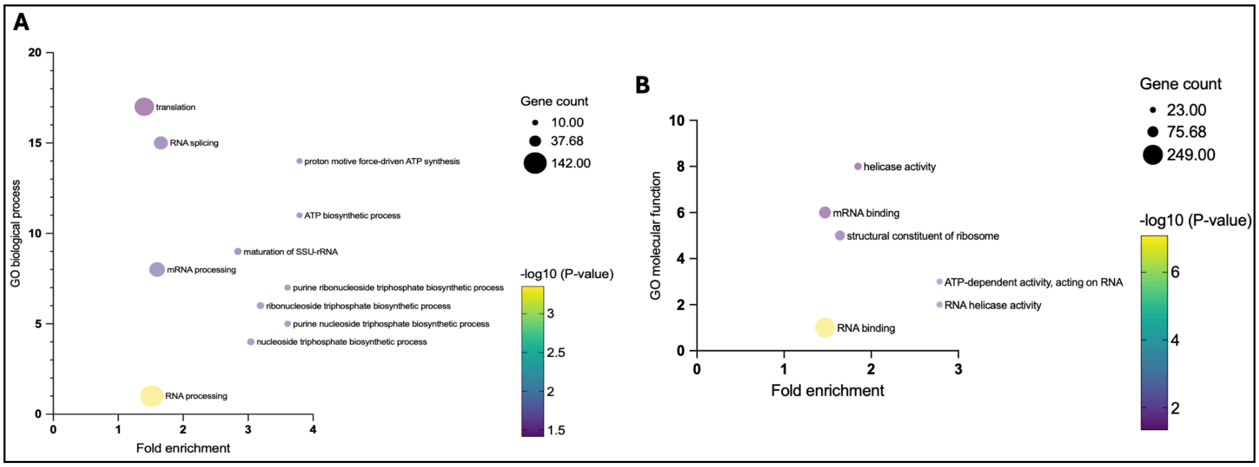

Supplemental Figure S5: Gene Ontology (GO) enrichment plots generated using g:Profiler. Data were obtained from the respective platforms and independently visualized. **(A)** GO BP enrichment from g:Profiler for all conserved boundaries; **(B)** GO Molecular Function (MF) enrichment from g:Profiler for all conserved boundaries.

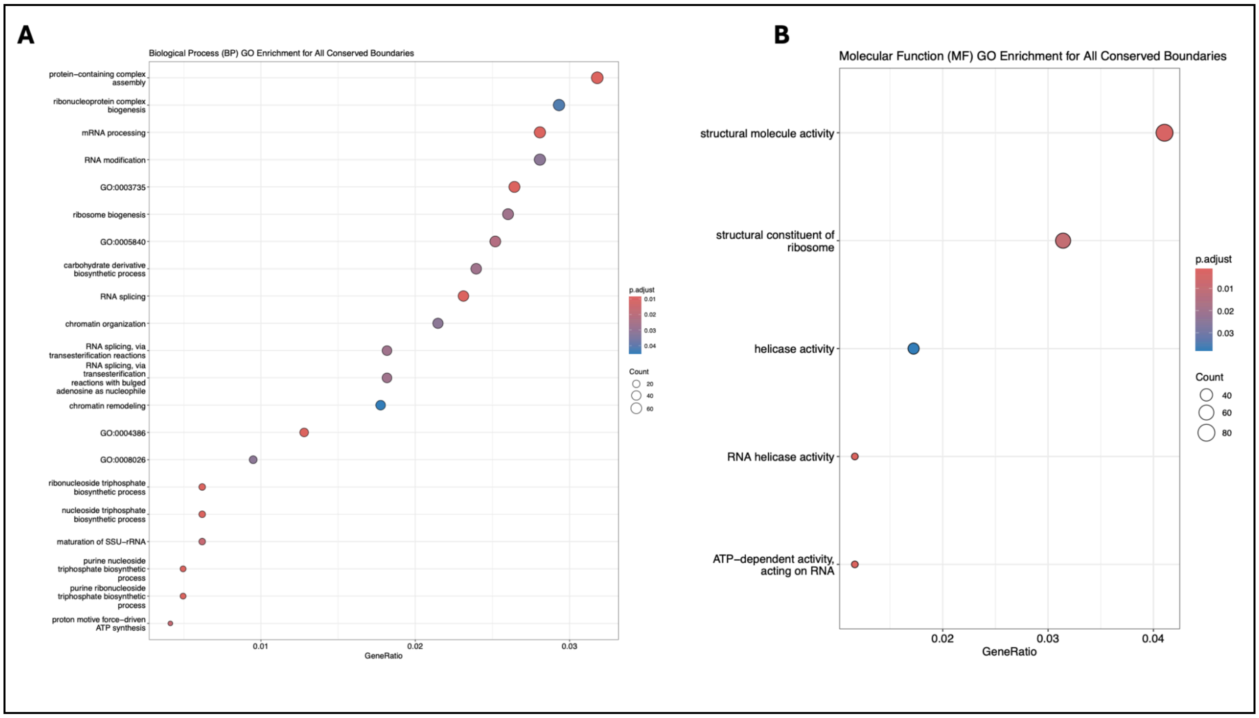

Supplemental Figure S6: Gene Ontology (GO) enrichment plots generated using clusterProfiler. **(A)** GO Biological Process (BP) enrichment for all conserved boundaries; **(B)** GO Molecular Function (MF) enrichment for all conserved boundaries.
