# Supplementary material for: Evolutionary and Functional Conservation of Topologically Associating Domain Boundaries in Rice Genomes
Source: Int J Mol Sci. 2026 Oct 10;27(20):8979. doi: 10.3390/ijms27208979 (PMC13704375; doi:10.3390/ijms27208979)
Supplement: Supplementary file 1 [file ijms-27-08979-s001.zip › Sup_Table_S1.pdf]

Supplemental Table S1: TAD boundary conservation groups across all (5) Oryza species

Group	O. meridionalis	Azucena	O. rufipogon
Group_1	meridionalis_boundary_103;meridionalis_boundary_104;meridionalis_boundary_746	azucena_boundary_126;azucena_boundary_127	rufipogon_boundary_121;rufipogon_boundary_122;rufipogon_boundary_123
Group_2	meridionalis_boundary_286;meridionalis_boundary_287;meridionalis_boundary_288	azucena_boundary_295;azucena_boundary_296;azucena_boundary_297	rufipogon_boundary_311
Group_3	meridionalis_boundary_352;meridionalis_boundary_157;meridionalis_boundary_158	azucena_boundary_361;azucena_boundary_362	rufipogon_boundary_364;rufipogon_boundary_363;rufipogon_boundary_365
Group_4	meridionalis_boundary_89;meridionalis_boundary_90	azucena_boundary_110;azucena_boundary_111	rufipogon_boundary_107;rufipogon_boundary_106;rufipogon_boundary_108
Group_5	meridionalis_boundary_969	azucena_boundary_1047;azucena_boundary_1048;azucena_boundary_1049	rufipogon_boundary_971;rufipogon_boundary_972
Group_6	meridionalis_boundary_1405;meridionalis_boundary_1406;meridionalis_boundary_1407;meridionalis_boundary_1408	azucena_boundary_1471;azucena_boundary_1470	rufipogon_boundary_1361
Group_7	meridionalis_boundary_285;meridionalis_boundary_284	azucena_boundary_654	rufipogon_boundary_307;rufipogon_boundary_308;rufipogon_boundary_606
Group_8	meridionalis_boundary_511	azucena_boundary_567;azucena_boundary_568	rufipogon_boundary_537;rufipogon_boundary_538;rufipogon_boundary_539
Group_9	meridionalis_boundary_55	azucena_boundary_54;azucena_boundary_52;azucena_boundary_53	rufipogon_boundary_54;rufipogon_boundary_55
Group_10		azucena_boundary_141;azucena_boundary_142	rufipogon_boundary_138;rufipogon_boundary_139
Group_11		azucena_boundary_1494;azucena_boundary_1495;azucena_boundary_1496;azucena_boundary_1497	rufipogon_boundary_1371;rufipogon_boundary_1370;rufipogon_boundary_1372
Group_12	meridionalis_boundary_1107;meridionalis_boundary_1108;meridionalis_boundary_1109	azucena_boundary_1180;azucena_boundary_1181	rufipogon_boundary_1104;rufipogon_boundary_1105
Group_13	meridionalis_boundary_1357;meridionalis_boundary_471;meridionalis_boundary_472	azucena_boundary_1510;azucena_boundary_1511	rufipogon_boundary_1389;rufipogon_boundary_1390
Group_14	meridionalis_boundary_152;meridionalis_boundary_153	azucena_boundary_172	rufipogon_boundary_168;rufipogon_boundary_169
Group_15	meridionalis_boundary_722	azucena_boundary_788;azucena_boundary_789	rufipogon_boundary_743;rufipogon_boundary_744
Group_16	meridionalis_boundary_855;meridionalis_boundary_856;meridionalis_boundary_857;meridionalis_boundary_858	azucena_boundary_921;azucena_boundary_922;azucena_boundary_920	rufipogon_boundary_859;rufipogon_boundary_860
Group_17	meridionalis_boundary_925;meridionalis_boundary_924	azucena_boundary_979;azucena_boundary_978	rufipogon_boundary_907;rufipogon_boundary_906
Group_18		azucena_boundary_184;azucena_boundary_185;azucena_boundary_186	rufipogon_boundary_181;rufipogon_boundary_182
Group_19	meridionalis_boundary_105;meridionalis_boundary_106	azucena_boundary_130;azucena_boundary_131;azucena_boundary_132	rufipogon_boundary_126
Group_20	meridionalis_boundary_1227;meridionalis_boundary_1228	azucena_boundary_1292	rufipogon_boundary_1197
Group_21		azucena_boundary_1398;azucena_boundary_1504;azucena_boundary_1397	rufipogon_boundary_1385;rufipogon_boundary_1386
Group_22	meridionalis_boundary_1451	azucena_boundary_1395;azucena_boundary_1503	rufipogon_boundary_1383
Group_23	meridionalis_boundary_169;meridionalis_boundary_170;meridionalis_boundary_171	azucena_boundary_190;azucena_boundary_191	rufipogon_boundary_187
Group_24	meridionalis_boundary_360	azucena_boundary_371;azucena_boundary_369;azucena_boundary_370	rufipogon_boundary_371
Group_25	meridionalis_boundary_371;meridionalis_boundary_372;meridionalis_boundary_373	azucena_boundary_380;azucena_boundary_381	rufipogon_boundary_374
Group_26	meridionalis_boundary_375;meridionalis_boundary_376;meridionalis_boundary_378	azucena_boundary_382	rufipogon_boundary_379;rufipogon_boundary_378
Group_27	meridionalis_boundary_381	azucena_boundary_1376;azucena_boundary_1377	rufipogon_boundary_1284;rufipogon_boundary_1283
Group_28	meridionalis_boundary_402	azucena_boundary_412;azucena_boundary_411	rufipogon_boundary_414;rufipogon_boundary_413
Group_29	meridionalis_boundary_436	azucena_boundary_440;azucena_boundary_441	rufipogon_boundary_441;rufipogon_boundary_442;rufipogon_boundary_443
Group_30	meridionalis_boundary_437;meridionalis_boundary_438	azucena_boundary_442;azucena_boundary_443	rufipogon_boundary_444;rufipogon_boundary_445
Group_31	meridionalis_boundary_734	azucena_boundary_803;azucena_boundary_802	rufipogon_boundary_753
Group_32	meridionalis_boundary_945;meridionalis_boundary_946	azucena_boundary_1000;azucena_boundary_999	
Group_33	meridionalis_boundary_96;meridionalis_boundary_97;meridionalis_boundary_484;meridionalis_boundary_485	azucena_boundary_122	rufipogon_boundary_117
Group_34	meridionalis_boundary_971;meridionalis_boundary_972	azucena_boundary_1052;azucena_boundary_1053	rufipogon_boundary_975
Group_35	meridionalis_boundary_122	azucena_boundary_798	rufipogon_boundary_146;rufipogon_boundary_147;rufipogon_boundary_750
Group_36	meridionalis_boundary_844;meridionalis_boundary_845		rufipogon_boundary_840;rufipogon_boundary_839
Group_37	meridionalis_boundary_336	azucena_boundary_344;azucena_boundary_345	rufipogon_boundary_354;rufipogon_boundary_355;rufipogon_boundary_795
Group_38		azucena_boundary_1448;azucena_boundary_1449	rufipogon_boundary_1342;rufipogon_boundary_1343
Group_39		azucena_boundary_1481;azucena_boundary_1482	rufipogon_boundary_1363;rufipogon_boundary_1364
Group_40		azucena_boundary_625	rufipogon_boundary_585;rufipogon_boundary_587;rufipogon_boundary_586
Group_41		azucena_boundary_824;azucena_boundary_121	rufipogon_boundary_764
Group_42		azucena_boundary_94;azucena_boundary_95;azucena_boundary_96	rufipogon_boundary_94;rufipogon_boundary_95
Group_43	meridionalis_boundary_1218	azucena_boundary_1283;azucena_boundary_1197	rufipogon_boundary_1193;rufipogon_boundary_1120
Group_44	meridionalis_boundary_13;meridionalis_boundary_12	azucena_boundary_8	rufipogon_boundary_9
Group_45	meridionalis_boundary_1419	azucena_boundary_1485;azucena_boundary_1486	rufipogon_boundary_1368;rufipogon_boundary_1369
Group_46	meridionalis_boundary_145	azucena_boundary_163;azucena_boundary_773;azucena_boundary_774;azucena_boundary_164	rufipogon_boundary_162
Group_47	meridionalis_boundary_1449	azucena_boundary_1514;azucena_boundary_1515	
Group_48	meridionalis_boundary_147	azucena_boundary_771	rufipogon_boundary_731;rufipogon_boundary_164
Group_49	meridionalis_boundary_1486;meridionalis_boundary_1487	azucena_boundary_1591	rufipogon_boundary_1464;rufipogon_boundary_1463
Group_50	meridionalis_boundary_297	azucena_boundary_302	rufipogon_boundary_615
Group_51	meridionalis_boundary_353	azucena_boundary_363	rufipogon_boundary_366
Group_52	meridionalis_boundary_433;meridionalis_boundary_434	azucena_boundary_435;azucena_boundary_436;azucena_boundary_437	rufipogon_boundary_437
Group_53	meridionalis_boundary_455	azucena_boundary_500;azucena_boundary_501;azucena_boundary_502	rufipogon_boundary_498;rufipogon_boundary_499
Group_54	meridionalis_boundary_853;meridionalis_boundary_854	azucena_boundary_918;azucena_boundary_919	rufipogon_boundary_857;rufipogon_boundary_858
Group_55	meridionalis_boundary_906	azucena_boundary_956;azucena_boundary_957	rufipogon_boundary_883
Group_56	meridionalis_boundary_912;meridionalis_boundary_911	azucena_boundary_969	rufipogon_boundary_895
Group_57	meridionalis_boundary_327;meridionalis_boundary_328;meridionalis_boundary_329		rufipogon_boundary_342;rufipogon_boundary_343
Group_58	meridionalis_boundary_740;meridionalis_boundary_741		rufipogon_boundary_757
Group_59	meridionalis_boundary_785;meridionalis_boundary_786;meridionalis_boundary_787;meridionalis_boundary_788		rufipogon_boundary_781;rufipogon_boundary_782
Group_60	meridionalis_boundary_1055;meridionalis_boundary_1056		
Group_61	meridionalis_boundary_165	azucena_boundary_173;azucena_boundary_174	rufipogon_boundary_170;rufipogon_boundary_171
Group_62	meridionalis_boundary_725;meridionalis_boundary_726	azucena_boundary_791	rufipogon_boundary_745
Group_63		azucena_boundary_1062;azucena_boundary_1063	rufipogon_boundary_982;rufipogon_boundary_983
Group_64		azucena_boundary_1580;azucena_boundary_1581	rufipogon_boundary_1455;rufipogon_boundary_1456
Group_65		azucena_boundary_495;azucena_boundary_496	rufipogon_boundary_495;rufipogon_boundary_496
Group_66		azucena_boundary_877;azucena_boundary_878	rufipogon_boundary_813;rufipogon_boundary_812;rufipogon_boundary_814;rufip
Group_67		azucena_boundary_966;azucena_boundary_967;azucena_boundary_968	rufipogon_boundary_890;rufipogon_boundary_891
Group_68		azucena_boundary_1303	rufipogon_boundary_1221;rufipogon_boundary_1222
Group_69	meridionalis_boundary_1008;meridionalis_boundary_1009	azucena_boundary_1082	
Group_70	meridionalis_boundary_1052;meridionalis_boundary_1053	azucena_boundary_1106	
Group_71	meridionalis_boundary_1088;meridionalis_boundary_1089	azucena_boundary_1150	rufipogon_boundary_1068;rufipogon_boundary_1067
Group_72	meridionalis_boundary_1098;meridionalis_boundary_1097	azucena_boundary_1163	rufipogon_boundary_1086;rufipogon_boundary_1087
Group_73	meridionalis_boundary_1212	azucena_boundary_1273;azucena_boundary_1274	rufipogon_boundary_1184;rufipogon_boundary_1185
Group_74	meridionalis_boundary_1216	azucena_boundary_1277;azucena_boundary_1278	
Group_75	meridionalis_boundary_1223;meridionalis_boundary_1224	azucena_boundary_1291	
Group_76	meridionalis_boundary_1235;meridionalis_boundary_1236	azucena_boundary_1296;azucena_boundary_1297	rufipogon_boundary_1211;rufipogon_boundary_1212
Group_77	meridionalis_boundary_131	azucena_boundary_149;azucena_boundary_784;azucena_boundary_785;azucena_boundary_151	rufipogon_boundary_741
Group_78	meridionalis_boundary_14	azucena_boundary_9;azucena_boundary_10	rufipogon_boundary_10;rufipogon_boundary_11
Group_79	meridionalis_boundary_308;meridionalis_boundary_309	azucena_boundary_313;azucena_boundary_314	rufipogon_boundary_331
Group_80	meridionalis_boundary_311;meridionalis_boundary_312	azucena_boundary_316;azucena_boundary_317;azucena_boundary_315	
Group_81	meridionalis_boundary_316	azucena_boundary_323;azucena_boundary_324	
Group_82	meridionalis_boundary_354	azucena_boundary_364;azucena_boundary_365	rufipogon_boundary_367
Group_83	meridionalis_boundary_473	azucena_boundary_529;azucena_boundary_530	rufipogon_boundary_511;rufipogon_boundary_512
Group_84	meridionalis_boundary_496;meridionalis_boundary_495	azucena_boundary_549	rufipogon_boundary_529
Group_85	meridionalis_boundary_6	azucena_boundary_7	rufipogon_boundary_7;rufipogon_boundary_8
Group_86	meridionalis_boundary_607;meridionalis_boundary_608	azucena_boundary_672;azucena_boundary_673	rufipogon_boundary_627
Group_87	meridionalis_boundary_677	azucena_boundary_714;azucena_boundary_715;azucena_boundary_716	rufipogon_boundary_660
Group_88	meridionalis_boundary_678	azucena_boundary_717	rufipogon_boundary_661;rufipogon_boundary_662;rufipogon_boundary_663
Group_89	meridionalis_boundary_731;meridionalis_boundary_732	azucena_boundary_800	rufipogon_boundary_751
Group_90	meridionalis_boundary_864	azucena_boundary_925;azucena_boundary_926	rufipogon_boundary_862;rufipogon_boundary_863
Group_91	meridionalis_boundary_872	azucena_boundary_929;azucena_boundary_928	rufipogon_boundary_867
Group_92	meridionalis_boundary_879;meridionalis_boundary_880	azucena_boundary_936	
Group_93	meridionalis_boundary_898	azucena_boundary_948;azucena_boundary_949	rufipogon_boundary_873
Group_94	meridionalis_boundary_918	azucena_boundary_974;azucena_boundary_975	
Group_95	meridionalis_boundary_920	azucena_boundary_976	rufipogon_boundary_902
Group_96	meridionalis_boundary_974;meridionalis_boundary_975	azucena_boundary_1056;azucena_boundary_1057	
Group_97	meridionalis_boundary_1370		rufipogon_boundary_1316;rufipogon_boundary_1334
Group_98	meridionalis_boundary_186	azucena_boundary_205	rufipogon_boundary_204;rufipogon_boundary_205
Group_99	meridionalis_boundary_201;meridionalis_boundary_202;meridionalis_boundary_200		rufipogon_boundary_219
Group_100	meridionalis_boundary_292	azucena_boundary_300;azucena_boundary_657	rufipogon_boundary_314;rufipogon_boundary_612
Group_101	meridionalis_boundary_435		rufipogon_boundary_438;rufipogon_boundary_439
Group_102	meridionalis_boundary_794;meridionalis_boundary_795		rufipogon_boundary_784
Group_103	meridionalis_boundary_883	azucena_boundary_940;azucena_boundary_941;azucena_boundary_939	rufipogon_boundary_869
Group_104	meridionalis_boundary_926	azucena_boundary_988;azucena_boundary_989	rufipogon_boundary_917;rufipogon_boundary_916
Group_105	meridionalis_boundary_1059;meridionalis_boundary_1060		
Group_106	meridionalis_boundary_1210;meridionalis_boundary_1211	azucena_boundary_1271	rufipogon_boundary_1182;rufipogon_boundary_1183
Group_107	meridionalis_boundary_1429;meridionalis_boundary_1430		
Group_108	meridionalis_boundary_334	azucena_boundary_339;azucena_boundary_340	rufipogon_boundary_347;rufipogon_boundary_348
Group_109	meridionalis_boundary_400;meridionalis_boundary_401	azucena_boundary_399	rufipogon_boundary_401
Group_110	meridionalis_boundary_742;meridionalis_boundary_743	azucena_boundary_816	rufipogon_boundary_758
Group_111	meridionalis_boundary_10;meridionalis_boundary_7;meridionalis_boundary_8;meridionalis_boundary_9		
Group_112	meridionalis_boundary_1012	azucena_boundary_1328;azucena_boundary_1327	rufipogon_boundary_1250
Group_113	meridionalis_boundary_935	azucena_boundary_1194	rufipogon_boundary_1119;rufipogon_boundary_1191
Group_114		azucena_boundary_1168;azucena_boundary_1169	rufipogon_boundary_1093;rufipogon_boundary_1094
Group_115		azucena_boundary_117	rufipogon_boundary_111;rufipogon_boundary_766
Group_116		azucena_boundary_1202;azucena_boundary_1203	rufipogon_boundary_1127
Group_117		azucena_boundary_1250;azucena_boundary_1249	rufipogon_boundary_1167;rufipogon_boundary_1166
Group_118		azucena_boundary_147;azucena_boundary_148	rufipogon_boundary_144
Group_119		azucena_boundary_1529	rufipogon_boundary_1411;rufipogon_boundary_1412
Group_120		azucena_boundary_391;azucena_boundary_392	rufipogon_boundary_395;rufipogon_boundary_396
Group_121		azucena_boundary_404	rufipogon_boundary_403;rufipogon_boundary_404;rufipogon_boundary_405
Group_122		azucena_boundary_556	rufipogon_boundary_533;rufipogon_boundary_532
Group_123		azucena_boundary_563;azucena_boundary_564	rufipogon_boundary_535
Group_124		azucena_boundary_593;azucena_boundary_594	rufipogon_boundary_555;rufipogon_boundary_556
Group_125		azucena_boundary_617	rufipogon_boundary_580;rufipogon_boundary_581;rufipogon_boundary_582;rufip
Group_126		azucena_boundary_814;azucena_boundary_815	rufipogon_boundary_756

Group_127		azucena_boundary_911	rufipogon_boundary_846;rufipogon_boundary_845
Group_128		azucena_boundary_991;azucena_boundary_992	rufipogon_boundary_919;rufipogon_boundary_935
Group_129		azucena_boundary_1269;azucena_boundary_1270	rufipogon_boundary_1180;rufipogon_boundary_1181
Group_130		azucena_boundary_697	rufipogon_boundary_643
Group_131		azucena_boundary_534;azucena_boundary_535	rufipogon_boundary_518
Group_132	meridionalis_boundary_1007	azucena_boundary_1081	
Group_133	meridionalis_boundary_1037;meridionalis_boundary_1038	azucena_boundary_1097	
Group_134	meridionalis_boundary_107;meridionalis_boundary_108	azucena_boundary_133	rufipogon_boundary_130
Group_135	meridionalis_boundary_1090	azucena_boundary_1151	rufipogon_boundary_1072;rufipogon_boundary_1073
Group_136	meridionalis_boundary_1099	azucena_boundary_1164	rufipogon_boundary_1088;rufipogon_boundary_1169
Group_137	meridionalis_boundary_1106	azucena_boundary_1178;azucena_boundary_1179	
Group_138	meridionalis_boundary_115;meridionalis_boundary_116;meridionalis_boundary_117		rufipogon_boundary_141
Group_139	meridionalis_boundary_1219	azucena_boundary_1284;azucena_boundary_1285	
Group_140	meridionalis_boundary_1300	azucena_boundary_1364;azucena_boundary_1365	rufipogon_boundary_1267
Group_141	meridionalis_boundary_1303	azucena_boundary_1371	rufipogon_boundary_1276
Group_142	meridionalis_boundary_1305;meridionalis_boundary_1306	azucena_boundary_1373	rufipogon_boundary_1278
Group_143	meridionalis_boundary_136;meridionalis_boundary_137	azucena_boundary_152	
Group_144	meridionalis_boundary_140	azucena_boundary_160	rufipogon_boundary_161
Group_145	meridionalis_boundary_1420	azucena_boundary_1487;azucena_boundary_1488	
Group_146	meridionalis_boundary_143;meridionalis_boundary_144	azucena_boundary_162	
Group_147	meridionalis_boundary_1447	azucena_boundary_1404;azucena_boundary_1508;azucena_boundary_1507	
Group_148		azucena_boundary_1544;azucena_boundary_1542;azucena_boundary_1543	rufipogon_boundary_1416;rufipogon_boundary_1417
Group_149	meridionalis_boundary_1489	azucena_boundary_1589;azucena_boundary_1588	
Group_150	meridionalis_boundary_208	azucena_boundary_215;azucena_boundary_942	rufipogon_boundary_225;rufipogon_boundary_226
Group_151	meridionalis_boundary_244	azucena_boundary_240	rufipogon_boundary_252
Group_152	meridionalis_boundary_258;meridionalis_boundary_259	azucena_boundary_270	rufipogon_boundary_289
Group_153		azucena_boundary_293;azucena_boundary_294	rufipogon_boundary_310;rufipogon_boundary_309
Group_154	meridionalis_boundary_290	azucena_boundary_299	rufipogon_boundary_313
Group_155	meridionalis_boundary_305	azucena_boundary_310	rufipogon_boundary_325;rufipogon_boundary_324
Group_156	meridionalis_boundary_315;meridionalis_boundary_616	azucena_boundary_679	rufipogon_boundary_629
Group_157	meridionalis_boundary_320	azucena_boundary_326;azucena_boundary_327	rufipogon_boundary_336
Group_158	meridionalis_boundary_351	azucena_boundary_358;azucena_boundary_359	
Group_159	meridionalis_boundary_363;meridionalis_boundary_364	azucena_boundary_372;azucena_boundary_375	
Group_160	meridionalis_boundary_379		rufipogon_boundary_381;rufipogon_boundary_382
Group_161	meridionalis_boundary_389;meridionalis_boundary_388	azucena_boundary_390	rufipogon_boundary_393;rufipogon_boundary_394
Group_162	meridionalis_boundary_448;meridionalis_boundary_449	azucena_boundary_451	rufipogon_boundary_451
Group_163	meridionalis_boundary_493	azucena_boundary_548	rufipogon_boundary_527
Group_164	meridionalis_boundary_498	azucena_boundary_551;azucena_boundary_552	rufipogon_boundary_530
Group_165	meridionalis_boundary_502;meridionalis_boundary_503	azucena_boundary_986;azucena_boundary_558	rufipogon_boundary_913
Group_166	meridionalis_boundary_605	azucena_boundary_661;azucena_boundary_662	rufipogon_boundary_614
Group_167	meridionalis_boundary_617	azucena_boundary_683	rufipogon_boundary_632
Group_168	meridionalis_boundary_619	azucena_boundary_685;azucena_boundary_684	rufipogon_boundary_636
Group_169	meridionalis_boundary_683;meridionalis_boundary_684	azucena_boundary_724	rufipogon_boundary_671
Group_170	meridionalis_boundary_707;meridionalis_boundary_708	azucena_boundary_732;azucena_boundary_733	
Group_171	meridionalis_boundary_78	azucena_boundary_84;azucena_boundary_83	rufipogon_boundary_86
Group_172	meridionalis_boundary_797;meridionalis_boundary_798	azucena_boundary_848	
Group_173	meridionalis_boundary_823;meridionalis_boundary_824	azucena_boundary_873	rufipogon_boundary_806
Group_174	meridionalis_boundary_862;meridionalis_boundary_863	azucena_boundary_923;azucena_boundary_924	rufipogon_boundary_861
Group_175	meridionalis_boundary_881	azucena_boundary_937	rufipogon_boundary_229
Group_176	meridionalis_boundary_978	azucena_boundary_1058;azucena_boundary_1059	
Group_177	meridionalis_boundary_988	azucena_boundary_1073;azucena_boundary_1074	
Group_178	meridionalis_boundary_1004;meridionalis_boundary_1005;meridionalis_boundary_1003		
Group_179	meridionalis_boundary_1119	azucena_boundary_1201	rufipogon_boundary_1123
Group_180	meridionalis_boundary_1178;meridionalis_boundary_1179	azucena_boundary_1219;azucena_boundary_1220	rufipogon_boundary_1139;rufipogon_boundary_1140
Group_181	meridionalis_boundary_1215	azucena_boundary_1276	rufipogon_boundary_1189
Group_182	meridionalis_boundary_1312		rufipogon_boundary_1290
Group_183		azucena_boundary_780	rufipogon_boundary_739
Group_184	meridionalis_boundary_194;meridionalis_boundary_195;meridionalis_boundary_196		rufipogon_boundary_213;rufipogon_boundary_214
Group_185	meridionalis_boundary_228	azucena_boundary_200	rufipogon_boundary_200
Group_186	meridionalis_boundary_24;meridionalis_boundary_25		rufipogon_boundary_24;rufipogon_boundary_23
Group_187	meridionalis_boundary_269	azucena_boundary_283	rufipogon_boundary_296;rufipogon_boundary_297
Group_188		azucena_boundary_680	rufipogon_boundary_630
Group_189	meridionalis_boundary_439;meridionalis_boundary_440		rufipogon_boundary_447
Group_190	meridionalis_boundary_488		rufipogon_boundary_526
Group_191	meridionalis_boundary_520;meridionalis_boundary_521		rufipogon_boundary_545;rufipogon_boundary_546
Group_192	meridionalis_boundary_675		rufipogon_boundary_656;rufipogon_boundary_657
Group_193	meridionalis_boundary_851		rufipogon_boundary_852;rufipogon_boundary_851
Group_194	meridionalis_boundary_1172;meridionalis_boundary_1173		
Group_195	meridionalis_boundary_1232;meridionalis_boundary_1233	azucena_boundary_1294	rufipogon_boundary_1199
Group_196	meridionalis_boundary_1426		
Group_197	meridionalis_boundary_1471	azucena_boundary_1562	
Group_198	meridionalis_boundary_172;meridionalis_boundary_173;meridionalis_boundary_174		
Group_199	meridionalis_boundary_664	azucena_boundary_705	rufipogon_boundary_653
Group_200	meridionalis_boundary_1295	azucena_boundary_1353	
Group_201		azucena_boundary_1038	rufipogon_boundary_478;rufipogon_boundary_955
Group_202		azucena_boundary_1050	rufipogon_boundary_973;rufipogon_boundary_974
Group_203		azucena_boundary_1077;azucena_boundary_1078	rufipogon_boundary_998
Group_204		azucena_boundary_1079	rufipogon_boundary_1000
Group_205		azucena_boundary_1148	rufipogon_boundary_1063;rufipogon_boundary_1064
Group_206		azucena_boundary_1324	rufipogon_boundary_1246;rufipogon_boundary_1247
Group_207		azucena_boundary_1370	rufipogon_boundary_1272;rufipogon_boundary_1273;rufipogon_boundary_1274
Group_208		azucena_boundary_159;azucena_boundary_777	rufipogon_boundary_160
Group_209		azucena_boundary_177	rufipogon_boundary_174;rufipogon_boundary_175
Group_210		azucena_boundary_180;azucena_boundary_179	rufipogon_boundary_177
Group_211		azucena_boundary_305	rufipogon_boundary_319;rufipogon_boundary_320
Group_212		azucena_boundary_349	rufipogon_boundary_357;rufipogon_boundary_356
Group_213		azucena_boundary_493;azucena_boundary_494	rufipogon_boundary_494
Group_214		azucena_boundary_519	rufipogon_boundary_506
Group_215		azucena_boundary_586;azucena_boundary_589	rufipogon_boundary_554
Group_216		azucena_boundary_635	rufipogon_boundary_597
Group_217		azucena_boundary_776	rufipogon_boundary_734
Group_218		azucena_boundary_792;azucena_boundary_793	rufipogon_boundary_746;rufipogon_boundary_747
Group_219		azucena_boundary_794;azucena_boundary_795	rufipogon_boundary_748;rufipogon_boundary_153
Group_220		azucena_boundary_821;azucena_boundary_822	rufipogon_boundary_762;rufipogon_boundary_763
Group_221		azucena_boundary_891;azucena_boundary_892	rufipogon_boundary_832
Group_222		azucena_boundary_1189;azucena_boundary_1190	
Group_223		azucena_boundary_1192;azucena_boundary_1193	rufipogon_boundary_1118
Group_224		azucena_boundary_427;azucena_boundary_428	
Group_225		azucena_boundary_742;azucena_boundary_743	rufipogon_boundary_708
Group_226		azucena_boundary_749	
Group_227		azucena_boundary_810;azucena_boundary_811	
Group_228		azucena_boundary_1570	rufipogon_boundary_1439;rufipogon_boundary_1440
Group_229		azucena_boundary_636	rufipogon_boundary_599;rufipogon_boundary_600
Group_230			rufipogon_boundary_1153;rufipogon_boundary_1154
Group_231			rufipogon_boundary_1297;rufipogon_boundary_1298
Group_232			rufipogon_boundary_1441;rufipogon_boundary_1442
Group_233			rufipogon_boundary_666;rufipogon_boundary_667
Group_234			rufipogon_boundary_718
Group_235			rufipogon_boundary_91
Group_236	meridionalis_boundary_1047;meridionalis_boundary_1048	azucena_boundary_1100;azucena_boundary_1101	
Group_237	meridionalis_boundary_1096	azucena_boundary_1158	
Group_238	meridionalis_boundary_1100	azucena_boundary_1166	rufipogon_boundary_1090
Group_239		azucena_boundary_144	rufipogon_boundary_140
Group_240	meridionalis_boundary_1196	azucena_boundary_1255	rufipogon_boundary_1172
Group_241	meridionalis_boundary_1203	azucena_boundary_1262;azucena_boundary_1261	
Group_242	meridionalis_boundary_1270;meridionalis_boundary_1272	azucena_boundary_1313;azucena_boundary_1311	
Group_243	meridionalis_boundary_1297;meridionalis_boundary_1298	azucena_boundary_1354	
Group_244	meridionalis_boundary_1311	azucena_boundary_1379;azucena_boundary_1380	
Group_245	meridionalis_boundary_1317;meridionalis_boundary_1316	azucena_boundary_1389	rufipogon_boundary_1294
Group_246	meridionalis_boundary_1318	azucena_boundary_1388	
Group_247	meridionalis_boundary_1372	azucena_boundary_1415	rufipogon_boundary_1320;rufipogon_boundary_1321
Group_248	meridionalis_boundary_1445	azucena_boundary_1399;azucena_boundary_1505	
Group_249	meridionalis_boundary_1465		
Group_250		azucena_boundary_764;azucena_boundary_763	
Group_251	meridionalis_boundary_168	azucena_boundary_189	rufipogon_boundary_186
Group_252	meridionalis_boundary_180	azucena_boundary_198	rufipogon_boundary_198
Group_253	meridionalis_boundary_204	azucena_boundary_209	rufipogon_boundary_221
Group_254	meridionalis_boundary_21	azucena_boundary_15	rufipogon_boundary_17;rufipogon_boundary_18
Group_255	meridionalis_boundary_217;meridionalis_boundary_218	azucena_boundary_220	rufipogon_boundary_234

Group_256	meridionalis_boundary_227	azucena_boundary_201	rufipogon_boundary_201
Group_257	meridionalis_boundary_23	azucena_boundary_16	rufipogon_boundary_20
Group_258	meridionalis_boundary_245	azucena_boundary_241	rufipogon_boundary_253
Group_259	meridionalis_boundary_246	azucena_boundary_242	rufipogon_boundary_254
Group_260	meridionalis_boundary_247	azucena_boundary_243	rufipogon_boundary_255
Group_261	meridionalis_boundary_268	azucena_boundary_282	rufipogon_boundary_295
Group_262	meridionalis_boundary_321	azucena_boundary_328	rufipogon_boundary_337
Group_263	meridionalis_boundary_356;meridionalis_boundary_357	azucena_boundary_366	rufipogon_boundary_370
Group_264	meridionalis_boundary_37;meridionalis_boundary_36	azucena_boundary_35;azucena_boundary_34	rufipogon_boundary_40
Group_265	meridionalis_boundary_380	azucena_boundary_383;azucena_boundary_384	rufipogon_boundary_383;rufipogon_boundary_384
Group_266	meridionalis_boundary_382	azucena_boundary_386;azucena_boundary_387	rufipogon_boundary_385
Group_267	meridionalis_boundary_390;meridionalis_boundary_391	azucena_boundary_394	rufipogon_boundary_398
Group_268	meridionalis_boundary_392;meridionalis_boundary_393	azucena_boundary_395	rufipogon_boundary_399
Group_269	meridionalis_boundary_404	azucena_boundary_415	rufipogon_boundary_420
Group_270	meridionalis_boundary_410;meridionalis_boundary_411	azucena_boundary_418;azucena_boundary_419	rufipogon_boundary_424
Group_271	meridionalis_boundary_414	azucena_boundary_422;azucena_boundary_423	
Group_272	meridionalis_boundary_415;meridionalis_boundary_416		
Group_273	meridionalis_boundary_417	azucena_boundary_424;azucena_boundary_425	
Group_274	meridionalis_boundary_419	azucena_boundary_426	
Group_275	meridionalis_boundary_424	azucena_boundary_430	
Group_276	meridionalis_boundary_444	azucena_boundary_448	rufipogon_boundary_449
Group_277	meridionalis_boundary_462;meridionalis_boundary_463	azucena_boundary_525;azucena_boundary_526	
Group_278	meridionalis_boundary_468	azucena_boundary_527	rufipogon_boundary_509
Group_279	meridionalis_boundary_475	azucena_boundary_531	rufipogon_boundary_514
Group_280	meridionalis_boundary_49;meridionalis_boundary_50	azucena_boundary_39	rufipogon_boundary_46
Group_281	meridionalis_boundary_505;meridionalis_boundary_929	azucena_boundary_994	
Group_282	meridionalis_boundary_518	azucena_boundary_573	rufipogon_boundary_543
Group_283	meridionalis_boundary_582	azucena_boundary_639	
Group_284	meridionalis_boundary_599;meridionalis_boundary_600	azucena_boundary_658	rufipogon_boundary_613
Group_285	meridionalis_boundary_620	azucena_boundary_686	
Group_286	meridionalis_boundary_641;meridionalis_boundary_642	azucena_boundary_701;azucena_boundary_702	
Group_287	meridionalis_boundary_674	azucena_boundary_710	
Group_288	meridionalis_boundary_698	azucena_boundary_730	rufipogon_boundary_683
Group_289		azucena_boundary_787;azucena_boundary_786	rufipogon_boundary_742
Group_290	meridionalis_boundary_750;meridionalis_boundary_749	azucena_boundary_825	
Group_291	meridionalis_boundary_752;meridionalis_boundary_753	azucena_boundary_827;azucena_boundary_828	
Group_292	meridionalis_boundary_774	azucena_boundary_831;azucena_boundary_832	rufipogon_boundary_773
Group_293	meridionalis_boundary_847	azucena_boundary_912	rufipogon_boundary_847
Group_294	meridionalis_boundary_848	azucena_boundary_913	rufipogon_boundary_1134
Group_295	meridionalis_boundary_907;meridionalis_boundary_908	azucena_boundary_959	rufipogon_boundary_885
Group_296	meridionalis_boundary_938;meridionalis_boundary_506	azucena_boundary_995	
Group_297	meridionalis_boundary_942;meridionalis_boundary_943	azucena_boundary_997	
Group_298	meridionalis_boundary_980	azucena_boundary_1064	rufipogon_boundary_988
Group_299	meridionalis_boundary_99	azucena_boundary_124	rufipogon_boundary_119
Group_300	meridionalis_boundary_993	azucena_boundary_1080	rufipogon_boundary_1001
Group_301		azucena_boundary_456;azucena_boundary_457	rufipogon_boundary_456;rufipogon_boundary_457
Group_302	meridionalis_boundary_1076;meridionalis_boundary_705		rufipogon_boundary_1039
Group_303	meridionalis_boundary_1192;meridionalis_boundary_1193		rufipogon_boundary_1171
Group_304	meridionalis_boundary_1214		rufipogon_boundary_1187
Group_305	meridionalis_boundary_125;meridionalis_boundary_126		
Group_306	meridionalis_boundary_1264		rufipogon_boundary_1226;rufipogon_boundary_1225
Group_307	meridionalis_boundary_1424;meridionalis_boundary_1422;meridionalis_boundary_1425		
Group_308	meridionalis_boundary_148	azucena_boundary_770	rufipogon_boundary_730
Group_309	meridionalis_boundary_149	azucena_boundary_769;azucena_boundary_166	rufipogon_boundary_729
Group_310	meridionalis_boundary_167		rufipogon_boundary_184;rufipogon_boundary_185
Group_311	meridionalis_boundary_215		rufipogon_boundary_231;rufipogon_boundary_230
Group_312	meridionalis_boundary_221		rufipogon_boundary_236
Group_313	meridionalis_boundary_270		rufipogon_boundary_298
Group_314	meridionalis_boundary_281;meridionalis_boundary_282		rufipogon_boundary_303
Group_315	meridionalis_boundary_298	azucena_boundary_664	rufipogon_boundary_316
Group_316	meridionalis_boundary_30		rufipogon_boundary_31;rufipogon_boundary_30
Group_317	meridionalis_boundary_38;meridionalis_boundary_39	azucena_boundary_36	rufipogon_boundary_41;rufipogon_boundary_42
Group_318	meridionalis_boundary_1309	azucena_boundary_1375	rufipogon_boundary_1282
Group_319	meridionalis_boundary_405	azucena_boundary_416	rufipogon_boundary_421
Group_320		azucena_boundary_434	rufipogon_boundary_436;rufipogon_boundary_435
Group_321	meridionalis_boundary_441		rufipogon_boundary_448
Group_322	meridionalis_boundary_59		rufipogon_boundary_66
Group_323	meridionalis_boundary_626	azucena_boundary_691	rufipogon_boundary_638
Group_324	meridionalis_boundary_696	azucena_boundary_729	rufipogon_boundary_680
Group_325	meridionalis_boundary_733	azucena_boundary_801	rufipogon_boundary_752
Group_326	meridionalis_boundary_754	azucena_boundary_829	rufipogon_boundary_768;rufipogon_boundary_769
Group_327	meridionalis_boundary_849		rufipogon_boundary_849
Group_328	meridionalis_boundary_899;meridionalis_boundary_900		rufipogon_boundary_876
Group_329	meridionalis_boundary_1042;meridionalis_boundary_1041		
Group_330		azucena_boundary_1196	
Group_331	meridionalis_boundary_1313;meridionalis_boundary_1314;meridionalis_boundary_1315		
Group_332	meridionalis_boundary_1416;meridionalis_boundary_1417	azucena_boundary_1478;azucena_boundary_1479	
Group_333	meridionalis_boundary_205;meridionalis_boundary_206	azucena_boundary_211	
Group_334	meridionalis_boundary_233		rufipogon_boundary_853
Group_335	meridionalis_boundary_33	azucena_boundary_29	rufipogon_boundary_34
Group_336	meridionalis_boundary_46		rufipogon_boundary_45
Group_337	meridionalis_boundary_623		
Group_338		azucena_boundary_1060	rufipogon_boundary_978;rufipogon_boundary_980
Group_339	meridionalis_boundary_954	azucena_boundary_1027;azucena_boundary_1026	
Group_340		azucena_boundary_1005;azucena_boundary_1004	rufipogon_boundary_931
Group_341		azucena_boundary_1036;azucena_boundary_1037	rufipogon_boundary_953;rufipogon_boundary_954
Group_342		azucena_boundary_1118;azucena_boundary_1117	rufipogon_boundary_1024
Group_343		azucena_boundary_1175;azucena_boundary_1174	rufipogon_boundary_1100
Group_344		azucena_boundary_123	rufipogon_boundary_118
Group_345		azucena_boundary_1233;azucena_boundary_1234;azucena_boundary_1235;azucena_boundary_1236	rufipogon_boundary_1152
Group_346		azucena_boundary_1295	rufipogon_boundary_1206;rufipogon_boundary_1207
Group_347		azucena_boundary_1325;azucena_boundary_1326	rufipogon_boundary_1248;rufipogon_boundary_1249
Group_348		azucena_boundary_1360;azucena_boundary_1361	rufipogon_boundary_1265;rufipogon_boundary_1266
Group_349		azucena_boundary_139	rufipogon_boundary_137
Group_350		azucena_boundary_1393;azucena_boundary_1500	rufipogon_boundary_1380
Group_351		azucena_boundary_1519	rufipogon_boundary_1394;rufipogon_boundary_1395
Group_352		azucena_boundary_1545;azucena_boundary_1546	rufipogon_boundary_1419
Group_353		azucena_boundary_1565;azucena_boundary_1566	rufipogon_boundary_1443;rufipogon_boundary_1444
Group_354		azucena_boundary_157;azucena_boundary_158	rufipogon_boundary_159;rufipogon_boundary_158
Group_355		azucena_boundary_1578	rufipogon_boundary_1453;rufipogon_boundary_1454
Group_356		azucena_boundary_169;azucena_boundary_170;azucena_boundary_768	rufipogon_boundary_165
Group_357		azucena_boundary_181	rufipogon_boundary_178;rufipogon_boundary_179
Group_358		azucena_boundary_407;azucena_boundary_408	rufipogon_boundary_410
Group_359		azucena_boundary_41	rufipogon_boundary_48
Group_360		azucena_boundary_508	rufipogon_boundary_505
Group_361		azucena_boundary_584	rufipogon_boundary_552
Group_362		azucena_boundary_624;azucena_boundary_623	rufipogon_boundary_584
Group_363		azucena_boundary_64;azucena_boundary_65	rufipogon_boundary_64;rufipogon_boundary_65
Group_364		azucena_boundary_69	rufipogon_boundary_69
Group_365		azucena_boundary_745;azucena_boundary_746	rufipogon_boundary_710
Group_366		azucena_boundary_782;azucena_boundary_783	rufipogon_boundary_740
Group_367		azucena_boundary_817	rufipogon_boundary_760
Group_368		azucena_boundary_864	rufipogon_boundary_797;rufipogon_boundary_796
Group_369		azucena_boundary_866	rufipogon_boundary_799
Group_370		azucena_boundary_98;azucena_boundary_97	rufipogon_boundary_96
Group_371		azucena_boundary_115;azucena_boundary_116	
Group_372		azucena_boundary_1160;azucena_boundary_1159	
Group_373		azucena_boundary_1299;azucena_boundary_1300	
Group_374		azucena_boundary_1394;azucena_boundary_1501	rufipogon_boundary_1301
Group_375		azucena_boundary_1466	
Group_376		azucena_boundary_1473;azucena_boundary_1474	
Group_377		azucena_boundary_1492;azucena_boundary_1493	
Group_378		azucena_boundary_1539	
Group_379		azucena_boundary_1590	
Group_380		azucena_boundary_218	rufipogon_boundary_232
Group_381		azucena_boundary_254	rufipogon_boundary_277;rufipogon_boundary_278
Group_382		azucena_boundary_260;azucena_boundary_261	
Group_383		azucena_boundary_513;azucena_boundary_514	
Group_384		azucena_boundary_644;azucena_boundary_643	

Group_385		azucena_boundary_931;azucena_boundary_932	
Group_386		azucena_boundary_1582	rufipogon_boundary_1457
Group_387		azucena_boundary_342	rufipogon_boundary_350;rufipogon_boundary_351
Group_388			
Group_389			rufipogon_boundary_1079;rufipogon_boundary_1080
Group_390			rufipogon_boundary_1446
Group_391			rufipogon_boundary_3;rufipogon_boundary_4
Group_392			rufipogon_boundary_488;rufipogon_boundary_489
Group_393			rufipogon_boundary_909;rufipogon_boundary_910
Group_394	meridionalis_boundary_100	azucena_boundary_125	rufipogon_boundary_120
Group_395	meridionalis_boundary_1016	azucena_boundary_1087	rufipogon_boundary_1007
Group_396	meridionalis_boundary_1073	azucena_boundary_1128	
Group_397	meridionalis_boundary_1083	azucena_boundary_1142	rufipogon_boundary_1055
Group_398	meridionalis_boundary_1093;meridionalis_boundary_1092	azucena_boundary_1153	
Group_399	meridionalis_boundary_1105	azucena_boundary_1170	rufipogon_boundary_1099
Group_400	meridionalis_boundary_112	azucena_boundary_135	rufipogon_boundary_134
Group_401	meridionalis_boundary_1204	azucena_boundary_1263;azucena_boundary_1264	
Group_402	meridionalis_boundary_1213	azucena_boundary_1275	rufipogon_boundary_1186
Group_403	meridionalis_boundary_1285	azucena_boundary_1340	
Group_404	meridionalis_boundary_1286	azucena_boundary_1341	rufipogon_boundary_1257
Group_405	meridionalis_boundary_1294	azucena_boundary_1352	rufipogon_boundary_1263
Group_406	meridionalis_boundary_1299	azucena_boundary_1362;azucena_boundary_1363	
Group_407	meridionalis_boundary_1301	azucena_boundary_1366	rufipogon_boundary_1268
Group_408	meridionalis_boundary_1302	azucena_boundary_1368	
Group_409	meridionalis_boundary_1359	azucena_boundary_1408;azucena_boundary_1409	rufipogon_boundary_1305
Group_410	meridionalis_boundary_1371	azucena_boundary_1414	rufipogon_boundary_1318;rufipogon_boundary_1319
Group_411	meridionalis_boundary_1376	azucena_boundary_1418	
Group_412	meridionalis_boundary_1379	azucena_boundary_1423	
Group_413	meridionalis_boundary_141	azucena_boundary_161	
Group_414	meridionalis_boundary_1455	azucena_boundary_1525	rufipogon_boundary_1409;rufipogon_boundary_1410
Group_415	meridionalis_boundary_1460	azucena_boundary_1532;azucena_boundary_1533	
Group_416		azucena_boundary_1230	rufipogon_boundary_1145
Group_417	meridionalis_boundary_207	azucena_boundary_213	rufipogon_boundary_224
Group_418	meridionalis_boundary_219	azucena_boundary_221	rufipogon_boundary_235
Group_419	meridionalis_boundary_226	azucena_boundary_202	rufipogon_boundary_202
Group_420	meridionalis_boundary_232	azucena_boundary_228	rufipogon_boundary_238
Group_421	meridionalis_boundary_237	azucena_boundary_232	rufipogon_boundary_244
Group_422	meridionalis_boundary_241	azucena_boundary_238	rufipogon_boundary_248
Group_423	meridionalis_boundary_262	azucena_boundary_277	
Group_424	meridionalis_boundary_263	azucena_boundary_279	rufipogon_boundary_292
Group_425	meridionalis_boundary_267	azucena_boundary_281	rufipogon_boundary_294
Group_426	meridionalis_boundary_273	azucena_boundary_285;azucena_boundary_286	
Group_427	meridionalis_boundary_28	azucena_boundary_26	rufipogon_boundary_25
Group_428	meridionalis_boundary_313	azucena_boundary_677;azucena_boundary_318	
Group_429		azucena_boundary_325	rufipogon_boundary_333;rufipogon_boundary_334
Group_430	meridionalis_boundary_322	azucena_boundary_329	rufipogon_boundary_338
Group_431	meridionalis_boundary_333	azucena_boundary_338	rufipogon_boundary_346
Group_432	meridionalis_boundary_34	azucena_boundary_31	rufipogon_boundary_37
Group_433	meridionalis_boundary_35	azucena_boundary_32	rufipogon_boundary_38
Group_434	meridionalis_boundary_358	azucena_boundary_367	rufipogon_boundary_369
Group_435	meridionalis_boundary_396	azucena_boundary_396	
Group_436	meridionalis_boundary_4;meridionalis_boundary_5	azucena_boundary_6	
Group_437	meridionalis_boundary_469	azucena_boundary_528	rufipogon_boundary_510
Group_438	meridionalis_boundary_482	azucena_boundary_542	rufipogon_boundary_524
Group_439	meridionalis_boundary_492	azucena_boundary_547	
Group_440	meridionalis_boundary_499;meridionalis_boundary_500	azucena_boundary_553	rufipogon_boundary_531
Group_441	meridionalis_boundary_513;meridionalis_boundary_512	azucena_boundary_571	
Group_442	meridionalis_boundary_519	azucena_boundary_574	rufipogon_boundary_544
Group_443	meridionalis_boundary_555	azucena_boundary_608;azucena_boundary_609	
Group_444	meridionalis_boundary_604;meridionalis_boundary_603	azucena_boundary_660	
Group_445	meridionalis_boundary_606	azucena_boundary_665;azucena_boundary_666	
Group_446	meridionalis_boundary_632	azucena_boundary_693	
Group_447	meridionalis_boundary_667	azucena_boundary_707	rufipogon_boundary_655
Group_448	meridionalis_boundary_790	azucena_boundary_846	
Group_449		azucena_boundary_853	rufipogon_boundary_787
Group_450	meridionalis_boundary_83	azucena_boundary_103	rufipogon_boundary_101
Group_451	meridionalis_boundary_830	azucena_boundary_881	rufipogon_boundary_817
Group_452	meridionalis_boundary_84	azucena_boundary_104	rufipogon_boundary_102
Group_453	meridionalis_boundary_865	azucena_boundary_927	
Group_454	meridionalis_boundary_894;meridionalis_boundary_895	azucena_boundary_947	rufipogon_boundary_872
Group_455	meridionalis_boundary_951	azucena_boundary_1021	rufipogon_boundary_943
Group_456	meridionalis_boundary_952	azucena_boundary_1022	rufipogon_boundary_944
Group_457	meridionalis_boundary_958	azucena_boundary_1032	rufipogon_boundary_950
Group_458	meridionalis_boundary_960	azucena_boundary_1039	rufipogon_boundary_958
Group_459	meridionalis_boundary_976		rufipogon_boundary_976
Group_460	meridionalis_boundary_982	azucena_boundary_1067	
Group_461	meridionalis_boundary_1102		rufipogon_boundary_1096;rufipogon_boundary_1097
Group_462	meridionalis_boundary_1120		rufipogon_boundary_1125
Group_463	meridionalis_boundary_1206	azucena_boundary_1266	rufipogon_boundary_1174
Group_464	meridionalis_boundary_1226		rufipogon_boundary_1195
Group_465	meridionalis_boundary_1229		rufipogon_boundary_1198
Group_466	meridionalis_boundary_1234		rufipogon_boundary_1209;rufipogon_boundary_1210
Group_467	meridionalis_boundary_1266		rufipogon_boundary_1227
Group_468	meridionalis_boundary_1296		rufipogon_boundary_1264
Group_469	meridionalis_boundary_1307;meridionalis_boundary_1308		rufipogon_boundary_1279;rufipogon_boundary_1280
Group_470	meridionalis_boundary_132	azucena_boundary_150	rufipogon_boundary_154
Group_471	meridionalis_boundary_133;meridionalis_boundary_135		rufipogon_boundary_156
Group_472	meridionalis_boundary_1374;meridionalis_boundary_1375		
Group_473	meridionalis_boundary_1401	azucena_boundary_1442	rufipogon_boundary_1341
Group_474		azucena_boundary_1498	rufipogon_boundary_1374;rufipogon_boundary_1373
Group_475	meridionalis_boundary_1488		rufipogon_boundary_1462
Group_476	meridionalis_boundary_177		rufipogon_boundary_188
Group_477	meridionalis_boundary_197;meridionalis_boundary_198		rufipogon_boundary_215;rufipogon_boundary_216
Group_478	meridionalis_boundary_203		rufipogon_boundary_220
Group_479	meridionalis_boundary_255		rufipogon_boundary_416
Group_480	meridionalis_boundary_256;meridionalis_boundary_257		rufipogon_boundary_415
Group_481	meridionalis_boundary_280		
Group_482		azucena_boundary_319;azucena_boundary_320	rufipogon_boundary_332
Group_483	meridionalis_boundary_326;meridionalis_boundary_325		
Group_484	meridionalis_boundary_423		rufipogon_boundary_428
Group_485	meridionalis_boundary_430;meridionalis_boundary_431		rufipogon_boundary_434
Group_486	meridionalis_boundary_479		rufipogon_boundary_521
Group_487	meridionalis_boundary_480		rufipogon_boundary_522
Group_488	meridionalis_boundary_494		rufipogon_boundary_528
Group_489	meridionalis_boundary_586;meridionalis_boundary_587		rufipogon_boundary_602
Group_490	meridionalis_boundary_666	azucena_boundary_706	rufipogon_boundary_654
Group_491	meridionalis_boundary_713		rufipogon_boundary_684;rufipogon_boundary_687;rufipogon_boundary_688
Group_492	meridionalis_boundary_736		rufipogon_boundary_754
Group_493	meridionalis_boundary_747		rufipogon_boundary_767
Group_494	meridionalis_boundary_783		rufipogon_boundary_779
Group_495	meridionalis_boundary_784		rufipogon_boundary_780
Group_496	meridionalis_boundary_796		rufipogon_boundary_786
Group_497	meridionalis_boundary_905		rufipogon_boundary_882
Group_498	meridionalis_boundary_965		rufipogon_boundary_963
Group_499	meridionalis_boundary_979		rufipogon_boundary_987
Group_500	meridionalis_boundary_1010		rufipogon_boundary_1005
Group_501	meridionalis_boundary_1014	azucena_boundary_1086	
Group_502	meridionalis_boundary_1020	azucena_boundary_1091	
Group_503	meridionalis_boundary_1071	azucena_boundary_1127	
Group_504	meridionalis_boundary_1171;meridionalis_boundary_1170		
Group_505	meridionalis_boundary_121	azucena_boundary_140	
Group_506	meridionalis_boundary_1268		
Group_507		azucena_boundary_751	rufipogon_boundary_714
Group_508		azucena_boundary_652	rufipogon_boundary_604
Group_509			rufipogon_boundary_312;rufipogon_boundary_611
Group_510	meridionalis_boundary_300;meridionalis_boundary_301		
Group_511	meridionalis_boundary_403		
Group_512	meridionalis_boundary_76	azucena_boundary_86	rufipogon_boundary_90
Group_513	meridionalis_boundary_789		

Group_514	meridionalis_boundary_882		
Group_515	meridionalis_boundary_903	azucena_boundary_955	
Group_516	meridionalis_boundary_91		
Group_517	meridionalis_boundary_963		rufipogon_boundary_962
Group_518	meridionalis_boundary_1116;meridionalis_boundary_1117		
Group_519	meridionalis_boundary_113	azucena_boundary_136	rufipogon_boundary_135
Group_520	meridionalis_boundary_1174		rufipogon_boundary_1131
Group_521	meridionalis_boundary_1475;meridionalis_boundary_1476	azucena_boundary_60	
Group_522	meridionalis_boundary_44		rufipogon_boundary_43
Group_523		azucena_boundary_909;azucena_boundary_910	rufipogon_boundary_838
Group_524		azucena_boundary_938	rufipogon_boundary_868
Group_525			rufipogon_boundary_881;rufipogon_boundary_880
Group_526		azucena_boundary_0;azucena_boundary_1	rufipogon_boundary_0
Group_527		azucena_boundary_1003	rufipogon_boundary_926
Group_528			rufipogon_boundary_932;rufipogon_boundary_924
Group_529		azucena_boundary_1017	rufipogon_boundary_936
Group_530		azucena_boundary_1033	rufipogon_boundary_951
Group_531		azucena_boundary_1034	rufipogon_boundary_952
Group_532		azucena_boundary_106	rufipogon_boundary_104
Group_533		azucena_boundary_1061	rufipogon_boundary_981
Group_534		azucena_boundary_1070	rufipogon_boundary_992
Group_535		azucena_boundary_1099	rufipogon_boundary_1019;rufipogon_boundary_1020
Group_536		azucena_boundary_1126	rufipogon_boundary_1031
Group_537		azucena_boundary_113	rufipogon_boundary_109
Group_538		azucena_boundary_1144	rufipogon_boundary_1057
Group_539		azucena_boundary_1188	rufipogon_boundary_1113
Group_540		azucena_boundary_1198	rufipogon_boundary_1121
Group_541		azucena_boundary_1199	rufipogon_boundary_1122
Group_542		azucena_boundary_1239	rufipogon_boundary_1155
Group_543		azucena_boundary_1248	rufipogon_boundary_1165
Group_544		azucena_boundary_1253	rufipogon_boundary_1170
Group_545		azucena_boundary_13	rufipogon_boundary_15
Group_546		azucena_boundary_1383	rufipogon_boundary_1288
Group_547		azucena_boundary_1384	rufipogon_boundary_1291;rufipogon_boundary_1292
Group_548		azucena_boundary_1396	rufipogon_boundary_1384
Group_549			rufipogon_boundary_1387
Group_550		azucena_boundary_1420	rufipogon_boundary_1326
Group_551		azucena_boundary_1424	rufipogon_boundary_1327
Group_552		azucena_boundary_1425	rufipogon_boundary_1328
Group_553		azucena_boundary_1426	rufipogon_boundary_1329
Group_554		azucena_boundary_1456	rufipogon_boundary_1348
Group_555		azucena_boundary_1457;azucena_boundary_1458	rufipogon_boundary_1349
Group_556		azucena_boundary_146	rufipogon_boundary_142;rufipogon_boundary_143
Group_557		azucena_boundary_1462	rufipogon_boundary_1354
Group_558		azucena_boundary_1463;azucena_boundary_1464	rufipogon_boundary_1355
Group_559		azucena_boundary_1468	rufipogon_boundary_1357
Group_560		azucena_boundary_1517	rufipogon_boundary_1392
Group_561		azucena_boundary_1518	rufipogon_boundary_1393
Group_562		azucena_boundary_1520	rufipogon_boundary_1396
Group_563		azucena_boundary_1521	rufipogon_boundary_1400
Group_564		azucena_boundary_1522	rufipogon_boundary_1401
Group_565		azucena_boundary_1572	rufipogon_boundary_1452
Group_566		azucena_boundary_176	rufipogon_boundary_173
Group_567		azucena_boundary_178	rufipogon_boundary_176
Group_568		azucena_boundary_196	rufipogon_boundary_197
Group_569		azucena_boundary_2	rufipogon_boundary_1
Group_570		azucena_boundary_207	rufipogon_boundary_207
Group_571		azucena_boundary_208	rufipogon_boundary_208
Group_572		azucena_boundary_212	rufipogon_boundary_223
Group_573		azucena_boundary_219	rufipogon_boundary_233
Group_574		azucena_boundary_234;azucena_boundary_235	rufipogon_boundary_245
Group_575		azucena_boundary_239	rufipogon_boundary_249;rufipogon_boundary_250
Group_576		azucena_boundary_311	rufipogon_boundary_328;rufipogon_boundary_329
Group_577		azucena_boundary_388	rufipogon_boundary_388
Group_578		azucena_boundary_393	rufipogon_boundary_397
Group_579		azucena_boundary_403	rufipogon_boundary_402
Group_580		azucena_boundary_405	rufipogon_boundary_406
Group_581		azucena_boundary_406	rufipogon_boundary_409
Group_582		azucena_boundary_409;azucena_boundary_410	rufipogon_boundary_411
Group_583		azucena_boundary_413	rufipogon_boundary_418
Group_584		azucena_boundary_42	rufipogon_boundary_49
Group_585		azucena_boundary_429	rufipogon_boundary_429
Group_586		azucena_boundary_43	rufipogon_boundary_50
Group_587		azucena_boundary_453	rufipogon_boundary_453
Group_588		azucena_boundary_458	rufipogon_boundary_458;rufipogon_boundary_459
Group_589		azucena_boundary_462	rufipogon_boundary_465;rufipogon_boundary_466
Group_590		azucena_boundary_465	rufipogon_boundary_469
Group_591		azucena_boundary_533	rufipogon_boundary_516;rufipogon_boundary_517
Group_592		azucena_boundary_538	rufipogon_boundary_520
Group_593		azucena_boundary_560	rufipogon_boundary_534
Group_594		azucena_boundary_576	rufipogon_boundary_547
Group_595		azucena_boundary_583	rufipogon_boundary_550;rufipogon_boundary_551
Group_596		azucena_boundary_634	rufipogon_boundary_594
Group_597		azucena_boundary_700	rufipogon_boundary_649
Group_598		azucena_boundary_703	rufipogon_boundary_650
Group_599		azucena_boundary_726	rufipogon_boundary_674
Group_600		azucena_boundary_736	rufipogon_boundary_694;rufipogon_boundary_695
Group_601		azucena_boundary_747	rufipogon_boundary_711
Group_602		azucena_boundary_837;azucena_boundary_835	rufipogon_boundary_776
Group_603		azucena_boundary_871	rufipogon_boundary_803
Group_604		azucena_boundary_893	rufipogon_boundary_831
Group_605		azucena_boundary_904;azucena_boundary_905	rufipogon_boundary_822
Group_606		azucena_boundary_917	rufipogon_boundary_856
Group_607		azucena_boundary_951	rufipogon_boundary_875
Group_608		azucena_boundary_958	rufipogon_boundary_884
Group_609		azucena_boundary_960	rufipogon_boundary_886
Group_610		azucena_boundary_971	rufipogon_boundary_898;rufipogon_boundary_899
Group_611		azucena_boundary_981	rufipogon_boundary_911
Group_612		azucena_boundary_982	rufipogon_boundary_912
Group_613		azucena_boundary_1030	rufipogon_boundary_948
Group_614		azucena_boundary_11	rufipogon_boundary_13
Group_615		azucena_boundary_1132	
Group_616		azucena_boundary_1268	
Group_617		azucena_boundary_1329	rufipogon_boundary_1251
Group_618		azucena_boundary_1445;azucena_boundary_1446	
Group_619		azucena_boundary_1540	rufipogon_boundary_1415
Group_620		azucena_boundary_206	rufipogon_boundary_206
Group_621		azucena_boundary_236	
Group_622		azucena_boundary_3	rufipogon_boundary_2
Group_623		azucena_boundary_341	rufipogon_boundary_349
Group_624		azucena_boundary_350	rufipogon_boundary_358
Group_625		azucena_boundary_398	rufipogon_boundary_400
Group_626		azucena_boundary_402	
Group_627		azucena_boundary_483	
Group_628		azucena_boundary_510	
Group_629		azucena_boundary_518	
Group_630		azucena_boundary_554	
Group_631		azucena_boundary_590	
Group_632		azucena_boundary_698	rufipogon_boundary_647
Group_633		azucena_boundary_728	rufipogon_boundary_676
Group_634		azucena_boundary_796	rufipogon_boundary_749
Group_635		azucena_boundary_807;azucena_boundary_806	
Group_636		azucena_boundary_867	rufipogon_boundary_800
Group_637		azucena_boundary_1083;azucena_boundary_1084	
Group_638		azucena_boundary_1114	
Group_639		azucena_boundary_1310	rufipogon_boundary_1232
Group_640		azucena_boundary_1392	rufipogon_boundary_1296
Group_641		azucena_boundary_1455	
Group_642			rufipogon_boundary_128;rufipogon_boundary_129

Group_643		azucena_boundary_274	rufipogon_boundary_290
Group_644		azucena_boundary_334	rufipogon_boundary_341
Group_645		azucena_boundary_438	
Group_646		azucena_boundary_600;azucena_boundary_601	
Group_647		azucena_boundary_606;azucena_boundary_607	
Group_648			rufipogon_boundary_1117
Group_649			rufipogon_boundary_1130
Group_650			rufipogon_boundary_1147
Group_651			rufipogon_boundary_12
Group_652			rufipogon_boundary_1244
Group_653			rufipogon_boundary_1308
Group_654			rufipogon_boundary_1311
Group_655			rufipogon_boundary_239;rufipogon_boundary_240
Group_656			rufipogon_boundary_257
Group_657			rufipogon_boundary_353
Group_658			rufipogon_boundary_548;rufipogon_boundary_549
Group_659			rufipogon_boundary_564
Group_660			rufipogon_boundary_673
Group_661			rufipogon_boundary_712;rufipogon_boundary_713
Group_662			rufipogon_boundary_733
Group_663			rufipogon_boundary_735;rufipogon_boundary_736
Group_664			rufipogon_boundary_804
Group_665			rufipogon_boundary_844
Group_666			rufipogon_boundary_892
Group_667			rufipogon_boundary_809;rufipogon_boundary_810
Group_668			rufipogon_boundary_841
Group_669			
Group_670			
Group_671			
Group_672	meridionalis_boundary_1079	azucena_boundary_1138	
Group_673	meridionalis_boundary_1095	azucena_boundary_1157	
Group_674	meridionalis_boundary_1195	azucena_boundary_1254	
Group_675	meridionalis_boundary_1220	azucena_boundary_1286	
Group_676		azucena_boundary_1305	
Group_677	meridionalis_boundary_1274	azucena_boundary_1345	
Group_678	meridionalis_boundary_1291	azucena_boundary_1351	
Group_679	meridionalis_boundary_138	azucena_boundary_153	
Group_680	meridionalis_boundary_1382	azucena_boundary_1428	
Group_681	meridionalis_boundary_1383	azucena_boundary_1429	
Group_682	meridionalis_boundary_1384	azucena_boundary_1430;azucena_boundary_1431	
Group_683	meridionalis_boundary_139	azucena_boundary_154;azucena_boundary_155	
Group_684	meridionalis_boundary_1446	azucena_boundary_1400	
Group_685	meridionalis_boundary_1457	azucena_boundary_1530	
Group_686	meridionalis_boundary_1484	azucena_boundary_1587	
Group_687	meridionalis_boundary_179	azucena_boundary_197	
Group_688	meridionalis_boundary_222	azucena_boundary_225	
Group_689		azucena_boundary_222	
Group_690	meridionalis_boundary_223	azucena_boundary_226	
Group_691	meridionalis_boundary_252	azucena_boundary_264	
Group_692	meridionalis_boundary_261	azucena_boundary_276	
Group_693	meridionalis_boundary_338	azucena_boundary_347	
Group_694	meridionalis_boundary_350	azucena_boundary_356	
Group_695	meridionalis_boundary_156	azucena_boundary_360	
Group_696	meridionalis_boundary_368	azucena_boundary_377	
Group_697		azucena_boundary_379	rufipogon_boundary_373
Group_698	meridionalis_boundary_418		
Group_699	meridionalis_boundary_426	azucena_boundary_431	
Group_700	meridionalis_boundary_442	azucena_boundary_444	
Group_701	meridionalis_boundary_446	azucena_boundary_450	
Group_702	meridionalis_boundary_452	azucena_boundary_497	rufipogon_boundary_497
Group_703	meridionalis_boundary_457	azucena_boundary_515	
Group_704	meridionalis_boundary_459	azucena_boundary_517	
Group_705	meridionalis_boundary_476	azucena_boundary_532	rufipogon_boundary_515
Group_706	meridionalis_boundary_491	azucena_boundary_546	
Group_707	meridionalis_boundary_504	azucena_boundary_559	
Group_708	meridionalis_boundary_514	azucena_boundary_572	rufipogon_boundary_540
Group_709	meridionalis_boundary_54	azucena_boundary_46	
Group_710	meridionalis_boundary_562;meridionalis_boundary_563	azucena_boundary_614	
Group_711	meridionalis_boundary_564	azucena_boundary_615;azucena_boundary_616	
Group_712	meridionalis_boundary_577	azucena_boundary_627	
Group_713	meridionalis_boundary_580	azucena_boundary_637	
Group_714		azucena_boundary_638	
Group_715	meridionalis_boundary_583	azucena_boundary_640	
Group_716	meridionalis_boundary_637	azucena_boundary_696	
Group_717	meridionalis_boundary_72	azucena_boundary_78	
Group_718	meridionalis_boundary_723;meridionalis_boundary_724	azucena_boundary_790	
Group_719	meridionalis_boundary_73;meridionalis_boundary_74	azucena_boundary_79	
Group_720	meridionalis_boundary_737	azucena_boundary_804	
Group_721	meridionalis_boundary_739	azucena_boundary_809	
Group_722	meridionalis_boundary_775	azucena_boundary_834	
Group_723	meridionalis_boundary_776	azucena_boundary_836	
Group_724	meridionalis_boundary_79;meridionalis_boundary_80	azucena_boundary_85	
Group_725	meridionalis_boundary_804	azucena_boundary_852	
Group_726	meridionalis_boundary_825	azucena_boundary_874	
Group_727	meridionalis_boundary_840;meridionalis_boundary_839		
Group_728		azucena_boundary_888	
Group_729	meridionalis_boundary_86	azucena_boundary_107	
Group_730	meridionalis_boundary_87	azucena_boundary_108	
Group_731	meridionalis_boundary_878	azucena_boundary_934	
Group_732	meridionalis_boundary_913	azucena_boundary_970	
Group_733	meridionalis_boundary_928	azucena_boundary_993	rufipogon_boundary_920
Group_734	meridionalis_boundary_955	azucena_boundary_1028	
Group_735	meridionalis_boundary_973	azucena_boundary_1054;azucena_boundary_1055	
Group_736	meridionalis_boundary_1067		rufipogon_boundary_1030
Group_737	meridionalis_boundary_111		rufipogon_boundary_132;rufipogon_boundary_133
Group_738	meridionalis_boundary_1122		rufipogon_boundary_1126
Group_739	meridionalis_boundary_1207		rufipogon_boundary_1177
Group_740	meridionalis_boundary_124		rufipogon_boundary_148
Group_741	meridionalis_boundary_1269		rufipogon_boundary_1230
Group_742	meridionalis_boundary_1373		rufipogon_boundary_1322
Group_743	meridionalis_boundary_1402		rufipogon_boundary_571
Group_744		azucena_boundary_1469	rufipogon_boundary_1359;rufipogon_boundary_1360
Group_745			rufipogon_boundary_1398
Group_746	meridionalis_boundary_235		rufipogon_boundary_242
Group_747	meridionalis_boundary_304		rufipogon_boundary_323
Group_748	meridionalis_boundary_306		rufipogon_boundary_326
Group_749	meridionalis_boundary_32	azucena_boundary_28	rufipogon_boundary_33
Group_750	meridionalis_boundary_355		rufipogon_boundary_368
Group_751	meridionalis_boundary_445		rufipogon_boundary_450
Group_752	meridionalis_boundary_474		rufipogon_boundary_513
Group_753	meridionalis_boundary_486		rufipogon_boundary_116
Group_754	meridionalis_boundary_596		rufipogon_boundary_607
Group_755	meridionalis_boundary_782		rufipogon_boundary_778
Group_756	meridionalis_boundary_901;meridionalis_boundary_902		
Group_757		azucena_boundary_954	rufipogon_boundary_879
Group_758	meridionalis_boundary_977		rufipogon_boundary_977
Group_759	meridionalis_boundary_1015		
Group_760	meridionalis_boundary_1034		rufipogon_boundary_1011
Group_761	meridionalis_boundary_1036		
Group_762	meridionalis_boundary_1039		
Group_763	meridionalis_boundary_1054		
Group_764	meridionalis_boundary_1084;meridionalis_boundary_1085		
Group_765	meridionalis_boundary_1360		rufipogon_boundary_1306
Group_766	meridionalis_boundary_1362;meridionalis_boundary_1363		
Group_767	meridionalis_boundary_1421		
Group_768	meridionalis_boundary_151		
Group_769	meridionalis_boundary_159		
Group_770		azucena_boundary_271	
Group_771	meridionalis_boundary_260	azucena_boundary_272	

Group_772	meridionalis_boundary_29		rufipogon_boundary_27
Group_773	meridionalis_boundary_291		
Group_774	meridionalis_boundary_324		
Group_775	meridionalis_boundary_348		
Group_776		azucena_boundary_1372	rufipogon_boundary_1277
Group_777	meridionalis_boundary_41		
Group_778	meridionalis_boundary_425		
Group_779	meridionalis_boundary_490		
Group_780	meridionalis_boundary_497		
Group_781	meridionalis_boundary_507;meridionalis_boundary_508		
Group_782	meridionalis_boundary_517		
Group_783	meridionalis_boundary_633		
Group_784	meridionalis_boundary_670;meridionalis_boundary_671		
Group_785	meridionalis_boundary_692		
Group_786	meridionalis_boundary_694		
Group_787	meridionalis_boundary_748		
Group_788	meridionalis_boundary_904		
Group_789	meridionalis_boundary_92		
Group_790	meridionalis_boundary_93;meridionalis_boundary_94		
Group_791	meridionalis_boundary_944		
Group_792	meridionalis_boundary_95		
Group_793	meridionalis_boundary_968		
Group_794	meridionalis_boundary_986		
Group_795		azucena_boundary_1075	
Group_796	meridionalis_boundary_994		
Group_797	meridionalis_boundary_995		
Group_798	meridionalis_boundary_1000;meridionalis_boundary_1001		
Group_799			rufipogon_boundary_1069
Group_800	meridionalis_boundary_1118	azucena_boundary_1200	
Group_801	meridionalis_boundary_1208;meridionalis_boundary_1209		
Group_802		azucena_boundary_1403;azucena_boundary_1506	
Group_803	meridionalis_boundary_1364		rufipogon_boundary_1312
Group_804	meridionalis_boundary_1477		
Group_805	meridionalis_boundary_1469	azucena_boundary_1558	
Group_806	meridionalis_boundary_189		
Group_807	meridionalis_boundary_211		rufipogon_boundary_228
Group_808			rufipogon_boundary_306
Group_809		azucena_boundary_653	rufipogon_boundary_605
Group_810	meridionalis_boundary_299		
Group_811	meridionalis_boundary_339	azucena_boundary_348	
Group_812	meridionalis_boundary_342;meridionalis_boundary_343		
Group_813	meridionalis_boundary_40	azucena_boundary_37	
Group_814	meridionalis_boundary_464;meridionalis_boundary_465		
Group_815	meridionalis_boundary_621	azucena_boundary_688	
Group_816	meridionalis_boundary_624;meridionalis_boundary_625		
Group_817	meridionalis_boundary_751	azucena_boundary_826	
Group_818	meridionalis_boundary_868		rufipogon_boundary_864
Group_819	meridionalis_boundary_915		rufipogon_boundary_897
Group_820	meridionalis_boundary_934		rufipogon_boundary_1190
Group_821	meridionalis_boundary_998;meridionalis_boundary_999		
Group_822		azucena_boundary_1012	rufipogon_boundary_933
Group_823		azucena_boundary_1031	rufipogon_boundary_949
Group_824		azucena_boundary_1040	rufipogon_boundary_959
Group_825		azucena_boundary_105	rufipogon_boundary_103
Group_826		azucena_boundary_1071	rufipogon_boundary_993
Group_827		azucena_boundary_1076	rufipogon_boundary_994
Group_828		azucena_boundary_1093	rufipogon_boundary_1010
Group_829		azucena_boundary_1098	rufipogon_boundary_1018
Group_830		azucena_boundary_1135	rufipogon_boundary_1043;rufipogon_boundary_1044
Group_831		azucena_boundary_1136;azucena_boundary_1137	rufipogon_boundary_1045
Group_832		azucena_boundary_1155	rufipogon_boundary_1077
Group_833		azucena_boundary_1156	rufipogon_boundary_1078
Group_834		azucena_boundary_1161	rufipogon_boundary_1084
Group_835		azucena_boundary_1176	rufipogon_boundary_1101
Group_836		azucena_boundary_1182	rufipogon_boundary_1106
Group_837		azucena_boundary_119	rufipogon_boundary_115
Group_838		azucena_boundary_1231	rufipogon_boundary_1151
Group_839		azucena_boundary_1244;azucena_boundary_1245	rufipogon_boundary_1162
Group_840		azucena_boundary_1246	rufipogon_boundary_1163
Group_841		azucena_boundary_1247	rufipogon_boundary_1164
Group_842		azucena_boundary_1252	rufipogon_boundary_1168
Group_843			rufipogon_boundary_1204;rufipogon_boundary_1205
Group_844		azucena_boundary_1318	rufipogon_boundary_1239
Group_845		azucena_boundary_137	rufipogon_boundary_136
Group_846		azucena_boundary_1382	rufipogon_boundary_1287
Group_847		azucena_boundary_1416	rufipogon_boundary_1323
Group_848		azucena_boundary_1417	rufipogon_boundary_1324
Group_849		azucena_boundary_1502	rufipogon_boundary_1382
Group_850		azucena_boundary_1551	rufipogon_boundary_1424
Group_851		azucena_boundary_1568	rufipogon_boundary_1447
Group_852		azucena_boundary_1583	rufipogon_boundary_1458
Group_853		azucena_boundary_1585	rufipogon_boundary_1460
Group_854		azucena_boundary_165	rufipogon_boundary_163
Group_855		azucena_boundary_183	rufipogon_boundary_180
Group_856		azucena_boundary_187	rufipogon_boundary_183
Group_857		azucena_boundary_195	rufipogon_boundary_196
Group_858		azucena_boundary_199	rufipogon_boundary_199
Group_859		azucena_boundary_203	rufipogon_boundary_203
Group_860		azucena_boundary_229	rufipogon_boundary_241
Group_861		azucena_boundary_231	rufipogon_boundary_243
Group_862		azucena_boundary_237	rufipogon_boundary_247
Group_863		azucena_boundary_247	rufipogon_boundary_268
Group_864		azucena_boundary_253	rufipogon_boundary_275
Group_865		azucena_boundary_269	rufipogon_boundary_288
Group_866		azucena_boundary_289	rufipogon_boundary_301
Group_867		azucena_boundary_290	rufipogon_boundary_305
Group_868		azucena_boundary_333	rufipogon_boundary_340
Group_869		azucena_boundary_353	rufipogon_boundary_359
Group_870		azucena_boundary_354	rufipogon_boundary_360
Group_871		azucena_boundary_414	rufipogon_boundary_419
Group_872		azucena_boundary_432	rufipogon_boundary_430
Group_873		azucena_boundary_433	rufipogon_boundary_432
Group_874		azucena_boundary_439	rufipogon_boundary_440
Group_875		azucena_boundary_452	rufipogon_boundary_452
Group_876		azucena_boundary_460	rufipogon_boundary_461
Group_877		azucena_boundary_461	rufipogon_boundary_463
Group_878		azucena_boundary_463	rufipogon_boundary_467
Group_879		azucena_boundary_464	rufipogon_boundary_468
Group_880		azucena_boundary_48	rufipogon_boundary_51
Group_881		azucena_boundary_503	rufipogon_boundary_504
Group_882		azucena_boundary_521	rufipogon_boundary_508
Group_883		azucena_boundary_56	rufipogon_boundary_56
Group_884		azucena_boundary_61	rufipogon_boundary_58
Group_885		azucena_boundary_633	rufipogon_boundary_593
Group_886		azucena_boundary_656	rufipogon_boundary_610
Group_887		azucena_boundary_668;azucena_boundary_669	rufipogon_boundary_616
Group_888		azucena_boundary_692	rufipogon_boundary_642
Group_889		azucena_boundary_711	rufipogon_boundary_658
Group_890		azucena_boundary_737	rufipogon_boundary_696
Group_891		azucena_boundary_738	rufipogon_boundary_697
Group_892		azucena_boundary_740	rufipogon_boundary_706
Group_893		azucena_boundary_752	rufipogon_boundary_715
Group_894		azucena_boundary_753	rufipogon_boundary_716
Group_895		azucena_boundary_754	rufipogon_boundary_717
Group_896		azucena_boundary_755	rufipogon_boundary_719
Group_897		azucena_boundary_757	rufipogon_boundary_720
Group_898		azucena_boundary_761	rufipogon_boundary_723
Group_899		azucena_boundary_762	rufipogon_boundary_724
Group_900		azucena_boundary_781	

Group_901		azucena_boundary_82	rufipogon_boundary_85
Group_902		azucena_boundary_838	rufipogon_boundary_777
Group_903		azucena_boundary_862	rufipogon_boundary_794
Group_904		azucena_boundary_882	rufipogon_boundary_818
Group_905		azucena_boundary_908	rufipogon_boundary_835;rufipogon_boundary_836
Group_906		azucena_boundary_945	rufipogon_boundary_870
Group_907		azucena_boundary_952	rufipogon_boundary_877
Group_908		azucena_boundary_961	rufipogon_boundary_887
Group_909			rufipogon_boundary_97
Group_910		azucena_boundary_99	rufipogon_boundary_99
Group_911		azucena_boundary_996	rufipogon_boundary_921
Group_912		azucena_boundary_1001	
Group_913		azucena_boundary_1020	rufipogon_boundary_942
Group_914		azucena_boundary_1041	
Group_915		azucena_boundary_1042	
Group_916		azucena_boundary_1107	
Group_917		azucena_boundary_1115	
Group_918		azucena_boundary_1120	
Group_919		azucena_boundary_1122	
Group_920		azucena_boundary_1145	
Group_921		azucena_boundary_1207	
Group_922		azucena_boundary_1211	
Group_923		azucena_boundary_1229	rufipogon_boundary_1144
Group_924		azucena_boundary_1304	rufipogon_boundary_1223
Group_925		azucena_boundary_1342;azucena_boundary_1343	
Group_926		azucena_boundary_1358	
Group_927		azucena_boundary_1369	
Group_928			rufipogon_boundary_1275
Group_929		azucena_boundary_1405	
Group_930		azucena_boundary_1432;azucena_boundary_1433	
Group_931		azucena_boundary_1438	
Group_932		azucena_boundary_1467	
Group_933		azucena_boundary_1509	
Group_934		azucena_boundary_1531	
Group_935		azucena_boundary_1538	
Group_936		azucena_boundary_1547	
Group_937		azucena_boundary_1555	
Group_938		azucena_boundary_156	
Group_939		azucena_boundary_1569	rufipogon_boundary_1448
Group_940		azucena_boundary_216	
Group_941		azucena_boundary_258	
Group_942		azucena_boundary_287	
Group_943		azucena_boundary_292	
Group_944		azucena_boundary_306;azucena_boundary_307	
Group_945			rufipogon_boundary_321
Group_946		azucena_boundary_351	
Group_947		azucena_boundary_357	
Group_948		azucena_boundary_417	
Group_949		azucena_boundary_420;azucena_boundary_421	
Group_950		azucena_boundary_475	
Group_951		azucena_boundary_50	
Group_952		azucena_boundary_504	
Group_953		azucena_boundary_512	
Group_954		azucena_boundary_543	
Group_955		azucena_boundary_544	
Group_956		azucena_boundary_545	
Group_957		azucena_boundary_561	
Group_958		azucena_boundary_562	
Group_959		azucena_boundary_577	
Group_960		azucena_boundary_58	
Group_961		azucena_boundary_59	
Group_962		azucena_boundary_598	
Group_963		azucena_boundary_621;azucena_boundary_622	
Group_964		azucena_boundary_632	
Group_965		azucena_boundary_650	
Group_966		azucena_boundary_682	rufipogon_boundary_631
Group_967		azucena_boundary_695	
Group_968		azucena_boundary_699	
Group_969		azucena_boundary_70	
Group_970		azucena_boundary_709	
Group_971		azucena_boundary_739	
Group_972		azucena_boundary_744	
Group_973		azucena_boundary_748	
Group_974		azucena_boundary_765	
Group_975		azucena_boundary_840	
Group_976		azucena_boundary_88	
Group_977		azucena_boundary_883	
Group_978		azucena_boundary_90	
Group_979		azucena_boundary_973	
Group_980		azucena_boundary_1095	rufipogon_boundary_1016
Group_981		azucena_boundary_1140	rufipogon_boundary_1053
Group_982		azucena_boundary_1213	
Group_983		azucena_boundary_1217	
Group_984		azucena_boundary_129	rufipogon_boundary_125
Group_985		azucena_boundary_1320;azucena_boundary_1338	
Group_986		azucena_boundary_1330	rufipogon_boundary_1252
Group_987		azucena_boundary_1331;azucena_boundary_1332	
Group_988		azucena_boundary_143	
Group_989		azucena_boundary_18;azucena_boundary_19	
Group_990		azucena_boundary_263	
Group_991		azucena_boundary_303	rufipogon_boundary_317
Group_992		azucena_boundary_476	
Group_993		azucena_boundary_488	
Group_994		azucena_boundary_491;azucena_boundary_492	
Group_995		azucena_boundary_498;azucena_boundary_499	
Group_996		azucena_boundary_5	
Group_997		azucena_boundary_522	
Group_998		azucena_boundary_523	
Group_999		azucena_boundary_591	
Group_1000		azucena_boundary_759	rufipogon_boundary_721
Group_1001		azucena_boundary_81	rufipogon_boundary_74
Group_1002		azucena_boundary_847	
Group_1003		azucena_boundary_860	
Group_1004		azucena_boundary_885	rufipogon_boundary_820
Group_1005		azucena_boundary_889	
Group_1006		azucena_boundary_91;azucena_boundary_92	
Group_1007		azucena_boundary_950	rufipogon_boundary_874
Group_1008			rufipogon_boundary_1028
Group_1009			rufipogon_boundary_1075
Group_1010			rufipogon_boundary_1081
Group_1011			rufipogon_boundary_1095
Group_1012			rufipogon_boundary_1114
Group_1013			rufipogon_boundary_1229
Group_1014			rufipogon_boundary_1236
Group_1015			rufipogon_boundary_1289
Group_1016			rufipogon_boundary_1338
Group_1017			rufipogon_boundary_1399
Group_1018			rufipogon_boundary_14
Group_1019			rufipogon_boundary_1407
Group_1020			rufipogon_boundary_1426
Group_1021			rufipogon_boundary_1428
Group_1022			rufipogon_boundary_1445
Group_1023			rufipogon_boundary_263
Group_1024			rufipogon_boundary_272
Group_1025			rufipogon_boundary_276
Group_1026			rufipogon_boundary_376
Group_1027			rufipogon_boundary_389
Group_1028			rufipogon_boundary_427
Group_1029			rufipogon_boundary_482

Group_1030			rufipogon_boundary_490
Group_1031			rufipogon_boundary_501;rufipogon_boundary_502
Group_1032			rufipogon_boundary_588
Group_1033			rufipogon_boundary_595
Group_1034			rufipogon_boundary_652
Group_1035			rufipogon_boundary_67
Group_1036			rufipogon_boundary_70
Group_1037			rufipogon_boundary_761
Group_1038			rufipogon_boundary_82
Group_1039			rufipogon_boundary_843
Group_1040			rufipogon_boundary_88
Group_1041			rufipogon_boundary_900
Group_1042			rufipogon_boundary_968
Group_1043			rufipogon_boundary_995
Group_1044			rufipogon_boundary_1201
Group_1045			rufipogon_boundary_1228
Group_1046			rufipogon_boundary_1238
Group_1047			rufipogon_boundary_1344;rufipogon_boundary_1345
Group_1048			rufipogon_boundary_145
Group_1049			rufipogon_boundary_190
Group_1050			rufipogon_boundary_251
Group_1051			rufipogon_boundary_264;rufipogon_boundary_265
Group_1052			rufipogon_boundary_291
Group_1053			rufipogon_boundary_362
Group_1054			rufipogon_boundary_407;rufipogon_boundary_408
Group_1055			rufipogon_boundary_485;rufipogon_boundary_486
Group_1056			rufipogon_boundary_560;rufipogon_boundary_561
Group_1057			rufipogon_boundary_623;rufipogon_boundary_624
Group_1058			rufipogon_boundary_640
Group_1059			rufipogon_boundary_732
Group_1060			rufipogon_boundary_770
Group_1061			rufipogon_boundary_80
Group_1062			rufipogon_boundary_827
Group_1063			rufipogon_boundary_945;rufipogon_boundary_946
Group_1064			rufipogon_boundary_984
Group_1065			
Group_1066			
Group_1067			
Group_1068			
Group_1069			
Group_1070			
Group_1071			
Group_1072	meridionalis_boundary_1019	azucena_boundary_1089	
Group_1073	meridionalis_boundary_1049	azucena_boundary_1103	
Group_1074	meridionalis_boundary_1062	azucena_boundary_1113	
Group_1075	meridionalis_boundary_1181	azucena_boundary_1222	
Group_1076	meridionalis_boundary_1198	azucena_boundary_1256	
Group_1077	meridionalis_boundary_1202	azucena_boundary_1260	
Group_1078	meridionalis_boundary_1267		
Group_1079		azucena_boundary_1307	
Group_1080	meridionalis_boundary_1271	azucena_boundary_1312	
Group_1081	meridionalis_boundary_1289	azucena_boundary_1348	
Group_1082	meridionalis_boundary_1310	azucena_boundary_1378	
Group_1083	meridionalis_boundary_1355	azucena_boundary_1402	
Group_1084	meridionalis_boundary_1358	azucena_boundary_1406	
Group_1085	meridionalis_boundary_1369	azucena_boundary_1412	
Group_1086	meridionalis_boundary_1456	azucena_boundary_1528	
Group_1087	meridionalis_boundary_1462	azucena_boundary_1534	
Group_1088	meridionalis_boundary_1485	azucena_boundary_1593	
Group_1089	meridionalis_boundary_150	azucena_boundary_167	
Group_1090	meridionalis_boundary_176	azucena_boundary_193	
Group_1091	meridionalis_boundary_234	azucena_boundary_230	
Group_1092	meridionalis_boundary_289	azucena_boundary_298	
Group_1093	meridionalis_boundary_31	azucena_boundary_27	
Group_1094	meridionalis_boundary_332	azucena_boundary_337	
Group_1095	meridionalis_boundary_337	azucena_boundary_346	
Group_1096	meridionalis_boundary_341	azucena_boundary_352	
Group_1097	meridionalis_boundary_365	azucena_boundary_374	
Group_1098	meridionalis_boundary_366	azucena_boundary_376	
Group_1099	meridionalis_boundary_450	azucena_boundary_490	
Group_1100	meridionalis_boundary_458	azucena_boundary_516	
Group_1101	meridionalis_boundary_48	azucena_boundary_38	
Group_1102	meridionalis_boundary_53	azucena_boundary_45	
Group_1103	meridionalis_boundary_554	azucena_boundary_597	
Group_1104	meridionalis_boundary_56	azucena_boundary_55	
Group_1105	meridionalis_boundary_594	azucena_boundary_646	
Group_1106	meridionalis_boundary_60	azucena_boundary_68	
Group_1107	meridionalis_boundary_609	azucena_boundary_674	
Group_1108	meridionalis_boundary_612	azucena_boundary_675	
Group_1109	meridionalis_boundary_613	azucena_boundary_676	
Group_1110	meridionalis_boundary_622	azucena_boundary_690	
Group_1111	meridionalis_boundary_66	azucena_boundary_75	
Group_1112	meridionalis_boundary_679	azucena_boundary_722	
Group_1113	meridionalis_boundary_711	azucena_boundary_898	
Group_1114	meridionalis_boundary_729	azucena_boundary_797	
Group_1115	meridionalis_boundary_75	azucena_boundary_80	
Group_1116	meridionalis_boundary_778	azucena_boundary_844	
Group_1117	meridionalis_boundary_799	azucena_boundary_850	
Group_1118	meridionalis_boundary_803	azucena_boundary_851	
Group_1119	meridionalis_boundary_877	azucena_boundary_933	
Group_1120	meridionalis_boundary_933	azucena_boundary_1280	
Group_1121	meridionalis_boundary_987	azucena_boundary_1072	
Group_1122	meridionalis_boundary_110		rufipogon_boundary_131
Group_1123	meridionalis_boundary_1104		rufipogon_boundary_1098
Group_1124	meridionalis_boundary_1200		rufipogon_boundary_1173
Group_1125	meridionalis_boundary_1278		rufipogon_boundary_1242
Group_1126	meridionalis_boundary_1453		rufipogon_boundary_1404
Group_1127	meridionalis_boundary_193		rufipogon_boundary_210
Group_1128	meridionalis_boundary_2		rufipogon_boundary_5
Group_1129	meridionalis_boundary_307		rufipogon_boundary_327
Group_1130	meridionalis_boundary_323		rufipogon_boundary_339
Group_1131	meridionalis_boundary_421		rufipogon_boundary_425
Group_1132	meridionalis_boundary_429		rufipogon_boundary_433
Group_1133	meridionalis_boundary_45		rufipogon_boundary_44
Group_1134		azucena_boundary_541	rufipogon_boundary_523
Group_1135	meridionalis_boundary_578		rufipogon_boundary_598
Group_1136	meridionalis_boundary_58		rufipogon_boundary_63
Group_1137	meridionalis_boundary_584		rufipogon_boundary_601
Group_1138	meridionalis_boundary_597		rufipogon_boundary_608
Group_1139	meridionalis_boundary_618		rufipogon_boundary_633
Group_1140	meridionalis_boundary_682		rufipogon_boundary_670
Group_1141	meridionalis_boundary_704		
Group_1142	meridionalis_boundary_745		rufipogon_boundary_759
Group_1143	meridionalis_boundary_77		rufipogon_boundary_77
Group_1144	meridionalis_boundary_983		rufipogon_boundary_990
Group_1145	meridionalis_boundary_984		rufipogon_boundary_991
Group_1146	meridionalis_boundary_989		rufipogon_boundary_999
Group_1147	meridionalis_boundary_1006		
Group_1148	meridionalis_boundary_101		
Group_1149	meridionalis_boundary_102		
Group_1150	meridionalis_boundary_1040		
Group_1151	meridionalis_boundary_1121		
Group_1152	meridionalis_boundary_1230		
Group_1153	meridionalis_boundary_1356		
Group_1154	meridionalis_boundary_1361		
Group_1155	meridionalis_boundary_142		
Group_1156	meridionalis_boundary_155		
Group_1157	meridionalis_boundary_160		
Group_1158	meridionalis_boundary_187		

Group_1159	meridionalis_boundary_209		
Group_1160	meridionalis_boundary_210		
Group_1161	meridionalis_boundary_224		
Group_1162	meridionalis_boundary_254		
Group_1163	meridionalis_boundary_277		
Group_1164	meridionalis_boundary_279		
Group_1165	meridionalis_boundary_283		
Group_1166	meridionalis_boundary_3		
Group_1167	meridionalis_boundary_369		
Group_1168	meridionalis_boundary_407		
Group_1169	meridionalis_boundary_408		
Group_1170	meridionalis_boundary_42		
Group_1171			
Group_1172	meridionalis_boundary_43		
Group_1173	meridionalis_boundary_509		
Group_1174	meridionalis_boundary_516		
Group_1175	meridionalis_boundary_544		
Group_1176	meridionalis_boundary_545		
Group_1177	meridionalis_boundary_546		
Group_1178	meridionalis_boundary_590		
Group_1179	meridionalis_boundary_598		
Group_1180	meridionalis_boundary_628		
Group_1181	meridionalis_boundary_669		
Group_1182	meridionalis_boundary_672		
Group_1183	meridionalis_boundary_680		
Group_1184	meridionalis_boundary_701		
Group_1185	meridionalis_boundary_703		
Group_1186	meridionalis_boundary_71		
Group_1187	meridionalis_boundary_826		
Group_1188	meridionalis_boundary_827		
Group_1189	meridionalis_boundary_828		
Group_1190	meridionalis_boundary_843		
Group_1191	meridionalis_boundary_866		
Group_1192	meridionalis_boundary_891		
Group_1193	meridionalis_boundary_937		
Group_1194			
Group_1195	meridionalis_boundary_0		
Group_1196	meridionalis_boundary_1002		
Group_1197			
Group_1198	meridionalis_boundary_11		
Group_1199	meridionalis_boundary_1123		
Group_1200	meridionalis_boundary_114		
Group_1201	meridionalis_boundary_1166		
Group_1202	meridionalis_boundary_1182		
Group_1203	meridionalis_boundary_1201		
Group_1204	meridionalis_boundary_1287		
Group_1205	meridionalis_boundary_1389		
Group_1206	meridionalis_boundary_1410		
Group_1207	meridionalis_boundary_1466		
Group_1208	meridionalis_boundary_1473		
Group_1209	meridionalis_boundary_1474		
Group_1210	meridionalis_boundary_185		
Group_1211	meridionalis_boundary_22		
Group_1212	meridionalis_boundary_294		
Group_1213	meridionalis_boundary_295		
Group_1214	meridionalis_boundary_361		
Group_1215	meridionalis_boundary_362		
Group_1216	meridionalis_boundary_385		
Group_1217	meridionalis_boundary_409		
Group_1218	meridionalis_boundary_454		
Group_1219	meridionalis_boundary_501		
Group_1220	meridionalis_boundary_51		
Group_1221	meridionalis_boundary_627		
Group_1222	meridionalis_boundary_636		
Group_1223	meridionalis_boundary_640		
Group_1224	meridionalis_boundary_65		
Group_1225			rufipogon_boundary_685
Group_1226	meridionalis_boundary_744		
Group_1227	meridionalis_boundary_859		
Group_1228	meridionalis_boundary_871		
Group_1229	meridionalis_boundary_88		
Group_1230	meridionalis_boundary_897		
Group_1231	meridionalis_boundary_914		
Group_1232	meridionalis_boundary_931		
Group_1233	meridionalis_boundary_948		
Group_1234	meridionalis_boundary_962		
Group_1235	meridionalis_boundary_98		
Group_1236		azucena_boundary_1002	rufipogon_boundary_925
Group_1237		azucena_boundary_1011	
Group_1238		azucena_boundary_1018	rufipogon_boundary_940
Group_1239		azucena_boundary_1019	rufipogon_boundary_941
Group_1240		azucena_boundary_1046	rufipogon_boundary_961
Group_1241		azucena_boundary_1065	rufipogon_boundary_989
Group_1242		azucena_boundary_1085	rufipogon_boundary_1006
Group_1243		azucena_boundary_109	
Group_1244		azucena_boundary_1096	rufipogon_boundary_1017
Group_1245		azucena_boundary_1109	rufipogon_boundary_1021
Group_1246		azucena_boundary_1139	rufipogon_boundary_1052
Group_1247		azucena_boundary_1143	rufipogon_boundary_1056
Group_1248		azucena_boundary_1183	rufipogon_boundary_1107
Group_1249		azucena_boundary_1184	rufipogon_boundary_1108
Group_1250		azucena_boundary_1185	rufipogon_boundary_1109
Group_1251		azucena_boundary_120	
Group_1252		azucena_boundary_1225	rufipogon_boundary_1141
Group_1253		azucena_boundary_1226	rufipogon_boundary_1143
Group_1254		azucena_boundary_1243	rufipogon_boundary_1159
Group_1255		azucena_boundary_1314	rufipogon_boundary_1235
Group_1256		azucena_boundary_1385	rufipogon_boundary_1293
Group_1257		azucena_boundary_1410	rufipogon_boundary_1313
Group_1258		azucena_boundary_1411	rufipogon_boundary_1314
Group_1259		azucena_boundary_1413	rufipogon_boundary_1315
Group_1260		azucena_boundary_1427	rufipogon_boundary_1330
Group_1261		azucena_boundary_1459	rufipogon_boundary_1351
Group_1262		azucena_boundary_1480	rufipogon_boundary_1362
Group_1263		azucena_boundary_1483	rufipogon_boundary_1367
Group_1264		azucena_boundary_1524	rufipogon_boundary_1402
Group_1265		azucena_boundary_1537	rufipogon_boundary_1413
Group_1266		azucena_boundary_1592	rufipogon_boundary_1465
Group_1267		azucena_boundary_1594	rufipogon_boundary_1466
Group_1268		azucena_boundary_227	rufipogon_boundary_237
Group_1269		azucena_boundary_245	rufipogon_boundary_256
Group_1270		azucena_boundary_256	rufipogon_boundary_282
Group_1271		azucena_boundary_280	rufipogon_boundary_293
Group_1272		azucena_boundary_30	rufipogon_boundary_36
Group_1273		azucena_boundary_301	rufipogon_boundary_315
Group_1274		azucena_boundary_312	rufipogon_boundary_330
Group_1275		azucena_boundary_33	rufipogon_boundary_39
Group_1276		azucena_boundary_336	rufipogon_boundary_345
Group_1277			rufipogon_boundary_462
Group_1278		azucena_boundary_466	
Group_1279			rufipogon_boundary_470
Group_1280		azucena_boundary_595	rufipogon_boundary_557
Group_1281		azucena_boundary_602	rufipogon_boundary_567
Group_1282		azucena_boundary_62	rufipogon_boundary_61
Group_1283		azucena_boundary_63	rufipogon_boundary_62
Group_1284		azucena_boundary_678	rufipogon_boundary_628
Group_1285		azucena_boundary_723	rufipogon_boundary_669
Group_1286		azucena_boundary_735	rufipogon_boundary_692
Group_1287		azucena_boundary_767	

Group_1288			rufipogon_boundary_728
Group_1289		azucena_boundary_779	rufipogon_boundary_737
Group_1290		azucena_boundary_865	rufipogon_boundary_798
Group_1291		azucena_boundary_894	rufipogon_boundary_826
Group_1292		azucena_boundary_907	
Group_1293		azucena_boundary_953	rufipogon_boundary_878
Group_1294		azucena_boundary_100	
Group_1295		azucena_boundary_1092	
Group_1296		azucena_boundary_1102	
Group_1297		azucena_boundary_1119	
Group_1298		azucena_boundary_1124	
Group_1299		azucena_boundary_1154	
Group_1300		azucena_boundary_1171	
Group_1301		azucena_boundary_1172	
Group_1302		azucena_boundary_1209	
Group_1303		azucena_boundary_1237	
Group_1304		azucena_boundary_1238	
Group_1305		azucena_boundary_1301	
Group_1306		azucena_boundary_1315	
Group_1307		azucena_boundary_1322	
Group_1308		azucena_boundary_134	
Group_1309		azucena_boundary_1344	
Group_1310		azucena_boundary_1356	
Group_1311		azucena_boundary_1401	
Group_1312		azucena_boundary_1441	
Group_1313		azucena_boundary_1461	
Group_1314		azucena_boundary_1465	
Group_1315		azucena_boundary_1475	
Group_1316			
Group_1317		azucena_boundary_1541	
Group_1318		azucena_boundary_1576	
Group_1319		azucena_boundary_175	
Group_1320		azucena_boundary_273	
Group_1321		azucena_boundary_278	
Group_1322		azucena_boundary_288	
Group_1323		azucena_boundary_308	
Group_1324		azucena_boundary_322	
Group_1325		azucena_boundary_445	
Group_1326		azucena_boundary_479	
Group_1327		azucena_boundary_482	
Group_1328		azucena_boundary_524	
Group_1329		azucena_boundary_557	
Group_1330		azucena_boundary_565	
Group_1331		azucena_boundary_585	
Group_1332		azucena_boundary_592	
Group_1333		azucena_boundary_651	
Group_1334		azucena_boundary_667	
Group_1335		azucena_boundary_671	
Group_1336		azucena_boundary_687	
Group_1337		azucena_boundary_731	
Group_1338		azucena_boundary_756	
Group_1339		azucena_boundary_775	
Group_1340		azucena_boundary_808	
Group_1341		azucena_boundary_830	
Group_1342		azucena_boundary_859	
Group_1343		azucena_boundary_868	
Group_1344		azucena_boundary_89	
Group_1345		azucena_boundary_946	
Group_1346		azucena_boundary_962	
Group_1347		azucena_boundary_963	
Group_1348		azucena_boundary_983	
Group_1349		azucena_boundary_984	
Group_1350		azucena_boundary_1023	
Group_1351		azucena_boundary_1025	
Group_1352		azucena_boundary_1051	
Group_1353		azucena_boundary_1088	
Group_1354		azucena_boundary_1111	
Group_1355		azucena_boundary_1112	
Group_1356		azucena_boundary_1121	
Group_1357		azucena_boundary_1131	
Group_1358		azucena_boundary_1133	
Group_1359		azucena_boundary_1146	
Group_1360		azucena_boundary_1162	
Group_1361		azucena_boundary_1205	
Group_1362		azucena_boundary_1218	
Group_1363		azucena_boundary_1221	
Group_1364		azucena_boundary_1227	
Group_1365		azucena_boundary_1228	
Group_1366		azucena_boundary_1241	
Group_1367		azucena_boundary_1251	
Group_1368		azucena_boundary_1267	
Group_1369		azucena_boundary_1290	
Group_1370			
Group_1371		azucena_boundary_1333	
Group_1372		azucena_boundary_1339	
Group_1373		azucena_boundary_1422	
Group_1374		azucena_boundary_1434	
Group_1375		azucena_boundary_1440	
Group_1376		azucena_boundary_1443	
Group_1377		azucena_boundary_1444	
Group_1378		azucena_boundary_1447	
Group_1379		azucena_boundary_1549	
Group_1380		azucena_boundary_1552	
Group_1381		azucena_boundary_1553	
Group_1382		azucena_boundary_1554	
Group_1383		azucena_boundary_1557	
Group_1384		azucena_boundary_1559	
Group_1385		azucena_boundary_1563	
Group_1386		azucena_boundary_1595	
Group_1387		azucena_boundary_1596	
Group_1388		azucena_boundary_171	
Group_1389		azucena_boundary_248	
Group_1390		azucena_boundary_251	
Group_1391		azucena_boundary_291	
Group_1392		azucena_boundary_332	
Group_1393		azucena_boundary_343	
Group_1394		azucena_boundary_389	
Group_1395		azucena_boundary_446	
Group_1396		azucena_boundary_447	
Group_1397		azucena_boundary_449	
Group_1398		azucena_boundary_455	
Group_1399		azucena_boundary_470	
Group_1400		azucena_boundary_484	
Group_1401		azucena_boundary_485	
Group_1402		azucena_boundary_505	
Group_1403		azucena_boundary_506	
Group_1404		azucena_boundary_507	
Group_1405		azucena_boundary_570	
Group_1406		azucena_boundary_575	
Group_1407		azucena_boundary_578	
Group_1408		azucena_boundary_579	
Group_1409		azucena_boundary_580	
Group_1410		azucena_boundary_581	
Group_1411		azucena_boundary_610	
Group_1412		azucena_boundary_630	
Group_1413		azucena_boundary_648	
Group_1414		azucena_boundary_708	
Group_1415		azucena_boundary_725	
Group_1416			

Group_1417		azucena_boundary_805	
Group_1418		azucena_boundary_813	
Group_1419		azucena_boundary_854	
Group_1420		azucena_boundary_857	
Group_1421		azucena_boundary_902	
Group_1422		azucena_boundary_906	
Group_1423		azucena_boundary_914	
Group_1424		azucena_boundary_93	
Group_1425		azucena_boundary_930	
Group_1426		azucena_boundary_935	
Group_1427		azucena_boundary_965	
Group_1428		azucena_boundary_980	
Group_1429			rufipogon_boundary_1002
Group_1430			rufipogon_boundary_1008
Group_1431			rufipogon_boundary_1029
Group_1432			rufipogon_boundary_1034
Group_1433			rufipogon_boundary_1035
Group_1434			rufipogon_boundary_1036
Group_1435			rufipogon_boundary_1049
Group_1436			rufipogon_boundary_1061
Group_1437			rufipogon_boundary_1076
Group_1438			rufipogon_boundary_1092
Group_1439			rufipogon_boundary_113
Group_1440			rufipogon_boundary_1150
Group_1441			rufipogon_boundary_1158
Group_1442			rufipogon_boundary_1161
Group_1443			rufipogon_boundary_1178
Group_1444			rufipogon_boundary_1196
Group_1445			rufipogon_boundary_1200
Group_1446			rufipogon_boundary_1202
Group_1447			rufipogon_boundary_1216
Group_1448			rufipogon_boundary_1217
Group_1449			rufipogon_boundary_1237
Group_1450			rufipogon_boundary_1270
Group_1451			rufipogon_boundary_1310
Group_1452			rufipogon_boundary_1356
Group_1453			rufipogon_boundary_1365
Group_1454			rufipogon_boundary_1381
Group_1455			rufipogon_boundary_1397
Group_1456			rufipogon_boundary_1406
Group_1457			rufipogon_boundary_1418
Group_1458			rufipogon_boundary_1449
Group_1459			rufipogon_boundary_172
Group_1460			rufipogon_boundary_189
Group_1461			rufipogon_boundary_191
Group_1462			rufipogon_boundary_192
Group_1463			rufipogon_boundary_209
Group_1464			rufipogon_boundary_262
Group_1465			rufipogon_boundary_274
Group_1466			rufipogon_boundary_28
Group_1467			rufipogon_boundary_302
Group_1468			rufipogon_boundary_386
Group_1469			rufipogon_boundary_390
Group_1470			rufipogon_boundary_391
Group_1471			rufipogon_boundary_417
Group_1472			rufipogon_boundary_431
Group_1473			
Group_1474			rufipogon_boundary_471
Group_1475			rufipogon_boundary_472
Group_1476			rufipogon_boundary_500
Group_1477			rufipogon_boundary_525
Group_1478			rufipogon_boundary_578
Group_1479			rufipogon_boundary_590
Group_1480			rufipogon_boundary_592
Group_1481			rufipogon_boundary_603
Group_1482			rufipogon_boundary_634
Group_1483			rufipogon_boundary_672
Group_1484			rufipogon_boundary_678
Group_1485			rufipogon_boundary_700
Group_1486			rufipogon_boundary_704
Group_1487			rufipogon_boundary_722
Group_1488			rufipogon_boundary_725
Group_1489			rufipogon_boundary_726
Group_1490			rufipogon_boundary_727
Group_1491			rufipogon_boundary_73
Group_1492			rufipogon_boundary_738
Group_1493			rufipogon_boundary_774
Group_1494			rufipogon_boundary_785
Group_1495			rufipogon_boundary_802
Group_1496			rufipogon_boundary_854
Group_1497			rufipogon_boundary_866
Group_1498			rufipogon_boundary_89
Group_1499			rufipogon_boundary_915
Group_1500			rufipogon_boundary_928
Group_1501			rufipogon_boundary_965
Group_1502			rufipogon_boundary_969
Group_1503			rufipogon_boundary_985
Group_1504			rufipogon_boundary_986
Group_1505			rufipogon_boundary_1025
Group_1506			rufipogon_boundary_1054
Group_1507			rufipogon_boundary_1082
Group_1508			rufipogon_boundary_1083
Group_1509			rufipogon_boundary_1103
Group_1510			rufipogon_boundary_1115
Group_1511			rufipogon_boundary_1213
Group_1512			rufipogon_boundary_1219
Group_1513			rufipogon_boundary_1220
Group_1514			rufipogon_boundary_1240
Group_1515			rufipogon_boundary_1254
Group_1516			rufipogon_boundary_1258
Group_1517			rufipogon_boundary_1259
Group_1518			rufipogon_boundary_1262
Group_1519			rufipogon_boundary_1286
Group_1520			rufipogon_boundary_1295
Group_1521			rufipogon_boundary_1346
Group_1522			rufipogon_boundary_1375
Group_1523			rufipogon_boundary_1391
Group_1524			rufipogon_boundary_1414
Group_1525			rufipogon_boundary_152
Group_1526			rufipogon_boundary_273
Group_1527			rufipogon_boundary_280
Group_1528			
Group_1529			rufipogon_boundary_35
Group_1530			rufipogon_boundary_412
Group_1531			rufipogon_boundary_464
Group_1532			rufipogon_boundary_481
Group_1533			rufipogon_boundary_487
Group_1534			rufipogon_boundary_491
Group_1535			rufipogon_boundary_52
Group_1536			
Group_1537			rufipogon_boundary_591
Group_1538			rufipogon_boundary_622
Group_1539			rufipogon_boundary_637
Group_1540			rufipogon_boundary_639
Group_1541			rufipogon_boundary_646
Group_1542			rufipogon_boundary_648
Group_1543			rufipogon_boundary_686
Group_1544			rufipogon_boundary_703
Group_1545			rufipogon_boundary_801

Group_1546			rufipogon_boundary_83
Group_1547			rufipogon_boundary_842
Group_1548			
Group_1549			rufipogon_boundary_87
Group_1550			rufipogon_boundary_92
Group_1551			rufipogon_boundary_927
Group_1552			
Group_1553			rufipogon_boundary_966
Group_1554			rufipogon_boundary_967
Group_1555			rufipogon_boundary_997
Group_1556			
Group_1557			
Group_1558			
Group_1559			
Group_1560			
Group_1561			
Group_1562			
Group_1563			
Group_1564			
Group_1565			
Group_1566			
Group_1567			
Group_1568			
Group_1569			
Group_1570			
Group_1571			
Group_1572			
Group_1573			
Group_1574			
Group_1575			
Group_1576			
Group_1577			
Group_1578			
Group_1579			
Group_1580			
Group_1581			
Group_1582			
Group_1583			
Group_1584			
Group_1585			
Group_1586			
Group_1587			
Group_1588			
Group_1589			
Group_1590			
Group_1591			
Group_1592			
Group_1593			
Group_1594			
Group_1595			
Group_1596			
Group_1597			
Group_1598			
Group_1599			
Group_1600			
Group_1601			
Group_1602			
Group_1603			
Group_1604			
Group_1605			
Group_1606			
Group_1607			
Group_1608			
Group_1609			
Group_1610			
Group_1611			
Group_1612			
Group_1613			
Group_1614			
Group_1615			
Group_1616			
Group_1617			
Group_1618			
Group_1619			
Group_1620			
Group_1621			
Group_1622			
Group_1623			
Group_1624			
Group_1625			
Group_1626			
Group_1627			
Group_1628			
Group_1629			
Group_1630			

Nipponbare	IR64
nipponbare_boundary_141;nipponbare_boundary_919;nipponbare_boundary_920;nipponbare_boundary_142	IR64_boundary_145;IR64_boundary_884
nipponbare_boundary_351;nipponbare_boundary_352;nipponbare_boundary_353	IR64_boundary_342;IR64_boundary_343;IR64_boundary_344
nipponbare_boundary_411;nipponbare_boundary_412	IR64_boundary_408;IR64_boundary_409;IR64_boundary_410
nipponbare_boundary_113;nipponbare_boundary_114;nipponbare_boundary_115	IR64_boundary_120;IR64_boundary_121
nipponbare_boundary_1171;nipponbare_boundary_1172	IR64_boundary_1128;IR64_boundary_1129;IR64_boundary_1130;IR64_boundary_1131
nipponbare_boundary_1653;nipponbare_boundary_1652	IR64_boundary_1577;IR64_boundary_1578
nipponbare_boundary_347;nipponbare_boundary_348	IR64_boundary_339;IR64_boundary_714;IR64_boundary_715
nipponbare_boundary_618;nipponbare_boundary_619;nipponbare_boundary_620	IR64_boundary_621;IR64_boundary_622
nipponbare_boundary_57;nipponbare_boundary_55;nipponbare_boundary_56	IR64_boundary_55;IR64_boundary_56
nipponbare_boundary_150;nipponbare_boundary_152;nipponbare_boundary_153;nipponbare_boundary_151	IR64_boundary_163;IR64_boundary_162;IR64_boundary_161
nipponbare_boundary_1675;nipponbare_boundary_1676	IR64_boundary_1595;IR64_boundary_1596
nipponbare_boundary_1342	IR64_boundary_1280;IR64_boundary_1281
nipponbare_boundary_1703;nipponbare_boundary_1702	IR64_boundary_1633
nipponbare_boundary_190;nipponbare_boundary_868;nipponbare_boundary_191	IR64_boundary_204;IR64_boundary_205
nipponbare_boundary_891;nipponbare_boundary_892;nipponbare_boundary_171	IR64_boundary_857;IR64_boundary_858
nipponbare_boundary_1026	
nipponbare_boundary_1093;nipponbare_boundary_1092	IR64_boundary_1067;IR64_boundary_1068
nipponbare_boundary_212;nipponbare_boundary_213;nipponbare_boundary_214	IR64_boundary_215;IR64_boundary_214
	IR64_boundary_147;IR64_boundary_148;IR64_boundary_149
nipponbare_boundary_1472;nipponbare_boundary_1471	IR64_boundary_1395;IR64_boundary_1393;IR64_boundary_1394
nipponbare_boundary_1692;nipponbare_boundary_1693	IR64_boundary_1624;IR64_boundary_1623
nipponbare_boundary_1691;nipponbare_boundary_1690	IR64_boundary_1621;IR64_boundary_1622;IR64_boundary_1510
nipponbare_boundary_218;nipponbare_boundary_847	IR64_boundary_218
nipponbare_boundary_421	IR64_boundary_416;IR64_boundary_414;IR64_boundary_415
	IR64_boundary_421;IR64_boundary_422;IR64_boundary_423
nipponbare_boundary_428	IR64_boundary_428;IR64_boundary_429
nipponbare_boundary_1571;nipponbare_boundary_1570	IR64_boundary_1491;IR64_boundary_1492
nipponbare_boundary_462;nipponbare_boundary_463	IR64_boundary_458;IR64_boundary_457
nipponbare_boundary_1228;nipponbare_boundary_503;nipponbare_boundary_502	
nipponbare_boundary_504;nipponbare_boundary_505;nipponbare_boundary_1226	
nipponbare_boundary_904;nipponbare_boundary_903	IR64_boundary_866;IR64_boundary_867;IR64_boundary_868
nipponbare_boundary_1117;nipponbare_boundary_1118	IR64_boundary_1080;IR64_boundary_1081;IR64_boundary_1079
nipponbare_boundary_131	IR64_boundary_138;IR64_boundary_137
nipponbare_boundary_1174;nipponbare_boundary_1175	IR64_boundary_1136;IR64_boundary_1137
nipponbare_boundary_899	IR64_boundary_174;IR64_boundary_175;IR64_boundary_862
nipponbare_boundary_1003;nipponbare_boundary_1004	IR64_boundary_986;IR64_boundary_987;IR64_boundary_988
nipponbare_boundary_400	IR64_boundary_389;IR64_boundary_388
nipponbare_boundary_1631;nipponbare_boundary_1632	IR64_boundary_1562;IR64_boundary_1563;IR64_boundary_1564
nipponbare_boundary_1659;nipponbare_boundary_1660;nipponbare_boundary_1661;nipponbare_boundary_1662	IR64_boundary_1587
nipponbare_boundary_672;nipponbare_boundary_673	IR64_boundary_675;IR64_boundary_676;IR64_boundary_677
nipponbare_boundary_928;nipponbare_boundary_128	IR64_boundary_132;IR64_boundary_891;IR64_boundary_892;IR64_boundary_134
nipponbare_boundary_99;nipponbare_boundary_100	IR64_boundary_111;IR64_boundary_112
nipponbare_boundary_1363	IR64_boundary_1386;IR64_boundary_1298
nipponbare_boundary_9;nipponbare_boundary_10	IR64_boundary_17;IR64_boundary_18
nipponbare_boundary_1665;nipponbare_boundary_1666	IR64_boundary_1588
nipponbare_boundary_185	IR64_boundary_196
nipponbare_boundary_1704;nipponbare_boundary_1705	IR64_boundary_1635;IR64_boundary_1636;IR64_boundary_1634
nipponbare_boundary_875;nipponbare_boundary_186	IR64_boundary_199;IR64_boundary_200
nipponbare_boundary_1811	IR64_boundary_1709;IR64_boundary_1708
nipponbare_boundary_359;nipponbare_boundary_360;nipponbare_boundary_718	IR64_boundary_349;IR64_boundary_350
nipponbare_boundary_415;nipponbare_boundary_413;nipponbare_boundary_414	IR64_boundary_412;IR64_boundary_411
nipponbare_boundary_496	IR64_boundary_494
	IR64_boundary_556;IR64_boundary_557
nipponbare_boundary_1024;nipponbare_boundary_1025	
nipponbare_boundary_1063;nipponbare_boundary_1064	IR64_boundary_1040;IR64_boundary_1041
nipponbare_boundary_1079;nipponbare_boundary_1080	IR64_boundary_1052;IR64_boundary_1053
nipponbare_boundary_393;nipponbare_boundary_394	IR64_boundary_381
nipponbare_boundary_915;nipponbare_boundary_916	IR64_boundary_880;IR64_boundary_881;IR64_boundary_879
	IR64_boundary_908;IR64_boundary_909
nipponbare_boundary_1257;nipponbare_boundary_1258;nipponbare_boundary_1256	IR64_boundary_1200;IR64_boundary_1201;IR64_boundary_1199
nipponbare_boundary_200	IR64_boundary_208;IR64_boundary_209
nipponbare_boundary_170;nipponbare_boundary_893	IR64_boundary_859;IR64_boundary_182
nipponbare_boundary_1183;nipponbare_boundary_1184	IR64_boundary_1145;IR64_boundary_1146
nipponbare_boundary_1795;nipponbare_boundary_1794	IR64_boundary_1699;IR64_boundary_1700
nipponbare_boundary_540;nipponbare_boundary_541;nipponbare_boundary_542	IR64_boundary_549
nipponbare_boundary_978;nipponbare_boundary_979	
nipponbare_boundary_1075	IR64_boundary_1048;IR64_boundary_1049
nipponbare_boundary_1495;nipponbare_boundary_1496;nipponbare_boundary_1497	IR64_boundary_1408;IR64_boundary_1409
nipponbare_boundary_1216;nipponbare_boundary_1217	IR64_boundary_1172;IR64_boundary_1173
nipponbare_boundary_1249;nipponbare_boundary_1250	IR64_boundary_1194;IR64_boundary_1195
	IR64_boundary_1246;IR64_boundary_1247
nipponbare_boundary_1319	IR64_boundary_1269
nipponbare_boundary_1452;nipponbare_boundary_1453	
nipponbare_boundary_1458;nipponbare_boundary_1459	IR64_boundary_1380;IR64_boundary_1381
nipponbare_boundary_1467;nipponbare_boundary_1468	IR64_boundary_1390;IR64_boundary_1391
nipponbare_boundary_1483	
	IR64_boundary_855
nipponbare_boundary_11;nipponbare_boundary_12	
nipponbare_boundary_374	IR64_boundary_363
nipponbare_boundary_376	IR64_boundary_364
nipponbare_boundary_380;nipponbare_boundary_381	IR64_boundary_367;IR64_boundary_368
nipponbare_boundary_416;nipponbare_boundary_417	IR64_boundary_413
nipponbare_boundary_572;nipponbare_boundary_573	
nipponbare_boundary_594	IR64_boundary_603;IR64_boundary_604
nipponbare_boundary_7;nipponbare_boundary_8	IR64_boundary_12
nipponbare_boundary_725;nipponbare_boundary_726	
nipponbare_boundary_781;nipponbare_boundary_782	
nipponbare_boundary_783;nipponbare_boundary_784	
nipponbare_boundary_901	IR64_boundary_863;IR64_boundary_864
nipponbare_boundary_1030	IR64_boundary_265
nipponbare_boundary_1035	IR64_boundary_1011;IR64_boundary_1012
nipponbare_boundary_1038;nipponbare_boundary_1039	IR64_boundary_1019;IR64_boundary_1020
nipponbare_boundary_1051;nipponbare_boundary_1052	IR64_boundary_1029
nipponbare_boundary_1085;nipponbare_boundary_1086	IR64_boundary_1061;IR64_boundary_1062
nipponbare_boundary_1087;nipponbare_boundary_1088	IR64_boundary_1063;IR64_boundary_1064
nipponbare_boundary_1177	IR64_boundary_1138;IR64_boundary_1139
nipponbare_boundary_1597;nipponbare_boundary_1598	IR64_boundary_1519;IR64_boundary_1520
nipponbare_boundary_232;nipponbare_boundary_233	IR64_boundary_237
nipponbare_boundary_243	IR64_boundary_243;IR64_boundary_244
nipponbare_boundary_356	IR64_boundary_718
nipponbare_boundary_497;nipponbare_boundary_498	IR64_boundary_495;IR64_boundary_496
nipponbare_boundary_953;nipponbare_boundary_954	IR64_boundary_917;IR64_boundary_918
nipponbare_boundary_1045	IR64_boundary_1024
nipponbare_boundary_1101;nipponbare_boundary_1102	
nipponbare_boundary_1260;nipponbare_boundary_1261	IR64_boundary_1209;IR64_boundary_1210;IR64_boundary_1208
nipponbare_boundary_1448	IR64_boundary_1373
nipponbare_boundary_1682;nipponbare_boundary_1683;nipponbare_boundary_1681	IR64_boundary_1608;IR64_boundary_1607
nipponbare_boundary_396	IR64_boundary_382
nipponbare_boundary_450;nipponbare_boundary_449	IR64_boundary_446
nipponbare_boundary_917;nipponbare_boundary_918	IR64_boundary_882
nipponbare_boundary_14	IR64_boundary_14;IR64_boundary_13
nipponbare_boundary_1522	IR64_boundary_1444;IR64_boundary_1445
nipponbare_boundary_1358	IR64_boundary_1384;IR64_boundary_1295
nipponbare_boundary_1328	IR64_boundary_1273;IR64_boundary_1274
nipponbare_boundary_929;nipponbare_boundary_123	IR64_boundary_893;IR64_boundary_894
nipponbare_boundary_1372;nipponbare_boundary_1373;nipponbare_boundary_1374	IR64_boundary_1310
nipponbare_boundary_1433	IR64_boundary_1352;IR64_boundary_1351
nipponbare_boundary_159;nipponbare_boundary_160	IR64_boundary_169;IR64_boundary_170
nipponbare_boundary_1725;nipponbare_boundary_1724	IR64_boundary_1649;IR64_boundary_1650
nipponbare_boundary_443;nipponbare_boundary_444	IR64_boundary_438
nipponbare_boundary_454	IR64_boundary_450;IR64_boundary_451
nipponbare_boundary_600;nipponbare_boundary_601	IR64_boundary_613;IR64_boundary_614
nipponbare_boundary_614;nipponbare_boundary_615	IR64_boundary_619;IR64_boundary_620
nipponbare_boundary_646	IR64_boundary_645;IR64_boundary_646
	IR64_boundary_673;IR64_boundary_674
nipponbare_boundary_913;nipponbare_boundary_914	IR64_boundary_878;IR64_boundary_877

nipponbare_boundary_1011;nipponbare_boundary_1010	IR64_boundary_995;IR64_boundary_994
nipponbare_boundary_1104;nipponbare_boundary_1105	IR64_boundary_1073
nipponbare_boundary_1447;nipponbare_boundary_1446	IR64_boundary_1372
nipponbare_boundary_755;nipponbare_boundary_757;nipponbare_boundary_756	IR64_boundary_756;IR64_boundary_755
nipponbare_boundary_576;nipponbare_boundary_574;nipponbare_boundary_575	IR64_boundary_586
nipponbare_boundary_1214;nipponbare_boundary_1215	IR64_boundary_1170;IR64_boundary_1171
nipponbare_boundary_1241	IR64_boundary_1184;IR64_boundary_1185
nipponbare_boundary_145	IR64_boundary_153
nipponbare_boundary_1303;nipponbare_boundary_1304	
nipponbare_boundary_1320	IR64_boundary_1270
nipponbare_boundary_1340;nipponbare_boundary_1341	IR64_boundary_1279
nipponbare_boundary_156	IR64_boundary_168
nipponbare_boundary_1462;nipponbare_boundary_1463	IR64_boundary_1387
nipponbare_boundary_1558;nipponbare_boundary_1559	
nipponbare_boundary_1567;nipponbare_boundary_429	IR64_boundary_1486
	IR64_boundary_1488;IR64_boundary_1487
nipponbare_boundary_172;nipponbare_boundary_886	IR64_boundary_188
nipponbare_boundary_879	IR64_boundary_190;IR64_boundary_845
nipponbare_boundary_1667	IR64_boundary_1589;IR64_boundary_1590
nipponbare_boundary_183;nipponbare_boundary_184	IR64_boundary_195
nipponbare_boundary_1698	IR64_boundary_1630
nipponbare_boundary_1741	
nipponbare_boundary_1806;nipponbare_boundary_1805	IR64_boundary_1704
	IR64_boundary_251
nipponbare_boundary_276	IR64_boundary_282;IR64_boundary_283
nipponbare_boundary_313	IR64_boundary_321
nipponbare_boundary_350	IR64_boundary_341
nipponbare_boundary_355	IR64_boundary_345;IR64_boundary_346
nipponbare_boundary_371	IR64_boundary_360
nipponbare_boundary_728	IR64_boundary_365
	IR64_boundary_371;IR64_boundary_372
nipponbare_boundary_410;nipponbare_boundary_409	IR64_boundary_406
nipponbare_boundary_422;nipponbare_boundary_423	
nipponbare_boundary_431;nipponbare_boundary_430	IR64_boundary_430
nipponbare_boundary_442	
nipponbare_boundary_513	IR64_boundary_507
nipponbare_boundary_592	IR64_boundary_600;IR64_boundary_601
nipponbare_boundary_597	IR64_boundary_607
nipponbare_boundary_603	
nipponbare_boundary_716;nipponbare_boundary_717	
nipponbare_boundary_734	IR64_boundary_727;IR64_boundary_728
nipponbare_boundary_737	IR64_boundary_729
nipponbare_boundary_792;nipponbare_boundary_793	
nipponbare_boundary_818	IR64_boundary_793
nipponbare_boundary_87;nipponbare_boundary_88	
nipponbare_boundary_958	IR64_boundary_921;IR64_boundary_922
nipponbare_boundary_988	IR64_boundary_946
nipponbare_boundary_1029	
nipponbare_boundary_1040;nipponbare_boundary_253	IR64_boundary_1021
nipponbare_boundary_1180	IR64_boundary_1141;IR64_boundary_1142
nipponbare_boundary_1199	IR64_boundary_1154;IR64_boundary_1153
nipponbare_boundary_1212	IR64_boundary_1168;IR64_boundary_1169
nipponbare_boundary_1366;nipponbare_boundary_1367	IR64_boundary_1304
nipponbare_boundary_1457;nipponbare_boundary_1456	IR64_boundary_1379
nipponbare_boundary_1576;nipponbare_boundary_1577	IR64_boundary_1499;IR64_boundary_1500
nipponbare_boundary_887;nipponbare_boundary_888	IR64_boundary_850;IR64_boundary_851
nipponbare_boundary_241	
nipponbare_boundary_229	IR64_boundary_232;IR64_boundary_231
nipponbare_boundary_22	IR64_boundary_27
nipponbare_boundary_324	IR64_boundary_329
nipponbare_boundary_729;nipponbare_boundary_730	IR64_boundary_722;IR64_boundary_723
nipponbare_boundary_506	IR64_boundary_498;IR64_boundary_499
nipponbare_boundary_582;nipponbare_boundary_583	IR64_boundary_591;IR64_boundary_592
nipponbare_boundary_626;nipponbare_boundary_627	
nipponbare_boundary_780	IR64_boundary_775;IR64_boundary_776
nipponbare_boundary_1020;nipponbare_boundary_1019	IR64_boundary_1001
nipponbare_boundary_1385;nipponbare_boundary_1389;nipponbare_boundary_1386	IR64_boundary_1320
nipponbare_boundary_1476	IR64_boundary_1397
nipponbare_boundary_1679;nipponbare_boundary_1680	IR64_boundary_1601;IR64_boundary_1602;IR64_boundary_1603
nipponbare_boundary_1761;nipponbare_boundary_1762	IR64_boundary_1683;IR64_boundary_65
nipponbare_boundary_848	IR64_boundary_220;IR64_boundary_219
nipponbare_boundary_764;nipponbare_boundary_765	IR64_boundary_767
nipponbare_boundary_1546;nipponbare_boundary_1547	IR64_boundary_1472;IR64_boundary_1473
nipponbare_boundary_546;nipponbare_boundary_1154	IR64_boundary_974
nipponbare_boundary_1173	IR64_boundary_1132;IR64_boundary_1133
nipponbare_boundary_1204	IR64_boundary_1157;IR64_boundary_1158
nipponbare_boundary_1208;nipponbare_boundary_1207	IR64_boundary_1160;IR64_boundary_1161
nipponbare_boundary_1300;nipponbare_boundary_1301	IR64_boundary_1682
nipponbare_boundary_1521	IR64_boundary_1440;IR64_boundary_1441
nipponbare_boundary_1564	IR64_boundary_1483
nipponbare_boundary_882;nipponbare_boundary_178;nipponbare_boundary_179	
nipponbare_boundary_205;nipponbare_boundary_206	IR64_boundary_210
nipponbare_boundary_208;nipponbare_boundary_209	IR64_boundary_212
nipponbare_boundary_362	IR64_boundary_354;IR64_boundary_355
nipponbare_boundary_401;nipponbare_boundary_402	IR64_boundary_392
nipponbare_boundary_538;nipponbare_boundary_539	IR64_boundary_548
nipponbare_boundary_564;nipponbare_boundary_563	IR64_boundary_572;IR64_boundary_573
nipponbare_boundary_662	IR64_boundary_639;IR64_boundary_638
nipponbare_boundary_688;nipponbare_boundary_675	IR64_boundary_697;IR64_boundary_698
nipponbare_boundary_881;nipponbare_boundary_180;nipponbare_boundary_181	IR64_boundary_847
nipponbare_boundary_894;nipponbare_boundary_895	
nipponbare_boundary_896	IR64_boundary_181
nipponbare_boundary_927	IR64_boundary_890
nipponbare_boundary_996	IR64_boundary_966;IR64_boundary_967
nipponbare_boundary_1353;nipponbare_boundary_1354	IR64_boundary_1290;IR64_boundary_1291
nipponbare_boundary_1356;nipponbare_boundary_1357	IR64_boundary_1293
nipponbare_boundary_486;nipponbare_boundary_487	IR64_boundary_480;IR64_boundary_481
nipponbare_boundary_834	IR64_boundary_815;IR64_boundary_816
nipponbare_boundary_843;nipponbare_boundary_844;nipponbare_boundary_845	IR64_boundary_824;IR64_boundary_825
nipponbare_boundary_911;nipponbare_boundary_912	IR64_boundary_874;IR64_boundary_875
nipponbare_boundary_1759	IR64_boundary_1680;IR64_boundary_1679
nipponbare_boundary_689	IR64_boundary_700;IR64_boundary_699
nipponbare_boundary_1418;nipponbare_boundary_1419	IR64_boundary_1346;IR64_boundary_1347
nipponbare_boundary_1583;nipponbare_boundary_1584	IR64_boundary_1615;IR64_boundary_1616
nipponbare_boundary_1763;nipponbare_boundary_1764	IR64_boundary_1684;IR64_boundary_1685
nipponbare_boundary_786;nipponbare_boundary_787;nipponbare_boundary_788	IR64_boundary_779
nipponbare_boundary_854;nipponbare_boundary_855	IR64_boundary_831;IR64_boundary_832;IR64_boundary_833
nipponbare_boundary_97;nipponbare_boundary_98	IR64_boundary_104;IR64_boundary_105;IR64_boundary_106
nipponbare_boundary_1245	
nipponbare_boundary_1318	IR64_boundary_1262;IR64_boundary_1263
nipponbare_boundary_1322	IR64_boundary_1272
nipponbare_boundary_155;nipponbare_boundary_154	IR64_boundary_167
nipponbare_boundary_1436	IR64_boundary_1358
nipponbare_boundary_1440	IR64_boundary_1363
	IR64_boundary_1424
nipponbare_boundary_1550	IR64_boundary_1474
nipponbare_boundary_1573	IR64_boundary_1493
	IR64_boundary_1508
nipponbare_boundary_1581;nipponbare_boundary_1580	IR64_boundary_1507
	IR64_boundary_1521
nipponbare_boundary_1694	IR64_boundary_1625
nipponbare_boundary_1742;nipponbare_boundary_1743	IR64_boundary_1661;IR64_boundary_1662
nipponbare_boundary_867	IR64_boundary_836;IR64_boundary_837
nipponbare_boundary_217	IR64_boundary_217
	IR64_boundary_226;IR64_boundary_227
nipponbare_boundary_245	IR64_boundary_246
	IR64_boundary_22
nipponbare_boundary_258	

nipponbare_boundary_230	IR64_boundary_233
nipponbare_boundary_21	IR64_boundary_25
nipponbare_boundary_277	IR64_boundary_284
nipponbare_boundary_278	IR64_boundary_285
nipponbare_boundary_279	IR64_boundary_286
nipponbare_boundary_323	IR64_boundary_328
nipponbare_boundary_386	IR64_boundary_373
nipponbare_boundary_419	
nipponbare_boundary_435	
nipponbare_boundary_446	
nipponbare_boundary_447	
	IR64_boundary_466;IR64_boundary_467
nipponbare_boundary_475;nipponbare_boundary_476	
nipponbare_boundary_477;nipponbare_boundary_478	IR64_boundary_475
nipponbare_boundary_479	IR64_boundary_476
nipponbare_boundary_481;nipponbare_boundary_482	IR64_boundary_478
nipponbare_boundary_490	IR64_boundary_484;IR64_boundary_485
nipponbare_boundary_511	IR64_boundary_503
	IR64_boundary_577
nipponbare_boundary_570	IR64_boundary_581
	IR64_boundary_583;IR64_boundary_584
nipponbare_boundary_43	
nipponbare_boundary_605;nipponbare_boundary_1106	
nipponbare_boundary_624	IR64_boundary_626
nipponbare_boundary_692;nipponbare_boundary_693	IR64_boundary_703
nipponbare_boundary_713	
nipponbare_boundary_738;nipponbare_boundary_739	IR64_boundary_730
	IR64_boundary_763
nipponbare_boundary_779;nipponbare_boundary_778	IR64_boundary_774
nipponbare_boundary_814	IR64_boundary_789
nipponbare_boundary_890	IR64_boundary_856
nipponbare_boundary_932	IR64_boundary_897
	IR64_boundary_900
nipponbare_boundary_939	
nipponbare_boundary_1012	IR64_boundary_996
nipponbare_boundary_1013	IR64_boundary_997
nipponbare_boundary_1066	
nipponbare_boundary_606;nipponbare_boundary_1108	
nipponbare_boundary_1110	IR64_boundary_1074
nipponbare_boundary_1190;nipponbare_boundary_1191	
nipponbare_boundary_134	IR64_boundary_141
nipponbare_boundary_1209	IR64_boundary_1162
nipponbare_boundary_515	
nipponbare_boundary_1286;nipponbare_boundary_1287	
nipponbare_boundary_1435	IR64_boundary_1356
nipponbare_boundary_1454	IR64_boundary_1377;IR64_boundary_1378
nipponbare_boundary_164	IR64_boundary_176;IR64_boundary_177
nipponbare_boundary_1500;nipponbare_boundary_1499	
	IR64_boundary_1599;IR64_boundary_1600
nipponbare_boundary_874	IR64_boundary_201
nipponbare_boundary_873	
nipponbare_boundary_216	IR64_boundary_216
nipponbare_boundary_254;nipponbare_boundary_255	
	IR64_boundary_1008;IR64_boundary_259;IR64_boundary_261
nipponbare_boundary_325	IR64_boundary_330;IR64_boundary_331
nipponbare_boundary_337	IR64_boundary_334
nipponbare_boundary_361	IR64_boundary_351
nipponbare_boundary_32;nipponbare_boundary_31	
nipponbare_boundary_1569	IR64_boundary_1490
nipponbare_boundary_469	IR64_boundary_468
nipponbare_boundary_495	IR64_boundary_493
nipponbare_boundary_507;nipponbare_boundary_508	IR64_boundary_500
nipponbare_boundary_68;nipponbare_boundary_69	IR64_boundary_72
nipponbare_boundary_745	IR64_boundary_740
nipponbare_boundary_812	IR64_boundary_788
nipponbare_boundary_902	IR64_boundary_865
nipponbare_boundary_933	
nipponbare_boundary_1017;nipponbare_boundary_1018	IR64_boundary_1000
nipponbare_boundary_1054	IR64_boundary_1032
	IR64_boundary_1189;IR64_boundary_1188;IR64_boundary_1190
nipponbare_boundary_1461;nipponbare_boundary_1362	IR64_boundary_1296;IR64_boundary_1297
	IR64_boundary_1502;IR64_boundary_1501
nipponbare_boundary_1658	
nipponbare_boundary_247	IR64_boundary_248
nipponbare_boundary_267;nipponbare_boundary_1021	IR64_boundary_266
nipponbare_boundary_34	IR64_boundary_31
nipponbare_boundary_41;nipponbare_boundary_42	IR64_boundary_43
nipponbare_boundary_743;nipponbare_boundary_744	IR64_boundary_735;IR64_boundary_736
nipponbare_boundary_1181	IR64_boundary_1143
nipponbare_boundary_1145	IR64_boundary_1108
nipponbare_boundary_1129	IR64_boundary_1091
nipponbare_boundary_1153	
nipponbare_boundary_1265	IR64_boundary_1217
nipponbare_boundary_1337;nipponbare_boundary_1338	
nipponbare_boundary_132;nipponbare_boundary_133	IR64_boundary_139
nipponbare_boundary_1481	IR64_boundary_1400
	IR64_boundary_1443
	IR64_boundary_1475
nipponbare_boundary_149	IR64_boundary_160;IR64_boundary_159
	IR64_boundary_1619;IR64_boundary_1617
nipponbare_boundary_1709	IR64_boundary_1640
nipponbare_boundary_1747	IR64_boundary_1664
nipponbare_boundary_1766	
nipponbare_boundary_177	
nipponbare_boundary_1792;nipponbare_boundary_1793	
nipponbare_boundary_187	
nipponbare_boundary_210	IR64_boundary_213
nipponbare_boundary_458	IR64_boundary_454
nipponbare_boundary_46	IR64_boundary_44;IR64_boundary_45
nipponbare_boundary_553	IR64_boundary_565;IR64_boundary_566
nipponbare_boundary_636;nipponbare_boundary_637	IR64_boundary_636
nipponbare_boundary_670;nipponbare_boundary_671	
	IR64_boundary_62
nipponbare_boundary_71	IR64_boundary_75;IR64_boundary_76
nipponbare_boundary_838	IR64_boundary_819
	IR64_boundary_853;IR64_boundary_854
nipponbare_boundary_922	IR64_boundary_886;IR64_boundary_143
nipponbare_boundary_967	IR64_boundary_938
nipponbare_boundary_969	IR64_boundary_940;IR64_boundary_939
nipponbare_boundary_101	IR64_boundary_113
nipponbare_boundary_119;nipponbare_boundary_120	IR64_boundary_127
nipponbare_boundary_1317	IR64_boundary_1260;IR64_boundary_1261
nipponbare_boundary_1491	IR64_boundary_1406;IR64_boundary_1407
nipponbare_boundary_1687	IR64_boundary_1618
nipponbare_boundary_1646;nipponbare_boundary_1647;nipponbare_boundary_1648	IR64_boundary_1575
nipponbare_boundary_1655	IR64_boundary_1580;IR64_boundary_1581
nipponbare_boundary_1674	IR64_boundary_1592;IR64_boundary_1593
nipponbare_boundary_1734;nipponbare_boundary_1735;nipponbare_boundary_1152	IR64_boundary_1656
nipponbare_boundary_1808;nipponbare_boundary_1809	IR64_boundary_1705;IR64_boundary_1706
nipponbare_boundary_256	IR64_boundary_257;IR64_boundary_256
nipponbare_boundary_289	IR64_boundary_308
nipponbare_boundary_294;nipponbare_boundary_295	IR64_boundary_314
nipponbare_boundary_557;nipponbare_boundary_558	IR64_boundary_570
nipponbare_boundary_700;nipponbare_boundary_699	IR64_boundary_705

nipponbare_boundary_1037;nipponbare_boundary_1036	IR64_boundary_1014
nipponbare_boundary_1796;nipponbare_boundary_1797	IR64_boundary_1701
nipponbare_boundary_398	IR64_boundary_384
nipponbare_boundary_296;nipponbare_boundary_300;nipponbare_boundary_302	IR64_boundary_317;IR64_boundary_318
nipponbare_boundary_1314;nipponbare_boundary_1315	IR64_boundary_1255
nipponbare_boundary_1757;nipponbare_boundary_1769;nipponbare_boundary_1770	IR64_boundary_1690
nipponbare_boundary_4	IR64_boundary_3;IR64_boundary_4
nipponbare_boundary_536	IR64_boundary_545;IR64_boundary_546
nipponbare_boundary_1094;nipponbare_boundary_1095	IR64_boundary_1070
nipponbare_boundary_135	
nipponbare_boundary_1225	
nipponbare_boundary_1280;nipponbare_boundary_1281	
nipponbare_boundary_1293	
nipponbare_boundary_1309	
nipponbare_boundary_1333	
nipponbare_boundary_147	
	IR64_boundary_1364
	IR64_boundary_1376
nipponbare_boundary_1530	IR64_boundary_1456
nipponbare_boundary_1531	
	IR64_boundary_1471
	IR64_boundary_1476
	IR64_boundary_1478
nipponbare_boundary_1560	IR64_boundary_1480
nipponbare_boundary_1600	IR64_boundary_1526
nipponbare_boundary_1601	IR64_boundary_1530
	IR64_boundary_191;IR64_boundary_192
nipponbare_boundary_1732	
nipponbare_boundary_1412	IR64_boundary_1338
	IR64_boundary_250
nipponbare_boundary_259	
	IR64_boundary_234
nipponbare_boundary_266	
	IR64_boundary_274
nipponbare_boundary_274	
nipponbare_boundary_321	IR64_boundary_325
	IR64_boundary_327
nipponbare_boundary_322	
nipponbare_boundary_327	
nipponbare_boundary_26	
nipponbare_boundary_378	
nipponbare_boundary_382	
nipponbare_boundary_387	
nipponbare_boundary_395	
	IR64_boundary_33
	IR64_boundary_34
nipponbare_boundary_420	
	IR64_boundary_441;IR64_boundary_442
	IR64_boundary_11
nipponbare_boundary_571	
nipponbare_boundary_580	
nipponbare_boundary_591	IR64_boundary_599
	IR64_boundary_624
nipponbare_boundary_625	
	IR64_boundary_669
nipponbare_boundary_715	
nipponbare_boundary_719	
nipponbare_boundary_750	IR64_boundary_749
nipponbare_boundary_767	
nipponbare_boundary_952	IR64_boundary_913
nipponbare_boundary_960	IR64_boundary_924
nipponbare_boundary_106	
	IR64_boundary_954
nipponbare_boundary_107	
nipponbare_boundary_1031;nipponbare_boundary_1032	
nipponbare_boundary_1142	
nipponbare_boundary_1143	
	IR64_boundary_1111
	IR64_boundary_1114
nipponbare_boundary_1178	IR64_boundary_1140
nipponbare_boundary_1192;nipponbare_boundary_1193	
nipponbare_boundary_1331	
nipponbare_boundary_1369	IR64_boundary_1306
nipponbare_boundary_1441	
nipponbare_boundary_1469	IR64_boundary_1392
nipponbare_boundary_1474;nipponbare_boundary_1473	
nipponbare_boundary_1482	
nipponbare_boundary_1501	IR64_boundary_1410
nipponbare_boundary_1549;nipponbare_boundary_1548	
	IR64_boundary_185
	IR64_boundary_187
	IR64_boundary_1523;IR64_boundary_1524
	IR64_boundary_1555
	IR64_boundary_1598
nipponbare_boundary_1810	IR64_boundary_1707
nipponbare_boundary_219	IR64_boundary_221
nipponbare_boundary_244	IR64_boundary_245
nipponbare_boundary_467	IR64_boundary_462
	IR64_boundary_461
nipponbare_boundary_334;nipponbare_boundary_335;nipponbare_boundary_336	
	IR64_boundary_366
nipponbare_boundary_391	IR64_boundary_380
nipponbare_boundary_488	IR64_boundary_482
nipponbare_boundary_494	
nipponbare_boundary_578	IR64_boundary_589
nipponbare_boundary_579	IR64_boundary_590
nipponbare_boundary_593	IR64_boundary_602
nipponbare_boundary_695	
nipponbare_boundary_766	
nipponbare_boundary_906	IR64_boundary_869
nipponbare_boundary_930	IR64_boundary_895
nipponbare_boundary_949	IR64_boundary_906
nipponbare_boundary_950	IR64_boundary_907
nipponbare_boundary_956	IR64_boundary_919
nipponbare_boundary_1062	IR64_boundary_1038
nipponbare_boundary_1160	IR64_boundary_1122
nipponbare_boundary_1189	IR64_boundary_1150
nipponbare_boundary_1221;nipponbare_boundary_1222	
nipponbare_boundary_1223	IR64_boundary_1176
nipponbare_boundary_1232;nipponbare_boundary_1233	
nipponbare_boundary_1279	IR64_boundary_1229
nipponbare_boundary_1380	IR64_boundary_1317
nipponbare_boundary_162	IR64_boundary_173
nipponbare_boundary_1505;nipponbare_boundary_1506	IR64_boundary_1418
nipponbare_boundary_846	IR64_boundary_826
nipponbare_boundary_706	IR64_boundary_712
nipponbare_boundary_354;nipponbare_boundary_711	
nipponbare_boundary_366	IR64_boundary_359
nipponbare_boundary_464;nipponbare_boundary_465	IR64_boundary_459
nipponbare_boundary_92	
nipponbare_boundary_951	IR64_boundary_911;IR64_boundary_912

nipponbare_boundary_1042;nipponbare_boundary_1041	IR64_boundary_1022
nipponbare_boundary_1059	IR64_boundary_1033
nipponbare_boundary_122;nipponbare_boundary_121	IR64_boundary_128
nipponbare_boundary_1159	IR64_boundary_1121
	IR64_boundary_1301;IR64_boundary_1302
	IR64_boundary_155
nipponbare_boundary_1392	IR64_boundary_1324
	IR64_boundary_60
	IR64_boundary_41;IR64_boundary_42
	IR64_boundary_985
nipponbare_boundary_1043	IR64_boundary_1023
nipponbare_boundary_1060	IR64_boundary_1034
nipponbare_boundary_0	
nipponbare_boundary_1121	IR64_boundary_1084
nipponbare_boundary_1113	IR64_boundary_1078
nipponbare_boundary_1141	IR64_boundary_1098
nipponbare_boundary_1150	IR64_boundary_1112
nipponbare_boundary_1151	IR64_boundary_1113
nipponbare_boundary_109	IR64_boundary_115
nipponbare_boundary_1182	IR64_boundary_1144
nipponbare_boundary_1197	IR64_boundary_1151
	IR64_boundary_1193
nipponbare_boundary_1278	IR64_boundary_1228
nipponbare_boundary_117	IR64_boundary_125
nipponbare_boundary_1295	IR64_boundary_1242
nipponbare_boundary_1346	IR64_boundary_1283
nipponbare_boundary_1364	IR64_boundary_1299
nipponbare_boundary_1365	IR64_boundary_1300
nipponbare_boundary_1426;nipponbare_boundary_1427	
nipponbare_boundary_1432	IR64_boundary_1350
nipponbare_boundary_1434	IR64_boundary_1355
nipponbare_boundary_20	IR64_boundary_21
nipponbare_boundary_1574	IR64_boundary_1497
	IR64_boundary_1504
	IR64_boundary_1511;IR64_boundary_1512
nipponbare_boundary_1697	IR64_boundary_1626;IR64_boundary_1627
	IR64_boundary_1527;IR64_boundary_1528
nipponbare_boundary_1602	IR64_boundary_1531
nipponbare_boundary_1603	IR64_boundary_1532
nipponbare_boundary_1604	IR64_boundary_1533
nipponbare_boundary_1636	IR64_boundary_1571
nipponbare_boundary_1637	
nipponbare_boundary_158	
nipponbare_boundary_1640	IR64_boundary_1573
	IR64_boundary_1574
nipponbare_boundary_1650;nipponbare_boundary_1651	
nipponbare_boundary_1706	IR64_boundary_1638
nipponbare_boundary_1708	IR64_boundary_1639
nipponbare_boundary_1711	IR64_boundary_1643
nipponbare_boundary_1717	IR64_boundary_1646
nipponbare_boundary_1718	IR64_boundary_1647
nipponbare_boundary_1785	IR64_boundary_1698
nipponbare_boundary_203;nipponbare_boundary_204	
nipponbare_boundary_207	IR64_boundary_211
nipponbare_boundary_228	IR64_boundary_224
nipponbare_boundary_1	IR64_boundary_0
nipponbare_boundary_238	IR64_boundary_240
nipponbare_boundary_239	IR64_boundary_241
nipponbare_boundary_248	IR64_boundary_249
nipponbare_boundary_257	IR64_boundary_258
	IR64_boundary_276
nipponbare_boundary_275	
nipponbare_boundary_372	
nipponbare_boundary_437	IR64_boundary_431
nipponbare_boundary_445	IR64_boundary_439
nipponbare_boundary_453	IR64_boundary_448
nipponbare_boundary_455	IR64_boundary_452
nipponbare_boundary_456;nipponbare_boundary_457	
nipponbare_boundary_459	
	IR64_boundary_463;IR64_boundary_464
nipponbare_boundary_47	IR64_boundary_46
nipponbare_boundary_489	IR64_boundary_483
nipponbare_boundary_48	IR64_boundary_47
nipponbare_boundary_514	IR64_boundary_509
nipponbare_boundary_516	
	IR64_boundary_514
nipponbare_boundary_521	IR64_boundary_517
	IR64_boundary_585
nipponbare_boundary_577	IR64_boundary_588
nipponbare_boundary_608	IR64_boundary_616
	IR64_boundary_630;IR64_boundary_631
nipponbare_boundary_635	
nipponbare_boundary_682	IR64_boundary_692
nipponbare_boundary_760	IR64_boundary_762
nipponbare_boundary_761	IR64_boundary_765
nipponbare_boundary_797	IR64_boundary_783
	IR64_boundary_796
nipponbare_boundary_839	IR64_boundary_820
nipponbare_boundary_943	
nipponbare_boundary_976	IR64_boundary_944
nipponbare_boundary_995	IR64_boundary_968
	IR64_boundary_977
nipponbare_boundary_1023	IR64_boundary_1003
nipponbare_boundary_1053	IR64_boundary_1031
nipponbare_boundary_1065	IR64_boundary_1042
nipponbare_boundary_1068;nipponbare_boundary_1067	
	IR64_boundary_1057
nipponbare_boundary_1096	IR64_boundary_1071
nipponbare_boundary_1097	IR64_boundary_1072
nipponbare_boundary_1148	IR64_boundary_1110
nipponbare_boundary_17	IR64_boundary_20
nipponbare_boundary_1285	IR64_boundary_1235;IR64_boundary_1234
nipponbare_boundary_1445	IR64_boundary_1370;IR64_boundary_1371
nipponbare_boundary_1524	IR64_boundary_1446
nipponbare_boundary_1630	IR64_boundary_1559
nipponbare_boundary_1736	IR64_boundary_1657
nipponbare_boundary_237	IR64_boundary_239
nipponbare_boundary_272;nipponbare_boundary_271	IR64_boundary_278
nipponbare_boundary_2	IR64_boundary_1
nipponbare_boundary_397	IR64_boundary_383
nipponbare_boundary_403	IR64_boundary_393
nipponbare_boundary_448	IR64_boundary_445
nipponbare_boundary_452;nipponbare_boundary_451	IR64_boundary_447
nipponbare_boundary_533	IR64_boundary_528;IR64_boundary_529
nipponbare_boundary_554	IR64_boundary_568;IR64_boundary_567
nipponbare_boundary_562;nipponbare_boundary_561	IR64_boundary_571
nipponbare_boundary_599	IR64_boundary_611;IR64_boundary_612
nipponbare_boundary_643	IR64_boundary_641;IR64_boundary_642
nipponbare_boundary_758	IR64_boundary_759
nipponbare_boundary_799	IR64_boundary_785
nipponbare_boundary_897	IR64_boundary_860
nipponbare_boundary_908	IR64_boundary_873
nipponbare_boundary_971	IR64_boundary_941
nipponbare_boundary_1220	IR64_boundary_1175
nipponbare_boundary_1262	IR64_boundary_1213;IR64_boundary_1214
	IR64_boundary_1423;IR64_boundary_1422
nipponbare_boundary_1582	IR64_boundary_1614
nipponbare_boundary_1607;nipponbare_boundary_1616	IR64_boundary_1570
nipponbare_boundary_144	IR64_boundary_152

nipponbare_boundary_317	IR64_boundary_323
nipponbare_boundary_388	IR64_boundary_376
nipponbare_boundary_499;nipponbare_boundary_500	IR64_boundary_497
	IR64_boundary_652;IR64_boundary_651
	IR64_boundary_653;IR64_boundary_654
nipponbare_boundary_1350;nipponbare_boundary_1351	IR64_boundary_1288
nipponbare_boundary_1382;nipponbare_boundary_1028	IR64_boundary_1006
nipponbare_boundary_1414	IR64_boundary_1344;IR64_boundary_1345
nipponbare_boundary_16;nipponbare_boundary_15	IR64_boundary_19
nipponbare_boundary_1518;nipponbare_boundary_1517	IR64_boundary_1439
nipponbare_boundary_1592;nipponbare_boundary_1593	IR64_boundary_1514
nipponbare_boundary_1595	IR64_boundary_1515;IR64_boundary_1516
nipponbare_boundary_268	IR64_boundary_267
nipponbare_boundary_282	IR64_boundary_289;IR64_boundary_290
nipponbare_boundary_399	IR64_boundary_386;IR64_boundary_387
nipponbare_boundary_628;nipponbare_boundary_629	
nipponbare_boundary_647;nipponbare_boundary_648;nipponbare_boundary_649	
nipponbare_boundary_795;nipponbare_boundary_796	IR64_boundary_780
nipponbare_boundary_840	IR64_boundary_822
nipponbare_boundary_876	IR64_boundary_843;IR64_boundary_844
nipponbare_boundary_176	IR64_boundary_848
nipponbare_boundary_1324;nipponbare_boundary_1325;nipponbare_boundary_977	
nipponbare_boundary_1009	IR64_boundary_271;IR64_boundary_993
nipponbare_boundary_1077;nipponbare_boundary_1078	IR64_boundary_1051
	IR64_boundary_950;IR64_boundary_951
nipponbare_boundary_1006;nipponbare_boundary_1007	IR64_boundary_990
nipponbare_boundary_1072;nipponbare_boundary_1073	IR64_boundary_1045;IR64_boundary_1046
nipponbare_boundary_1656	IR64_boundary_1582;IR64_boundary_1583;IR64_boundary_1584
nipponbare_boundary_677;nipponbare_boundary_678	IR64_boundary_686;IR64_boundary_687
nipponbare_boundary_1288	
	IR64_boundary_1258
	IR64_boundary_1357
nipponbare_boundary_1464	
nipponbare_boundary_1502	IR64_boundary_1411
nipponbare_boundary_1543	
	IR64_boundary_1467
nipponbare_boundary_173	
nipponbare_boundary_1605	
nipponbare_boundary_1606	
nipponbare_boundary_1695	
	IR64_boundary_1651
nipponbare_boundary_1802	
	IR64_boundary_225
nipponbare_boundary_263	
nipponbare_boundary_260	IR64_boundary_262
nipponbare_boundary_264	
nipponbare_boundary_305	
nipponbare_boundary_320	
	IR64_boundary_390
nipponbare_boundary_407	
	IR64_boundary_407
	IR64_boundary_419
nipponbare_boundary_425	
nipponbare_boundary_480	IR64_boundary_477
	IR64_boundary_488
nipponbare_boundary_509	
	IR64_boundary_505
nipponbare_boundary_559	
nipponbare_boundary_560	
nipponbare_boundary_590	
nipponbare_boundary_604	
nipponbare_boundary_49	
	IR64_boundary_683
nipponbare_boundary_690	
nipponbare_boundary_691	IR64_boundary_701
nipponbare_boundary_694	
nipponbare_boundary_754	
	IR64_boundary_90
	IR64_boundary_871
nipponbare_boundary_910	
nipponbare_boundary_942	
nipponbare_boundary_944	
nipponbare_boundary_959	
	IR64_boundary_947
	IR64_boundary_980
nipponbare_boundary_999	IR64_boundary_964
	IR64_boundary_116
nipponbare_boundary_110	
	IR64_boundary_1017
nipponbare_boundary_1081	
nipponbare_boundary_1146	
nipponbare_boundary_1272	
	IR64_boundary_1308
	IR64_boundary_1365
nipponbare_boundary_163	
	IR64_boundary_1421
	IR64_boundary_1522
	IR64_boundary_661
nipponbare_boundary_1714	IR64_boundary_1644
	IR64_boundary_272
nipponbare_boundary_367	
	IR64_boundary_361
nipponbare_boundary_418	
nipponbare_boundary_512	
	IR64_boundary_582
	IR64_boundary_136
nipponbare_boundary_707	
nipponbare_boundary_948	
nipponbare_boundary_1058	
nipponbare_boundary_1056	
nipponbare_boundary_1179	
nipponbare_boundary_1224	IR64_boundary_1177
nipponbare_boundary_1236	
nipponbare_boundary_1238	IR64_boundary_1181
nipponbare_boundary_1243	IR64_boundary_1187
nipponbare_boundary_1251	IR64_boundary_1196
nipponbare_boundary_1294	
nipponbare_boundary_1586	
nipponbare_boundary_1590	
nipponbare_boundary_1669;nipponbare_boundary_1670	
nipponbare_boundary_189	IR64_boundary_203
nipponbare_boundary_197	IR64_boundary_207
nipponbare_boundary_314	IR64_boundary_322
nipponbare_boundary_315	

nipponbare_boundary_28	
nipponbare_boundary_712	IR64_boundary_717
nipponbare_boundary_390	IR64_boundary_379
nipponbare_boundary_405	IR64_boundary_403
nipponbare_boundary_432	
nipponbare_boundary_36	IR64_boundary_37
nipponbare_boundary_491	IR64_boundary_486
nipponbare_boundary_588;nipponbare_boundary_589	
nipponbare_boundary_596	IR64_boundary_606
nipponbare_boundary_611	
nipponbare_boundary_623	IR64_boundary_625
nipponbare_boundary_751	IR64_boundary_750
nipponbare_boundary_774	
nipponbare_boundary_806	IR64_boundary_786
nipponbare_boundary_807	IR64_boundary_787
nipponbare_boundary_931	IR64_boundary_896
nipponbare_boundary_1061	IR64_boundary_1037
nipponbare_boundary_127	IR64_boundary_133
nipponbare_boundary_129	
nipponbare_boundary_1111	IR64_boundary_1075
nipponbare_boundary_130	IR64_boundary_135
nipponbare_boundary_1170	IR64_boundary_1127
nipponbare_boundary_1194;nipponbare_boundary_1196	
nipponbare_boundary_1200;nipponbare_boundary_1201	
nipponbare_boundary_1210	IR64_boundary_1163
nipponbare_boundary_1211	IR64_boundary_1164
	IR64_boundary_1166
	IR64_boundary_1248;IR64_boundary_1249
	IR64_boundary_1303
	IR64_boundary_1366
	IR64_boundary_1629
	IR64_boundary_1518
nipponbare_boundary_61	IR64_boundary_61
	IR64_boundary_1674
nipponbare_boundary_235	IR64_boundary_238
	IR64_boundary_255
nipponbare_boundary_346	IR64_boundary_338
	IR64_boundary_713
nipponbare_boundary_365	IR64_boundary_358
	IR64_boundary_391
	IR64_boundary_396
	IR64_boundary_35
	IR64_boundary_580
	IR64_boundary_733
	IR64_boundary_737
	IR64_boundary_899
	IR64_boundary_1007
	IR64_boundary_1056
	IR64_boundary_1383
	IR64_boundary_1165
nipponbare_boundary_1132	
nipponbare_boundary_1149	
	IR64_boundary_1115
nipponbare_boundary_108	
	IR64_boundary_1152
nipponbare_boundary_1202	
nipponbare_boundary_1235	
	IR64_boundary_1192
nipponbare_boundary_1311	
nipponbare_boundary_1312	
	IR64_boundary_1267
nipponbare_boundary_1339	
nipponbare_boundary_1343	
	IR64_boundary_131
nipponbare_boundary_1416	
nipponbare_boundary_1430	
nipponbare_boundary_1431	
	IR64_boundary_1354
nipponbare_boundary_1480	
	IR64_boundary_1434
nipponbare_boundary_148	
	IR64_boundary_1496
	IR64_boundary_1525
nipponbare_boundary_1599	
	IR64_boundary_1620
nipponbare_boundary_1749	
	IR64_boundary_1694
nipponbare_boundary_1798	
nipponbare_boundary_1801	
	IR64_boundary_197
nipponbare_boundary_211	
nipponbare_boundary_215	
nipponbare_boundary_227	
	IR64_boundary_228
	IR64_boundary_235
	IR64_boundary_270
nipponbare_boundary_270	
nipponbare_boundary_273	
	IR64_boundary_293
	IR64_boundary_305
	IR64_boundary_320
nipponbare_boundary_330	
	IR64_boundary_335
	IR64_boundary_375
	IR64_boundary_398
	IR64_boundary_399
	IR64_boundary_465
	IR64_boundary_489
	IR64_boundary_492
nipponbare_boundary_501	
	IR64_boundary_508
nipponbare_boundary_517	
nipponbare_boundary_519	
	IR64_boundary_515
	IR64_boundary_516
nipponbare_boundary_51	
nipponbare_boundary_551	
nipponbare_boundary_565	
	IR64_boundary_57
nipponbare_boundary_60	
	IR64_boundary_691
nipponbare_boundary_710	
	IR64_boundary_748
	IR64_boundary_777
	IR64_boundary_797
	IR64_boundary_798
	IR64_boundary_813
	IR64_boundary_827
nipponbare_boundary_852	
nipponbare_boundary_853	
nipponbare_boundary_856	
nipponbare_boundary_858	
nipponbare_boundary_861	
nipponbare_boundary_862	
nipponbare_boundary_889	IR64_boundary_852

nipponbare_boundary_86	
	IR64_boundary_903
	IR64_boundary_936
	IR64_boundary_955
nipponbare_boundary_1049	
nipponbare_boundary_1055	
nipponbare_boundary_1069	
nipponbare_boundary_102	IR64_boundary_114
nipponbare_boundary_103	
nipponbare_boundary_1109	
nipponbare_boundary_1119	IR64_boundary_1082
nipponbare_boundary_1140	
nipponbare_boundary_1156	IR64_boundary_1117
nipponbare_boundary_1157	IR64_boundary_1118
nipponbare_boundary_1259	IR64_boundary_1203
nipponbare_boundary_1263	IR64_boundary_1215
nipponbare_boundary_1267	IR64_boundary_1220
nipponbare_boundary_1274	IR64_boundary_1225
nipponbare_boundary_1296	IR64_boundary_1243
nipponbare_boundary_1394	IR64_boundary_1327
nipponbare_boundary_1393;nipponbare_boundary_1396	
nipponbare_boundary_1411	
nipponbare_boundary_1498	
nipponbare_boundary_1541	
nipponbare_boundary_1554;nipponbare_boundary_1555	
nipponbare_boundary_1563	IR64_boundary_1482
nipponbare_boundary_1565	IR64_boundary_1484
nipponbare_boundary_1701	IR64_boundary_1632
nipponbare_boundary_1611	
nipponbare_boundary_1625	IR64_boundary_1549
nipponbare_boundary_1649	IR64_boundary_1576
nipponbare_boundary_1700	IR64_boundary_1631
nipponbare_boundary_1726	IR64_boundary_1652
nipponbare_boundary_1733	IR64_boundary_1655
nipponbare_boundary_1738	IR64_boundary_1659
nipponbare_boundary_1752	IR64_boundary_1671
nipponbare_boundary_175	IR64_boundary_849
nipponbare_boundary_1773	
nipponbare_boundary_249	IR64_boundary_252
nipponbare_boundary_292	IR64_boundary_312
nipponbare_boundary_328	IR64_boundary_333
nipponbare_boundary_340	IR64_boundary_337
	IR64_boundary_357
nipponbare_boundary_364	IR64_boundary_356
nipponbare_boundary_404	IR64_boundary_394
nipponbare_boundary_408	IR64_boundary_405
nipponbare_boundary_470	IR64_boundary_469
nipponbare_boundary_474	
nipponbare_boundary_524	IR64_boundary_525
nipponbare_boundary_52	IR64_boundary_51
nipponbare_boundary_552	IR64_boundary_558
nipponbare_boundary_556	IR64_boundary_569
nipponbare_boundary_585	IR64_boundary_594
nipponbare_boundary_586	IR64_boundary_595
nipponbare_boundary_587	IR64_boundary_596
nipponbare_boundary_609	IR64_boundary_617
nipponbare_boundary_610	IR64_boundary_618
nipponbare_boundary_630	IR64_boundary_637
nipponbare_boundary_58	IR64_boundary_58
nipponbare_boundary_59	IR64_boundary_59
nipponbare_boundary_656;nipponbare_boundary_657	
nipponbare_boundary_668	
nipponbare_boundary_681	IR64_boundary_690
nipponbare_boundary_703	IR64_boundary_710
nipponbare_boundary_733	
nipponbare_boundary_752	IR64_boundary_753
nipponbare_boundary_759	IR64_boundary_760
nipponbare_boundary_76	IR64_boundary_80
nipponbare_boundary_777	IR64_boundary_773
nipponbare_boundary_824	IR64_boundary_799
nipponbare_boundary_835	IR64_boundary_817
nipponbare_boundary_842	IR64_boundary_823
nipponbare_boundary_869	IR64_boundary_839
nipponbare_boundary_946;nipponbare_boundary_947	
nipponbare_boundary_93;nipponbare_boundary_94	
nipponbare_boundary_983;nipponbare_boundary_984	
nipponbare_boundary_96	IR64_boundary_103
nipponbare_boundary_1084	IR64_boundary_1060
	IR64_boundary_1182
	IR64_boundary_1240
	IR64_boundary_1329;IR64_boundary_1330
nipponbare_boundary_1400	IR64_boundary_1332
	IR64_boundary_146
	IR64_boundary_1436
	IR64_boundary_1447
	IR64_boundary_1448
	IR64_boundary_165;IR64_boundary_166
	IR64_boundary_9
	IR64_boundary_315;IR64_boundary_316
	IR64_boundary_352
nipponbare_boundary_525	IR64_boundary_526
nipponbare_boundary_535	IR64_boundary_542
	IR64_boundary_539
	IR64_boundary_550
	IR64_boundary_5;IR64_boundary_6
nipponbare_boundary_566	IR64_boundary_575
nipponbare_boundary_567	IR64_boundary_576
nipponbare_boundary_644	IR64_boundary_643
	IR64_boundary_834
	IR64_boundary_92
	IR64_boundary_915;IR64_boundary_916
	IR64_boundary_933;IR64_boundary_934
	IR64_boundary_960
nipponbare_boundary_998	IR64_boundary_965
	IR64_boundary_109
	IR64_boundary_1030
nipponbare_boundary_1270	IR64_boundary_1223
nipponbare_boundary_1306	IR64_boundary_1252
nipponbare_boundary_1316	IR64_boundary_1256
nipponbare_boundary_1330	IR64_boundary_1276
nipponbare_boundary_1347	IR64_boundary_1284
nipponbare_boundary_1508	IR64_boundary_1420
nipponbare_boundary_1510	IR64_boundary_1426
nipponbare_boundary_1575	IR64_boundary_1498
nipponbare_boundary_639	IR64_boundary_1083
nipponbare_boundary_1716	IR64_boundary_1645
nipponbare_boundary_18;nipponbare_boundary_19	
nipponbare_boundary_1722;nipponbare_boundary_1723	
nipponbare_boundary_1750	IR64_boundary_1668
nipponbare_boundary_1753	IR64_boundary_1672
nipponbare_boundary_1768	IR64_boundary_1689
nipponbare_boundary_285	IR64_boundary_291
nipponbare_boundary_286	IR64_boundary_303
nipponbare_boundary_290	IR64_boundary_306
nipponbare_boundary_427	IR64_boundary_424
nipponbare_boundary_438	IR64_boundary_434
nipponbare_boundary_485	IR64_boundary_479
nipponbare_boundary_534	IR64_boundary_541

nipponbare_boundary_537	IR64_boundary_547
nipponbare_boundary_549	
nipponbare_boundary_674	IR64_boundary_678
nipponbare_boundary_687	IR64_boundary_693
nipponbare_boundary_763	IR64_boundary_766
nipponbare_boundary_70	IR64_boundary_73
nipponbare_boundary_72	IR64_boundary_77
nipponbare_boundary_923	IR64_boundary_887
nipponbare_boundary_85	IR64_boundary_97
nipponbare_boundary_1008	IR64_boundary_992
nipponbare_boundary_90	IR64_boundary_102
nipponbare_boundary_1082	IR64_boundary_1058
nipponbare_boundary_1166;nipponbare_boundary_1167	
nipponbare_boundary_1203	IR64_boundary_1155
nipponbare_boundary_1478	IR64_boundary_1399
nipponbare_boundary_1507	IR64_boundary_1419
	IR64_boundary_1430;IR64_boundary_1431
	IR64_boundary_1567
nipponbare_boundary_161	IR64_boundary_172
nipponbare_boundary_221	IR64_boundary_222
	IR64_boundary_280;IR64_boundary_281
	IR64_boundary_292
nipponbare_boundary_318	IR64_boundary_324
	IR64_boundary_401;IR64_boundary_402
	IR64_boundary_453
	IR64_boundary_543
	IR64_boundary_648
	IR64_boundary_721
nipponbare_boundary_746	IR64_boundary_743
	IR64_boundary_198;IR64_boundary_842
nipponbare_boundary_934	IR64_boundary_901
	IR64_boundary_94;IR64_boundary_95
	IR64_boundary_1442;IR64_boundary_970
	IR64_boundary_1099
nipponbare_boundary_1186	IR64_boundary_1148
nipponbare_boundary_1253;nipponbare_boundary_1254	IR64_boundary_1198
nipponbare_boundary_138;nipponbare_boundary_139	IR64_boundary_142
nipponbare_boundary_1391	IR64_boundary_1323;IR64_boundary_975
nipponbare_boundary_1511	IR64_boundary_1437;IR64_boundary_1658
nipponbare_boundary_1513	IR64_boundary_1094;IR64_boundary_1438
nipponbare_boundary_769;nipponbare_boundary_770	IR64_boundary_771
nipponbare_boundary_832	IR64_boundary_811;IR64_boundary_812
	IR64_boundary_1412
	IR64_boundary_1413
	IR64_boundary_790
nipponbare_boundary_1213	
nipponbare_boundary_136	
nipponbare_boundary_137	
nipponbare_boundary_1244	
nipponbare_boundary_1370	
nipponbare_boundary_1475	
nipponbare_boundary_1585	
nipponbare_boundary_1589	
nipponbare_boundary_182	
nipponbare_boundary_192	
nipponbare_boundary_198	
nipponbare_boundary_234	

nipponbare_boundary_251	
nipponbare_boundary_252	
nipponbare_boundary_265	
nipponbare_boundary_309	
nipponbare_boundary_332	
nipponbare_boundary_333	
nipponbare_boundary_338	
nipponbare_boundary_6	
nipponbare_boundary_424	
nipponbare_boundary_471	
nipponbare_boundary_473	
nipponbare_boundary_39	
nipponbare_boundary_492	IR64_boundary_487
nipponbare_boundary_40	
nipponbare_boundary_612	
nipponbare_boundary_621	
nipponbare_boundary_631	
nipponbare_boundary_633	
nipponbare_boundary_634	
nipponbare_boundary_697	
nipponbare_boundary_708	
nipponbare_boundary_749	
nipponbare_boundary_772	
nipponbare_boundary_775	
nipponbare_boundary_789	
nipponbare_boundary_816	
nipponbare_boundary_817	
nipponbare_boundary_82	
nipponbare_boundary_1359	
nipponbare_boundary_1360	
nipponbare_boundary_1361	
nipponbare_boundary_1002	
nipponbare_boundary_1033	
nipponbare_boundary_1048	
nipponbare_boundary_1107	
nipponbare_boundary_1112	IR64_boundary_1076
	IR64_boundary_7
	IR64_boundary_1167
nipponbare_boundary_1218	IR64_boundary_1174
	IR64_boundary_15
	IR64_boundary_1309
	IR64_boundary_164
	IR64_boundary_1313
	IR64_boundary_1336
	IR64_boundary_1361
	IR64_boundary_1461
	IR64_boundary_1536
	IR64_boundary_1585
	IR64_boundary_1663
	IR64_boundary_1693
	IR64_boundary_1696
	IR64_boundary_236
	IR64_boundary_24
	IR64_boundary_347
	IR64_boundary_348
	IR64_boundary_417
	IR64_boundary_418
	IR64_boundary_437
	IR64_boundary_470
	IR64_boundary_555
	IR64_boundary_608
	IR64_boundary_49
	IR64_boundary_741
	IR64_boundary_751
	IR64_boundary_757
	IR64_boundary_89
	IR64_boundary_794
	IR64_boundary_883
	IR64_boundary_1004
	IR64_boundary_1010
	IR64_boundary_117
	IR64_boundary_1028
	IR64_boundary_1054
	IR64_boundary_1382
	IR64_boundary_1093
	IR64_boundary_1119
	IR64_boundary_140
nipponbare_boundary_1131	
	IR64_boundary_119
	IR64_boundary_130
nipponbare_boundary_518	
nipponbare_boundary_522	
	IR64_boundary_518
nipponbare_boundary_871	

nipponbare_boundary_872	
	IR64_boundary_982
nipponbare_boundary_104	
nipponbare_boundary_1234	
nipponbare_boundary_1246	
nipponbare_boundary_1266	
nipponbare_boundary_1276	
nipponbare_boundary_1310	
nipponbare_boundary_1334	
nipponbare_boundary_1335	
nipponbare_boundary_1395	
nipponbare_boundary_1422	
nipponbare_boundary_1423	
nipponbare_boundary_1493	
nipponbare_boundary_1516	
nipponbare_boundary_1520	
nipponbare_boundary_146	
nipponbare_boundary_1542	
nipponbare_boundary_1552	
nipponbare_boundary_1696	
nipponbare_boundary_1628	
nipponbare_boundary_1639	
nipponbare_boundary_1643	
nipponbare_boundary_1657	
nipponbare_boundary_1710	IR64_boundary_1641
nipponbare_boundary_1740	
nipponbare_boundary_1789	
nipponbare_boundary_201	
nipponbare_boundary_316	
	IR64_boundary_326
nipponbare_boundary_329	
nipponbare_boundary_369	
nipponbare_boundary_379	
nipponbare_boundary_510	
nipponbare_boundary_528	
nipponbare_boundary_530	
nipponbare_boundary_568	
nipponbare_boundary_602	
nipponbare_boundary_616	
nipponbare_boundary_638	
nipponbare_boundary_645	
nipponbare_boundary_705	
nipponbare_boundary_720	
nipponbare_boundary_722	
nipponbare_boundary_740	
nipponbare_boundary_815	
nipponbare_boundary_857	
nipponbare_boundary_878	
nipponbare_boundary_909	
nipponbare_boundary_937	
nipponbare_boundary_965	
	IR64_boundary_942
nipponbare_boundary_95	
nipponbare_boundary_1050	
nipponbare_boundary_1070	
nipponbare_boundary_1071	
nipponbare_boundary_1098	
nipponbare_boundary_1099	
	IR64_boundary_1102
	IR64_boundary_1107
	IR64_boundary_1134
	IR64_boundary_1179
	IR64_boundary_1205
	IR64_boundary_1206
	IR64_boundary_1222
	IR64_boundary_1231
	IR64_boundary_1236
	IR64_boundary_1244
	IR64_boundary_1268
	IR64_boundary_1325
	IR64_boundary_1333
	IR64_boundary_1335
	IR64_boundary_1339
	IR64_boundary_1340
	IR64_boundary_1348
	IR64_boundary_1353
	IR64_boundary_1368
	IR64_boundary_1389
nipponbare_boundary_1509	IR64_boundary_1425
	IR64_boundary_1449
	IR64_boundary_1454
	IR64_boundary_1529
	IR64_boundary_1545
	IR64_boundary_1552
	IR64_boundary_1556
	IR64_boundary_1558
	IR64_boundary_1561
	IR64_boundary_1665
	IR64_boundary_1097
	IR64_boundary_1669
	IR64_boundary_1670
	IR64_boundary_1673
	IR64_boundary_1675
	IR64_boundary_1686
	IR64_boundary_1710
	IR64_boundary_1711
	IR64_boundary_202
	IR64_boundary_294
	IR64_boundary_301
	IR64_boundary_336
	IR64_boundary_374
	IR64_boundary_385
	IR64_boundary_432
	IR64_boundary_501
	IR64_boundary_502
	IR64_boundary_504
	IR64_boundary_510
	IR64_boundary_520
	IR64_boundary_531
	IR64_boundary_532
	IR64_boundary_559
	IR64_boundary_563
	IR64_boundary_564
	IR64_boundary_623
	IR64_boundary_629
	IR64_boundary_632
	IR64_boundary_633
	IR64_boundary_634
	IR64_boundary_635
	IR64_boundary_656
	IR64_boundary_685
	IR64_boundary_708
	IR64_boundary_769
	IR64_boundary_782
nipponbare_boundary_836	IR64_boundary_818

	IR64_boundary_872
	IR64_boundary_876
	IR64_boundary_925
	IR64_boundary_926
	IR64_boundary_976
	IR64_boundary_978
	IR64_boundary_998
	IR64_boundary_110
	IR64_boundary_1013
	IR64_boundary_1018
	IR64_boundary_1043
	IR64_boundary_1069
nipponbare_boundary_1219	
nipponbare_boundary_1230	
nipponbare_boundary_1271	
nipponbare_boundary_1282	
nipponbare_boundary_1283	
nipponbare_boundary_1284	
nipponbare_boundary_1292	
nipponbare_boundary_1299	
nipponbare_boundary_1307	
nipponbare_boundary_1327	
nipponbare_boundary_125	
nipponbare_boundary_1415	
nipponbare_boundary_1437	
nipponbare_boundary_1428	
nipponbare_boundary_1444	
nipponbare_boundary_1470	
nipponbare_boundary_1477	
nipponbare_boundary_1479	
nipponbare_boundary_1487	
nipponbare_boundary_1488	
nipponbare_boundary_1514	
nipponbare_boundary_1561	
nipponbare_boundary_1594	
nipponbare_boundary_1642	
nipponbare_boundary_1663	
nipponbare_boundary_1689	
nipponbare_boundary_1713	
nipponbare_boundary_1721	
nipponbare_boundary_1746	
nipponbare_boundary_1777	
nipponbare_boundary_202	
nipponbare_boundary_220	
nipponbare_boundary_223	
nipponbare_boundary_224	
nipponbare_boundary_240	
nipponbare_boundary_284	
nipponbare_boundary_288	
nipponbare_boundary_30	
nipponbare_boundary_331	
nipponbare_boundary_436	
nipponbare_boundary_439	
nipponbare_boundary_441	
nipponbare_boundary_468	
nipponbare_boundary_493	
nipponbare_boundary_1231	IR64_boundary_1178
nipponbare_boundary_523	
nipponbare_boundary_527	
nipponbare_boundary_548	
nipponbare_boundary_581	
nipponbare_boundary_667	
nipponbare_boundary_676	
nipponbare_boundary_679	
nipponbare_boundary_696	
nipponbare_boundary_735	
nipponbare_boundary_794	
nipponbare_boundary_810	
nipponbare_boundary_825	
nipponbare_boundary_830	
nipponbare_boundary_860	
nipponbare_boundary_863	
nipponbare_boundary_864	
nipponbare_boundary_865	
nipponbare_boundary_78	
nipponbare_boundary_885	
nipponbare_boundary_940	
nipponbare_boundary_955	
nipponbare_boundary_973	
nipponbare_boundary_1022	
nipponbare_boundary_1034	
nipponbare_boundary_91	
nipponbare_boundary_1100	
nipponbare_boundary_1124	
nipponbare_boundary_1163	
nipponbare_boundary_1168	
nipponbare_boundary_1187	
nipponbare_boundary_1188	
	IR64_boundary_1219
	IR64_boundary_1241
	IR64_boundary_1257
	IR64_boundary_1266
	IR64_boundary_1278
	IR64_boundary_1286
	IR64_boundary_1401
	IR64_boundary_1403
	IR64_boundary_1404
	IR64_boundary_1435
	IR64_boundary_1453
	IR64_boundary_1457
	IR64_boundary_1458
	IR64_boundary_1470
	IR64_boundary_1495
	IR64_boundary_1509
	IR64_boundary_1568
	IR64_boundary_1605
	IR64_boundary_1637
	IR64_boundary_538
	IR64_boundary_180
	IR64_boundary_304
	IR64_boundary_310
nipponbare_boundary_723	IR64_boundary_719
	IR64_boundary_32
	IR64_boundary_456
	IR64_boundary_513
	IR64_boundary_540
	IR64_boundary_544
	IR64_boundary_1096
	IR64_boundary_53
nipponbare_boundary_607	IR64_boundary_615
	IR64_boundary_688
	IR64_boundary_720
	IR64_boundary_739
	IR64_boundary_742
	IR64_boundary_758
	IR64_boundary_761
	IR64_boundary_795
	IR64_boundary_807
	IR64_boundary_943

	IR64_boundary_98
	IR64_boundary_991
nipponbare_boundary_269	IR64_boundary_268
	IR64_boundary_101
	IR64_boundary_108
	IR64_boundary_1085
nipponbare_boundary_655	IR64_boundary_1095
	IR64_boundary_1125
	IR64_boundary_1126
	IR64_boundary_1156
nipponbare_boundary_1005	IR64_boundary_989
nipponbare_boundary_1046	IR64_boundary_1025
nipponbare_boundary_1074	IR64_boundary_1047
nipponbare_boundary_1089	IR64_boundary_1065
nipponbare_boundary_1122	IR64_boundary_1089
nipponbare_boundary_1130	IR64_boundary_1092
nipponbare_boundary_1161	IR64_boundary_1124
nipponbare_boundary_1185	IR64_boundary_1147
nipponbare_boundary_1206	IR64_boundary_1159
nipponbare_boundary_1237	IR64_boundary_1180
nipponbare_boundary_1264	IR64_boundary_1216
nipponbare_boundary_1273	IR64_boundary_1224
nipponbare_boundary_1305	IR64_boundary_1251
nipponbare_boundary_1329	IR64_boundary_1275
nipponbare_boundary_1348	IR64_boundary_1285
nipponbare_boundary_1349	IR64_boundary_1287
nipponbare_boundary_1352	IR64_boundary_1289
nipponbare_boundary_1379	IR64_boundary_1316
nipponbare_boundary_1383	IR64_boundary_1319
nipponbare_boundary_1451	IR64_boundary_1375
nipponbare_boundary_1490	IR64_boundary_1405
nipponbare_boundary_1535	IR64_boundary_1464
nipponbare_boundary_1536	IR64_boundary_1465
nipponbare_boundary_1538	IR64_boundary_1466
nipponbare_boundary_1562	IR64_boundary_1481
nipponbare_boundary_1566	IR64_boundary_1485
nipponbare_boundary_1591	IR64_boundary_1513
nipponbare_boundary_1596	IR64_boundary_1537
nipponbare_boundary_1617	IR64_boundary_1542
nipponbare_boundary_1626	IR64_boundary_1550
nipponbare_boundary_1633	IR64_boundary_1565
nipponbare_boundary_167	IR64_boundary_179
nipponbare_boundary_1684	IR64_boundary_1610
nipponbare_boundary_1686	IR64_boundary_1612
nipponbare_boundary_1771	IR64_boundary_1691
nipponbare_boundary_1774	IR64_boundary_1695
nipponbare_boundary_1784	IR64_boundary_1697
nipponbare_boundary_1800	IR64_boundary_1702
nipponbare_boundary_195	IR64_boundary_206
nipponbare_boundary_222	IR64_boundary_223
nipponbare_boundary_3	IR64_boundary_2
nipponbare_boundary_35	IR64_boundary_36
nipponbare_boundary_37	IR64_boundary_39
nipponbare_boundary_384	IR64_boundary_370
nipponbare_boundary_389	IR64_boundary_377
nipponbare_boundary_440	IR64_boundary_436
nipponbare_boundary_460	IR64_boundary_455
nipponbare_boundary_466	IR64_boundary_460
nipponbare_boundary_545	IR64_boundary_554
nipponbare_boundary_584	IR64_boundary_593
nipponbare_boundary_598	IR64_boundary_610
nipponbare_boundary_642	IR64_boundary_640
nipponbare_boundary_653	IR64_boundary_649
nipponbare_boundary_661	IR64_boundary_660
nipponbare_boundary_666	IR64_boundary_667
nipponbare_boundary_702	IR64_boundary_709
nipponbare_boundary_704	IR64_boundary_711
nipponbare_boundary_74	IR64_boundary_78
nipponbare_boundary_741	IR64_boundary_731
nipponbare_boundary_742	IR64_boundary_734
nipponbare_boundary_747	IR64_boundary_744
nipponbare_boundary_748	IR64_boundary_746
nipponbare_boundary_753	IR64_boundary_754
nipponbare_boundary_81	IR64_boundary_84
nipponbare_boundary_827	IR64_boundary_800
nipponbare_boundary_831	IR64_boundary_810
nipponbare_boundary_850	IR64_boundary_829
nipponbare_boundary_851	IR64_boundary_830
nipponbare_boundary_859	IR64_boundary_835
nipponbare_boundary_870	IR64_boundary_840
nipponbare_boundary_935	IR64_boundary_902
nipponbare_boundary_957	IR64_boundary_920
nipponbare_boundary_962	IR64_boundary_927
nipponbare_boundary_963	IR64_boundary_928
nipponbare_boundary_986	IR64_boundary_958
