# Supplementary material for: Evolutionary and Functional Conservation of Topologically Associating Domain Boundaries in Rice Genomes
Source: Int J Mol Sci. 2026 Oct 10;27(20):8979. doi: 10.3390/ijms27208979 (PMC13704375; doi:10.3390/ijms27208979)
Supplement: Supplementary file 1 [file ijms-27-08979-s001.zip › Sup_Table_S2.pdf]

Supplemental Table S2: TAD boundary coordinates for all (5) Oryza species

Species	Boundary Name	Chr	Start	End
Azucena	azucena_boundary_0	azucena_chr01	82500	87500
Azucena	azucena_boundary_1	azucena_chr01	127500	132500
Azucena	azucena_boundary_2	azucena_chr01	192500	197500
Azucena	azucena_boundary_3	azucena_chr01	277500	282500
Azucena	azucena_boundary_4	azucena_chr01	622500	627500
Azucena	azucena_boundary_5	azucena_chr01	702500	707500
Azucena	azucena_boundary_6	azucena_chr01	1797500	1837500
Azucena	azucena_boundary_7	azucena_chr01	1922500	1927500
Azucena	azucena_boundary_8	azucena_chr01	2737500	2742500
Azucena	azucena_boundary_9	azucena_chr01	2802500	2807500
Azucena	azucena_boundary_10	azucena_chr01	2937500	2942500
Azucena	azucena_boundary_11	azucena_chr01	3552500	3557500
Azucena	azucena_boundary_12	azucena_chr01	3647500	3652500
Azucena	azucena_boundary_13	azucena_chr01	4227500	4272500
Azucena	azucena_boundary_14	azucena_chr01	4417500	4422500
Azucena	azucena_boundary_15	azucena_chr01	4507500	4512500
Azucena	azucena_boundary_16	azucena_chr01	4907500	4947500
Azucena	azucena_boundary_17	azucena_chr01	5032500	5037500
Azucena	azucena_boundary_18	azucena_chr01	5317500	5322500
Azucena	azucena_boundary_19	azucena_chr01	5392500	5397500
Azucena	azucena_boundary_20	azucena_chr01	5442500	5447500
Azucena	azucena_boundary_21	azucena_chr01	5502500	5507500
Azucena	azucena_boundary_22	azucena_chr01	5657500	5662500
Azucena	azucena_boundary_23	azucena_chr01	5727500	5732500
Azucena	azucena_boundary_24	azucena_chr01	5847500	5852500
Azucena	azucena_boundary_25	azucena_chr01	5907500	5912500
Azucena	azucena_boundary_26	azucena_chr01	5962500	5967500
Azucena	azucena_boundary_27	azucena_chr01	6657500	6662500
Azucena	azucena_boundary_28	azucena_chr01	6717500	6722500
Azucena	azucena_boundary_29	azucena_chr01	6857500	6862500

Azucena	azucena_boundary_30	azucena_chr01	7027500	7032500
Azucena	azucena_boundary_31	azucena_chr01	7102500	7107500
Azucena	azucena_boundary_32	azucena_chr01	7182500	7187500
Azucena	azucena_boundary_33	azucena_chr01	7387500	7432500
Azucena	azucena_boundary_34	azucena_chr01	7477500	7482500
Azucena	azucena_boundary_35	azucena_chr01	7527500	7532500
Azucena	azucena_boundary_36	azucena_chr01	7692500	7697500
Azucena	azucena_boundary_37	azucena_chr01	7787500	7792500
Azucena	azucena_boundary_38	azucena_chr01	9192500	9197500
Azucena	azucena_boundary_39	azucena_chr01	9252500	9287500
Azucena	azucena_boundary_40	azucena_chr01	9372500	9377500
Azucena	azucena_boundary_41	azucena_chr01	9432500	9437500
Azucena	azucena_boundary_42	azucena_chr01	9537500	9542500
Azucena	azucena_boundary_43	azucena_chr01	9592500	9597500
Azucena	azucena_boundary_44	azucena_chr01	10242500	10247500
Azucena	azucena_boundary_45	azucena_chr01	10292500	10297500
Azucena	azucena_boundary_46	azucena_chr01	10397500	10402500
Azucena	azucena_boundary_47	azucena_chr01	10472500	10477500
Azucena	azucena_boundary_48	azucena_chr01	10542500	10602500
Azucena	azucena_boundary_49	azucena_chr01	10712500	10717500
Azucena	azucena_boundary_50	azucena_chr01	10772500	10777500
Azucena	azucena_boundary_51	azucena_chr01	10837500	10842500
Azucena	azucena_boundary_52	azucena_chr01	11262500	11267500
Azucena	azucena_boundary_53	azucena_chr01	11317500	11322500
Azucena	azucena_boundary_54	azucena_chr01	11367500	11372500
Azucena	azucena_boundary_55	azucena_chr01	11472500	11512500
Azucena	azucena_boundary_56	azucena_chr01	11627500	11632500
Azucena	azucena_boundary_57	azucena_chr01	11702500	11707500
Azucena	azucena_boundary_58	azucena_chr01	11902500	11907500
Azucena	azucena_boundary_59	azucena_chr01	11972500	11977500
Azucena	azucena_boundary_60	azucena_chr01	12032500	12037500
Azucena	azucena_boundary_61	azucena_chr01	12097500	12102500

Azucena	azucena_boundary_62	azucena_chr01	12687500	12692500
Azucena	azucena_boundary_63	azucena_chr01	12867500	12872500
Azucena	azucena_boundary_64	azucena_chr01	13462500	13467500
Azucena	azucena_boundary_65	azucena_chr01	13512500	13517500
Azucena	azucena_boundary_66	azucena_chr01	13862500	13867500
Azucena	azucena_boundary_67	azucena_chr01	13927500	13932500
Azucena	azucena_boundary_68	azucena_chr01	15192500	15197500
Azucena	azucena_boundary_69	azucena_chr01	15252500	15257500
Azucena	azucena_boundary_70	azucena_chr01	15867500	15872500
Azucena	azucena_boundary_71	azucena_chr01	15987500	15992500
Azucena	azucena_boundary_72	azucena_chr01	16062500	16067500
Azucena	azucena_boundary_73	azucena_chr01	16457500	16462500
Azucena	azucena_boundary_74	azucena_chr01	16537500	16542500
Azucena	azucena_boundary_75	azucena_chr01	18682500	18722500
Azucena	azucena_boundary_76	azucena_chr01	19192500	19197500
Azucena	azucena_boundary_77	azucena_chr01	19262500	19267500
Azucena	azucena_boundary_78	azucena_chr01	19727500	19732500
Azucena	azucena_boundary_79	azucena_chr01	19827500	19832500
Azucena	azucena_boundary_80	azucena_chr01	19962500	19967500
Azucena	azucena_boundary_81	azucena_chr01	20037500	20042500
Azucena	azucena_boundary_82	azucena_chr01	21777500	21822500
Azucena	azucena_boundary_83	azucena_chr01	21912500	21917500
Azucena	azucena_boundary_84	azucena_chr01	22177500	22182500
Azucena	azucena_boundary_85	azucena_chr01	22252500	22257500
Azucena	azucena_boundary_86	azucena_chr01	22792500	22797500
Azucena	azucena_boundary_87	azucena_chr01	22882500	22887500
Azucena	azucena_boundary_88	azucena_chr01	22957500	22962500
Azucena	azucena_boundary_89	azucena_chr01	23012500	23017500
Azucena	azucena_boundary_90	azucena_chr01	23072500	23107500
Azucena	azucena_boundary_91	azucena_chr01	23627500	23632500
Azucena	azucena_boundary_92	azucena_chr01	23722500	23727500
Azucena	azucena_boundary_93	azucena_chr01	23792500	23797500

Azucena	azucena_boundary_94	azucena_chr01	23982500	23987500
Azucena	azucena_boundary_95	azucena_chr01	24032500	24037500
Azucena	azucena_boundary_96	azucena_chr01	24127500	24132500
Azucena	azucena_boundary_97	azucena_chr01	24207500	24212500
Azucena	azucena_boundary_98	azucena_chr01	24272500	24277500
Azucena	azucena_boundary_99	azucena_chr01	24552500	24557500
Azucena	azucena_boundary_100	azucena_chr01	24632500	24712500
Azucena	azucena_boundary_101	azucena_chr01	24752500	24757500
Azucena	azucena_boundary_102	azucena_chr01	24857500	24862500
Azucena	azucena_boundary_103	azucena_chr01	24927500	24932500
Azucena	azucena_boundary_104	azucena_chr01	25037500	25042500
Azucena	azucena_boundary_105	azucena_chr01	25122500	25167500
Azucena	azucena_boundary_106	azucena_chr01	25252500	25257500
Azucena	azucena_boundary_107	azucena_chr01	25297500	25302500
Azucena	azucena_boundary_108	azucena_chr01	25372500	25377500
Azucena	azucena_boundary_109	azucena_chr01	25742500	25747500
Azucena	azucena_boundary_110	azucena_chr01	25792500	25857500
Azucena	azucena_boundary_111	azucena_chr01	25907500	25912500
Azucena	azucena_boundary_112	azucena_chr01	25972500	25977500
Azucena	azucena_boundary_113	azucena_chr01	26137500	26142500
Azucena	azucena_boundary_114	azucena_chr01	26222500	26227500
Azucena	azucena_boundary_115	azucena_chr01	26295000	26300000
Azucena	azucena_boundary_116	azucena_chr01	26350000	26355000
Azucena	azucena_boundary_117	azucena_chr01	26662500	26697500
Azucena	azucena_boundary_118	azucena_chr01	26787500	26792500
Azucena	azucena_boundary_119	azucena_chr01	26917500	26922500
Azucena	azucena_boundary_120	azucena_chr01	27057500	27062500
Azucena	azucena_boundary_121	azucena_chr01	27137500	27142500
Azucena	azucena_boundary_122	azucena_chr01	27797500	27842500
Azucena	azucena_boundary_123	azucena_chr01	27892500	27897500
Azucena	azucena_boundary_124	azucena_chr01	28272500	28337500
Azucena	azucena_boundary_125	azucena_chr01	28402500	28407500

Azucena	azucena_boundary_126	azucena_chr01	29012500	29017500
Azucena	azucena_boundary_127	azucena_chr01	29057500	29172500
Azucena	azucena_boundary_128	azucena_chr01	29297500	29302500
Azucena	azucena_boundary_129	azucena_chr01	29347500	29352500
Azucena	azucena_boundary_130	azucena_chr01	29447500	29452500
Azucena	azucena_boundary_131	azucena_chr01	29492500	29540000
Azucena	azucena_boundary_132	azucena_chr01	29587500	29592500
Azucena	azucena_boundary_133	azucena_chr01	30197500	30227500
Azucena	azucena_boundary_134	azucena_chr01	30647500	30652500
Azucena	azucena_boundary_135	azucena_chr01	30702500	30707500
Azucena	azucena_boundary_136	azucena_chr01	30892500	30897500
Azucena	azucena_boundary_137	azucena_chr01	30952500	30957500
Azucena	azucena_boundary_138	azucena_chr01	31037500	31042500
Azucena	azucena_boundary_139	azucena_chr01	31747500	31752500
Azucena	azucena_boundary_140	azucena_chr01	31875000	31880000
Azucena	azucena_boundary_141	azucena_chr01	31932500	31937500
Azucena	azucena_boundary_142	azucena_chr01	32007500	32052500
Azucena	azucena_boundary_143	azucena_chr01	32817500	32822500
Azucena	azucena_boundary_144	azucena_chr01	32892500	32927500
Azucena	azucena_boundary_145	azucena_chr01	33067500	33072500
Azucena	azucena_boundary_146	azucena_chr01	33192500	33267500
Azucena	azucena_boundary_147	azucena_chr01	33332500	33337500
Azucena	azucena_boundary_148	azucena_chr01	33387500	33392500
Azucena	azucena_boundary_149	azucena_chr01	35917500	35922500
Azucena	azucena_boundary_150	azucena_chr01	35977500	35982500
Azucena	azucena_boundary_151	azucena_chr01	36072500	36077500
Azucena	azucena_boundary_152	azucena_chr01	36312500	36317500
Azucena	azucena_boundary_153	azucena_chr01	36392500	36427500
Azucena	azucena_boundary_154	azucena_chr01	36537500	36542500
Azucena	azucena_boundary_155	azucena_chr01	36582500	36587500
Azucena	azucena_boundary_156	azucena_chr01	36667500	36672500
Azucena	azucena_boundary_157	azucena_chr01	36797500	36802500

Azucena	azucena_boundary_158	azucena_chr01	36847500	36852500
Azucena	azucena_boundary_159	azucena_chr01	36897500	36902500
Azucena	azucena_boundary_160	azucena_chr01	37352500	37357500
Azucena	azucena_boundary_161	azucena_chr01	37432500	37472500
Azucena	azucena_boundary_162	azucena_chr01	37937500	37982500
Azucena	azucena_boundary_163	azucena_chr01	38022500	38027500
Azucena	azucena_boundary_164	azucena_chr01	38072500	38077500
Azucena	azucena_boundary_165	azucena_chr01	38177500	38182500
Azucena	azucena_boundary_166	azucena_chr01	38612500	38617500
Azucena	azucena_boundary_167	azucena_chr01	38697500	38702500
Azucena	azucena_boundary_168	azucena_chr01	38772500	38777500
Azucena	azucena_boundary_169	azucena_chr01	38837500	38842500
Azucena	azucena_boundary_170	azucena_chr01	38882500	38887500
Azucena	azucena_boundary_171	azucena_chr01	38962500	38967500
Azucena	azucena_boundary_172	azucena_chr01	39142500	39187500
Azucena	azucena_boundary_173	azucena_chr01	40075000	40122500
Azucena	azucena_boundary_174	azucena_chr01	40172500	40217500
Azucena	azucena_boundary_175	azucena_chr01	40257500	40262500
Azucena	azucena_boundary_176	azucena_chr01	40467500	40502500
Azucena	azucena_boundary_177	azucena_chr01	40622500	40687500
Azucena	azucena_boundary_178	azucena_chr01	40737500	40742500
Azucena	azucena_boundary_179	azucena_chr01	40822500	40867500
Azucena	azucena_boundary_180	azucena_chr01	40962500	40967500
Azucena	azucena_boundary_181	azucena_chr01	41012500	41017500
Azucena	azucena_boundary_182	azucena_chr01	41057500	41062500
Azucena	azucena_boundary_183	azucena_chr01	41122500	41127500
Azucena	azucena_boundary_184	azucena_chr01	41192500	41197500
Azucena	azucena_boundary_185	azucena_chr01	41247500	41252500
Azucena	azucena_boundary_186	azucena_chr01	41372500	41412500
Azucena	azucena_boundary_187	azucena_chr01	41527500	41532500
Azucena	azucena_boundary_188	azucena_chr01	41627500	41632500
Azucena	azucena_boundary_189	azucena_chr01	41702500	41707500

Azucena	azucena_boundary_190	azucena_chr01	41757500	41762500
Azucena	azucena_boundary_191	azucena_chr01	41842500	41847500
Azucena	azucena_boundary_192	azucena_chr01	42457500	42462500
Azucena	azucena_boundary_193	azucena_chr01	42517500	42522500
Azucena	azucena_boundary_194	azucena_chr01	43257500	43262500
Azucena	azucena_boundary_195	azucena_chr01	43322500	43327500
Azucena	azucena_boundary_196	azucena_chr01	43427500	43432500
Azucena	azucena_boundary_197	azucena_chr01	43472500	43477500
Azucena	azucena_boundary_198	azucena_chr01	43567500	43572500
Azucena	azucena_boundary_199	azucena_chr01	43662500	43742500
Azucena	azucena_boundary_200	azucena_chr02	187500	192500
Azucena	azucena_boundary_201	azucena_chr02	302500	307500
Azucena	azucena_boundary_202	azucena_chr02	417500	422500
Azucena	azucena_boundary_203	azucena_chr02	567500	572500
Azucena	azucena_boundary_204	azucena_chr02	647500	652500
Azucena	azucena_boundary_205	azucena_chr02	742500	747500
Azucena	azucena_boundary_206	azucena_chr02	1492500	1497500
Azucena	azucena_boundary_207	azucena_chr02	1552500	1557500
Azucena	azucena_boundary_208	azucena_chr02	1617500	1622500
Azucena	azucena_boundary_209	azucena_chr02	3402500	3407500
Azucena	azucena_boundary_210	azucena_chr02	3522500	3527500
Azucena	azucena_boundary_211	azucena_chr02	3612500	3617500
Azucena	azucena_boundary_212	azucena_chr02	3677500	3682500
Azucena	azucena_boundary_213	azucena_chr02	3787500	3792500
Azucena	azucena_boundary_214	azucena_chr02	3917500	3922500
Azucena	azucena_boundary_215	azucena_chr02	3997500	4077500
Azucena	azucena_boundary_216	azucena_chr02	4172500	4177500
Azucena	azucena_boundary_217	azucena_chr02	4217500	4222500
Azucena	azucena_boundary_218	azucena_chr02	5052500	5057500
Azucena	azucena_boundary_219	azucena_chr02	5112500	5117500
Azucena	azucena_boundary_220	azucena_chr02	5317500	5322500
Azucena	azucena_boundary_221	azucena_chr02	5397500	5402500

Azucena	azucena_boundary_222	azucena_chr02	5727500	5762500
Azucena	azucena_boundary_223	azucena_chr02	5842500	5847500
Azucena	azucena_boundary_224	azucena_chr02	5927500	5932500
Azucena	azucena_boundary_225	azucena_chr02	5997500	6002500
Azucena	azucena_boundary_226	azucena_chr02	6107500	6112500
Azucena	azucena_boundary_227	azucena_chr02	6592500	6597500
Azucena	azucena_boundary_228	azucena_chr02	6642500	6647500
Azucena	azucena_boundary_229	azucena_chr02	7252500	7257500
Azucena	azucena_boundary_230	azucena_chr02	7302500	7307500
Azucena	azucena_boundary_231	azucena_chr02	7657500	7717500
Azucena	azucena_boundary_232	azucena_chr02	7767500	7772500
Azucena	azucena_boundary_233	azucena_chr02	7867500	7872500
Azucena	azucena_boundary_234	azucena_chr02	8217500	8262500
Azucena	azucena_boundary_235	azucena_chr02	8312500	8347500
Azucena	azucena_boundary_236	azucena_chr02	8827500	8832500
Azucena	azucena_boundary_237	azucena_chr02	8882500	8887500
Azucena	azucena_boundary_238	azucena_chr02	8932500	8937500
Azucena	azucena_boundary_239	azucena_chr02	9032500	9077500
Azucena	azucena_boundary_240	azucena_chr02	10052500	10057500
Azucena	azucena_boundary_241	azucena_chr02	10142500	10182500
Azucena	azucena_boundary_242	azucena_chr02	10247500	10252500
Azucena	azucena_boundary_243	azucena_chr02	10307500	10312500
Azucena	azucena_boundary_244	azucena_chr02	10427500	10432500
Azucena	azucena_boundary_245	azucena_chr02	10472500	10477500
Azucena	azucena_boundary_246	azucena_chr02	11967500	11972500
Azucena	azucena_boundary_247	azucena_chr02	12177500	12182500
Azucena	azucena_boundary_248	azucena_chr02	12297500	12302500
Azucena	azucena_boundary_249	azucena_chr02	12437500	12442500
Azucena	azucena_boundary_250	azucena_chr02	12737500	12742500
Azucena	azucena_boundary_251	azucena_chr02	12862500	12867500
Azucena	azucena_boundary_252	azucena_chr02	12957500	12962500
Azucena	azucena_boundary_253	azucena_chr02	13272500	13277500

Azucena	azucena_boundary_254	azucena_chr02	13407500	13412500
Azucena	azucena_boundary_255	azucena_chr02	13992500	13997500
Azucena	azucena_boundary_256	azucena_chr02	14112500	14147500
Azucena	azucena_boundary_257	azucena_chr02	15532500	15537500
Azucena	azucena_boundary_258	azucena_chr02	15627500	15632500
Azucena	azucena_boundary_259	azucena_chr02	15697500	15702500
Azucena	azucena_boundary_260	azucena_chr02	15757500	15762500
Azucena	azucena_boundary_261	azucena_chr02	15802500	15807500
Azucena	azucena_boundary_262	azucena_chr02	15942500	15947500
Azucena	azucena_boundary_263	azucena_chr02	16027500	16032500
Azucena	azucena_boundary_264	azucena_chr02	17552500	17557500
Azucena	azucena_boundary_265	azucena_chr02	17627500	17632500
Azucena	azucena_boundary_266	azucena_chr02	17672500	17677500
Azucena	azucena_boundary_267	azucena_chr02	17732500	17737500
Azucena	azucena_boundary_268	azucena_chr02	18852500	18857500
Azucena	azucena_boundary_269	azucena_chr02	18932500	18937500
Azucena	azucena_boundary_270	azucena_chr02	19022500	19027500
Azucena	azucena_boundary_271	azucena_chr02	19082500	19087500
Azucena	azucena_boundary_272	azucena_chr02	19147500	19152500
Azucena	azucena_boundary_273	azucena_chr02	19222500	19227500
Azucena	azucena_boundary_274	azucena_chr02	19302500	19307500
Azucena	azucena_boundary_275	azucena_chr02	19407500	19412500
Azucena	azucena_boundary_276	azucena_chr02	19467500	19512500
Azucena	azucena_boundary_277	azucena_chr02	19552500	19592500
Azucena	azucena_boundary_278	azucena_chr02	19632500	19677500
Azucena	azucena_boundary_279	azucena_chr02	19727500	19732500
Azucena	azucena_boundary_280	azucena_chr02	19852500	19857500
Azucena	azucena_boundary_281	azucena_chr02	19962500	20037500
Azucena	azucena_boundary_282	azucena_chr02	20077500	20082500
Azucena	azucena_boundary_283	azucena_chr02	20162500	20167500
Azucena	azucena_boundary_284	azucena_chr02	20977500	20982500
Azucena	azucena_boundary_285	azucena_chr02	21037500	21042500

Azucena	azucena_boundary_286	azucena_chr02	21092500	21097500
Azucena	azucena_boundary_287	azucena_chr02	21197500	21237500
Azucena	azucena_boundary_288	azucena_chr02	21482500	21487500
Azucena	azucena_boundary_289	azucena_chr02	21537500	21542500
Azucena	azucena_boundary_290	azucena_chr02	22612500	22617500
Azucena	azucena_boundary_291	azucena_chr02	22697500	22702500
Azucena	azucena_boundary_292	azucena_chr02	22777500	22782500
Azucena	azucena_boundary_293	azucena_chr02	23692500	23697500
Azucena	azucena_boundary_294	azucena_chr02	23742500	23747500
Azucena	azucena_boundary_295	azucena_chr02	23807500	23812500
Azucena	azucena_boundary_296	azucena_chr02	23872500	23877500
Azucena	azucena_boundary_297	azucena_chr02	24017500	24022500
Azucena	azucena_boundary_298	azucena_chr02	24072500	24077500
Azucena	azucena_boundary_299	azucena_chr02	24147500	24177500
Azucena	azucena_boundary_300	azucena_chr02	24347500	24352500
Azucena	azucena_boundary_301	azucena_chr02	24437500	24442500
Azucena	azucena_boundary_302	azucena_chr02	25122500	25167500
Azucena	azucena_boundary_303	azucena_chr02	25357500	25362500
Azucena	azucena_boundary_304	azucena_chr02	25512500	25517500
Azucena	azucena_boundary_305	azucena_chr02	25572500	25617500
Azucena	azucena_boundary_306	azucena_chr02	25872500	25877500
Azucena	azucena_boundary_307	azucena_chr02	25922500	25927500
Azucena	azucena_boundary_308	azucena_chr02	26717500	26757500
Azucena	azucena_boundary_309	azucena_chr02	26817500	26822500
Azucena	azucena_boundary_310	azucena_chr02	26867500	26872500
Azucena	azucena_boundary_311	azucena_chr02	27052500	27057500
Azucena	azucena_boundary_312	azucena_chr02	27242500	27247500
Azucena	azucena_boundary_313	azucena_chr02	27337500	27342500
Azucena	azucena_boundary_314	azucena_chr02	27397500	27402500
Azucena	azucena_boundary_315	azucena_chr02	27452500	27457500
Azucena	azucena_boundary_316	azucena_chr02	27502500	27507500
Azucena	azucena_boundary_317	azucena_chr02	27577500	27582500

Azucena	azucena_boundary_318	azucena_chr02	27857500	27862500
Azucena	azucena_boundary_319	azucena_chr02	28027500	28032500
Azucena	azucena_boundary_320	azucena_chr02	28072500	28077500
Azucena	azucena_boundary_321	azucena_chr02	28122500	28127500
Azucena	azucena_boundary_322	azucena_chr02	28212500	28242500
Azucena	azucena_boundary_323	azucena_chr02	28307500	28347500
Azucena	azucena_boundary_324	azucena_chr02	28387500	28392500
Azucena	azucena_boundary_325	azucena_chr02	28432500	28437500
Azucena	azucena_boundary_326	azucena_chr02	29022500	29027500
Azucena	azucena_boundary_327	azucena_chr02	29092500	29097500
Azucena	azucena_boundary_328	azucena_chr02	29142500	29177500
Azucena	azucena_boundary_329	azucena_chr02	29282500	29287500
Azucena	azucena_boundary_330	azucena_chr02	29367500	29372500
Azucena	azucena_boundary_331	azucena_chr02	29485000	29490000
Azucena	azucena_boundary_332	azucena_chr02	29537500	29542500
Azucena	azucena_boundary_333	azucena_chr02	29697500	29702500
Azucena	azucena_boundary_334	azucena_chr02	29777500	29782500
Azucena	azucena_boundary_335	azucena_chr02	30472500	30477500
Azucena	azucena_boundary_336	azucena_chr02	30542500	30547500
Azucena	azucena_boundary_337	azucena_chr02	30592500	30632500
Azucena	azucena_boundary_338	azucena_chr02	30737500	30742500
Azucena	azucena_boundary_339	azucena_chr02	30822500	30827500
Azucena	azucena_boundary_340	azucena_chr02	30907500	30912500
Azucena	azucena_boundary_341	azucena_chr02	31032500	31037500
Azucena	azucena_boundary_342	azucena_chr02	31132500	31137500
Azucena	azucena_boundary_343	azucena_chr02	31242500	31247500
Azucena	azucena_boundary_344	azucena_chr02	31402500	31407500
Azucena	azucena_boundary_345	azucena_chr02	31467500	31472500
Azucena	azucena_boundary_346	azucena_chr02	31537500	31562500
Azucena	azucena_boundary_347	azucena_chr02	31647500	31682500
Azucena	azucena_boundary_348	azucena_chr02	31762500	31767500
Azucena	azucena_boundary_349	azucena_chr02	31942500	31947500

Azucena	azucena_boundary_350	azucena_chr02	32002500	32007500
Azucena	azucena_boundary_351	azucena_chr02	32082500	32087500
Azucena	azucena_boundary_352	azucena_chr02	32167500	32172500
Azucena	azucena_boundary_353	azucena_chr02	32492500	32497500
Azucena	azucena_boundary_354	azucena_chr02	32607500	32612500
Azucena	azucena_boundary_355	azucena_chr02	32682500	32687500
Azucena	azucena_boundary_356	azucena_chr02	33317500	33322500
Azucena	azucena_boundary_357	azucena_chr02	33382500	33387500
Azucena	azucena_boundary_358	azucena_chr02	33442500	33447500
Azucena	azucena_boundary_359	azucena_chr02	33487500	33492500
Azucena	azucena_boundary_360	azucena_chr02	33547500	33582500
Azucena	azucena_boundary_361	azucena_chr02	33652500	33657500
Azucena	azucena_boundary_362	azucena_chr02	33717500	33722500
Azucena	azucena_boundary_363	azucena_chr02	33922500	33927500
Azucena	azucena_boundary_364	azucena_chr02	33982500	33987500
Azucena	azucena_boundary_365	azucena_chr02	34047500	34052500
Azucena	azucena_boundary_366	azucena_chr02	34242500	34247500
Azucena	azucena_boundary_367	azucena_chr02	34342500	34347500
Azucena	azucena_boundary_368	azucena_chr02	34427500	34432500
Azucena	azucena_boundary_369	azucena_chr02	34492500	34497500
Azucena	azucena_boundary_370	azucena_chr02	34537500	34542500
Azucena	azucena_boundary_371	azucena_chr02	34742500	34777500
Azucena	azucena_boundary_372	azucena_chr02	35047500	35052500
Azucena	azucena_boundary_373	azucena_chr02	35112500	35117500
Azucena	azucena_boundary_374	azucena_chr02	35187500	35192500
Azucena	azucena_boundary_375	azucena_chr02	35292500	35297500
Azucena	azucena_boundary_376	azucena_chr02	35462500	35467500
Azucena	azucena_boundary_377	azucena_chr02	35687500	35732500
Azucena	azucena_boundary_378	azucena_chr02	35797500	35802500
Azucena	azucena_boundary_379	azucena_chr02	35907500	35912500
Azucena	azucena_boundary_380	azucena_chr02	35962500	35967500
Azucena	azucena_boundary_381	azucena_chr02	36047500	36052500

Azucena	azucena_boundary_382	azucena_chr03	602500	642500
Azucena	azucena_boundary_383	azucena_chr03	907500	912500
Azucena	azucena_boundary_384	azucena_chr03	967500	972500
Azucena	azucena_boundary_385	azucena_chr03	1092500	1097500
Azucena	azucena_boundary_386	azucena_chr03	1357500	1362500
Azucena	azucena_boundary_387	azucena_chr03	1407500	1412500
Azucena	azucena_boundary_388	azucena_chr03	1612500	1617500
Azucena	azucena_boundary_389	azucena_chr03	1702500	1707500
Azucena	azucena_boundary_390	azucena_chr03	2902500	2907500
Azucena	azucena_boundary_391	azucena_chr03	2962500	2967500
Azucena	azucena_boundary_392	azucena_chr03	3012500	3017500
Azucena	azucena_boundary_393	azucena_chr03	3147500	3152500
Azucena	azucena_boundary_394	azucena_chr03	3217500	3257500
Azucena	azucena_boundary_395	azucena_chr03	3347500	3382500
Azucena	azucena_boundary_396	azucena_chr03	3717500	3722500
Azucena	azucena_boundary_397	azucena_chr03	3807500	3812500
Azucena	azucena_boundary_398	azucena_chr03	4082500	4087500
Azucena	azucena_boundary_399	azucena_chr03	4147500	4152500
Azucena	azucena_boundary_400	azucena_chr03	4292500	4327500
Azucena	azucena_boundary_401	azucena_chr03	4397500	4402500
Azucena	azucena_boundary_402	azucena_chr03	4457500	4462500
Azucena	azucena_boundary_403	azucena_chr03	4512500	4517500
Azucena	azucena_boundary_404	azucena_chr03	4617500	4727500
Azucena	azucena_boundary_405	azucena_chr03	4797500	4837500
Azucena	azucena_boundary_406	azucena_chr03	5037500	5042500
Azucena	azucena_boundary_407	azucena_chr03	5112500	5117500
Azucena	azucena_boundary_408	azucena_chr03	5172500	5177500
Azucena	azucena_boundary_409	azucena_chr03	5217500	5222500
Azucena	azucena_boundary_410	azucena_chr03	5277500	5282500
Azucena	azucena_boundary_411	azucena_chr03	5767500	5772500
Azucena	azucena_boundary_412	azucena_chr03	5812500	5817500
Azucena	azucena_boundary_413	azucena_chr03	6617500	6622500

Azucena	azucena_boundary_414	azucena_chr03	6667500	6672500
Azucena	azucena_boundary_415	azucena_chr03	6737500	6742500
Azucena	azucena_boundary_416	azucena_chr03	6897500	6902500
Azucena	azucena_boundary_417	azucena_chr03	6952500	6957500
Azucena	azucena_boundary_418	azucena_chr03	7792500	7797500
Azucena	azucena_boundary_419	azucena_chr03	7842500	7847500
Azucena	azucena_boundary_420	azucena_chr03	8352500	8357500
Azucena	azucena_boundary_421	azucena_chr03	8397500	8402500
Azucena	azucena_boundary_422	azucena_chr03	8512500	8517500
Azucena	azucena_boundary_423	azucena_chr03	8592500	8597500
Azucena	azucena_boundary_424	azucena_chr03	8787500	8792500
Azucena	azucena_boundary_425	azucena_chr03	8837500	8842500
Azucena	azucena_boundary_426	azucena_chr03	8927500	8967500
Azucena	azucena_boundary_427	azucena_chr03	9502500	9507500
Azucena	azucena_boundary_428	azucena_chr03	9552500	9557500
Azucena	azucena_boundary_429	azucena_chr03	9687500	9692500
Azucena	azucena_boundary_430	azucena_chr03	9792500	9797500
Azucena	azucena_boundary_431	azucena_chr03	10122500	10127500
Azucena	azucena_boundary_432	azucena_chr03	10242500	10247500
Azucena	azucena_boundary_433	azucena_chr03	10752500	10797500
Azucena	azucena_boundary_434	azucena_chr03	11197500	11277500
Azucena	azucena_boundary_435	azucena_chr03	11322500	11327500
Azucena	azucena_boundary_436	azucena_chr03	11372500	11377500
Azucena	azucena_boundary_437	azucena_chr03	11417500	11422500
Azucena	azucena_boundary_438	azucena_chr03	11852500	11857500
Azucena	azucena_boundary_439	azucena_chr03	11922500	11927500
Azucena	azucena_boundary_440	azucena_chr03	12042500	12047500
Azucena	azucena_boundary_441	azucena_chr03	12102500	12107500
Azucena	azucena_boundary_442	azucena_chr03	12307500	12312500
Azucena	azucena_boundary_443	azucena_chr03	12352500	12357500
Azucena	azucena_boundary_444	azucena_chr03	12757500	12762500
Azucena	azucena_boundary_445	azucena_chr03	12807500	12812500

Azucena	azucena_boundary_446	azucena_chr03	12937500	12942500
Azucena	azucena_boundary_447	azucena_chr03	13017500	13022500
Azucena	azucena_boundary_448	azucena_chr03	13207500	13237500
Azucena	azucena_boundary_449	azucena_chr03	13362500	13367500
Azucena	azucena_boundary_450	azucena_chr03	13432500	13437500
Azucena	azucena_boundary_451	azucena_chr03	14187500	14192500
Azucena	azucena_boundary_452	azucena_chr03	14267500	14272500
Azucena	azucena_boundary_453	azucena_chr03	14332500	14337500
Azucena	azucena_boundary_454	azucena_chr03	14427500	14432500
Azucena	azucena_boundary_455	azucena_chr03	14512500	14517500
Azucena	azucena_boundary_456	azucena_chr03	14857500	14862500
Azucena	azucena_boundary_457	azucena_chr03	14907500	14947500
Azucena	azucena_boundary_458	azucena_chr03	15007500	15052500
Azucena	azucena_boundary_459	azucena_chr03	15172500	15177500
Azucena	azucena_boundary_460	azucena_chr03	15487500	15492500
Azucena	azucena_boundary_461	azucena_chr03	15602500	15607500
Azucena	azucena_boundary_462	azucena_chr03	15787500	15792500
Azucena	azucena_boundary_463	azucena_chr03	15852500	15857500
Azucena	azucena_boundary_464	azucena_chr03	15917500	15922500
Azucena	azucena_boundary_465	azucena_chr03	16122500	16127500
Azucena	azucena_boundary_466	azucena_chr03	16172500	16177500
Azucena	azucena_boundary_467	azucena_chr03	16687500	16732500
Azucena	azucena_boundary_468	azucena_chr03	16897500	16902500
Azucena	azucena_boundary_469	azucena_chr03	16957500	16997500
Azucena	azucena_boundary_470	azucena_chr03	17047500	17052500
Azucena	azucena_boundary_471	azucena_chr03	17387500	17392500
Azucena	azucena_boundary_472	azucena_chr03	17442500	17447500
Azucena	azucena_boundary_473	azucena_chr03	17487500	17492500
Azucena	azucena_boundary_474	azucena_chr03	17582500	17587500
Azucena	azucena_boundary_475	azucena_chr03	17637500	17642500
Azucena	azucena_boundary_476	azucena_chr03	17782500	17787500
Azucena	azucena_boundary_477	azucena_chr03	17830000	17835000

Azucena	azucena_boundary_478	azucena_chr03	18307500	18312500
Azucena	azucena_boundary_479	azucena_chr03	18372500	18377500
Azucena	azucena_boundary_480	azucena_chr03	18467500	18472500
Azucena	azucena_boundary_481	azucena_chr03	18527500	18532500
Azucena	azucena_boundary_482	azucena_chr03	18577500	18582500
Azucena	azucena_boundary_483	azucena_chr03	18882500	18922500
Azucena	azucena_boundary_484	azucena_chr03	19432500	19437500
Azucena	azucena_boundary_485	azucena_chr03	19492500	19537500
Azucena	azucena_boundary_486	azucena_chr03	20002500	20007500
Azucena	azucena_boundary_487	azucena_chr03	20207500	20212500
Azucena	azucena_boundary_488	azucena_chr03	21637500	21642500
Azucena	azucena_boundary_489	azucena_chr03	21727500	21732500
Azucena	azucena_boundary_490	azucena_chr03	21982500	22027500
Azucena	azucena_boundary_491	azucena_chr03	22187500	22192500
Azucena	azucena_boundary_492	azucena_chr03	22237500	22242500
Azucena	azucena_boundary_493	azucena_chr03	24107500	24112500
Azucena	azucena_boundary_494	azucena_chr03	24157500	24162500
Azucena	azucena_boundary_495	azucena_chr03	24227500	24232500
Azucena	azucena_boundary_496	azucena_chr03	24277500	24282500
Azucena	azucena_boundary_497	azucena_chr03	24362500	24367500
Azucena	azucena_boundary_498	azucena_chr03	24572500	24577500
Azucena	azucena_boundary_499	azucena_chr03	24677500	24682500
Azucena	azucena_boundary_500	azucena_chr03	25427500	25432500
Azucena	azucena_boundary_501	azucena_chr03	25472500	25477500
Azucena	azucena_boundary_502	azucena_chr03	25557500	25562500
Azucena	azucena_boundary_503	azucena_chr03	26162500	26207500
Azucena	azucena_boundary_504	azucena_chr03	26260000	26297500
Azucena	azucena_boundary_505	azucena_chr03	26387500	26392500
Azucena	azucena_boundary_506	azucena_chr03	26867500	26872500
Azucena	azucena_boundary_507	azucena_chr03	26952500	26957500
Azucena	azucena_boundary_508	azucena_chr03	27017500	27127500
Azucena	azucena_boundary_509	azucena_chr03	27187500	27192500

Azucena	azucena_boundary_510	azucena_chr03	27287500	27292500
Azucena	azucena_boundary_511	azucena_chr03	27345000	27350000
Azucena	azucena_boundary_512	azucena_chr03	27462500	27467500
Azucena	azucena_boundary_513	azucena_chr03	27537500	27542500
Azucena	azucena_boundary_514	azucena_chr03	27582500	27587500
Azucena	azucena_boundary_515	azucena_chr03	27707500	27712500
Azucena	azucena_boundary_516	azucena_chr03	27767500	27772500
Azucena	azucena_boundary_517	azucena_chr03	27817500	27822500
Azucena	azucena_boundary_518	azucena_chr03	27902500	27907500
Azucena	azucena_boundary_519	azucena_chr03	28082500	28087500
Azucena	azucena_boundary_520	azucena_chr03	28152500	28157500
Azucena	azucena_boundary_521	azucena_chr03	28235000	28240000
Azucena	azucena_boundary_522	azucena_chr03	28432500	28437500
Azucena	azucena_boundary_523	azucena_chr03	28502500	28507500
Azucena	azucena_boundary_524	azucena_chr03	28567500	28572500
Azucena	azucena_boundary_525	azucena_chr03	29217500	29222500
Azucena	azucena_boundary_526	azucena_chr03	29292500	29297500
Azucena	azucena_boundary_527	azucena_chr03	30142500	30177500
Azucena	azucena_boundary_528	azucena_chr03	30217500	30222500
Azucena	azucena_boundary_529	azucena_chr03	30292500	30297500
Azucena	azucena_boundary_530	azucena_chr03	30352500	30382500
Azucena	azucena_boundary_531	azucena_chr03	30547500	30582500
Azucena	azucena_boundary_532	azucena_chr03	30727500	30732500
Azucena	azucena_boundary_533	azucena_chr03	30817500	30862500
Azucena	azucena_boundary_534	azucena_chr03	30937500	30942500
Azucena	azucena_boundary_535	azucena_chr03	30982500	30987500
Azucena	azucena_boundary_536	azucena_chr03	31262500	31267500
Azucena	azucena_boundary_537	azucena_chr03	31307500	31312500
Azucena	azucena_boundary_538	azucena_chr03	31507500	31572500
Azucena	azucena_boundary_539	azucena_chr03	31967500	31972500
Azucena	azucena_boundary_540	azucena_chr03	32022500	32027500
Azucena	azucena_boundary_541	azucena_chr03	32117500	32122500

Azucena	azucena_boundary_542	azucena_chr03	32187500	32232500
Azucena	azucena_boundary_543	azucena_chr03	32752500	32757500
Azucena	azucena_boundary_544	azucena_chr03	32807500	32812500
Azucena	azucena_boundary_545	azucena_chr03	32892500	32897500
Azucena	azucena_boundary_546	azucena_chr03	33422500	33427500
Azucena	azucena_boundary_547	azucena_chr03	33472500	33477500
Azucena	azucena_boundary_548	azucena_chr03	33647500	33692500
Azucena	azucena_boundary_549	azucena_chr03	33872500	33877500
Azucena	azucena_boundary_550	azucena_chr03	33952500	33957500
Azucena	azucena_boundary_551	azucena_chr03	34112500	34117500
Azucena	azucena_boundary_552	azucena_chr03	34162500	34167500
Azucena	azucena_boundary_553	azucena_chr03	34252500	34257500
Azucena	azucena_boundary_554	azucena_chr03	34737500	34742500
Azucena	azucena_boundary_555	azucena_chr03	34822500	34827500
Azucena	azucena_boundary_556	azucena_chr03	35182500	35187500
Azucena	azucena_boundary_557	azucena_chr03	35277500	35282500
Azucena	azucena_boundary_558	azucena_chr03	35462500	35467500
Azucena	azucena_boundary_559	azucena_chr03	35507500	35512500
Azucena	azucena_boundary_560	azucena_chr03	35807500	35847500
Azucena	azucena_boundary_561	azucena_chr03	35917500	35922500
Azucena	azucena_boundary_562	azucena_chr03	36007500	36012500
Azucena	azucena_boundary_563	azucena_chr03	36397500	36402500
Azucena	azucena_boundary_564	azucena_chr03	36457500	36462500
Azucena	azucena_boundary_565	azucena_chr03	36512500	36517500
Azucena	azucena_boundary_566	azucena_chr03	36597500	36602500
Azucena	azucena_boundary_567	azucena_chr03	36742500	36747500
Azucena	azucena_boundary_568	azucena_chr03	36787500	36792500
Azucena	azucena_boundary_569	azucena_chr03	36847500	36852500
Azucena	azucena_boundary_570	azucena_chr03	36957500	36997500
Azucena	azucena_boundary_571	azucena_chr03	37107500	37112500
Azucena	azucena_boundary_572	azucena_chr03	37167500	37172500
Azucena	azucena_boundary_573	azucena_chr03	37922500	37927500

Azucena	azucena_boundary_574	azucena_chr03	37972500	37977500
Azucena	azucena_boundary_575	azucena_chr04	287500	292500
Azucena	azucena_boundary_576	azucena_chr04	347500	352500
Azucena	azucena_boundary_577	azucena_chr04	1022500	1027500
Azucena	azucena_boundary_578	azucena_chr04	1092500	1132500
Azucena	azucena_boundary_579	azucena_chr04	1222500	1227500
Azucena	azucena_boundary_580	azucena_chr04	1267500	1272500
Azucena	azucena_boundary_581	azucena_chr04	1460000	1465000
Azucena	azucena_boundary_582	azucena_chr04	1577500	1582500
Azucena	azucena_boundary_583	azucena_chr04	4025000	4030000
Azucena	azucena_boundary_584	azucena_chr04	4107500	4212500
Azucena	azucena_boundary_585	azucena_chr04	4302500	4307500
Azucena	azucena_boundary_586	azucena_chr04	4360000	4365000
Azucena	azucena_boundary_587	azucena_chr04	4415000	4420000
Azucena	azucena_boundary_588	azucena_chr04	4557500	4562500
Azucena	azucena_boundary_589	azucena_chr04	4605000	4652500
Azucena	azucena_boundary_590	azucena_chr04	4957500	5002500
Azucena	azucena_boundary_591	azucena_chr04	5097500	5102500
Azucena	azucena_boundary_592	azucena_chr04	5167500	5172500
Azucena	azucena_boundary_593	azucena_chr04	5232500	5237500
Azucena	azucena_boundary_594	azucena_chr04	5362500	5367500
Azucena	azucena_boundary_595	azucena_chr04	5422500	5427500
Azucena	azucena_boundary_596	azucena_chr04	5477500	5482500
Azucena	azucena_boundary_597	azucena_chr04	7252500	7257500
Azucena	azucena_boundary_598	azucena_chr04	7365000	7370000
Azucena	azucena_boundary_599	azucena_chr04	7775000	7780000
Azucena	azucena_boundary_600	azucena_chr04	7845000	7850000
Azucena	azucena_boundary_601	azucena_chr04	7897500	7902500
Azucena	azucena_boundary_602	azucena_chr04	7962500	7967500
Azucena	azucena_boundary_603	azucena_chr04	8060000	8065000
Azucena	azucena_boundary_604	azucena_chr04	9437500	9462500
Azucena	azucena_boundary_605	azucena_chr04	9917500	9922500

Azucena	azucena_boundary_606	azucena_chr04	10017500	10022500
Azucena	azucena_boundary_607	azucena_chr04	10102500	10107500
Azucena	azucena_boundary_608	azucena_chr04	10202500	10207500
Azucena	azucena_boundary_609	azucena_chr04	10347500	10352500
Azucena	azucena_boundary_610	azucena_chr04	10462500	10467500
Azucena	azucena_boundary_611	azucena_chr04	10537500	10542500
Azucena	azucena_boundary_612	azucena_chr04	10737500	10772500
Azucena	azucena_boundary_613	azucena_chr04	11662500	11702500
Azucena	azucena_boundary_614	azucena_chr04	11967500	11972500
Azucena	azucena_boundary_615	azucena_chr04	12057500	12062500
Azucena	azucena_boundary_616	azucena_chr04	12107500	12112500
Azucena	azucena_boundary_617	azucena_chr04	14182500	14227500
Azucena	azucena_boundary_618	azucena_chr04	14367500	14372500
Azucena	azucena_boundary_619	azucena_chr04	14532500	14537500
Azucena	azucena_boundary_620	azucena_chr04	14670000	14675000
Azucena	azucena_boundary_621	azucena_chr04	14772500	14777500
Azucena	azucena_boundary_622	azucena_chr04	14822500	14827500
Azucena	azucena_boundary_623	azucena_chr04	14897500	14902500
Azucena	azucena_boundary_624	azucena_chr04	14947500	14952500
Azucena	azucena_boundary_625	azucena_chr04	15052500	15057500
Azucena	azucena_boundary_626	azucena_chr04	17105000	17110000
Azucena	azucena_boundary_627	azucena_chr04	17152500	17157500
Azucena	azucena_boundary_628	azucena_chr04	17307500	17312500
Azucena	azucena_boundary_629	azucena_chr04	17352500	17357500
Azucena	azucena_boundary_630	azucena_chr04	17412500	17417500
Azucena	azucena_boundary_631	azucena_chr04	17490000	17495000
Azucena	azucena_boundary_632	azucena_chr04	18042500	18047500
Azucena	azucena_boundary_633	azucena_chr04	18132500	18137500
Azucena	azucena_boundary_634	azucena_chr04	18232500	18277500
Azucena	azucena_boundary_635	azucena_chr04	19312500	19352500
Azucena	azucena_boundary_636	azucena_chr04	19822500	19827500
Azucena	azucena_boundary_637	azucena_chr04	19877500	19882500

Azucena	azucena_boundary_638	azucena_chr04	19937500	19942500
Azucena	azucena_boundary_639	azucena_chr04	20147500	20152500
Azucena	azucena_boundary_640	azucena_chr04	20202500	20207500
Azucena	azucena_boundary_641	azucena_chr04	20692500	20697500
Azucena	azucena_boundary_642	azucena_chr04	20787500	20792500
Azucena	azucena_boundary_643	azucena_chr04	21682500	21687500
Azucena	azucena_boundary_644	azucena_chr04	21727500	21732500
Azucena	azucena_boundary_645	azucena_chr04	21822500	21827500
Azucena	azucena_boundary_646	azucena_chr04	21892500	21937500
Azucena	azucena_boundary_647	azucena_chr04	22217500	22222500
Azucena	azucena_boundary_648	azucena_chr04	22302500	22307500
Azucena	azucena_boundary_649	azucena_chr04	22497500	22502500
Azucena	azucena_boundary_650	azucena_chr04	22542500	22547500
Azucena	azucena_boundary_651	azucena_chr04	22652500	22657500
Azucena	azucena_boundary_652	azucena_chr04	22712500	22717500
Azucena	azucena_boundary_653	azucena_chr04	22767500	22772500
Azucena	azucena_boundary_654	azucena_chr04	22822500	22827500
Azucena	azucena_boundary_655	azucena_chr04	23232500	23237500
Azucena	azucena_boundary_656	azucena_chr04	23342500	23347500
Azucena	azucena_boundary_657	azucena_chr04	23602500	23607500
Azucena	azucena_boundary_658	azucena_chr04	23657500	23662500
Azucena	azucena_boundary_659	azucena_chr04	23952500	23957500
Azucena	azucena_boundary_660	azucena_chr04	24107500	24112500
Azucena	azucena_boundary_661	azucena_chr04	24227500	24232500
Azucena	azucena_boundary_662	azucena_chr04	24302500	24307500
Azucena	azucena_boundary_663	azucena_chr04	24347500	24352500
Azucena	azucena_boundary_664	azucena_chr04	24427500	24432500
Azucena	azucena_boundary_665	azucena_chr04	24492500	24497500
Azucena	azucena_boundary_666	azucena_chr04	24537500	24542500
Azucena	azucena_boundary_667	azucena_chr04	24627500	24632500
Azucena	azucena_boundary_668	azucena_chr04	24697500	24702500
Azucena	azucena_boundary_669	azucena_chr04	24767500	24772500

Azucena	azucena_boundary_670	azucena_chr04	25102500	25107500
Azucena	azucena_boundary_671	azucena_chr04	25292500	25297500
Azucena	azucena_boundary_672	azucena_chr04	26652500	26657500
Azucena	azucena_boundary_673	azucena_chr04	26697500	26702500
Azucena	azucena_boundary_674	azucena_chr04	26752500	26757500
Azucena	azucena_boundary_675	azucena_chr04	26922500	26927500
Azucena	azucena_boundary_676	azucena_chr04	27002500	27007500
Azucena	azucena_boundary_677	azucena_chr04	27067500	27072500
Azucena	azucena_boundary_678	azucena_chr04	27142500	27147500
Azucena	azucena_boundary_679	azucena_chr04	27192500	27197500
Azucena	azucena_boundary_680	azucena_chr04	27292500	27297500
Azucena	azucena_boundary_681	azucena_chr04	27362500	27367500
Azucena	azucena_boundary_682	azucena_chr04	27732500	27737500
Azucena	azucena_boundary_683	azucena_chr04	27782500	27787500
Azucena	azucena_boundary_684	azucena_chr04	28242500	28247500
Azucena	azucena_boundary_685	azucena_chr04	28315000	28320000
Azucena	azucena_boundary_686	azucena_chr04	28397500	28402500
Azucena	azucena_boundary_687	azucena_chr04	28467500	28472500
Azucena	azucena_boundary_688	azucena_chr04	29042500	29047500
Azucena	azucena_boundary_689	azucena_chr04	29117500	29122500
Azucena	azucena_boundary_690	azucena_chr04	29187500	29222500
Azucena	azucena_boundary_691	azucena_chr04	29822500	29862500
Azucena	azucena_boundary_692	azucena_chr04	31612500	31617500
Azucena	azucena_boundary_693	azucena_chr04	31737500	31742500
Azucena	azucena_boundary_694	azucena_chr04	32270000	32275000
Azucena	azucena_boundary_695	azucena_chr04	32357500	32362500
Azucena	azucena_boundary_696	azucena_chr04	32507500	32512500
Azucena	azucena_boundary_697	azucena_chr04	32597500	32602500
Azucena	azucena_boundary_698	azucena_chr04	33322500	33327500
Azucena	azucena_boundary_699	azucena_chr04	33382500	33387500
Azucena	azucena_boundary_700	azucena_chr04	33522500	33527500
Azucena	azucena_boundary_701	azucena_chr04	33997500	34002500

Azucena	azucena_boundary_702	azucena_chr04	34052500	34057500
Azucena	azucena_boundary_703	azucena_chr05	92500	97500
Azucena	azucena_boundary_704	azucena_chr05	142500	147500
Azucena	azucena_boundary_705	azucena_chr05	662500	667500
Azucena	azucena_boundary_706	azucena_chr05	762500	767500
Azucena	azucena_boundary_707	azucena_chr05	867500	872500
Azucena	azucena_boundary_708	azucena_chr05	947500	952500
Azucena	azucena_boundary_709	azucena_chr05	2302500	2307500
Azucena	azucena_boundary_710	azucena_chr05	2427500	2472500
Azucena	azucena_boundary_711	azucena_chr05	2867500	2872500
Azucena	azucena_boundary_712	azucena_chr05	2937500	2942500
Azucena	azucena_boundary_713	azucena_chr05	3007500	3012500
Azucena	azucena_boundary_714	azucena_chr05	3107500	3112500
Azucena	azucena_boundary_715	azucena_chr05	3162500	3167500
Azucena	azucena_boundary_716	azucena_chr05	3217500	3222500
Azucena	azucena_boundary_717	azucena_chr05	3297500	3302500
Azucena	azucena_boundary_718	azucena_chr05	4877500	4882500
Azucena	azucena_boundary_719	azucena_chr05	4927500	4932500
Azucena	azucena_boundary_720	azucena_chr05	4992500	4997500
Azucena	azucena_boundary_721	azucena_chr05	5072500	5077500
Azucena	azucena_boundary_722	azucena_chr05	5167500	5172500
Azucena	azucena_boundary_723	azucena_chr05	5257500	5262500
Azucena	azucena_boundary_724	azucena_chr05	5732500	5777500
Azucena	azucena_boundary_725	azucena_chr05	6982500	6987500
Azucena	azucena_boundary_726	azucena_chr05	7027500	7032500
Azucena	azucena_boundary_727	azucena_chr05	7117500	7122500
Azucena	azucena_boundary_728	azucena_chr05	7177500	7182500
Azucena	azucena_boundary_729	azucena_chr05	8720000	8725000
Azucena	azucena_boundary_730	azucena_chr05	8887500	8892500
Azucena	azucena_boundary_731	azucena_chr05	8957500	9002500
Azucena	azucena_boundary_732	azucena_chr05	9817500	9822500
Azucena	azucena_boundary_733	azucena_chr05	9907500	9912500

Azucena	azucena_boundary_734	azucena_chr05	13072500	13077500
Azucena	azucena_boundary_735	azucena_chr05	13212500	13217500
Azucena	azucena_boundary_736	azucena_chr05	13752500	13757500
Azucena	azucena_boundary_737	azucena_chr05	13902500	13907500
Azucena	azucena_boundary_738	azucena_chr05	14017500	14022500
Azucena	azucena_boundary_739	azucena_chr05	14092500	14097500
Azucena	azucena_boundary_740	azucena_chr05	17157500	17162500
Azucena	azucena_boundary_741	azucena_chr05	17222500	17227500
Azucena	azucena_boundary_742	azucena_chr05	17337500	17342500
Azucena	azucena_boundary_743	azucena_chr05	17402500	17407500
Azucena	azucena_boundary_744	azucena_chr05	17487500	17527500
Azucena	azucena_boundary_745	azucena_chr05	17702500	17707500
Azucena	azucena_boundary_746	azucena_chr05	17752500	17757500
Azucena	azucena_boundary_747	azucena_chr05	17817500	17822500
Azucena	azucena_boundary_748	azucena_chr05	18377500	18382500
Azucena	azucena_boundary_749	azucena_chr05	18462500	18467500
Azucena	azucena_boundary_750	azucena_chr05	18812500	18817500
Azucena	azucena_boundary_751	azucena_chr05	18882500	18887500
Azucena	azucena_boundary_752	azucena_chr05	18932500	18937500
Azucena	azucena_boundary_753	azucena_chr05	19672500	19707500
Azucena	azucena_boundary_754	azucena_chr05	19772500	19777500
Azucena	azucena_boundary_755	azucena_chr05	20267500	20272500
Azucena	azucena_boundary_756	azucena_chr05	20377500	20382500
Azucena	azucena_boundary_757	azucena_chr05	20447500	20452500
Azucena	azucena_boundary_758	azucena_chr05	20562500	20567500
Azucena	azucena_boundary_759	azucena_chr05	20662500	20667500
Azucena	azucena_boundary_760	azucena_chr05	20797500	20802500
Azucena	azucena_boundary_761	azucena_chr05	20872500	20877500
Azucena	azucena_boundary_762	azucena_chr05	20947500	20952500
Azucena	azucena_boundary_763	azucena_chr05	21537500	21542500
Azucena	azucena_boundary_764	azucena_chr05	21582500	21587500
Azucena	azucena_boundary_765	azucena_chr05	21727500	21732500

Azucena	azucena_boundary_766	azucena_chr05	21787500	21792500
Azucena	azucena_boundary_767	azucena_chr05	21877500	21882500
Azucena	azucena_boundary_768	azucena_chr05	21932500	21937500
Azucena	azucena_boundary_769	azucena_chr05	22082500	22087500
Azucena	azucena_boundary_770	azucena_chr05	22142500	22147500
Azucena	azucena_boundary_771	azucena_chr05	22222500	22227500
Azucena	azucena_boundary_772	azucena_chr05	22297500	22302500
Azucena	azucena_boundary_773	azucena_chr05	22572500	22577500
Azucena	azucena_boundary_774	azucena_chr05	22632500	22637500
Azucena	azucena_boundary_775	azucena_chr05	22722500	22727500
Azucena	azucena_boundary_776	azucena_chr05	23407500	23412500
Azucena	azucena_boundary_777	azucena_chr05	23462500	23467500
Azucena	azucena_boundary_778	azucena_chr05	23652500	23657500
Azucena	azucena_boundary_779	azucena_chr05	23722500	23727500
Azucena	azucena_boundary_780	azucena_chr05	24002500	24007500
Azucena	azucena_boundary_781	azucena_chr05	24082500	24117500
Azucena	azucena_boundary_782	azucena_chr05	24207500	24212500
Azucena	azucena_boundary_783	azucena_chr05	24257500	24262500
Azucena	azucena_boundary_784	azucena_chr05	24327500	24332500
Azucena	azucena_boundary_785	azucena_chr05	24407500	24412500
Azucena	azucena_boundary_786	azucena_chr05	24532500	24537500
Azucena	azucena_boundary_787	azucena_chr05	24577500	24582500
Azucena	azucena_boundary_788	azucena_chr05	24647500	24652500
Azucena	azucena_boundary_789	azucena_chr05	24712500	24717500
Azucena	azucena_boundary_790	azucena_chr05	24767500	24772500
Azucena	azucena_boundary_791	azucena_chr05	25077500	25082500
Azucena	azucena_boundary_792	azucena_chr05	25127500	25132500
Azucena	azucena_boundary_793	azucena_chr05	25172500	25177500
Azucena	azucena_boundary_794	azucena_chr05	25232500	25237500
Azucena	azucena_boundary_795	azucena_chr05	25277500	25317500
Azucena	azucena_boundary_796	azucena_chr05	25612500	25617500
Azucena	azucena_boundary_797	azucena_chr05	25667500	25672500

Azucena	azucena_boundary_798	azucena_chr05	25712500	25717500
Azucena	azucena_boundary_799	azucena_chr05	25822500	25827500
Azucena	azucena_boundary_800	azucena_chr05	26192500	26197500
Azucena	azucena_boundary_801	azucena_chr05	26342500	26347500
Azucena	azucena_boundary_802	azucena_chr05	26422500	26427500
Azucena	azucena_boundary_803	azucena_chr05	26477500	26482500
Azucena	azucena_boundary_804	azucena_chr05	26857500	26862500
Azucena	azucena_boundary_805	azucena_chr05	26982500	26987500
Azucena	azucena_boundary_806	azucena_chr05	27087500	27092500
Azucena	azucena_boundary_807	azucena_chr05	27142500	27147500
Azucena	azucena_boundary_808	azucena_chr05	27227500	27232500
Azucena	azucena_boundary_809	azucena_chr05	27312500	27317500
Azucena	azucena_boundary_810	azucena_chr05	27412500	27417500
Azucena	azucena_boundary_811	azucena_chr05	27537500	27542500
Azucena	azucena_boundary_812	azucena_chr05	27602500	27607500
Azucena	azucena_boundary_813	azucena_chr05	27657500	27662500
Azucena	azucena_boundary_814	azucena_chr05	27747500	27752500
Azucena	azucena_boundary_815	azucena_chr05	27832500	27837500
Azucena	azucena_boundary_816	azucena_chr05	28207500	28242500
Azucena	azucena_boundary_817	azucena_chr05	28557500	28602500
Azucena	azucena_boundary_818	azucena_chr05	28867500	28872500
Azucena	azucena_boundary_819	azucena_chr05	28912500	28917500
Azucena	azucena_boundary_820	azucena_chr05	28967500	28972500
Azucena	azucena_boundary_821	azucena_chr05	29272500	29317500
Azucena	azucena_boundary_822	azucena_chr05	29402500	29407500
Azucena	azucena_boundary_823	azucena_chr05	29487500	29492500
Azucena	azucena_boundary_824	azucena_chr05	29547500	29552500
Azucena	azucena_boundary_825	azucena_chr05	29962500	30037500
Azucena	azucena_boundary_826	azucena_chr05	30090000	30095000
Azucena	azucena_boundary_827	azucena_chr05	30142500	30172500
Azucena	azucena_boundary_828	azucena_chr05	30217500	30222500
Azucena	azucena_boundary_829	azucena_chr05	30297500	30302500

Azucena	azucena_boundary_830	azucena_chr06	187500	192500
Azucena	azucena_boundary_831	azucena_chr06	267500	272500
Azucena	azucena_boundary_832	azucena_chr06	332500	337500
Azucena	azucena_boundary_833	azucena_chr06	467500	472500
Azucena	azucena_boundary_834	azucena_chr06	532500	537500
Azucena	azucena_boundary_835	azucena_chr06	682500	687500
Azucena	azucena_boundary_836	azucena_chr06	742500	747500
Azucena	azucena_boundary_837	azucena_chr06	867500	902500
Azucena	azucena_boundary_838	azucena_chr06	1027500	1032500
Azucena	azucena_boundary_839	azucena_chr06	1497500	1502500
Azucena	azucena_boundary_840	azucena_chr06	1612500	1617500
Azucena	azucena_boundary_841	azucena_chr06	2012500	2017500
Azucena	azucena_boundary_842	azucena_chr06	2097500	2102500
Azucena	azucena_boundary_843	azucena_chr06	2147500	2152500
Azucena	azucena_boundary_844	azucena_chr06	2252500	2257500
Azucena	azucena_boundary_845	azucena_chr06	2332500	2337500
Azucena	azucena_boundary_846	azucena_chr06	3252500	3282500
Azucena	azucena_boundary_847	azucena_chr06	3482500	3487500
Azucena	azucena_boundary_848	azucena_chr06	4067500	4072500
Azucena	azucena_boundary_849	azucena_chr06	4167500	4172500
Azucena	azucena_boundary_850	azucena_chr06	4277500	4282500
Azucena	azucena_boundary_851	azucena_chr06	4517500	4522500
Azucena	azucena_boundary_852	azucena_chr06	4567500	4572500
Azucena	azucena_boundary_853	azucena_chr06	4772500	4817500
Azucena	azucena_boundary_854	azucena_chr06	4882500	4887500
Azucena	azucena_boundary_855	azucena_chr06	4967500	4972500
Azucena	azucena_boundary_856	azucena_chr06	5027500	5032500
Azucena	azucena_boundary_857	azucena_chr06	5152500	5157500
Azucena	azucena_boundary_858	azucena_chr06	5197500	5202500
Azucena	azucena_boundary_859	azucena_chr06	5737500	5782500
Azucena	azucena_boundary_860	azucena_chr06	7287500	7292500
Azucena	azucena_boundary_861	azucena_chr06	7422500	7427500

Azucena	azucena_boundary_862	azucena_chr06	7787500	7792500
Azucena	azucena_boundary_863	azucena_chr06	7847500	7852500
Azucena	azucena_boundary_864	azucena_chr06	7907500	7947500
Azucena	azucena_boundary_865	azucena_chr06	8037500	8042500
Azucena	azucena_boundary_866	azucena_chr06	8097500	8137500
Azucena	azucena_boundary_867	azucena_chr06	8192500	8197500
Azucena	azucena_boundary_868	azucena_chr06	8267500	8272500
Azucena	azucena_boundary_869	azucena_chr06	9282500	9287500
Azucena	azucena_boundary_870	azucena_chr06	9357500	9362500
Azucena	azucena_boundary_871	azucena_chr06	9412500	9417500
Azucena	azucena_boundary_872	azucena_chr06	9672500	9677500
Azucena	azucena_boundary_873	azucena_chr06	9812500	9817500
Azucena	azucena_boundary_874	azucena_chr06	9897500	9902500
Azucena	azucena_boundary_875	azucena_chr06	9987500	9992500
Azucena	azucena_boundary_876	azucena_chr06	10042500	10047500
Azucena	azucena_boundary_877	azucena_chr06	10517500	10522500
Azucena	azucena_boundary_878	azucena_chr06	10592500	10597500
Azucena	azucena_boundary_879	azucena_chr06	10850000	10855000
Azucena	azucena_boundary_880	azucena_chr06	10912500	10917500
Azucena	azucena_boundary_881	azucena_chr06	10962500	10967500
Azucena	azucena_boundary_882	azucena_chr06	11052500	11057500
Azucena	azucena_boundary_883	azucena_chr06	11837500	11842500
Azucena	azucena_boundary_884	azucena_chr06	11967500	11972500
Azucena	azucena_boundary_885	azucena_chr06	12602500	12607500
Azucena	azucena_boundary_886	azucena_chr06	12717500	12722500
Azucena	azucena_boundary_887	azucena_chr06	12762500	12767500
Azucena	azucena_boundary_888	azucena_chr06	13002500	13007500
Azucena	azucena_boundary_889	azucena_chr06	13057500	13062500
Azucena	azucena_boundary_890	azucena_chr06	13147500	13152500
Azucena	azucena_boundary_891	azucena_chr06	13237500	13242500
Azucena	azucena_boundary_892	azucena_chr06	13312500	13317500
Azucena	azucena_boundary_893	azucena_chr06	13367500	13372500

Azucena	azucena_boundary_894	azucena_chr06	14630000	14677500
Azucena	azucena_boundary_895	azucena_chr06	14880000	14885000
Azucena	azucena_boundary_896	azucena_chr06	15005000	15010000
Azucena	azucena_boundary_897	azucena_chr06	15152500	15157500
Azucena	azucena_boundary_898	azucena_chr06	16037500	16042500
Azucena	azucena_boundary_899	azucena_chr06	16127500	16132500
Azucena	azucena_boundary_900	azucena_chr06	16862500	16867500
Azucena	azucena_boundary_901	azucena_chr06	16932500	16937500
Azucena	azucena_boundary_902	azucena_chr06	17007500	17012500
Azucena	azucena_boundary_903	azucena_chr06	17497500	17502500
Azucena	azucena_boundary_904	azucena_chr06	17807500	17812500
Azucena	azucena_boundary_905	azucena_chr06	17872500	17877500
Azucena	azucena_boundary_906	azucena_chr06	17927500	17932500
Azucena	azucena_boundary_907	azucena_chr06	19387500	19392500
Azucena	azucena_boundary_908	azucena_chr06	19452500	19457500
Azucena	azucena_boundary_909	azucena_chr06	19777500	19782500
Azucena	azucena_boundary_910	azucena_chr06	19822500	19827500
Azucena	azucena_boundary_911	azucena_chr06	21302500	21307500
Azucena	azucena_boundary_912	azucena_chr06	21367500	21372500
Azucena	azucena_boundary_913	azucena_chr06	21427500	21432500
Azucena	azucena_boundary_914	azucena_chr06	21517500	21522500
Azucena	azucena_boundary_915	azucena_chr06	21572500	21577500
Azucena	azucena_boundary_916	azucena_chr06	22372500	22377500
Azucena	azucena_boundary_917	azucena_chr06	22442500	22447500
Azucena	azucena_boundary_918	azucena_chr06	22767500	22772500
Azucena	azucena_boundary_919	azucena_chr06	22862500	22867500
Azucena	azucena_boundary_920	azucena_chr06	22937500	22942500
Azucena	azucena_boundary_921	azucena_chr06	23162500	23167500
Azucena	azucena_boundary_922	azucena_chr06	23252500	23257500
Azucena	azucena_boundary_923	azucena_chr06	23652500	23657500
Azucena	azucena_boundary_924	azucena_chr06	23697500	23737500
Azucena	azucena_boundary_925	azucena_chr06	23802500	23807500

Azucena	azucena_boundary_926	azucena_chr06	23847500	23852500
Azucena	azucena_boundary_927	azucena_chr06	23922500	23967500
Azucena	azucena_boundary_928	azucena_chr06	24612500	24617500
Azucena	azucena_boundary_929	azucena_chr06	24672500	24677500
Azucena	azucena_boundary_930	azucena_chr06	25087500	25092500
Azucena	azucena_boundary_931	azucena_chr06	25147500	25152500
Azucena	azucena_boundary_932	azucena_chr06	25197500	25202500
Azucena	azucena_boundary_933	azucena_chr06	25902500	25907500
Azucena	azucena_boundary_934	azucena_chr06	26027500	26032500
Azucena	azucena_boundary_935	azucena_chr06	26137500	26142500
Azucena	azucena_boundary_936	azucena_chr06	26372500	26417500
Azucena	azucena_boundary_937	azucena_chr06	26492500	26497500
Azucena	azucena_boundary_938	azucena_chr06	26712500	26717500
Azucena	azucena_boundary_939	azucena_chr06	26767500	26772500
Azucena	azucena_boundary_940	azucena_chr06	26817500	26822500
Azucena	azucena_boundary_941	azucena_chr06	26892500	26897500
Azucena	azucena_boundary_942	azucena_chr06	27467500	27472500
Azucena	azucena_boundary_943	azucena_chr06	27567500	27572500
Azucena	azucena_boundary_944	azucena_chr06	27632500	27637500
Azucena	azucena_boundary_945	azucena_chr06	27992500	27997500
Azucena	azucena_boundary_946	azucena_chr06	28062500	28067500
Azucena	azucena_boundary_947	azucena_chr06	28142500	28147500
Azucena	azucena_boundary_948	azucena_chr06	28332500	28337500
Azucena	azucena_boundary_949	azucena_chr06	28382500	28387500
Azucena	azucena_boundary_950	azucena_chr06	28447500	28452500
Azucena	azucena_boundary_951	azucena_chr06	28557500	28562500
Azucena	azucena_boundary_952	azucena_chr06	28727500	28732500
Azucena	azucena_boundary_953	azucena_chr06	28827500	28832500
Azucena	azucena_boundary_954	azucena_chr06	28877500	28882500
Azucena	azucena_boundary_955	azucena_chr06	28962500	28967500
Azucena	azucena_boundary_956	azucena_chr06	29782500	29787500
Azucena	azucena_boundary_957	azucena_chr06	29827500	29832500

Azucena	azucena_boundary_958	azucena_chr06	29902500	29907500
Azucena	azucena_boundary_959	azucena_chr06	29962500	29967500
Azucena	azucena_boundary_960	azucena_chr06	30057500	30062500
Azucena	azucena_boundary_961	azucena_chr06	30132500	30137500
Azucena	azucena_boundary_962	azucena_chr06	30442500	30482500
Azucena	azucena_boundary_963	azucena_chr06	30537500	30542500
Azucena	azucena_boundary_964	azucena_chr06	30687500	30692500
Azucena	azucena_boundary_965	azucena_chr06	30757500	30762500
Azucena	azucena_boundary_966	azucena_chr07	592500	597500
Azucena	azucena_boundary_967	azucena_chr07	637500	642500
Azucena	azucena_boundary_968	azucena_chr07	687500	692500
Azucena	azucena_boundary_969	azucena_chr07	1247500	1252500
Azucena	azucena_boundary_970	azucena_chr07	1332500	1372500
Azucena	azucena_boundary_971	azucena_chr07	2045000	2050000
Azucena	azucena_boundary_972	azucena_chr07	2177500	2182500
Azucena	azucena_boundary_973	azucena_chr07	2227500	2232500
Azucena	azucena_boundary_974	azucena_chr07	2420000	2425000
Azucena	azucena_boundary_975	azucena_chr07	2527500	2532500
Azucena	azucena_boundary_976	azucena_chr07	2617500	2622500
Azucena	azucena_boundary_977	azucena_chr07	3567500	3572500
Azucena	azucena_boundary_978	azucena_chr07	3672500	3677500
Azucena	azucena_boundary_979	azucena_chr07	3717500	3722500
Azucena	azucena_boundary_980	azucena_chr07	3802500	3807500
Azucena	azucena_boundary_981	azucena_chr07	3957500	3962500
Azucena	azucena_boundary_982	azucena_chr07	4037500	4042500
Azucena	azucena_boundary_983	azucena_chr07	4127500	4132500
Azucena	azucena_boundary_984	azucena_chr07	4217500	4222500
Azucena	azucena_boundary_985	azucena_chr07	4282500	4287500
Azucena	azucena_boundary_986	azucena_chr07	4357500	4362500
Azucena	azucena_boundary_987	azucena_chr07	4457500	4462500
Azucena	azucena_boundary_988	azucena_chr07	4607500	4612500
Azucena	azucena_boundary_989	azucena_chr07	4662500	4707500

Azucena	azucena_boundary_990	azucena_chr07	4782500	4787500
Azucena	azucena_boundary_991	azucena_chr07	4847500	4852500
Azucena	azucena_boundary_992	azucena_chr07	4897500	4902500
Azucena	azucena_boundary_993	azucena_chr07	4972500	4977500
Azucena	azucena_boundary_994	azucena_chr07	5082500	5087500
Azucena	azucena_boundary_995	azucena_chr07	5477500	5482500
Azucena	azucena_boundary_996	azucena_chr07	5637500	5642500
Azucena	azucena_boundary_997	azucena_chr07	5857500	5862500
Azucena	azucena_boundary_998	azucena_chr07	5922500	5927500
Azucena	azucena_boundary_999	azucena_chr07	7137500	7142500
Azucena	azucena_boundary_1000	azucena_chr07	7212500	7217500
Azucena	azucena_boundary_1001	azucena_chr07	7292500	7297500
Azucena	azucena_boundary_1002	azucena_chr07	7407500	7412500
Azucena	azucena_boundary_1003	azucena_chr07	7562500	7567500
Azucena	azucena_boundary_1004	azucena_chr07	10087500	10092500
Azucena	azucena_boundary_1005	azucena_chr07	10162500	10167500
Azucena	azucena_boundary_1006	azucena_chr07	10302500	10307500
Azucena	azucena_boundary_1007	azucena_chr07	10522500	10527500
Azucena	azucena_boundary_1008	azucena_chr07	10857500	10862500
Azucena	azucena_boundary_1009	azucena_chr07	10922500	10927500
Azucena	azucena_boundary_1010	azucena_chr07	11157500	11162500
Azucena	azucena_boundary_1011	azucena_chr07	11240000	11282500
Azucena	azucena_boundary_1012	azucena_chr07	11362500	11367500
Azucena	azucena_boundary_1013	azucena_chr07	12457500	12462500
Azucena	azucena_boundary_1014	azucena_chr07	12557500	12562500
Azucena	azucena_boundary_1015	azucena_chr07	12627500	12632500
Azucena	azucena_boundary_1016	azucena_chr07	12672500	12677500
Azucena	azucena_boundary_1017	azucena_chr07	12772500	12777500
Azucena	azucena_boundary_1018	azucena_chr07	12877500	12957500
Azucena	azucena_boundary_1019	azucena_chr07	13287500	13292500
Azucena	azucena_boundary_1020	azucena_chr07	13457500	13462500
Azucena	azucena_boundary_1021	azucena_chr07	14147500	14152500

Azucena	azucena_boundary_1022	azucena_chr07	14262500	14267500
Azucena	azucena_boundary_1023	azucena_chr07	14902500	14907500
Azucena	azucena_boundary_1024	azucena_chr07	14947500	14952500
Azucena	azucena_boundary_1025	azucena_chr07	15232500	15237500
Azucena	azucena_boundary_1026	azucena_chr07	15292500	15297500
Azucena	azucena_boundary_1027	azucena_chr07	15357500	15362500
Azucena	azucena_boundary_1028	azucena_chr07	15457500	15462500
Azucena	azucena_boundary_1029	azucena_chr07	15607500	15612500
Azucena	azucena_boundary_1030	azucena_chr07	15777500	15782500
Azucena	azucena_boundary_1031	azucena_chr07	15872500	15877500
Azucena	azucena_boundary_1032	azucena_chr07	15937500	15942500
Azucena	azucena_boundary_1033	azucena_chr07	16032500	16037500
Azucena	azucena_boundary_1034	azucena_chr07	16112500	16117500
Azucena	azucena_boundary_1035	azucena_chr07	16262500	16267500
Azucena	azucena_boundary_1036	azucena_chr07	16327500	16332500
Azucena	azucena_boundary_1037	azucena_chr07	16407500	16412500
Azucena	azucena_boundary_1038	azucena_chr07	16507500	16512500
Azucena	azucena_boundary_1039	azucena_chr07	16932500	16937500
Azucena	azucena_boundary_1040	azucena_chr07	17012500	17017500
Azucena	azucena_boundary_1041	azucena_chr07	17142500	17147500
Azucena	azucena_boundary_1042	azucena_chr07	17242500	17247500
Azucena	azucena_boundary_1043	azucena_chr07	17422500	17427500
Azucena	azucena_boundary_1044	azucena_chr07	17482500	17487500
Azucena	azucena_boundary_1045	azucena_chr07	17727500	17732500
Azucena	azucena_boundary_1046	azucena_chr07	17907500	17912500
Azucena	azucena_boundary_1047	azucena_chr07	19532500	19537500
Azucena	azucena_boundary_1048	azucena_chr07	19602500	19607500
Azucena	azucena_boundary_1049	azucena_chr07	19677500	19717500
Azucena	azucena_boundary_1050	azucena_chr07	19797500	19802500
Azucena	azucena_boundary_1051	azucena_chr07	19992500	20032500
Azucena	azucena_boundary_1052	azucena_chr07	20247500	20252500
Azucena	azucena_boundary_1053	azucena_chr07	20317500	20352500

Azucena	azucena_boundary_1054	azucena_chr07	20477500	20482500
Azucena	azucena_boundary_1055	azucena_chr07	20552500	20557500
Azucena	azucena_boundary_1056	azucena_chr07	20672500	20677500
Azucena	azucena_boundary_1057	azucena_chr07	20777500	20782500
Azucena	azucena_boundary_1058	azucena_chr07	21222500	21227500
Azucena	azucena_boundary_1059	azucena_chr07	21312500	21317500
Azucena	azucena_boundary_1060	azucena_chr07	21382500	21422500
Azucena	azucena_boundary_1061	azucena_chr07	21687500	21692500
Azucena	azucena_boundary_1062	azucena_chr07	21752500	21757500
Azucena	azucena_boundary_1063	azucena_chr07	21807500	21812500
Azucena	azucena_boundary_1064	azucena_chr07	22462500	22502500
Azucena	azucena_boundary_1065	azucena_chr07	22577500	22582500
Azucena	azucena_boundary_1066	azucena_chr07	22727500	22732500
Azucena	azucena_boundary_1067	azucena_chr07	22812500	22817500
Azucena	azucena_boundary_1068	azucena_chr07	23517500	23522500
Azucena	azucena_boundary_1069	azucena_chr07	23582500	23587500
Azucena	azucena_boundary_1070	azucena_chr07	23647500	23692500
Azucena	azucena_boundary_1071	azucena_chr07	23747500	23752500
Azucena	azucena_boundary_1072	azucena_chr07	23827500	23832500
Azucena	azucena_boundary_1073	azucena_chr07	23892500	23897500
Azucena	azucena_boundary_1074	azucena_chr07	24002500	24007500
Azucena	azucena_boundary_1075	azucena_chr07	24072500	24077500
Azucena	azucena_boundary_1076	azucena_chr07	24137500	24172500
Azucena	azucena_boundary_1077	azucena_chr07	24537500	24542500
Azucena	azucena_boundary_1078	azucena_chr07	24597500	24602500
Azucena	azucena_boundary_1079	azucena_chr07	25212500	25217500
Azucena	azucena_boundary_1080	azucena_chr07	25307500	25312500
Azucena	azucena_boundary_1081	azucena_chr07	26495000	26500000
Azucena	azucena_boundary_1082	azucena_chr07	26562500	26567500
Azucena	azucena_boundary_1083	azucena_chr07	27192500	27197500
Azucena	azucena_boundary_1084	azucena_chr07	27262500	27267500
Azucena	azucena_boundary_1085	azucena_chr07	27457500	27462500

Azucena	azucena_boundary_1086	azucena_chr07	27532500	27537500
Azucena	azucena_boundary_1087	azucena_chr07	27867500	27912500
Azucena	azucena_boundary_1088	azucena_chr07	28782500	28787500
Azucena	azucena_boundary_1089	azucena_chr07	28857500	28862500
Azucena	azucena_boundary_1090	azucena_chr07	28952500	28957500
Azucena	azucena_boundary_1091	azucena_chr07	29087500	29092500
Azucena	azucena_boundary_1092	azucena_chr08	57500	62500
Azucena	azucena_boundary_1093	azucena_chr08	112500	117500
Azucena	azucena_boundary_1094	azucena_chr08	757500	762500
Azucena	azucena_boundary_1095	azucena_chr08	902500	907500
Azucena	azucena_boundary_1096	azucena_chr08	972500	977500
Azucena	azucena_boundary_1097	azucena_chr08	1052500	1057500
Azucena	azucena_boundary_1098	azucena_chr08	3302500	3307500
Azucena	azucena_boundary_1099	azucena_chr08	3412500	3417500
Azucena	azucena_boundary_1100	azucena_chr08	3467500	3472500
Azucena	azucena_boundary_1101	azucena_chr08	3522500	3527500
Azucena	azucena_boundary_1102	azucena_chr08	3597500	3602500
Azucena	azucena_boundary_1103	azucena_chr08	3692500	3697500
Azucena	azucena_boundary_1104	azucena_chr08	3792500	3797500
Azucena	azucena_boundary_1105	azucena_chr08	3837500	3842500
Azucena	azucena_boundary_1106	azucena_chr08	3917500	3922500
Azucena	azucena_boundary_1107	azucena_chr08	4757500	4837500
Azucena	azucena_boundary_1108	azucena_chr08	4887500	4892500
Azucena	azucena_boundary_1109	azucena_chr08	5047500	5092500
Azucena	azucena_boundary_1110	azucena_chr08	5192500	5197500
Azucena	azucena_boundary_1111	azucena_chr08	5532500	5537500
Azucena	azucena_boundary_1112	azucena_chr08	5752500	5757500
Azucena	azucena_boundary_1113	azucena_chr08	6387500	6427500
Azucena	azucena_boundary_1114	azucena_chr08	7487500	7492500
Azucena	azucena_boundary_1115	azucena_chr08	7602500	7607500
Azucena	azucena_boundary_1116	azucena_chr08	7847500	7852500
Azucena	azucena_boundary_1117	azucena_chr08	7912500	7917500

Azucena	azucena_boundary_1118	azucena_chr08	8157500	8162500
Azucena	azucena_boundary_1119	azucena_chr08	8217500	8222500
Azucena	azucena_boundary_1120	azucena_chr08	8327500	8332500
Azucena	azucena_boundary_1121	azucena_chr08	8457500	8487500
Azucena	azucena_boundary_1122	azucena_chr08	9012500	9017500
Azucena	azucena_boundary_1123	azucena_chr08	9122500	9127500
Azucena	azucena_boundary_1124	azucena_chr08	9167500	9197500
Azucena	azucena_boundary_1125	azucena_chr08	9460000	9465000
Azucena	azucena_boundary_1126	azucena_chr08	9772500	9777500
Azucena	azucena_boundary_1127	azucena_chr08	9817500	9822500
Azucena	azucena_boundary_1128	azucena_chr08	9962500	9967500
Azucena	azucena_boundary_1129	azucena_chr08	10492500	10497500
Azucena	azucena_boundary_1130	azucena_chr08	10617500	10622500
Azucena	azucena_boundary_1131	azucena_chr08	10690000	10695000
Azucena	azucena_boundary_1132	azucena_chr08	11392500	11397500
Azucena	azucena_boundary_1133	azucena_chr08	11627500	11632500
Azucena	azucena_boundary_1134	azucena_chr08	12237500	12242500
Azucena	azucena_boundary_1135	azucena_chr08	12327500	12332500
Azucena	azucena_boundary_1136	azucena_chr08	12792500	12797500
Azucena	azucena_boundary_1137	azucena_chr08	12840000	12845000
Azucena	azucena_boundary_1138	azucena_chr08	12957500	12962500
Azucena	azucena_boundary_1139	azucena_chr08	14345000	14350000
Azucena	azucena_boundary_1140	azucena_chr08	14442500	14447500
Azucena	azucena_boundary_1141	azucena_chr08	14757500	14762500
Azucena	azucena_boundary_1142	azucena_chr08	14867500	14872500
Azucena	azucena_boundary_1143	azucena_chr08	14932500	14937500
Azucena	azucena_boundary_1144	azucena_chr08	15007500	15012500
Azucena	azucena_boundary_1145	azucena_chr08	15082500	15087500
Azucena	azucena_boundary_1146	azucena_chr08	15147500	15152500
Azucena	azucena_boundary_1147	azucena_chr08	16752500	16757500
Azucena	azucena_boundary_1148	azucena_chr08	16822500	16827500
Azucena	azucena_boundary_1149	azucena_chr08	17742500	17747500

Azucena	azucena_boundary_1150	azucena_chr08	17827500	17832500
Azucena	azucena_boundary_1151	azucena_chr08	18567500	18572500
Azucena	azucena_boundary_1152	azucena_chr08	18622500	18627500
Azucena	azucena_boundary_1153	azucena_chr08	20042500	20047500
Azucena	azucena_boundary_1154	azucena_chr08	20132500	20137500
Azucena	azucena_boundary_1155	azucena_chr08	20202500	20207500
Azucena	azucena_boundary_1156	azucena_chr08	20262500	20267500
Azucena	azucena_boundary_1157	azucena_chr08	20912500	20917500
Azucena	azucena_boundary_1158	azucena_chr08	20987500	20992500
Azucena	azucena_boundary_1159	azucena_chr08	21032500	21037500
Azucena	azucena_boundary_1160	azucena_chr08	21097500	21102500
Azucena	azucena_boundary_1161	azucena_chr08	21575000	21580000
Azucena	azucena_boundary_1162	azucena_chr08	21687500	21692500
Azucena	azucena_boundary_1163	azucena_chr08	21837500	21842500
Azucena	azucena_boundary_1164	azucena_chr08	21917500	21922500
Azucena	azucena_boundary_1165	azucena_chr08	21972500	21977500
Azucena	azucena_boundary_1166	azucena_chr08	22027500	22072500
Azucena	azucena_boundary_1167	azucena_chr08	22262500	22297500
Azucena	azucena_boundary_1168	azucena_chr08	22702500	22742500
Azucena	azucena_boundary_1169	azucena_chr08	22807500	22812500
Azucena	azucena_boundary_1170	azucena_chr08	23662500	23667500
Azucena	azucena_boundary_1171	azucena_chr08	23757500	23762500
Azucena	azucena_boundary_1172	azucena_chr08	23832500	23837500
Azucena	azucena_boundary_1173	azucena_chr08	23892500	23897500
Azucena	azucena_boundary_1174	azucena_chr08	23967500	23972500
Azucena	azucena_boundary_1175	azucena_chr08	24017500	24022500
Azucena	azucena_boundary_1176	azucena_chr08	24097500	24102500
Azucena	azucena_boundary_1177	azucena_chr08	24702500	24737500
Azucena	azucena_boundary_1178	azucena_chr08	24817500	24822500
Azucena	azucena_boundary_1179	azucena_chr08	24867500	24872500
Azucena	azucena_boundary_1180	azucena_chr08	24957500	24962500
Azucena	azucena_boundary_1181	azucena_chr08	25077500	25082500

Azucena	azucena_boundary_1182	azucena_chr08	25152500	25157500
Azucena	azucena_boundary_1183	azucena_chr08	25237500	25242500
Azucena	azucena_boundary_1184	azucena_chr08	25422500	25427500
Azucena	azucena_boundary_1185	azucena_chr08	25532500	25537500
Azucena	azucena_boundary_1186	azucena_chr08	25592500	25597500
Azucena	azucena_boundary_1187	azucena_chr08	26112500	26117500
Azucena	azucena_boundary_1188	azucena_chr08	26177500	26182500
Azucena	azucena_boundary_1189	azucena_chr08	26722500	26727500
Azucena	azucena_boundary_1190	azucena_chr08	26792500	26837500
Azucena	azucena_boundary_1191	azucena_chr08	26877500	26882500
Azucena	azucena_boundary_1192	azucena_chr08	26937500	26942500
Azucena	azucena_boundary_1193	azucena_chr08	27042500	27047500
Azucena	azucena_boundary_1194	azucena_chr08	27132500	27137500
Azucena	azucena_boundary_1195	azucena_chr08	27502500	27507500
Azucena	azucena_boundary_1196	azucena_chr08	27547500	27552500
Azucena	azucena_boundary_1197	azucena_chr08	27637500	27642500
Azucena	azucena_boundary_1198	azucena_chr08	27737500	27742500
Azucena	azucena_boundary_1199	azucena_chr08	27787500	27792500
Azucena	azucena_boundary_1200	azucena_chr08	28057500	28062500
Azucena	azucena_boundary_1201	azucena_chr08	28102500	28132500
Azucena	azucena_boundary_1202	azucena_chr08	28737500	28772500
Azucena	azucena_boundary_1203	azucena_chr08	28817500	28822500
Azucena	azucena_boundary_1204	azucena_chr08	28872500	28877500
Azucena	azucena_boundary_1205	azucena_chr09	2872500	2877500
Azucena	azucena_boundary_1206	azucena_chr09	2977500	2982500
Azucena	azucena_boundary_1207	azucena_chr09	3947500	3952500
Azucena	azucena_boundary_1208	azucena_chr09	3997500	4002500
Azucena	azucena_boundary_1209	azucena_chr09	4127500	4132500
Azucena	azucena_boundary_1210	azucena_chr09	4207500	4212500
Azucena	azucena_boundary_1211	azucena_chr09	4297500	4302500
Azucena	azucena_boundary_1212	azucena_chr09	4417500	4422500
Azucena	azucena_boundary_1213	azucena_chr09	4552500	4557500

Azucena	azucena_boundary_1214	azucena_chr09	4637500	4642500
Azucena	azucena_boundary_1215	azucena_chr09	4817500	4822500
Azucena	azucena_boundary_1216	azucena_chr09	4982500	4987500
Azucena	azucena_boundary_1217	azucena_chr09	5032500	5037500
Azucena	azucena_boundary_1218	azucena_chr09	5182500	5187500
Azucena	azucena_boundary_1219	azucena_chr09	6277500	6282500
Azucena	azucena_boundary_1220	azucena_chr09	6352500	6357500
Azucena	azucena_boundary_1221	azucena_chr09	6537500	6542500
Azucena	azucena_boundary_1222	azucena_chr09	6727500	6732500
Azucena	azucena_boundary_1223	azucena_chr09	6857500	6862500
Azucena	azucena_boundary_1224	azucena_chr09	7922500	7927500
Azucena	azucena_boundary_1225	azucena_chr09	8057500	8062500
Azucena	azucena_boundary_1226	azucena_chr09	8457500	8462500
Azucena	azucena_boundary_1227	azucena_chr09	8512500	8517500
Azucena	azucena_boundary_1228	azucena_chr09	8602500	8607500
Azucena	azucena_boundary_1229	azucena_chr09	8877500	8882500
Azucena	azucena_boundary_1230	azucena_chr09	8930000	8977500
Azucena	azucena_boundary_1231	azucena_chr09	10757500	10762500
Azucena	azucena_boundary_1232	azucena_chr09	10877500	10882500
Azucena	azucena_boundary_1233	azucena_chr09	11502500	11507500
Azucena	azucena_boundary_1234	azucena_chr09	11572500	11612500
Azucena	azucena_boundary_1235	azucena_chr09	11707500	11712500
Azucena	azucena_boundary_1236	azucena_chr09	11782500	11787500
Azucena	azucena_boundary_1237	azucena_chr09	12522500	12527500
Azucena	azucena_boundary_1238	azucena_chr09	12582500	12587500
Azucena	azucena_boundary_1239	azucena_chr09	12882500	12927500
Azucena	azucena_boundary_1240	azucena_chr09	13002500	13007500
Azucena	azucena_boundary_1241	azucena_chr09	13122500	13127500
Azucena	azucena_boundary_1242	azucena_chr09	13277500	13282500
Azucena	azucena_boundary_1243	azucena_chr09	13362500	13367500
Azucena	azucena_boundary_1244	azucena_chr09	13832500	13837500
Azucena	azucena_boundary_1245	azucena_chr09	13882500	13887500

Azucena	azucena_boundary_1246	azucena_chr09	13932500	13937500
Azucena	azucena_boundary_1247	azucena_chr09	14007500	14012500
Azucena	azucena_boundary_1248	azucena_chr09	14057500	14062500
Azucena	azucena_boundary_1249	azucena_chr09	14137500	14142500
Azucena	azucena_boundary_1250	azucena_chr09	14182500	14187500
Azucena	azucena_boundary_1251	azucena_chr09	14647500	14652500
Azucena	azucena_boundary_1252	azucena_chr09	14727500	14732500
Azucena	azucena_boundary_1253	azucena_chr09	14932500	14972500
Azucena	azucena_boundary_1254	azucena_chr09	15297500	15302500
Azucena	azucena_boundary_1255	azucena_chr09	15452500	15497500
Azucena	azucena_boundary_1256	azucena_chr09	15887500	15892500
Azucena	azucena_boundary_1257	azucena_chr09	15947500	15952500
Azucena	azucena_boundary_1258	azucena_chr09	16032500	16037500
Azucena	azucena_boundary_1259	azucena_chr09	16077500	16082500
Azucena	azucena_boundary_1260	azucena_chr09	16382500	16387500
Azucena	azucena_boundary_1261	azucena_chr09	16442500	16447500
Azucena	azucena_boundary_1262	azucena_chr09	16512500	16517500
Azucena	azucena_boundary_1263	azucena_chr09	16587500	16592500
Azucena	azucena_boundary_1264	azucena_chr09	16652500	16657500
Azucena	azucena_boundary_1265	azucena_chr09	16757500	16762500
Azucena	azucena_boundary_1266	azucena_chr09	16842500	16847500
Azucena	azucena_boundary_1267	azucena_chr09	17632500	17672500
Azucena	azucena_boundary_1268	azucena_chr09	17862500	17867500
Azucena	azucena_boundary_1269	azucena_chr09	17922500	17927500
Azucena	azucena_boundary_1270	azucena_chr09	17972500	17977500
Azucena	azucena_boundary_1271	azucena_chr09	18147500	18152500
Azucena	azucena_boundary_1272	azucena_chr09	18252500	18257500
Azucena	azucena_boundary_1273	azucena_chr09	18472500	18512500
Azucena	azucena_boundary_1274	azucena_chr09	18552500	18557500
Azucena	azucena_boundary_1275	azucena_chr09	18617500	18622500
Azucena	azucena_boundary_1276	azucena_chr09	19087500	19092500
Azucena	azucena_boundary_1277	azucena_chr09	19217500	19222500

Azucena	azucena_boundary_1278	azucena_chr09	19262500	19267500
Azucena	azucena_boundary_1279	azucena_chr09	19362500	19367500
Azucena	azucena_boundary_1280	azucena_chr09	19452500	19457500
Azucena	azucena_boundary_1281	azucena_chr09	19532500	19537500
Azucena	azucena_boundary_1282	azucena_chr09	19807500	19812500
Azucena	azucena_boundary_1283	azucena_chr09	19917500	19922500
Azucena	azucena_boundary_1284	azucena_chr09	19977500	19982500
Azucena	azucena_boundary_1285	azucena_chr09	20055000	20060000
Azucena	azucena_boundary_1286	azucena_chr09	20127500	20132500
Azucena	azucena_boundary_1287	azucena_chr09	20197500	20202500
Azucena	azucena_boundary_1288	azucena_chr09	20272500	20277500
Azucena	azucena_boundary_1289	azucena_chr09	20372500	20377500
Azucena	azucena_boundary_1290	azucena_chr09	20447500	20452500
Azucena	azucena_boundary_1291	azucena_chr09	20587500	20627500
Azucena	azucena_boundary_1292	azucena_chr09	20957500	20997500
Azucena	azucena_boundary_1293	azucena_chr09	21362500	21367500
Azucena	azucena_boundary_1294	azucena_chr09	21432500	21437500
Azucena	azucena_boundary_1295	azucena_chr09	21982500	22022500
Azucena	azucena_boundary_1296	azucena_chr09	22357500	22402500
Azucena	azucena_boundary_1297	azucena_chr09	22487500	22492500
Azucena	azucena_boundary_1298	azucena_chr09	22607500	22612500
Azucena	azucena_boundary_1299	azucena_chr10	1567500	1572500
Azucena	azucena_boundary_1300	azucena_chr10	1622500	1627500
Azucena	azucena_boundary_1301	azucena_chr10	1677500	1722500
Azucena	azucena_boundary_1302	azucena_chr10	1787500	1792500
Azucena	azucena_boundary_1303	azucena_chr10	2117500	2122500
Azucena	azucena_boundary_1304	azucena_chr10	2177500	2182500
Azucena	azucena_boundary_1305	azucena_chr10	2932500	2937500
Azucena	azucena_boundary_1306	azucena_chr10	2997500	3002500
Azucena	azucena_boundary_1307	azucena_chr10	3102500	3107500
Azucena	azucena_boundary_1308	azucena_chr10	3322500	3327500
Azucena	azucena_boundary_1309	azucena_chr10	3367500	3372500

Azucena	azucena_boundary_1310	azucena_chr10	4592500	4627500
Azucena	azucena_boundary_1311	azucena_chr10	4717500	4722500
Azucena	azucena_boundary_1312	azucena_chr10	4762500	4767500
Azucena	azucena_boundary_1313	azucena_chr10	4882500	4887500
Azucena	azucena_boundary_1314	azucena_chr10	5132500	5137500
Azucena	azucena_boundary_1315	azucena_chr10	7757500	7762500
Azucena	azucena_boundary_1316	azucena_chr10	7880000	7885000
Azucena	azucena_boundary_1317	azucena_chr10	8062500	8067500
Azucena	azucena_boundary_1318	azucena_chr10	8142500	8147500
Azucena	azucena_boundary_1319	azucena_chr10	8497500	8502500
Azucena	azucena_boundary_1320	azucena_chr10	8600000	8605000
Azucena	azucena_boundary_1321	azucena_chr10	10252500	10257500
Azucena	azucena_boundary_1322	azucena_chr10	10380000	10385000
Azucena	azucena_boundary_1323	azucena_chr10	10747500	10752500
Azucena	azucena_boundary_1324	azucena_chr10	10807500	10812500
Azucena	azucena_boundary_1325	azucena_chr10	11737500	11742500
Azucena	azucena_boundary_1326	azucena_chr10	11812500	11817500
Azucena	azucena_boundary_1327	azucena_chr10	11957500	11962500
Azucena	azucena_boundary_1328	azucena_chr10	12037500	12042500
Azucena	azucena_boundary_1329	azucena_chr10	12297500	12302500
Azucena	azucena_boundary_1330	azucena_chr10	12362500	12367500
Azucena	azucena_boundary_1331	azucena_chr10	12427500	12432500
Azucena	azucena_boundary_1332	azucena_chr10	12477500	12522500
Azucena	azucena_boundary_1333	azucena_chr10	12587500	12592500
Azucena	azucena_boundary_1334	azucena_chr10	12652500	12657500
Azucena	azucena_boundary_1335	azucena_chr10	13352500	13357500
Azucena	azucena_boundary_1336	azucena_chr10	13637500	13642500
Azucena	azucena_boundary_1337	azucena_chr10	13922500	13927500
Azucena	azucena_boundary_1338	azucena_chr10	14002500	14007500
Azucena	azucena_boundary_1339	azucena_chr10	14242500	14247500
Azucena	azucena_boundary_1340	azucena_chr10	14382500	14417500
Azucena	azucena_boundary_1341	azucena_chr10	14457500	14462500

Azucena	azucena_boundary_1342	azucena_chr10	16762500	16767500
Azucena	azucena_boundary_1343	azucena_chr10	16837500	16842500
Azucena	azucena_boundary_1344	azucena_chr10	16922500	16927500
Azucena	azucena_boundary_1345	azucena_chr10	17002500	17007500
Azucena	azucena_boundary_1346	azucena_chr10	17112500	17117500
Azucena	azucena_boundary_1347	azucena_chr10	17197500	17242500
Azucena	azucena_boundary_1348	azucena_chr10	17287500	17292500
Azucena	azucena_boundary_1349	azucena_chr10	17367500	17372500
Azucena	azucena_boundary_1350	azucena_chr10	17422500	17427500
Azucena	azucena_boundary_1351	azucena_chr10	17492500	17497500
Azucena	azucena_boundary_1352	azucena_chr10	18912500	18917500
Azucena	azucena_boundary_1353	azucena_chr10	18977500	18982500
Azucena	azucena_boundary_1354	azucena_chr10	19567500	19572500
Azucena	azucena_boundary_1355	azucena_chr10	19617500	19622500
Azucena	azucena_boundary_1356	azucena_chr10	19732500	19777500
Azucena	azucena_boundary_1357	azucena_chr10	19862500	19867500
Azucena	azucena_boundary_1358	azucena_chr10	19917500	19962500
Azucena	azucena_boundary_1359	azucena_chr10	20052500	20057500
Azucena	azucena_boundary_1360	azucena_chr10	20342500	20347500
Azucena	azucena_boundary_1361	azucena_chr10	20397500	20402500
Azucena	azucena_boundary_1362	azucena_chr10	20497500	20502500
Azucena	azucena_boundary_1363	azucena_chr10	20552500	20557500
Azucena	azucena_boundary_1364	azucena_chr10	20597500	20602500
Azucena	azucena_boundary_1365	azucena_chr10	20667500	20672500
Azucena	azucena_boundary_1366	azucena_chr10	20932500	20937500
Azucena	azucena_boundary_1367	azucena_chr10	20982500	20987500
Azucena	azucena_boundary_1368	azucena_chr10	21042500	21047500
Azucena	azucena_boundary_1369	azucena_chr10	21397500	21402500
Azucena	azucena_boundary_1370	azucena_chr10	21447500	21552500
Azucena	azucena_boundary_1371	azucena_chr10	21792500	21837500
Azucena	azucena_boundary_1372	azucena_chr10	21907500	21912500
Azucena	azucena_boundary_1373	azucena_chr10	21982500	22062500

Azucena	azucena_boundary_1374	azucena_chr10	22447500	22452500
Azucena	azucena_boundary_1375	azucena_chr10	22522500	22527500
Azucena	azucena_boundary_1376	azucena_chr10	22582500	22587500
Azucena	azucena_boundary_1377	azucena_chr10	22627500	22632500
Azucena	azucena_boundary_1378	azucena_chr10	22752500	22757500
Azucena	azucena_boundary_1379	azucena_chr10	22812500	22817500
Azucena	azucena_boundary_1380	azucena_chr10	22882500	22887500
Azucena	azucena_boundary_1381	azucena_chr10	22962500	22967500
Azucena	azucena_boundary_1382	azucena_chr10	23102500	23107500
Azucena	azucena_boundary_1383	azucena_chr10	23187500	23192500
Azucena	azucena_boundary_1384	azucena_chr10	23997500	24037500
Azucena	azucena_boundary_1385	azucena_chr10	24277500	24282500
Azucena	azucena_boundary_1386	azucena_chr10	24402500	24407500
Azucena	azucena_boundary_1387	azucena_chr10	24467500	24472500
Azucena	azucena_boundary_1388	azucena_chr10	24582500	24587500
Azucena	azucena_boundary_1389	azucena_chr10	24637500	24642500
Azucena	azucena_boundary_1390	azucena_chr11	62500	67500
Azucena	azucena_boundary_1391	azucena_chr11	140000	145000
Azucena	azucena_boundary_1392	azucena_chr11	282500	287500
Azucena	azucena_boundary_1393	azucena_chr11	687500	692500
Azucena	azucena_boundary_1394	azucena_chr11	842500	847500
Azucena	azucena_boundary_1395	azucena_chr11	1467500	1472500
Azucena	azucena_boundary_1396	azucena_chr11	1582500	1587500
Azucena	azucena_boundary_1397	azucena_chr11	1647500	1652500
Azucena	azucena_boundary_1398	azucena_chr11	1722500	1727500
Azucena	azucena_boundary_1399	azucena_chr11	1852500	1857500
Azucena	azucena_boundary_1400	azucena_chr11	1932500	1977500
Azucena	azucena_boundary_1401	azucena_chr11	2037500	2042500
Azucena	azucena_boundary_1402	azucena_chr11	2087500	2092500
Azucena	azucena_boundary_1403	azucena_chr11	2182500	2187500
Azucena	azucena_boundary_1404	azucena_chr11	2252500	2282500
Azucena	azucena_boundary_1405	azucena_chr11	2382500	2387500

Azucena	azucena_boundary_1406	azucena_chr11	2562500	2567500
Azucena	azucena_boundary_1407	azucena_chr11	2607500	2612500
Azucena	azucena_boundary_1408	azucena_chr11	3012500	3017500
Azucena	azucena_boundary_1409	azucena_chr11	3077500	3082500
Azucena	azucena_boundary_1410	azucena_chr11	4392500	4397500
Azucena	azucena_boundary_1411	azucena_chr11	4482500	4487500
Azucena	azucena_boundary_1412	azucena_chr11	4867500	4872500
Azucena	azucena_boundary_1413	azucena_chr11	4957500	4962500
Azucena	azucena_boundary_1414	azucena_chr11	5767500	5772500
Azucena	azucena_boundary_1415	azucena_chr11	5817500	5822500
Azucena	azucena_boundary_1416	azucena_chr11	6175000	6217500
Azucena	azucena_boundary_1417	azucena_chr11	6322500	6327500
Azucena	azucena_boundary_1418	azucena_chr11	6392500	6397500
Azucena	azucena_boundary_1419	azucena_chr11	6487500	6492500
Azucena	azucena_boundary_1420	azucena_chr11	6992500	6997500
Azucena	azucena_boundary_1421	azucena_chr11	7077500	7082500
Azucena	azucena_boundary_1422	azucena_chr11	7152500	7157500
Azucena	azucena_boundary_1423	azucena_chr11	7227500	7232500
Azucena	azucena_boundary_1424	azucena_chr11	7312500	7317500
Azucena	azucena_boundary_1425	azucena_chr11	7477500	7482500
Azucena	azucena_boundary_1426	azucena_chr11	7542500	7547500
Azucena	azucena_boundary_1427	azucena_chr11	7687500	7692500
Azucena	azucena_boundary_1428	azucena_chr11	7747500	7752500
Azucena	azucena_boundary_1429	azucena_chr11	7792500	7797500
Azucena	azucena_boundary_1430	azucena_chr11	7847500	7852500
Azucena	azucena_boundary_1431	azucena_chr11	7907500	7912500
Azucena	azucena_boundary_1432	azucena_chr11	10532500	10537500
Azucena	azucena_boundary_1433	azucena_chr11	10582500	10587500
Azucena	azucena_boundary_1434	azucena_chr11	13892500	13897500
Azucena	azucena_boundary_1435	azucena_chr11	14042500	14047500
Azucena	azucena_boundary_1436	azucena_chr11	14152500	14157500
Azucena	azucena_boundary_1437	azucena_chr11	14312500	14317500

Azucena	azucena_boundary_1438	azucena_chr11	14952500	14997500
Azucena	azucena_boundary_1439	azucena_chr11	15067500	15072500
Azucena	azucena_boundary_1440	azucena_chr11	15652500	15657500
Azucena	azucena_boundary_1441	azucena_chr11	15722500	15767500
Azucena	azucena_boundary_1442	azucena_chr11	16372500	16377500
Azucena	azucena_boundary_1443	azucena_chr11	16467500	16472500
Azucena	azucena_boundary_1444	azucena_chr11	17112500	17147500
Azucena	azucena_boundary_1445	azucena_chr11	17242500	17247500
Azucena	azucena_boundary_1446	azucena_chr11	17312500	17317500
Azucena	azucena_boundary_1447	azucena_chr11	17512500	17542500
Azucena	azucena_boundary_1448	azucena_chr11	17582500	17587500
Azucena	azucena_boundary_1449	azucena_chr11	17762500	17767500
Azucena	azucena_boundary_1450	azucena_chr11	18147500	18152500
Azucena	azucena_boundary_1451	azucena_chr11	18197500	18202500
Azucena	azucena_boundary_1452	azucena_chr11	18587500	18592500
Azucena	azucena_boundary_1453	azucena_chr11	18642500	18647500
Azucena	azucena_boundary_1454	azucena_chr11	18887500	18892500
Azucena	azucena_boundary_1455	azucena_chr11	18997500	19002500
Azucena	azucena_boundary_1456	azucena_chr11	19177500	19222500
Azucena	azucena_boundary_1457	azucena_chr11	19277500	19282500
Azucena	azucena_boundary_1458	azucena_chr11	19322500	19327500
Azucena	azucena_boundary_1459	azucena_chr11	19422500	19427500
Azucena	azucena_boundary_1460	azucena_chr11	19847500	19852500
Azucena	azucena_boundary_1461	azucena_chr11	19932500	19937500
Azucena	azucena_boundary_1462	azucena_chr11	19982500	19987500
Azucena	azucena_boundary_1463	azucena_chr11	20032500	20037500
Azucena	azucena_boundary_1464	azucena_chr11	20082500	20087500
Azucena	azucena_boundary_1465	azucena_chr11	20162500	20207500
Azucena	azucena_boundary_1466	azucena_chr11	20887500	20892500
Azucena	azucena_boundary_1467	azucena_chr11	20962500	20967500
Azucena	azucena_boundary_1468	azucena_chr11	21007500	21047500
Azucena	azucena_boundary_1469	azucena_chr11	21497500	21502500

Azucena	azucena_boundary_1470	azucena_chr11	21632500	21637500
Azucena	azucena_boundary_1471	azucena_chr11	21712500	21717500
Azucena	azucena_boundary_1472	azucena_chr11	21792500	21797500
Azucena	azucena_boundary_1473	azucena_chr11	21885000	21890000
Azucena	azucena_boundary_1474	azucena_chr11	21987500	21992500
Azucena	azucena_boundary_1475	azucena_chr11	22047500	22052500
Azucena	azucena_boundary_1476	azucena_chr11	22487500	22492500
Azucena	azucena_boundary_1477	azucena_chr11	22552500	22557500
Azucena	azucena_boundary_1478	azucena_chr11	23067500	23072500
Azucena	azucena_boundary_1479	azucena_chr11	23122500	23127500
Azucena	azucena_boundary_1480	azucena_chr11	23537500	23542500
Azucena	azucena_boundary_1481	azucena_chr11	23602500	23607500
Azucena	azucena_boundary_1482	azucena_chr11	23662500	23667500
Azucena	azucena_boundary_1483	azucena_chr11	24152500	24157500
Azucena	azucena_boundary_1484	azucena_chr11	24262500	24267500
Azucena	azucena_boundary_1485	azucena_chr11	24362500	24367500
Azucena	azucena_boundary_1486	azucena_chr11	24422500	24427500
Azucena	azucena_boundary_1487	azucena_chr11	24527500	24532500
Azucena	azucena_boundary_1488	azucena_chr11	24602500	24607500
Azucena	azucena_boundary_1489	azucena_chr11	25497500	25502500
Azucena	azucena_boundary_1490	azucena_chr11	25577500	25582500
Azucena	azucena_boundary_1491	azucena_chr11	26147500	26152500
Azucena	azucena_boundary_1492	azucena_chr11	26217500	26222500
Azucena	azucena_boundary_1493	azucena_chr11	26315000	26320000
Azucena	azucena_boundary_1494	azucena_chr11	26587500	26592500
Azucena	azucena_boundary_1495	azucena_chr11	26672500	26677500
Azucena	azucena_boundary_1496	azucena_chr11	26727500	26732500
Azucena	azucena_boundary_1497	azucena_chr11	26787500	26832500
Azucena	azucena_boundary_1498	azucena_chr11	26977500	27017500
Azucena	azucena_boundary_1499	azucena_chr11	27117500	27122500
Azucena	azucena_boundary_1500	azucena_chr12	642500	647500
Azucena	azucena_boundary_1501	azucena_chr12	797500	802500

Azucena	azucena_boundary_1502	azucena_chr12	1142500	1147500
Azucena	azucena_boundary_1503	azucena_chr12	1252500	1257500
Azucena	azucena_boundary_1504	azucena_chr12	1602500	1607500
Azucena	azucena_boundary_1505	azucena_chr12	1687500	1692500
Azucena	azucena_boundary_1506	azucena_chr12	2097500	2102500
Azucena	azucena_boundary_1507	azucena_chr12	2197500	2202500
Azucena	azucena_boundary_1508	azucena_chr12	2267500	2272500
Azucena	azucena_boundary_1509	azucena_chr12	2347500	2352500
Azucena	azucena_boundary_1510	azucena_chr12	2557500	2562500
Azucena	azucena_boundary_1511	azucena_chr12	2602500	2607500
Azucena	azucena_boundary_1512	azucena_chr12	2687500	2692500
Azucena	azucena_boundary_1513	azucena_chr12	2787500	2792500
Azucena	azucena_boundary_1514	azucena_chr12	2852500	2887500
Azucena	azucena_boundary_1515	azucena_chr12	2927500	2972500
Azucena	azucena_boundary_1516	azucena_chr12	3132500	3137500
Azucena	azucena_boundary_1517	azucena_chr12	3382500	3387500
Azucena	azucena_boundary_1518	azucena_chr12	3442500	3447500
Azucena	azucena_boundary_1519	azucena_chr12	3542500	3547500
Azucena	azucena_boundary_1520	azucena_chr12	3807500	3812500
Azucena	azucena_boundary_1521	azucena_chr12	4472500	4502500
Azucena	azucena_boundary_1522	azucena_chr12	4582500	4587500
Azucena	azucena_boundary_1523	azucena_chr12	4692500	4697500
Azucena	azucena_boundary_1524	azucena_chr12	4762500	4792500
Azucena	azucena_boundary_1525	azucena_chr12	5937500	5942500
Azucena	azucena_boundary_1526	azucena_chr12	6217500	6222500
Azucena	azucena_boundary_1527	azucena_chr12	6392500	6397500
Azucena	azucena_boundary_1528	azucena_chr12	6452500	6457500
Azucena	azucena_boundary_1529	azucena_chr12	6700000	6737500
Azucena	azucena_boundary_1530	azucena_chr12	6797500	6802500
Azucena	azucena_boundary_1531	azucena_chr12	6917500	6922500
Azucena	azucena_boundary_1532	azucena_chr12	8500000	8505000
Azucena	azucena_boundary_1533	azucena_chr12	8547500	8552500

Azucena	azucena_boundary_1534	azucena_chr12	8607500	8612500
Azucena	azucena_boundary_1535	azucena_chr12	8855000	8860000
Azucena	azucena_boundary_1536	azucena_chr12	8942500	8947500
Azucena	azucena_boundary_1537	azucena_chr12	8987500	8992500
Azucena	azucena_boundary_1538	azucena_chr12	9067500	9072500
Azucena	azucena_boundary_1539	azucena_chr12	9162500	9167500
Azucena	azucena_boundary_1540	azucena_chr12	9242500	9247500
Azucena	azucena_boundary_1541	azucena_chr12	9932500	9937500
Azucena	azucena_boundary_1542	azucena_chr12	10237500	10242500
Azucena	azucena_boundary_1543	azucena_chr12	10292500	10297500
Azucena	azucena_boundary_1544	azucena_chr12	10352500	10357500
Azucena	azucena_boundary_1545	azucena_chr12	11272500	11277500
Azucena	azucena_boundary_1546	azucena_chr12	11330000	11335000
Azucena	azucena_boundary_1547	azucena_chr12	12672500	12677500
Azucena	azucena_boundary_1548	azucena_chr12	12772500	12777500
Azucena	azucena_boundary_1549	azucena_chr12	12902500	12907500
Azucena	azucena_boundary_1550	azucena_chr12	12952500	12957500
Azucena	azucena_boundary_1551	azucena_chr12	13037500	13042500
Azucena	azucena_boundary_1552	azucena_chr12	13207500	13212500
Azucena	azucena_boundary_1553	azucena_chr12	13307500	13312500
Azucena	azucena_boundary_1554	azucena_chr12	13602500	13607500
Azucena	azucena_boundary_1555	azucena_chr12	13652500	13657500
Azucena	azucena_boundary_1556	azucena_chr12	15072500	15077500
Azucena	azucena_boundary_1557	azucena_chr12	15140000	15145000
Azucena	azucena_boundary_1558	azucena_chr12	15307500	15312500
Azucena	azucena_boundary_1559	azucena_chr12	15377500	15382500
Azucena	azucena_boundary_1560	azucena_chr12	17665000	17670000
Azucena	azucena_boundary_1561	azucena_chr12	17857500	17862500
Azucena	azucena_boundary_1562	azucena_chr12	18067500	18107500
Azucena	azucena_boundary_1563	azucena_chr12	18337500	18342500
Azucena	azucena_boundary_1564	azucena_chr12	18422500	18427500
Azucena	azucena_boundary_1565	azucena_chr12	18952500	18957500

Azucena	azucena_boundary_1566	azucena_chr12	19002500	19007500
Azucena	azucena_boundary_1567	azucena_chr12	19342500	19382500
Azucena	azucena_boundary_1568	azucena_chr12	20217500	20222500
Azucena	azucena_boundary_1569	azucena_chr12	20282500	20287500
Azucena	azucena_boundary_1570	azucena_chr12	21492500	21497500
Azucena	azucena_boundary_1571	azucena_chr12	21547500	21552500
Azucena	azucena_boundary_1572	azucena_chr12	22302500	22307500
Azucena	azucena_boundary_1573	azucena_chr12	22347500	22352500
Azucena	azucena_boundary_1574	azucena_chr12	22417500	22422500
Azucena	azucena_boundary_1575	azucena_chr12	22787500	22792500
Azucena	azucena_boundary_1576	azucena_chr12	22877500	22882500
Azucena	azucena_boundary_1577	azucena_chr12	23027500	23032500
Azucena	azucena_boundary_1578	azucena_chr12	23167500	23172500
Azucena	azucena_boundary_1579	azucena_chr12	23287500	23292500
Azucena	azucena_boundary_1580	azucena_chr12	23362500	23367500
Azucena	azucena_boundary_1581	azucena_chr12	23462500	23467500
Azucena	azucena_boundary_1582	azucena_chr12	23727500	23732500
Azucena	azucena_boundary_1583	azucena_chr12	23837500	23872500
Azucena	azucena_boundary_1584	azucena_chr12	23942500	23947500
Azucena	azucena_boundary_1585	azucena_chr12	25442500	25447500
Azucena	azucena_boundary_1586	azucena_chr12	25497500	25502500
Azucena	azucena_boundary_1587	azucena_chr12	25542500	25547500
Azucena	azucena_boundary_1588	azucena_chr12	25852500	25857500
Azucena	azucena_boundary_1589	azucena_chr12	25897500	25902500
Azucena	azucena_boundary_1590	azucena_chr12	26032500	26072500
Azucena	azucena_boundary_1591	azucena_chr12	26392500	26397500
Azucena	azucena_boundary_1592	azucena_chr12	26447500	26452500
Azucena	azucena_boundary_1593	azucena_chr12	26492500	26497500
Azucena	azucena_boundary_1594	azucena_chr12	26557500	26562500
Azucena	azucena_boundary_1595	azucena_chr12	26852500	26857500
Azucena	azucena_boundary_1596	azucena_chr12	26947500	26952500
Azucena	azucena_boundary_1597	azucena_chr12	27042500	27047500

Azucena	azucena_boundary_1598	azucena_chr12	27117500	27122500
Azucena	azucena_boundary_1599	azucena_chr12	27162500	27167500
Azucena	azucena_boundary_1600	azucena_chr12	27212500	27217500
Nipponbare	nipponbare_boundary_0	nipponbare_chr01	147500	152500
Nipponbare	nipponbare_boundary_1	nipponbare_chr01	207500	212500
Nipponbare	nipponbare_boundary_2	nipponbare_chr01	367500	372500
Nipponbare	nipponbare_boundary_3	nipponbare_chr01	417500	422500
Nipponbare	nipponbare_boundary_4	nipponbare_chr01	522500	562500
Nipponbare	nipponbare_boundary_5	nipponbare_chr01	1672500	1677500
Nipponbare	nipponbare_boundary_6	nipponbare_chr01	1762500	1767500
Nipponbare	nipponbare_boundary_7	nipponbare_chr01	1997500	2002500
Nipponbare	nipponbare_boundary_8	nipponbare_chr01	2052500	2057500
Nipponbare	nipponbare_boundary_9	nipponbare_chr01	2587500	2592500
Nipponbare	nipponbare_boundary_10	nipponbare_chr01	2637500	2642500
Nipponbare	nipponbare_boundary_11	nipponbare_chr01	2692500	2697500
Nipponbare	nipponbare_boundary_12	nipponbare_chr01	2807500	2842500
Nipponbare	nipponbare_boundary_13	nipponbare_chr01	2887500	2892500
Nipponbare	nipponbare_boundary_14	nipponbare_chr01	2962500	2967500
Nipponbare	nipponbare_boundary_15	nipponbare_chr01	3047500	3092500
Nipponbare	nipponbare_boundary_16	nipponbare_chr01	3322500	3327500
Nipponbare	nipponbare_boundary_17	nipponbare_chr01	3422500	3427500
Nipponbare	nipponbare_boundary_18	nipponbare_chr01	3692500	3697500
Nipponbare	nipponbare_boundary_19	nipponbare_chr01	3742500	3747500
Nipponbare	nipponbare_boundary_20	nipponbare_chr01	4077500	4122500
Nipponbare	nipponbare_boundary_21	nipponbare_chr01	4722500	4802500
Nipponbare	nipponbare_boundary_22	nipponbare_chr01	4977500	5022500
Nipponbare	nipponbare_boundary_23	nipponbare_chr01	5397500	5402500
Nipponbare	nipponbare_boundary_24	nipponbare_chr01	5477500	5482500
Nipponbare	nipponbare_boundary_25	nipponbare_chr01	5542500	5547500
Nipponbare	nipponbare_boundary_26	nipponbare_chr01	5792500	5797500
Nipponbare	nipponbare_boundary_27	nipponbare_chr01	5882500	5887500
Nipponbare	nipponbare_boundary_28	nipponbare_chr01	5982500	6027500

Nipponbare	nipponbare_boundary_29	nipponbare_chr01	6112500	6117500
Nipponbare	nipponbare_boundary_30	nipponbare_chr01	6192500	6197500
Nipponbare	nipponbare_boundary_31	nipponbare_chr01	6307500	6312500
Nipponbare	nipponbare_boundary_32	nipponbare_chr01	6392500	6397500
Nipponbare	nipponbare_boundary_33	nipponbare_chr01	6462500	6467500
Nipponbare	nipponbare_boundary_34	nipponbare_chr01	6612500	6647500
Nipponbare	nipponbare_boundary_35	nipponbare_chr01	7522500	7527500
Nipponbare	nipponbare_boundary_36	nipponbare_chr01	7577500	7582500
Nipponbare	nipponbare_boundary_37	nipponbare_chr01	7707500	7747500
Nipponbare	nipponbare_boundary_38	nipponbare_chr01	7812500	7817500
Nipponbare	nipponbare_boundary_39	nipponbare_chr01	8197500	8202500
Nipponbare	nipponbare_boundary_40	nipponbare_chr01	8267500	8272500
Nipponbare	nipponbare_boundary_41	nipponbare_chr01	8732500	8737500
Nipponbare	nipponbare_boundary_42	nipponbare_chr01	8782500	8787500
Nipponbare	nipponbare_boundary_43	nipponbare_chr01	9012500	9017500
Nipponbare	nipponbare_boundary_44	nipponbare_chr01	9062500	9067500
Nipponbare	nipponbare_boundary_45	nipponbare_chr01	9112500	9117500
Nipponbare	nipponbare_boundary_46	nipponbare_chr01	9167500	9172500
Nipponbare	nipponbare_boundary_47	nipponbare_chr01	9272500	9277500
Nipponbare	nipponbare_boundary_48	nipponbare_chr01	9327500	9332500
Nipponbare	nipponbare_boundary_49	nipponbare_chr01	10117500	10122500
Nipponbare	nipponbare_boundary_50	nipponbare_chr01	10192500	10197500
Nipponbare	nipponbare_boundary_51	nipponbare_chr01	10262500	10327500
Nipponbare	nipponbare_boundary_52	nipponbare_chr01	10487500	10492500
Nipponbare	nipponbare_boundary_53	nipponbare_chr01	10557500	10602500
Nipponbare	nipponbare_boundary_54	nipponbare_chr01	10897500	10902500
Nipponbare	nipponbare_boundary_55	nipponbare_chr01	10982500	10987500
Nipponbare	nipponbare_boundary_56	nipponbare_chr01	11037500	11042500
Nipponbare	nipponbare_boundary_57	nipponbare_chr01	11082500	11087500
Nipponbare	nipponbare_boundary_58	nipponbare_chr01	11622500	11627500
Nipponbare	nipponbare_boundary_59	nipponbare_chr01	11687500	11692500
Nipponbare	nipponbare_boundary_60	nipponbare_chr01	11747500	11752500

Nipponbare	nipponbare_boundary_61	nipponbare_chr01	11902500	11907500
Nipponbare	nipponbare_boundary_62	nipponbare_chr01	11972500	11977500
Nipponbare	nipponbare_boundary_63	nipponbare_chr01	12077500	12082500
Nipponbare	nipponbare_boundary_64	nipponbare_chr01	12127500	12132500
Nipponbare	nipponbare_boundary_65	nipponbare_chr01	13937500	13942500
Nipponbare	nipponbare_boundary_66	nipponbare_chr01	14062500	14067500
Nipponbare	nipponbare_boundary_67	nipponbare_chr01	14227500	14232500
Nipponbare	nipponbare_boundary_68	nipponbare_chr01	14467500	14472500
Nipponbare	nipponbare_boundary_69	nipponbare_chr01	14512500	14517500
Nipponbare	nipponbare_boundary_70	nipponbare_chr01	14617500	14622500
Nipponbare	nipponbare_boundary_71	nipponbare_chr01	14832500	14837500
Nipponbare	nipponbare_boundary_72	nipponbare_chr01	14992500	14997500
Nipponbare	nipponbare_boundary_73	nipponbare_chr01	15182500	15187500
Nipponbare	nipponbare_boundary_74	nipponbare_chr01	15282500	15287500
Nipponbare	nipponbare_boundary_75	nipponbare_chr01	15382500	15387500
Nipponbare	nipponbare_boundary_76	nipponbare_chr01	15457500	15462500
Nipponbare	nipponbare_boundary_77	nipponbare_chr01	15577500	15582500
Nipponbare	nipponbare_boundary_78	nipponbare_chr01	16347500	16392500
Nipponbare	nipponbare_boundary_79	nipponbare_chr01	17042500	17047500
Nipponbare	nipponbare_boundary_80	nipponbare_chr01	17147500	17152500
Nipponbare	nipponbare_boundary_81	nipponbare_chr01	17222500	17227500
Nipponbare	nipponbare_boundary_82	nipponbare_chr01	19002500	19007500
Nipponbare	nipponbare_boundary_83	nipponbare_chr01	19072500	19077500
Nipponbare	nipponbare_boundary_84	nipponbare_chr01	19122500	19127500
Nipponbare	nipponbare_boundary_85	nipponbare_chr01	20537500	20570000
Nipponbare	nipponbare_boundary_86	nipponbare_chr01	21192500	21197500
Nipponbare	nipponbare_boundary_87	nipponbare_chr01	21277500	21282500
Nipponbare	nipponbare_boundary_88	nipponbare_chr01	21552500	21557500
Nipponbare	nipponbare_boundary_89	nipponbare_chr01	21627500	21632500
Nipponbare	nipponbare_boundary_90	nipponbare_chr01	21972500	21977500
Nipponbare	nipponbare_boundary_91	nipponbare_chr01	22077500	22082500
Nipponbare	nipponbare_boundary_92	nipponbare_chr01	22155000	22160000

Nipponbare	nipponbare_boundary_93	nipponbare_chr01	22257500	22262500
Nipponbare	nipponbare_boundary_94	nipponbare_chr01	22332500	22337500
Nipponbare	nipponbare_boundary_95	nipponbare_chr01	22382500	22387500
Nipponbare	nipponbare_boundary_96	nipponbare_chr01	22447500	22452500
Nipponbare	nipponbare_boundary_97	nipponbare_chr01	22532500	22537500
Nipponbare	nipponbare_boundary_98	nipponbare_chr01	22587500	22632500
Nipponbare	nipponbare_boundary_99	nipponbare_chr01	23322500	23352500
Nipponbare	nipponbare_boundary_100	nipponbare_chr01	23432500	23437500
Nipponbare	nipponbare_boundary_101	nipponbare_chr01	23512500	23582500
Nipponbare	nipponbare_boundary_102	nipponbare_chr01	23642500	23647500
Nipponbare	nipponbare_boundary_103	nipponbare_chr01	23777500	23782500
Nipponbare	nipponbare_boundary_104	nipponbare_chr01	23852500	23887500
Nipponbare	nipponbare_boundary_105	nipponbare_chr01	24097500	24102500
Nipponbare	nipponbare_boundary_106	nipponbare_chr01	24167500	24172500
Nipponbare	nipponbare_boundary_107	nipponbare_chr01	24272500	24277500
Nipponbare	nipponbare_boundary_108	nipponbare_chr01	24357500	24402500
Nipponbare	nipponbare_boundary_109	nipponbare_chr01	24482500	24487500
Nipponbare	nipponbare_boundary_110	nipponbare_chr01	24602500	24607500
Nipponbare	nipponbare_boundary_111	nipponbare_chr01	24852500	24857500
Nipponbare	nipponbare_boundary_112	nipponbare_chr01	24902500	24907500
Nipponbare	nipponbare_boundary_113	nipponbare_chr01	25002500	25007500
Nipponbare	nipponbare_boundary_114	nipponbare_chr01	25067500	25072500
Nipponbare	nipponbare_boundary_115	nipponbare_chr01	25122500	25127500
Nipponbare	nipponbare_boundary_116	nipponbare_chr01	25187500	25192500
Nipponbare	nipponbare_boundary_117	nipponbare_chr01	25362500	25367500
Nipponbare	nipponbare_boundary_118	nipponbare_chr01	25442500	25447500
Nipponbare	nipponbare_boundary_119	nipponbare_chr01	25507500	25512500
Nipponbare	nipponbare_boundary_120	nipponbare_chr01	25582500	25587500
Nipponbare	nipponbare_boundary_121	nipponbare_chr01	25630000	25635000
Nipponbare	nipponbare_boundary_122	nipponbare_chr01	25752500	25757500
Nipponbare	nipponbare_boundary_123	nipponbare_chr01	25797500	25802500
Nipponbare	nipponbare_boundary_124	nipponbare_chr01	26032500	26037500

Nipponbare	nipponbare_boundary_125	nipponbare_chr01	26092500	26097500
Nipponbare	nipponbare_boundary_126	nipponbare_chr01	26157500	26162500
Nipponbare	nipponbare_boundary_127	nipponbare_chr01	26462500	26467500
Nipponbare	nipponbare_boundary_128	nipponbare_chr01	26522500	26527500
Nipponbare	nipponbare_boundary_129	nipponbare_chr01	26587500	26592500
Nipponbare	nipponbare_boundary_130	nipponbare_chr01	26707500	26712500
Nipponbare	nipponbare_boundary_131	nipponbare_chr01	27142500	27187500
Nipponbare	nipponbare_boundary_132	nipponbare_chr01	27237500	27242500
Nipponbare	nipponbare_boundary_133	nipponbare_chr01	27297500	27302500
Nipponbare	nipponbare_boundary_134	nipponbare_chr01	27597500	27627500
Nipponbare	nipponbare_boundary_135	nipponbare_chr01	27682500	27727500
Nipponbare	nipponbare_boundary_136	nipponbare_chr01	27805000	27810000
Nipponbare	nipponbare_boundary_137	nipponbare_chr01	27932500	27937500
Nipponbare	nipponbare_boundary_138	nipponbare_chr01	28007500	28012500
Nipponbare	nipponbare_boundary_139	nipponbare_chr01	28057500	28062500
Nipponbare	nipponbare_boundary_140	nipponbare_chr01	28122500	28127500
Nipponbare	nipponbare_boundary_141	nipponbare_chr01	28332500	28452500
Nipponbare	nipponbare_boundary_142	nipponbare_chr01	28542500	28547500
Nipponbare	nipponbare_boundary_143	nipponbare_chr01	28602500	28607500
Nipponbare	nipponbare_boundary_144	nipponbare_chr01	29242500	29287500
Nipponbare	nipponbare_boundary_145	nipponbare_chr01	29402500	29512500
Nipponbare	nipponbare_boundary_146	nipponbare_chr01	29967500	29972500
Nipponbare	nipponbare_boundary_147	nipponbare_chr01	30022500	30027500
Nipponbare	nipponbare_boundary_148	nipponbare_chr01	30267500	30302500
Nipponbare	nipponbare_boundary_149	nipponbare_chr01	31062500	31067500
Nipponbare	nipponbare_boundary_150	nipponbare_chr01	31202500	31207500
Nipponbare	nipponbare_boundary_151	nipponbare_chr01	31247500	31252500
Nipponbare	nipponbare_boundary_152	nipponbare_chr01	31317500	31322500
Nipponbare	nipponbare_boundary_153	nipponbare_chr01	31362500	31367500
Nipponbare	nipponbare_boundary_154	nipponbare_chr01	32142500	32147500
Nipponbare	nipponbare_boundary_155	nipponbare_chr01	32187500	32192500
Nipponbare	nipponbare_boundary_156	nipponbare_chr01	32237500	32272500

Nipponbare	nipponbare_boundary_157	nipponbare_chr01	32322500	32327500
Nipponbare	nipponbare_boundary_158	nipponbare_chr01	32467500	32472500
Nipponbare	nipponbare_boundary_159	nipponbare_chr01	32612500	32617500
Nipponbare	nipponbare_boundary_160	nipponbare_chr01	32667500	32672500
Nipponbare	nipponbare_boundary_161	nipponbare_chr01	32937500	32942500
Nipponbare	nipponbare_boundary_162	nipponbare_chr01	33042500	33047500
Nipponbare	nipponbare_boundary_163	nipponbare_chr01	33585000	33590000
Nipponbare	nipponbare_boundary_164	nipponbare_chr01	33652500	33697500
Nipponbare	nipponbare_boundary_165	nipponbare_chr01	33747500	33752500
Nipponbare	nipponbare_boundary_166	nipponbare_chr01	33797500	33802500
Nipponbare	nipponbare_boundary_167	nipponbare_chr01	33887500	33892500
Nipponbare	nipponbare_boundary_168	nipponbare_chr01	34467500	34472500
Nipponbare	nipponbare_boundary_169	nipponbare_chr01	34547500	34552500
Nipponbare	nipponbare_boundary_170	nipponbare_chr01	34632500	34637500
Nipponbare	nipponbare_boundary_171	nipponbare_chr01	34802500	34847500
Nipponbare	nipponbare_boundary_172	nipponbare_chr01	35657500	35662500
Nipponbare	nipponbare_boundary_173	nipponbare_chr01	35727500	35822500
Nipponbare	nipponbare_boundary_174	nipponbare_chr01	35872500	35877500
Nipponbare	nipponbare_boundary_175	nipponbare_chr01	36002500	36007500
Nipponbare	nipponbare_boundary_176	nipponbare_chr01	36082500	36087500
Nipponbare	nipponbare_boundary_177	nipponbare_chr01	36132500	36167500
Nipponbare	nipponbare_boundary_178	nipponbare_chr01	36227500	36232500
Nipponbare	nipponbare_boundary_179	nipponbare_chr01	36322500	36327500
Nipponbare	nipponbare_boundary_180	nipponbare_chr01	36392500	36397500
Nipponbare	nipponbare_boundary_181	nipponbare_chr01	36437500	36442500
Nipponbare	nipponbare_boundary_182	nipponbare_chr01	37057500	37062500
Nipponbare	nipponbare_boundary_183	nipponbare_chr01	37147500	37152500
Nipponbare	nipponbare_boundary_184	nipponbare_chr01	37272500	37317500
Nipponbare	nipponbare_boundary_185	nipponbare_chr01	37362500	37367500
Nipponbare	nipponbare_boundary_186	nipponbare_chr01	37682500	37722500
Nipponbare	nipponbare_boundary_187	nipponbare_chr01	38167500	38207500
Nipponbare	nipponbare_boundary_188	nipponbare_chr01	38322500	38327500

Nipponbare	nipponbare_boundary_189	nipponbare_chr01	38387500	38392500
Nipponbare	nipponbare_boundary_190	nipponbare_chr01	38472500	38477500
Nipponbare	nipponbare_boundary_191	nipponbare_chr01	38517500	38522500
Nipponbare	nipponbare_boundary_192	nipponbare_chr01	38702500	38707500
Nipponbare	nipponbare_boundary_193	nipponbare_chr01	38747500	38752500
Nipponbare	nipponbare_boundary_194	nipponbare_chr01	38792500	38797500
Nipponbare	nipponbare_boundary_195	nipponbare_chr01	38852500	38857500
Nipponbare	nipponbare_boundary_196	nipponbare_chr01	38902500	38907500
Nipponbare	nipponbare_boundary_197	nipponbare_chr01	38962500	38967500
Nipponbare	nipponbare_boundary_198	nipponbare_chr01	39057500	39137500
Nipponbare	nipponbare_boundary_199	nipponbare_chr01	39297500	39302500
Nipponbare	nipponbare_boundary_200	nipponbare_chr01	39497500	39542500
Nipponbare	nipponbare_boundary_201	nipponbare_chr01	39582500	39587500
Nipponbare	nipponbare_boundary_202	nipponbare_chr01	39682500	39687500
Nipponbare	nipponbare_boundary_203	nipponbare_chr01	39765000	39770000
Nipponbare	nipponbare_boundary_204	nipponbare_chr01	39817500	39857500
Nipponbare	nipponbare_boundary_205	nipponbare_chr01	39952500	39957500
Nipponbare	nipponbare_boundary_206	nipponbare_chr01	40012500	40017500
Nipponbare	nipponbare_boundary_207	nipponbare_chr01	40062500	40067500
Nipponbare	nipponbare_boundary_208	nipponbare_chr01	40147500	40192500
Nipponbare	nipponbare_boundary_209	nipponbare_chr01	40237500	40282500
Nipponbare	nipponbare_boundary_210	nipponbare_chr01	40337500	40342500
Nipponbare	nipponbare_boundary_211	nipponbare_chr01	40432500	40437500
Nipponbare	nipponbare_boundary_212	nipponbare_chr01	40497500	40502500
Nipponbare	nipponbare_boundary_213	nipponbare_chr01	40552500	40557500
Nipponbare	nipponbare_boundary_214	nipponbare_chr01	40677500	40742500
Nipponbare	nipponbare_boundary_215	nipponbare_chr01	40797500	40832500
Nipponbare	nipponbare_boundary_216	nipponbare_chr01	40922500	40927500
Nipponbare	nipponbare_boundary_217	nipponbare_chr01	40997500	41002500
Nipponbare	nipponbare_boundary_218	nipponbare_chr01	41057500	41062500
Nipponbare	nipponbare_boundary_219	nipponbare_chr01	41917500	41922500
Nipponbare	nipponbare_boundary_220	nipponbare_chr01	41982500	42027500

Nipponbare	nipponbare_boundary_221	nipponbare_chr01	42072500	42077500
Nipponbare	nipponbare_boundary_222	nipponbare_chr01	42187500	42192500
Nipponbare	nipponbare_boundary_223	nipponbare_chr01	42237500	42242500
Nipponbare	nipponbare_boundary_224	nipponbare_chr01	42347500	42352500
Nipponbare	nipponbare_boundary_225	nipponbare_chr01	42452500	42457500
Nipponbare	nipponbare_boundary_226	nipponbare_chr01	42557500	42562500
Nipponbare	nipponbare_boundary_227	nipponbare_chr01	42622500	42627500
Nipponbare	nipponbare_boundary_228	nipponbare_chr01	42712500	42717500
Nipponbare	nipponbare_boundary_229	nipponbare_chr02	182500	187500
Nipponbare	nipponbare_boundary_230	nipponbare_chr02	297500	302500
Nipponbare	nipponbare_boundary_231	nipponbare_chr02	642500	647500
Nipponbare	nipponbare_boundary_232	nipponbare_chr02	737500	742500
Nipponbare	nipponbare_boundary_233	nipponbare_chr02	782500	787500
Nipponbare	nipponbare_boundary_234	nipponbare_chr02	877500	882500
Nipponbare	nipponbare_boundary_235	nipponbare_chr02	1187500	1192500
Nipponbare	nipponbare_boundary_236	nipponbare_chr02	1232500	1237500
Nipponbare	nipponbare_boundary_237	nipponbare_chr02	1442500	1487500
Nipponbare	nipponbare_boundary_238	nipponbare_chr02	1542500	1547500
Nipponbare	nipponbare_boundary_239	nipponbare_chr02	1602500	1647500
Nipponbare	nipponbare_boundary_240	nipponbare_chr02	1727500	1732500
Nipponbare	nipponbare_boundary_241	nipponbare_chr02	2237500	2242500
Nipponbare	nipponbare_boundary_242	nipponbare_chr02	2327500	2332500
Nipponbare	nipponbare_boundary_243	nipponbare_chr02	3122500	3127500
Nipponbare	nipponbare_boundary_244	nipponbare_chr02	3192500	3237500
Nipponbare	nipponbare_boundary_245	nipponbare_chr02	3357500	3362500
Nipponbare	nipponbare_boundary_246	nipponbare_chr02	3457500	3462500
Nipponbare	nipponbare_boundary_247	nipponbare_chr02	3522500	3557500
Nipponbare	nipponbare_boundary_248	nipponbare_chr02	3617500	3622500
Nipponbare	nipponbare_boundary_249	nipponbare_chr02	4137500	4142500
Nipponbare	nipponbare_boundary_250	nipponbare_chr02	4182500	4187500
Nipponbare	nipponbare_boundary_251	nipponbare_chr02	4267500	4272500
Nipponbare	nipponbare_boundary_252	nipponbare_chr02	4327500	4332500

Nipponbare	nipponbare_boundary_253	nipponbare_chr02	4642500	4647500
Nipponbare	nipponbare_boundary_254	nipponbare_chr02	4722500	4807500
Nipponbare	nipponbare_boundary_255	nipponbare_chr02	4857500	4862500
Nipponbare	nipponbare_boundary_256	nipponbare_chr02	4942500	4987500
Nipponbare	nipponbare_boundary_257	nipponbare_chr02	5052500	5057500
Nipponbare	nipponbare_boundary_258	nipponbare_chr02	5247500	5252500
Nipponbare	nipponbare_boundary_259	nipponbare_chr02	5327500	5332500
Nipponbare	nipponbare_boundary_260	nipponbare_chr02	5667500	5702500
Nipponbare	nipponbare_boundary_261	nipponbare_chr02	5747500	5752500
Nipponbare	nipponbare_boundary_262	nipponbare_chr02	5822500	5827500
Nipponbare	nipponbare_boundary_263	nipponbare_chr02	5912500	5917500
Nipponbare	nipponbare_boundary_264	nipponbare_chr02	6062500	6067500
Nipponbare	nipponbare_boundary_265	nipponbare_chr02	6127500	6132500
Nipponbare	nipponbare_boundary_266	nipponbare_chr02	6567500	6572500
Nipponbare	nipponbare_boundary_267	nipponbare_chr02	6672500	6677500
Nipponbare	nipponbare_boundary_268	nipponbare_chr02	6717500	6722500
Nipponbare	nipponbare_boundary_269	nipponbare_chr02	6787500	6792500
Nipponbare	nipponbare_boundary_270	nipponbare_chr02	7512500	7542500
Nipponbare	nipponbare_boundary_271	nipponbare_chr02	8690000	8727500
Nipponbare	nipponbare_boundary_272	nipponbare_chr02	8767500	8772500
Nipponbare	nipponbare_boundary_273	nipponbare_chr02	8822500	8827500
Nipponbare	nipponbare_boundary_274	nipponbare_chr02	8872500	8877500
Nipponbare	nipponbare_boundary_275	nipponbare_chr02	8972500	9057500
Nipponbare	nipponbare_boundary_276	nipponbare_chr02	9957500	9962500
Nipponbare	nipponbare_boundary_277	nipponbare_chr02	10047500	10087500
Nipponbare	nipponbare_boundary_278	nipponbare_chr02	10157500	10162500
Nipponbare	nipponbare_boundary_279	nipponbare_chr02	10207500	10212500
Nipponbare	nipponbare_boundary_280	nipponbare_chr02	10682500	10687500
Nipponbare	nipponbare_boundary_281	nipponbare_chr02	10762500	10767500
Nipponbare	nipponbare_boundary_282	nipponbare_chr02	10852500	10897500
Nipponbare	nipponbare_boundary_283	nipponbare_chr02	10942500	10947500
Nipponbare	nipponbare_boundary_284	nipponbare_chr02	11577500	11582500

Nipponbare	nipponbare_boundary_285	nipponbare_chr02	11667500	11672500
Nipponbare	nipponbare_boundary_286	nipponbare_chr02	12997500	13002500
Nipponbare	nipponbare_boundary_287	nipponbare_chr02	13062500	13067500
Nipponbare	nipponbare_boundary_288	nipponbare_chr02	13122500	13127500
Nipponbare	nipponbare_boundary_289	nipponbare_chr02	13317500	13357500
Nipponbare	nipponbare_boundary_290	nipponbare_chr02	13407500	13412500
Nipponbare	nipponbare_boundary_291	nipponbare_chr02	13565000	13570000
Nipponbare	nipponbare_boundary_292	nipponbare_chr02	15442500	15447500
Nipponbare	nipponbare_boundary_293	nipponbare_chr02	15522500	15527500
Nipponbare	nipponbare_boundary_294	nipponbare_chr02	15577500	15582500
Nipponbare	nipponbare_boundary_295	nipponbare_chr02	15622500	15627500
Nipponbare	nipponbare_boundary_296	nipponbare_chr02	15927500	15932500
Nipponbare	nipponbare_boundary_297	nipponbare_chr02	15992500	15997500
Nipponbare	nipponbare_boundary_298	nipponbare_chr02	16127500	16132500
Nipponbare	nipponbare_boundary_299	nipponbare_chr02	16182500	16187500
Nipponbare	nipponbare_boundary_300	nipponbare_chr02	16262500	16267500
Nipponbare	nipponbare_boundary_301	nipponbare_chr02	16342500	16347500
Nipponbare	nipponbare_boundary_302	nipponbare_chr02	16447500	16452500
Nipponbare	nipponbare_boundary_303	nipponbare_chr02	17032500	17037500
Nipponbare	nipponbare_boundary_304	nipponbare_chr02	17092500	17132500
Nipponbare	nipponbare_boundary_305	nipponbare_chr02	17337500	17342500
Nipponbare	nipponbare_boundary_306	nipponbare_chr02	17407500	17412500
Nipponbare	nipponbare_boundary_307	nipponbare_chr02	17462500	17467500
Nipponbare	nipponbare_boundary_308	nipponbare_chr02	17517500	17522500
Nipponbare	nipponbare_boundary_309	nipponbare_chr02	17920000	17925000
Nipponbare	nipponbare_boundary_310	nipponbare_chr02	17987500	17992500
Nipponbare	nipponbare_boundary_311	nipponbare_chr02	18312500	18317500
Nipponbare	nipponbare_boundary_312	nipponbare_chr02	18377500	18382500
Nipponbare	nipponbare_boundary_313	nipponbare_chr02	18652500	18657500
Nipponbare	nipponbare_boundary_314	nipponbare_chr02	18712500	18717500
Nipponbare	nipponbare_boundary_315	nipponbare_chr02	18777500	18782500
Nipponbare	nipponbare_boundary_316	nipponbare_chr02	18842500	18847500

Nipponbare	nipponbare_boundary_317	nipponbare_chr02	18927500	18932500
Nipponbare	nipponbare_boundary_318	nipponbare_chr02	18982500	18987500
Nipponbare	nipponbare_boundary_319	nipponbare_chr02	19032500	19037500
Nipponbare	nipponbare_boundary_320	nipponbare_chr02	19092500	19132500
Nipponbare	nipponbare_boundary_321	nipponbare_chr02	19177500	19217500
Nipponbare	nipponbare_boundary_322	nipponbare_chr02	19592500	19657500
Nipponbare	nipponbare_boundary_323	nipponbare_chr02	19702500	19707500
Nipponbare	nipponbare_boundary_324	nipponbare_chr02	19782500	19787500
Nipponbare	nipponbare_boundary_325	nipponbare_chr02	19957500	19962500
Nipponbare	nipponbare_boundary_326	nipponbare_chr02	20032500	20037500
Nipponbare	nipponbare_boundary_327	nipponbare_chr02	20732500	20737500
Nipponbare	nipponbare_boundary_328	nipponbare_chr02	20832500	20872500
Nipponbare	nipponbare_boundary_329	nipponbare_chr02	21112500	21117500
Nipponbare	nipponbare_boundary_330	nipponbare_chr02	21162500	21167500
Nipponbare	nipponbare_boundary_331	nipponbare_chr02	21307500	21312500
Nipponbare	nipponbare_boundary_332	nipponbare_chr02	21412500	21417500
Nipponbare	nipponbare_boundary_333	nipponbare_chr02	21642500	21647500
Nipponbare	nipponbare_boundary_334	nipponbare_chr02	21772500	21777500
Nipponbare	nipponbare_boundary_335	nipponbare_chr02	21817500	21822500
Nipponbare	nipponbare_boundary_336	nipponbare_chr02	21872500	21877500
Nipponbare	nipponbare_boundary_337	nipponbare_chr02	21917500	21952500
Nipponbare	nipponbare_boundary_338	nipponbare_chr02	22072500	22077500
Nipponbare	nipponbare_boundary_339	nipponbare_chr02	22167500	22172500
Nipponbare	nipponbare_boundary_340	nipponbare_chr02	22412500	22417500
Nipponbare	nipponbare_boundary_341	nipponbare_chr02	22477500	22482500
Nipponbare	nipponbare_boundary_342	nipponbare_chr02	22532500	22577500
Nipponbare	nipponbare_boundary_343	nipponbare_chr02	22882500	22887500
Nipponbare	nipponbare_boundary_344	nipponbare_chr02	22932500	22937500
Nipponbare	nipponbare_boundary_345	nipponbare_chr02	22977500	22982500
Nipponbare	nipponbare_boundary_346	nipponbare_chr02	23037500	23107500
Nipponbare	nipponbare_boundary_347	nipponbare_chr02	23222500	23227500
Nipponbare	nipponbare_boundary_348	nipponbare_chr02	23457500	23462500

Nipponbare	nipponbare_boundary_349	nipponbare_chr02	23527500	23532500
Nipponbare	nipponbare_boundary_350	nipponbare_chr02	23577500	23582500
Nipponbare	nipponbare_boundary_351	nipponbare_chr02	23657500	23662500
Nipponbare	nipponbare_boundary_352	nipponbare_chr02	23782500	23787500
Nipponbare	nipponbare_boundary_353	nipponbare_chr02	23842500	23847500
Nipponbare	nipponbare_boundary_354	nipponbare_chr02	23907500	23912500
Nipponbare	nipponbare_boundary_355	nipponbare_chr02	23972500	24007500
Nipponbare	nipponbare_boundary_356	nipponbare_chr02	24097500	24137500
Nipponbare	nipponbare_boundary_357	nipponbare_chr02	24377500	24382500
Nipponbare	nipponbare_boundary_358	nipponbare_chr02	24502500	24507500
Nipponbare	nipponbare_boundary_359	nipponbare_chr02	24732500	24737500
Nipponbare	nipponbare_boundary_360	nipponbare_chr02	24782500	24787500
Nipponbare	nipponbare_boundary_361	nipponbare_chr02	24867500	24872500
Nipponbare	nipponbare_boundary_362	nipponbare_chr02	25192500	25232500
Nipponbare	nipponbare_boundary_363	nipponbare_chr02	25282500	25287500
Nipponbare	nipponbare_boundary_364	nipponbare_chr02	25352500	25357500
Nipponbare	nipponbare_boundary_365	nipponbare_chr02	25577500	25582500
Nipponbare	nipponbare_boundary_366	nipponbare_chr02	25657500	25662500
Nipponbare	nipponbare_boundary_367	nipponbare_chr02	26012500	26052500
Nipponbare	nipponbare_boundary_368	nipponbare_chr02	26107500	26112500
Nipponbare	nipponbare_boundary_369	nipponbare_chr02	26242500	26282500
Nipponbare	nipponbare_boundary_370	nipponbare_chr02	26337500	26342500
Nipponbare	nipponbare_boundary_371	nipponbare_chr02	26387500	26392500
Nipponbare	nipponbare_boundary_372	nipponbare_chr02	26667500	26672500
Nipponbare	nipponbare_boundary_373	nipponbare_chr02	26777500	26782500
Nipponbare	nipponbare_boundary_374	nipponbare_chr02	26922500	26927500
Nipponbare	nipponbare_boundary_375	nipponbare_chr02	26982500	26987500
Nipponbare	nipponbare_boundary_376	nipponbare_chr02	27027500	27032500
Nipponbare	nipponbare_boundary_377	nipponbare_chr02	27267500	27272500
Nipponbare	nipponbare_boundary_378	nipponbare_chr02	27357500	27362500
Nipponbare	nipponbare_boundary_379	nipponbare_chr02	27737500	27742500
Nipponbare	nipponbare_boundary_380	nipponbare_chr02	27832500	27837500

Nipponbare	nipponbare_boundary_381	nipponbare_chr02	27912500	27917500
Nipponbare	nipponbare_boundary_382	nipponbare_chr02	27957500	27962500
Nipponbare	nipponbare_boundary_383	nipponbare_chr02	28262500	28267500
Nipponbare	nipponbare_boundary_384	nipponbare_chr02	28332500	28337500
Nipponbare	nipponbare_boundary_385	nipponbare_chr02	28422500	28427500
Nipponbare	nipponbare_boundary_386	nipponbare_chr02	28667500	28707500
Nipponbare	nipponbare_boundary_387	nipponbare_chr02	28797500	28802500
Nipponbare	nipponbare_boundary_388	nipponbare_chr02	29292500	29297500
Nipponbare	nipponbare_boundary_389	nipponbare_chr02	29337500	29342500
Nipponbare	nipponbare_boundary_390	nipponbare_chr02	29492500	29497500
Nipponbare	nipponbare_boundary_391	nipponbare_chr02	29612500	29617500
Nipponbare	nipponbare_boundary_392	nipponbare_chr02	29672500	29677500
Nipponbare	nipponbare_boundary_393	nipponbare_chr02	29717500	29752500
Nipponbare	nipponbare_boundary_394	nipponbare_chr02	29792500	29832500
Nipponbare	nipponbare_boundary_395	nipponbare_chr02	30272500	30277500
Nipponbare	nipponbare_boundary_396	nipponbare_chr02	30352500	30392500
Nipponbare	nipponbare_boundary_397	nipponbare_chr02	30562500	30567500
Nipponbare	nipponbare_boundary_398	nipponbare_chr02	30652500	30657500
Nipponbare	nipponbare_boundary_399	nipponbare_chr02	30867500	30872500
Nipponbare	nipponbare_boundary_400	nipponbare_chr02	30927500	30967500
Nipponbare	nipponbare_boundary_401	nipponbare_chr02	31412500	31417500
Nipponbare	nipponbare_boundary_402	nipponbare_chr02	31467500	31472500
Nipponbare	nipponbare_boundary_403	nipponbare_chr02	31527500	31532500
Nipponbare	nipponbare_boundary_404	nipponbare_chr02	31607500	31612500
Nipponbare	nipponbare_boundary_405	nipponbare_chr02	32582500	32587500
Nipponbare	nipponbare_boundary_406	nipponbare_chr02	32697500	32702500
Nipponbare	nipponbare_boundary_407	nipponbare_chr02	32832500	32837500
Nipponbare	nipponbare_boundary_408	nipponbare_chr02	32877500	32882500
Nipponbare	nipponbare_boundary_409	nipponbare_chr02	32932500	32937500
Nipponbare	nipponbare_boundary_410	nipponbare_chr02	32992500	32997500
Nipponbare	nipponbare_boundary_411	nipponbare_chr02	33162500	33167500
Nipponbare	nipponbare_boundary_412	nipponbare_chr02	33227500	33232500

Nipponbare	nipponbare_boundary_413	nipponbare_chr02	33292500	33297500
Nipponbare	nipponbare_boundary_414	nipponbare_chr02	33357500	33362500
Nipponbare	nipponbare_boundary_415	nipponbare_chr02	33427500	33432500
Nipponbare	nipponbare_boundary_416	nipponbare_chr02	33487500	33492500
Nipponbare	nipponbare_boundary_417	nipponbare_chr02	33542500	33547500
Nipponbare	nipponbare_boundary_418	nipponbare_chr02	33597500	33637500
Nipponbare	nipponbare_boundary_419	nipponbare_chr02	33742500	33747500
Nipponbare	nipponbare_boundary_420	nipponbare_chr02	33842500	33847500
Nipponbare	nipponbare_boundary_421	nipponbare_chr02	33997500	34042500
Nipponbare	nipponbare_boundary_422	nipponbare_chr02	34512500	34517500
Nipponbare	nipponbare_boundary_423	nipponbare_chr02	34587500	34592500
Nipponbare	nipponbare_boundary_424	nipponbare_chr02	35257500	35262500
Nipponbare	nipponbare_boundary_425	nipponbare_chr02	35372500	35377500
Nipponbare	nipponbare_boundary_426	nipponbare_chr02	35677500	35682500
Nipponbare	nipponbare_boundary_427	nipponbare_chr02	35742500	35747500
Nipponbare	nipponbare_boundary_428	nipponbare_chr03	647500	652500
Nipponbare	nipponbare_boundary_429	nipponbare_chr03	727500	732500
Nipponbare	nipponbare_boundary_430	nipponbare_chr03	792500	797500
Nipponbare	nipponbare_boundary_431	nipponbare_chr03	847500	852500
Nipponbare	nipponbare_boundary_432	nipponbare_chr03	902500	907500
Nipponbare	nipponbare_boundary_433	nipponbare_chr03	1132500	1137500
Nipponbare	nipponbare_boundary_434	nipponbare_chr03	1177500	1182500
Nipponbare	nipponbare_boundary_435	nipponbare_chr03	1432500	1437500
Nipponbare	nipponbare_boundary_436	nipponbare_chr03	1507500	1512500
Nipponbare	nipponbare_boundary_437	nipponbare_chr03	1572500	1577500
Nipponbare	nipponbare_boundary_438	nipponbare_chr03	1887500	1892500
Nipponbare	nipponbare_boundary_439	nipponbare_chr03	1957500	1962500
Nipponbare	nipponbare_boundary_440	nipponbare_chr03	2012500	2017500
Nipponbare	nipponbare_boundary_441	nipponbare_chr03	2342500	2387500
Nipponbare	nipponbare_boundary_442	nipponbare_chr03	2827500	2857500
Nipponbare	nipponbare_boundary_443	nipponbare_chr03	2927500	2932500
Nipponbare	nipponbare_boundary_444	nipponbare_chr03	2977500	2982500

Nipponbare	nipponbare_boundary_445	nipponbare_chr03	3112500	3117500
Nipponbare	nipponbare_boundary_446	nipponbare_chr03	3182500	3222500
Nipponbare	nipponbare_boundary_447	nipponbare_chr03	3312500	3347500
Nipponbare	nipponbare_boundary_448	nipponbare_chr03	4077500	4082500
Nipponbare	nipponbare_boundary_449	nipponbare_chr03	4142500	4147500
Nipponbare	nipponbare_boundary_450	nipponbare_chr03	4187500	4227500
Nipponbare	nipponbare_boundary_451	nipponbare_chr03	4387500	4392500
Nipponbare	nipponbare_boundary_452	nipponbare_chr03	4432500	4437500
Nipponbare	nipponbare_boundary_453	nipponbare_chr03	4487500	4492500
Nipponbare	nipponbare_boundary_454	nipponbare_chr03	4592500	4702500
Nipponbare	nipponbare_boundary_455	nipponbare_chr03	4772500	4812500
Nipponbare	nipponbare_boundary_456	nipponbare_chr03	4942500	4947500
Nipponbare	nipponbare_boundary_457	nipponbare_chr03	4992500	4997500
Nipponbare	nipponbare_boundary_458	nipponbare_chr03	5057500	5092500
Nipponbare	nipponbare_boundary_459	nipponbare_chr03	5162500	5202500
Nipponbare	nipponbare_boundary_460	nipponbare_chr03	5272500	5307500
Nipponbare	nipponbare_boundary_461	nipponbare_chr03	5617500	5622500
Nipponbare	nipponbare_boundary_462	nipponbare_chr03	5682500	5687500
Nipponbare	nipponbare_boundary_463	nipponbare_chr03	5767500	5772500
Nipponbare	nipponbare_boundary_464	nipponbare_chr03	5817500	5822500
Nipponbare	nipponbare_boundary_465	nipponbare_chr03	5862500	5897500
Nipponbare	nipponbare_boundary_466	nipponbare_chr03	5942500	5947500
Nipponbare	nipponbare_boundary_467	nipponbare_chr03	6362500	6367500
Nipponbare	nipponbare_boundary_468	nipponbare_chr03	6407500	6412500
Nipponbare	nipponbare_boundary_469	nipponbare_chr03	6812500	6817500
Nipponbare	nipponbare_boundary_470	nipponbare_chr03	6867500	6872500
Nipponbare	nipponbare_boundary_471	nipponbare_chr03	6987500	7017500
Nipponbare	nipponbare_boundary_472	nipponbare_chr03	7062500	7067500
Nipponbare	nipponbare_boundary_473	nipponbare_chr03	7132500	7172500
Nipponbare	nipponbare_boundary_474	nipponbare_chr03	8172500	8217500
Nipponbare	nipponbare_boundary_475	nipponbare_chr03	8397500	8402500
Nipponbare	nipponbare_boundary_476	nipponbare_chr03	8442500	8447500

Nipponbare	nipponbare_boundary_477	nipponbare_chr03	8497500	8502500
Nipponbare	nipponbare_boundary_478	nipponbare_chr03	8557500	8562500
Nipponbare	nipponbare_boundary_479	nipponbare_chr03	8662500	8667500
Nipponbare	nipponbare_boundary_480	nipponbare_chr03	8712500	8717500
Nipponbare	nipponbare_boundary_481	nipponbare_chr03	8787500	8792500
Nipponbare	nipponbare_boundary_482	nipponbare_chr03	8832500	8837500
Nipponbare	nipponbare_boundary_483	nipponbare_chr03	9082500	9087500
Nipponbare	nipponbare_boundary_484	nipponbare_chr03	9167500	9172500
Nipponbare	nipponbare_boundary_485	nipponbare_chr03	9217500	9262500
Nipponbare	nipponbare_boundary_486	nipponbare_chr03	9362500	9367500
Nipponbare	nipponbare_boundary_487	nipponbare_chr03	9417500	9422500
Nipponbare	nipponbare_boundary_488	nipponbare_chr03	9492500	9497500
Nipponbare	nipponbare_boundary_489	nipponbare_chr03	9552500	9557500
Nipponbare	nipponbare_boundary_490	nipponbare_chr03	9697500	9702500
Nipponbare	nipponbare_boundary_491	nipponbare_chr03	9752500	9757500
Nipponbare	nipponbare_boundary_492	nipponbare_chr03	9847500	9882500
Nipponbare	nipponbare_boundary_493	nipponbare_chr03	10397500	10437500
Nipponbare	nipponbare_boundary_494	nipponbare_chr03	10987500	10992500
Nipponbare	nipponbare_boundary_495	nipponbare_chr03	11047500	11122500
Nipponbare	nipponbare_boundary_496	nipponbare_chr03	11167500	11172500
Nipponbare	nipponbare_boundary_497	nipponbare_chr03	11467500	11472500
Nipponbare	nipponbare_boundary_498	nipponbare_chr03	11522500	11527500
Nipponbare	nipponbare_boundary_499	nipponbare_chr03	11597500	11602500
Nipponbare	nipponbare_boundary_500	nipponbare_chr03	11647500	11652500
Nipponbare	nipponbare_boundary_501	nipponbare_chr03	11752500	11757500
Nipponbare	nipponbare_boundary_502	nipponbare_chr03	11807500	11842500
Nipponbare	nipponbare_boundary_503	nipponbare_chr03	11897500	11962500
Nipponbare	nipponbare_boundary_504	nipponbare_chr03	12022500	12027500
Nipponbare	nipponbare_boundary_505	nipponbare_chr03	12102500	12147500
Nipponbare	nipponbare_boundary_506	nipponbare_chr03	12277500	12282500
Nipponbare	nipponbare_boundary_507	nipponbare_chr03	12387500	12392500
Nipponbare	nipponbare_boundary_508	nipponbare_chr03	12442500	12447500

Nipponbare	nipponbare_boundary_509	nipponbare_chr03	12542500	12547500
Nipponbare	nipponbare_boundary_510	nipponbare_chr03	12607500	12612500
Nipponbare	nipponbare_boundary_511	nipponbare_chr03	12997500	13002500
Nipponbare	nipponbare_boundary_512	nipponbare_chr03	13097500	13102500
Nipponbare	nipponbare_boundary_513	nipponbare_chr03	13762500	13767500
Nipponbare	nipponbare_boundary_514	nipponbare_chr03	13837500	13842500
Nipponbare	nipponbare_boundary_515	nipponbare_chr03	14422500	14462500
Nipponbare	nipponbare_boundary_516	nipponbare_chr03	14507500	14512500
Nipponbare	nipponbare_boundary_517	nipponbare_chr03	14952500	14957500
Nipponbare	nipponbare_boundary_518	nipponbare_chr03	15002500	15007500
Nipponbare	nipponbare_boundary_519	nipponbare_chr03	15097500	15102500
Nipponbare	nipponbare_boundary_520	nipponbare_chr03	15577500	15582500
Nipponbare	nipponbare_boundary_521	nipponbare_chr03	15642500	15647500
Nipponbare	nipponbare_boundary_522	nipponbare_chr03	15707500	15712500
Nipponbare	nipponbare_boundary_523	nipponbare_chr03	15772500	15777500
Nipponbare	nipponbare_boundary_524	nipponbare_chr03	16972500	16977500
Nipponbare	nipponbare_boundary_525	nipponbare_chr03	17047500	17052500
Nipponbare	nipponbare_boundary_526	nipponbare_chr03	17282500	17287500
Nipponbare	nipponbare_boundary_527	nipponbare_chr03	17382500	17387500
Nipponbare	nipponbare_boundary_528	nipponbare_chr03	17717500	17722500
Nipponbare	nipponbare_boundary_529	nipponbare_chr03	17777500	17782500
Nipponbare	nipponbare_boundary_530	nipponbare_chr03	17917500	17922500
Nipponbare	nipponbare_boundary_531	nipponbare_chr03	18022500	18027500
Nipponbare	nipponbare_boundary_532	nipponbare_chr03	18117500	18122500
Nipponbare	nipponbare_boundary_533	nipponbare_chr03	18177500	18182500
Nipponbare	nipponbare_boundary_534	nipponbare_chr03	20687500	20692500
Nipponbare	nipponbare_boundary_535	nipponbare_chr03	20767500	20772500
Nipponbare	nipponbare_boundary_536	nipponbare_chr03	21622500	21627500
Nipponbare	nipponbare_boundary_537	nipponbare_chr03	21727500	21732500
Nipponbare	nipponbare_boundary_538	nipponbare_chr03	22167500	22172500
Nipponbare	nipponbare_boundary_539	nipponbare_chr03	22212500	22217500
Nipponbare	nipponbare_boundary_540	nipponbare_chr03	22287500	22292500

Nipponbare	nipponbare_boundary_541	nipponbare_chr03	22337500	22377500
Nipponbare	nipponbare_boundary_542	nipponbare_chr03	22417500	22422500
Nipponbare	nipponbare_boundary_543	nipponbare_chr03	22737500	22742500
Nipponbare	nipponbare_boundary_544	nipponbare_chr03	22812500	22817500
Nipponbare	nipponbare_boundary_545	nipponbare_chr03	23097500	23102500
Nipponbare	nipponbare_boundary_546	nipponbare_chr03	23170000	23175000
Nipponbare	nipponbare_boundary_547	nipponbare_chr03	23737500	23742500
Nipponbare	nipponbare_boundary_548	nipponbare_chr03	23792500	23797500
Nipponbare	nipponbare_boundary_549	nipponbare_chr03	23897500	23902500
Nipponbare	nipponbare_boundary_550	nipponbare_chr03	24167500	24172500
Nipponbare	nipponbare_boundary_551	nipponbare_chr03	24217500	24257500
Nipponbare	nipponbare_boundary_552	nipponbare_chr03	24307500	24312500
Nipponbare	nipponbare_boundary_553	nipponbare_chr03	25062500	25102500
Nipponbare	nipponbare_boundary_554	nipponbare_chr03	25297500	25337500
Nipponbare	nipponbare_boundary_555	nipponbare_chr03	25387500	25392500
Nipponbare	nipponbare_boundary_556	nipponbare_chr03	25507500	25512500
Nipponbare	nipponbare_boundary_557	nipponbare_chr03	25572500	25577500
Nipponbare	nipponbare_boundary_558	nipponbare_chr03	25622500	25627500
Nipponbare	nipponbare_boundary_559	nipponbare_chr03	25747500	25792500
Nipponbare	nipponbare_boundary_560	nipponbare_chr03	25847500	25852500
Nipponbare	nipponbare_boundary_561	nipponbare_chr03	25902500	25942500
Nipponbare	nipponbare_boundary_562	nipponbare_chr03	25987500	25992500
Nipponbare	nipponbare_boundary_563	nipponbare_chr03	26067500	26072500
Nipponbare	nipponbare_boundary_564	nipponbare_chr03	26117500	26122500
Nipponbare	nipponbare_boundary_565	nipponbare_chr03	26192500	26197500
Nipponbare	nipponbare_boundary_566	nipponbare_chr03	26462500	26467500
Nipponbare	nipponbare_boundary_567	nipponbare_chr03	26532500	26537500
Nipponbare	nipponbare_boundary_568	nipponbare_chr03	26597500	26627500
Nipponbare	nipponbare_boundary_569	nipponbare_chr03	26692500	26697500
Nipponbare	nipponbare_boundary_570	nipponbare_chr03	28157500	28192500
Nipponbare	nipponbare_boundary_571	nipponbare_chr03	28257500	28262500
Nipponbare	nipponbare_boundary_572	nipponbare_chr03	28312500	28317500

Nipponbare	nipponbare_boundary_573	nipponbare_chr03	28372500	28402500
Nipponbare	nipponbare_boundary_574	nipponbare_chr03	28952500	28957500
Nipponbare	nipponbare_boundary_575	nipponbare_chr03	28997500	29002500
Nipponbare	nipponbare_boundary_576	nipponbare_chr03	29057500	29062500
Nipponbare	nipponbare_boundary_577	nipponbare_chr03	29522500	29587500
Nipponbare	nipponbare_boundary_578	nipponbare_chr03	29782500	29787500
Nipponbare	nipponbare_boundary_579	nipponbare_chr03	29832500	29837500
Nipponbare	nipponbare_boundary_580	nipponbare_chr03	30192500	30235000
Nipponbare	nipponbare_boundary_581	nipponbare_chr03	30317500	30387500
Nipponbare	nipponbare_boundary_582	nipponbare_chr03	30557500	30562500
Nipponbare	nipponbare_boundary_583	nipponbare_chr03	30602500	30607500
Nipponbare	nipponbare_boundary_584	nipponbare_chr03	30687500	30692500
Nipponbare	nipponbare_boundary_585	nipponbare_chr03	30757500	30762500
Nipponbare	nipponbare_boundary_586	nipponbare_chr03	30812500	30817500
Nipponbare	nipponbare_boundary_587	nipponbare_chr03	30897500	30902500
Nipponbare	nipponbare_boundary_588	nipponbare_chr03	31110000	31115000
Nipponbare	nipponbare_boundary_589	nipponbare_chr03	31202500	31207500
Nipponbare	nipponbare_boundary_590	nipponbare_chr03	31302500	31342500
Nipponbare	nipponbare_boundary_591	nipponbare_chr03	31387500	31462500
Nipponbare	nipponbare_boundary_592	nipponbare_chr03	31562500	31607500
Nipponbare	nipponbare_boundary_593	nipponbare_chr03	31672500	31677500
Nipponbare	nipponbare_boundary_594	nipponbare_chr03	31727500	31732500
Nipponbare	nipponbare_boundary_595	nipponbare_chr03	31882500	31887500
Nipponbare	nipponbare_boundary_596	nipponbare_chr03	31952500	31957500
Nipponbare	nipponbare_boundary_597	nipponbare_chr03	32012500	32017500
Nipponbare	nipponbare_boundary_598	nipponbare_chr03	32552500	32557500
Nipponbare	nipponbare_boundary_599	nipponbare_chr03	32622500	32667500
Nipponbare	nipponbare_boundary_600	nipponbare_chr03	33062500	33067500
Nipponbare	nipponbare_boundary_601	nipponbare_chr03	33112500	33117500
Nipponbare	nipponbare_boundary_602	nipponbare_chr03	33197500	33202500
Nipponbare	nipponbare_boundary_603	nipponbare_chr03	33367500	33372500
Nipponbare	nipponbare_boundary_604	nipponbare_chr03	33437500	33442500

Nipponbare	nipponbare_boundary_605	nipponbare_chr03	33517500	33522500
Nipponbare	nipponbare_boundary_606	nipponbare_chr03	33602500	33607500
Nipponbare	nipponbare_boundary_607	nipponbare_chr03	33687500	33692500
Nipponbare	nipponbare_boundary_608	nipponbare_chr03	33737500	33772500
Nipponbare	nipponbare_boundary_609	nipponbare_chr03	33847500	33852500
Nipponbare	nipponbare_boundary_610	nipponbare_chr03	33932500	33937500
Nipponbare	nipponbare_boundary_611	nipponbare_chr03	34002500	34047500
Nipponbare	nipponbare_boundary_612	nipponbare_chr03	34172500	34177500
Nipponbare	nipponbare_boundary_613	nipponbare_chr03	34222500	34227500
Nipponbare	nipponbare_boundary_614	nipponbare_chr03	34332500	34337500
Nipponbare	nipponbare_boundary_615	nipponbare_chr03	34392500	34397500
Nipponbare	nipponbare_boundary_616	nipponbare_chr03	34442500	34447500
Nipponbare	nipponbare_boundary_617	nipponbare_chr03	34532500	34537500
Nipponbare	nipponbare_boundary_618	nipponbare_chr03	34617500	34622500
Nipponbare	nipponbare_boundary_619	nipponbare_chr03	34677500	34682500
Nipponbare	nipponbare_boundary_620	nipponbare_chr03	34722500	34727500
Nipponbare	nipponbare_boundary_621	nipponbare_chr03	35662500	35702500
Nipponbare	nipponbare_boundary_622	nipponbare_chr03	35767500	35772500
Nipponbare	nipponbare_boundary_623	nipponbare_chr03	35817500	35822500
Nipponbare	nipponbare_boundary_624	nipponbare_chr03	35902500	35907500
Nipponbare	nipponbare_boundary_625	nipponbare_chr03	35967500	35972500
Nipponbare	nipponbare_boundary_626	nipponbare_chr03	36072500	36112500
Nipponbare	nipponbare_boundary_627	nipponbare_chr03	36177500	36182500
Nipponbare	nipponbare_boundary_628	nipponbare_chr04	612500	617500
Nipponbare	nipponbare_boundary_629	nipponbare_chr04	712500	717500
Nipponbare	nipponbare_boundary_630	nipponbare_chr04	875000	917500
Nipponbare	nipponbare_boundary_631	nipponbare_chr04	2017500	2022500
Nipponbare	nipponbare_boundary_632	nipponbare_chr04	2257500	2262500
Nipponbare	nipponbare_boundary_633	nipponbare_chr04	2417500	2422500
Nipponbare	nipponbare_boundary_634	nipponbare_chr04	2487500	2492500
Nipponbare	nipponbare_boundary_635	nipponbare_chr04	4402500	4447500
Nipponbare	nipponbare_boundary_636	nipponbare_chr04	4502500	4507500

Nipponbare	nipponbare_boundary_637	nipponbare_chr04	4552500	4557500
Nipponbare	nipponbare_boundary_638	nipponbare_chr04	4697500	4702500
Nipponbare	nipponbare_boundary_639	nipponbare_chr04	4917500	4922500
Nipponbare	nipponbare_boundary_640	nipponbare_chr04	4972500	4977500
Nipponbare	nipponbare_boundary_641	nipponbare_chr04	5112500	5117500
Nipponbare	nipponbare_boundary_642	nipponbare_chr04	5162500	5167500
Nipponbare	nipponbare_boundary_643	nipponbare_chr04	5302500	5307500
Nipponbare	nipponbare_boundary_644	nipponbare_chr04	5522500	5527500
Nipponbare	nipponbare_boundary_645	nipponbare_chr04	5587500	5592500
Nipponbare	nipponbare_boundary_646	nipponbare_chr04	5662500	5667500
Nipponbare	nipponbare_boundary_647	nipponbare_chr04	6892500	6897500
Nipponbare	nipponbare_boundary_648	nipponbare_chr04	6952500	6957500
Nipponbare	nipponbare_boundary_649	nipponbare_chr04	6997500	7002500
Nipponbare	nipponbare_boundary_650	nipponbare_chr04	7122500	7127500
Nipponbare	nipponbare_boundary_651	nipponbare_chr04	7167500	7172500
Nipponbare	nipponbare_boundary_652	nipponbare_chr04	7252500	7257500
Nipponbare	nipponbare_boundary_653	nipponbare_chr04	7382500	7387500
Nipponbare	nipponbare_boundary_654	nipponbare_chr04	7537500	7542500
Nipponbare	nipponbare_boundary_655	nipponbare_chr04	7827500	7832500
Nipponbare	nipponbare_boundary_656	nipponbare_chr04	8122500	8127500
Nipponbare	nipponbare_boundary_657	nipponbare_chr04	8197500	8202500
Nipponbare	nipponbare_boundary_658	nipponbare_chr04	8272500	8277500
Nipponbare	nipponbare_boundary_659	nipponbare_chr04	12397500	12402500
Nipponbare	nipponbare_boundary_660	nipponbare_chr04	12447500	12452500
Nipponbare	nipponbare_boundary_661	nipponbare_chr04	12527500	12532500
Nipponbare	nipponbare_boundary_662	nipponbare_chr04	12732500	12737500
Nipponbare	nipponbare_boundary_663	nipponbare_chr04	12852500	12857500
Nipponbare	nipponbare_boundary_664	nipponbare_chr04	12957500	12962500
Nipponbare	nipponbare_boundary_665	nipponbare_chr04	13017500	13022500
Nipponbare	nipponbare_boundary_666	nipponbare_chr04	14077500	14082500
Nipponbare	nipponbare_boundary_667	nipponbare_chr04	14157500	14162500
Nipponbare	nipponbare_boundary_668	nipponbare_chr04	16402500	16407500

Nipponbare	nipponbare_boundary_669	nipponbare_chr04	16492500	16497500
Nipponbare	nipponbare_boundary_670	nipponbare_chr04	16542500	16547500
Nipponbare	nipponbare_boundary_671	nipponbare_chr04	16587500	16592500
Nipponbare	nipponbare_boundary_672	nipponbare_chr04	16642500	16647500
Nipponbare	nipponbare_boundary_673	nipponbare_chr04	16697500	16702500
Nipponbare	nipponbare_boundary_674	nipponbare_chr04	16817500	16822500
Nipponbare	nipponbare_boundary_675	nipponbare_chr04	18460000	18465000
Nipponbare	nipponbare_boundary_676	nipponbare_chr04	18552500	18557500
Nipponbare	nipponbare_boundary_677	nipponbare_chr04	19132500	19137500
Nipponbare	nipponbare_boundary_678	nipponbare_chr04	19182500	19187500
Nipponbare	nipponbare_boundary_679	nipponbare_chr04	19402500	19407500
Nipponbare	nipponbare_boundary_680	nipponbare_chr04	19502500	19507500
Nipponbare	nipponbare_boundary_681	nipponbare_chr04	19597500	19602500
Nipponbare	nipponbare_boundary_682	nipponbare_chr04	19797500	19802500
Nipponbare	nipponbare_boundary_683	nipponbare_chr04	19932500	19937500
Nipponbare	nipponbare_boundary_684	nipponbare_chr04	20007500	20012500
Nipponbare	nipponbare_boundary_685	nipponbare_chr04	20062500	20067500
Nipponbare	nipponbare_boundary_686	nipponbare_chr04	20117500	20122500
Nipponbare	nipponbare_boundary_687	nipponbare_chr04	20237500	20282500
Nipponbare	nipponbare_boundary_688	nipponbare_chr04	20842500	20882500
Nipponbare	nipponbare_boundary_689	nipponbare_chr04	21227500	21262500
Nipponbare	nipponbare_boundary_690	nipponbare_chr04	21382500	21387500
Nipponbare	nipponbare_boundary_691	nipponbare_chr04	21442500	21447500
Nipponbare	nipponbare_boundary_692	nipponbare_chr04	21592500	21597500
Nipponbare	nipponbare_boundary_693	nipponbare_chr04	21642500	21647500
Nipponbare	nipponbare_boundary_694	nipponbare_chr04	21697500	21702500
Nipponbare	nipponbare_boundary_695	nipponbare_chr04	21902500	21907500
Nipponbare	nipponbare_boundary_696	nipponbare_chr04	21992500	21997500
Nipponbare	nipponbare_boundary_697	nipponbare_chr04	22362500	22367500
Nipponbare	nipponbare_boundary_698	nipponbare_chr04	22412500	22417500
Nipponbare	nipponbare_boundary_699	nipponbare_chr04	23097500	23102500
Nipponbare	nipponbare_boundary_700	nipponbare_chr04	23142500	23147500

Nipponbare	nipponbare_boundary_701	nipponbare_chr04	23242500	23247500
Nipponbare	nipponbare_boundary_702	nipponbare_chr04	23847500	23852500
Nipponbare	nipponbare_boundary_703	nipponbare_chr04	23967500	23972500
Nipponbare	nipponbare_boundary_704	nipponbare_chr04	24017500	24022500
Nipponbare	nipponbare_boundary_705	nipponbare_chr04	24072500	24077500
Nipponbare	nipponbare_boundary_706	nipponbare_chr04	24132500	24137500
Nipponbare	nipponbare_boundary_707	nipponbare_chr04	24477500	24482500
Nipponbare	nipponbare_boundary_708	nipponbare_chr04	24532500	24537500
Nipponbare	nipponbare_boundary_709	nipponbare_chr04	24642500	24647500
Nipponbare	nipponbare_boundary_710	nipponbare_chr04	24767500	24772500
Nipponbare	nipponbare_boundary_711	nipponbare_chr04	24842500	24847500
Nipponbare	nipponbare_boundary_712	nipponbare_chr04	24917500	24922500
Nipponbare	nipponbare_boundary_713	nipponbare_chr04	25067500	25072500
Nipponbare	nipponbare_boundary_714	nipponbare_chr04	25117500	25122500
Nipponbare	nipponbare_boundary_715	nipponbare_chr04	25542500	25582500
Nipponbare	nipponbare_boundary_716	nipponbare_chr04	25642500	25647500
Nipponbare	nipponbare_boundary_717	nipponbare_chr04	25707500	25712500
Nipponbare	nipponbare_boundary_718	nipponbare_chr04	25757500	25762500
Nipponbare	nipponbare_boundary_719	nipponbare_chr04	25937500	25942500
Nipponbare	nipponbare_boundary_720	nipponbare_chr04	26037500	26042500
Nipponbare	nipponbare_boundary_721	nipponbare_chr04	26517500	26522500
Nipponbare	nipponbare_boundary_722	nipponbare_chr04	26712500	26717500
Nipponbare	nipponbare_boundary_723	nipponbare_chr04	27252500	27257500
Nipponbare	nipponbare_boundary_724	nipponbare_chr04	27342500	27347500
Nipponbare	nipponbare_boundary_725	nipponbare_chr04	27897500	27902500
Nipponbare	nipponbare_boundary_726	nipponbare_chr04	28027500	28032500
Nipponbare	nipponbare_boundary_727	nipponbare_chr04	28517500	28522500
Nipponbare	nipponbare_boundary_728	nipponbare_chr04	28572500	28577500
Nipponbare	nipponbare_boundary_729	nipponbare_chr04	28667500	28672500
Nipponbare	nipponbare_boundary_730	nipponbare_chr04	28742500	28747500
Nipponbare	nipponbare_boundary_731	nipponbare_chr04	28852500	28857500
Nipponbare	nipponbare_boundary_732	nipponbare_chr04	28917500	28922500

Nipponbare	nipponbare_boundary_733	nipponbare_chr04	29027500	29067500
Nipponbare	nipponbare_boundary_734	nipponbare_chr04	29117500	29162500
Nipponbare	nipponbare_boundary_735	nipponbare_chr04	29352500	29357500
Nipponbare	nipponbare_boundary_736	nipponbare_chr04	29402500	29407500
Nipponbare	nipponbare_boundary_737	nipponbare_chr04	29542500	29582500
Nipponbare	nipponbare_boundary_738	nipponbare_chr04	29645000	29650000
Nipponbare	nipponbare_boundary_739	nipponbare_chr04	29727500	29732500
Nipponbare	nipponbare_boundary_740	nipponbare_chr04	29797500	29802500
Nipponbare	nipponbare_boundary_741	nipponbare_chr04	29850000	29855000
Nipponbare	nipponbare_boundary_742	nipponbare_chr04	30677500	30682500
Nipponbare	nipponbare_boundary_743	nipponbare_chr04	30727500	30732500
Nipponbare	nipponbare_boundary_744	nipponbare_chr04	30812500	30817500
Nipponbare	nipponbare_boundary_745	nipponbare_chr04	31222500	31262500
Nipponbare	nipponbare_boundary_746	nipponbare_chr04	31852500	31857500
Nipponbare	nipponbare_boundary_747	nipponbare_chr04	31962500	31967500
Nipponbare	nipponbare_boundary_748	nipponbare_chr04	32277500	32282500
Nipponbare	nipponbare_boundary_749	nipponbare_chr04	32337500	32342500
Nipponbare	nipponbare_boundary_750	nipponbare_chr04	33112500	33117500
Nipponbare	nipponbare_boundary_751	nipponbare_chr04	33177500	33217500
Nipponbare	nipponbare_boundary_752	nipponbare_chr04	33702500	33707500
Nipponbare	nipponbare_boundary_753	nipponbare_chr04	33777500	33782500
Nipponbare	nipponbare_boundary_754	nipponbare_chr04	33852500	33857500
Nipponbare	nipponbare_boundary_755	nipponbare_chr04	33947500	33952500
Nipponbare	nipponbare_boundary_756	nipponbare_chr04	33992500	33997500
Nipponbare	nipponbare_boundary_757	nipponbare_chr04	34057500	34062500
Nipponbare	nipponbare_boundary_758	nipponbare_chr04	34662500	34667500
Nipponbare	nipponbare_boundary_759	nipponbare_chr04	34722500	34727500
Nipponbare	nipponbare_boundary_760	nipponbare_chr04	34892500	34897500
Nipponbare	nipponbare_boundary_761	nipponbare_chr05	97500	102500
Nipponbare	nipponbare_boundary_762	nipponbare_chr05	142500	147500
Nipponbare	nipponbare_boundary_763	nipponbare_chr05	202500	207500
Nipponbare	nipponbare_boundary_764	nipponbare_chr05	562500	602500

Nipponbare	nipponbare_boundary_765	nipponbare_chr05	672500	677500
Nipponbare	nipponbare_boundary_766	nipponbare_chr05	772500	777500
Nipponbare	nipponbare_boundary_767	nipponbare_chr05	882500	887500
Nipponbare	nipponbare_boundary_768	nipponbare_chr05	952500	957500
Nipponbare	nipponbare_boundary_769	nipponbare_chr05	1142500	1147500
Nipponbare	nipponbare_boundary_770	nipponbare_chr05	1187500	1192500
Nipponbare	nipponbare_boundary_771	nipponbare_chr05	1312500	1317500
Nipponbare	nipponbare_boundary_772	nipponbare_chr05	1422500	1427500
Nipponbare	nipponbare_boundary_773	nipponbare_chr05	1497500	1502500
Nipponbare	nipponbare_boundary_774	nipponbare_chr05	1717500	1757500
Nipponbare	nipponbare_boundary_775	nipponbare_chr05	1852500	1857500
Nipponbare	nipponbare_boundary_776	nipponbare_chr05	2222500	2262500
Nipponbare	nipponbare_boundary_777	nipponbare_chr05	2317500	2322500
Nipponbare	nipponbare_boundary_778	nipponbare_chr05	2487500	2492500
Nipponbare	nipponbare_boundary_779	nipponbare_chr05	2532500	2537500
Nipponbare	nipponbare_boundary_780	nipponbare_chr05	2622500	2627500
Nipponbare	nipponbare_boundary_781	nipponbare_chr05	3182500	3187500
Nipponbare	nipponbare_boundary_782	nipponbare_chr05	3237500	3242500
Nipponbare	nipponbare_boundary_783	nipponbare_chr05	3367500	3372500
Nipponbare	nipponbare_boundary_784	nipponbare_chr05	3467500	3472500
Nipponbare	nipponbare_boundary_785	nipponbare_chr05	3532500	3537500
Nipponbare	nipponbare_boundary_786	nipponbare_chr05	3662500	3667500
Nipponbare	nipponbare_boundary_787	nipponbare_chr05	3722500	3727500
Nipponbare	nipponbare_boundary_788	nipponbare_chr05	3772500	3777500
Nipponbare	nipponbare_boundary_789	nipponbare_chr05	5277500	5282500
Nipponbare	nipponbare_boundary_790	nipponbare_chr05	5342500	5347500
Nipponbare	nipponbare_boundary_791	nipponbare_chr05	5387500	5392500
Nipponbare	nipponbare_boundary_792	nipponbare_chr05	5447500	5492500
Nipponbare	nipponbare_boundary_793	nipponbare_chr05	5617500	5622500
Nipponbare	nipponbare_boundary_794	nipponbare_chr05	5932500	5937500
Nipponbare	nipponbare_boundary_795	nipponbare_chr05	6042500	6047500
Nipponbare	nipponbare_boundary_796	nipponbare_chr05	6087500	6092500

Nipponbare	nipponbare_boundary_797	nipponbare_chr05	6697500	6702500
Nipponbare	nipponbare_boundary_798	nipponbare_chr05	6767500	6772500
Nipponbare	nipponbare_boundary_799	nipponbare_chr05	6832500	6837500
Nipponbare	nipponbare_boundary_800	nipponbare_chr05	7067500	7072500
Nipponbare	nipponbare_boundary_801	nipponbare_chr05	7127500	7132500
Nipponbare	nipponbare_boundary_802	nipponbare_chr05	7217500	7222500
Nipponbare	nipponbare_boundary_803	nipponbare_chr05	7337500	7342500
Nipponbare	nipponbare_boundary_804	nipponbare_chr05	7447500	7490000
Nipponbare	nipponbare_boundary_805	nipponbare_chr05	7552500	7557500
Nipponbare	nipponbare_boundary_806	nipponbare_chr05	7622500	7627500
Nipponbare	nipponbare_boundary_807	nipponbare_chr05	7772500	7777500
Nipponbare	nipponbare_boundary_808	nipponbare_chr05	7842500	7847500
Nipponbare	nipponbare_boundary_809	nipponbare_chr05	7917500	7922500
Nipponbare	nipponbare_boundary_810	nipponbare_chr05	8037500	8042500
Nipponbare	nipponbare_boundary_811	nipponbare_chr05	8192500	8197500
Nipponbare	nipponbare_boundary_812	nipponbare_chr05	8312500	8317500
Nipponbare	nipponbare_boundary_813	nipponbare_chr05	8397500	8402500
Nipponbare	nipponbare_boundary_814	nipponbare_chr05	8502500	8507500
Nipponbare	nipponbare_boundary_815	nipponbare_chr05	8572500	8577500
Nipponbare	nipponbare_boundary_816	nipponbare_chr05	8702500	8707500
Nipponbare	nipponbare_boundary_817	nipponbare_chr05	8877500	8882500
Nipponbare	nipponbare_boundary_818	nipponbare_chr05	9617500	9622500
Nipponbare	nipponbare_boundary_819	nipponbare_chr05	9765000	9770000
Nipponbare	nipponbare_boundary_820	nipponbare_chr05	12682500	12687500
Nipponbare	nipponbare_boundary_821	nipponbare_chr05	12827500	12832500
Nipponbare	nipponbare_boundary_822	nipponbare_chr05	12927500	12932500
Nipponbare	nipponbare_boundary_823	nipponbare_chr05	13057500	13062500
Nipponbare	nipponbare_boundary_824	nipponbare_chr05	13702500	13707500
Nipponbare	nipponbare_boundary_825	nipponbare_chr05	13812500	13817500
Nipponbare	nipponbare_boundary_826	nipponbare_chr05	14032500	14037500
Nipponbare	nipponbare_boundary_827	nipponbare_chr05	14152500	14157500
Nipponbare	nipponbare_boundary_828	nipponbare_chr05	14692500	14697500

Nipponbare	nipponbare_boundary_829	nipponbare_chr05	14817500	14822500
Nipponbare	nipponbare_boundary_830	nipponbare_chr05	16012500	16017500
Nipponbare	nipponbare_boundary_831	nipponbare_chr05	16112500	16117500
Nipponbare	nipponbare_boundary_832	nipponbare_chr05	16162500	16167500
Nipponbare	nipponbare_boundary_833	nipponbare_chr05	16387500	16392500
Nipponbare	nipponbare_boundary_834	nipponbare_chr05	16467500	16552500
Nipponbare	nipponbare_boundary_835	nipponbare_chr05	16667500	16672500
Nipponbare	nipponbare_boundary_836	nipponbare_chr05	16752500	16757500
Nipponbare	nipponbare_boundary_837	nipponbare_chr05	16847500	16852500
Nipponbare	nipponbare_boundary_838	nipponbare_chr05	16917500	16922500
Nipponbare	nipponbare_boundary_839	nipponbare_chr05	16992500	16997500
Nipponbare	nipponbare_boundary_840	nipponbare_chr05	17337500	17342500
Nipponbare	nipponbare_boundary_841	nipponbare_chr05	17385000	17390000
Nipponbare	nipponbare_boundary_842	nipponbare_chr05	17452500	17457500
Nipponbare	nipponbare_boundary_843	nipponbare_chr05	17522500	17527500
Nipponbare	nipponbare_boundary_844	nipponbare_chr05	17577500	17582500
Nipponbare	nipponbare_boundary_845	nipponbare_chr05	17622500	17627500
Nipponbare	nipponbare_boundary_846	nipponbare_chr05	17952500	17957500
Nipponbare	nipponbare_boundary_847	nipponbare_chr05	18007500	18012500
Nipponbare	nipponbare_boundary_848	nipponbare_chr05	18082500	18127500
Nipponbare	nipponbare_boundary_849	nipponbare_chr05	18192500	18197500
Nipponbare	nipponbare_boundary_850	nipponbare_chr05	18297500	18302500
Nipponbare	nipponbare_boundary_851	nipponbare_chr05	18397500	18432500
Nipponbare	nipponbare_boundary_852	nipponbare_chr05	18732500	18777500
Nipponbare	nipponbare_boundary_853	nipponbare_chr05	18837500	18877500
Nipponbare	nipponbare_boundary_854	nipponbare_chr05	19112500	19117500
Nipponbare	nipponbare_boundary_855	nipponbare_chr05	19157500	19162500
Nipponbare	nipponbare_boundary_856	nipponbare_chr05	19352500	19357500
Nipponbare	nipponbare_boundary_857	nipponbare_chr05	19417500	19422500
Nipponbare	nipponbare_boundary_858	nipponbare_chr05	19477500	19482500
Nipponbare	nipponbare_boundary_859	nipponbare_chr05	19772500	19777500
Nipponbare	nipponbare_boundary_860	nipponbare_chr05	19832500	19837500

Nipponbare	nipponbare_boundary_861	nipponbare_chr05	19897500	19902500
Nipponbare	nipponbare_boundary_862	nipponbare_chr05	19987500	19992500
Nipponbare	nipponbare_boundary_863	nipponbare_chr05	20107500	20112500
Nipponbare	nipponbare_boundary_864	nipponbare_chr05	20167500	20172500
Nipponbare	nipponbare_boundary_865	nipponbare_chr05	20257500	20262500
Nipponbare	nipponbare_boundary_866	nipponbare_chr05	20312500	20317500
Nipponbare	nipponbare_boundary_867	nipponbare_chr05	20537500	20582500
Nipponbare	nipponbare_boundary_868	nipponbare_chr05	20657500	20662500
Nipponbare	nipponbare_boundary_869	nipponbare_chr05	20722500	20727500
Nipponbare	nipponbare_boundary_870	nipponbare_chr05	20787500	20792500
Nipponbare	nipponbare_boundary_871	nipponbare_chr05	20877500	20882500
Nipponbare	nipponbare_boundary_872	nipponbare_chr05	20932500	20937500
Nipponbare	nipponbare_boundary_873	nipponbare_chr05	21047500	21082500
Nipponbare	nipponbare_boundary_874	nipponbare_chr05	21137500	21142500
Nipponbare	nipponbare_boundary_875	nipponbare_chr05	21217500	21222500
Nipponbare	nipponbare_boundary_876	nipponbare_chr05	21562500	21567500
Nipponbare	nipponbare_boundary_877	nipponbare_chr05	21642500	21647500
Nipponbare	nipponbare_boundary_878	nipponbare_chr05	21712500	21717500
Nipponbare	nipponbare_boundary_879	nipponbare_chr05	22157500	22192500
Nipponbare	nipponbare_boundary_880	nipponbare_chr05	22277500	22282500
Nipponbare	nipponbare_boundary_881	nipponbare_chr05	22392500	22397500
Nipponbare	nipponbare_boundary_882	nipponbare_chr05	22457500	22502500
Nipponbare	nipponbare_boundary_883	nipponbare_chr05	22567500	22572500
Nipponbare	nipponbare_boundary_884	nipponbare_chr05	22642500	22647500
Nipponbare	nipponbare_boundary_885	nipponbare_chr05	22772500	22777500
Nipponbare	nipponbare_boundary_886	nipponbare_chr05	22882500	22887500
Nipponbare	nipponbare_boundary_887	nipponbare_chr05	22937500	22942500
Nipponbare	nipponbare_boundary_888	nipponbare_chr05	22992500	22997500
Nipponbare	nipponbare_boundary_889	nipponbare_chr05	23102500	23107500
Nipponbare	nipponbare_boundary_890	nipponbare_chr05	23527500	23567500
Nipponbare	nipponbare_boundary_891	nipponbare_chr05	23632500	23637500
Nipponbare	nipponbare_boundary_892	nipponbare_chr05	23692500	23697500

Nipponbare	nipponbare_boundary_893	nipponbare_chr05	24027500	24032500
Nipponbare	nipponbare_boundary_894	nipponbare_chr05	24077500	24082500
Nipponbare	nipponbare_boundary_895	nipponbare_chr05	24122500	24127500
Nipponbare	nipponbare_boundary_896	nipponbare_chr05	24182500	24187500
Nipponbare	nipponbare_boundary_897	nipponbare_chr05	24582500	24587500
Nipponbare	nipponbare_boundary_898	nipponbare_chr05	24627500	24632500
Nipponbare	nipponbare_boundary_899	nipponbare_chr05	24672500	24677500
Nipponbare	nipponbare_boundary_900	nipponbare_chr05	24782500	24787500
Nipponbare	nipponbare_boundary_901	nipponbare_chr05	25157500	25262500
Nipponbare	nipponbare_boundary_902	nipponbare_chr05	25317500	25322500
Nipponbare	nipponbare_boundary_903	nipponbare_chr05	25392500	25397500
Nipponbare	nipponbare_boundary_904	nipponbare_chr05	25452500	25457500
Nipponbare	nipponbare_boundary_905	nipponbare_chr05	25507500	25512500
Nipponbare	nipponbare_boundary_906	nipponbare_chr05	25662500	25667500
Nipponbare	nipponbare_boundary_907	nipponbare_chr05	25747500	25752500
Nipponbare	nipponbare_boundary_908	nipponbare_chr05	26102500	26107500
Nipponbare	nipponbare_boundary_909	nipponbare_chr05	26187500	26192500
Nipponbare	nipponbare_boundary_910	nipponbare_chr05	26272500	26277500
Nipponbare	nipponbare_boundary_911	nipponbare_chr05	26372500	26412500
Nipponbare	nipponbare_boundary_912	nipponbare_chr05	26497500	26502500
Nipponbare	nipponbare_boundary_913	nipponbare_chr05	26712500	26717500
Nipponbare	nipponbare_boundary_914	nipponbare_chr05	26797500	26837500
Nipponbare	nipponbare_boundary_915	nipponbare_chr05	26887500	26972500
Nipponbare	nipponbare_boundary_916	nipponbare_chr05	27047500	27052500
Nipponbare	nipponbare_boundary_917	nipponbare_chr05	27092500	27127500
Nipponbare	nipponbare_boundary_918	nipponbare_chr05	27217500	27252500
Nipponbare	nipponbare_boundary_919	nipponbare_chr05	27367500	27372500
Nipponbare	nipponbare_boundary_920	nipponbare_chr05	27452500	27457500
Nipponbare	nipponbare_boundary_921	nipponbare_chr05	27512500	27517500
Nipponbare	nipponbare_boundary_922	nipponbare_chr05	27602500	27642500
Nipponbare	nipponbare_boundary_923	nipponbare_chr05	27692500	27697500
Nipponbare	nipponbare_boundary_924	nipponbare_chr05	27932500	27937500

Nipponbare	nipponbare_boundary_925	nipponbare_chr05	27987500	27992500
Nipponbare	nipponbare_boundary_926	nipponbare_chr05	28042500	28047500
Nipponbare	nipponbare_boundary_927	nipponbare_chr05	28267500	28352500
Nipponbare	nipponbare_boundary_928	nipponbare_chr05	28542500	28627500
Nipponbare	nipponbare_boundary_929	nipponbare_chr05	28742500	28747500
Nipponbare	nipponbare_boundary_930	nipponbare_chr05	28797500	28802500
Nipponbare	nipponbare_boundary_931	nipponbare_chr05	28885000	28890000
Nipponbare	nipponbare_boundary_932	nipponbare_chr05	28947500	28952500
Nipponbare	nipponbare_boundary_933	nipponbare_chr05	29322500	29327500
Nipponbare	nipponbare_boundary_934	nipponbare_chr05	29487500	29492500
Nipponbare	nipponbare_boundary_935	nipponbare_chr05	29662500	29667500
Nipponbare	nipponbare_boundary_936	nipponbare_chr05	29747500	29752500
Nipponbare	nipponbare_boundary_937	nipponbare_chr06	137500	142500
Nipponbare	nipponbare_boundary_938	nipponbare_chr06	217500	222500
Nipponbare	nipponbare_boundary_939	nipponbare_chr06	282500	287500
Nipponbare	nipponbare_boundary_940	nipponbare_chr06	417500	422500
Nipponbare	nipponbare_boundary_941	nipponbare_chr06	487500	492500
Nipponbare	nipponbare_boundary_942	nipponbare_chr06	547500	552500
Nipponbare	nipponbare_boundary_943	nipponbare_chr06	627500	632500
Nipponbare	nipponbare_boundary_944	nipponbare_chr06	697500	702500
Nipponbare	nipponbare_boundary_945	nipponbare_chr06	1142500	1147500
Nipponbare	nipponbare_boundary_946	nipponbare_chr06	1357500	1362500
Nipponbare	nipponbare_boundary_947	nipponbare_chr06	1467500	1472500
Nipponbare	nipponbare_boundary_948	nipponbare_chr06	2212500	2217500
Nipponbare	nipponbare_boundary_949	nipponbare_chr06	2282500	2287500
Nipponbare	nipponbare_boundary_950	nipponbare_chr06	2377500	2382500
Nipponbare	nipponbare_boundary_951	nipponbare_chr06	3037500	3042500
Nipponbare	nipponbare_boundary_952	nipponbare_chr06	3122500	3127500
Nipponbare	nipponbare_boundary_953	nipponbare_chr06	3467500	3472500
Nipponbare	nipponbare_boundary_954	nipponbare_chr06	3532500	3537500
Nipponbare	nipponbare_boundary_955	nipponbare_chr06	3582500	3587500
Nipponbare	nipponbare_boundary_956	nipponbare_chr06	3687500	3692500

Nipponbare	nipponbare_boundary_957	nipponbare_chr06	3757500	3762500
Nipponbare	nipponbare_boundary_958	nipponbare_chr06	3932500	3937500
Nipponbare	nipponbare_boundary_959	nipponbare_chr06	4552500	4557500
Nipponbare	nipponbare_boundary_960	nipponbare_chr06	4657500	4662500
Nipponbare	nipponbare_boundary_961	nipponbare_chr06	4722500	4727500
Nipponbare	nipponbare_boundary_962	nipponbare_chr06	5237500	5242500
Nipponbare	nipponbare_boundary_963	nipponbare_chr06	5307500	5312500
Nipponbare	nipponbare_boundary_964	nipponbare_chr06	5372500	5377500
Nipponbare	nipponbare_boundary_965	nipponbare_chr06	5617500	5622500
Nipponbare	nipponbare_boundary_966	nipponbare_chr06	5692500	5697500
Nipponbare	nipponbare_boundary_967	nipponbare_chr06	7692500	7697500
Nipponbare	nipponbare_boundary_968	nipponbare_chr06	7787500	7792500
Nipponbare	nipponbare_boundary_969	nipponbare_chr06	7842500	7847500
Nipponbare	nipponbare_boundary_970	nipponbare_chr06	7917500	7922500
Nipponbare	nipponbare_boundary_971	nipponbare_chr06	7987500	7992500
Nipponbare	nipponbare_boundary_972	nipponbare_chr06	8112500	8117500
Nipponbare	nipponbare_boundary_973	nipponbare_chr06	8182500	8187500
Nipponbare	nipponbare_boundary_974	nipponbare_chr06	8237500	8242500
Nipponbare	nipponbare_boundary_975	nipponbare_chr06	8937500	8972500
Nipponbare	nipponbare_boundary_976	nipponbare_chr06	9357500	9362500
Nipponbare	nipponbare_boundary_977	nipponbare_chr06	9407500	9412500
Nipponbare	nipponbare_boundary_978	nipponbare_chr06	10882500	10922500
Nipponbare	nipponbare_boundary_979	nipponbare_chr06	11022500	11027500
Nipponbare	nipponbare_boundary_980	nipponbare_chr06	11387500	11392500
Nipponbare	nipponbare_boundary_981	nipponbare_chr06	11447500	11452500
Nipponbare	nipponbare_boundary_982	nipponbare_chr06	11967500	11972500
Nipponbare	nipponbare_boundary_983	nipponbare_chr06	12057500	12062500
Nipponbare	nipponbare_boundary_984	nipponbare_chr06	12102500	12107500
Nipponbare	nipponbare_boundary_985	nipponbare_chr06	12317500	12322500
Nipponbare	nipponbare_boundary_986	nipponbare_chr06	12397500	12402500
Nipponbare	nipponbare_boundary_987	nipponbare_chr06	13882500	13887500
Nipponbare	nipponbare_boundary_988	nipponbare_chr06	13940000	13945000

Nipponbare	nipponbare_boundary_989	nipponbare_chr06	14037500	14042500
Nipponbare	nipponbare_boundary_990	nipponbare_chr06	14087500	14092500
Nipponbare	nipponbare_boundary_991	nipponbare_chr06	15197500	15202500
Nipponbare	nipponbare_boundary_992	nipponbare_chr06	15282500	15287500
Nipponbare	nipponbare_boundary_993	nipponbare_chr06	16992500	16997500
Nipponbare	nipponbare_boundary_994	nipponbare_chr06	17037500	17042500
Nipponbare	nipponbare_boundary_995	nipponbare_chr06	17142500	17147500
Nipponbare	nipponbare_boundary_996	nipponbare_chr06	17267500	17272500
Nipponbare	nipponbare_boundary_997	nipponbare_chr06	17352500	17357500
Nipponbare	nipponbare_boundary_998	nipponbare_chr06	17447500	17452500
Nipponbare	nipponbare_boundary_999	nipponbare_chr06	17502500	17507500
Nipponbare	nipponbare_boundary_1000	nipponbare_chr06	17587500	17592500
Nipponbare	nipponbare_boundary_1001	nipponbare_chr06	17657500	17662500
Nipponbare	nipponbare_boundary_1002	nipponbare_chr06	19917500	19922500
Nipponbare	nipponbare_boundary_1003	nipponbare_chr06	20022500	20027500
Nipponbare	nipponbare_boundary_1004	nipponbare_chr06	20097500	20102500
Nipponbare	nipponbare_boundary_1005	nipponbare_chr06	20807500	20812500
Nipponbare	nipponbare_boundary_1006	nipponbare_chr06	20852500	20857500
Nipponbare	nipponbare_boundary_1007	nipponbare_chr06	20932500	20937500
Nipponbare	nipponbare_boundary_1008	nipponbare_chr06	20982500	20987500
Nipponbare	nipponbare_boundary_1009	nipponbare_chr06	21097500	21102500
Nipponbare	nipponbare_boundary_1010	nipponbare_chr06	21167500	21172500
Nipponbare	nipponbare_boundary_1011	nipponbare_chr06	21212500	21217500
Nipponbare	nipponbare_boundary_1012	nipponbare_chr06	21282500	21287500
Nipponbare	nipponbare_boundary_1013	nipponbare_chr06	21342500	21347500
Nipponbare	nipponbare_boundary_1014	nipponbare_chr06	21432500	21437500
Nipponbare	nipponbare_boundary_1015	nipponbare_chr06	21482500	21487500
Nipponbare	nipponbare_boundary_1016	nipponbare_chr06	21597500	21602500
Nipponbare	nipponbare_boundary_1017	nipponbare_chr06	21692500	21697500
Nipponbare	nipponbare_boundary_1018	nipponbare_chr06	21757500	21762500
Nipponbare	nipponbare_boundary_1019	nipponbare_chr06	21972500	21977500
Nipponbare	nipponbare_boundary_1020	nipponbare_chr06	22077500	22082500

Nipponbare	nipponbare_boundary_1021	nipponbare_chr06	22137500	22142500
Nipponbare	nipponbare_boundary_1022	nipponbare_chr06	22252500	22257500
Nipponbare	nipponbare_boundary_1023	nipponbare_chr06	22362500	22367500
Nipponbare	nipponbare_boundary_1024	nipponbare_chr06	22652500	22657500
Nipponbare	nipponbare_boundary_1025	nipponbare_chr06	22702500	22707500
Nipponbare	nipponbare_boundary_1026	nipponbare_chr06	22797500	22802500
Nipponbare	nipponbare_boundary_1027	nipponbare_chr06	23302500	23307500
Nipponbare	nipponbare_boundary_1028	nipponbare_chr06	23372500	23452500
Nipponbare	nipponbare_boundary_1029	nipponbare_chr06	23512500	23517500
Nipponbare	nipponbare_boundary_1030	nipponbare_chr06	23682500	23727500
Nipponbare	nipponbare_boundary_1031	nipponbare_chr06	23817500	23822500
Nipponbare	nipponbare_boundary_1032	nipponbare_chr06	23902500	23907500
Nipponbare	nipponbare_boundary_1033	nipponbare_chr06	23962500	23967500
Nipponbare	nipponbare_boundary_1034	nipponbare_chr06	24512500	24517500
Nipponbare	nipponbare_boundary_1035	nipponbare_chr06	24572500	24577500
Nipponbare	nipponbare_boundary_1036	nipponbare_chr06	25037500	25042500
Nipponbare	nipponbare_boundary_1037	nipponbare_chr06	25082500	25087500
Nipponbare	nipponbare_boundary_1038	nipponbare_chr06	26187500	26192500
Nipponbare	nipponbare_boundary_1039	nipponbare_chr06	26232500	26237500
Nipponbare	nipponbare_boundary_1040	nipponbare_chr06	26312500	26317500
Nipponbare	nipponbare_boundary_1041	nipponbare_chr06	26357500	26362500
Nipponbare	nipponbare_boundary_1042	nipponbare_chr06	26407500	26412500
Nipponbare	nipponbare_boundary_1043	nipponbare_chr06	26527500	26532500
Nipponbare	nipponbare_boundary_1044	nipponbare_chr06	26582500	26587500
Nipponbare	nipponbare_boundary_1045	nipponbare_chr06	26627500	26712500
Nipponbare	nipponbare_boundary_1046	nipponbare_chr06	27382500	27387500
Nipponbare	nipponbare_boundary_1047	nipponbare_chr06	27442500	27447500
Nipponbare	nipponbare_boundary_1048	nipponbare_chr06	27702500	27707500
Nipponbare	nipponbare_boundary_1049	nipponbare_chr06	27807500	27812500
Nipponbare	nipponbare_boundary_1050	nipponbare_chr06	27877500	27882500
Nipponbare	nipponbare_boundary_1051	nipponbare_chr06	28147500	28152500
Nipponbare	nipponbare_boundary_1052	nipponbare_chr06	28192500	28197500

Nipponbare	nipponbare_boundary_1053	nipponbare_chr06	28372500	28377500
Nipponbare	nipponbare_boundary_1054	nipponbare_chr06	28467500	28497500
Nipponbare	nipponbare_boundary_1055	nipponbare_chr06	28552500	28557500
Nipponbare	nipponbare_boundary_1056	nipponbare_chr06	28697500	28702500
Nipponbare	nipponbare_boundary_1057	nipponbare_chr06	28782500	28787500
Nipponbare	nipponbare_boundary_1058	nipponbare_chr06	28857500	28862500
Nipponbare	nipponbare_boundary_1059	nipponbare_chr06	28922500	28957500
Nipponbare	nipponbare_boundary_1060	nipponbare_chr06	29067500	29072500
Nipponbare	nipponbare_boundary_1061	nipponbare_chr06	29412500	29417500
Nipponbare	nipponbare_boundary_1062	nipponbare_chr06	29522500	29527500
Nipponbare	nipponbare_boundary_1063	nipponbare_chr06	29622500	29627500
Nipponbare	nipponbare_boundary_1064	nipponbare_chr06	29667500	29672500
Nipponbare	nipponbare_boundary_1065	nipponbare_chr06	29742500	29747500
Nipponbare	nipponbare_boundary_1066	nipponbare_chr06	29802500	29807500
Nipponbare	nipponbare_boundary_1067	nipponbare_chr06	29887500	29892500
Nipponbare	nipponbare_boundary_1068	nipponbare_chr06	29947500	29952500
Nipponbare	nipponbare_boundary_1069	nipponbare_chr06	29992500	29997500
Nipponbare	nipponbare_boundary_1070	nipponbare_chr06	30337500	30342500
Nipponbare	nipponbare_boundary_1071	nipponbare_chr06	30472500	30477500
Nipponbare	nipponbare_boundary_1072	nipponbare_chr07	257500	262500
Nipponbare	nipponbare_boundary_1073	nipponbare_chr07	302500	307500
Nipponbare	nipponbare_boundary_1074	nipponbare_chr07	452500	457500
Nipponbare	nipponbare_boundary_1075	nipponbare_chr07	522500	562500
Nipponbare	nipponbare_boundary_1076	nipponbare_chr07	612500	617500
Nipponbare	nipponbare_boundary_1077	nipponbare_chr07	697500	702500
Nipponbare	nipponbare_boundary_1078	nipponbare_chr07	752500	757500
Nipponbare	nipponbare_boundary_1079	nipponbare_chr07	1102500	1107500
Nipponbare	nipponbare_boundary_1080	nipponbare_chr07	1162500	1167500
Nipponbare	nipponbare_boundary_1081	nipponbare_chr07	1237500	1282500
Nipponbare	nipponbare_boundary_1082	nipponbare_chr07	2097500	2102500
Nipponbare	nipponbare_boundary_1083	nipponbare_chr07	2167500	2172500
Nipponbare	nipponbare_boundary_1084	nipponbare_chr07	2222500	2227500

Nipponbare	nipponbare_boundary_1085	nipponbare_chr07	2457500	2462500
Nipponbare	nipponbare_boundary_1086	nipponbare_chr07	2522500	2527500
Nipponbare	nipponbare_boundary_1087	nipponbare_chr07	2592500	2597500
Nipponbare	nipponbare_boundary_1088	nipponbare_chr07	2662500	2667500
Nipponbare	nipponbare_boundary_1089	nipponbare_chr07	2727500	2732500
Nipponbare	nipponbare_boundary_1090	nipponbare_chr07	3452500	3457500
Nipponbare	nipponbare_boundary_1091	nipponbare_chr07	3572500	3577500
Nipponbare	nipponbare_boundary_1092	nipponbare_chr07	3672500	3677500
Nipponbare	nipponbare_boundary_1093	nipponbare_chr07	3727500	3732500
Nipponbare	nipponbare_boundary_1094	nipponbare_chr07	3867500	3872500
Nipponbare	nipponbare_boundary_1095	nipponbare_chr07	3912500	3917500
Nipponbare	nipponbare_boundary_1096	nipponbare_chr07	3977500	4012500
Nipponbare	nipponbare_boundary_1097	nipponbare_chr07	4087500	4092500
Nipponbare	nipponbare_boundary_1098	nipponbare_chr07	4177500	4182500
Nipponbare	nipponbare_boundary_1099	nipponbare_chr07	4262500	4267500
Nipponbare	nipponbare_boundary_1100	nipponbare_chr07	4602500	4607500
Nipponbare	nipponbare_boundary_1101	nipponbare_chr07	4662500	4667500
Nipponbare	nipponbare_boundary_1102	nipponbare_chr07	4737500	4777500
Nipponbare	nipponbare_boundary_1103	nipponbare_chr07	4827500	4832500
Nipponbare	nipponbare_boundary_1104	nipponbare_chr07	4887500	4892500
Nipponbare	nipponbare_boundary_1105	nipponbare_chr07	4932500	4937500
Nipponbare	nipponbare_boundary_1106	nipponbare_chr07	5170000	5175000
Nipponbare	nipponbare_boundary_1107	nipponbare_chr07	5217500	5222500
Nipponbare	nipponbare_boundary_1108	nipponbare_chr07	5492500	5497500
Nipponbare	nipponbare_boundary_1109	nipponbare_chr07	5657500	5662500
Nipponbare	nipponbare_boundary_1110	nipponbare_chr07	5877500	5882500
Nipponbare	nipponbare_boundary_1111	nipponbare_chr07	5947500	5952500
Nipponbare	nipponbare_boundary_1112	nipponbare_chr07	6017500	6057500
Nipponbare	nipponbare_boundary_1113	nipponbare_chr07	6757500	6762500
Nipponbare	nipponbare_boundary_1114	nipponbare_chr07	6802500	6807500
Nipponbare	nipponbare_boundary_1115	nipponbare_chr07	6862500	6867500
Nipponbare	nipponbare_boundary_1116	nipponbare_chr07	7107500	7112500

Nipponbare	nipponbare_boundary_1117	nipponbare_chr07	7152500	7157500
Nipponbare	nipponbare_boundary_1118	nipponbare_chr07	7232500	7237500
Nipponbare	nipponbare_boundary_1119	nipponbare_chr07	7312500	7317500
Nipponbare	nipponbare_boundary_1120	nipponbare_chr07	7417500	7422500
Nipponbare	nipponbare_boundary_1121	nipponbare_chr07	7582500	7587500
Nipponbare	nipponbare_boundary_1122	nipponbare_chr07	8162500	8167500
Nipponbare	nipponbare_boundary_1123	nipponbare_chr07	8347500	8352500
Nipponbare	nipponbare_boundary_1124	nipponbare_chr07	8537500	8542500
Nipponbare	nipponbare_boundary_1125	nipponbare_chr07	8620000	8625000
Nipponbare	nipponbare_boundary_1126	nipponbare_chr07	8752500	8757500
Nipponbare	nipponbare_boundary_1127	nipponbare_chr07	8812500	8817500
Nipponbare	nipponbare_boundary_1128	nipponbare_chr07	8897500	8902500
Nipponbare	nipponbare_boundary_1129	nipponbare_chr07	10137500	10142500
Nipponbare	nipponbare_boundary_1130	nipponbare_chr07	10257500	10262500
Nipponbare	nipponbare_boundary_1131	nipponbare_chr07	11267500	11272500
Nipponbare	nipponbare_boundary_1132	nipponbare_chr07	11352500	11357500
Nipponbare	nipponbare_boundary_1133	nipponbare_chr07	12417500	12422500
Nipponbare	nipponbare_boundary_1134	nipponbare_chr07	12547500	12552500
Nipponbare	nipponbare_boundary_1135	nipponbare_chr07	12617500	12622500
Nipponbare	nipponbare_boundary_1136	nipponbare_chr07	12697500	12702500
Nipponbare	nipponbare_boundary_1137	nipponbare_chr07	12777500	12782500
Nipponbare	nipponbare_boundary_1138	nipponbare_chr07	13382500	13387500
Nipponbare	nipponbare_boundary_1139	nipponbare_chr07	13437500	13442500
Nipponbare	nipponbare_boundary_1140	nipponbare_chr07	13602500	13607500
Nipponbare	nipponbare_boundary_1141	nipponbare_chr07	13652500	13657500
Nipponbare	nipponbare_boundary_1142	nipponbare_chr07	14522500	14527500
Nipponbare	nipponbare_boundary_1143	nipponbare_chr07	14607500	14612500
Nipponbare	nipponbare_boundary_1144	nipponbare_chr07	14662500	14667500
Nipponbare	nipponbare_boundary_1145	nipponbare_chr07	15627500	15632500
Nipponbare	nipponbare_boundary_1146	nipponbare_chr07	15712500	15717500
Nipponbare	nipponbare_boundary_1147	nipponbare_chr07	15937500	15942500
Nipponbare	nipponbare_boundary_1148	nipponbare_chr07	16032500	16037500

Nipponbare	nipponbare_boundary_1149	nipponbare_chr07	16132500	16137500
Nipponbare	nipponbare_boundary_1150	nipponbare_chr07	16292500	16297500
Nipponbare	nipponbare_boundary_1151	nipponbare_chr07	16372500	16377500
Nipponbare	nipponbare_boundary_1152	nipponbare_chr07	16602500	16607500
Nipponbare	nipponbare_boundary_1153	nipponbare_chr07	16657500	16662500
Nipponbare	nipponbare_boundary_1154	nipponbare_chr07	16762500	16767500
Nipponbare	nipponbare_boundary_1155	nipponbare_chr07	16867500	16872500
Nipponbare	nipponbare_boundary_1156	nipponbare_chr07	17402500	17407500
Nipponbare	nipponbare_boundary_1157	nipponbare_chr07	17497500	17502500
Nipponbare	nipponbare_boundary_1158	nipponbare_chr07	17592500	17597500
Nipponbare	nipponbare_boundary_1159	nipponbare_chr07	18337500	18342500
Nipponbare	nipponbare_boundary_1160	nipponbare_chr07	18502500	18537500
Nipponbare	nipponbare_boundary_1161	nipponbare_chr07	18757500	18762500
Nipponbare	nipponbare_boundary_1162	nipponbare_chr07	18807500	18847500
Nipponbare	nipponbare_boundary_1163	nipponbare_chr07	18917500	18922500
Nipponbare	nipponbare_boundary_1164	nipponbare_chr07	19002500	19007500
Nipponbare	nipponbare_boundary_1165	nipponbare_chr07	19092500	19097500
Nipponbare	nipponbare_boundary_1166	nipponbare_chr07	19432500	19437500
Nipponbare	nipponbare_boundary_1167	nipponbare_chr07	19482500	19487500
Nipponbare	nipponbare_boundary_1168	nipponbare_chr07	19537500	19542500
Nipponbare	nipponbare_boundary_1169	nipponbare_chr07	19762500	19767500
Nipponbare	nipponbare_boundary_1170	nipponbare_chr07	19857500	19862500
Nipponbare	nipponbare_boundary_1171	nipponbare_chr07	19937500	19977500
Nipponbare	nipponbare_boundary_1172	nipponbare_chr07	20087500	20132500
Nipponbare	nipponbare_boundary_1173	nipponbare_chr07	20207500	20212500
Nipponbare	nipponbare_boundary_1174	nipponbare_chr07	20727500	20732500
Nipponbare	nipponbare_boundary_1175	nipponbare_chr07	20782500	20787500
Nipponbare	nipponbare_boundary_1176	nipponbare_chr07	21227500	21232500
Nipponbare	nipponbare_boundary_1177	nipponbare_chr07	21292500	21297500
Nipponbare	nipponbare_boundary_1178	nipponbare_chr07	21467500	21472500
Nipponbare	nipponbare_boundary_1179	nipponbare_chr07	21577500	21582500
Nipponbare	nipponbare_boundary_1180	nipponbare_chr07	21672500	21677500

Nipponbare	nipponbare_boundary_1181	nipponbare_chr07	21757500	21827500
Nipponbare	nipponbare_boundary_1182	nipponbare_chr07	22112500	22117500
Nipponbare	nipponbare_boundary_1183	nipponbare_chr07	22192500	22197500
Nipponbare	nipponbare_boundary_1184	nipponbare_chr07	22252500	22257500
Nipponbare	nipponbare_boundary_1185	nipponbare_chr07	22307500	22347500
Nipponbare	nipponbare_boundary_1186	nipponbare_chr07	22507500	22512500
Nipponbare	nipponbare_boundary_1187	nipponbare_chr07	22567500	22572500
Nipponbare	nipponbare_boundary_1188	nipponbare_chr07	22657500	22662500
Nipponbare	nipponbare_boundary_1189	nipponbare_chr07	22822500	22827500
Nipponbare	nipponbare_boundary_1190	nipponbare_chr07	22877500	22882500
Nipponbare	nipponbare_boundary_1191	nipponbare_chr07	22927500	22932500
Nipponbare	nipponbare_boundary_1192	nipponbare_chr07	23247500	23252500
Nipponbare	nipponbare_boundary_1193	nipponbare_chr07	23292500	23297500
Nipponbare	nipponbare_boundary_1194	nipponbare_chr07	23657500	23662500
Nipponbare	nipponbare_boundary_1195	nipponbare_chr07	23712500	23717500
Nipponbare	nipponbare_boundary_1196	nipponbare_chr07	23757500	23802500
Nipponbare	nipponbare_boundary_1197	nipponbare_chr07	24172500	24177500
Nipponbare	nipponbare_boundary_1198	nipponbare_chr07	24237500	24242500
Nipponbare	nipponbare_boundary_1199	nipponbare_chr07	24387500	24392500
Nipponbare	nipponbare_boundary_1200	nipponbare_chr07	24482500	24487500
Nipponbare	nipponbare_boundary_1201	nipponbare_chr07	24567500	24572500
Nipponbare	nipponbare_boundary_1202	nipponbare_chr07	24627500	24692500
Nipponbare	nipponbare_boundary_1203	nipponbare_chr07	24762500	24767500
Nipponbare	nipponbare_boundary_1204	nipponbare_chr07	25047500	25052500
Nipponbare	nipponbare_boundary_1205	nipponbare_chr07	25097500	25102500
Nipponbare	nipponbare_boundary_1206	nipponbare_chr07	25207500	25212500
Nipponbare	nipponbare_boundary_1207	nipponbare_chr07	25647500	25652500
Nipponbare	nipponbare_boundary_1208	nipponbare_chr07	25727500	25732500
Nipponbare	nipponbare_boundary_1209	nipponbare_chr07	25797500	25842500
Nipponbare	nipponbare_boundary_1210	nipponbare_chr07	25932500	25937500
Nipponbare	nipponbare_boundary_1211	nipponbare_chr07	26022500	26027500
Nipponbare	nipponbare_boundary_1212	nipponbare_chr07	26727500	26732500

Nipponbare	nipponbare_boundary_1213	nipponbare_chr07	26777500	26857500
Nipponbare	nipponbare_boundary_1214	nipponbare_chr07	26907500	26912500
Nipponbare	nipponbare_boundary_1215	nipponbare_chr07	26980000	26985000
Nipponbare	nipponbare_boundary_1216	nipponbare_chr07	27047500	27052500
Nipponbare	nipponbare_boundary_1217	nipponbare_chr07	27097500	27102500
Nipponbare	nipponbare_boundary_1218	nipponbare_chr07	27582500	27587500
Nipponbare	nipponbare_boundary_1219	nipponbare_chr07	27652500	27657500
Nipponbare	nipponbare_boundary_1220	nipponbare_chr07	27802500	27807500
Nipponbare	nipponbare_boundary_1221	nipponbare_chr07	27877500	27882500
Nipponbare	nipponbare_boundary_1222	nipponbare_chr07	27942500	27947500
Nipponbare	nipponbare_boundary_1223	nipponbare_chr07	28142500	28182500
Nipponbare	nipponbare_boundary_1224	nipponbare_chr07	28237500	28242500
Nipponbare	nipponbare_boundary_1225	nipponbare_chr07	28442500	28482500
Nipponbare	nipponbare_boundary_1226	nipponbare_chr07	28907500	28912500
Nipponbare	nipponbare_boundary_1227	nipponbare_chr07	28957500	28962500
Nipponbare	nipponbare_boundary_1228	nipponbare_chr07	29007500	29012500
Nipponbare	nipponbare_boundary_1229	nipponbare_chr07	29057500	29062500
Nipponbare	nipponbare_boundary_1230	nipponbare_chr07	29107500	29132500
Nipponbare	nipponbare_boundary_1231	nipponbare_chr07	29202500	29207500
Nipponbare	nipponbare_boundary_1232	nipponbare_chr07	29487500	29492500
Nipponbare	nipponbare_boundary_1233	nipponbare_chr07	29532500	29537500
Nipponbare	nipponbare_boundary_1234	nipponbare_chr08	52500	57500
Nipponbare	nipponbare_boundary_1235	nipponbare_chr08	112500	117500
Nipponbare	nipponbare_boundary_1236	nipponbare_chr08	182500	187500
Nipponbare	nipponbare_boundary_1237	nipponbare_chr08	352500	357500
Nipponbare	nipponbare_boundary_1238	nipponbare_chr08	437500	442500
Nipponbare	nipponbare_boundary_1239	nipponbare_chr08	547500	552500
Nipponbare	nipponbare_boundary_1240	nipponbare_chr08	647500	652500
Nipponbare	nipponbare_boundary_1241	nipponbare_chr08	1117500	1122500
Nipponbare	nipponbare_boundary_1242	nipponbare_chr08	1192500	1197500
Nipponbare	nipponbare_boundary_1243	nipponbare_chr08	1582500	1587500
Nipponbare	nipponbare_boundary_1244	nipponbare_chr08	1642500	1762500

Nipponbare	nipponbare_boundary_1245	nipponbare_chr08	3352500	3357500
Nipponbare	nipponbare_boundary_1246	nipponbare_chr08	3417500	3422500
Nipponbare	nipponbare_boundary_1247	nipponbare_chr08	3537500	3542500
Nipponbare	nipponbare_boundary_1248	nipponbare_chr08	3677500	3682500
Nipponbare	nipponbare_boundary_1249	nipponbare_chr08	3747500	3752500
Nipponbare	nipponbare_boundary_1250	nipponbare_chr08	3842500	3847500
Nipponbare	nipponbare_boundary_1251	nipponbare_chr08	3942500	3947500
Nipponbare	nipponbare_boundary_1252	nipponbare_chr08	4012500	4017500
Nipponbare	nipponbare_boundary_1253	nipponbare_chr08	4077500	4082500
Nipponbare	nipponbare_boundary_1254	nipponbare_chr08	4177500	4182500
Nipponbare	nipponbare_boundary_1255	nipponbare_chr08	4237500	4242500
Nipponbare	nipponbare_boundary_1256	nipponbare_chr08	4282500	4287500
Nipponbare	nipponbare_boundary_1257	nipponbare_chr08	4332500	4362500
Nipponbare	nipponbare_boundary_1258	nipponbare_chr08	4432500	4437500
Nipponbare	nipponbare_boundary_1259	nipponbare_chr08	4632500	4677500
Nipponbare	nipponbare_boundary_1260	nipponbare_chr08	5967500	5972500
Nipponbare	nipponbare_boundary_1261	nipponbare_chr08	6047500	6052500
Nipponbare	nipponbare_boundary_1262	nipponbare_chr08	7617500	7622500
Nipponbare	nipponbare_boundary_1263	nipponbare_chr08	7732500	7762500
Nipponbare	nipponbare_boundary_1264	nipponbare_chr08	7907500	7912500
Nipponbare	nipponbare_boundary_1265	nipponbare_chr08	7967500	7972500
Nipponbare	nipponbare_boundary_1266	nipponbare_chr08	8302500	8347500
Nipponbare	nipponbare_boundary_1267	nipponbare_chr08	8482500	8487500
Nipponbare	nipponbare_boundary_1268	nipponbare_chr08	8572500	8577500
Nipponbare	nipponbare_boundary_1269	nipponbare_chr08	8662500	8667500
Nipponbare	nipponbare_boundary_1270	nipponbare_chr08	8767500	8797500
Nipponbare	nipponbare_boundary_1271	nipponbare_chr08	8877500	8882500
Nipponbare	nipponbare_boundary_1272	nipponbare_chr08	8932500	8972500
Nipponbare	nipponbare_boundary_1273	nipponbare_chr08	9042500	9047500
Nipponbare	nipponbare_boundary_1274	nipponbare_chr08	9127500	9132500
Nipponbare	nipponbare_boundary_1275	nipponbare_chr08	9332500	9337500
Nipponbare	nipponbare_boundary_1276	nipponbare_chr08	9387500	9392500

Nipponbare	nipponbare_boundary_1277	nipponbare_chr08	9502500	9507500
Nipponbare	nipponbare_boundary_1278	nipponbare_chr08	10082500	10087500
Nipponbare	nipponbare_boundary_1279	nipponbare_chr08	10137500	10142500
Nipponbare	nipponbare_boundary_1280	nipponbare_chr08	10267500	10272500
Nipponbare	nipponbare_boundary_1281	nipponbare_chr08	10327500	10332500
Nipponbare	nipponbare_boundary_1282	nipponbare_chr08	10492500	10497500
Nipponbare	nipponbare_boundary_1283	nipponbare_chr08	10565000	10570000
Nipponbare	nipponbare_boundary_1284	nipponbare_chr08	10637500	10677500
Nipponbare	nipponbare_boundary_1285	nipponbare_chr08	11402500	11442500
Nipponbare	nipponbare_boundary_1286	nipponbare_chr08	11772500	11777500
Nipponbare	nipponbare_boundary_1287	nipponbare_chr08	11817500	11822500
Nipponbare	nipponbare_boundary_1288	nipponbare_chr08	13392500	13397500
Nipponbare	nipponbare_boundary_1289	nipponbare_chr08	13482500	13487500
Nipponbare	nipponbare_boundary_1290	nipponbare_chr08	14047500	14052500
Nipponbare	nipponbare_boundary_1291	nipponbare_chr08	14192500	14197500
Nipponbare	nipponbare_boundary_1292	nipponbare_chr08	14327500	14332500
Nipponbare	nipponbare_boundary_1293	nipponbare_chr08	15202500	15207500
Nipponbare	nipponbare_boundary_1294	nipponbare_chr08	15272500	15277500
Nipponbare	nipponbare_boundary_1295	nipponbare_chr08	15352500	15357500
Nipponbare	nipponbare_boundary_1296	nipponbare_chr08	15432500	15437500
Nipponbare	nipponbare_boundary_1297	nipponbare_chr08	15977500	15982500
Nipponbare	nipponbare_boundary_1298	nipponbare_chr08	16062500	16067500
Nipponbare	nipponbare_boundary_1299	nipponbare_chr08	16257500	16297500
Nipponbare	nipponbare_boundary_1300	nipponbare_chr08	16512500	16592500
Nipponbare	nipponbare_boundary_1301	nipponbare_chr08	16632500	16637500
Nipponbare	nipponbare_boundary_1302	nipponbare_chr08	16812500	16817500
Nipponbare	nipponbare_boundary_1303	nipponbare_chr08	17812500	17817500
Nipponbare	nipponbare_boundary_1304	nipponbare_chr08	17857500	17862500
Nipponbare	nipponbare_boundary_1305	nipponbare_chr08	18077500	18082500
Nipponbare	nipponbare_boundary_1306	nipponbare_chr08	18167500	18172500
Nipponbare	nipponbare_boundary_1307	nipponbare_chr08	18272500	18277500
Nipponbare	nipponbare_boundary_1308	nipponbare_chr08	19212500	19217500

Nipponbare	nipponbare_boundary_1309	nipponbare_chr08	19337500	19342500
Nipponbare	nipponbare_boundary_1310	nipponbare_chr08	19417500	19422500
Nipponbare	nipponbare_boundary_1311	nipponbare_chr08	19462500	19467500
Nipponbare	nipponbare_boundary_1312	nipponbare_chr08	19517500	19557500
Nipponbare	nipponbare_boundary_1313	nipponbare_chr08	19672500	19677500
Nipponbare	nipponbare_boundary_1314	nipponbare_chr08	19717500	19722500
Nipponbare	nipponbare_boundary_1315	nipponbare_chr08	19792500	19827500
Nipponbare	nipponbare_boundary_1316	nipponbare_chr08	19877500	19912500
Nipponbare	nipponbare_boundary_1317	nipponbare_chr08	20437500	20482500
Nipponbare	nipponbare_boundary_1318	nipponbare_chr08	20557500	20562500
Nipponbare	nipponbare_boundary_1319	nipponbare_chr08	21097500	21102500
Nipponbare	nipponbare_boundary_1320	nipponbare_chr08	21162500	21167500
Nipponbare	nipponbare_boundary_1321	nipponbare_chr08	21217500	21222500
Nipponbare	nipponbare_boundary_1322	nipponbare_chr08	21282500	21322500
Nipponbare	nipponbare_boundary_1323	nipponbare_chr08	21637500	21642500
Nipponbare	nipponbare_boundary_1324	nipponbare_chr08	21707500	21712500
Nipponbare	nipponbare_boundary_1325	nipponbare_chr08	21752500	21757500
Nipponbare	nipponbare_boundary_1326	nipponbare_chr08	21837500	21882500
Nipponbare	nipponbare_boundary_1327	nipponbare_chr08	21942500	21947500
Nipponbare	nipponbare_boundary_1328	nipponbare_chr08	22012500	22017500
Nipponbare	nipponbare_boundary_1329	nipponbare_chr08	22227500	22232500
Nipponbare	nipponbare_boundary_1330	nipponbare_chr08	22327500	22372500
Nipponbare	nipponbare_boundary_1331	nipponbare_chr08	22717500	22722500
Nipponbare	nipponbare_boundary_1332	nipponbare_chr08	22772500	22777500
Nipponbare	nipponbare_boundary_1333	nipponbare_chr08	22977500	22982500
Nipponbare	nipponbare_boundary_1334	nipponbare_chr08	23042500	23047500
Nipponbare	nipponbare_boundary_1335	nipponbare_chr08	23152500	23157500
Nipponbare	nipponbare_boundary_1336	nipponbare_chr08	23202500	23207500
Nipponbare	nipponbare_boundary_1337	nipponbare_chr08	23272500	23277500
Nipponbare	nipponbare_boundary_1338	nipponbare_chr08	23332500	23337500
Nipponbare	nipponbare_boundary_1339	nipponbare_chr08	23382500	23387500
Nipponbare	nipponbare_boundary_1340	nipponbare_chr08	24097500	24132500

Nipponbare	nipponbare_boundary_1341	nipponbare_chr08	24177500	24182500
Nipponbare	nipponbare_boundary_1342	nipponbare_chr08	24262500	24267500
Nipponbare	nipponbare_boundary_1343	nipponbare_chr08	24452500	24457500
Nipponbare	nipponbare_boundary_1344	nipponbare_chr08	24537500	24542500
Nipponbare	nipponbare_boundary_1345	nipponbare_chr08	25362500	25367500
Nipponbare	nipponbare_boundary_1346	nipponbare_chr08	25432500	25437500
Nipponbare	nipponbare_boundary_1347	nipponbare_chr08	25492500	25497500
Nipponbare	nipponbare_boundary_1348	nipponbare_chr08	25575000	25580000
Nipponbare	nipponbare_boundary_1349	nipponbare_chr08	25722500	25727500
Nipponbare	nipponbare_boundary_1350	nipponbare_chr08	25797500	25802500
Nipponbare	nipponbare_boundary_1351	nipponbare_chr08	25852500	25857500
Nipponbare	nipponbare_boundary_1352	nipponbare_chr08	25897500	25902500
Nipponbare	nipponbare_boundary_1353	nipponbare_chr08	25977500	25982500
Nipponbare	nipponbare_boundary_1354	nipponbare_chr08	26087500	26092500
Nipponbare	nipponbare_boundary_1355	nipponbare_chr08	26132500	26137500
Nipponbare	nipponbare_boundary_1356	nipponbare_chr08	26202500	26207500
Nipponbare	nipponbare_boundary_1357	nipponbare_chr08	26297500	26302500
Nipponbare	nipponbare_boundary_1358	nipponbare_chr08	26387500	26392500
Nipponbare	nipponbare_boundary_1359	nipponbare_chr08	26447500	26452500
Nipponbare	nipponbare_boundary_1360	nipponbare_chr08	26527500	26532500
Nipponbare	nipponbare_boundary_1361	nipponbare_chr08	26742500	26747500
Nipponbare	nipponbare_boundary_1362	nipponbare_chr08	26792500	26797500
Nipponbare	nipponbare_boundary_1363	nipponbare_chr08	26877500	26882500
Nipponbare	nipponbare_boundary_1364	nipponbare_chr08	26932500	26937500
Nipponbare	nipponbare_boundary_1365	nipponbare_chr08	27032500	27037500
Nipponbare	nipponbare_boundary_1366	nipponbare_chr08	27352500	27357500
Nipponbare	nipponbare_boundary_1367	nipponbare_chr08	27397500	27402500
Nipponbare	nipponbare_boundary_1368	nipponbare_chr08	27492500	27497500
Nipponbare	nipponbare_boundary_1369	nipponbare_chr08	27592500	27597500
Nipponbare	nipponbare_boundary_1370	nipponbare_chr08	27697500	27702500
Nipponbare	nipponbare_boundary_1371	nipponbare_chr08	27827500	27832500
Nipponbare	nipponbare_boundary_1372	nipponbare_chr08	27962500	27967500

Nipponbare	nipponbare_boundary_1373	nipponbare_chr08	28007500	28012500
Nipponbare	nipponbare_boundary_1374	nipponbare_chr08	28067500	28072500
Nipponbare	nipponbare_boundary_1375	nipponbare_chr08	28127500	28132500
Nipponbare	nipponbare_boundary_1376	nipponbare_chr08	28212500	28217500
Nipponbare	nipponbare_boundary_1377	nipponbare_chr08	28272500	28277500
Nipponbare	nipponbare_boundary_1378	nipponbare_chr08	28342500	28347500
Nipponbare	nipponbare_boundary_1379	nipponbare_chr09	1162500	1167500
Nipponbare	nipponbare_boundary_1380	nipponbare_chr09	1212500	1255000
Nipponbare	nipponbare_boundary_1381	nipponbare_chr09	1312500	1317500
Nipponbare	nipponbare_boundary_1382	nipponbare_chr09	1367500	1372500
Nipponbare	nipponbare_boundary_1383	nipponbare_chr09	1492500	1497500
Nipponbare	nipponbare_boundary_1384	nipponbare_chr09	1662500	1667500
Nipponbare	nipponbare_boundary_1385	nipponbare_chr09	1777500	1817500
Nipponbare	nipponbare_boundary_1386	nipponbare_chr09	1912500	1917500
Nipponbare	nipponbare_boundary_1387	nipponbare_chr09	1970000	1975000
Nipponbare	nipponbare_boundary_1388	nipponbare_chr09	2032500	2037500
Nipponbare	nipponbare_boundary_1389	nipponbare_chr09	2122500	2127500
Nipponbare	nipponbare_boundary_1390	nipponbare_chr09	2280000	2285000
Nipponbare	nipponbare_boundary_1391	nipponbare_chr09	2357500	2397500
Nipponbare	nipponbare_boundary_1392	nipponbare_chr09	2452500	2457500
Nipponbare	nipponbare_boundary_1393	nipponbare_chr09	3407500	3412500
Nipponbare	nipponbare_boundary_1394	nipponbare_chr09	3492500	3497500
Nipponbare	nipponbare_boundary_1395	nipponbare_chr09	3547500	3587500
Nipponbare	nipponbare_boundary_1396	nipponbare_chr09	3742500	3747500
Nipponbare	nipponbare_boundary_1397	nipponbare_chr09	3827500	3832500
Nipponbare	nipponbare_boundary_1398	nipponbare_chr09	4222500	4227500
Nipponbare	nipponbare_boundary_1399	nipponbare_chr09	4342500	4347500
Nipponbare	nipponbare_boundary_1400	nipponbare_chr09	4392500	4397500
Nipponbare	nipponbare_boundary_1401	nipponbare_chr09	5097500	5102500
Nipponbare	nipponbare_boundary_1402	nipponbare_chr09	5152500	5157500
Nipponbare	nipponbare_boundary_1403	nipponbare_chr09	7252500	7257500
Nipponbare	nipponbare_boundary_1404	nipponbare_chr09	7352500	7357500

Nipponbare	nipponbare_boundary_1405	nipponbare_chr09	7642500	7647500
Nipponbare	nipponbare_boundary_1406	nipponbare_chr09	7747500	7752500
Nipponbare	nipponbare_boundary_1407	nipponbare_chr09	7792500	7797500
Nipponbare	nipponbare_boundary_1408	nipponbare_chr09	8167500	8172500
Nipponbare	nipponbare_boundary_1409	nipponbare_chr09	8212500	8217500
Nipponbare	nipponbare_boundary_1410	nipponbare_chr09	8417500	8422500
Nipponbare	nipponbare_boundary_1411	nipponbare_chr09	8607500	8612500
Nipponbare	nipponbare_boundary_1412	nipponbare_chr09	8727500	8732500
Nipponbare	nipponbare_boundary_1413	nipponbare_chr09	9447500	9452500
Nipponbare	nipponbare_boundary_1414	nipponbare_chr09	9512500	9517500
Nipponbare	nipponbare_boundary_1415	nipponbare_chr09	10347500	10352500
Nipponbare	nipponbare_boundary_1416	nipponbare_chr09	10487500	10492500
Nipponbare	nipponbare_boundary_1417	nipponbare_chr09	10737500	10742500
Nipponbare	nipponbare_boundary_1418	nipponbare_chr09	11777500	11782500
Nipponbare	nipponbare_boundary_1419	nipponbare_chr09	11877500	11882500
Nipponbare	nipponbare_boundary_1420	nipponbare_chr09	12020000	12025000
Nipponbare	nipponbare_boundary_1421	nipponbare_chr09	12087500	12122500
Nipponbare	nipponbare_boundary_1422	nipponbare_chr09	12377500	12382500
Nipponbare	nipponbare_boundary_1423	nipponbare_chr09	12442500	12447500
Nipponbare	nipponbare_boundary_1424	nipponbare_chr09	12542500	12547500
Nipponbare	nipponbare_boundary_1425	nipponbare_chr09	12627500	12632500
Nipponbare	nipponbare_boundary_1426	nipponbare_chr09	12727500	12732500
Nipponbare	nipponbare_boundary_1427	nipponbare_chr09	12772500	12777500
Nipponbare	nipponbare_boundary_1428	nipponbare_chr09	13562500	13607500
Nipponbare	nipponbare_boundary_1429	nipponbare_chr09	13712500	13717500
Nipponbare	nipponbare_boundary_1430	nipponbare_chr09	13772500	13777500
Nipponbare	nipponbare_boundary_1431	nipponbare_chr09	13842500	13847500
Nipponbare	nipponbare_boundary_1432	nipponbare_chr09	13907500	13912500
Nipponbare	nipponbare_boundary_1433	nipponbare_chr09	13977500	14092500
Nipponbare	nipponbare_boundary_1434	nipponbare_chr09	14827500	14867500
Nipponbare	nipponbare_boundary_1435	nipponbare_chr09	14932500	14937500
Nipponbare	nipponbare_boundary_1436	nipponbare_chr09	15397500	15437500

Nipponbare	nipponbare_boundary_1437	nipponbare_chr09	15477500	15482500
Nipponbare	nipponbare_boundary_1438	nipponbare_chr09	16292500	16297500
Nipponbare	nipponbare_boundary_1439	nipponbare_chr09	16347500	16352500
Nipponbare	nipponbare_boundary_1440	nipponbare_chr09	16407500	16412500
Nipponbare	nipponbare_boundary_1441	nipponbare_chr09	16782500	16787500
Nipponbare	nipponbare_boundary_1442	nipponbare_chr09	16827500	16832500
Nipponbare	nipponbare_boundary_1443	nipponbare_chr09	17337500	17342500
Nipponbare	nipponbare_boundary_1444	nipponbare_chr09	17402500	17407500
Nipponbare	nipponbare_boundary_1445	nipponbare_chr09	17772500	17777500
Nipponbare	nipponbare_boundary_1446	nipponbare_chr09	17832500	17837500
Nipponbare	nipponbare_boundary_1447	nipponbare_chr09	17877500	17912500
Nipponbare	nipponbare_boundary_1448	nipponbare_chr09	17982500	18017500
Nipponbare	nipponbare_boundary_1449	nipponbare_chr09	18057500	18062500
Nipponbare	nipponbare_boundary_1450	nipponbare_chr09	18242500	18247500
Nipponbare	nipponbare_boundary_1451	nipponbare_chr09	18287500	18292500
Nipponbare	nipponbare_boundary_1452	nipponbare_chr09	18367500	18412500
Nipponbare	nipponbare_boundary_1453	nipponbare_chr09	18452500	18457500
Nipponbare	nipponbare_boundary_1454	nipponbare_chr09	18667500	18672500
Nipponbare	nipponbare_boundary_1455	nipponbare_chr09	18747500	18752500
Nipponbare	nipponbare_boundary_1456	nipponbare_chr09	18937500	18942500
Nipponbare	nipponbare_boundary_1457	nipponbare_chr09	18982500	18987500
Nipponbare	nipponbare_boundary_1458	nipponbare_chr09	19112500	19117500
Nipponbare	nipponbare_boundary_1459	nipponbare_chr09	19157500	19162500
Nipponbare	nipponbare_boundary_1460	nipponbare_chr09	19682500	19687500
Nipponbare	nipponbare_boundary_1461	nipponbare_chr09	19797500	19802500
Nipponbare	nipponbare_boundary_1462	nipponbare_chr09	19852500	19857500
Nipponbare	nipponbare_boundary_1463	nipponbare_chr09	19942500	19947500
Nipponbare	nipponbare_boundary_1464	nipponbare_chr09	20007500	20012500
Nipponbare	nipponbare_boundary_1465	nipponbare_chr09	20152500	20157500
Nipponbare	nipponbare_boundary_1466	nipponbare_chr09	20252500	20257500
Nipponbare	nipponbare_boundary_1467	nipponbare_chr09	20522500	20527500
Nipponbare	nipponbare_boundary_1468	nipponbare_chr09	20607500	20612500

Nipponbare	nipponbare_boundary_1469	nipponbare_chr09	20742500	20747500
Nipponbare	nipponbare_boundary_1470	nipponbare_chr09	20797500	20802500
Nipponbare	nipponbare_boundary_1471	nipponbare_chr09	20857500	20862500
Nipponbare	nipponbare_boundary_1472	nipponbare_chr09	20937500	20942500
Nipponbare	nipponbare_boundary_1473	nipponbare_chr09	20982500	20987500
Nipponbare	nipponbare_boundary_1474	nipponbare_chr09	21052500	21057500
Nipponbare	nipponbare_boundary_1475	nipponbare_chr09	21262500	21267500
Nipponbare	nipponbare_boundary_1476	nipponbare_chr09	21332500	21337500
Nipponbare	nipponbare_boundary_1477	nipponbare_chr09	21402500	21407500
Nipponbare	nipponbare_boundary_1478	nipponbare_chr09	21517500	21522500
Nipponbare	nipponbare_boundary_1479	nipponbare_chr09	21582500	21587500
Nipponbare	nipponbare_boundary_1480	nipponbare_chr09	21757500	21762500
Nipponbare	nipponbare_boundary_1481	nipponbare_chr09	21882500	21887500
Nipponbare	nipponbare_boundary_1482	nipponbare_chr09	22197500	22202500
Nipponbare	nipponbare_boundary_1483	nipponbare_chr09	22252500	22257500
Nipponbare	nipponbare_boundary_1484	nipponbare_chr10	347500	387500
Nipponbare	nipponbare_boundary_1485	nipponbare_chr10	505000	510000
Nipponbare	nipponbare_boundary_1486	nipponbare_chr10	565000	570000
Nipponbare	nipponbare_boundary_1487	nipponbare_chr10	677500	682500
Nipponbare	nipponbare_boundary_1488	nipponbare_chr10	852500	857500
Nipponbare	nipponbare_boundary_1489	nipponbare_chr10	927500	932500
Nipponbare	nipponbare_boundary_1490	nipponbare_chr10	1122500	1127500
Nipponbare	nipponbare_boundary_1491	nipponbare_chr10	1192500	1232500
Nipponbare	nipponbare_boundary_1492	nipponbare_chr10	1302500	1307500
Nipponbare	nipponbare_boundary_1493	nipponbare_chr10	1432500	1437500
Nipponbare	nipponbare_boundary_1494	nipponbare_chr10	1527500	1532500
Nipponbare	nipponbare_boundary_1495	nipponbare_chr10	1657500	1662500
Nipponbare	nipponbare_boundary_1496	nipponbare_chr10	1712500	1717500
Nipponbare	nipponbare_boundary_1497	nipponbare_chr10	1777500	1782500
Nipponbare	nipponbare_boundary_1498	nipponbare_chr10	1872500	1917500
Nipponbare	nipponbare_boundary_1499	nipponbare_chr10	2182500	2187500
Nipponbare	nipponbare_boundary_1500	nipponbare_chr10	2232500	2237500

Nipponbare	nipponbare_boundary_1501	nipponbare_chr10	2512500	2517500
Nipponbare	nipponbare_boundary_1502	nipponbare_chr10	2557500	2562500
Nipponbare	nipponbare_boundary_1503	nipponbare_chr10	2687500	2692500
Nipponbare	nipponbare_boundary_1504	nipponbare_chr10	3732500	3737500
Nipponbare	nipponbare_boundary_1505	nipponbare_chr10	3787500	3792500
Nipponbare	nipponbare_boundary_1506	nipponbare_chr10	3837500	3842500
Nipponbare	nipponbare_boundary_1507	nipponbare_chr10	3892500	3897500
Nipponbare	nipponbare_boundary_1508	nipponbare_chr10	3982500	3987500
Nipponbare	nipponbare_boundary_1509	nipponbare_chr10	4677500	4682500
Nipponbare	nipponbare_boundary_1510	nipponbare_chr10	4747500	4752500
Nipponbare	nipponbare_boundary_1511	nipponbare_chr10	4937500	4972500
Nipponbare	nipponbare_boundary_1512	nipponbare_chr10	5022500	5027500
Nipponbare	nipponbare_boundary_1513	nipponbare_chr10	5512500	5517500
Nipponbare	nipponbare_boundary_1514	nipponbare_chr10	5572500	5577500
Nipponbare	nipponbare_boundary_1515	nipponbare_chr10	6742500	6747500
Nipponbare	nipponbare_boundary_1516	nipponbare_chr10	6877500	6882500
Nipponbare	nipponbare_boundary_1517	nipponbare_chr10	8837500	8842500
Nipponbare	nipponbare_boundary_1518	nipponbare_chr10	8912500	8917500
Nipponbare	nipponbare_boundary_1519	nipponbare_chr10	9265000	9270000
Nipponbare	nipponbare_boundary_1520	nipponbare_chr10	9342500	9347500
Nipponbare	nipponbare_boundary_1521	nipponbare_chr10	9897500	9937500
Nipponbare	nipponbare_boundary_1522	nipponbare_chr10	11002500	11007500
Nipponbare	nipponbare_boundary_1523	nipponbare_chr10	11052500	11057500
Nipponbare	nipponbare_boundary_1524	nipponbare_chr10	11187500	11192500
Nipponbare	nipponbare_boundary_1525	nipponbare_chr10	11247500	11252500
Nipponbare	nipponbare_boundary_1526	nipponbare_chr10	11397500	11402500
Nipponbare	nipponbare_boundary_1527	nipponbare_chr10	11557500	11562500
Nipponbare	nipponbare_boundary_1528	nipponbare_chr10	12727500	12732500
Nipponbare	nipponbare_boundary_1529	nipponbare_chr10	12862500	12867500
Nipponbare	nipponbare_boundary_1530	nipponbare_chr10	13012500	13052500
Nipponbare	nipponbare_boundary_1531	nipponbare_chr10	13092500	13097500
Nipponbare	nipponbare_boundary_1532	nipponbare_chr10	13857500	13862500

Nipponbare	nipponbare_boundary_1533	nipponbare_chr10	13937500	13942500
Nipponbare	nipponbare_boundary_1534	nipponbare_chr10	14257500	14262500
Nipponbare	nipponbare_boundary_1535	nipponbare_chr10	14322500	14327500
Nipponbare	nipponbare_boundary_1536	nipponbare_chr10	14432500	14437500
Nipponbare	nipponbare_boundary_1537	nipponbare_chr10	14477500	14482500
Nipponbare	nipponbare_boundary_1538	nipponbare_chr10	14552500	14557500
Nipponbare	nipponbare_boundary_1539	nipponbare_chr10	14747500	14787500
Nipponbare	nipponbare_boundary_1540	nipponbare_chr10	15212500	15252500
Nipponbare	nipponbare_boundary_1541	nipponbare_chr10	15332500	15450000
Nipponbare	nipponbare_boundary_1542	nipponbare_chr10	15522500	15527500
Nipponbare	nipponbare_boundary_1543	nipponbare_chr10	15612500	15617500
Nipponbare	nipponbare_boundary_1544	nipponbare_chr10	15737500	15742500
Nipponbare	nipponbare_boundary_1545	nipponbare_chr10	15817500	15822500
Nipponbare	nipponbare_boundary_1546	nipponbare_chr10	17352500	17357500
Nipponbare	nipponbare_boundary_1547	nipponbare_chr10	17425000	17430000
Nipponbare	nipponbare_boundary_1548	nipponbare_chr10	17612500	17617500
Nipponbare	nipponbare_boundary_1549	nipponbare_chr10	17682500	17722500
Nipponbare	nipponbare_boundary_1550	nipponbare_chr10	17802500	17807500
Nipponbare	nipponbare_boundary_1551	nipponbare_chr10	17852500	17857500
Nipponbare	nipponbare_boundary_1552	nipponbare_chr10	17962500	18007500
Nipponbare	nipponbare_boundary_1553	nipponbare_chr10	18092500	18097500
Nipponbare	nipponbare_boundary_1554	nipponbare_chr10	18147500	18152500
Nipponbare	nipponbare_boundary_1555	nipponbare_chr10	18192500	18197500
Nipponbare	nipponbare_boundary_1556	nipponbare_chr10	18357500	18362500
Nipponbare	nipponbare_boundary_1557	nipponbare_chr10	18407500	18412500
Nipponbare	nipponbare_boundary_1558	nipponbare_chr10	18742500	18747500
Nipponbare	nipponbare_boundary_1559	nipponbare_chr10	18792500	18797500
Nipponbare	nipponbare_boundary_1560	nipponbare_chr10	19217500	19262500
Nipponbare	nipponbare_boundary_1561	nipponbare_chr10	19347500	19412500
Nipponbare	nipponbare_boundary_1562	nipponbare_chr10	19522500	19527500
Nipponbare	nipponbare_boundary_1563	nipponbare_chr10	19597500	19602500
Nipponbare	nipponbare_boundary_1564	nipponbare_chr10	19672500	19782500

Nipponbare	nipponbare_boundary_1565	nipponbare_chr10	19877500	19917500
Nipponbare	nipponbare_boundary_1566	nipponbare_chr10	19957500	19962500
Nipponbare	nipponbare_boundary_1567	nipponbare_chr10	20027500	20072500
Nipponbare	nipponbare_boundary_1568	nipponbare_chr10	20712500	20717500
Nipponbare	nipponbare_boundary_1569	nipponbare_chr10	20782500	20787500
Nipponbare	nipponbare_boundary_1570	nipponbare_chr10	20852500	20857500
Nipponbare	nipponbare_boundary_1571	nipponbare_chr10	20897500	20937500
Nipponbare	nipponbare_boundary_1572	nipponbare_chr10	21017500	21022500
Nipponbare	nipponbare_boundary_1573	nipponbare_chr10	21077500	21082500
Nipponbare	nipponbare_boundary_1574	nipponbare_chr10	21462500	21467500
Nipponbare	nipponbare_boundary_1575	nipponbare_chr10	21587500	21592500
Nipponbare	nipponbare_boundary_1576	nipponbare_chr10	21702500	21707500
Nipponbare	nipponbare_boundary_1577	nipponbare_chr10	21747500	21752500
Nipponbare	nipponbare_boundary_1578	nipponbare_chr10	22677500	22682500
Nipponbare	nipponbare_boundary_1579	nipponbare_chr10	22747500	22752500
Nipponbare	nipponbare_boundary_1580	nipponbare_chr10	22817500	22822500
Nipponbare	nipponbare_boundary_1581	nipponbare_chr10	22862500	22867500
Nipponbare	nipponbare_boundary_1582	nipponbare_chr11	237500	242500
Nipponbare	nipponbare_boundary_1583	nipponbare_chr11	295000	300000
Nipponbare	nipponbare_boundary_1584	nipponbare_chr11	357500	362500
Nipponbare	nipponbare_boundary_1585	nipponbare_chr11	2117500	2152500
Nipponbare	nipponbare_boundary_1586	nipponbare_chr11	3082500	3187500
Nipponbare	nipponbare_boundary_1587	nipponbare_chr11	3252500	3257500
Nipponbare	nipponbare_boundary_1588	nipponbare_chr11	3362500	3367500
Nipponbare	nipponbare_boundary_1589	nipponbare_chr11	3425000	3430000
Nipponbare	nipponbare_boundary_1590	nipponbare_chr11	3672500	3677500
Nipponbare	nipponbare_boundary_1591	nipponbare_chr11	3742500	3747500
Nipponbare	nipponbare_boundary_1592	nipponbare_chr11	3807500	3812500
Nipponbare	nipponbare_boundary_1593	nipponbare_chr11	3852500	3857500
Nipponbare	nipponbare_boundary_1594	nipponbare_chr11	4012500	4017500
Nipponbare	nipponbare_boundary_1595	nipponbare_chr11	4097500	4102500
Nipponbare	nipponbare_boundary_1596	nipponbare_chr11	4855000	4860000

Nipponbare	nipponbare_boundary_1597	nipponbare_chr11	4927500	4932500
Nipponbare	nipponbare_boundary_1598	nipponbare_chr11	4992500	4997500
Nipponbare	nipponbare_boundary_1599	nipponbare_chr11	6222500	6227500
Nipponbare	nipponbare_boundary_1600	nipponbare_chr11	6322500	6327500
Nipponbare	nipponbare_boundary_1601	nipponbare_chr11	7347500	7352500
Nipponbare	nipponbare_boundary_1602	nipponbare_chr11	7432500	7437500
Nipponbare	nipponbare_boundary_1603	nipponbare_chr11	7592500	7597500
Nipponbare	nipponbare_boundary_1604	nipponbare_chr11	7657500	7662500
Nipponbare	nipponbare_boundary_1605	nipponbare_chr11	7857500	7862500
Nipponbare	nipponbare_boundary_1606	nipponbare_chr11	7907500	7912500
Nipponbare	nipponbare_boundary_1607	nipponbare_chr11	8922500	8927500
Nipponbare	nipponbare_boundary_1608	nipponbare_chr11	9107500	9112500
Nipponbare	nipponbare_boundary_1609	nipponbare_chr11	9772500	9777500
Nipponbare	nipponbare_boundary_1610	nipponbare_chr11	9857500	9862500
Nipponbare	nipponbare_boundary_1611	nipponbare_chr11	10652500	10657500
Nipponbare	nipponbare_boundary_1612	nipponbare_chr11	10732500	10737500
Nipponbare	nipponbare_boundary_1613	nipponbare_chr11	10817500	10822500
Nipponbare	nipponbare_boundary_1614	nipponbare_chr11	10907500	10912500
Nipponbare	nipponbare_boundary_1615	nipponbare_chr11	11017500	11022500
Nipponbare	nipponbare_boundary_1616	nipponbare_chr11	11197500	11202500
Nipponbare	nipponbare_boundary_1617	nipponbare_chr11	11802500	11807500
Nipponbare	nipponbare_boundary_1618	nipponbare_chr11	11907500	11912500
Nipponbare	nipponbare_boundary_1619	nipponbare_chr11	13527500	13532500
Nipponbare	nipponbare_boundary_1620	nipponbare_chr11	13632500	13637500
Nipponbare	nipponbare_boundary_1621	nipponbare_chr11	13712500	13717500
Nipponbare	nipponbare_boundary_1622	nipponbare_chr11	14592500	14597500
Nipponbare	nipponbare_boundary_1623	nipponbare_chr11	14682500	14687500
Nipponbare	nipponbare_boundary_1624	nipponbare_chr11	14742500	14747500
Nipponbare	nipponbare_boundary_1625	nipponbare_chr11	14827500	14832500
Nipponbare	nipponbare_boundary_1626	nipponbare_chr11	15417500	15422500
Nipponbare	nipponbare_boundary_1627	nipponbare_chr11	15547500	15552500
Nipponbare	nipponbare_boundary_1628	nipponbare_chr11	15667500	15672500

Nipponbare	nipponbare_boundary_1629	nipponbare_chr11	15787500	15792500
Nipponbare	nipponbare_boundary_1630	nipponbare_chr11	17042500	17077500
Nipponbare	nipponbare_boundary_1631	nipponbare_chr11	17427500	17432500
Nipponbare	nipponbare_boundary_1632	nipponbare_chr11	17487500	17492500
Nipponbare	nipponbare_boundary_1633	nipponbare_chr11	17627500	17672500
Nipponbare	nipponbare_boundary_1634	nipponbare_chr11	17732500	17737500
Nipponbare	nipponbare_boundary_1635	nipponbare_chr11	17807500	17812500
Nipponbare	nipponbare_boundary_1636	nipponbare_chr11	18777500	18782500
Nipponbare	nipponbare_boundary_1637	nipponbare_chr11	18840000	18845000
Nipponbare	nipponbare_boundary_1638	nipponbare_chr11	19427500	19432500
Nipponbare	nipponbare_boundary_1639	nipponbare_chr11	19522500	19527500
Nipponbare	nipponbare_boundary_1640	nipponbare_chr11	19577500	19582500
Nipponbare	nipponbare_boundary_1641	nipponbare_chr11	19627500	19632500
Nipponbare	nipponbare_boundary_1642	nipponbare_chr11	19702500	19707500
Nipponbare	nipponbare_boundary_1643	nipponbare_chr11	19872500	19877500
Nipponbare	nipponbare_boundary_1644	nipponbare_chr11	19942500	19947500
Nipponbare	nipponbare_boundary_1645	nipponbare_chr11	20127500	20132500
Nipponbare	nipponbare_boundary_1646	nipponbare_chr11	20317500	20322500
Nipponbare	nipponbare_boundary_1647	nipponbare_chr11	20367500	20372500
Nipponbare	nipponbare_boundary_1648	nipponbare_chr11	20417500	20422500
Nipponbare	nipponbare_boundary_1649	nipponbare_chr11	20487500	20492500
Nipponbare	nipponbare_boundary_1650	nipponbare_chr11	20537500	20542500
Nipponbare	nipponbare_boundary_1651	nipponbare_chr11	20582500	20587500
Nipponbare	nipponbare_boundary_1652	nipponbare_chr11	21177500	21182500
Nipponbare	nipponbare_boundary_1653	nipponbare_chr11	21252500	21257500
Nipponbare	nipponbare_boundary_1654	nipponbare_chr11	21337500	21342500
Nipponbare	nipponbare_boundary_1655	nipponbare_chr11	21492500	21497500
Nipponbare	nipponbare_boundary_1656	nipponbare_chr11	21552500	21557500
Nipponbare	nipponbare_boundary_1657	nipponbare_chr11	21732500	21737500
Nipponbare	nipponbare_boundary_1658	nipponbare_chr11	22527500	22612500
Nipponbare	nipponbare_boundary_1659	nipponbare_chr11	23177500	23182500
Nipponbare	nipponbare_boundary_1660	nipponbare_chr11	23232500	23237500

Nipponbare	nipponbare_boundary_1661	nipponbare_chr11	23297500	23302500
Nipponbare	nipponbare_boundary_1662	nipponbare_chr11	23347500	23352500
Nipponbare	nipponbare_boundary_1663	nipponbare_chr11	23442500	23447500
Nipponbare	nipponbare_boundary_1664	nipponbare_chr11	23832500	23837500
Nipponbare	nipponbare_boundary_1665	nipponbare_chr11	23897500	23937500
Nipponbare	nipponbare_boundary_1666	nipponbare_chr11	23992500	23997500
Nipponbare	nipponbare_boundary_1667	nipponbare_chr11	24162500	24167500
Nipponbare	nipponbare_boundary_1668	nipponbare_chr11	24237500	24242500
Nipponbare	nipponbare_boundary_1669	nipponbare_chr11	24392500	24397500
Nipponbare	nipponbare_boundary_1670	nipponbare_chr11	24447500	24452500
Nipponbare	nipponbare_boundary_1671	nipponbare_chr11	24977500	24982500
Nipponbare	nipponbare_boundary_1672	nipponbare_chr11	25152500	25157500
Nipponbare	nipponbare_boundary_1673	nipponbare_chr11	25577500	25582500
Nipponbare	nipponbare_boundary_1674	nipponbare_chr11	25627500	25632500
Nipponbare	nipponbare_boundary_1675	nipponbare_chr11	26137500	26142500
Nipponbare	nipponbare_boundary_1676	nipponbare_chr11	26217500	26222500
Nipponbare	nipponbare_boundary_1677	nipponbare_chr11	26667500	26672500
Nipponbare	nipponbare_boundary_1678	nipponbare_chr11	26737500	26742500
Nipponbare	nipponbare_boundary_1679	nipponbare_chr11	27217500	27222500
Nipponbare	nipponbare_boundary_1680	nipponbare_chr11	27345000	27350000
Nipponbare	nipponbare_boundary_1681	nipponbare_chr11	27712500	27717500
Nipponbare	nipponbare_boundary_1682	nipponbare_chr11	27792500	27797500
Nipponbare	nipponbare_boundary_1683	nipponbare_chr11	27842500	27847500
Nipponbare	nipponbare_boundary_1684	nipponbare_chr11	28462500	28467500
Nipponbare	nipponbare_boundary_1685	nipponbare_chr11	28717500	28722500
Nipponbare	nipponbare_boundary_1686	nipponbare_chr11	28767500	28812500
Nipponbare	nipponbare_boundary_1687	nipponbare_chr12	802500	847500
Nipponbare	nipponbare_boundary_1688	nipponbare_chr12	892500	897500
Nipponbare	nipponbare_boundary_1689	nipponbare_chr12	1167500	1172500
Nipponbare	nipponbare_boundary_1690	nipponbare_chr12	1297500	1302500
Nipponbare	nipponbare_boundary_1691	nipponbare_chr12	1357500	1362500
Nipponbare	nipponbare_boundary_1692	nipponbare_chr12	1592500	1597500

Nipponbare	nipponbare_boundary_1693	nipponbare_chr12	1647500	1652500
Nipponbare	nipponbare_boundary_1694	nipponbare_chr12	1747500	1752500
Nipponbare	nipponbare_boundary_1695	nipponbare_chr12	1802500	1807500
Nipponbare	nipponbare_boundary_1696	nipponbare_chr12	1887500	1892500
Nipponbare	nipponbare_boundary_1697	nipponbare_chr12	1932500	1937500
Nipponbare	nipponbare_boundary_1698	nipponbare_chr12	2247500	2252500
Nipponbare	nipponbare_boundary_1699	nipponbare_chr12	2312500	2317500
Nipponbare	nipponbare_boundary_1700	nipponbare_chr12	2387500	2392500
Nipponbare	nipponbare_boundary_1701	nipponbare_chr12	2447500	2452500
Nipponbare	nipponbare_boundary_1702	nipponbare_chr12	2497500	2502500
Nipponbare	nipponbare_boundary_1703	nipponbare_chr12	2597500	2602500
Nipponbare	nipponbare_boundary_1704	nipponbare_chr12	2882500	2927500
Nipponbare	nipponbare_boundary_1705	nipponbare_chr12	2967500	2972500
Nipponbare	nipponbare_boundary_1706	nipponbare_chr12	3352500	3357500
Nipponbare	nipponbare_boundary_1707	nipponbare_chr12	3422500	3427500
Nipponbare	nipponbare_boundary_1708	nipponbare_chr12	3482500	3487500
Nipponbare	nipponbare_boundary_1709	nipponbare_chr12	3582500	3587500
Nipponbare	nipponbare_boundary_1710	nipponbare_chr12	3632500	3637500
Nipponbare	nipponbare_boundary_1711	nipponbare_chr12	3882500	3887500
Nipponbare	nipponbare_boundary_1712	nipponbare_chr12	3937500	3977500
Nipponbare	nipponbare_boundary_1713	nipponbare_chr12	4032500	4037500
Nipponbare	nipponbare_boundary_1714	nipponbare_chr12	4182500	4222500
Nipponbare	nipponbare_boundary_1715	nipponbare_chr12	4397500	4402500
Nipponbare	nipponbare_boundary_1716	nipponbare_chr12	4467500	4472500
Nipponbare	nipponbare_boundary_1717	nipponbare_chr12	4552500	4557500
Nipponbare	nipponbare_boundary_1718	nipponbare_chr12	4632500	4637500
Nipponbare	nipponbare_boundary_1719	nipponbare_chr12	4742500	4747500
Nipponbare	nipponbare_boundary_1720	nipponbare_chr12	5177500	5182500
Nipponbare	nipponbare_boundary_1721	nipponbare_chr12	5387500	5392500
Nipponbare	nipponbare_boundary_1722	nipponbare_chr12	5467500	5472500
Nipponbare	nipponbare_boundary_1723	nipponbare_chr12	5537500	5542500
Nipponbare	nipponbare_boundary_1724	nipponbare_chr12	7287500	7317500

Nipponbare	nipponbare_boundary_1725	nipponbare_chr12	7382500	7387500
Nipponbare	nipponbare_boundary_1726	nipponbare_chr12	7562500	7567500
Nipponbare	nipponbare_boundary_1727	nipponbare_chr12	7642500	7647500
Nipponbare	nipponbare_boundary_1728	nipponbare_chr12	7842500	7877500
Nipponbare	nipponbare_boundary_1729	nipponbare_chr12	7927500	7932500
Nipponbare	nipponbare_boundary_1730	nipponbare_chr12	8532500	8537500
Nipponbare	nipponbare_boundary_1731	nipponbare_chr12	8662500	8667500
Nipponbare	nipponbare_boundary_1732	nipponbare_chr12	8817500	8862500
Nipponbare	nipponbare_boundary_1733	nipponbare_chr12	9362500	9367500
Nipponbare	nipponbare_boundary_1734	nipponbare_chr12	9432500	9437500
Nipponbare	nipponbare_boundary_1735	nipponbare_chr12	9492500	9497500
Nipponbare	nipponbare_boundary_1736	nipponbare_chr12	9567500	9572500
Nipponbare	nipponbare_boundary_1737	nipponbare_chr12	9802500	9807500
Nipponbare	nipponbare_boundary_1738	nipponbare_chr12	9917500	9922500
Nipponbare	nipponbare_boundary_1739	nipponbare_chr12	10142500	10147500
Nipponbare	nipponbare_boundary_1740	nipponbare_chr12	10192500	10197500
Nipponbare	nipponbare_boundary_1741	nipponbare_chr12	10462500	10467500
Nipponbare	nipponbare_boundary_1742	nipponbare_chr12	10547500	10552500
Nipponbare	nipponbare_boundary_1743	nipponbare_chr12	10722500	10727500
Nipponbare	nipponbare_boundary_1744	nipponbare_chr12	10777500	10782500
Nipponbare	nipponbare_boundary_1745	nipponbare_chr12	11257500	11262500
Nipponbare	nipponbare_boundary_1746	nipponbare_chr12	11317500	11352500
Nipponbare	nipponbare_boundary_1747	nipponbare_chr12	11457500	11462500
Nipponbare	nipponbare_boundary_1748	nipponbare_chr12	12897500	12902500
Nipponbare	nipponbare_boundary_1749	nipponbare_chr12	12967500	12972500
Nipponbare	nipponbare_boundary_1750	nipponbare_chr12	13117500	13122500
Nipponbare	nipponbare_boundary_1751	nipponbare_chr12	13192500	13197500
Nipponbare	nipponbare_boundary_1752	nipponbare_chr12	13697500	13702500
Nipponbare	nipponbare_boundary_1753	nipponbare_chr12	13762500	13767500
Nipponbare	nipponbare_boundary_1754	nipponbare_chr12	14417500	14422500
Nipponbare	nipponbare_boundary_1755	nipponbare_chr12	14502500	14507500
Nipponbare	nipponbare_boundary_1756	nipponbare_chr12	14677500	14682500

Nipponbare	nipponbare_boundary_1757	nipponbare_chr12	14760000	14765000
Nipponbare	nipponbare_boundary_1758	nipponbare_chr12	16452500	16500000
Nipponbare	nipponbare_boundary_1759	nipponbare_chr12	16557500	16562500
Nipponbare	nipponbare_boundary_1760	nipponbare_chr12	17352500	17357500
Nipponbare	nipponbare_boundary_1761	nipponbare_chr12	17412500	17417500
Nipponbare	nipponbare_boundary_1762	nipponbare_chr12	17497500	17502500
Nipponbare	nipponbare_boundary_1763	nipponbare_chr12	17567500	17572500
Nipponbare	nipponbare_boundary_1764	nipponbare_chr12	17622500	17627500
Nipponbare	nipponbare_boundary_1765	nipponbare_chr12	18477500	18482500
Nipponbare	nipponbare_boundary_1766	nipponbare_chr12	18582500	18587500
Nipponbare	nipponbare_boundary_1767	nipponbare_chr12	19202500	19207500
Nipponbare	nipponbare_boundary_1768	nipponbare_chr12	19422500	19427500
Nipponbare	nipponbare_boundary_1769	nipponbare_chr12	19492500	19497500
Nipponbare	nipponbare_boundary_1770	nipponbare_chr12	19537500	19542500
Nipponbare	nipponbare_boundary_1771	nipponbare_chr12	19632500	19637500
Nipponbare	nipponbare_boundary_1772	nipponbare_chr12	19892500	19897500
Nipponbare	nipponbare_boundary_1773	nipponbare_chr12	19952500	19957500
Nipponbare	nipponbare_boundary_1774	nipponbare_chr12	20082500	20087500
Nipponbare	nipponbare_boundary_1775	nipponbare_chr12	20207500	20212500
Nipponbare	nipponbare_boundary_1776	nipponbare_chr12	20782500	20787500
Nipponbare	nipponbare_boundary_1777	nipponbare_chr12	20842500	20847500
Nipponbare	nipponbare_boundary_1778	nipponbare_chr12	21457500	21497500
Nipponbare	nipponbare_boundary_1779	nipponbare_chr12	21547500	21552500
Nipponbare	nipponbare_boundary_1780	nipponbare_chr12	21617500	21622500
Nipponbare	nipponbare_boundary_1781	nipponbare_chr12	21687500	21692500
Nipponbare	nipponbare_boundary_1782	nipponbare_chr12	22092500	22097500
Nipponbare	nipponbare_boundary_1783	nipponbare_chr12	22182500	22187500
Nipponbare	nipponbare_boundary_1784	nipponbare_chr12	22252500	22297500
Nipponbare	nipponbare_boundary_1785	nipponbare_chr12	22527500	22572500
Nipponbare	nipponbare_boundary_1786	nipponbare_chr12	22767500	22772500
Nipponbare	nipponbare_boundary_1787	nipponbare_chr12	22847500	22852500
Nipponbare	nipponbare_boundary_1788	nipponbare_chr12	22937500	22942500

Nipponbare	nipponbare_boundary_1789	nipponbare_chr12	23102500	23107500
Nipponbare	nipponbare_boundary_1790	nipponbare_chr12	23162500	23167500
Nipponbare	nipponbare_boundary_1791	nipponbare_chr12	23262500	23267500
Nipponbare	nipponbare_boundary_1792	nipponbare_chr12	23347500	23352500
Nipponbare	nipponbare_boundary_1793	nipponbare_chr12	23397500	23442500
Nipponbare	nipponbare_boundary_1794	nipponbare_chr12	23657500	23662500
Nipponbare	nipponbare_boundary_1795	nipponbare_chr12	23702500	23737500
Nipponbare	nipponbare_boundary_1796	nipponbare_chr12	23847500	23852500
Nipponbare	nipponbare_boundary_1797	nipponbare_chr12	23912500	23917500
Nipponbare	nipponbare_boundary_1798	nipponbare_chr12	24027500	24032500
Nipponbare	nipponbare_boundary_1799	nipponbare_chr12	24877500	24882500
Nipponbare	nipponbare_boundary_1800	nipponbare_chr12	24922500	24927500
Nipponbare	nipponbare_boundary_1801	nipponbare_chr12	25532500	25537500
Nipponbare	nipponbare_boundary_1802	nipponbare_chr12	25627500	25672500
Nipponbare	nipponbare_boundary_1803	nipponbare_chr12	25747500	25752500
Nipponbare	nipponbare_boundary_1804	nipponbare_chr12	25832500	25837500
Nipponbare	nipponbare_boundary_1805	nipponbare_chr12	25912500	25917500
Nipponbare	nipponbare_boundary_1806	nipponbare_chr12	25957500	25962500
Nipponbare	nipponbare_boundary_1807	nipponbare_chr12	26022500	26027500
Nipponbare	nipponbare_boundary_1808	nipponbare_chr12	26092500	26097500
Nipponbare	nipponbare_boundary_1809	nipponbare_chr12	26152500	26157500
Nipponbare	nipponbare_boundary_1810	nipponbare_chr12	26222500	26227500
Nipponbare	nipponbare_boundary_1811	nipponbare_chr12	26297500	26302500
IR64	IR64_boundary_0	IR64_chr01	212500	217500
IR64	IR64_boundary_1	IR64_chr01	292500	377500
IR64	IR64_boundary_2	IR64_chr01	422500	427500
IR64	IR64_boundary_3	IR64_chr01	517500	522500
IR64	IR64_boundary_4	IR64_chr01	602500	607500
IR64	IR64_boundary_5	IR64_chr01	697500	702500
IR64	IR64_boundary_6	IR64_chr01	857500	862500
IR64	IR64_boundary_7	IR64_chr01	942500	947500
IR64	IR64_boundary_8	IR64_chr01	1327500	1332500

IR64	IR64_boundary_9	IR64_chr01	1372500	1420000
IR64	IR64_boundary_10	IR64_chr01	1682500	1687500
IR64	IR64_boundary_11	IR64_chr01	1767500	1807500
IR64	IR64_boundary_12	IR64_chr01	1992500	1997500
IR64	IR64_boundary_13	IR64_chr01	2052500	2057500
IR64	IR64_boundary_14	IR64_chr01	2122500	2205000
IR64	IR64_boundary_15	IR64_chr01	2247500	2287500
IR64	IR64_boundary_16	IR64_chr01	2397500	2402500
IR64	IR64_boundary_17	IR64_chr01	2492500	2497500
IR64	IR64_boundary_18	IR64_chr01	2542500	2547500
IR64	IR64_boundary_19	IR64_chr01	3202500	3207500
IR64	IR64_boundary_20	IR64_chr01	3302500	3307500
IR64	IR64_boundary_21	IR64_chr01	3972500	4012500
IR64	IR64_boundary_22	IR64_chr01	4272500	4277500
IR64	IR64_boundary_23	IR64_chr01	4332500	4337500
IR64	IR64_boundary_24	IR64_chr01	4492500	4497500
IR64	IR64_boundary_25	IR64_chr01	4547500	4592500
IR64	IR64_boundary_26	IR64_chr01	4657500	4662500
IR64	IR64_boundary_27	IR64_chr01	4807500	4852500
IR64	IR64_boundary_28	IR64_chr01	5242500	5247500
IR64	IR64_boundary_29	IR64_chr01	5302500	5307500
IR64	IR64_boundary_30	IR64_chr01	5372500	5377500
IR64	IR64_boundary_31	IR64_chr01	6572500	6577500
IR64	IR64_boundary_32	IR64_chr01	6657500	6697500
IR64	IR64_boundary_33	IR64_chr01	6812500	6817500
IR64	IR64_boundary_34	IR64_chr01	6907500	6912500
IR64	IR64_boundary_35	IR64_chr01	7432500	7437500
IR64	IR64_boundary_36	IR64_chr01	7482500	7487500
IR64	IR64_boundary_37	IR64_chr01	7547500	7552500
IR64	IR64_boundary_38	IR64_chr01	7627500	7632500
IR64	IR64_boundary_39	IR64_chr01	7687500	7692500
IR64	IR64_boundary_40	IR64_chr01	8372500	8377500

IR64	IR64_boundary_41	IR64_chr01	8417500	8422500
IR64	IR64_boundary_42	IR64_chr01	8497500	8537500
IR64	IR64_boundary_43	IR64_chr01	8702500	8747500
IR64	IR64_boundary_44	IR64_chr01	9037500	9042500
IR64	IR64_boundary_45	IR64_chr01	9082500	9087500
IR64	IR64_boundary_46	IR64_chr01	9187500	9192500
IR64	IR64_boundary_47	IR64_chr01	9237500	9242500
IR64	IR64_boundary_48	IR64_chr01	9577500	9582500
IR64	IR64_boundary_49	IR64_chr01	9632500	9637500
IR64	IR64_boundary_50	IR64_chr01	10422500	10427500
IR64	IR64_boundary_51	IR64_chr01	10492500	10497500
IR64	IR64_boundary_52	IR64_chr01	10557500	10562500
IR64	IR64_boundary_53	IR64_chr01	10647500	10652500
IR64	IR64_boundary_54	IR64_chr01	10802500	10807500
IR64	IR64_boundary_55	IR64_chr01	10962500	10967500
IR64	IR64_boundary_56	IR64_chr01	11027500	11032500
IR64	IR64_boundary_57	IR64_chr01	11302500	11347500
IR64	IR64_boundary_58	IR64_chr01	11597500	11602500
IR64	IR64_boundary_59	IR64_chr01	11672500	11677500
IR64	IR64_boundary_60	IR64_chr01	11727500	11732500
IR64	IR64_boundary_61	IR64_chr01	11797500	11842500
IR64	IR64_boundary_62	IR64_chr01	13142500	13147500
IR64	IR64_boundary_63	IR64_chr01	13207500	13212500
IR64	IR64_boundary_64	IR64_chr01	13312500	13317500
IR64	IR64_boundary_65	IR64_chr01	13437500	13482500
IR64	IR64_boundary_66	IR64_chr01	13707500	13712500
IR64	IR64_boundary_67	IR64_chr01	14062500	14067500
IR64	IR64_boundary_68	IR64_chr01	14117500	14122500
IR64	IR64_boundary_69	IR64_chr01	14647500	14652500
IR64	IR64_boundary_70	IR64_chr01	14707500	14712500
IR64	IR64_boundary_71	IR64_chr01	14815000	14820000
IR64	IR64_boundary_72	IR64_chr01	14872500	14877500

IR64	IR64_boundary_73	IR64_chr01	15007500	15012500
IR64	IR64_boundary_74	IR64_chr01	15212500	15217500
IR64	IR64_boundary_75	IR64_chr01	15317500	15322500
IR64	IR64_boundary_76	IR64_chr01	15367500	15372500
IR64	IR64_boundary_77	IR64_chr01	15452500	15457500
IR64	IR64_boundary_78	IR64_chr01	15712500	15717500
IR64	IR64_boundary_79	IR64_chr01	15772500	15777500
IR64	IR64_boundary_80	IR64_chr01	15852500	15857500
IR64	IR64_boundary_81	IR64_chr01	16097500	16102500
IR64	IR64_boundary_82	IR64_chr01	16187500	16192500
IR64	IR64_boundary_83	IR64_chr01	16272500	16277500
IR64	IR64_boundary_84	IR64_chr01	18652500	18697500
IR64	IR64_boundary_85	IR64_chr01	18867500	18907500
IR64	IR64_boundary_86	IR64_chr01	18967500	18972500
IR64	IR64_boundary_87	IR64_chr01	19117500	19122500
IR64	IR64_boundary_88	IR64_chr01	19272500	19277500
IR64	IR64_boundary_89	IR64_chr01	19337500	19342500
IR64	IR64_boundary_90	IR64_chr01	20732500	20737500
IR64	IR64_boundary_91	IR64_chr01	20777500	20782500
IR64	IR64_boundary_92	IR64_chr01	20997500	21032500
IR64	IR64_boundary_93	IR64_chr01	21092500	21097500
IR64	IR64_boundary_94	IR64_chr01	21702500	21707500
IR64	IR64_boundary_95	IR64_chr01	21757500	21762500
IR64	IR64_boundary_96	IR64_chr01	21892500	21897500
IR64	IR64_boundary_97	IR64_chr01	21972500	22017500
IR64	IR64_boundary_98	IR64_chr01	22127500	22132500
IR64	IR64_boundary_99	IR64_chr01	23027500	23032500
IR64	IR64_boundary_100	IR64_chr01	23072500	23077500
IR64	IR64_boundary_101	IR64_chr01	23212500	23217500
IR64	IR64_boundary_102	IR64_chr01	23287500	23292500
IR64	IR64_boundary_103	IR64_chr01	23792500	23797500
IR64	IR64_boundary_104	IR64_chr01	23872500	23877500

IR64	IR64_boundary_105	IR64_chr01	23927500	23932500
IR64	IR64_boundary_106	IR64_chr01	23982500	23987500
IR64	IR64_boundary_107	IR64_chr01	24052500	24057500
IR64	IR64_boundary_108	IR64_chr01	24132500	24137500
IR64	IR64_boundary_109	IR64_chr01	24412500	24417500
IR64	IR64_boundary_110	IR64_chr01	24497500	24502500
IR64	IR64_boundary_111	IR64_chr01	24692500	24697500
IR64	IR64_boundary_112	IR64_chr01	24782500	24787500
IR64	IR64_boundary_113	IR64_chr01	24922500	24927500
IR64	IR64_boundary_114	IR64_chr01	24972500	24977500
IR64	IR64_boundary_115	IR64_chr01	25787500	25792500
IR64	IR64_boundary_116	IR64_chr01	25832500	25837500
IR64	IR64_boundary_117	IR64_chr01	26007500	26012500
IR64	IR64_boundary_118	IR64_chr01	26087500	26092500
IR64	IR64_boundary_119	IR64_chr01	26277500	26282500
IR64	IR64_boundary_120	IR64_chr01	26332500	26392500
IR64	IR64_boundary_121	IR64_chr01	26442500	26447500
IR64	IR64_boundary_122	IR64_chr01	26507500	26512500
IR64	IR64_boundary_123	IR64_chr01	26592500	26597500
IR64	IR64_boundary_124	IR64_chr01	26637500	26642500
IR64	IR64_boundary_125	IR64_chr01	26727500	26732500
IR64	IR64_boundary_126	IR64_chr01	26812500	26817500
IR64	IR64_boundary_127	IR64_chr01	26882500	26887500
IR64	IR64_boundary_128	IR64_chr01	27012500	27052500
IR64	IR64_boundary_129	IR64_chr01	27382500	27387500
IR64	IR64_boundary_130	IR64_chr01	27532500	27537500
IR64	IR64_boundary_131	IR64_chr01	27652500	27657500
IR64	IR64_boundary_132	IR64_chr01	27752500	27757500
IR64	IR64_boundary_133	IR64_chr01	27817500	27822500
IR64	IR64_boundary_134	IR64_chr01	27902500	27907500
IR64	IR64_boundary_135	IR64_chr01	28017500	28022500
IR64	IR64_boundary_136	IR64_chr01	28317500	28322500

IR64	IR64_boundary_137	IR64_chr01	28382500	28387500
IR64	IR64_boundary_138	IR64_chr01	28427500	28432500
IR64	IR64_boundary_139	IR64_chr01	28567500	28572500
IR64	IR64_boundary_140	IR64_chr01	28612500	28617500
IR64	IR64_boundary_141	IR64_chr01	28777500	28882500
IR64	IR64_boundary_142	IR64_chr01	29272500	29307500
IR64	IR64_boundary_143	IR64_chr01	29372500	29377500
IR64	IR64_boundary_144	IR64_chr01	29482500	29487500
IR64	IR64_boundary_145	IR64_chr01	29587500	29697500
IR64	IR64_boundary_146	IR64_chr01	29972500	29977500
IR64	IR64_boundary_147	IR64_chr01	30047500	30052500
IR64	IR64_boundary_148	IR64_chr01	30092500	30097500
IR64	IR64_boundary_149	IR64_chr01	30202500	30232500
IR64	IR64_boundary_150	IR64_chr01	30307500	30342500
IR64	IR64_boundary_151	IR64_chr01	30387500	30392500
IR64	IR64_boundary_152	IR64_chr01	30467500	30510000
IR64	IR64_boundary_153	IR64_chr01	30702500	30732500
IR64	IR64_boundary_154	IR64_chr01	30837500	30842500
IR64	IR64_boundary_155	IR64_chr01	31432500	31502500
IR64	IR64_boundary_156	IR64_chr01	31722500	31727500
IR64	IR64_boundary_157	IR64_chr01	31812500	31817500
IR64	IR64_boundary_158	IR64_chr01	31857500	31862500
IR64	IR64_boundary_159	IR64_chr01	32342500	32347500
IR64	IR64_boundary_160	IR64_chr01	32402500	32407500
IR64	IR64_boundary_161	IR64_chr01	32502500	32507500
IR64	IR64_boundary_162	IR64_chr01	32552500	32557500
IR64	IR64_boundary_163	IR64_chr01	32627500	32672500
IR64	IR64_boundary_164	IR64_chr01	32917500	32957500
IR64	IR64_boundary_165	IR64_chr01	33407500	33412500
IR64	IR64_boundary_166	IR64_chr01	33462500	33467500
IR64	IR64_boundary_167	IR64_chr01	33522500	33547500
IR64	IR64_boundary_168	IR64_chr01	33607500	33612500

IR64	IR64_boundary_169	IR64_chr01	33957500	33962500
IR64	IR64_boundary_170	IR64_chr01	34007500	34012500
IR64	IR64_boundary_171	IR64_chr01	34177500	34182500
IR64	IR64_boundary_172	IR64_chr01	34227500	34267500
IR64	IR64_boundary_173	IR64_chr01	34342500	34347500
IR64	IR64_boundary_174	IR64_chr01	34427500	34432500
IR64	IR64_boundary_175	IR64_chr01	34502500	34507500
IR64	IR64_boundary_176	IR64_chr01	34882500	34887500
IR64	IR64_boundary_177	IR64_chr01	34967500	34972500
IR64	IR64_boundary_178	IR64_chr01	35017500	35022500
IR64	IR64_boundary_179	IR64_chr01	35097500	35102500
IR64	IR64_boundary_180	IR64_chr01	35382500	35387500
IR64	IR64_boundary_181	IR64_chr01	35507500	35512500
IR64	IR64_boundary_182	IR64_chr01	35847500	35852500
IR64	IR64_boundary_183	IR64_chr01	35892500	35897500
IR64	IR64_boundary_184	IR64_chr01	36452500	36457500
IR64	IR64_boundary_185	IR64_chr01	36517500	36562500
IR64	IR64_boundary_186	IR64_chr01	36612500	36617500
IR64	IR64_boundary_187	IR64_chr01	36662500	36667500
IR64	IR64_boundary_188	IR64_chr01	36832500	36877500
IR64	IR64_boundary_189	IR64_chr01	37757500	37762500
IR64	IR64_boundary_190	IR64_chr01	37802500	37877500
IR64	IR64_boundary_191	IR64_chr01	37947500	37952500
IR64	IR64_boundary_192	IR64_chr01	37997500	38002500
IR64	IR64_boundary_193	IR64_chr01	38052500	38057500
IR64	IR64_boundary_194	IR64_chr01	38137500	38142500
IR64	IR64_boundary_195	IR64_chr01	38367500	38372500
IR64	IR64_boundary_196	IR64_chr01	38472500	38512500
IR64	IR64_boundary_197	IR64_chr01	38602500	38607500
IR64	IR64_boundary_198	IR64_chr01	38677500	38682500
IR64	IR64_boundary_199	IR64_chr01	38737500	38892500
IR64	IR64_boundary_200	IR64_chr01	38952500	38957500

IR64	IR64_boundary_201	IR64_chr01	39002500	39007500
IR64	IR64_boundary_202	IR64_chr01	39477500	39482500
IR64	IR64_boundary_203	IR64_chr01	39527500	39532500
IR64	IR64_boundary_204	IR64_chr01	39622500	39627500
IR64	IR64_boundary_205	IR64_chr01	39667500	39712500
IR64	IR64_boundary_206	IR64_chr01	40047500	40052500
IR64	IR64_boundary_207	IR64_chr01	40112500	40117500
IR64	IR64_boundary_208	IR64_chr01	40572500	40577500
IR64	IR64_boundary_209	IR64_chr01	40617500	40622500
IR64	IR64_boundary_210	IR64_chr01	41187500	41257500
IR64	IR64_boundary_211	IR64_chr01	41297500	41302500
IR64	IR64_boundary_212	IR64_chr01	41377500	41422500
IR64	IR64_boundary_213	IR64_chr01	41577500	41582500
IR64	IR64_boundary_214	IR64_chr01	41652500	41657500
IR64	IR64_boundary_215	IR64_chr01	41697500	41852500
IR64	IR64_boundary_216	IR64_chr01	42137500	42142500
IR64	IR64_boundary_217	IR64_chr01	42217500	42222500
IR64	IR64_boundary_218	IR64_chr01	42272500	42312500
IR64	IR64_boundary_219	IR64_chr01	42667500	42672500
IR64	IR64_boundary_220	IR64_chr01	42732500	42737500
IR64	IR64_boundary_221	IR64_chr01	42942500	42947500
IR64	IR64_boundary_222	IR64_chr01	43157500	43162500
IR64	IR64_boundary_223	IR64_chr01	43207500	43247500
IR64	IR64_boundary_224	IR64_chr01	43822500	43827500
IR64	IR64_boundary_225	IR64_chr01	43882500	43887500
IR64	IR64_boundary_226	IR64_chr01	43930000	43935000
IR64	IR64_boundary_227	IR64_chr01	43977500	43982500
IR64	IR64_boundary_228	IR64_chr01	44042500	44047500
IR64	IR64_boundary_229	IR64_chr01	44207500	44212500
IR64	IR64_boundary_230	IR64_chr01	44257500	44262500
IR64	IR64_boundary_231	IR64_chr02	137500	142500
IR64	IR64_boundary_232	IR64_chr02	197500	202500

IR64	IR64_boundary_233	IR64_chr02	302500	307500
IR64	IR64_boundary_234	IR64_chr02	407500	412500
IR64	IR64_boundary_235	IR64_chr02	552500	557500
IR64	IR64_boundary_236	IR64_chr02	617500	622500
IR64	IR64_boundary_237	IR64_chr02	680000	685000
IR64	IR64_boundary_238	IR64_chr02	1067500	1112500
IR64	IR64_boundary_239	IR64_chr02	1392500	1432500
IR64	IR64_boundary_240	IR64_chr02	1492500	1497500
IR64	IR64_boundary_241	IR64_chr02	1547500	1592500
IR64	IR64_boundary_242	IR64_chr02	1672500	1677500
IR64	IR64_boundary_243	IR64_chr02	2957500	2962500
IR64	IR64_boundary_244	IR64_chr02	3027500	3032500
IR64	IR64_boundary_245	IR64_chr02	3097500	3102500
IR64	IR64_boundary_246	IR64_chr02	3307500	3312500
IR64	IR64_boundary_247	IR64_chr02	3372500	3377500
IR64	IR64_boundary_248	IR64_chr02	3432500	3477500
IR64	IR64_boundary_249	IR64_chr02	3527500	3532500
IR64	IR64_boundary_250	IR64_chr02	3645000	3650000
IR64	IR64_boundary_251	IR64_chr02	3917500	3922500
IR64	IR64_boundary_252	IR64_chr02	4012500	4017500
IR64	IR64_boundary_253	IR64_chr02	4057500	4062500
IR64	IR64_boundary_254	IR64_chr02	4262500	4267500
IR64	IR64_boundary_255	IR64_chr02	4317500	4322500
IR64	IR64_boundary_256	IR64_chr02	4817500	4822500
IR64	IR64_boundary_257	IR64_chr02	4892500	4897500
IR64	IR64_boundary_258	IR64_chr02	4952500	4957500
IR64	IR64_boundary_259	IR64_chr02	5442500	5447500
IR64	IR64_boundary_260	IR64_chr02	5492500	5497500
IR64	IR64_boundary_261	IR64_chr02	5537500	5542500
IR64	IR64_boundary_262	IR64_chr02	5592500	5597500
IR64	IR64_boundary_263	IR64_chr02	5657500	5662500
IR64	IR64_boundary_264	IR64_chr02	5707500	5712500

IR64	IR64_boundary_265	IR64_chr02	5807500	5812500
IR64	IR64_boundary_266	IR64_chr02	6617500	6622500
IR64	IR64_boundary_267	IR64_chr02	6662500	6667500
IR64	IR64_boundary_268	IR64_chr02	6742500	6747500
IR64	IR64_boundary_269	IR64_chr02	7007500	7012500
IR64	IR64_boundary_270	IR64_chr02	7087500	7092500
IR64	IR64_boundary_271	IR64_chr02	7247500	7252500
IR64	IR64_boundary_272	IR64_chr02	7302500	7342500
IR64	IR64_boundary_273	IR64_chr02	7397500	7402500
IR64	IR64_boundary_274	IR64_chr02	7607500	7612500
IR64	IR64_boundary_275	IR64_chr02	7707500	7712500
IR64	IR64_boundary_276	IR64_chr02	8267500	8357500
IR64	IR64_boundary_277	IR64_chr02	8412500	8417500
IR64	IR64_boundary_278	IR64_chr02	8922500	8965000
IR64	IR64_boundary_279	IR64_chr02	9027500	9032500
IR64	IR64_boundary_280	IR64_chr02	9552500	9557500
IR64	IR64_boundary_281	IR64_chr02	9607500	9647500
IR64	IR64_boundary_282	IR64_chr02	9902500	9907500
IR64	IR64_boundary_283	IR64_chr02	9962500	9967500
IR64	IR64_boundary_284	IR64_chr02	10082500	10087500
IR64	IR64_boundary_285	IR64_chr02	10137500	10142500
IR64	IR64_boundary_286	IR64_chr02	10202500	10207500
IR64	IR64_boundary_287	IR64_chr02	10627500	10632500
IR64	IR64_boundary_288	IR64_chr02	10692500	10697500
IR64	IR64_boundary_289	IR64_chr02	10822500	10827500
IR64	IR64_boundary_290	IR64_chr02	10872500	10877500
IR64	IR64_boundary_291	IR64_chr02	11692500	11697500
IR64	IR64_boundary_292	IR64_chr02	11797500	11802500
IR64	IR64_boundary_293	IR64_chr02	12117500	12122500
IR64	IR64_boundary_294	IR64_chr02	12237500	12242500
IR64	IR64_boundary_295	IR64_chr02	12477500	12482500
IR64	IR64_boundary_296	IR64_chr02	12532500	12537500

IR64	IR64_boundary_297	IR64_chr02	12587500	12592500
IR64	IR64_boundary_298	IR64_chr02	12637500	12642500
IR64	IR64_boundary_299	IR64_chr02	12722500	12727500
IR64	IR64_boundary_300	IR64_chr02	12812500	12817500
IR64	IR64_boundary_301	IR64_chr02	12887500	12892500
IR64	IR64_boundary_302	IR64_chr02	13062500	13067500
IR64	IR64_boundary_303	IR64_chr02	13252500	13257500
IR64	IR64_boundary_304	IR64_chr02	13307500	13312500
IR64	IR64_boundary_305	IR64_chr02	13432500	13437500
IR64	IR64_boundary_306	IR64_chr02	13487500	13492500
IR64	IR64_boundary_307	IR64_chr02	13632500	13637500
IR64	IR64_boundary_308	IR64_chr02	13682500	13687500
IR64	IR64_boundary_309	IR64_chr02	13827500	13832500
IR64	IR64_boundary_310	IR64_chr02	13990000	13995000
IR64	IR64_boundary_311	IR64_chr02	16217500	16222500
IR64	IR64_boundary_312	IR64_chr02	16327500	16332500
IR64	IR64_boundary_313	IR64_chr02	16402500	16407500
IR64	IR64_boundary_314	IR64_chr02	16457500	16502500
IR64	IR64_boundary_315	IR64_chr02	16737500	16742500
IR64	IR64_boundary_316	IR64_chr02	16787500	16792500
IR64	IR64_boundary_317	IR64_chr02	16840000	16845000
IR64	IR64_boundary_318	IR64_chr02	16902500	16907500
IR64	IR64_boundary_319	IR64_chr02	19447500	19452500
IR64	IR64_boundary_320	IR64_chr02	19527500	19532500
IR64	IR64_boundary_321	IR64_chr02	19617500	19622500
IR64	IR64_boundary_322	IR64_chr02	19662500	19667500
IR64	IR64_boundary_323	IR64_chr02	19877500	19882500
IR64	IR64_boundary_324	IR64_chr02	19937500	19942500
IR64	IR64_boundary_325	IR64_chr02	20192500	20197500
IR64	IR64_boundary_326	IR64_chr02	20277500	20312500
IR64	IR64_boundary_327	IR64_chr02	20367500	20372500
IR64	IR64_boundary_328	IR64_chr02	20707500	20712500

IR64	IR64_boundary_329	IR64_chr02	20802500	20807500
IR64	IR64_boundary_330	IR64_chr02	20907500	20912500
IR64	IR64_boundary_331	IR64_chr02	20987500	20992500
IR64	IR64_boundary_332	IR64_chr02	21910000	21915000
IR64	IR64_boundary_333	IR64_chr02	22012500	22017500
IR64	IR64_boundary_334	IR64_chr02	23067500	23102500
IR64	IR64_boundary_335	IR64_chr02	23422500	23427500
IR64	IR64_boundary_336	IR64_chr02	23502500	23507500
IR64	IR64_boundary_337	IR64_chr02	23582500	23587500
IR64	IR64_boundary_338	IR64_chr02	24182500	24262500
IR64	IR64_boundary_339	IR64_chr02	24512500	24517500
IR64	IR64_boundary_340	IR64_chr02	24582500	24587500
IR64	IR64_boundary_341	IR64_chr02	24632500	24637500
IR64	IR64_boundary_342	IR64_chr02	24782500	24787500
IR64	IR64_boundary_343	IR64_chr02	24837500	24842500
IR64	IR64_boundary_344	IR64_chr02	24902500	24907500
IR64	IR64_boundary_345	IR64_chr02	24967500	24972500
IR64	IR64_boundary_346	IR64_chr02	25057500	25102500
IR64	IR64_boundary_347	IR64_chr02	25807500	25812500
IR64	IR64_boundary_348	IR64_chr02	25877500	25882500
IR64	IR64_boundary_349	IR64_chr02	25947500	25952500
IR64	IR64_boundary_350	IR64_chr02	26002500	26007500
IR64	IR64_boundary_351	IR64_chr02	26087500	26092500
IR64	IR64_boundary_352	IR64_chr02	26187500	26192500
IR64	IR64_boundary_353	IR64_chr02	26342500	26347500
IR64	IR64_boundary_354	IR64_chr02	26412500	26417500
IR64	IR64_boundary_355	IR64_chr02	26472500	26477500
IR64	IR64_boundary_356	IR64_chr02	26517500	26522500
IR64	IR64_boundary_357	IR64_chr02	26652500	26692500
IR64	IR64_boundary_358	IR64_chr02	26832500	26837500
IR64	IR64_boundary_359	IR64_chr02	26912500	26917500
IR64	IR64_boundary_360	IR64_chr02	27617500	27622500

IR64	IR64_boundary_361	IR64_chr02	27702500	27707500
IR64	IR64_boundary_362	IR64_chr02	27957500	27962500
IR64	IR64_boundary_363	IR64_chr02	28062500	28067500
IR64	IR64_boundary_364	IR64_chr02	28257500	28292500
IR64	IR64_boundary_365	IR64_chr02	28677500	28682500
IR64	IR64_boundary_366	IR64_chr02	28737500	28742500
IR64	IR64_boundary_367	IR64_chr02	29027500	29067500
IR64	IR64_boundary_368	IR64_chr02	29107500	29112500
IR64	IR64_boundary_369	IR64_chr02	29452500	29457500
IR64	IR64_boundary_370	IR64_chr02	29517500	29522500
IR64	IR64_boundary_371	IR64_chr02	29667500	29672500
IR64	IR64_boundary_372	IR64_chr02	29792500	29797500
IR64	IR64_boundary_373	IR64_chr02	29842500	29887500
IR64	IR64_boundary_374	IR64_chr02	30262500	30302500
IR64	IR64_boundary_375	IR64_chr02	30377500	30412500
IR64	IR64_boundary_376	IR64_chr02	30472500	30477500
IR64	IR64_boundary_377	IR64_chr02	30537500	30542500
IR64	IR64_boundary_378	IR64_chr02	30642500	30647500
IR64	IR64_boundary_379	IR64_chr02	30697500	30732500
IR64	IR64_boundary_380	IR64_chr02	30792500	30797500
IR64	IR64_boundary_381	IR64_chr02	30927500	31012500
IR64	IR64_boundary_382	IR64_chr02	31552500	31637500
IR64	IR64_boundary_383	IR64_chr02	31797500	31802500
IR64	IR64_boundary_384	IR64_chr02	31882500	31922500
IR64	IR64_boundary_385	IR64_chr02	31992500	31997500
IR64	IR64_boundary_386	IR64_chr02	32037500	32042500
IR64	IR64_boundary_387	IR64_chr02	32087500	32092500
IR64	IR64_boundary_388	IR64_chr02	32137500	32142500
IR64	IR64_boundary_389	IR64_chr02	32182500	32217500
IR64	IR64_boundary_390	IR64_chr02	32387500	32422500
IR64	IR64_boundary_391	IR64_chr02	32497500	32502500
IR64	IR64_boundary_392	IR64_chr02	32637500	32677500

IR64	IR64_boundary_393	IR64_chr02	32727500	32732500
IR64	IR64_boundary_394	IR64_chr02	32802500	32807500
IR64	IR64_boundary_395	IR64_chr02	32902500	32907500
IR64	IR64_boundary_396	IR64_chr02	32962500	33007500
IR64	IR64_boundary_397	IR64_chr02	33132500	33137500
IR64	IR64_boundary_398	IR64_chr02	33182500	33187500
IR64	IR64_boundary_399	IR64_chr02	33292500	33297500
IR64	IR64_boundary_400	IR64_chr02	33537500	33542500
IR64	IR64_boundary_401	IR64_chr02	33595000	33600000
IR64	IR64_boundary_402	IR64_chr02	33647500	33652500
IR64	IR64_boundary_403	IR64_chr02	33797500	33837500
IR64	IR64_boundary_404	IR64_chr02	33907500	33912500
IR64	IR64_boundary_405	IR64_chr02	34062500	34067500
IR64	IR64_boundary_406	IR64_chr02	34172500	34177500
IR64	IR64_boundary_407	IR64_chr02	34217500	34222500
IR64	IR64_boundary_408	IR64_chr02	34337500	34342500
IR64	IR64_boundary_409	IR64_chr02	34402500	34407500
IR64	IR64_boundary_410	IR64_chr02	34462500	34467500
IR64	IR64_boundary_411	IR64_chr02	34537500	34542500
IR64	IR64_boundary_412	IR64_chr02	34627500	34672500
IR64	IR64_boundary_413	IR64_chr02	34722500	34762500
IR64	IR64_boundary_414	IR64_chr02	35247500	35287500
IR64	IR64_boundary_415	IR64_chr02	35327500	35332500
IR64	IR64_boundary_416	IR64_chr02	35407500	35437500
IR64	IR64_boundary_417	IR64_chr02	35557500	35562500
IR64	IR64_boundary_418	IR64_chr02	35622500	35627500
IR64	IR64_boundary_419	IR64_chr02	36392500	36397500
IR64	IR64_boundary_420	IR64_chr02	36437500	36442500
IR64	IR64_boundary_421	IR64_chr02	36597500	36602500
IR64	IR64_boundary_422	IR64_chr02	36652500	36657500
IR64	IR64_boundary_423	IR64_chr02	36722500	36727500
IR64	IR64_boundary_424	IR64_chr02	36892500	36897500

IR64	IR64_boundary_425	IR64_chr02	36937500	36942500
IR64	IR64_boundary_426	IR64_chr02	36982500	36987500
IR64	IR64_boundary_427	IR64_chr03	497500	502500
IR64	IR64_boundary_428	IR64_chr03	557500	597500
IR64	IR64_boundary_429	IR64_chr03	717500	722500
IR64	IR64_boundary_430	IR64_chr03	782500	827500
IR64	IR64_boundary_431	IR64_chr03	1587500	1592500
IR64	IR64_boundary_432	IR64_chr03	1687500	1692500
IR64	IR64_boundary_433	IR64_chr03	1747500	1752500
IR64	IR64_boundary_434	IR64_chr03	1912500	1917500
IR64	IR64_boundary_435	IR64_chr03	1992500	1997500
IR64	IR64_boundary_436	IR64_chr03	2037500	2042500
IR64	IR64_boundary_437	IR64_chr03	2102500	2137500
IR64	IR64_boundary_438	IR64_chr03	2982500	3030000
IR64	IR64_boundary_439	IR64_chr03	3132500	3137500
IR64	IR64_boundary_440	IR64_chr03	3582500	3587500
IR64	IR64_boundary_441	IR64_chr03	3662500	3667500
IR64	IR64_boundary_442	IR64_chr03	3712500	3717500
IR64	IR64_boundary_443	IR64_chr03	3807500	3812500
IR64	IR64_boundary_444	IR64_chr03	3902500	3907500
IR64	IR64_boundary_445	IR64_chr03	4057500	4062500
IR64	IR64_boundary_446	IR64_chr03	4137500	4142500
IR64	IR64_boundary_447	IR64_chr03	4407500	4412500
IR64	IR64_boundary_448	IR64_chr03	4457500	4462500
IR64	IR64_boundary_449	IR64_chr03	4502500	4507500
IR64	IR64_boundary_450	IR64_chr03	4562500	4567500
IR64	IR64_boundary_451	IR64_chr03	4622500	4657500
IR64	IR64_boundary_452	IR64_chr03	4817500	4822500
IR64	IR64_boundary_453	IR64_chr03	4877500	4917500
IR64	IR64_boundary_454	IR64_chr03	5102500	5142500
IR64	IR64_boundary_455	IR64_chr03	5342500	5377500
IR64	IR64_boundary_456	IR64_chr03	5697500	5702500

IR64	IR64_boundary_457	IR64_chr03	5762500	5802500
IR64	IR64_boundary_458	IR64_chr03	5842500	5847500
IR64	IR64_boundary_459	IR64_chr03	5897500	5902500
IR64	IR64_boundary_460	IR64_chr03	6022500	6062500
IR64	IR64_boundary_461	IR64_chr03	6342500	6347500
IR64	IR64_boundary_462	IR64_chr03	6432500	6437500
IR64	IR64_boundary_463	IR64_chr03	6562500	6567500
IR64	IR64_boundary_464	IR64_chr03	6637500	6642500
IR64	IR64_boundary_465	IR64_chr03	6687500	6692500
IR64	IR64_boundary_466	IR64_chr03	6742500	6747500
IR64	IR64_boundary_467	IR64_chr03	6827500	6832500
IR64	IR64_boundary_468	IR64_chr03	6892500	6897500
IR64	IR64_boundary_469	IR64_chr03	6947500	6952500
IR64	IR64_boundary_470	IR64_chr03	7367500	7372500
IR64	IR64_boundary_471	IR64_chr03	7412500	7417500
IR64	IR64_boundary_472	IR64_chr03	7490000	7495000
IR64	IR64_boundary_473	IR64_chr03	7567500	7572500
IR64	IR64_boundary_474	IR64_chr03	7632500	7637500
IR64	IR64_boundary_475	IR64_chr03	8752500	8757500
IR64	IR64_boundary_476	IR64_chr03	8852500	8857500
IR64	IR64_boundary_477	IR64_chr03	8902500	8907500
IR64	IR64_boundary_478	IR64_chr03	8987500	9027500
IR64	IR64_boundary_479	IR64_chr03	9447500	9522500
IR64	IR64_boundary_480	IR64_chr03	9627500	9632500
IR64	IR64_boundary_481	IR64_chr03	9677500	9682500
IR64	IR64_boundary_482	IR64_chr03	9752500	9757500
IR64	IR64_boundary_483	IR64_chr03	9812500	9817500
IR64	IR64_boundary_484	IR64_chr03	9917500	9922500
IR64	IR64_boundary_485	IR64_chr03	9962500	9967500
IR64	IR64_boundary_486	IR64_chr03	10017500	10022500
IR64	IR64_boundary_487	IR64_chr03	10137500	10142500
IR64	IR64_boundary_488	IR64_chr03	10217500	10247500

IR64	IR64_boundary_489	IR64_chr03	10377500	10412500
IR64	IR64_boundary_490	IR64_chr03	10472500	10477500
IR64	IR64_boundary_491	IR64_chr03	10582500	10587500
IR64	IR64_boundary_492	IR64_chr03	10872500	10907500
IR64	IR64_boundary_493	IR64_chr03	11302500	11382500
IR64	IR64_boundary_494	IR64_chr03	11437500	11442500
IR64	IR64_boundary_495	IR64_chr03	11752500	11757500
IR64	IR64_boundary_496	IR64_chr03	11807500	11812500
IR64	IR64_boundary_497	IR64_chr03	11897500	11957500
IR64	IR64_boundary_498	IR64_chr03	12527500	12532500
IR64	IR64_boundary_499	IR64_chr03	12577500	12582500
IR64	IR64_boundary_500	IR64_chr03	12677500	12682500
IR64	IR64_boundary_501	IR64_chr03	13037500	13042500
IR64	IR64_boundary_502	IR64_chr03	13117500	13122500
IR64	IR64_boundary_503	IR64_chr03	13232500	13237500
IR64	IR64_boundary_504	IR64_chr03	13482500	13487500
IR64	IR64_boundary_505	IR64_chr03	13532500	13537500
IR64	IR64_boundary_506	IR64_chr03	14282500	14287500
IR64	IR64_boundary_507	IR64_chr03	14372500	14377500
IR64	IR64_boundary_508	IR64_chr03	14457500	14462500
IR64	IR64_boundary_509	IR64_chr03	14512500	14517500
IR64	IR64_boundary_510	IR64_chr03	14697500	14702500
IR64	IR64_boundary_511	IR64_chr03	14852500	14857500
IR64	IR64_boundary_512	IR64_chr03	14927500	14932500
IR64	IR64_boundary_513	IR64_chr03	15882500	15887500
IR64	IR64_boundary_514	IR64_chr03	15972500	15977500
IR64	IR64_boundary_515	IR64_chr03	16067500	16072500
IR64	IR64_boundary_516	IR64_chr03	16132500	16137500
IR64	IR64_boundary_517	IR64_chr03	16337500	16342500
IR64	IR64_boundary_518	IR64_chr03	16397500	16402500
IR64	IR64_boundary_519	IR64_chr03	17237500	17277500
IR64	IR64_boundary_520	IR64_chr03	17322500	17327500

IR64	IR64_boundary_521	IR64_chr03	17572500	17577500
IR64	IR64_boundary_522	IR64_chr03	17617500	17622500
IR64	IR64_boundary_523	IR64_chr03	17672500	17677500
IR64	IR64_boundary_524	IR64_chr03	17812500	17817500
IR64	IR64_boundary_525	IR64_chr03	17867500	17872500
IR64	IR64_boundary_526	IR64_chr03	17972500	18017500
IR64	IR64_boundary_527	IR64_chr03	18067500	18072500
IR64	IR64_boundary_528	IR64_chr03	19142500	19147500
IR64	IR64_boundary_529	IR64_chr03	19187500	19192500
IR64	IR64_boundary_530	IR64_chr03	19362500	19367500
IR64	IR64_boundary_531	IR64_chr03	19557500	19562500
IR64	IR64_boundary_532	IR64_chr03	19617500	19622500
IR64	IR64_boundary_533	IR64_chr03	20712500	20717500
IR64	IR64_boundary_534	IR64_chr03	20822500	20827500
IR64	IR64_boundary_535	IR64_chr03	21362500	21402500
IR64	IR64_boundary_536	IR64_chr03	21472500	21477500
IR64	IR64_boundary_537	IR64_chr03	21562500	21567500
IR64	IR64_boundary_538	IR64_chr03	21830000	21835000
IR64	IR64_boundary_539	IR64_chr03	21882500	21887500
IR64	IR64_boundary_540	IR64_chr03	22012500	22077500
IR64	IR64_boundary_541	IR64_chr03	22272500	22277500
IR64	IR64_boundary_542	IR64_chr03	22342500	22347500
IR64	IR64_boundary_543	IR64_chr03	23007500	23012500
IR64	IR64_boundary_544	IR64_chr03	23087500	23092500
IR64	IR64_boundary_545	IR64_chr03	23207500	23252500
IR64	IR64_boundary_546	IR64_chr03	23292500	23297500
IR64	IR64_boundary_547	IR64_chr03	23352500	23357500
IR64	IR64_boundary_548	IR64_chr03	23942500	23947500
IR64	IR64_boundary_549	IR64_chr03	24022500	24107500
IR64	IR64_boundary_550	IR64_chr03	24442500	24487500
IR64	IR64_boundary_551	IR64_chr03	24572500	24577500
IR64	IR64_boundary_552	IR64_chr03	24672500	24677500

IR64	IR64_boundary_553	IR64_chr03	24757500	24762500
IR64	IR64_boundary_554	IR64_chr03	25187500	25192500
IR64	IR64_boundary_555	IR64_chr03	25437500	25442500
IR64	IR64_boundary_556	IR64_chr03	25577500	25582500
IR64	IR64_boundary_557	IR64_chr03	25647500	25652500
IR64	IR64_boundary_558	IR64_chr03	26415000	26420000
IR64	IR64_boundary_559	IR64_chr03	26532500	26537500
IR64	IR64_boundary_560	IR64_chr03	26802500	26807500
IR64	IR64_boundary_561	IR64_chr03	26847500	26852500
IR64	IR64_boundary_562	IR64_chr03	26922500	26927500
IR64	IR64_boundary_563	IR64_chr03	27007500	27012500
IR64	IR64_boundary_564	IR64_chr03	27077500	27082500
IR64	IR64_boundary_565	IR64_chr03	27142500	27182500
IR64	IR64_boundary_566	IR64_chr03	27247500	27252500
IR64	IR64_boundary_567	IR64_chr03	27312500	27317500
IR64	IR64_boundary_568	IR64_chr03	27357500	27362500
IR64	IR64_boundary_569	IR64_chr03	27612500	27617500
IR64	IR64_boundary_570	IR64_chr03	27687500	27692500
IR64	IR64_boundary_571	IR64_chr03	28092500	28097500
IR64	IR64_boundary_572	IR64_chr03	28147500	28187500
IR64	IR64_boundary_573	IR64_chr03	28227500	28232500
IR64	IR64_boundary_574	IR64_chr03	28297500	28302500
IR64	IR64_boundary_575	IR64_chr03	28582500	28587500
IR64	IR64_boundary_576	IR64_chr03	28642500	28647500
IR64	IR64_boundary_577	IR64_chr03	29407500	29447500
IR64	IR64_boundary_578	IR64_chr03	29837500	29842500
IR64	IR64_boundary_579	IR64_chr03	29897500	29902500
IR64	IR64_boundary_580	IR64_chr03	29997500	30002500
IR64	IR64_boundary_581	IR64_chr03	30377500	30407500
IR64	IR64_boundary_582	IR64_chr03	30682500	30687500
IR64	IR64_boundary_583	IR64_chr03	30787500	30792500
IR64	IR64_boundary_584	IR64_chr03	30862500	30867500

IR64	IR64_boundary_585	IR64_chr03	31127500	31132500
IR64	IR64_boundary_586	IR64_chr03	31197500	31277500
IR64	IR64_boundary_587	IR64_chr03	31697500	31702500
IR64	IR64_boundary_588	IR64_chr03	31832500	31947500
IR64	IR64_boundary_589	IR64_chr03	32152500	32157500
IR64	IR64_boundary_590	IR64_chr03	32212500	32217500
IR64	IR64_boundary_591	IR64_chr03	33022500	33027500
IR64	IR64_boundary_592	IR64_chr03	33072500	33077500
IR64	IR64_boundary_593	IR64_chr03	33142500	33147500
IR64	IR64_boundary_594	IR64_chr03	33222500	33227500
IR64	IR64_boundary_595	IR64_chr03	33292500	33297500
IR64	IR64_boundary_596	IR64_chr03	33352500	33357500
IR64	IR64_boundary_597	IR64_chr03	33442500	33447500
IR64	IR64_boundary_598	IR64_chr03	33497500	33502500
IR64	IR64_boundary_599	IR64_chr03	33872500	33907500
IR64	IR64_boundary_600	IR64_chr03	33997500	34002500
IR64	IR64_boundary_601	IR64_chr03	34052500	34097500
IR64	IR64_boundary_602	IR64_chr03	34147500	34152500
IR64	IR64_boundary_603	IR64_chr03	34222500	34227500
IR64	IR64_boundary_604	IR64_chr03	34277500	34282500
IR64	IR64_boundary_605	IR64_chr03	34362500	34367500
IR64	IR64_boundary_606	IR64_chr03	34437500	34442500
IR64	IR64_boundary_607	IR64_chr03	34532500	34567500
IR64	IR64_boundary_608	IR64_chr03	34792500	34797500
IR64	IR64_boundary_609	IR64_chr03	34872500	34877500
IR64	IR64_boundary_610	IR64_chr03	35022500	35027500
IR64	IR64_boundary_611	IR64_chr03	35097500	35102500
IR64	IR64_boundary_612	IR64_chr03	35147500	35152500
IR64	IR64_boundary_613	IR64_chr03	35557500	35562500
IR64	IR64_boundary_614	IR64_chr03	35602500	35607500
IR64	IR64_boundary_615	IR64_chr03	36187500	36192500
IR64	IR64_boundary_616	IR64_chr03	36232500	36272500

IR64	IR64_boundary_617	IR64_chr03	36327500	36332500
IR64	IR64_boundary_618	IR64_chr03	36402500	36407500
IR64	IR64_boundary_619	IR64_chr03	36772500	36777500
IR64	IR64_boundary_620	IR64_chr03	36847500	36852500
IR64	IR64_boundary_621	IR64_chr03	37107500	37137500
IR64	IR64_boundary_622	IR64_chr03	37177500	37182500
IR64	IR64_boundary_623	IR64_chr03	37342500	37377500
IR64	IR64_boundary_624	IR64_chr03	37467500	37472500
IR64	IR64_boundary_625	IR64_chr03	38242500	38247500
IR64	IR64_boundary_626	IR64_chr03	38317500	38322500
IR64	IR64_boundary_627	IR64_chr04	100000	105000
IR64	IR64_boundary_628	IR64_chr04	197500	202500
IR64	IR64_boundary_629	IR64_chr04	242500	282500
IR64	IR64_boundary_630	IR64_chr04	322500	327500
IR64	IR64_boundary_631	IR64_chr04	372500	377500
IR64	IR64_boundary_632	IR64_chr04	1262500	1297500
IR64	IR64_boundary_633	IR64_chr04	1357500	1362500
IR64	IR64_boundary_634	IR64_chr04	1402500	1407500
IR64	IR64_boundary_635	IR64_chr04	1517500	1522500
IR64	IR64_boundary_636	IR64_chr04	3977500	3982500
IR64	IR64_boundary_637	IR64_chr04	4102500	4107500
IR64	IR64_boundary_638	IR64_chr04	4225000	4230000
IR64	IR64_boundary_639	IR64_chr04	4272500	4277500
IR64	IR64_boundary_640	IR64_chr04	4447500	4452500
IR64	IR64_boundary_641	IR64_chr04	4637500	4642500
IR64	IR64_boundary_642	IR64_chr04	4712500	4717500
IR64	IR64_boundary_643	IR64_chr04	4830000	4835000
IR64	IR64_boundary_644	IR64_chr04	4897500	4902500
IR64	IR64_boundary_645	IR64_chr04	4992500	4997500
IR64	IR64_boundary_646	IR64_chr04	5067500	5072500
IR64	IR64_boundary_647	IR64_chr04	5657500	5662500
IR64	IR64_boundary_648	IR64_chr04	5732500	5807500

IR64	IR64_boundary_649	IR64_chr04	6497500	6540000
IR64	IR64_boundary_650	IR64_chr04	6617500	6622500
IR64	IR64_boundary_651	IR64_chr04	7522500	7527500
IR64	IR64_boundary_652	IR64_chr04	7587500	7592500
IR64	IR64_boundary_653	IR64_chr04	11092500	11097500
IR64	IR64_boundary_654	IR64_chr04	11137500	11142500
IR64	IR64_boundary_655	IR64_chr04	11352500	11357500
IR64	IR64_boundary_656	IR64_chr04	11517500	11522500
IR64	IR64_boundary_657	IR64_chr04	11617500	11622500
IR64	IR64_boundary_658	IR64_chr04	11672500	11677500
IR64	IR64_boundary_659	IR64_chr04	11747500	11752500
IR64	IR64_boundary_660	IR64_chr04	12102500	12107500
IR64	IR64_boundary_661	IR64_chr04	12182500	12187500
IR64	IR64_boundary_662	IR64_chr04	12740000	12745000
IR64	IR64_boundary_663	IR64_chr04	12827500	12832500
IR64	IR64_boundary_664	IR64_chr04	13057500	13062500
IR64	IR64_boundary_665	IR64_chr04	13132500	13137500
IR64	IR64_boundary_666	IR64_chr04	13652500	13657500
IR64	IR64_boundary_667	IR64_chr04	13807500	13852500
IR64	IR64_boundary_668	IR64_chr04	13902500	13907500
IR64	IR64_boundary_669	IR64_chr04	13957500	13997500
IR64	IR64_boundary_670	IR64_chr04	14167500	14172500
IR64	IR64_boundary_671	IR64_chr04	14217500	14222500
IR64	IR64_boundary_672	IR64_chr04	14322500	14327500
IR64	IR64_boundary_673	IR64_chr04	14637500	14642500
IR64	IR64_boundary_674	IR64_chr04	14682500	14687500
IR64	IR64_boundary_675	IR64_chr04	15477500	15482500
IR64	IR64_boundary_676	IR64_chr04	15522500	15527500
IR64	IR64_boundary_677	IR64_chr04	15572500	15577500
IR64	IR64_boundary_678	IR64_chr04	15622500	15627500
IR64	IR64_boundary_679	IR64_chr04	16017500	16022500
IR64	IR64_boundary_680	IR64_chr04	16062500	16067500

IR64	IR64_boundary_681	IR64_chr04	16282500	16287500
IR64	IR64_boundary_682	IR64_chr04	16442500	16447500
IR64	IR64_boundary_683	IR64_chr04	18382500	18387500
IR64	IR64_boundary_684	IR64_chr04	18452500	18497500
IR64	IR64_boundary_685	IR64_chr04	18547500	18552500
IR64	IR64_boundary_686	IR64_chr04	18672500	18677500
IR64	IR64_boundary_687	IR64_chr04	18722500	18767500
IR64	IR64_boundary_688	IR64_chr04	18832500	18837500
IR64	IR64_boundary_689	IR64_chr04	19057500	19062500
IR64	IR64_boundary_690	IR64_chr04	19182500	19187500
IR64	IR64_boundary_691	IR64_chr04	19272500	19277500
IR64	IR64_boundary_692	IR64_chr04	19347500	19387500
IR64	IR64_boundary_693	IR64_chr04	19932500	19937500
IR64	IR64_boundary_694	IR64_chr04	20042500	20047500
IR64	IR64_boundary_695	IR64_chr04	20247500	20252500
IR64	IR64_boundary_696	IR64_chr04	20307500	20352500
IR64	IR64_boundary_697	IR64_chr04	20457500	20462500
IR64	IR64_boundary_698	IR64_chr04	20512500	20517500
IR64	IR64_boundary_699	IR64_chr04	20767500	20772500
IR64	IR64_boundary_700	IR64_chr04	20827500	20832500
IR64	IR64_boundary_701	IR64_chr04	20992500	20997500
IR64	IR64_boundary_702	IR64_chr04	21067500	21072500
IR64	IR64_boundary_703	IR64_chr04	21127500	21132500
IR64	IR64_boundary_704	IR64_chr04	21987500	22032500
IR64	IR64_boundary_705	IR64_chr04	22597500	22642500
IR64	IR64_boundary_706	IR64_chr04	23002500	23007500
IR64	IR64_boundary_707	IR64_chr04	23102500	23107500
IR64	IR64_boundary_708	IR64_chr04	23347500	23352500
IR64	IR64_boundary_709	IR64_chr04	23397500	23402500
IR64	IR64_boundary_710	IR64_chr04	23522500	23527500
IR64	IR64_boundary_711	IR64_chr04	23577500	23582500
IR64	IR64_boundary_712	IR64_chr04	23687500	23692500

IR64	IR64_boundary_713	IR64_chr04	23737500	23742500
IR64	IR64_boundary_714	IR64_chr04	23792500	23797500
IR64	IR64_boundary_715	IR64_chr04	23852500	23857500
IR64	IR64_boundary_716	IR64_chr04	23912500	23917500
IR64	IR64_boundary_717	IR64_chr04	24522500	24567500
IR64	IR64_boundary_718	IR64_chr04	24617500	24622500
IR64	IR64_boundary_719	IR64_chr04	26827500	26897500
IR64	IR64_boundary_720	IR64_chr04	27057500	27062500
IR64	IR64_boundary_721	IR64_chr04	27167500	27197500
IR64	IR64_boundary_722	IR64_chr04	28352500	28357500
IR64	IR64_boundary_723	IR64_chr04	28412500	28417500
IR64	IR64_boundary_724	IR64_chr04	28477500	28482500
IR64	IR64_boundary_725	IR64_chr04	28545000	28550000
IR64	IR64_boundary_726	IR64_chr04	28742500	28747500
IR64	IR64_boundary_727	IR64_chr04	28792500	28797500
IR64	IR64_boundary_728	IR64_chr04	28837500	28867500
IR64	IR64_boundary_729	IR64_chr04	29327500	29372500
IR64	IR64_boundary_730	IR64_chr04	29512500	29517500
IR64	IR64_boundary_731	IR64_chr04	29597500	29602500
IR64	IR64_boundary_732	IR64_chr04	30182500	30187500
IR64	IR64_boundary_733	IR64_chr04	30252500	30257500
IR64	IR64_boundary_734	IR64_chr04	30462500	30467500
IR64	IR64_boundary_735	IR64_chr04	30507500	30512500
IR64	IR64_boundary_736	IR64_chr04	30612500	30617500
IR64	IR64_boundary_737	IR64_chr04	30722500	30727500
IR64	IR64_boundary_738	IR64_chr04	30892500	30897500
IR64	IR64_boundary_739	IR64_chr04	30967500	30972500
IR64	IR64_boundary_740	IR64_chr04	31052500	31092500
IR64	IR64_boundary_741	IR64_chr04	31302500	31307500
IR64	IR64_boundary_742	IR64_chr04	31377500	31382500
IR64	IR64_boundary_743	IR64_chr04	31662500	31697500
IR64	IR64_boundary_744	IR64_chr04	31807500	31812500

IR64	IR64_boundary_745	IR64_chr04	31882500	31887500
IR64	IR64_boundary_746	IR64_chr04	31957500	32042500
IR64	IR64_boundary_747	IR64_chr04	32637500	32642500
IR64	IR64_boundary_748	IR64_chr04	32742500	32747500
IR64	IR64_boundary_749	IR64_chr04	32867500	32872500
IR64	IR64_boundary_750	IR64_chr04	32932500	32937500
IR64	IR64_boundary_751	IR64_chr04	33257500	33262500
IR64	IR64_boundary_752	IR64_chr04	33342500	33347500
IR64	IR64_boundary_753	IR64_chr04	33412500	33417500
IR64	IR64_boundary_754	IR64_chr04	33492500	33497500
IR64	IR64_boundary_755	IR64_chr04	33712500	33717500
IR64	IR64_boundary_756	IR64_chr04	33777500	33782500
IR64	IR64_boundary_757	IR64_chr04	34122500	34127500
IR64	IR64_boundary_758	IR64_chr04	34202500	34207500
IR64	IR64_boundary_759	IR64_chr04	34307500	34382500
IR64	IR64_boundary_760	IR64_chr04	34432500	34437500
IR64	IR64_boundary_761	IR64_chr04	34502500	34507500
IR64	IR64_boundary_762	IR64_chr04	34592500	34597500
IR64	IR64_boundary_763	IR64_chr04	35107500	35112500
IR64	IR64_boundary_764	IR64_chr04	35177500	35182500
IR64	IR64_boundary_765	IR64_chr05	137500	142500
IR64	IR64_boundary_766	IR64_chr05	197500	202500
IR64	IR64_boundary_767	IR64_chr05	572500	612500
IR64	IR64_boundary_768	IR64_chr05	947500	952500
IR64	IR64_boundary_769	IR64_chr05	997500	1002500
IR64	IR64_boundary_770	IR64_chr05	1052500	1092500
IR64	IR64_boundary_771	IR64_chr05	1132500	1177500
IR64	IR64_boundary_772	IR64_chr05	1217500	1222500
IR64	IR64_boundary_773	IR64_chr05	2387500	2422500
IR64	IR64_boundary_774	IR64_chr05	2517500	2562500
IR64	IR64_boundary_775	IR64_chr05	2652500	2657500
IR64	IR64_boundary_776	IR64_chr05	2722500	2727500

IR64	IR64_boundary_777	IR64_chr05	2942500	2947500
IR64	IR64_boundary_778	IR64_chr05	3012500	3017500
IR64	IR64_boundary_779	IR64_chr05	3662500	3707500
IR64	IR64_boundary_780	IR64_chr05	6317500	6322500
IR64	IR64_boundary_781	IR64_chr05	6467500	6472500
IR64	IR64_boundary_782	IR64_chr05	6882500	6887500
IR64	IR64_boundary_783	IR64_chr05	6932500	6937500
IR64	IR64_boundary_784	IR64_chr05	7007500	7012500
IR64	IR64_boundary_785	IR64_chr05	7067500	7072500
IR64	IR64_boundary_786	IR64_chr05	7877500	7882500
IR64	IR64_boundary_787	IR64_chr05	8047500	8052500
IR64	IR64_boundary_788	IR64_chr05	8622500	8627500
IR64	IR64_boundary_789	IR64_chr05	8762500	8767500
IR64	IR64_boundary_790	IR64_chr05	9367500	9372500
IR64	IR64_boundary_791	IR64_chr05	9442500	9447500
IR64	IR64_boundary_792	IR64_chr05	9545000	9550000
IR64	IR64_boundary_793	IR64_chr05	9682500	9687500
IR64	IR64_boundary_794	IR64_chr05	11162500	11167500
IR64	IR64_boundary_795	IR64_chr05	11277500	11282500
IR64	IR64_boundary_796	IR64_chr05	13642500	13647500
IR64	IR64_boundary_797	IR64_chr05	13767500	13772500
IR64	IR64_boundary_798	IR64_chr05	13887500	13892500
IR64	IR64_boundary_799	IR64_chr05	13962500	13967500
IR64	IR64_boundary_800	IR64_chr05	14482500	14487500
IR64	IR64_boundary_801	IR64_chr05	14532500	14537500
IR64	IR64_boundary_802	IR64_chr05	14662500	14697500
IR64	IR64_boundary_803	IR64_chr05	14737500	14742500
IR64	IR64_boundary_804	IR64_chr05	14817500	14822500
IR64	IR64_boundary_805	IR64_chr05	14897500	14902500
IR64	IR64_boundary_806	IR64_chr05	15037500	15042500
IR64	IR64_boundary_807	IR64_chr05	16537500	16542500
IR64	IR64_boundary_808	IR64_chr05	16602500	16607500

IR64	IR64_boundary_809	IR64_chr05	16657500	16662500
IR64	IR64_boundary_810	IR64_chr05	16742500	16782500
IR64	IR64_boundary_811	IR64_chr05	16842500	16847500
IR64	IR64_boundary_812	IR64_chr05	16922500	16927500
IR64	IR64_boundary_813	IR64_chr05	16992500	16997500
IR64	IR64_boundary_814	IR64_chr05	17052500	17057500
IR64	IR64_boundary_815	IR64_chr05	17217500	17222500
IR64	IR64_boundary_816	IR64_chr05	17262500	17267500
IR64	IR64_boundary_817	IR64_chr05	17382500	17387500
IR64	IR64_boundary_818	IR64_chr05	17442500	17487500
IR64	IR64_boundary_819	IR64_chr05	17672500	17677500
IR64	IR64_boundary_820	IR64_chr05	17747500	17752500
IR64	IR64_boundary_821	IR64_chr05	17812500	17817500
IR64	IR64_boundary_822	IR64_chr05	18147500	18152500
IR64	IR64_boundary_823	IR64_chr05	18307500	18312500
IR64	IR64_boundary_824	IR64_chr05	18382500	18387500
IR64	IR64_boundary_825	IR64_chr05	18452500	18457500
IR64	IR64_boundary_826	IR64_chr05	18992500	18997500
IR64	IR64_boundary_827	IR64_chr05	19047500	19052500
IR64	IR64_boundary_828	IR64_chr05	19232500	19237500
IR64	IR64_boundary_829	IR64_chr05	19332500	19337500
IR64	IR64_boundary_830	IR64_chr05	19397500	19472500
IR64	IR64_boundary_831	IR64_chr05	20117500	20122500
IR64	IR64_boundary_832	IR64_chr05	20162500	20167500
IR64	IR64_boundary_833	IR64_chr05	20207500	20212500
IR64	IR64_boundary_834	IR64_chr05	20842500	20847500
IR64	IR64_boundary_835	IR64_chr05	20932500	20937500
IR64	IR64_boundary_836	IR64_chr05	21672500	21677500
IR64	IR64_boundary_837	IR64_chr05	21717500	21722500
IR64	IR64_boundary_838	IR64_chr05	21792500	21797500
IR64	IR64_boundary_839	IR64_chr05	21857500	21862500
IR64	IR64_boundary_840	IR64_chr05	21947500	21952500

IR64	IR64_boundary_841	IR64_chr05	22637500	22642500
IR64	IR64_boundary_842	IR64_chr05	22702500	22707500
IR64	IR64_boundary_843	IR64_chr05	22747500	22752500
IR64	IR64_boundary_844	IR64_chr05	22792500	22797500
IR64	IR64_boundary_845	IR64_chr05	23427500	23432500
IR64	IR64_boundary_846	IR64_chr05	23512500	23517500
IR64	IR64_boundary_847	IR64_chr05	23582500	23622500
IR64	IR64_boundary_848	IR64_chr05	23827500	23832500
IR64	IR64_boundary_849	IR64_chr05	23877500	23882500
IR64	IR64_boundary_850	IR64_chr05	24182500	24187500
IR64	IR64_boundary_851	IR64_chr05	24242500	24247500
IR64	IR64_boundary_852	IR64_chr05	24382500	24387500
IR64	IR64_boundary_853	IR64_chr05	24457500	24462500
IR64	IR64_boundary_854	IR64_chr05	24502500	24507500
IR64	IR64_boundary_855	IR64_chr05	24547500	24552500
IR64	IR64_boundary_856	IR64_chr05	24812500	24817500
IR64	IR64_boundary_857	IR64_chr05	24872500	24877500
IR64	IR64_boundary_858	IR64_chr05	24927500	24932500
IR64	IR64_boundary_859	IR64_chr05	25217500	25262500
IR64	IR64_boundary_860	IR64_chr05	25787500	25792500
IR64	IR64_boundary_861	IR64_chr05	25847500	25852500
IR64	IR64_boundary_862	IR64_chr05	25892500	25897500
IR64	IR64_boundary_863	IR64_chr05	26327500	26332500
IR64	IR64_boundary_864	IR64_chr05	26392500	26397500
IR64	IR64_boundary_865	IR64_chr05	26477500	26482500
IR64	IR64_boundary_866	IR64_chr05	26567500	26572500
IR64	IR64_boundary_867	IR64_chr05	26612500	26617500
IR64	IR64_boundary_868	IR64_chr05	26662500	26667500
IR64	IR64_boundary_869	IR64_chr05	26822500	26827500
IR64	IR64_boundary_870	IR64_chr05	26907500	26912500
IR64	IR64_boundary_871	IR64_chr05	26987500	26992500
IR64	IR64_boundary_872	IR64_chr05	27062500	27067500

IR64	IR64_boundary_873	IR64_chr05	27132500	27137500
IR64	IR64_boundary_874	IR64_chr05	27487500	27492500
IR64	IR64_boundary_875	IR64_chr05	27537500	27542500
IR64	IR64_boundary_876	IR64_chr05	27712500	27717500
IR64	IR64_boundary_877	IR64_chr05	27797500	27802500
IR64	IR64_boundary_878	IR64_chr05	27892500	27932500
IR64	IR64_boundary_879	IR64_chr05	27977500	27982500
IR64	IR64_boundary_880	IR64_chr05	28062500	28067500
IR64	IR64_boundary_881	IR64_chr05	28132500	28137500
IR64	IR64_boundary_882	IR64_chr05	28217500	28222500
IR64	IR64_boundary_883	IR64_chr05	28317500	28322500
IR64	IR64_boundary_884	IR64_chr05	28442500	28447500
IR64	IR64_boundary_885	IR64_chr05	28497500	28502500
IR64	IR64_boundary_886	IR64_chr05	28612500	28617500
IR64	IR64_boundary_887	IR64_chr05	28667500	28672500
IR64	IR64_boundary_888	IR64_chr05	29042500	29047500
IR64	IR64_boundary_889	IR64_chr05	29112500	29117500
IR64	IR64_boundary_890	IR64_chr05	29267500	29312500
IR64	IR64_boundary_891	IR64_chr05	29542500	29582500
IR64	IR64_boundary_892	IR64_chr05	29622500	29627500
IR64	IR64_boundary_893	IR64_chr05	29677500	29682500
IR64	IR64_boundary_894	IR64_chr05	29732500	29737500
IR64	IR64_boundary_895	IR64_chr05	29782500	29787500
IR64	IR64_boundary_896	IR64_chr05	29837500	29842500
IR64	IR64_boundary_897	IR64_chr05	29927500	29932500
IR64	IR64_boundary_898	IR64_chr05	30047500	30052500
IR64	IR64_boundary_899	IR64_chr05	30092500	30097500
IR64	IR64_boundary_900	IR64_chr05	30147500	30152500
IR64	IR64_boundary_901	IR64_chr05	30507500	30512500
IR64	IR64_boundary_902	IR64_chr05	30622500	30627500
IR64	IR64_boundary_903	IR64_chr06	1102500	1107500
IR64	IR64_boundary_904	IR64_chr06	1167500	1172500

IR64	IR64_boundary_905	IR64_chr06	2882500	2887500
IR64	IR64_boundary_906	IR64_chr06	2952500	2957500
IR64	IR64_boundary_907	IR64_chr06	3042500	3082500
IR64	IR64_boundary_908	IR64_chr06	3207500	3212500
IR64	IR64_boundary_909	IR64_chr06	3267500	3312500
IR64	IR64_boundary_910	IR64_chr06	3597500	3602500
IR64	IR64_boundary_911	IR64_chr06	3647500	3652500
IR64	IR64_boundary_912	IR64_chr06	3692500	3697500
IR64	IR64_boundary_913	IR64_chr06	3812500	3817500
IR64	IR64_boundary_914	IR64_chr06	3865000	3870000
IR64	IR64_boundary_915	IR64_chr06	3982500	3987500
IR64	IR64_boundary_916	IR64_chr06	4032500	4037500
IR64	IR64_boundary_917	IR64_chr06	4087500	4092500
IR64	IR64_boundary_918	IR64_chr06	4132500	4137500
IR64	IR64_boundary_919	IR64_chr06	4312500	4317500
IR64	IR64_boundary_920	IR64_chr06	4387500	4392500
IR64	IR64_boundary_921	IR64_chr06	4457500	4462500
IR64	IR64_boundary_922	IR64_chr06	4522500	4527500
IR64	IR64_boundary_923	IR64_chr06	4622500	4627500
IR64	IR64_boundary_924	IR64_chr06	5197500	5202500
IR64	IR64_boundary_925	IR64_chr06	5262500	5267500
IR64	IR64_boundary_926	IR64_chr06	5487500	5492500
IR64	IR64_boundary_927	IR64_chr06	5722500	5727500
IR64	IR64_boundary_928	IR64_chr06	5822500	5827500
IR64	IR64_boundary_929	IR64_chr06	7192500	7197500
IR64	IR64_boundary_930	IR64_chr06	7252500	7257500
IR64	IR64_boundary_931	IR64_chr06	7322500	7327500
IR64	IR64_boundary_932	IR64_chr06	7367500	7407500
IR64	IR64_boundary_933	IR64_chr06	7462500	7467500
IR64	IR64_boundary_934	IR64_chr06	7532500	7537500
IR64	IR64_boundary_935	IR64_chr06	7587500	7592500
IR64	IR64_boundary_936	IR64_chr06	8047500	8052500

IR64	IR64_boundary_937	IR64_chr06	8117500	8122500
IR64	IR64_boundary_938	IR64_chr06	8177500	8212500
IR64	IR64_boundary_939	IR64_chr06	8307500	8312500
IR64	IR64_boundary_940	IR64_chr06	8357500	8362500
IR64	IR64_boundary_941	IR64_chr06	8427500	8432500
IR64	IR64_boundary_942	IR64_chr06	8512500	8517500
IR64	IR64_boundary_943	IR64_chr06	8582500	8587500
IR64	IR64_boundary_944	IR64_chr06	9672500	9677500
IR64	IR64_boundary_945	IR64_chr06	9732500	9737500
IR64	IR64_boundary_946	IR64_chr06	9947500	9952500
IR64	IR64_boundary_947	IR64_chr06	10032500	10037500
IR64	IR64_boundary_948	IR64_chr06	10197500	10202500
IR64	IR64_boundary_949	IR64_chr06	10257500	10262500
IR64	IR64_boundary_950	IR64_chr06	10317500	10322500
IR64	IR64_boundary_951	IR64_chr06	10392500	10397500
IR64	IR64_boundary_952	IR64_chr06	10482500	10487500
IR64	IR64_boundary_953	IR64_chr06	10972500	10977500
IR64	IR64_boundary_954	IR64_chr06	11142500	11147500
IR64	IR64_boundary_955	IR64_chr06	11237500	11242500
IR64	IR64_boundary_956	IR64_chr06	11357500	11362500
IR64	IR64_boundary_957	IR64_chr06	12167500	12172500
IR64	IR64_boundary_958	IR64_chr06	12272500	12317500
IR64	IR64_boundary_959	IR64_chr06	12482500	12487500
IR64	IR64_boundary_960	IR64_chr06	12607500	12642500
IR64	IR64_boundary_961	IR64_chr06	12802500	12807500
IR64	IR64_boundary_962	IR64_chr06	12892500	12897500
IR64	IR64_boundary_963	IR64_chr06	13037500	13042500
IR64	IR64_boundary_964	IR64_chr06	13117500	13122500
IR64	IR64_boundary_965	IR64_chr06	13172500	13177500
IR64	IR64_boundary_966	IR64_chr06	13377500	13382500
IR64	IR64_boundary_967	IR64_chr06	13457500	13462500
IR64	IR64_boundary_968	IR64_chr06	13512500	13517500

IR64	IR64_boundary_969	IR64_chr06	14462500	14467500
IR64	IR64_boundary_970	IR64_chr06	14512500	14517500
IR64	IR64_boundary_971	IR64_chr06	15322500	15327500
IR64	IR64_boundary_972	IR64_chr06	15452500	15457500
IR64	IR64_boundary_973	IR64_chr06	15822500	15827500
IR64	IR64_boundary_974	IR64_chr06	15917500	15922500
IR64	IR64_boundary_975	IR64_chr06	15965000	15970000
IR64	IR64_boundary_976	IR64_chr06	18347500	18392500
IR64	IR64_boundary_977	IR64_chr06	19122500	19127500
IR64	IR64_boundary_978	IR64_chr06	19192500	19197500
IR64	IR64_boundary_979	IR64_chr06	19292500	19297500
IR64	IR64_boundary_980	IR64_chr06	19397500	19402500
IR64	IR64_boundary_981	IR64_chr06	20377500	20382500
IR64	IR64_boundary_982	IR64_chr06	20422500	20427500
IR64	IR64_boundary_983	IR64_chr06	20822500	20827500
IR64	IR64_boundary_984	IR64_chr06	20892500	20897500
IR64	IR64_boundary_985	IR64_chr06	20972500	21052500
IR64	IR64_boundary_986	IR64_chr06	21167500	21172500
IR64	IR64_boundary_987	IR64_chr06	21247500	21252500
IR64	IR64_boundary_988	IR64_chr06	21347500	21377500
IR64	IR64_boundary_989	IR64_chr06	21842500	21847500
IR64	IR64_boundary_990	IR64_chr06	21917500	21922500
IR64	IR64_boundary_991	IR64_chr06	21962500	21967500
IR64	IR64_boundary_992	IR64_chr06	22012500	22057500
IR64	IR64_boundary_993	IR64_chr06	22152500	22157500
IR64	IR64_boundary_994	IR64_chr06	22212500	22217500
IR64	IR64_boundary_995	IR64_chr06	22262500	22267500
IR64	IR64_boundary_996	IR64_chr06	22322500	22327500
IR64	IR64_boundary_997	IR64_chr06	22382500	22387500
IR64	IR64_boundary_998	IR64_chr06	22437500	22442500
IR64	IR64_boundary_999	IR64_chr06	22622500	22627500
IR64	IR64_boundary_1000	IR64_chr06	22772500	22777500

IR64	IR64_boundary_1001	IR64_chr06	23137500	23142500
IR64	IR64_boundary_1002	IR64_chr06	23182500	23187500
IR64	IR64_boundary_1003	IR64_chr06	23297500	23302500
IR64	IR64_boundary_1004	IR64_chr06	24377500	24382500
IR64	IR64_boundary_1005	IR64_chr06	24442500	24447500
IR64	IR64_boundary_1006	IR64_chr06	24562500	24657500
IR64	IR64_boundary_1007	IR64_chr06	25267500	25297500
IR64	IR64_boundary_1008	IR64_chr06	25387500	25392500
IR64	IR64_boundary_1009	IR64_chr06	25457500	25462500
IR64	IR64_boundary_1010	IR64_chr06	25507500	25512500
IR64	IR64_boundary_1011	IR64_chr06	25612500	25617500
IR64	IR64_boundary_1012	IR64_chr06	25667500	25672500
IR64	IR64_boundary_1013	IR64_chr06	26052500	26092500
IR64	IR64_boundary_1014	IR64_chr06	26147500	26152500
IR64	IR64_boundary_1015	IR64_chr06	26802500	26807500
IR64	IR64_boundary_1016	IR64_chr06	26902500	26907500
IR64	IR64_boundary_1017	IR64_chr06	27007500	27012500
IR64	IR64_boundary_1018	IR64_chr06	27102500	27107500
IR64	IR64_boundary_1019	IR64_chr06	27302500	27307500
IR64	IR64_boundary_1020	IR64_chr06	27347500	27352500
IR64	IR64_boundary_1021	IR64_chr06	27427500	27432500
IR64	IR64_boundary_1022	IR64_chr06	27492500	27537500
IR64	IR64_boundary_1023	IR64_chr06	27602500	27677500
IR64	IR64_boundary_1024	IR64_chr06	27732500	27812500
IR64	IR64_boundary_1025	IR64_chr06	28552500	28557500
IR64	IR64_boundary_1026	IR64_chr06	28602500	28607500
IR64	IR64_boundary_1027	IR64_chr06	29102500	29107500
IR64	IR64_boundary_1028	IR64_chr06	29167500	29212500
IR64	IR64_boundary_1029	IR64_chr06	29252500	29292500
IR64	IR64_boundary_1030	IR64_chr06	29377500	29407500
IR64	IR64_boundary_1031	IR64_chr06	29467500	29472500
IR64	IR64_boundary_1032	IR64_chr06	29582500	29617500

IR64	IR64_boundary_1033	IR64_chr06	30092500	30097500
IR64	IR64_boundary_1034	IR64_chr06	30172500	30177500
IR64	IR64_boundary_1035	IR64_chr06	30302500	30307500
IR64	IR64_boundary_1036	IR64_chr06	30382500	30387500
IR64	IR64_boundary_1037	IR64_chr06	30437500	30442500
IR64	IR64_boundary_1038	IR64_chr06	30557500	30562500
IR64	IR64_boundary_1039	IR64_chr06	30607500	30612500
IR64	IR64_boundary_1040	IR64_chr06	30692500	30697500
IR64	IR64_boundary_1041	IR64_chr06	30737500	30742500
IR64	IR64_boundary_1042	IR64_chr06	30807500	30812500
IR64	IR64_boundary_1043	IR64_chr06	31702500	31707500
IR64	IR64_boundary_1044	IR64_chr06	31787500	31792500
IR64	IR64_boundary_1045	IR64_chr07	237500	242500
IR64	IR64_boundary_1046	IR64_chr07	307500	312500
IR64	IR64_boundary_1047	IR64_chr07	417500	422500
IR64	IR64_boundary_1048	IR64_chr07	527500	532500
IR64	IR64_boundary_1049	IR64_chr07	577500	582500
IR64	IR64_boundary_1050	IR64_chr07	622500	627500
IR64	IR64_boundary_1051	IR64_chr07	667500	672500
IR64	IR64_boundary_1052	IR64_chr07	1077500	1082500
IR64	IR64_boundary_1053	IR64_chr07	1127500	1132500
IR64	IR64_boundary_1054	IR64_chr07	1547500	1552500
IR64	IR64_boundary_1055	IR64_chr07	1652500	1657500
IR64	IR64_boundary_1056	IR64_chr07	1747500	1752500
IR64	IR64_boundary_1057	IR64_chr07	1887500	1892500
IR64	IR64_boundary_1058	IR64_chr07	1997500	2002500
IR64	IR64_boundary_1059	IR64_chr07	2067500	2072500
IR64	IR64_boundary_1060	IR64_chr07	2122500	2127500
IR64	IR64_boundary_1061	IR64_chr07	2277500	2282500
IR64	IR64_boundary_1062	IR64_chr07	2332500	2337500
IR64	IR64_boundary_1063	IR64_chr07	2407500	2412500
IR64	IR64_boundary_1064	IR64_chr07	2452500	2487500

IR64	IR64_boundary_1065	IR64_chr07	2542500	2547500
IR64	IR64_boundary_1066	IR64_chr07	3992500	3997500
IR64	IR64_boundary_1067	IR64_chr07	4092500	4097500
IR64	IR64_boundary_1068	IR64_chr07	4142500	4147500
IR64	IR64_boundary_1069	IR64_chr07	4227500	4232500
IR64	IR64_boundary_1070	IR64_chr07	4282500	4287500
IR64	IR64_boundary_1071	IR64_chr07	4352500	4387500
IR64	IR64_boundary_1072	IR64_chr07	4467500	4472500
IR64	IR64_boundary_1073	IR64_chr07	5312500	5360000
IR64	IR64_boundary_1074	IR64_chr07	6327500	6332500
IR64	IR64_boundary_1075	IR64_chr07	6397500	6402500
IR64	IR64_boundary_1076	IR64_chr07	6472500	6477500
IR64	IR64_boundary_1077	IR64_chr07	7007500	7012500
IR64	IR64_boundary_1078	IR64_chr07	7102500	7107500
IR64	IR64_boundary_1079	IR64_chr07	7442500	7447500
IR64	IR64_boundary_1080	IR64_chr07	7487500	7492500
IR64	IR64_boundary_1081	IR64_chr07	7567500	7572500
IR64	IR64_boundary_1082	IR64_chr07	7677500	7682500
IR64	IR64_boundary_1083	IR64_chr07	8097500	8102500
IR64	IR64_boundary_1084	IR64_chr07	8147500	8152500
IR64	IR64_boundary_1085	IR64_chr07	8247500	8252500
IR64	IR64_boundary_1086	IR64_chr07	8420000	8425000
IR64	IR64_boundary_1087	IR64_chr07	8512500	8517500
IR64	IR64_boundary_1088	IR64_chr07	8722500	8727500
IR64	IR64_boundary_1089	IR64_chr07	8797500	8832500
IR64	IR64_boundary_1090	IR64_chr07	10752500	10757500
IR64	IR64_boundary_1091	IR64_chr07	10862500	10892500
IR64	IR64_boundary_1092	IR64_chr07	11022500	11057500
IR64	IR64_boundary_1093	IR64_chr07	11575000	11580000
IR64	IR64_boundary_1094	IR64_chr07	11710000	11715000
IR64	IR64_boundary_1095	IR64_chr07	11802500	11847500
IR64	IR64_boundary_1096	IR64_chr07	11940000	11945000

IR64	IR64_boundary_1097	IR64_chr07	12332500	12337500
IR64	IR64_boundary_1098	IR64_chr07	12627500	12632500
IR64	IR64_boundary_1099	IR64_chr07	15447500	15492500
IR64	IR64_boundary_1100	IR64_chr07	15597500	15602500
IR64	IR64_boundary_1101	IR64_chr07	15942500	15947500
IR64	IR64_boundary_1102	IR64_chr07	15992500	15997500
IR64	IR64_boundary_1103	IR64_chr07	16047500	16052500
IR64	IR64_boundary_1104	IR64_chr07	16152500	16157500
IR64	IR64_boundary_1105	IR64_chr07	16222500	16227500
IR64	IR64_boundary_1106	IR64_chr07	16317500	16322500
IR64	IR64_boundary_1107	IR64_chr07	16407500	16412500
IR64	IR64_boundary_1108	IR64_chr07	16482500	16522500
IR64	IR64_boundary_1109	IR64_chr07	17377500	17382500
IR64	IR64_boundary_1110	IR64_chr07	17442500	17447500
IR64	IR64_boundary_1111	IR64_chr07	17552500	17557500
IR64	IR64_boundary_1112	IR64_chr07	17652500	17657500
IR64	IR64_boundary_1113	IR64_chr07	17732500	17737500
IR64	IR64_boundary_1114	IR64_chr07	18672500	18677500
IR64	IR64_boundary_1115	IR64_chr07	18752500	18757500
IR64	IR64_boundary_1116	IR64_chr07	18857500	18862500
IR64	IR64_boundary_1117	IR64_chr07	18902500	18907500
IR64	IR64_boundary_1118	IR64_chr07	18997500	19002500
IR64	IR64_boundary_1119	IR64_chr07	19222500	19227500
IR64	IR64_boundary_1120	IR64_chr07	19267500	19272500
IR64	IR64_boundary_1121	IR64_chr07	19687500	19692500
IR64	IR64_boundary_1122	IR64_chr07	19852500	19857500
IR64	IR64_boundary_1123	IR64_chr07	19922500	19927500
IR64	IR64_boundary_1124	IR64_chr07	20042500	20047500
IR64	IR64_boundary_1125	IR64_chr07	20532500	20537500
IR64	IR64_boundary_1126	IR64_chr07	20627500	20632500
IR64	IR64_boundary_1127	IR64_chr07	21092500	21097500
IR64	IR64_boundary_1128	IR64_chr07	21137500	21142500

IR64	IR64_boundary_1129	IR64_chr07	21207500	21212500
IR64	IR64_boundary_1130	IR64_chr07	21267500	21272500
IR64	IR64_boundary_1131	IR64_chr07	21322500	21327500
IR64	IR64_boundary_1132	IR64_chr07	21472500	21477500
IR64	IR64_boundary_1133	IR64_chr07	21522500	21527500
IR64	IR64_boundary_1134	IR64_chr07	21662500	21697500
IR64	IR64_boundary_1135	IR64_chr07	21802500	21807500
IR64	IR64_boundary_1136	IR64_chr07	21902500	21907500
IR64	IR64_boundary_1137	IR64_chr07	21962500	22002500
IR64	IR64_boundary_1138	IR64_chr07	22757500	22762500
IR64	IR64_boundary_1139	IR64_chr07	22827500	22832500
IR64	IR64_boundary_1140	IR64_chr07	22967500	22997500
IR64	IR64_boundary_1141	IR64_chr07	23097500	23137500
IR64	IR64_boundary_1142	IR64_chr07	23222500	23227500
IR64	IR64_boundary_1143	IR64_chr07	23282500	23287500
IR64	IR64_boundary_1144	IR64_chr07	23607500	23612500
IR64	IR64_boundary_1145	IR64_chr07	23662500	23667500
IR64	IR64_boundary_1146	IR64_chr07	23722500	23727500
IR64	IR64_boundary_1147	IR64_chr07	23767500	23877500
IR64	IR64_boundary_1148	IR64_chr07	23922500	23927500
IR64	IR64_boundary_1149	IR64_chr07	24232500	24237500
IR64	IR64_boundary_1150	IR64_chr07	24317500	24322500
IR64	IR64_boundary_1151	IR64_chr07	25657500	25662500
IR64	IR64_boundary_1152	IR64_chr07	25732500	25737500
IR64	IR64_boundary_1153	IR64_chr07	25847500	25852500
IR64	IR64_boundary_1154	IR64_chr07	25942500	25947500
IR64	IR64_boundary_1155	IR64_chr07	26227500	26232500
IR64	IR64_boundary_1156	IR64_chr07	26377500	26382500
IR64	IR64_boundary_1157	IR64_chr07	26437500	26442500
IR64	IR64_boundary_1158	IR64_chr07	26482500	26487500
IR64	IR64_boundary_1159	IR64_chr07	26552500	26557500
IR64	IR64_boundary_1160	IR64_chr07	26982500	27017500

IR64	IR64_boundary_1161	IR64_chr07	27077500	27082500
IR64	IR64_boundary_1162	IR64_chr07	27122500	27202500
IR64	IR64_boundary_1163	IR64_chr07	27287500	27292500
IR64	IR64_boundary_1164	IR64_chr07	27387500	27392500
IR64	IR64_boundary_1165	IR64_chr07	27622500	27697500
IR64	IR64_boundary_1166	IR64_chr07	27762500	27792500
IR64	IR64_boundary_1167	IR64_chr07	27852500	27857500
IR64	IR64_boundary_1168	IR64_chr07	27927500	27962500
IR64	IR64_boundary_1169	IR64_chr07	28057500	28062500
IR64	IR64_boundary_1170	IR64_chr07	28312500	28317500
IR64	IR64_boundary_1171	IR64_chr07	28382500	28387500
IR64	IR64_boundary_1172	IR64_chr07	28452500	28457500
IR64	IR64_boundary_1173	IR64_chr07	28562500	28607500
IR64	IR64_boundary_1174	IR64_chr07	28692500	28732500
IR64	IR64_boundary_1175	IR64_chr07	28877500	28947500
IR64	IR64_boundary_1176	IR64_chr07	29322500	29327500
IR64	IR64_boundary_1177	IR64_chr07	29382500	29387500
IR64	IR64_boundary_1178	IR64_chr07	30462500	30467500
IR64	IR64_boundary_1179	IR64_chr07	30527500	30532500
IR64	IR64_boundary_1180	IR64_chr08	357500	362500
IR64	IR64_boundary_1181	IR64_chr08	457500	502500
IR64	IR64_boundary_1182	IR64_chr08	822500	862500
IR64	IR64_boundary_1183	IR64_chr08	927500	932500
IR64	IR64_boundary_1184	IR64_chr08	1002500	1007500
IR64	IR64_boundary_1185	IR64_chr08	1082500	1087500
IR64	IR64_boundary_1186	IR64_chr08	1392500	1397500
IR64	IR64_boundary_1187	IR64_chr08	1452500	1457500
IR64	IR64_boundary_1188	IR64_chr08	1762500	1767500
IR64	IR64_boundary_1189	IR64_chr08	1817500	1822500
IR64	IR64_boundary_1190	IR64_chr08	1887500	1892500
IR64	IR64_boundary_1191	IR64_chr08	1987500	2022500
IR64	IR64_boundary_1192	IR64_chr08	3152500	3157500

IR64	IR64_boundary_1193	IR64_chr08	3262500	3267500
IR64	IR64_boundary_1194	IR64_chr08	3742500	3747500
IR64	IR64_boundary_1195	IR64_chr08	3832500	3837500
IR64	IR64_boundary_1196	IR64_chr08	3897500	3902500
IR64	IR64_boundary_1197	IR64_chr08	3952500	3957500
IR64	IR64_boundary_1198	IR64_chr08	4057500	4102500
IR64	IR64_boundary_1199	IR64_chr08	4212500	4217500
IR64	IR64_boundary_1200	IR64_chr08	4262500	4297500
IR64	IR64_boundary_1201	IR64_chr08	4357500	4362500
IR64	IR64_boundary_1202	IR64_chr08	4457500	4462500
IR64	IR64_boundary_1203	IR64_chr08	4517500	4597500
IR64	IR64_boundary_1204	IR64_chr08	5152500	5157500
IR64	IR64_boundary_1205	IR64_chr08	5242500	5247500
IR64	IR64_boundary_1206	IR64_chr08	5552500	5557500
IR64	IR64_boundary_1207	IR64_chr08	5602500	5607500
IR64	IR64_boundary_1208	IR64_chr08	5692500	5697500
IR64	IR64_boundary_1209	IR64_chr08	5787500	5792500
IR64	IR64_boundary_1210	IR64_chr08	5872500	5877500
IR64	IR64_boundary_1211	IR64_chr08	5942500	5947500
IR64	IR64_boundary_1212	IR64_chr08	6022500	6027500
IR64	IR64_boundary_1213	IR64_chr08	7972500	7977500
IR64	IR64_boundary_1214	IR64_chr08	8052500	8057500
IR64	IR64_boundary_1215	IR64_chr08	8192500	8197500
IR64	IR64_boundary_1216	IR64_chr08	8277500	8312500
IR64	IR64_boundary_1217	IR64_chr08	8372500	8377500
IR64	IR64_boundary_1218	IR64_chr08	8517500	8522500
IR64	IR64_boundary_1219	IR64_chr08	8577500	8582500
IR64	IR64_boundary_1220	IR64_chr08	8822500	8827500
IR64	IR64_boundary_1221	IR64_chr08	8922500	8927500
IR64	IR64_boundary_1222	IR64_chr08	8977500	8982500
IR64	IR64_boundary_1223	IR64_chr08	9072500	9107500
IR64	IR64_boundary_1224	IR64_chr08	9357500	9362500

IR64	IR64_boundary_1225	IR64_chr08	9462500	9467500
IR64	IR64_boundary_1226	IR64_chr08	10152500	10157500
IR64	IR64_boundary_1227	IR64_chr08	10202500	10207500
IR64	IR64_boundary_1228	IR64_chr08	10272500	10277500
IR64	IR64_boundary_1229	IR64_chr08	10322500	10327500
IR64	IR64_boundary_1230	IR64_chr08	11057500	11062500
IR64	IR64_boundary_1231	IR64_chr08	11132500	11137500
IR64	IR64_boundary_1232	IR64_chr08	11422500	11427500
IR64	IR64_boundary_1233	IR64_chr08	11497500	11502500
IR64	IR64_boundary_1234	IR64_chr08	11557500	11562500
IR64	IR64_boundary_1235	IR64_chr08	11607500	11612500
IR64	IR64_boundary_1236	IR64_chr08	11727500	11732500
IR64	IR64_boundary_1237	IR64_chr08	11887500	11892500
IR64	IR64_boundary_1238	IR64_chr08	15387500	15392500
IR64	IR64_boundary_1239	IR64_chr08	15447500	15452500
IR64	IR64_boundary_1240	IR64_chr08	15692500	15697500
IR64	IR64_boundary_1241	IR64_chr08	15837500	15842500
IR64	IR64_boundary_1242	IR64_chr08	16237500	16242500
IR64	IR64_boundary_1243	IR64_chr08	16307500	16312500
IR64	IR64_boundary_1244	IR64_chr08	16412500	16417500
IR64	IR64_boundary_1245	IR64_chr08	16507500	16512500
IR64	IR64_boundary_1246	IR64_chr08	18532500	18537500
IR64	IR64_boundary_1247	IR64_chr08	18612500	18617500
IR64	IR64_boundary_1248	IR64_chr08	18832500	18867500
IR64	IR64_boundary_1249	IR64_chr08	19002500	19007500
IR64	IR64_boundary_1250	IR64_chr08	19472500	19477500
IR64	IR64_boundary_1251	IR64_chr08	19582500	19587500
IR64	IR64_boundary_1252	IR64_chr08	19667500	19672500
IR64	IR64_boundary_1253	IR64_chr08	20502500	20507500
IR64	IR64_boundary_1254	IR64_chr08	20567500	20572500
IR64	IR64_boundary_1255	IR64_chr08	21277500	21362500
IR64	IR64_boundary_1256	IR64_chr08	21407500	21442500

IR64	IR64_boundary_1257	IR64_chr08	21502500	21507500
IR64	IR64_boundary_1258	IR64_chr08	21652500	21657500
IR64	IR64_boundary_1259	IR64_chr08	21722500	21727500
IR64	IR64_boundary_1260	IR64_chr08	21862500	21907500
IR64	IR64_boundary_1261	IR64_chr08	21972500	21977500
IR64	IR64_boundary_1262	IR64_chr08	22047500	22052500
IR64	IR64_boundary_1263	IR64_chr08	22095000	22100000
IR64	IR64_boundary_1264	IR64_chr08	22157500	22162500
IR64	IR64_boundary_1265	IR64_chr08	22202500	22207500
IR64	IR64_boundary_1266	IR64_chr08	22272500	22327500
IR64	IR64_boundary_1267	IR64_chr08	22387500	22392500
IR64	IR64_boundary_1268	IR64_chr08	22492500	22497500
IR64	IR64_boundary_1269	IR64_chr08	22582500	22587500
IR64	IR64_boundary_1270	IR64_chr08	22657500	22662500
IR64	IR64_boundary_1271	IR64_chr08	22712500	22717500
IR64	IR64_boundary_1272	IR64_chr08	22777500	22822500
IR64	IR64_boundary_1273	IR64_chr08	23607500	23642500
IR64	IR64_boundary_1274	IR64_chr08	23707500	23747500
IR64	IR64_boundary_1275	IR64_chr08	23812500	23817500
IR64	IR64_boundary_1276	IR64_chr08	23927500	23932500
IR64	IR64_boundary_1277	IR64_chr08	24007500	24012500
IR64	IR64_boundary_1278	IR64_chr08	25412500	25457500
IR64	IR64_boundary_1279	IR64_chr08	25777500	25782500
IR64	IR64_boundary_1280	IR64_chr08	25852500	25857500
IR64	IR64_boundary_1281	IR64_chr08	25902500	25977500
IR64	IR64_boundary_1282	IR64_chr08	26942500	26947500
IR64	IR64_boundary_1283	IR64_chr08	27007500	27012500
IR64	IR64_boundary_1284	IR64_chr08	27067500	27072500
IR64	IR64_boundary_1285	IR64_chr08	27167500	27172500
IR64	IR64_boundary_1286	IR64_chr08	27262500	27267500
IR64	IR64_boundary_1287	IR64_chr08	27312500	27317500
IR64	IR64_boundary_1288	IR64_chr08	27392500	27397500

IR64	IR64_boundary_1289	IR64_chr08	27497500	27502500
IR64	IR64_boundary_1290	IR64_chr08	27557500	27562500
IR64	IR64_boundary_1291	IR64_chr08	27622500	27662500
IR64	IR64_boundary_1292	IR64_chr08	27702500	27707500
IR64	IR64_boundary_1293	IR64_chr08	27782500	27827500
IR64	IR64_boundary_1294	IR64_chr08	27882500	27887500
IR64	IR64_boundary_1295	IR64_chr08	27977500	27982500
IR64	IR64_boundary_1296	IR64_chr08	28257500	28262500
IR64	IR64_boundary_1297	IR64_chr08	28307500	28312500
IR64	IR64_boundary_1298	IR64_chr08	28392500	28397500
IR64	IR64_boundary_1299	IR64_chr08	28452500	28457500
IR64	IR64_boundary_1300	IR64_chr08	28582500	28612500
IR64	IR64_boundary_1301	IR64_chr08	28677500	28722500
IR64	IR64_boundary_1302	IR64_chr08	28787500	28792500
IR64	IR64_boundary_1303	IR64_chr08	28857500	28887500
IR64	IR64_boundary_1304	IR64_chr08	28942500	28987500
IR64	IR64_boundary_1305	IR64_chr08	29092500	29097500
IR64	IR64_boundary_1306	IR64_chr08	29187500	29192500
IR64	IR64_boundary_1307	IR64_chr08	29322500	29327500
IR64	IR64_boundary_1308	IR64_chr08	29422500	29427500
IR64	IR64_boundary_1309	IR64_chr08	29467500	29472500
IR64	IR64_boundary_1310	IR64_chr08	29597500	29637500
IR64	IR64_boundary_1311	IR64_chr09	197500	202500
IR64	IR64_boundary_1312	IR64_chr09	272500	277500
IR64	IR64_boundary_1313	IR64_chr09	342500	347500
IR64	IR64_boundary_1314	IR64_chr09	492500	497500
IR64	IR64_boundary_1315	IR64_chr09	580000	585000
IR64	IR64_boundary_1316	IR64_chr09	1062500	1067500
IR64	IR64_boundary_1317	IR64_chr09	1112500	1152500
IR64	IR64_boundary_1318	IR64_chr09	1267500	1272500
IR64	IR64_boundary_1319	IR64_chr09	1467500	1472500
IR64	IR64_boundary_1320	IR64_chr09	1992500	2037500

IR64	IR64_boundary_1321	IR64_chr09	2117500	2122500
IR64	IR64_boundary_1322	IR64_chr09	2247500	2252500
IR64	IR64_boundary_1323	IR64_chr09	2582500	2587500
IR64	IR64_boundary_1324	IR64_chr09	2802500	2807500
IR64	IR64_boundary_1325	IR64_chr09	2922500	2927500
IR64	IR64_boundary_1326	IR64_chr09	3667500	3672500
IR64	IR64_boundary_1327	IR64_chr09	3967500	3972500
IR64	IR64_boundary_1328	IR64_chr09	4447500	4452500
IR64	IR64_boundary_1329	IR64_chr09	4552500	4557500
IR64	IR64_boundary_1330	IR64_chr09	4602500	4607500
IR64	IR64_boundary_1331	IR64_chr09	4687500	4692500
IR64	IR64_boundary_1332	IR64_chr09	5277500	5282500
IR64	IR64_boundary_1333	IR64_chr09	5367500	5372500
IR64	IR64_boundary_1334	IR64_chr09	6807500	6812500
IR64	IR64_boundary_1335	IR64_chr09	6872500	6917500
IR64	IR64_boundary_1336	IR64_chr09	7542500	7582500
IR64	IR64_boundary_1337	IR64_chr09	8887500	8952500
IR64	IR64_boundary_1338	IR64_chr09	9017500	9022500
IR64	IR64_boundary_1339	IR64_chr09	9342500	9347500
IR64	IR64_boundary_1340	IR64_chr09	9432500	9437500
IR64	IR64_boundary_1341	IR64_chr09	9552500	9557500
IR64	IR64_boundary_1342	IR64_chr09	9602500	9607500
IR64	IR64_boundary_1343	IR64_chr09	10802500	10807500
IR64	IR64_boundary_1344	IR64_chr09	10852500	10857500
IR64	IR64_boundary_1345	IR64_chr09	10922500	10927500
IR64	IR64_boundary_1346	IR64_chr09	12822500	12892500
IR64	IR64_boundary_1347	IR64_chr09	12942500	12947500
IR64	IR64_boundary_1348	IR64_chr09	13987500	13992500
IR64	IR64_boundary_1349	IR64_chr09	14082500	14087500
IR64	IR64_boundary_1350	IR64_chr09	15057500	15062500
IR64	IR64_boundary_1351	IR64_chr09	15127500	15132500
IR64	IR64_boundary_1352	IR64_chr09	15172500	15242500

IR64	IR64_boundary_1353	IR64_chr09	15587500	15592500
IR64	IR64_boundary_1354	IR64_chr09	15657500	15662500
IR64	IR64_boundary_1355	IR64_chr09	15857500	15897500
IR64	IR64_boundary_1356	IR64_chr09	15967500	16012500
IR64	IR64_boundary_1357	IR64_chr09	16237500	16242500
IR64	IR64_boundary_1358	IR64_chr09	16392500	16457500
IR64	IR64_boundary_1359	IR64_chr09	16497500	16502500
IR64	IR64_boundary_1360	IR64_chr09	16577500	16642500
IR64	IR64_boundary_1361	IR64_chr09	17232500	17237500
IR64	IR64_boundary_1362	IR64_chr09	17322500	17327500
IR64	IR64_boundary_1363	IR64_chr09	17442500	17447500
IR64	IR64_boundary_1364	IR64_chr09	17537500	17542500
IR64	IR64_boundary_1365	IR64_chr09	18152500	18182500
IR64	IR64_boundary_1366	IR64_chr09	18222500	18297500
IR64	IR64_boundary_1367	IR64_chr09	18372500	18377500
IR64	IR64_boundary_1368	IR64_chr09	18712500	18717500
IR64	IR64_boundary_1369	IR64_chr09	18767500	18772500
IR64	IR64_boundary_1370	IR64_chr09	18872500	18877500
IR64	IR64_boundary_1371	IR64_chr09	18922500	18927500
IR64	IR64_boundary_1372	IR64_chr09	19017500	19052500
IR64	IR64_boundary_1373	IR64_chr09	19112500	19117500
IR64	IR64_boundary_1374	IR64_chr09	19382500	19387500
IR64	IR64_boundary_1375	IR64_chr09	19442500	19447500
IR64	IR64_boundary_1376	IR64_chr09	19627500	19662500
IR64	IR64_boundary_1377	IR64_chr09	19732500	19737500
IR64	IR64_boundary_1378	IR64_chr09	19777500	19782500
IR64	IR64_boundary_1379	IR64_chr09	20142500	20182500
IR64	IR64_boundary_1380	IR64_chr09	20282500	20287500
IR64	IR64_boundary_1381	IR64_chr09	20327500	20332500
IR64	IR64_boundary_1382	IR64_chr09	20412500	20417500
IR64	IR64_boundary_1383	IR64_chr09	20672500	20677500
IR64	IR64_boundary_1384	IR64_chr09	20727500	20732500

IR64	IR64_boundary_1385	IR64_chr09	20852500	20857500
IR64	IR64_boundary_1386	IR64_chr09	20957500	20962500
IR64	IR64_boundary_1387	IR64_chr09	21037500	21042500
IR64	IR64_boundary_1388	IR64_chr09	21617500	21622500
IR64	IR64_boundary_1389	IR64_chr09	21677500	21712500
IR64	IR64_boundary_1390	IR64_chr09	21897500	21902500
IR64	IR64_boundary_1391	IR64_chr09	21982500	21987500
IR64	IR64_boundary_1392	IR64_chr09	22107500	22112500
IR64	IR64_boundary_1393	IR64_chr09	22167500	22172500
IR64	IR64_boundary_1394	IR64_chr09	22217500	22222500
IR64	IR64_boundary_1395	IR64_chr09	22267500	22307500
IR64	IR64_boundary_1396	IR64_chr09	22612500	22617500
IR64	IR64_boundary_1397	IR64_chr09	22682500	22687500
IR64	IR64_boundary_1398	IR64_chr09	22747500	22752500
IR64	IR64_boundary_1399	IR64_chr09	22827500	22832500
IR64	IR64_boundary_1400	IR64_chr09	23267500	23312500
IR64	IR64_boundary_1401	IR64_chr09	24142500	24147500
IR64	IR64_boundary_1402	IR64_chr09	24187500	24192500
IR64	IR64_boundary_1403	IR64_chr10	1292500	1297500
IR64	IR64_boundary_1404	IR64_chr10	1387500	1392500
IR64	IR64_boundary_1405	IR64_chr10	1452500	1457500
IR64	IR64_boundary_1406	IR64_chr10	1527500	1532500
IR64	IR64_boundary_1407	IR64_chr10	1577500	1582500
IR64	IR64_boundary_1408	IR64_chr10	2047500	2052500
IR64	IR64_boundary_1409	IR64_chr10	2142500	2147500
IR64	IR64_boundary_1410	IR64_chr10	2872500	2877500
IR64	IR64_boundary_1411	IR64_chr10	2917500	2922500
IR64	IR64_boundary_1412	IR64_chr10	3027500	3032500
IR64	IR64_boundary_1413	IR64_chr10	3172500	3177500
IR64	IR64_boundary_1414	IR64_chr10	3277500	3282500
IR64	IR64_boundary_1415	IR64_chr10	3367500	3372500
IR64	IR64_boundary_1416	IR64_chr10	3582500	3587500

IR64	IR64_boundary_1417	IR64_chr10	3672500	3737500
IR64	IR64_boundary_1418	IR64_chr10	4267500	4312500
IR64	IR64_boundary_1419	IR64_chr10	4362500	4367500
IR64	IR64_boundary_1420	IR64_chr10	4430000	4435000
IR64	IR64_boundary_1421	IR64_chr10	4527500	4532500
IR64	IR64_boundary_1422	IR64_chr10	4702500	4707500
IR64	IR64_boundary_1423	IR64_chr10	4777500	4782500
IR64	IR64_boundary_1424	IR64_chr10	4897500	4902500
IR64	IR64_boundary_1425	IR64_chr10	5487500	5492500
IR64	IR64_boundary_1426	IR64_chr10	5587500	5592500
IR64	IR64_boundary_1427	IR64_chr10	6027500	6032500
IR64	IR64_boundary_1428	IR64_chr10	6092500	6097500
IR64	IR64_boundary_1429	IR64_chr10	6412500	6417500
IR64	IR64_boundary_1430	IR64_chr10	6457500	6462500
IR64	IR64_boundary_1431	IR64_chr10	6527500	6532500
IR64	IR64_boundary_1432	IR64_chr10	6912500	6917500
IR64	IR64_boundary_1433	IR64_chr10	6962500	6967500
IR64	IR64_boundary_1434	IR64_chr10	8362500	8367500
IR64	IR64_boundary_1435	IR64_chr10	8472500	8477500
IR64	IR64_boundary_1436	IR64_chr10	9852500	9895000
IR64	IR64_boundary_1437	IR64_chr10	9972500	10020000
IR64	IR64_boundary_1438	IR64_chr10	10487500	10492500
IR64	IR64_boundary_1439	IR64_chr10	10592500	10597500
IR64	IR64_boundary_1440	IR64_chr10	11592500	11597500
IR64	IR64_boundary_1441	IR64_chr10	11677500	11682500
IR64	IR64_boundary_1442	IR64_chr10	11787500	11792500
IR64	IR64_boundary_1443	IR64_chr10	12402500	12407500
IR64	IR64_boundary_1444	IR64_chr10	12547500	12632500
IR64	IR64_boundary_1445	IR64_chr10	12682500	12687500
IR64	IR64_boundary_1446	IR64_chr10	12977500	12982500
IR64	IR64_boundary_1447	IR64_chr10	13042500	13047500
IR64	IR64_boundary_1448	IR64_chr10	13147500	13152500

IR64	IR64_boundary_1449	IR64_chr10	13267500	13272500
IR64	IR64_boundary_1450	IR64_chr10	13327500	13332500
IR64	IR64_boundary_1451	IR64_chr10	13407500	13412500
IR64	IR64_boundary_1452	IR64_chr10	13532500	13537500
IR64	IR64_boundary_1453	IR64_chr10	13672500	13677500
IR64	IR64_boundary_1454	IR64_chr10	14867500	14872500
IR64	IR64_boundary_1455	IR64_chr10	14927500	14932500
IR64	IR64_boundary_1456	IR64_chr10	15007500	15012500
IR64	IR64_boundary_1457	IR64_chr10	15162500	15167500
IR64	IR64_boundary_1458	IR64_chr10	15247500	15252500
IR64	IR64_boundary_1459	IR64_chr10	15372500	15377500
IR64	IR64_boundary_1460	IR64_chr10	15462500	15467500
IR64	IR64_boundary_1461	IR64_chr10	15517500	15522500
IR64	IR64_boundary_1462	IR64_chr10	15772500	15802500
IR64	IR64_boundary_1463	IR64_chr10	16217500	16222500
IR64	IR64_boundary_1464	IR64_chr10	16277500	16282500
IR64	IR64_boundary_1465	IR64_chr10	16422500	16427500
IR64	IR64_boundary_1466	IR64_chr10	16502500	16507500
IR64	IR64_boundary_1467	IR64_chr10	17947500	17952500
IR64	IR64_boundary_1468	IR64_chr10	18027500	18032500
IR64	IR64_boundary_1469	IR64_chr10	19002500	19007500
IR64	IR64_boundary_1470	IR64_chr10	19082500	19087500
IR64	IR64_boundary_1471	IR64_chr10	19182500	19187500
IR64	IR64_boundary_1472	IR64_chr10	19247500	19252500
IR64	IR64_boundary_1473	IR64_chr10	19352500	19357500
IR64	IR64_boundary_1474	IR64_chr10	19872500	19912500
IR64	IR64_boundary_1475	IR64_chr10	20757500	20762500
IR64	IR64_boundary_1476	IR64_chr10	20817500	20822500
IR64	IR64_boundary_1477	IR64_chr10	21207500	21212500
IR64	IR64_boundary_1478	IR64_chr10	21312500	21317500
IR64	IR64_boundary_1479	IR64_chr10	21357500	21362500
IR64	IR64_boundary_1480	IR64_chr10	21422500	21467500

IR64	IR64_boundary_1481	IR64_chr10	21717500	21722500
IR64	IR64_boundary_1482	IR64_chr10	21767500	21772500
IR64	IR64_boundary_1483	IR64_chr10	21837500	21882500
IR64	IR64_boundary_1484	IR64_chr10	22057500	22062500
IR64	IR64_boundary_1485	IR64_chr10	22112500	22117500
IR64	IR64_boundary_1486	IR64_chr10	22202500	22242500
IR64	IR64_boundary_1487	IR64_chr10	22402500	22407500
IR64	IR64_boundary_1488	IR64_chr10	22447500	22482500
IR64	IR64_boundary_1489	IR64_chr10	22922500	22927500
IR64	IR64_boundary_1490	IR64_chr10	22997500	23002500
IR64	IR64_boundary_1491	IR64_chr10	23057500	23062500
IR64	IR64_boundary_1492	IR64_chr10	23107500	23112500
IR64	IR64_boundary_1493	IR64_chr10	23377500	23382500
IR64	IR64_boundary_1494	IR64_chr10	23432500	23437500
IR64	IR64_boundary_1495	IR64_chr10	23522500	23527500
IR64	IR64_boundary_1496	IR64_chr10	23572500	23577500
IR64	IR64_boundary_1497	IR64_chr10	23662500	23667500
IR64	IR64_boundary_1498	IR64_chr10	23792500	23797500
IR64	IR64_boundary_1499	IR64_chr10	23867500	23872500
IR64	IR64_boundary_1500	IR64_chr10	23947500	23952500
IR64	IR64_boundary_1501	IR64_chr10	24057500	24127500
IR64	IR64_boundary_1502	IR64_chr10	24222500	24267500
IR64	IR64_boundary_1503	IR64_chr10	24362500	24367500
IR64	IR64_boundary_1504	IR64_chr10	24502500	24542500
IR64	IR64_boundary_1505	IR64_chr10	24892500	24897500
IR64	IR64_boundary_1506	IR64_chr10	24967500	24972500
IR64	IR64_boundary_1507	IR64_chr10	25037500	25082500
IR64	IR64_boundary_1508	IR64_chr10	25182500	25187500
IR64	IR64_boundary_1509	IR64_chr10	25262500	25267500
IR64	IR64_boundary_1510	IR64_chr11	1452500	1457500
IR64	IR64_boundary_1511	IR64_chr11	1512500	1517500
IR64	IR64_boundary_1512	IR64_chr11	1582500	1587500

IR64	IR64_boundary_1513	IR64_chr11	3692500	3697500
IR64	IR64_boundary_1514	IR64_chr11	3752500	3757500
IR64	IR64_boundary_1515	IR64_chr11	4117500	4122500
IR64	IR64_boundary_1516	IR64_chr11	4172500	4177500
IR64	IR64_boundary_1517	IR64_chr11	4232500	4237500
IR64	IR64_boundary_1518	IR64_chr11	4292500	4322500
IR64	IR64_boundary_1519	IR64_chr11	5037500	5042500
IR64	IR64_boundary_1520	IR64_chr11	5102500	5142500
IR64	IR64_boundary_1521	IR64_chr11	5702500	5707500
IR64	IR64_boundary_1522	IR64_chr11	5832500	5837500
IR64	IR64_boundary_1523	IR64_chr11	5937500	5942500
IR64	IR64_boundary_1524	IR64_chr11	6127500	6157500
IR64	IR64_boundary_1525	IR64_chr11	6222500	6227500
IR64	IR64_boundary_1526	IR64_chr11	6357500	6362500
IR64	IR64_boundary_1527	IR64_chr11	6967500	6972500
IR64	IR64_boundary_1528	IR64_chr11	7042500	7047500
IR64	IR64_boundary_1529	IR64_chr11	7117500	7122500
IR64	IR64_boundary_1530	IR64_chr11	7222500	7227500
IR64	IR64_boundary_1531	IR64_chr11	7342500	7347500
IR64	IR64_boundary_1532	IR64_chr11	7437500	7442500
IR64	IR64_boundary_1533	IR64_chr11	7507500	7512500
IR64	IR64_boundary_1534	IR64_chr11	8912500	8917500
IR64	IR64_boundary_1535	IR64_chr11	9007500	9012500
IR64	IR64_boundary_1536	IR64_chr11	9102500	9107500
IR64	IR64_boundary_1537	IR64_chr11	11222500	11227500
IR64	IR64_boundary_1538	IR64_chr11	11330000	11335000
IR64	IR64_boundary_1539	IR64_chr11	11502500	11507500
IR64	IR64_boundary_1540	IR64_chr11	11562500	11567500
IR64	IR64_boundary_1541	IR64_chr11	11737500	11742500
IR64	IR64_boundary_1542	IR64_chr11	11822500	11827500
IR64	IR64_boundary_1543	IR64_chr11	12587500	12592500
IR64	IR64_boundary_1544	IR64_chr11	12712500	12717500

IR64	IR64_boundary_1545	IR64_chr11	14772500	14777500
IR64	IR64_boundary_1546	IR64_chr11	14907500	14912500
IR64	IR64_boundary_1547	IR64_chr11	15287500	15332500
IR64	IR64_boundary_1548	IR64_chr11	15930000	15935000
IR64	IR64_boundary_1549	IR64_chr11	16107500	16112500
IR64	IR64_boundary_1550	IR64_chr11	17022500	17027500
IR64	IR64_boundary_1551	IR64_chr11	17092500	17097500
IR64	IR64_boundary_1552	IR64_chr11	17182500	17187500
IR64	IR64_boundary_1553	IR64_chr11	17427500	17432500
IR64	IR64_boundary_1554	IR64_chr11	17537500	17542500
IR64	IR64_boundary_1555	IR64_chr11	17897500	17902500
IR64	IR64_boundary_1556	IR64_chr11	18002500	18007500
IR64	IR64_boundary_1557	IR64_chr11	18477500	18482500
IR64	IR64_boundary_1558	IR64_chr11	18547500	18552500
IR64	IR64_boundary_1559	IR64_chr11	18882500	18887500
IR64	IR64_boundary_1560	IR64_chr11	19132500	19137500
IR64	IR64_boundary_1561	IR64_chr11	19272500	19317500
IR64	IR64_boundary_1562	IR64_chr11	19362500	19367500
IR64	IR64_boundary_1563	IR64_chr11	19417500	19422500
IR64	IR64_boundary_1564	IR64_chr11	19502500	19507500
IR64	IR64_boundary_1565	IR64_chr11	19627500	19632500
IR64	IR64_boundary_1566	IR64_chr11	19742500	19747500
IR64	IR64_boundary_1567	IR64_chr11	19847500	19882500
IR64	IR64_boundary_1568	IR64_chr11	19992500	19997500
IR64	IR64_boundary_1569	IR64_chr11	20082500	20087500
IR64	IR64_boundary_1570	IR64_chr11	20662500	20667500
IR64	IR64_boundary_1571	IR64_chr11	20707500	20712500
IR64	IR64_boundary_1572	IR64_chr11	20762500	20767500
IR64	IR64_boundary_1573	IR64_chr11	21532500	21537500
IR64	IR64_boundary_1574	IR64_chr11	21582500	21587500
IR64	IR64_boundary_1575	IR64_chr11	22502500	22537500
IR64	IR64_boundary_1576	IR64_chr11	22627500	22632500

IR64	IR64_boundary_1577	IR64_chr11	23567500	23572500
IR64	IR64_boundary_1578	IR64_chr11	23687500	23692500
IR64	IR64_boundary_1579	IR64_chr11	23740000	23745000
IR64	IR64_boundary_1580	IR64_chr11	23812500	23817500
IR64	IR64_boundary_1581	IR64_chr11	23857500	23862500
IR64	IR64_boundary_1582	IR64_chr11	23922500	23927500
IR64	IR64_boundary_1583	IR64_chr11	24107500	24112500
IR64	IR64_boundary_1584	IR64_chr11	24162500	24167500
IR64	IR64_boundary_1585	IR64_chr11	24522500	24527500
IR64	IR64_boundary_1586	IR64_chr11	24567500	24572500
IR64	IR64_boundary_1587	IR64_chr11	25382500	25452500
IR64	IR64_boundary_1588	IR64_chr11	26222500	26227500
IR64	IR64_boundary_1589	IR64_chr11	26282500	26287500
IR64	IR64_boundary_1590	IR64_chr11	26327500	26332500
IR64	IR64_boundary_1591	IR64_chr11	27782500	27787500
IR64	IR64_boundary_1592	IR64_chr11	27842500	27847500
IR64	IR64_boundary_1593	IR64_chr11	27972500	27977500
IR64	IR64_boundary_1594	IR64_chr11	28202500	28207500
IR64	IR64_boundary_1595	IR64_chr11	28647500	28652500
IR64	IR64_boundary_1596	IR64_chr11	28712500	28752500
IR64	IR64_boundary_1597	IR64_chr11	28807500	28812500
IR64	IR64_boundary_1598	IR64_chr11	28905000	28910000
IR64	IR64_boundary_1599	IR64_chr11	29312500	29317500
IR64	IR64_boundary_1600	IR64_chr11	29387500	29392500
IR64	IR64_boundary_1601	IR64_chr11	29522500	29527500
IR64	IR64_boundary_1602	IR64_chr11	29602500	29607500
IR64	IR64_boundary_1603	IR64_chr11	29657500	29662500
IR64	IR64_boundary_1604	IR64_chr11	29722500	29727500
IR64	IR64_boundary_1605	IR64_chr11	29772500	29777500
IR64	IR64_boundary_1606	IR64_chr11	29852500	29857500
IR64	IR64_boundary_1607	IR64_chr11	30007500	30012500
IR64	IR64_boundary_1608	IR64_chr11	30087500	30092500

IR64	IR64_boundary_1609	IR64_chr11	30407500	30412500
IR64	IR64_boundary_1610	IR64_chr11	30637500	30642500
IR64	IR64_boundary_1611	IR64_chr11	30752500	30757500
IR64	IR64_boundary_1612	IR64_chr11	30827500	30832500
IR64	IR64_boundary_1613	IR64_chr12	182500	187500
IR64	IR64_boundary_1614	IR64_chr12	255000	260000
IR64	IR64_boundary_1615	IR64_chr12	332500	367500
IR64	IR64_boundary_1616	IR64_chr12	417500	422500
IR64	IR64_boundary_1617	IR64_chr12	847500	852500
IR64	IR64_boundary_1618	IR64_chr12	892500	937500
IR64	IR64_boundary_1619	IR64_chr12	1012500	1042500
IR64	IR64_boundary_1620	IR64_chr12	1217500	1222500
IR64	IR64_boundary_1621	IR64_chr12	1332500	1337500
IR64	IR64_boundary_1622	IR64_chr12	1382500	1387500
IR64	IR64_boundary_1623	IR64_chr12	1632500	1637500
IR64	IR64_boundary_1624	IR64_chr12	1682500	1687500
IR64	IR64_boundary_1625	IR64_chr12	1787500	1792500
IR64	IR64_boundary_1626	IR64_chr12	1942500	1947500
IR64	IR64_boundary_1627	IR64_chr12	2002500	2007500
IR64	IR64_boundary_1628	IR64_chr12	2057500	2062500
IR64	IR64_boundary_1629	IR64_chr12	2157500	2162500
IR64	IR64_boundary_1630	IR64_chr12	2207500	2242500
IR64	IR64_boundary_1631	IR64_chr12	2362500	2367500
IR64	IR64_boundary_1632	IR64_chr12	2437500	2442500
IR64	IR64_boundary_1633	IR64_chr12	2522500	2597500
IR64	IR64_boundary_1634	IR64_chr12	2877500	2882500
IR64	IR64_boundary_1635	IR64_chr12	2932500	2937500
IR64	IR64_boundary_1636	IR64_chr12	2977500	3017500
IR64	IR64_boundary_1637	IR64_chr12	3337500	3367500
IR64	IR64_boundary_1638	IR64_chr12	3412500	3417500
IR64	IR64_boundary_1639	IR64_chr12	3557500	3562500
IR64	IR64_boundary_1640	IR64_chr12	3652500	3657500

IR64	IR64_boundary_1641	IR64_chr12	3722500	3727500
IR64	IR64_boundary_1642	IR64_chr12	3847500	3852500
IR64	IR64_boundary_1643	IR64_chr12	3917500	3922500
IR64	IR64_boundary_1644	IR64_chr12	4202500	4242500
IR64	IR64_boundary_1645	IR64_chr12	4327500	4332500
IR64	IR64_boundary_1646	IR64_chr12	4417500	4422500
IR64	IR64_boundary_1647	IR64_chr12	4487500	4492500
IR64	IR64_boundary_1648	IR64_chr12	6537500	6542500
IR64	IR64_boundary_1649	IR64_chr12	6592500	6637500
IR64	IR64_boundary_1650	IR64_chr12	6697500	6737500
IR64	IR64_boundary_1651	IR64_chr12	6782500	6787500
IR64	IR64_boundary_1652	IR64_chr12	6917500	6922500
IR64	IR64_boundary_1653	IR64_chr12	7002500	7007500
IR64	IR64_boundary_1654	IR64_chr12	7082500	7087500
IR64	IR64_boundary_1655	IR64_chr12	8472500	8477500
IR64	IR64_boundary_1656	IR64_chr12	8572500	8577500
IR64	IR64_boundary_1657	IR64_chr12	8702500	8707500
IR64	IR64_boundary_1658	IR64_chr12	8977500	8982500
IR64	IR64_boundary_1659	IR64_chr12	9097500	9102500
IR64	IR64_boundary_1660	IR64_chr12	9177500	9182500
IR64	IR64_boundary_1661	IR64_chr12	9832500	9837500
IR64	IR64_boundary_1662	IR64_chr12	9892500	9897500
IR64	IR64_boundary_1663	IR64_chr12	10012500	10017500
IR64	IR64_boundary_1664	IR64_chr12	10602500	10607500
IR64	IR64_boundary_1665	IR64_chr12	10717500	10722500
IR64	IR64_boundary_1666	IR64_chr12	10927500	10932500
IR64	IR64_boundary_1667	IR64_chr12	11002500	11007500
IR64	IR64_boundary_1668	IR64_chr12	12592500	12597500
IR64	IR64_boundary_1669	IR64_chr12	12667500	12672500
IR64	IR64_boundary_1670	IR64_chr12	12747500	12787500
IR64	IR64_boundary_1671	IR64_chr12	13022500	13027500
IR64	IR64_boundary_1672	IR64_chr12	13177500	13182500

IR64	IR64_boundary_1673	IR64_chr12	14275000	14280000
IR64	IR64_boundary_1674	IR64_chr12	14457500	14462500
IR64	IR64_boundary_1675	IR64_chr12	14612500	14617500
IR64	IR64_boundary_1676	IR64_chr12	14697500	14702500
IR64	IR64_boundary_1677	IR64_chr12	14980000	14985000
IR64	IR64_boundary_1678	IR64_chr12	15147500	15152500
IR64	IR64_boundary_1679	IR64_chr12	16047500	16052500
IR64	IR64_boundary_1680	IR64_chr12	16107500	16112500
IR64	IR64_boundary_1681	IR64_chr12	16395000	16400000
IR64	IR64_boundary_1682	IR64_chr12	16467500	16472500
IR64	IR64_boundary_1683	IR64_chr12	16642500	16647500
IR64	IR64_boundary_1684	IR64_chr12	16747500	16752500
IR64	IR64_boundary_1685	IR64_chr12	16792500	16797500
IR64	IR64_boundary_1686	IR64_chr12	16867500	16872500
IR64	IR64_boundary_1687	IR64_chr12	17272500	17277500
IR64	IR64_boundary_1688	IR64_chr12	17327500	17332500
IR64	IR64_boundary_1689	IR64_chr12	18087500	18092500
IR64	IR64_boundary_1690	IR64_chr12	18147500	18187500
IR64	IR64_boundary_1691	IR64_chr12	18287500	18292500
IR64	IR64_boundary_1692	IR64_chr12	18412500	18417500
IR64	IR64_boundary_1693	IR64_chr12	18457500	18462500
IR64	IR64_boundary_1694	IR64_chr12	18552500	18557500
IR64	IR64_boundary_1695	IR64_chr12	18702500	18707500
IR64	IR64_boundary_1696	IR64_chr12	18882500	18887500
IR64	IR64_boundary_1697	IR64_chr12	20282500	20287500
IR64	IR64_boundary_1698	IR64_chr12	20392500	20397500
IR64	IR64_boundary_1699	IR64_chr12	21402500	21407500
IR64	IR64_boundary_1700	IR64_chr12	21452500	21527500
IR64	IR64_boundary_1701	IR64_chr12	21682500	21752500
IR64	IR64_boundary_1702	IR64_chr12	22817500	22852500
IR64	IR64_boundary_1703	IR64_chr12	23867500	23872500
IR64	IR64_boundary_1704	IR64_chr12	23937500	23982500

IR64	IR64_boundary_1705	IR64_chr12	24107500	24112500
IR64	IR64_boundary_1706	IR64_chr12	24182500	24187500
IR64	IR64_boundary_1707	IR64_chr12	24252500	24257500
IR64	IR64_boundary_1708	IR64_chr12	24327500	24332500
IR64	IR64_boundary_1709	IR64_chr12	24467500	24472500
IR64	IR64_boundary_1710	IR64_chr12	24887500	24892500
IR64	IR64_boundary_1711	IR64_chr12	24942500	24947500
O. meridionalis	meridionalis_boundary_0	meridionalis_chr01	522500	527500
O. meridionalis	meridionalis_boundary_1	meridionalis_chr01	572500	577500
O. meridionalis	meridionalis_boundary_2	meridionalis_chr01	652500	697500
O. meridionalis	meridionalis_boundary_3	meridionalis_chr01	1167500	1172500
O. meridionalis	meridionalis_boundary_4	meridionalis_chr01	1282500	1322500
O. meridionalis	meridionalis_boundary_5	meridionalis_chr01	1362500	1367500
O. meridionalis	meridionalis_boundary_6	meridionalis_chr01	1812500	1817500
O. meridionalis	meridionalis_boundary_7	meridionalis_chr01	1877500	1882500
O. meridionalis	meridionalis_boundary_8	meridionalis_chr01	1937500	1942500
O. meridionalis	meridionalis_boundary_9	meridionalis_chr01	2002500	2037500
O. meridionalis	meridionalis_boundary_10	meridionalis_chr01	2082500	2087500
O. meridionalis	meridionalis_boundary_11	meridionalis_chr01	2132500	2137500
O. meridionalis	meridionalis_boundary_12	meridionalis_chr01	2292500	2297500
O. meridionalis	meridionalis_boundary_13	meridionalis_chr01	2347500	2352500
O. meridionalis	meridionalis_boundary_14	meridionalis_chr01	2392500	2397500
O. meridionalis	meridionalis_boundary_15	meridionalis_chr01	3842500	3847500
O. meridionalis	meridionalis_boundary_16	meridionalis_chr01	3897500	3902500
O. meridionalis	meridionalis_boundary_17	meridionalis_chr01	4072500	4077500
O. meridionalis	meridionalis_boundary_18	meridionalis_chr01	4152500	4157500
O. meridionalis	meridionalis_boundary_19	meridionalis_chr01	4292500	4297500
O. meridionalis	meridionalis_boundary_20	meridionalis_chr01	4362500	4367500
O. meridionalis	meridionalis_boundary_21	meridionalis_chr01	4437500	4472500
O. meridionalis	meridionalis_boundary_22	meridionalis_chr01	4737500	4772500
O. meridionalis	meridionalis_boundary_23	meridionalis_chr01	4867500	4912500
O. meridionalis	meridionalis_boundary_24	meridionalis_chr01	5052500	5057500

O. meridionalis	meridionalis_boundary_25	meridionalis_chr01	5112500	5117500
O. meridionalis	meridionalis_boundary_26	meridionalis_chr01	5182500	5187500
O. meridionalis	meridionalis_boundary_27	meridionalis_chr01	5802500	5807500
O. meridionalis	meridionalis_boundary_28	meridionalis_chr01	5892500	5897500
O. meridionalis	meridionalis_boundary_29	meridionalis_chr01	6037500	6042500
O. meridionalis	meridionalis_boundary_30	meridionalis_chr01	6447500	6452500
O. meridionalis	meridionalis_boundary_31	meridionalis_chr01	6502500	6507500
O. meridionalis	meridionalis_boundary_32	meridionalis_chr01	6577500	6582500
O. meridionalis	meridionalis_boundary_33	meridionalis_chr01	6672500	6677500
O. meridionalis	meridionalis_boundary_34	meridionalis_chr01	6962500	6967500
O. meridionalis	meridionalis_boundary_35	meridionalis_chr01	7057500	7062500
O. meridionalis	meridionalis_boundary_36	meridionalis_chr01	7352500	7397500
O. meridionalis	meridionalis_boundary_37	meridionalis_chr01	7452500	7457500
O. meridionalis	meridionalis_boundary_38	meridionalis_chr01	7562500	7567500
O. meridionalis	meridionalis_boundary_39	meridionalis_chr01	7642500	7647500
O. meridionalis	meridionalis_boundary_40	meridionalis_chr01	7722500	7727500
O. meridionalis	meridionalis_boundary_41	meridionalis_chr01	7827500	7832500
O. meridionalis	meridionalis_boundary_42	meridionalis_chr01	8327500	8442500
O. meridionalis	meridionalis_boundary_43	meridionalis_chr01	8482500	8487500
O. meridionalis	meridionalis_boundary_44	meridionalis_chr01	8692500	8697500
O. meridionalis	meridionalis_boundary_45	meridionalis_chr01	8817500	8822500
O. meridionalis	meridionalis_boundary_46	meridionalis_chr01	8927500	8932500
O. meridionalis	meridionalis_boundary_47	meridionalis_chr01	8997500	9002500
O. meridionalis	meridionalis_boundary_48	meridionalis_chr01	9052500	9057500
O. meridionalis	meridionalis_boundary_49	meridionalis_chr01	9172500	9177500
O. meridionalis	meridionalis_boundary_50	meridionalis_chr01	9237500	9242500
O. meridionalis	meridionalis_boundary_51	meridionalis_chr01	9937500	9972500
O. meridionalis	meridionalis_boundary_52	meridionalis_chr01	10277500	10282500
O. meridionalis	meridionalis_boundary_53	meridionalis_chr01	10327500	10332500
O. meridionalis	meridionalis_boundary_54	meridionalis_chr01	10392500	10397500
O. meridionalis	meridionalis_boundary_55	meridionalis_chr01	10487500	10492500
O. meridionalis	meridionalis_boundary_56	meridionalis_chr01	10652500	10657500

O. meridionalis	meridionalis_boundary_57	meridionalis_chr01	10727500	10732500
O. meridionalis	meridionalis_boundary_58	meridionalis_chr01	11197500	11242500
O. meridionalis	meridionalis_boundary_59	meridionalis_chr01	13317500	13357500
O. meridionalis	meridionalis_boundary_60	meridionalis_chr01	13492500	13497500
O. meridionalis	meridionalis_boundary_61	meridionalis_chr01	15302500	15307500
O. meridionalis	meridionalis_boundary_62	meridionalis_chr01	15357500	15362500
O. meridionalis	meridionalis_boundary_63	meridionalis_chr01	15407500	15412500
O. meridionalis	meridionalis_boundary_64	meridionalis_chr01	15582500	15587500
O. meridionalis	meridionalis_boundary_65	meridionalis_chr01	16827500	16832500
O. meridionalis	meridionalis_boundary_66	meridionalis_chr01	16977500	16982500
O. meridionalis	meridionalis_boundary_67	meridionalis_chr01	17212500	17217500
O. meridionalis	meridionalis_boundary_68	meridionalis_chr01	17267500	17272500
O. meridionalis	meridionalis_boundary_69	meridionalis_chr01	17522500	17527500
O. meridionalis	meridionalis_boundary_70	meridionalis_chr01	17592500	17597500
O. meridionalis	meridionalis_boundary_71	meridionalis_chr01	17657500	17662500
O. meridionalis	meridionalis_boundary_72	meridionalis_chr01	17767500	17772500
O. meridionalis	meridionalis_boundary_73	meridionalis_chr01	17937500	17942500
O. meridionalis	meridionalis_boundary_74	meridionalis_chr01	17982500	17987500
O. meridionalis	meridionalis_boundary_75	meridionalis_chr01	18092500	18097500
O. meridionalis	meridionalis_boundary_76	meridionalis_chr01	18487500	18517500
O. meridionalis	meridionalis_boundary_77	meridionalis_chr01	18702500	18707500
O. meridionalis	meridionalis_boundary_78	meridionalis_chr01	19597500	19602500
O. meridionalis	meridionalis_boundary_79	meridionalis_chr01	19687500	19725000
O. meridionalis	meridionalis_boundary_80	meridionalis_chr01	19782500	19787500
O. meridionalis	meridionalis_boundary_81	meridionalis_chr01	20737500	20742500
O. meridionalis	meridionalis_boundary_82	meridionalis_chr01	20872500	20877500
O. meridionalis	meridionalis_boundary_83	meridionalis_chr01	21902500	21907500
O. meridionalis	meridionalis_boundary_84	meridionalis_chr01	22007500	22012500
O. meridionalis	meridionalis_boundary_85	meridionalis_chr01	22107500	22112500
O. meridionalis	meridionalis_boundary_86	meridionalis_chr01	22202500	22207500
O. meridionalis	meridionalis_boundary_87	meridionalis_chr01	22287500	22292500
O. meridionalis	meridionalis_boundary_88	meridionalis_chr01	22347500	22352500

O. meridionalis	meridionalis_boundary_89	meridionalis_chr01	22762500	22767500
O. meridionalis	meridionalis_boundary_90	meridionalis_chr01	22817500	22822500
O. meridionalis	meridionalis_boundary_91	meridionalis_chr01	23382500	23417500
O. meridionalis	meridionalis_boundary_92	meridionalis_chr01	24012500	24017500
O. meridionalis	meridionalis_boundary_93	meridionalis_chr01	24177500	24182500
O. meridionalis	meridionalis_boundary_94	meridionalis_chr01	24222500	24227500
O. meridionalis	meridionalis_boundary_95	meridionalis_chr01	24272500	24277500
O. meridionalis	meridionalis_boundary_96	meridionalis_chr01	24722500	24727500
O. meridionalis	meridionalis_boundary_97	meridionalis_chr01	24772500	24777500
O. meridionalis	meridionalis_boundary_98	meridionalis_chr01	25117500	25122500
O. meridionalis	meridionalis_boundary_99	meridionalis_chr01	25282500	25287500
O. meridionalis	meridionalis_boundary_100	meridionalis_chr01	25357500	25362500
O. meridionalis	meridionalis_boundary_101	meridionalis_chr01	25417500	25522500
O. meridionalis	meridionalis_boundary_102	meridionalis_chr01	25567500	25597500
O. meridionalis	meridionalis_boundary_103	meridionalis_chr01	26177500	26182500
O. meridionalis	meridionalis_boundary_104	meridionalis_chr01	26247500	26252500
O. meridionalis	meridionalis_boundary_105	meridionalis_chr01	26782500	26787500
O. meridionalis	meridionalis_boundary_106	meridionalis_chr01	26832500	26872500
O. meridionalis	meridionalis_boundary_107	meridionalis_chr01	27002500	27007500
O. meridionalis	meridionalis_boundary_108	meridionalis_chr01	27047500	27052500
O. meridionalis	meridionalis_boundary_109	meridionalis_chr01	27427500	27472500
O. meridionalis	meridionalis_boundary_110	meridionalis_chr01	27672500	27677500
O. meridionalis	meridionalis_boundary_111	meridionalis_chr01	27747500	27752500
O. meridionalis	meridionalis_boundary_112	meridionalis_chr01	28297500	28302500
O. meridionalis	meridionalis_boundary_113	meridionalis_chr01	28442500	28447500
O. meridionalis	meridionalis_boundary_114	meridionalis_chr01	29327500	29362500
O. meridionalis	meridionalis_boundary_115	meridionalis_chr01	29977500	29982500
O. meridionalis	meridionalis_boundary_116	meridionalis_chr01	30022500	30027500
O. meridionalis	meridionalis_boundary_117	meridionalis_chr01	30087500	30092500
O. meridionalis	meridionalis_boundary_118	meridionalis_chr01	30152500	30157500
O. meridionalis	meridionalis_boundary_119	meridionalis_chr01	30607500	30612500
O. meridionalis	meridionalis_boundary_120	meridionalis_chr01	30682500	30687500

O. meridionalis	meridionalis_boundary_121	meridionalis_chr01	30882500	30887500
O. meridionalis	meridionalis_boundary_122	meridionalis_chr01	30962500	31037500
O. meridionalis	meridionalis_boundary_123	meridionalis_chr01	31207500	31212500
O. meridionalis	meridionalis_boundary_124	meridionalis_chr01	31267500	31272500
O. meridionalis	meridionalis_boundary_125	meridionalis_chr01	31312500	31357500
O. meridionalis	meridionalis_boundary_126	meridionalis_chr01	31462500	31467500
O. meridionalis	meridionalis_boundary_127	meridionalis_chr01	31977500	31982500
O. meridionalis	meridionalis_boundary_128	meridionalis_chr01	32032500	32037500
O. meridionalis	meridionalis_boundary_129	meridionalis_chr01	32107500	32112500
O. meridionalis	meridionalis_boundary_130	meridionalis_chr01	33367500	33372500
O. meridionalis	meridionalis_boundary_131	meridionalis_chr01	33432500	33477500
O. meridionalis	meridionalis_boundary_132	meridionalis_chr01	33577500	33582500
O. meridionalis	meridionalis_boundary_133	meridionalis_chr01	33717500	33722500
O. meridionalis	meridionalis_boundary_134	meridionalis_chr01	33792500	33797500
O. meridionalis	meridionalis_boundary_135	meridionalis_chr01	33847500	33852500
O. meridionalis	meridionalis_boundary_136	meridionalis_chr01	33937500	33972500
O. meridionalis	meridionalis_boundary_137	meridionalis_chr01	34037500	34042500
O. meridionalis	meridionalis_boundary_138	meridionalis_chr01	34137500	34142500
O. meridionalis	meridionalis_boundary_139	meridionalis_chr01	34292500	34297500
O. meridionalis	meridionalis_boundary_140	meridionalis_chr01	35002500	35007500
O. meridionalis	meridionalis_boundary_141	meridionalis_chr01	35087500	35127500
O. meridionalis	meridionalis_boundary_142	meridionalis_chr01	35387500	35392500
O. meridionalis	meridionalis_boundary_143	meridionalis_chr01	35477500	35547500
O. meridionalis	meridionalis_boundary_144	meridionalis_chr01	35602500	35607500
O. meridionalis	meridionalis_boundary_145	meridionalis_chr01	35657500	35662500
O. meridionalis	meridionalis_boundary_146	meridionalis_chr01	35702500	35707500
O. meridionalis	meridionalis_boundary_147	meridionalis_chr01	36112500	36117500
O. meridionalis	meridionalis_boundary_148	meridionalis_chr01	36167500	36172500
O. meridionalis	meridionalis_boundary_149	meridionalis_chr01	36242500	36247500
O. meridionalis	meridionalis_boundary_150	meridionalis_chr01	36337500	36342500
O. meridionalis	meridionalis_boundary_151	meridionalis_chr01	36675000	36680000
O. meridionalis	meridionalis_boundary_152	meridionalis_chr01	36747500	36752500

O. meridionalis	meridionalis_boundary_153	meridionalis_chr01	36887500	36892500
O. meridionalis	meridionalis_boundary_154	meridionalis_chr01	36967500	36972500
O. meridionalis	meridionalis_boundary_155	meridionalis_chr01	37032500	37037500
O. meridionalis	meridionalis_boundary_156	meridionalis_chr01	37092500	37097500
O. meridionalis	meridionalis_boundary_157	meridionalis_chr01	37197500	37202500
O. meridionalis	meridionalis_boundary_158	meridionalis_chr01	37252500	37257500
O. meridionalis	meridionalis_boundary_159	meridionalis_chr01	37452500	37457500
O. meridionalis	meridionalis_boundary_160	meridionalis_chr01	37547500	37552500
O. meridionalis	meridionalis_boundary_161	meridionalis_chr01	37667500	37672500
O. meridionalis	meridionalis_boundary_162	meridionalis_chr01	37717500	37722500
O. meridionalis	meridionalis_boundary_163	meridionalis_chr01	37817500	37822500
O. meridionalis	meridionalis_boundary_164	meridionalis_chr01	37872500	37877500
O. meridionalis	meridionalis_boundary_165	meridionalis_chr01	37955000	37960000
O. meridionalis	meridionalis_boundary_166	meridionalis_chr01	39447500	39452500
O. meridionalis	meridionalis_boundary_167	meridionalis_chr01	39517500	39582500
O. meridionalis	meridionalis_boundary_168	meridionalis_chr01	39652500	39657500
O. meridionalis	meridionalis_boundary_169	meridionalis_chr01	39722500	39727500
O. meridionalis	meridionalis_boundary_170	meridionalis_chr01	39802500	39807500
O. meridionalis	meridionalis_boundary_171	meridionalis_chr01	39892500	39897500
O. meridionalis	meridionalis_boundary_172	meridionalis_chr01	40017500	40052500
O. meridionalis	meridionalis_boundary_173	meridionalis_chr01	40142500	40182500
O. meridionalis	meridionalis_boundary_174	meridionalis_chr01	40227500	40232500
O. meridionalis	meridionalis_boundary_175	meridionalis_chr01	40337500	40342500
O. meridionalis	meridionalis_boundary_176	meridionalis_chr01	40417500	40422500
O. meridionalis	meridionalis_boundary_177	meridionalis_chr01	40552500	40557500
O. meridionalis	meridionalis_boundary_178	meridionalis_chr01	40602500	40607500
O. meridionalis	meridionalis_boundary_179	meridionalis_chr01	41527500	41532500
O. meridionalis	meridionalis_boundary_180	meridionalis_chr01	41587500	41592500
O. meridionalis	meridionalis_boundary_181	meridionalis_chr01	42067500	42072500
O. meridionalis	meridionalis_boundary_182	meridionalis_chr01	42137500	42142500
O. meridionalis	meridionalis_boundary_183	meridionalis_chr01	42387500	42392500
O. meridionalis	meridionalis_boundary_184	meridionalis_chr01	42492500	42497500

O. meridionalis	meridionalis_boundary_185	meridionalis_chr02	342500	347500
O. meridionalis	meridionalis_boundary_186	meridionalis_chr02	427500	467500
O. meridionalis	meridionalis_boundary_187	meridionalis_chr02	612500	652500
O. meridionalis	meridionalis_boundary_188	meridionalis_chr02	712500	717500
O. meridionalis	meridionalis_boundary_189	meridionalis_chr02	807500	812500
O. meridionalis	meridionalis_boundary_190	meridionalis_chr02	902500	907500
O. meridionalis	meridionalis_boundary_191	meridionalis_chr02	1622500	1627500
O. meridionalis	meridionalis_boundary_192	meridionalis_chr02	1687500	1727500
O. meridionalis	meridionalis_boundary_193	meridionalis_chr02	2852500	2887500
O. meridionalis	meridionalis_boundary_194	meridionalis_chr02	3067500	3072500
O. meridionalis	meridionalis_boundary_195	meridionalis_chr02	3207500	3212500
O. meridionalis	meridionalis_boundary_196	meridionalis_chr02	3277500	3322500
O. meridionalis	meridionalis_boundary_197	meridionalis_chr02	3732500	3737500
O. meridionalis	meridionalis_boundary_198	meridionalis_chr02	3777500	3782500
O. meridionalis	meridionalis_boundary_199	meridionalis_chr02	3882500	3887500
O. meridionalis	meridionalis_boundary_200	meridionalis_chr02	4067500	4112500
O. meridionalis	meridionalis_boundary_201	meridionalis_chr02	4197500	4202500
O. meridionalis	meridionalis_boundary_202	meridionalis_chr02	4242500	4247500
O. meridionalis	meridionalis_boundary_203	meridionalis_chr02	4412500	4417500
O. meridionalis	meridionalis_boundary_204	meridionalis_chr02	4512500	4517500
O. meridionalis	meridionalis_boundary_205	meridionalis_chr02	4777500	4782500
O. meridionalis	meridionalis_boundary_206	meridionalis_chr02	4827500	4832500
O. meridionalis	meridionalis_boundary_207	meridionalis_chr02	5047500	5052500
O. meridionalis	meridionalis_boundary_208	meridionalis_chr02	5227500	5232500
O. meridionalis	meridionalis_boundary_209	meridionalis_chr02	5492500	5497500
O. meridionalis	meridionalis_boundary_210	meridionalis_chr02	5637500	5642500
O. meridionalis	meridionalis_boundary_211	meridionalis_chr02	5762500	5767500
O. meridionalis	meridionalis_boundary_212	meridionalis_chr02	5817500	5822500
O. meridionalis	meridionalis_boundary_213	meridionalis_chr02	5892500	5897500
O. meridionalis	meridionalis_boundary_214	meridionalis_chr02	6187500	6192500
O. meridionalis	meridionalis_boundary_215	meridionalis_chr02	6287500	6292500
O. meridionalis	meridionalis_boundary_216	meridionalis_chr02	6712500	6717500

O. meridionalis	meridionalis_boundary_217	meridionalis_chr02	6812500	6817500
O. meridionalis	meridionalis_boundary_218	meridionalis_chr02	6857500	6862500
O. meridionalis	meridionalis_boundary_219	meridionalis_chr02	6952500	6957500
O. meridionalis	meridionalis_boundary_220	meridionalis_chr02	6997500	7002500
O. meridionalis	meridionalis_boundary_221	meridionalis_chr02	7197500	7242500
O. meridionalis	meridionalis_boundary_222	meridionalis_chr02	7342500	7377500
O. meridionalis	meridionalis_boundary_223	meridionalis_chr02	7435000	7440000
O. meridionalis	meridionalis_boundary_224	meridionalis_chr02	7572500	7577500
O. meridionalis	meridionalis_boundary_225	meridionalis_chr02	7617500	7622500
O. meridionalis	meridionalis_boundary_226	meridionalis_chr02	7717500	7722500
O. meridionalis	meridionalis_boundary_227	meridionalis_chr02	7787500	7792500
O. meridionalis	meridionalis_boundary_228	meridionalis_chr02	7867500	7872500
O. meridionalis	meridionalis_boundary_229	meridionalis_chr02	8147500	8152500
O. meridionalis	meridionalis_boundary_230	meridionalis_chr02	8197500	8202500
O. meridionalis	meridionalis_boundary_231	meridionalis_chr02	8472500	8477500
O. meridionalis	meridionalis_boundary_232	meridionalis_chr02	8542500	8547500
O. meridionalis	meridionalis_boundary_233	meridionalis_chr02	8597500	8602500
O. meridionalis	meridionalis_boundary_234	meridionalis_chr02	9252500	9317500
O. meridionalis	meridionalis_boundary_235	meridionalis_chr02	9392500	9397500
O. meridionalis	meridionalis_boundary_236	meridionalis_chr02	9497500	9527500
O. meridionalis	meridionalis_boundary_237	meridionalis_chr02	9757500	9787500
O. meridionalis	meridionalis_boundary_238	meridionalis_chr02	10392500	10397500
O. meridionalis	meridionalis_boundary_239	meridionalis_chr02	10472500	10477500
O. meridionalis	meridionalis_boundary_240	meridionalis_chr02	10592500	10597500
O. meridionalis	meridionalis_boundary_241	meridionalis_chr02	10652500	10657500
O. meridionalis	meridionalis_boundary_242	meridionalis_chr02	11462500	11467500
O. meridionalis	meridionalis_boundary_243	meridionalis_chr02	11537500	11542500
O. meridionalis	meridionalis_boundary_244	meridionalis_chr02	11657500	11662500
O. meridionalis	meridionalis_boundary_245	meridionalis_chr02	11887500	11927500
O. meridionalis	meridionalis_boundary_246	meridionalis_chr02	12017500	12022500
O. meridionalis	meridionalis_boundary_247	meridionalis_chr02	12112500	12117500
O. meridionalis	meridionalis_boundary_248	meridionalis_chr02	13942500	13947500

O. meridionalis	meridionalis_boundary_249	meridionalis_chr02	14022500	14027500
O. meridionalis	meridionalis_boundary_250	meridionalis_chr02	18202500	18207500
O. meridionalis	meridionalis_boundary_251	meridionalis_chr02	18272500	18277500
O. meridionalis	meridionalis_boundary_252	meridionalis_chr02	18392500	18397500
O. meridionalis	meridionalis_boundary_253	meridionalis_chr02	18972500	19047500
O. meridionalis	meridionalis_boundary_254	meridionalis_chr02	19107500	19112500
O. meridionalis	meridionalis_boundary_255	meridionalis_chr02	19687500	19692500
O. meridionalis	meridionalis_boundary_256	meridionalis_chr02	19782500	19787500
O. meridionalis	meridionalis_boundary_257	meridionalis_chr02	19837500	19842500
O. meridionalis	meridionalis_boundary_258	meridionalis_chr02	20047500	20092500
O. meridionalis	meridionalis_boundary_259	meridionalis_chr02	20157500	20162500
O. meridionalis	meridionalis_boundary_260	meridionalis_chr02	20222500	20227500
O. meridionalis	meridionalis_boundary_261	meridionalis_chr02	20522500	20527500
O. meridionalis	meridionalis_boundary_262	meridionalis_chr02	20572500	20577500
O. meridionalis	meridionalis_boundary_263	meridionalis_chr02	20757500	20762500
O. meridionalis	meridionalis_boundary_264	meridionalis_chr02	20837500	20842500
O. meridionalis	meridionalis_boundary_265	meridionalis_chr02	20902500	20907500
O. meridionalis	meridionalis_boundary_266	meridionalis_chr02	20987500	20992500
O. meridionalis	meridionalis_boundary_267	meridionalis_chr02	21092500	21097500
O. meridionalis	meridionalis_boundary_268	meridionalis_chr02	21182500	21187500
O. meridionalis	meridionalis_boundary_269	meridionalis_chr02	21272500	21277500
O. meridionalis	meridionalis_boundary_270	meridionalis_chr02	21352500	21387500
O. meridionalis	meridionalis_boundary_271	meridionalis_chr02	21912500	21917500
O. meridionalis	meridionalis_boundary_272	meridionalis_chr02	21957500	22002500
O. meridionalis	meridionalis_boundary_273	meridionalis_chr02	22132500	22137500
O. meridionalis	meridionalis_boundary_274	meridionalis_chr02	22202500	22207500
O. meridionalis	meridionalis_boundary_275	meridionalis_chr02	22742500	22747500
O. meridionalis	meridionalis_boundary_276	meridionalis_chr02	22817500	22822500
O. meridionalis	meridionalis_boundary_277	meridionalis_chr02	22875000	22880000
O. meridionalis	meridionalis_boundary_278	meridionalis_chr02	22957500	22962500
O. meridionalis	meridionalis_boundary_279	meridionalis_chr02	23187500	23192500
O. meridionalis	meridionalis_boundary_280	meridionalis_chr02	23262500	23267500

O. meridionalis	meridionalis_boundary_281	meridionalis_chr02	23347500	23352500
O. meridionalis	meridionalis_boundary_282	meridionalis_chr02	23402500	23407500
O. meridionalis	meridionalis_boundary_283	meridionalis_chr02	23517500	23522500
O. meridionalis	meridionalis_boundary_284	meridionalis_chr02	24742500	24822500
O. meridionalis	meridionalis_boundary_285	meridionalis_chr02	24877500	24882500
O. meridionalis	meridionalis_boundary_286	meridionalis_chr02	25092500	25097500
O. meridionalis	meridionalis_boundary_287	meridionalis_chr02	25142500	25147500
O. meridionalis	meridionalis_boundary_288	meridionalis_chr02	25227500	25272500
O. meridionalis	meridionalis_boundary_289	meridionalis_chr02	25357500	25402500
O. meridionalis	meridionalis_boundary_290	meridionalis_chr02	25477500	25482500
O. meridionalis	meridionalis_boundary_291	meridionalis_chr02	25572500	25577500
O. meridionalis	meridionalis_boundary_292	meridionalis_chr02	25652500	25657500
O. meridionalis	meridionalis_boundary_293	meridionalis_chr02	25857500	25862500
O. meridionalis	meridionalis_boundary_294	meridionalis_chr02	25917500	25922500
O. meridionalis	meridionalis_boundary_295	meridionalis_chr02	25982500	25987500
O. meridionalis	meridionalis_boundary_296	meridionalis_chr02	26082500	26087500
O. meridionalis	meridionalis_boundary_297	meridionalis_chr02	26222500	26227500
O. meridionalis	meridionalis_boundary_298	meridionalis_chr02	26292500	26297500
O. meridionalis	meridionalis_boundary_299	meridionalis_chr02	26927500	26972500
O. meridionalis	meridionalis_boundary_300	meridionalis_chr02	27062500	27067500
O. meridionalis	meridionalis_boundary_301	meridionalis_chr02	27117500	27122500
O. meridionalis	meridionalis_boundary_302	meridionalis_chr02	27197500	27202500
O. meridionalis	meridionalis_boundary_303	meridionalis_chr02	27357500	27362500
O. meridionalis	meridionalis_boundary_304	meridionalis_chr02	27407500	27412500
O. meridionalis	meridionalis_boundary_305	meridionalis_chr02	27782500	27787500
O. meridionalis	meridionalis_boundary_306	meridionalis_chr02	27852500	27857500
O. meridionalis	meridionalis_boundary_307	meridionalis_chr02	27937500	27942500
O. meridionalis	meridionalis_boundary_308	meridionalis_chr02	28192500	28197500
O. meridionalis	meridionalis_boundary_309	meridionalis_chr02	28267500	28272500
O. meridionalis	meridionalis_boundary_310	meridionalis_chr02	28322500	28327500
O. meridionalis	meridionalis_boundary_311	meridionalis_chr02	28412500	28417500
O. meridionalis	meridionalis_boundary_312	meridionalis_chr02	28477500	28482500

O. meridionalis	meridionalis_boundary_313	meridionalis_chr02	28707500	28712500
O. meridionalis	meridionalis_boundary_314	meridionalis_chr02	28817500	28822500
O. meridionalis	meridionalis_boundary_315	meridionalis_chr02	28867500	28872500
O. meridionalis	meridionalis_boundary_316	meridionalis_chr02	29185000	29232500
O. meridionalis	meridionalis_boundary_317	meridionalis_chr02	29592500	29597500
O. meridionalis	meridionalis_boundary_318	meridionalis_chr02	29647500	29652500
O. meridionalis	meridionalis_boundary_319	meridionalis_chr02	29712500	29717500
O. meridionalis	meridionalis_boundary_320	meridionalis_chr02	29827500	29902500
O. meridionalis	meridionalis_boundary_321	meridionalis_chr02	29997500	30002500
O. meridionalis	meridionalis_boundary_322	meridionalis_chr02	30082500	30087500
O. meridionalis	meridionalis_boundary_323	meridionalis_chr02	30207500	30212500
O. meridionalis	meridionalis_boundary_324	meridionalis_chr02	30752500	30757500
O. meridionalis	meridionalis_boundary_325	meridionalis_chr02	30802500	30807500
O. meridionalis	meridionalis_boundary_326	meridionalis_chr02	30872500	30877500
O. meridionalis	meridionalis_boundary_327	meridionalis_chr02	30942500	30947500
O. meridionalis	meridionalis_boundary_328	meridionalis_chr02	30992500	30997500
O. meridionalis	meridionalis_boundary_329	meridionalis_chr02	31057500	31062500
O. meridionalis	meridionalis_boundary_330	meridionalis_chr02	31257500	31262500
O. meridionalis	meridionalis_boundary_331	meridionalis_chr02	31302500	31307500
O. meridionalis	meridionalis_boundary_332	meridionalis_chr02	31457500	31462500
O. meridionalis	meridionalis_boundary_333	meridionalis_chr02	31532500	31537500
O. meridionalis	meridionalis_boundary_334	meridionalis_chr02	31692500	31697500
O. meridionalis	meridionalis_boundary_335	meridionalis_chr02	31747500	31752500
O. meridionalis	meridionalis_boundary_336	meridionalis_chr02	32242500	32247500
O. meridionalis	meridionalis_boundary_337	meridionalis_chr02	32342500	32347500
O. meridionalis	meridionalis_boundary_338	meridionalis_chr02	32512500	32517500
O. meridionalis	meridionalis_boundary_339	meridionalis_chr02	32557500	32562500
O. meridionalis	meridionalis_boundary_340	meridionalis_chr02	32617500	32622500
O. meridionalis	meridionalis_boundary_341	meridionalis_chr02	33007500	33012500
O. meridionalis	meridionalis_boundary_342	meridionalis_chr02	33072500	33077500
O. meridionalis	meridionalis_boundary_343	meridionalis_chr02	33132500	33137500
O. meridionalis	meridionalis_boundary_344	meridionalis_chr02	33202500	33207500

O. meridionalis	meridionalis_boundary_345	meridionalis_chr02	33452500	33457500
O. meridionalis	meridionalis_boundary_346	meridionalis_chr02	33532500	33537500
O. meridionalis	meridionalis_boundary_347	meridionalis_chr02	33592500	33597500
O. meridionalis	meridionalis_boundary_348	meridionalis_chr02	33887500	33927500
O. meridionalis	meridionalis_boundary_349	meridionalis_chr02	34017500	34022500
O. meridionalis	meridionalis_boundary_350	meridionalis_chr02	34117500	34152500
O. meridionalis	meridionalis_boundary_351	meridionalis_chr02	34357500	34362500
O. meridionalis	meridionalis_boundary_352	meridionalis_chr02	34422500	34427500
O. meridionalis	meridionalis_boundary_353	meridionalis_chr02	34542500	34547500
O. meridionalis	meridionalis_boundary_354	meridionalis_chr02	34642500	34687500
O. meridionalis	meridionalis_boundary_355	meridionalis_chr02	34792500	34797500
O. meridionalis	meridionalis_boundary_356	meridionalis_chr02	34842500	34847500
O. meridionalis	meridionalis_boundary_357	meridionalis_chr02	34902500	34907500
O. meridionalis	meridionalis_boundary_358	meridionalis_chr02	34952500	34957500
O. meridionalis	meridionalis_boundary_359	meridionalis_chr02	35002500	35007500
O. meridionalis	meridionalis_boundary_360	meridionalis_chr02	35447500	35452500
O. meridionalis	meridionalis_boundary_361	meridionalis_chr02	35517500	35587500
O. meridionalis	meridionalis_boundary_362	meridionalis_chr02	35640000	35645000
O. meridionalis	meridionalis_boundary_363	meridionalis_chr02	35687500	35692500
O. meridionalis	meridionalis_boundary_364	meridionalis_chr02	35782500	35787500
O. meridionalis	meridionalis_boundary_365	meridionalis_chr02	35882500	35922500
O. meridionalis	meridionalis_boundary_366	meridionalis_chr02	36092500	36097500
O. meridionalis	meridionalis_boundary_367	meridionalis_chr02	36182500	36187500
O. meridionalis	meridionalis_boundary_368	meridionalis_chr02	36392500	36397500
O. meridionalis	meridionalis_boundary_369	meridionalis_chr02	36472500	36477500
O. meridionalis	meridionalis_boundary_370	meridionalis_chr02	36542500	36547500
O. meridionalis	meridionalis_boundary_371	meridionalis_chr02	36772500	36777500
O. meridionalis	meridionalis_boundary_372	meridionalis_chr02	36827500	36832500
O. meridionalis	meridionalis_boundary_373	meridionalis_chr02	36882500	36887500
O. meridionalis	meridionalis_boundary_374	meridionalis_chr02	36957500	36962500
O. meridionalis	meridionalis_boundary_375	meridionalis_chr03	547500	552500
O. meridionalis	meridionalis_boundary_376	meridionalis_chr03	607500	612500

O. meridionalis	meridionalis_boundary_377	meridionalis_chr03	677500	682500
O. meridionalis	meridionalis_boundary_378	meridionalis_chr03	727500	732500
O. meridionalis	meridionalis_boundary_379	meridionalis_chr03	887500	922500
O. meridionalis	meridionalis_boundary_380	meridionalis_chr03	992500	997500
O. meridionalis	meridionalis_boundary_381	meridionalis_chr03	1472500	1512500
O. meridionalis	meridionalis_boundary_382	meridionalis_chr03	1612500	1657500
O. meridionalis	meridionalis_boundary_383	meridionalis_chr03	1702500	1707500
O. meridionalis	meridionalis_boundary_384	meridionalis_chr03	2402500	2407500
O. meridionalis	meridionalis_boundary_385	meridionalis_chr03	2537500	2542500
O. meridionalis	meridionalis_boundary_386	meridionalis_chr03	2602500	2607500
O. meridionalis	meridionalis_boundary_387	meridionalis_chr03	2662500	2667500
O. meridionalis	meridionalis_boundary_388	meridionalis_chr03	3237500	3242500
O. meridionalis	meridionalis_boundary_389	meridionalis_chr03	3292500	3337500
O. meridionalis	meridionalis_boundary_390	meridionalis_chr03	3807500	3812500
O. meridionalis	meridionalis_boundary_391	meridionalis_chr03	3852500	3857500
O. meridionalis	meridionalis_boundary_392	meridionalis_chr03	3907500	3912500
O. meridionalis	meridionalis_boundary_393	meridionalis_chr03	3957500	3962500
O. meridionalis	meridionalis_boundary_394	meridionalis_chr03	4002500	4007500
O. meridionalis	meridionalis_boundary_395	meridionalis_chr03	4062500	4067500
O. meridionalis	meridionalis_boundary_396	meridionalis_chr03	4277500	4282500
O. meridionalis	meridionalis_boundary_397	meridionalis_chr03	4347500	4352500
O. meridionalis	meridionalis_boundary_398	meridionalis_chr03	4412500	4417500
O. meridionalis	meridionalis_boundary_399	meridionalis_chr03	4507500	4512500
O. meridionalis	meridionalis_boundary_400	meridionalis_chr03	4942500	4947500
O. meridionalis	meridionalis_boundary_401	meridionalis_chr03	5002500	5007500
O. meridionalis	meridionalis_boundary_402	meridionalis_chr03	6737500	6742500
O. meridionalis	meridionalis_boundary_403	meridionalis_chr03	6787500	6792500
O. meridionalis	meridionalis_boundary_404	meridionalis_chr03	7562500	7567500
O. meridionalis	meridionalis_boundary_405	meridionalis_chr03	7632500	7637500
O. meridionalis	meridionalis_boundary_406	meridionalis_chr03	7777500	7782500
O. meridionalis	meridionalis_boundary_407	meridionalis_chr03	7857500	7862500
O. meridionalis	meridionalis_boundary_408	meridionalis_chr03	8057500	8062500

O. meridionalis	meridionalis_boundary_409	meridionalis_chr03	8127500	8162500
O. meridionalis	meridionalis_boundary_410	meridionalis_chr03	8657500	8662500
O. meridionalis	meridionalis_boundary_411	meridionalis_chr03	8702500	8707500
O. meridionalis	meridionalis_boundary_412	meridionalis_chr03	9022500	9027500
O. meridionalis	meridionalis_boundary_413	meridionalis_chr03	9082500	9087500
O. meridionalis	meridionalis_boundary_414	meridionalis_chr03	9332500	9367500
O. meridionalis	meridionalis_boundary_415	meridionalis_chr03	9422500	9427500
O. meridionalis	meridionalis_boundary_416	meridionalis_chr03	9502500	9507500
O. meridionalis	meridionalis_boundary_417	meridionalis_chr03	9572500	9577500
O. meridionalis	meridionalis_boundary_418	meridionalis_chr03	9657500	9662500
O. meridionalis	meridionalis_boundary_419	meridionalis_chr03	9707500	9712500
O. meridionalis	meridionalis_boundary_420	meridionalis_chr03	9857500	9862500
O. meridionalis	meridionalis_boundary_421	meridionalis_chr03	9907500	9912500
O. meridionalis	meridionalis_boundary_422	meridionalis_chr03	10312500	10317500
O. meridionalis	meridionalis_boundary_423	meridionalis_chr03	10357500	10362500
O. meridionalis	meridionalis_boundary_424	meridionalis_chr03	10562500	10567500
O. meridionalis	meridionalis_boundary_425	meridionalis_chr03	10652500	10697500
O. meridionalis	meridionalis_boundary_426	meridionalis_chr03	10797500	10877500
O. meridionalis	meridionalis_boundary_427	meridionalis_chr03	11482500	11487500
O. meridionalis	meridionalis_boundary_428	meridionalis_chr03	11562500	11567500
O. meridionalis	meridionalis_boundary_429	meridionalis_chr03	11642500	11647500
O. meridionalis	meridionalis_boundary_430	meridionalis_chr03	11727500	11732500
O. meridionalis	meridionalis_boundary_431	meridionalis_chr03	11772500	11777500
O. meridionalis	meridionalis_boundary_432	meridionalis_chr03	11852500	11857500
O. meridionalis	meridionalis_boundary_433	meridionalis_chr03	12107500	12112500
O. meridionalis	meridionalis_boundary_434	meridionalis_chr03	12152500	12192500
O. meridionalis	meridionalis_boundary_435	meridionalis_chr03	12302500	12342500
O. meridionalis	meridionalis_boundary_436	meridionalis_chr03	12572500	12607500
O. meridionalis	meridionalis_boundary_437	meridionalis_chr03	12772500	12812500
O. meridionalis	meridionalis_boundary_438	meridionalis_chr03	12857500	12862500
O. meridionalis	meridionalis_boundary_439	meridionalis_chr03	12997500	13002500
O. meridionalis	meridionalis_boundary_440	meridionalis_chr03	13062500	13067500

O. meridionalis	meridionalis_boundary_441	meridionalis_chr03	13162500	13167500
O. meridionalis	meridionalis_boundary_442	meridionalis_chr03	13272500	13277500
O. meridionalis	meridionalis_boundary_443	meridionalis_chr03	13682500	13687500
O. meridionalis	meridionalis_boundary_444	meridionalis_chr03	13787500	13872500
O. meridionalis	meridionalis_boundary_445	meridionalis_chr03	14087500	14132500
O. meridionalis	meridionalis_boundary_446	meridionalis_chr03	14217500	14222500
O. meridionalis	meridionalis_boundary_447	meridionalis_chr03	14352500	14357500
O. meridionalis	meridionalis_boundary_448	meridionalis_chr03	14412500	14417500
O. meridionalis	meridionalis_boundary_449	meridionalis_chr03	14462500	14467500
O. meridionalis	meridionalis_boundary_450	meridionalis_chr03	15057500	15095000
O. meridionalis	meridionalis_boundary_451	meridionalis_chr03	15162500	15167500
O. meridionalis	meridionalis_boundary_452	meridionalis_chr03	23772500	23812500
O. meridionalis	meridionalis_boundary_453	meridionalis_chr03	23952500	23957500
O. meridionalis	meridionalis_boundary_454	meridionalis_chr03	24297500	24332500
O. meridionalis	meridionalis_boundary_455	meridionalis_chr03	24487500	24492500
O. meridionalis	meridionalis_boundary_456	meridionalis_chr03	24927500	24932500
O. meridionalis	meridionalis_boundary_457	meridionalis_chr03	26132500	26137500
O. meridionalis	meridionalis_boundary_458	meridionalis_chr03	26207500	26212500
O. meridionalis	meridionalis_boundary_459	meridionalis_chr03	26327500	26367500
O. meridionalis	meridionalis_boundary_460	meridionalis_chr03	27437500	27482500
O. meridionalis	meridionalis_boundary_461	meridionalis_chr03	27522500	27527500
O. meridionalis	meridionalis_boundary_462	meridionalis_chr03	27607500	27612500
O. meridionalis	meridionalis_boundary_463	meridionalis_chr03	27652500	27657500
O. meridionalis	meridionalis_boundary_464	meridionalis_chr03	28092500	28097500
O. meridionalis	meridionalis_boundary_465	meridionalis_chr03	28180000	28185000
O. meridionalis	meridionalis_boundary_466	meridionalis_chr03	28255000	28260000
O. meridionalis	meridionalis_boundary_467	meridionalis_chr03	28362500	28407500
O. meridionalis	meridionalis_boundary_468	meridionalis_chr03	28497500	28502500
O. meridionalis	meridionalis_boundary_469	meridionalis_chr03	28622500	28627500
O. meridionalis	meridionalis_boundary_470	meridionalis_chr03	28697500	28702500
O. meridionalis	meridionalis_boundary_471	meridionalis_chr03	28832500	28837500
O. meridionalis	meridionalis_boundary_472	meridionalis_chr03	28892500	28897500

O. meridionalis	meridionalis_boundary_473	meridionalis_chr03	29132500	29137500
O. meridionalis	meridionalis_boundary_474	meridionalis_chr03	29347500	29352500
O. meridionalis	meridionalis_boundary_475	meridionalis_chr03	29472500	29517500
O. meridionalis	meridionalis_boundary_476	meridionalis_chr03	29642500	29647500
O. meridionalis	meridionalis_boundary_477	meridionalis_chr03	30622500	30627500
O. meridionalis	meridionalis_boundary_478	meridionalis_chr03	30677500	30682500
O. meridionalis	meridionalis_boundary_479	meridionalis_chr03	31032500	31072500
O. meridionalis	meridionalis_boundary_480	meridionalis_chr03	31122500	31127500
O. meridionalis	meridionalis_boundary_481	meridionalis_chr03	31322500	31327500
O. meridionalis	meridionalis_boundary_482	meridionalis_chr03	31452500	31487500
O. meridionalis	meridionalis_boundary_483	meridionalis_chr03	31572500	31577500
O. meridionalis	meridionalis_boundary_484	meridionalis_chr03	31647500	31652500
O. meridionalis	meridionalis_boundary_485	meridionalis_chr03	31717500	31722500
O. meridionalis	meridionalis_boundary_486	meridionalis_chr03	31777500	31782500
O. meridionalis	meridionalis_boundary_487	meridionalis_chr03	32217500	32257500
O. meridionalis	meridionalis_boundary_488	meridionalis_chr03	32437500	32442500
O. meridionalis	meridionalis_boundary_489	meridionalis_chr03	32482500	32487500
O. meridionalis	meridionalis_boundary_490	meridionalis_chr03	33027500	33032500
O. meridionalis	meridionalis_boundary_491	meridionalis_chr03	33147500	33192500
O. meridionalis	meridionalis_boundary_492	meridionalis_chr03	33232500	33237500
O. meridionalis	meridionalis_boundary_493	meridionalis_chr03	33617500	33682500
O. meridionalis	meridionalis_boundary_494	meridionalis_chr03	33757500	33762500
O. meridionalis	meridionalis_boundary_495	meridionalis_chr03	33802500	33807500
O. meridionalis	meridionalis_boundary_496	meridionalis_chr03	33872500	33877500
O. meridionalis	meridionalis_boundary_497	meridionalis_chr03	34057500	34062500
O. meridionalis	meridionalis_boundary_498	meridionalis_chr03	34112500	34117500
O. meridionalis	meridionalis_boundary_499	meridionalis_chr03	34227500	34232500
O. meridionalis	meridionalis_boundary_500	meridionalis_chr03	34282500	34287500
O. meridionalis	meridionalis_boundary_501	meridionalis_chr03	34422500	34462500
O. meridionalis	meridionalis_boundary_502	meridionalis_chr03	35517500	35597500
O. meridionalis	meridionalis_boundary_503	meridionalis_chr03	35692500	35697500
O. meridionalis	meridionalis_boundary_504	meridionalis_chr03	35742500	35787500

O. meridionalis	meridionalis_boundary_505	meridionalis_chr03	35832500	35837500
O. meridionalis	meridionalis_boundary_506	meridionalis_chr03	35892500	35897500
O. meridionalis	meridionalis_boundary_507	meridionalis_chr03	36132500	36137500
O. meridionalis	meridionalis_boundary_508	meridionalis_chr03	36185000	36190000
O. meridionalis	meridionalis_boundary_509	meridionalis_chr03	36267500	36272500
O. meridionalis	meridionalis_boundary_510	meridionalis_chr03	36662500	36667500
O. meridionalis	meridionalis_boundary_511	meridionalis_chr03	36732500	36777500
O. meridionalis	meridionalis_boundary_512	meridionalis_chr03	37137500	37142500
O. meridionalis	meridionalis_boundary_513	meridionalis_chr03	37187500	37192500
O. meridionalis	meridionalis_boundary_514	meridionalis_chr03	37282500	37327500
O. meridionalis	meridionalis_boundary_515	meridionalis_chr03	37732500	37737500
O. meridionalis	meridionalis_boundary_516	meridionalis_chr03	37792500	37797500
O. meridionalis	meridionalis_boundary_517	meridionalis_chr03	37922500	37927500
O. meridionalis	meridionalis_boundary_518	meridionalis_chr03	37972500	37977500
O. meridionalis	meridionalis_boundary_519	meridionalis_chr03	38037500	38042500
O. meridionalis	meridionalis_boundary_520	meridionalis_chr03	38097500	38102500
O. meridionalis	meridionalis_boundary_521	meridionalis_chr03	38187500	38192500
O. meridionalis	meridionalis_boundary_522	meridionalis_chr03	38292500	38297500
O. meridionalis	meridionalis_boundary_523	meridionalis_chr03	38337500	38342500
O. meridionalis	meridionalis_boundary_524	meridionalis_chr03	38447500	38452500
O. meridionalis	meridionalis_boundary_525	meridionalis_chr03	38497500	38502500
O. meridionalis	meridionalis_boundary_526	meridionalis_chr03	38547500	38552500
O. meridionalis	meridionalis_boundary_527	meridionalis_chr03	38637500	38642500
O. meridionalis	meridionalis_boundary_528	meridionalis_chr03	38717500	38722500
O. meridionalis	meridionalis_boundary_529	meridionalis_chr03	38777500	38782500
O. meridionalis	meridionalis_boundary_530	meridionalis_chr03	38822500	38857500
O. meridionalis	meridionalis_boundary_531	meridionalis_chr03	38920000	38925000
O. meridionalis	meridionalis_boundary_532	meridionalis_chr03	39007500	39012500
O. meridionalis	meridionalis_boundary_533	meridionalis_chr03	39137500	39142500
O. meridionalis	meridionalis_boundary_534	meridionalis_chr03	39212500	39217500
O. meridionalis	meridionalis_boundary_535	meridionalis_chr03	39587500	39592500
O. meridionalis	meridionalis_boundary_536	meridionalis_chr03	39717500	39722500

O. meridionalis	meridionalis_boundary_537	meridionalis_chr03	39782500	39787500
O. meridionalis	meridionalis_boundary_538	meridionalis_chr03	39847500	39852500
O. meridionalis	meridionalis_boundary_539	meridionalis_chr03	39897500	39902500
O. meridionalis	meridionalis_boundary_540	meridionalis_chr04	317500	322500
O. meridionalis	meridionalis_boundary_541	meridionalis_chr04	367500	372500
O. meridionalis	meridionalis_boundary_542	meridionalis_chr04	1332500	1337500
O. meridionalis	meridionalis_boundary_543	meridionalis_chr04	1417500	1422500
O. meridionalis	meridionalis_boundary_544	meridionalis_chr04	1500000	1505000
O. meridionalis	meridionalis_boundary_545	meridionalis_chr04	1942500	1947500
O. meridionalis	meridionalis_boundary_546	meridionalis_chr04	2007500	2012500
O. meridionalis	meridionalis_boundary_547	meridionalis_chr04	2862500	2867500
O. meridionalis	meridionalis_boundary_548	meridionalis_chr04	3827500	3832500
O. meridionalis	meridionalis_boundary_549	meridionalis_chr04	3937500	3942500
O. meridionalis	meridionalis_boundary_550	meridionalis_chr04	5897500	5902500
O. meridionalis	meridionalis_boundary_551	meridionalis_chr04	6247500	6252500
O. meridionalis	meridionalis_boundary_552	meridionalis_chr04	6317500	6322500
O. meridionalis	meridionalis_boundary_553	meridionalis_chr04	6367500	6372500
O. meridionalis	meridionalis_boundary_554	meridionalis_chr04	6732500	6737500
O. meridionalis	meridionalis_boundary_555	meridionalis_chr04	6857500	6892500
O. meridionalis	meridionalis_boundary_556	meridionalis_chr04	7067500	7072500
O. meridionalis	meridionalis_boundary_557	meridionalis_chr04	7947500	7952500
O. meridionalis	meridionalis_boundary_558	meridionalis_chr04	8077500	8082500
O. meridionalis	meridionalis_boundary_559	meridionalis_chr04	8155000	8160000
O. meridionalis	meridionalis_boundary_560	meridionalis_chr04	8202500	8207500
O. meridionalis	meridionalis_boundary_561	meridionalis_chr04	10002500	10007500
O. meridionalis	meridionalis_boundary_562	meridionalis_chr04	10132500	10137500
O. meridionalis	meridionalis_boundary_563	meridionalis_chr04	10177500	10182500
O. meridionalis	meridionalis_boundary_564	meridionalis_chr04	10242500	10247500
O. meridionalis	meridionalis_boundary_565	meridionalis_chr04	10777500	10782500
O. meridionalis	meridionalis_boundary_566	meridionalis_chr04	10827500	10832500
O. meridionalis	meridionalis_boundary_567	meridionalis_chr04	10902500	10907500
O. meridionalis	meridionalis_boundary_568	meridionalis_chr04	10987500	10992500

O. meridionalis	meridionalis_boundary_569	meridionalis_chr04	11192500	11197500
O. meridionalis	meridionalis_boundary_570	meridionalis_chr04	12827500	12832500
O. meridionalis	meridionalis_boundary_571	meridionalis_chr04	12872500	12877500
O. meridionalis	meridionalis_boundary_572	meridionalis_chr04	13127500	13132500
O. meridionalis	meridionalis_boundary_573	meridionalis_chr04	13207500	13212500
O. meridionalis	meridionalis_boundary_574	meridionalis_chr04	13982500	13987500
O. meridionalis	meridionalis_boundary_575	meridionalis_chr04	14267500	14272500
O. meridionalis	meridionalis_boundary_576	meridionalis_chr04	14602500	14607500
O. meridionalis	meridionalis_boundary_577	meridionalis_chr04	14737500	14742500
O. meridionalis	meridionalis_boundary_578	meridionalis_chr04	16932500	16937500
O. meridionalis	meridionalis_boundary_579	meridionalis_chr04	16992500	16997500
O. meridionalis	meridionalis_boundary_580	meridionalis_chr04	17302500	17307500
O. meridionalis	meridionalis_boundary_581	meridionalis_chr04	17387500	17392500
O. meridionalis	meridionalis_boundary_582	meridionalis_chr04	17467500	17512500
O. meridionalis	meridionalis_boundary_583	meridionalis_chr04	17577500	17617500
O. meridionalis	meridionalis_boundary_584	meridionalis_chr04	17707500	17752500
O. meridionalis	meridionalis_boundary_585	meridionalis_chr04	17792500	17797500
O. meridionalis	meridionalis_boundary_586	meridionalis_chr04	17842500	17847500
O. meridionalis	meridionalis_boundary_587	meridionalis_chr04	17917500	17922500
O. meridionalis	meridionalis_boundary_588	meridionalis_chr04	18207500	18212500
O. meridionalis	meridionalis_boundary_589	meridionalis_chr04	18360000	18365000
O. meridionalis	meridionalis_boundary_590	meridionalis_chr04	18502500	18507500
O. meridionalis	meridionalis_boundary_591	meridionalis_chr04	18587500	18592500
O. meridionalis	meridionalis_boundary_592	meridionalis_chr04	18642500	18647500
O. meridionalis	meridionalis_boundary_593	meridionalis_chr04	18697500	18702500
O. meridionalis	meridionalis_boundary_594	meridionalis_chr04	18837500	18842500
O. meridionalis	meridionalis_boundary_595	meridionalis_chr04	18907500	18912500
O. meridionalis	meridionalis_boundary_596	meridionalis_chr04	19682500	19687500
O. meridionalis	meridionalis_boundary_597	meridionalis_chr04	19752500	19757500
O. meridionalis	meridionalis_boundary_598	meridionalis_chr04	19807500	19812500
O. meridionalis	meridionalis_boundary_599	meridionalis_chr04	20227500	20232500
O. meridionalis	meridionalis_boundary_600	meridionalis_chr04	20292500	20297500

O. meridionalis	meridionalis_boundary_601	meridionalis_chr04	20377500	20382500
O. meridionalis	meridionalis_boundary_602	meridionalis_chr04	20487500	20492500
O. meridionalis	meridionalis_boundary_603	meridionalis_chr04	20797500	20802500
O. meridionalis	meridionalis_boundary_604	meridionalis_chr04	20842500	20847500
O. meridionalis	meridionalis_boundary_605	meridionalis_chr04	20892500	20897500
O. meridionalis	meridionalis_boundary_606	meridionalis_chr04	20972500	21017500
O. meridionalis	meridionalis_boundary_607	meridionalis_chr04	22812500	22892500
O. meridionalis	meridionalis_boundary_608	meridionalis_chr04	22932500	22937500
O. meridionalis	meridionalis_boundary_609	meridionalis_chr04	22987500	22992500
O. meridionalis	meridionalis_boundary_610	meridionalis_chr04	23112500	23117500
O. meridionalis	meridionalis_boundary_611	meridionalis_chr04	23187500	23192500
O. meridionalis	meridionalis_boundary_612	meridionalis_chr04	23277500	23282500
O. meridionalis	meridionalis_boundary_613	meridionalis_chr04	23342500	23347500
O. meridionalis	meridionalis_boundary_614	meridionalis_chr04	23412500	23417500
O. meridionalis	meridionalis_boundary_615	meridionalis_chr04	23492500	23497500
O. meridionalis	meridionalis_boundary_616	meridionalis_chr04	23537500	23542500
O. meridionalis	meridionalis_boundary_617	meridionalis_chr04	23942500	23947500
O. meridionalis	meridionalis_boundary_618	meridionalis_chr04	24032500	24037500
O. meridionalis	meridionalis_boundary_619	meridionalis_chr04	24437500	24442500
O. meridionalis	meridionalis_boundary_620	meridionalis_chr04	24527500	24532500
O. meridionalis	meridionalis_boundary_621	meridionalis_chr04	25007500	25012500
O. meridionalis	meridionalis_boundary_622	meridionalis_chr04	25067500	25072500
O. meridionalis	meridionalis_boundary_623	meridionalis_chr04	25277500	25322500
O. meridionalis	meridionalis_boundary_624	meridionalis_chr04	25427500	25432500
O. meridionalis	meridionalis_boundary_625	meridionalis_chr04	25487500	25492500
O. meridionalis	meridionalis_boundary_626	meridionalis_chr04	25887500	25892500
O. meridionalis	meridionalis_boundary_627	meridionalis_chr04	26027500	26032500
O. meridionalis	meridionalis_boundary_628	meridionalis_chr04	26757500	26762500
O. meridionalis	meridionalis_boundary_629	meridionalis_chr04	26892500	26897500
O. meridionalis	meridionalis_boundary_630	meridionalis_chr04	27002500	27007500
O. meridionalis	meridionalis_boundary_631	meridionalis_chr04	27072500	27077500
O. meridionalis	meridionalis_boundary_632	meridionalis_chr04	27477500	27482500

O. meridionalis	meridionalis_boundary_633	meridionalis_chr04	27562500	27567500
O. meridionalis	meridionalis_boundary_634	meridionalis_chr04	27777500	27817500
O. meridionalis	meridionalis_boundary_635	meridionalis_chr04	27867500	27872500
O. meridionalis	meridionalis_boundary_636	meridionalis_chr04	27967500	27972500
O. meridionalis	meridionalis_boundary_637	meridionalis_chr04	28327500	28332500
O. meridionalis	meridionalis_boundary_638	meridionalis_chr04	28397500	28402500
O. meridionalis	meridionalis_boundary_639	meridionalis_chr04	29087500	29092500
O. meridionalis	meridionalis_boundary_640	meridionalis_chr04	29132500	29172500
O. meridionalis	meridionalis_boundary_641	meridionalis_chr04	29952500	29957500
O. meridionalis	meridionalis_boundary_642	meridionalis_chr04	30017500	30022500
O. meridionalis	meridionalis_boundary_643	meridionalis_chr04	30202500	30207500
O. meridionalis	meridionalis_boundary_644	meridionalis_chr04	30277500	30317500
O. meridionalis	meridionalis_boundary_645	meridionalis_chr04	31157500	31197500
O. meridionalis	meridionalis_boundary_646	meridionalis_chr04	31272500	31277500
O. meridionalis	meridionalis_boundary_647	meridionalis_chr04	31397500	31402500
O. meridionalis	meridionalis_boundary_648	meridionalis_chr04	31562500	31567500
O. meridionalis	meridionalis_boundary_649	meridionalis_chr04	31612500	31617500
O. meridionalis	meridionalis_boundary_650	meridionalis_chr04	31667500	31672500
O. meridionalis	meridionalis_boundary_651	meridionalis_chr04	31792500	31797500
O. meridionalis	meridionalis_boundary_652	meridionalis_chr04	31887500	31892500
O. meridionalis	meridionalis_boundary_653	meridionalis_chr04	32422500	32427500
O. meridionalis	meridionalis_boundary_654	meridionalis_chr04	32507500	32512500
O. meridionalis	meridionalis_boundary_655	meridionalis_chr04	33162500	33167500
O. meridionalis	meridionalis_boundary_656	meridionalis_chr04	33267500	33272500
O. meridionalis	meridionalis_boundary_657	meridionalis_chr04	33337500	33342500
O. meridionalis	meridionalis_boundary_658	meridionalis_chr04	33397500	33402500
O. meridionalis	meridionalis_boundary_659	meridionalis_chr04	33962500	33967500
O. meridionalis	meridionalis_boundary_660	meridionalis_chr04	34077500	34082500
O. meridionalis	meridionalis_boundary_661	meridionalis_chr05	202500	207500
O. meridionalis	meridionalis_boundary_662	meridionalis_chr05	277500	282500
O. meridionalis	meridionalis_boundary_663	meridionalis_chr05	332500	337500
O. meridionalis	meridionalis_boundary_664	meridionalis_chr05	962500	967500

O. meridionalis	meridionalis_boundary_665	meridionalis_chr05	1027500	1032500
O. meridionalis	meridionalis_boundary_666	meridionalis_chr05	1095000	1100000
O. meridionalis	meridionalis_boundary_667	meridionalis_chr05	1177500	1182500
O. meridionalis	meridionalis_boundary_668	meridionalis_chr05	1557500	1562500
O. meridionalis	meridionalis_boundary_669	meridionalis_chr05	1757500	1762500
O. meridionalis	meridionalis_boundary_670	meridionalis_chr05	2012500	2017500
O. meridionalis	meridionalis_boundary_671	meridionalis_chr05	2087500	2092500
O. meridionalis	meridionalis_boundary_672	meridionalis_chr05	2165000	2170000
O. meridionalis	meridionalis_boundary_673	meridionalis_chr05	2232500	2237500
O. meridionalis	meridionalis_boundary_674	meridionalis_chr05	2667500	2672500
O. meridionalis	meridionalis_boundary_675	meridionalis_chr05	2732500	2737500
O. meridionalis	meridionalis_boundary_676	meridionalis_chr05	3312500	3317500
O. meridionalis	meridionalis_boundary_677	meridionalis_chr05	3447500	3492500
O. meridionalis	meridionalis_boundary_678	meridionalis_chr05	3582500	3587500
O. meridionalis	meridionalis_boundary_679	meridionalis_chr05	4837500	4867500
O. meridionalis	meridionalis_boundary_680	meridionalis_chr05	5217500	5222500
O. meridionalis	meridionalis_boundary_681	meridionalis_chr05	5267500	5272500
O. meridionalis	meridionalis_boundary_682	meridionalis_chr05	5322500	5327500
O. meridionalis	meridionalis_boundary_683	meridionalis_chr05	5382500	5387500
O. meridionalis	meridionalis_boundary_684	meridionalis_chr05	5442500	5447500
O. meridionalis	meridionalis_boundary_685	meridionalis_chr05	5607500	5612500
O. meridionalis	meridionalis_boundary_686	meridionalis_chr05	5687500	5692500
O. meridionalis	meridionalis_boundary_687	meridionalis_chr05	6612500	6617500
O. meridionalis	meridionalis_boundary_688	meridionalis_chr05	6667500	6672500
O. meridionalis	meridionalis_boundary_689	meridionalis_chr05	6752500	6757500
O. meridionalis	meridionalis_boundary_690	meridionalis_chr05	6807500	6812500
O. meridionalis	meridionalis_boundary_691	meridionalis_chr05	6897500	6902500
O. meridionalis	meridionalis_boundary_692	meridionalis_chr05	7132500	7137500
O. meridionalis	meridionalis_boundary_693	meridionalis_chr05	7212500	7217500
O. meridionalis	meridionalis_boundary_694	meridionalis_chr05	7312500	7357500
O. meridionalis	meridionalis_boundary_695	meridionalis_chr05	7437500	7442500
O. meridionalis	meridionalis_boundary_696	meridionalis_chr05	7522500	7527500

O. meridionalis	meridionalis_boundary_697	meridionalis_chr05	7717500	7722500
O. meridionalis	meridionalis_boundary_698	meridionalis_chr05	7847500	7852500
O. meridionalis	meridionalis_boundary_699	meridionalis_chr05	8042500	8047500
O. meridionalis	meridionalis_boundary_700	meridionalis_chr05	8137500	8142500
O. meridionalis	meridionalis_boundary_701	meridionalis_chr05	8235000	8240000
O. meridionalis	meridionalis_boundary_702	meridionalis_chr05	8407500	8412500
O. meridionalis	meridionalis_boundary_703	meridionalis_chr05	8507500	8512500
O. meridionalis	meridionalis_boundary_704	meridionalis_chr05	8657500	8662500
O. meridionalis	meridionalis_boundary_705	meridionalis_chr05	8712500	8717500
O. meridionalis	meridionalis_boundary_706	meridionalis_chr05	8817500	8822500
O. meridionalis	meridionalis_boundary_707	meridionalis_chr05	8892500	8897500
O. meridionalis	meridionalis_boundary_708	meridionalis_chr05	9027500	9032500
O. meridionalis	meridionalis_boundary_709	meridionalis_chr05	9142500	9147500
O. meridionalis	meridionalis_boundary_710	meridionalis_chr05	9187500	9192500
O. meridionalis	meridionalis_boundary_711	meridionalis_chr05	9477500	9525000
O. meridionalis	meridionalis_boundary_712	meridionalis_chr05	9727500	9732500
O. meridionalis	meridionalis_boundary_713	meridionalis_chr05	9777500	9817500
O. meridionalis	meridionalis_boundary_714	meridionalis_chr05	10097500	10102500
O. meridionalis	meridionalis_boundary_715	meridionalis_chr05	10272500	10277500
O. meridionalis	meridionalis_boundary_716	meridionalis_chr05	10592500	10637500
O. meridionalis	meridionalis_boundary_717	meridionalis_chr05	12902500	12907500
O. meridionalis	meridionalis_boundary_718	meridionalis_chr05	12977500	12982500
O. meridionalis	meridionalis_boundary_719	meridionalis_chr05	13107500	13112500
O. meridionalis	meridionalis_boundary_720	meridionalis_chr05	13522500	13527500
O. meridionalis	meridionalis_boundary_721	meridionalis_chr05	13602500	13607500
O. meridionalis	meridionalis_boundary_722	meridionalis_chr05	21697500	21772500
O. meridionalis	meridionalis_boundary_723	meridionalis_chr05	21902500	21907500
O. meridionalis	meridionalis_boundary_724	meridionalis_chr05	21967500	21972500
O. meridionalis	meridionalis_boundary_725	meridionalis_chr05	22112500	22117500
O. meridionalis	meridionalis_boundary_726	meridionalis_chr05	22162500	22167500
O. meridionalis	meridionalis_boundary_727	meridionalis_chr05	22602500	22642500
O. meridionalis	meridionalis_boundary_728	meridionalis_chr05	22722500	22727500

O. meridionalis	meridionalis_boundary_729	meridionalis_chr05	22792500	22797500
O. meridionalis	meridionalis_boundary_730	meridionalis_chr05	23002500	23007500
O. meridionalis	meridionalis_boundary_731	meridionalis_chr05	23062500	23067500
O. meridionalis	meridionalis_boundary_732	meridionalis_chr05	23187500	23192500
O. meridionalis	meridionalis_boundary_733	meridionalis_chr05	23257500	23262500
O. meridionalis	meridionalis_boundary_734	meridionalis_chr05	23337500	23372500
O. meridionalis	meridionalis_boundary_735	meridionalis_chr05	23502500	23507500
O. meridionalis	meridionalis_boundary_736	meridionalis_chr05	23607500	23612500
O. meridionalis	meridionalis_boundary_737	meridionalis_chr05	23822500	23827500
O. meridionalis	meridionalis_boundary_738	meridionalis_chr05	23887500	23892500
O. meridionalis	meridionalis_boundary_739	meridionalis_chr05	23952500	23957500
O. meridionalis	meridionalis_boundary_740	meridionalis_chr05	24412500	24417500
O. meridionalis	meridionalis_boundary_741	meridionalis_chr05	24492500	24547500
O. meridionalis	meridionalis_boundary_742	meridionalis_chr05	24602500	24607500
O. meridionalis	meridionalis_boundary_743	meridionalis_chr05	24647500	24652500
O. meridionalis	meridionalis_boundary_744	meridionalis_chr05	24842500	24847500
O. meridionalis	meridionalis_boundary_745	meridionalis_chr05	24887500	24892500
O. meridionalis	meridionalis_boundary_746	meridionalis_chr05	24962500	24967500
O. meridionalis	meridionalis_boundary_747	meridionalis_chr05	25617500	25622500
O. meridionalis	meridionalis_boundary_748	meridionalis_chr05	25667500	25672500
O. meridionalis	meridionalis_boundary_749	meridionalis_chr05	25712500	25717500
O. meridionalis	meridionalis_boundary_750	meridionalis_chr05	25757500	25762500
O. meridionalis	meridionalis_boundary_751	meridionalis_chr05	25857500	25862500
O. meridionalis	meridionalis_boundary_752	meridionalis_chr05	25947500	25952500
O. meridionalis	meridionalis_boundary_753	meridionalis_chr05	25992500	25997500
O. meridionalis	meridionalis_boundary_754	meridionalis_chr05	26142500	26147500
O. meridionalis	meridionalis_boundary_755	meridionalis_chr05	26197500	26202500
O. meridionalis	meridionalis_boundary_756	meridionalis_chr05	26677500	26682500
O. meridionalis	meridionalis_boundary_757	meridionalis_chr05	26742500	26747500
O. meridionalis	meridionalis_boundary_758	meridionalis_chr05	26817500	26822500
O. meridionalis	meridionalis_boundary_759	meridionalis_chr05	26912500	26947500
O. meridionalis	meridionalis_boundary_760	meridionalis_chr05	27162500	27167500

O. meridionalis	meridionalis_boundary_761	meridionalis_chr05	27212500	27257500
O. meridionalis	meridionalis_boundary_762	meridionalis_chr05	27442500	27447500
O. meridionalis	meridionalis_boundary_763	meridionalis_chr05	27562500	27567500
O. meridionalis	meridionalis_boundary_764	meridionalis_chr05	27872500	27877500
O. meridionalis	meridionalis_boundary_765	meridionalis_chr05	27952500	27957500
O. meridionalis	meridionalis_boundary_766	meridionalis_chr05	28052500	28057500
O. meridionalis	meridionalis_boundary_767	meridionalis_chr05	28107500	28112500
O. meridionalis	meridionalis_boundary_768	meridionalis_chr05	28482500	28487500
O. meridionalis	meridionalis_boundary_769	meridionalis_chr05	28537500	28542500
O. meridionalis	meridionalis_boundary_770	meridionalis_chr05	28592500	28597500
O. meridionalis	meridionalis_boundary_771	meridionalis_chr05	28852500	28857500
O. meridionalis	meridionalis_boundary_772	meridionalis_chr05	28942500	28947500
O. meridionalis	meridionalis_boundary_773	meridionalis_chr05	28997500	29002500
O. meridionalis	meridionalis_boundary_774	meridionalis_chr06	162500	167500
O. meridionalis	meridionalis_boundary_775	meridionalis_chr06	357500	362500
O. meridionalis	meridionalis_boundary_776	meridionalis_chr06	612500	617500
O. meridionalis	meridionalis_boundary_777	meridionalis_chr06	702500	707500
O. meridionalis	meridionalis_boundary_778	meridionalis_chr06	2042500	2047500
O. meridionalis	meridionalis_boundary_779	meridionalis_chr06	2112500	2117500
O. meridionalis	meridionalis_boundary_780	meridionalis_chr06	2167500	2172500
O. meridionalis	meridionalis_boundary_781	meridionalis_chr06	2242500	2247500
O. meridionalis	meridionalis_boundary_782	meridionalis_chr06	2512500	2517500
O. meridionalis	meridionalis_boundary_783	meridionalis_chr06	2582500	2587500
O. meridionalis	meridionalis_boundary_784	meridionalis_chr06	2707500	2747500
O. meridionalis	meridionalis_boundary_785	meridionalis_chr06	3072500	3077500
O. meridionalis	meridionalis_boundary_786	meridionalis_chr06	3132500	3137500
O. meridionalis	meridionalis_boundary_787	meridionalis_chr06	3202500	3207500
O. meridionalis	meridionalis_boundary_788	meridionalis_chr06	3272500	3277500
O. meridionalis	meridionalis_boundary_789	meridionalis_chr06	3632500	3637500
O. meridionalis	meridionalis_boundary_790	meridionalis_chr06	3692500	3697500
O. meridionalis	meridionalis_boundary_791	meridionalis_chr06	4267500	4272500
O. meridionalis	meridionalis_boundary_792	meridionalis_chr06	4312500	4317500

O. meridionalis	meridionalis_boundary_793	meridionalis_chr06	4422500	4427500
O. meridionalis	meridionalis_boundary_794	meridionalis_chr06	4542500	4547500
O. meridionalis	meridionalis_boundary_795	meridionalis_chr06	4592500	4597500
O. meridionalis	meridionalis_boundary_796	meridionalis_chr06	4707500	4752500
O. meridionalis	meridionalis_boundary_797	meridionalis_chr06	4942500	4947500
O. meridionalis	meridionalis_boundary_798	meridionalis_chr06	4987500	4992500
O. meridionalis	meridionalis_boundary_799	meridionalis_chr06	5212500	5217500
O. meridionalis	meridionalis_boundary_800	meridionalis_chr06	5292500	5297500
O. meridionalis	meridionalis_boundary_801	meridionalis_chr06	5357500	5362500
O. meridionalis	meridionalis_boundary_802	meridionalis_chr06	5422500	5427500
O. meridionalis	meridionalis_boundary_803	meridionalis_chr06	5497500	5537500
O. meridionalis	meridionalis_boundary_804	meridionalis_chr06	5592500	5597500
O. meridionalis	meridionalis_boundary_805	meridionalis_chr06	5782500	5827500
O. meridionalis	meridionalis_boundary_806	meridionalis_chr06	6742500	6747500
O. meridionalis	meridionalis_boundary_807	meridionalis_chr06	6787500	6792500
O. meridionalis	meridionalis_boundary_808	meridionalis_chr06	7292500	7332500
O. meridionalis	meridionalis_boundary_809	meridionalis_chr06	7747500	7752500
O. meridionalis	meridionalis_boundary_810	meridionalis_chr06	7792500	7797500
O. meridionalis	meridionalis_boundary_811	meridionalis_chr06	7922500	7927500
O. meridionalis	meridionalis_boundary_812	meridionalis_chr06	7987500	7992500
O. meridionalis	meridionalis_boundary_813	meridionalis_chr06	8117500	8122500
O. meridionalis	meridionalis_boundary_814	meridionalis_chr06	8217500	8222500
O. meridionalis	meridionalis_boundary_815	meridionalis_chr06	9112500	9117500
O. meridionalis	meridionalis_boundary_816	meridionalis_chr06	9197500	9202500
O. meridionalis	meridionalis_boundary_817	meridionalis_chr06	10242500	10247500
O. meridionalis	meridionalis_boundary_818	meridionalis_chr06	10307500	10312500
O. meridionalis	meridionalis_boundary_819	meridionalis_chr06	10722500	10727500
O. meridionalis	meridionalis_boundary_820	meridionalis_chr06	10802500	10807500
O. meridionalis	meridionalis_boundary_821	meridionalis_chr06	10892500	10897500
O. meridionalis	meridionalis_boundary_822	meridionalis_chr06	10997500	11002500
O. meridionalis	meridionalis_boundary_823	meridionalis_chr06	11752500	11757500
O. meridionalis	meridionalis_boundary_824	meridionalis_chr06	11807500	11812500

O. meridionalis	meridionalis_boundary_825	meridionalis_chr06	11992500	11997500
O. meridionalis	meridionalis_boundary_826	meridionalis_chr06	12847500	12852500
O. meridionalis	meridionalis_boundary_827	meridionalis_chr06	12930000	12935000
O. meridionalis	meridionalis_boundary_828	meridionalis_chr06	13047500	13087500
O. meridionalis	meridionalis_boundary_829	meridionalis_chr06	13152500	13157500
O. meridionalis	meridionalis_boundary_830	meridionalis_chr06	13442500	13447500
O. meridionalis	meridionalis_boundary_831	meridionalis_chr06	13512500	13517500
O. meridionalis	meridionalis_boundary_832	meridionalis_chr06	14827500	14832500
O. meridionalis	meridionalis_boundary_833	meridionalis_chr06	14972500	14977500
O. meridionalis	meridionalis_boundary_834	meridionalis_chr06	15052500	15057500
O. meridionalis	meridionalis_boundary_835	meridionalis_chr06	17047500	17082500
O. meridionalis	meridionalis_boundary_836	meridionalis_chr06	18257500	18262500
O. meridionalis	meridionalis_boundary_837	meridionalis_chr06	18385000	18390000
O. meridionalis	meridionalis_boundary_838	meridionalis_chr06	19402500	19407500
O. meridionalis	meridionalis_boundary_839	meridionalis_chr06	19510000	19515000
O. meridionalis	meridionalis_boundary_840	meridionalis_chr06	19582500	19587500
O. meridionalis	meridionalis_boundary_841	meridionalis_chr06	19742500	19747500
O. meridionalis	meridionalis_boundary_842	meridionalis_chr06	20552500	20557500
O. meridionalis	meridionalis_boundary_843	meridionalis_chr06	20662500	20667500
O. meridionalis	meridionalis_boundary_844	meridionalis_chr06	20952500	20957500
O. meridionalis	meridionalis_boundary_845	meridionalis_chr06	21062500	21067500
O. meridionalis	meridionalis_boundary_846	meridionalis_chr06	21192500	21197500
O. meridionalis	meridionalis_boundary_847	meridionalis_chr06	21627500	21662500
O. meridionalis	meridionalis_boundary_848	meridionalis_chr06	21707500	21712500
O. meridionalis	meridionalis_boundary_849	meridionalis_chr06	22167500	22197500
O. meridionalis	meridionalis_boundary_850	meridionalis_chr06	22287500	22292500
O. meridionalis	meridionalis_boundary_851	meridionalis_chr06	22477500	22482500
O. meridionalis	meridionalis_boundary_852	meridionalis_chr06	22557500	22562500
O. meridionalis	meridionalis_boundary_853	meridionalis_chr06	22912500	22917500
O. meridionalis	meridionalis_boundary_854	meridionalis_chr06	22962500	22967500
O. meridionalis	meridionalis_boundary_855	meridionalis_chr06	23062500	23067500
O. meridionalis	meridionalis_boundary_856	meridionalis_chr06	23132500	23172500

O. meridionalis	meridionalis_boundary_857	meridionalis_chr06	23252500	23257500
O. meridionalis	meridionalis_boundary_858	meridionalis_chr06	23317500	23322500
O. meridionalis	meridionalis_boundary_859	meridionalis_chr06	23382500	23387500
O. meridionalis	meridionalis_boundary_860	meridionalis_chr06	23827500	23832500
O. meridionalis	meridionalis_boundary_861	meridionalis_chr06	23917500	23922500
O. meridionalis	meridionalis_boundary_862	meridionalis_chr06	24002500	24007500
O. meridionalis	meridionalis_boundary_863	meridionalis_chr06	24087500	24092500
O. meridionalis	meridionalis_boundary_864	meridionalis_chr06	24172500	24177500
O. meridionalis	meridionalis_boundary_865	meridionalis_chr06	24232500	24237500
O. meridionalis	meridionalis_boundary_866	meridionalis_chr06	24502500	24507500
O. meridionalis	meridionalis_boundary_867	meridionalis_chr06	24550000	24582500
O. meridionalis	meridionalis_boundary_868	meridionalis_chr06	24662500	24667500
O. meridionalis	meridionalis_boundary_869	meridionalis_chr06	24792500	24797500
O. meridionalis	meridionalis_boundary_870	meridionalis_chr06	24892500	24897500
O. meridionalis	meridionalis_boundary_871	meridionalis_chr06	25067500	25072500
O. meridionalis	meridionalis_boundary_872	meridionalis_chr06	25187500	25192500
O. meridionalis	meridionalis_boundary_873	meridionalis_chr06	25247500	25252500
O. meridionalis	meridionalis_boundary_874	meridionalis_chr06	25332500	25337500
O. meridionalis	meridionalis_boundary_875	meridionalis_chr06	25437500	25517500
O. meridionalis	meridionalis_boundary_876	meridionalis_chr06	25562500	25567500
O. meridionalis	meridionalis_boundary_877	meridionalis_chr06	25617500	25622500
O. meridionalis	meridionalis_boundary_878	meridionalis_chr06	25777500	25782500
O. meridionalis	meridionalis_boundary_879	meridionalis_chr06	26092500	26097500
O. meridionalis	meridionalis_boundary_880	meridionalis_chr06	26152500	26157500
O. meridionalis	meridionalis_boundary_881	meridionalis_chr06	26212500	26217500
O. meridionalis	meridionalis_boundary_882	meridionalis_chr06	26387500	26392500
O. meridionalis	meridionalis_boundary_883	meridionalis_chr06	26602500	26607500
O. meridionalis	meridionalis_boundary_884	meridionalis_chr06	26862500	26867500
O. meridionalis	meridionalis_boundary_885	meridionalis_chr06	26937500	26942500
O. meridionalis	meridionalis_boundary_886	meridionalis_chr06	27057500	27097500
O. meridionalis	meridionalis_boundary_887	meridionalis_chr06	27692500	27697500
O. meridionalis	meridionalis_boundary_888	meridionalis_chr06	27757500	27762500

O. meridionalis	meridionalis_boundary_889	meridionalis_chr06	27822500	27857500
O. meridionalis	meridionalis_boundary_890	meridionalis_chr06	28082500	28087500
O. meridionalis	meridionalis_boundary_891	meridionalis_chr06	28137500	28142500
O. meridionalis	meridionalis_boundary_892	meridionalis_chr06	28232500	28272500
O. meridionalis	meridionalis_boundary_893	meridionalis_chr06	28342500	28347500
O. meridionalis	meridionalis_boundary_894	meridionalis_chr06	28427500	28432500
O. meridionalis	meridionalis_boundary_895	meridionalis_chr06	28472500	28477500
O. meridionalis	meridionalis_boundary_896	meridionalis_chr06	28542500	28547500
O. meridionalis	meridionalis_boundary_897	meridionalis_chr06	28597500	28602500
O. meridionalis	meridionalis_boundary_898	meridionalis_chr06	28732500	28737500
O. meridionalis	meridionalis_boundary_899	meridionalis_chr06	29062500	29067500
O. meridionalis	meridionalis_boundary_900	meridionalis_chr06	29107500	29112500
O. meridionalis	meridionalis_boundary_901	meridionalis_chr06	29577500	29582500
O. meridionalis	meridionalis_boundary_902	meridionalis_chr06	29622500	29627500
O. meridionalis	meridionalis_boundary_903	meridionalis_chr06	29692500	29697500
O. meridionalis	meridionalis_boundary_904	meridionalis_chr06	30102500	30107500
O. meridionalis	meridionalis_boundary_905	meridionalis_chr06	30167500	30172500
O. meridionalis	meridionalis_boundary_906	meridionalis_chr06	30242500	30247500
O. meridionalis	meridionalis_boundary_907	meridionalis_chr06	30507500	30512500
O. meridionalis	meridionalis_boundary_908	meridionalis_chr06	30557500	30562500
O. meridionalis	meridionalis_boundary_909	meridionalis_chr06	30872500	30877500
O. meridionalis	meridionalis_boundary_910	meridionalis_chr06	30927500	30932500
O. meridionalis	meridionalis_boundary_911	meridionalis_chr07	512500	517500
O. meridionalis	meridionalis_boundary_912	meridionalis_chr07	657500	662500
O. meridionalis	meridionalis_boundary_913	meridionalis_chr07	762500	767500
O. meridionalis	meridionalis_boundary_914	meridionalis_chr07	1032500	1077500
O. meridionalis	meridionalis_boundary_915	meridionalis_chr07	1127500	1132500
O. meridionalis	meridionalis_boundary_916	meridionalis_chr07	1207500	1212500
O. meridionalis	meridionalis_boundary_917	meridionalis_chr07	1377500	1382500
O. meridionalis	meridionalis_boundary_918	meridionalis_chr07	1477500	1557500
O. meridionalis	meridionalis_boundary_919	meridionalis_chr07	1637500	1642500
O. meridionalis	meridionalis_boundary_920	meridionalis_chr07	1717500	1722500

O. meridionalis	meridionalis_boundary_921	meridionalis_chr07	1937500	1982500
O. meridionalis	meridionalis_boundary_922	meridionalis_chr07	2050000	2055000
O. meridionalis	meridionalis_boundary_923	meridionalis_chr07	2787500	2792500
O. meridionalis	meridionalis_boundary_924	meridionalis_chr07	2832500	2837500
O. meridionalis	meridionalis_boundary_925	meridionalis_chr07	2892500	2897500
O. meridionalis	meridionalis_boundary_926	meridionalis_chr07	3622500	3627500
O. meridionalis	meridionalis_boundary_927	meridionalis_chr07	3707500	3712500
O. meridionalis	meridionalis_boundary_928	meridionalis_chr07	3832500	3837500
O. meridionalis	meridionalis_boundary_929	meridionalis_chr07	4022500	4057500
O. meridionalis	meridionalis_boundary_930	meridionalis_chr07	4102500	4107500
O. meridionalis	meridionalis_boundary_931	meridionalis_chr07	4177500	4182500
O. meridionalis	meridionalis_boundary_932	meridionalis_chr07	4247500	4252500
O. meridionalis	meridionalis_boundary_933	meridionalis_chr07	4317500	4322500
O. meridionalis	meridionalis_boundary_934	meridionalis_chr07	4532500	4537500
O. meridionalis	meridionalis_boundary_935	meridionalis_chr07	4607500	4612500
O. meridionalis	meridionalis_boundary_936	meridionalis_chr07	4687500	4692500
O. meridionalis	meridionalis_boundary_937	meridionalis_chr07	4747500	4752500
O. meridionalis	meridionalis_boundary_938	meridionalis_chr07	4892500	4937500
O. meridionalis	meridionalis_boundary_939	meridionalis_chr07	5112500	5117500
O. meridionalis	meridionalis_boundary_940	meridionalis_chr07	5162500	5167500
O. meridionalis	meridionalis_boundary_941	meridionalis_chr07	5257500	5262500
O. meridionalis	meridionalis_boundary_942	meridionalis_chr07	5377500	5382500
O. meridionalis	meridionalis_boundary_943	meridionalis_chr07	5437500	5442500
O. meridionalis	meridionalis_boundary_944	meridionalis_chr07	5517500	5522500
O. meridionalis	meridionalis_boundary_945	meridionalis_chr07	6427500	6432500
O. meridionalis	meridionalis_boundary_946	meridionalis_chr07	6497500	6502500
O. meridionalis	meridionalis_boundary_947	meridionalis_chr07	9615000	9620000
O. meridionalis	meridionalis_boundary_948	meridionalis_chr07	9717500	9722500
O. meridionalis	meridionalis_boundary_949	meridionalis_chr07	10627500	10632500
O. meridionalis	meridionalis_boundary_950	meridionalis_chr07	10707500	10712500
O. meridionalis	meridionalis_boundary_951	meridionalis_chr07	11342500	11382500
O. meridionalis	meridionalis_boundary_952	meridionalis_chr07	11437500	11442500

O. meridionalis	meridionalis_boundary_953	meridionalis_chr07	11977500	11982500
O. meridionalis	meridionalis_boundary_954	meridionalis_chr07	12312500	12317500
O. meridionalis	meridionalis_boundary_955	meridionalis_chr07	12382500	12387500
O. meridionalis	meridionalis_boundary_956	meridionalis_chr07	12562500	12567500
O. meridionalis	meridionalis_boundary_957	meridionalis_chr07	12852500	12857500
O. meridionalis	meridionalis_boundary_958	meridionalis_chr07	12897500	12902500
O. meridionalis	meridionalis_boundary_959	meridionalis_chr07	13462500	13467500
O. meridionalis	meridionalis_boundary_960	meridionalis_chr07	13542500	13547500
O. meridionalis	meridionalis_boundary_961	meridionalis_chr07	13872500	13877500
O. meridionalis	meridionalis_boundary_962	meridionalis_chr07	13987500	13992500
O. meridionalis	meridionalis_boundary_963	meridionalis_chr07	14397500	14402500
O. meridionalis	meridionalis_boundary_964	meridionalis_chr07	14472500	14477500
O. meridionalis	meridionalis_boundary_965	meridionalis_chr07	14537500	14542500
O. meridionalis	meridionalis_boundary_966	meridionalis_chr07	15742500	15747500
O. meridionalis	meridionalis_boundary_967	meridionalis_chr07	15802500	15807500
O. meridionalis	meridionalis_boundary_968	meridionalis_chr07	16287500	16292500
O. meridionalis	meridionalis_boundary_969	meridionalis_chr07	16442500	16487500
O. meridionalis	meridionalis_boundary_970	meridionalis_chr07	17102500	17107500
O. meridionalis	meridionalis_boundary_971	meridionalis_chr07	17167500	17172500
O. meridionalis	meridionalis_boundary_972	meridionalis_chr07	17282500	17287500
O. meridionalis	meridionalis_boundary_973	meridionalis_chr07	17392500	17397500
O. meridionalis	meridionalis_boundary_974	meridionalis_chr07	17497500	17502500
O. meridionalis	meridionalis_boundary_975	meridionalis_chr07	18047500	18052500
O. meridionalis	meridionalis_boundary_976	meridionalis_chr07	18092500	18097500
O. meridionalis	meridionalis_boundary_977	meridionalis_chr07	18162500	18167500
O. meridionalis	meridionalis_boundary_978	meridionalis_chr07	18277500	18282500
O. meridionalis	meridionalis_boundary_979	meridionalis_chr07	19242500	19247500
O. meridionalis	meridionalis_boundary_980	meridionalis_chr07	19292500	19297500
O. meridionalis	meridionalis_boundary_981	meridionalis_chr07	19647500	19652500
O. meridionalis	meridionalis_boundary_982	meridionalis_chr07	19752500	19757500
O. meridionalis	meridionalis_boundary_983	meridionalis_chr07	19927500	19967500
O. meridionalis	meridionalis_boundary_984	meridionalis_chr07	20012500	20082500

O. meridionalis	meridionalis_boundary_985	meridionalis_chr07	20192500	20197500
O. meridionalis	meridionalis_boundary_986	meridionalis_chr07	20277500	20282500
O. meridionalis	meridionalis_boundary_987	meridionalis_chr07	20987500	20992500
O. meridionalis	meridionalis_boundary_988	meridionalis_chr07	21077500	21112500
O. meridionalis	meridionalis_boundary_989	meridionalis_chr07	21672500	21702500
O. meridionalis	meridionalis_boundary_990	meridionalis_chr07	21752500	21757500
O. meridionalis	meridionalis_boundary_991	meridionalis_chr07	21827500	21832500
O. meridionalis	meridionalis_boundary_992	meridionalis_chr07	21997500	22002500
O. meridionalis	meridionalis_boundary_993	meridionalis_chr07	22342500	22347500
O. meridionalis	meridionalis_boundary_994	meridionalis_chr07	22412500	22472500
O. meridionalis	meridionalis_boundary_995	meridionalis_chr07	22557500	22562500
O. meridionalis	meridionalis_boundary_996	meridionalis_chr07	22637500	22642500
O. meridionalis	meridionalis_boundary_997	meridionalis_chr07	22707500	22747500
O. meridionalis	meridionalis_boundary_998	meridionalis_chr07	22787500	22792500
O. meridionalis	meridionalis_boundary_999	meridionalis_chr07	22857500	22862500
O. meridionalis	meridionalis_boundary_1000	meridionalis_chr07	22982500	22987500
O. meridionalis	meridionalis_boundary_1001	meridionalis_chr07	23047500	23052500
O. meridionalis	meridionalis_boundary_1002	meridionalis_chr07	23097500	23102500
O. meridionalis	meridionalis_boundary_1003	meridionalis_chr07	23172500	23177500
O. meridionalis	meridionalis_boundary_1004	meridionalis_chr07	23237500	23277500
O. meridionalis	meridionalis_boundary_1005	meridionalis_chr07	23327500	23357500
O. meridionalis	meridionalis_boundary_1006	meridionalis_chr07	23422500	23427500
O. meridionalis	meridionalis_boundary_1007	meridionalis_chr07	23472500	23477500
O. meridionalis	meridionalis_boundary_1008	meridionalis_chr07	23642500	23647500
O. meridionalis	meridionalis_boundary_1009	meridionalis_chr07	23697500	23732500
O. meridionalis	meridionalis_boundary_1010	meridionalis_chr07	24452500	24492500
O. meridionalis	meridionalis_boundary_1011	meridionalis_chr07	24937500	24942500
O. meridionalis	meridionalis_boundary_1012	meridionalis_chr07	25007500	25012500
O. meridionalis	meridionalis_boundary_1013	meridionalis_chr07	25167500	25172500
O. meridionalis	meridionalis_boundary_1014	meridionalis_chr07	25282500	25287500
O. meridionalis	meridionalis_boundary_1015	meridionalis_chr07	25372500	25377500
O. meridionalis	meridionalis_boundary_1016	meridionalis_chr07	25512500	25517500

O. meridionalis	meridionalis_boundary_1017	meridionalis_chr07	25827500	25832500
O. meridionalis	meridionalis_boundary_1018	meridionalis_chr07	25882500	25887500
O. meridionalis	meridionalis_boundary_1019	meridionalis_chr07	26552500	26557500
O. meridionalis	meridionalis_boundary_1020	meridionalis_chr07	26637500	26642500
O. meridionalis	meridionalis_boundary_1021	meridionalis_chr07	26972500	26977500
O. meridionalis	meridionalis_boundary_1022	meridionalis_chr07	27087500	27092500
O. meridionalis	meridionalis_boundary_1023	meridionalis_chr07	27722500	27727500
O. meridionalis	meridionalis_boundary_1024	meridionalis_chr07	27822500	27827500
O. meridionalis	meridionalis_boundary_1025	meridionalis_chr07	28547500	28617500
O. meridionalis	meridionalis_boundary_1026	meridionalis_chr07	28912500	28917500
O. meridionalis	meridionalis_boundary_1027	meridionalis_chr07	28972500	28977500
O. meridionalis	meridionalis_boundary_1028	meridionalis_chr07	29202500	29242500
O. meridionalis	meridionalis_boundary_1029	meridionalis_chr07	29777500	29782500
O. meridionalis	meridionalis_boundary_1030	meridionalis_chr07	29827500	29832500
O. meridionalis	meridionalis_boundary_1031	meridionalis_chr07	30217500	30222500
O. meridionalis	meridionalis_boundary_1032	meridionalis_chr07	30292500	30377500
O. meridionalis	meridionalis_boundary_1033	meridionalis_chr08	262500	267500
O. meridionalis	meridionalis_boundary_1034	meridionalis_chr08	437500	442500
O. meridionalis	meridionalis_boundary_1035	meridionalis_chr08	687500	692500
O. meridionalis	meridionalis_boundary_1036	meridionalis_chr08	867500	872500
O. meridionalis	meridionalis_boundary_1037	meridionalis_chr08	1347500	1352500
O. meridionalis	meridionalis_boundary_1038	meridionalis_chr08	1417500	1422500
O. meridionalis	meridionalis_boundary_1039	meridionalis_chr08	1722500	1727500
O. meridionalis	meridionalis_boundary_1040	meridionalis_chr08	1817500	1822500
O. meridionalis	meridionalis_boundary_1041	meridionalis_chr08	2007500	2012500
O. meridionalis	meridionalis_boundary_1042	meridionalis_chr08	2067500	2072500
O. meridionalis	meridionalis_boundary_1043	meridionalis_chr08	2877500	2882500
O. meridionalis	meridionalis_boundary_1044	meridionalis_chr08	2942500	2947500
O. meridionalis	meridionalis_boundary_1045	meridionalis_chr08	2992500	2997500
O. meridionalis	meridionalis_boundary_1046	meridionalis_chr08	3137500	3142500
O. meridionalis	meridionalis_boundary_1047	meridionalis_chr08	3197500	3202500
O. meridionalis	meridionalis_boundary_1048	meridionalis_chr08	3257500	3262500

O. meridionalis	meridionalis_boundary_1049	meridionalis_chr08	3467500	3472500
O. meridionalis	meridionalis_boundary_1050	meridionalis_chr08	3557500	3562500
O. meridionalis	meridionalis_boundary_1051	meridionalis_chr08	3622500	3627500
O. meridionalis	meridionalis_boundary_1052	meridionalis_chr08	3822500	3827500
O. meridionalis	meridionalis_boundary_1053	meridionalis_chr08	3867500	3872500
O. meridionalis	meridionalis_boundary_1054	meridionalis_chr08	3917500	3922500
O. meridionalis	meridionalis_boundary_1055	meridionalis_chr08	4352500	4357500
O. meridionalis	meridionalis_boundary_1056	meridionalis_chr08	4397500	4402500
O. meridionalis	meridionalis_boundary_1057	meridionalis_chr08	5007500	5012500
O. meridionalis	meridionalis_boundary_1058	meridionalis_chr08	5217500	5222500
O. meridionalis	meridionalis_boundary_1059	meridionalis_chr08	5682500	5687500
O. meridionalis	meridionalis_boundary_1060	meridionalis_chr08	5792500	5797500
O. meridionalis	meridionalis_boundary_1061	meridionalis_chr08	5867500	5872500
O. meridionalis	meridionalis_boundary_1062	meridionalis_chr08	6097500	6102500
O. meridionalis	meridionalis_boundary_1063	meridionalis_chr08	6162500	6167500
O. meridionalis	meridionalis_boundary_1064	meridionalis_chr08	6232500	6237500
O. meridionalis	meridionalis_boundary_1065	meridionalis_chr08	6287500	6292500
O. meridionalis	meridionalis_boundary_1066	meridionalis_chr08	6342500	6347500
O. meridionalis	meridionalis_boundary_1067	meridionalis_chr08	7712500	7717500
O. meridionalis	meridionalis_boundary_1068	meridionalis_chr08	7762500	7767500
O. meridionalis	meridionalis_boundary_1069	meridionalis_chr08	8037500	8067500
O. meridionalis	meridionalis_boundary_1070	meridionalis_chr08	8122500	8127500
O. meridionalis	meridionalis_boundary_1071	meridionalis_chr08	8307500	8312500
O. meridionalis	meridionalis_boundary_1072	meridionalis_chr08	8377500	8382500
O. meridionalis	meridionalis_boundary_1073	meridionalis_chr08	8452500	8457500
O. meridionalis	meridionalis_boundary_1074	meridionalis_chr08	9352500	9357500
O. meridionalis	meridionalis_boundary_1075	meridionalis_chr08	9402500	9407500
O. meridionalis	meridionalis_boundary_1076	meridionalis_chr08	9612500	9617500
O. meridionalis	meridionalis_boundary_1077	meridionalis_chr08	9847500	9852500
O. meridionalis	meridionalis_boundary_1078	meridionalis_chr08	9912500	9917500
O. meridionalis	meridionalis_boundary_1079	meridionalis_chr08	10092500	10097500
O. meridionalis	meridionalis_boundary_1080	meridionalis_chr08	10272500	10277500

O. meridionalis	meridionalis_boundary_1081	meridionalis_chr08	11142500	11217500
O. meridionalis	meridionalis_boundary_1082	meridionalis_chr08	11382500	11387500
O. meridionalis	meridionalis_boundary_1083	meridionalis_chr08	11452500	11457500
O. meridionalis	meridionalis_boundary_1084	meridionalis_chr08	11522500	11527500
O. meridionalis	meridionalis_boundary_1085	meridionalis_chr08	11577500	11582500
O. meridionalis	meridionalis_boundary_1086	meridionalis_chr08	12182500	12187500
O. meridionalis	meridionalis_boundary_1087	meridionalis_chr08	12232500	12237500
O. meridionalis	meridionalis_boundary_1088	meridionalis_chr08	13147500	13152500
O. meridionalis	meridionalis_boundary_1089	meridionalis_chr08	13207500	13212500
O. meridionalis	meridionalis_boundary_1090	meridionalis_chr08	13787500	13792500
O. meridionalis	meridionalis_boundary_1091	meridionalis_chr08	13857500	13862500
O. meridionalis	meridionalis_boundary_1092	meridionalis_chr08	15267500	15272500
O. meridionalis	meridionalis_boundary_1093	meridionalis_chr08	15317500	15322500
O. meridionalis	meridionalis_boundary_1094	meridionalis_chr08	15497500	15502500
O. meridionalis	meridionalis_boundary_1095	meridionalis_chr08	15592500	15597500
O. meridionalis	meridionalis_boundary_1096	meridionalis_chr08	15742500	15747500
O. meridionalis	meridionalis_boundary_1097	meridionalis_chr08	16277500	16282500
O. meridionalis	meridionalis_boundary_1098	meridionalis_chr08	16322500	16327500
O. meridionalis	meridionalis_boundary_1099	meridionalis_chr08	16417500	16422500
O. meridionalis	meridionalis_boundary_1100	meridionalis_chr08	16507500	16512500
O. meridionalis	meridionalis_boundary_1101	meridionalis_chr08	16647500	16652500
O. meridionalis	meridionalis_boundary_1102	meridionalis_chr08	17212500	17217500
O. meridionalis	meridionalis_boundary_1103	meridionalis_chr08	17377500	17382500
O. meridionalis	meridionalis_boundary_1104	meridionalis_chr08	17422500	17467500
O. meridionalis	meridionalis_boundary_1105	meridionalis_chr08	17582500	17587500
O. meridionalis	meridionalis_boundary_1106	meridionalis_chr08	18197500	18202500
O. meridionalis	meridionalis_boundary_1107	meridionalis_chr08	18257500	18262500
O. meridionalis	meridionalis_boundary_1108	meridionalis_chr08	18302500	18307500
O. meridionalis	meridionalis_boundary_1109	meridionalis_chr08	18352500	18357500
O. meridionalis	meridionalis_boundary_1110	meridionalis_chr08	18572500	18577500
O. meridionalis	meridionalis_boundary_1111	meridionalis_chr08	18727500	18732500
O. meridionalis	meridionalis_boundary_1112	meridionalis_chr08	18872500	18877500

O. meridionalis	meridionalis_boundary_1113	meridionalis_chr08	19572500	19577500
O. meridionalis	meridionalis_boundary_1114	meridionalis_chr08	19707500	19712500
O. meridionalis	meridionalis_boundary_1115	meridionalis_chr08	19772500	19777500
O. meridionalis	meridionalis_boundary_1116	meridionalis_chr08	20402500	20407500
O. meridionalis	meridionalis_boundary_1117	meridionalis_chr08	20457500	20462500
O. meridionalis	meridionalis_boundary_1118	meridionalis_chr08	20517500	20522500
O. meridionalis	meridionalis_boundary_1119	meridionalis_chr08	20607500	20612500
O. meridionalis	meridionalis_boundary_1120	meridionalis_chr08	20937500	20982500
O. meridionalis	meridionalis_boundary_1121	meridionalis_chr08	21022500	21027500
O. meridionalis	meridionalis_boundary_1122	meridionalis_chr08	21217500	21222500
O. meridionalis	meridionalis_boundary_1123	meridionalis_chr08	21332500	21337500
O. meridionalis	meridionalis_boundary_1124	meridionalis_chr08	22032500	22037500
O. meridionalis	meridionalis_boundary_1125	meridionalis_chr08	22092500	22097500
O. meridionalis	meridionalis_boundary_1126	meridionalis_chr08	22147500	22152500
O. meridionalis	meridionalis_boundary_1127	meridionalis_chr08	22357500	22362500
O. meridionalis	meridionalis_boundary_1128	meridionalis_chr08	22407500	22412500
O. meridionalis	meridionalis_boundary_1129	meridionalis_chr08	22482500	22517500
O. meridionalis	meridionalis_boundary_1130	meridionalis_chr08	22787500	22792500
O. meridionalis	meridionalis_boundary_1131	meridionalis_chr08	22857500	22862500
O. meridionalis	meridionalis_boundary_1132	meridionalis_chr08	23267500	23272500
O. meridionalis	meridionalis_boundary_1133	meridionalis_chr08	23360000	23365000
O. meridionalis	meridionalis_boundary_1134	meridionalis_chr08	23592500	23597500
O. meridionalis	meridionalis_boundary_1135	meridionalis_chr08	23647500	23652500
O. meridionalis	meridionalis_boundary_1136	meridionalis_chr08	23777500	23862500
O. meridionalis	meridionalis_boundary_1137	meridionalis_chr08	23907500	23912500
O. meridionalis	meridionalis_boundary_1138	meridionalis_chr08	24317500	24322500
O. meridionalis	meridionalis_boundary_1139	meridionalis_chr08	24422500	24457500
O. meridionalis	meridionalis_boundary_1140	meridionalis_chr08	24662500	24667500
O. meridionalis	meridionalis_boundary_1141	meridionalis_chr08	24882500	24887500
O. meridionalis	meridionalis_boundary_1142	meridionalis_chr08	24932500	24972500
O. meridionalis	meridionalis_boundary_1143	meridionalis_chr08	25352500	25357500
O. meridionalis	meridionalis_boundary_1144	meridionalis_chr08	25422500	25427500

O. meridionalis	meridionalis_boundary_1145	meridionalis_chr08	25667500	25672500
O. meridionalis	meridionalis_boundary_1146	meridionalis_chr08	25737500	25742500
O. meridionalis	meridionalis_boundary_1147	meridionalis_chr08	25797500	25802500
O. meridionalis	meridionalis_boundary_1148	meridionalis_chr08	25862500	25867500
O. meridionalis	meridionalis_boundary_1149	meridionalis_chr08	25967500	26012500
O. meridionalis	meridionalis_boundary_1150	meridionalis_chr08	26087500	26132500
O. meridionalis	meridionalis_boundary_1151	meridionalis_chr08	26172500	26177500
O. meridionalis	meridionalis_boundary_1152	meridionalis_chr08	26247500	26327500
O. meridionalis	meridionalis_boundary_1153	meridionalis_chr08	26432500	26437500
O. meridionalis	meridionalis_boundary_1154	meridionalis_chr08	26502500	26507500
O. meridionalis	meridionalis_boundary_1155	meridionalis_chr08	26552500	26557500
O. meridionalis	meridionalis_boundary_1156	meridionalis_chr08	26617500	26662500
O. meridionalis	meridionalis_boundary_1157	meridionalis_chr08	26852500	26892500
O. meridionalis	meridionalis_boundary_1158	meridionalis_chr08	26952500	26957500
O. meridionalis	meridionalis_boundary_1159	meridionalis_chr08	27517500	27522500
O. meridionalis	meridionalis_boundary_1160	meridionalis_chr08	27572500	27577500
O. meridionalis	meridionalis_boundary_1161	meridionalis_chr08	27737500	27742500
O. meridionalis	meridionalis_boundary_1162	meridionalis_chr08	27797500	27802500
O. meridionalis	meridionalis_boundary_1163	meridionalis_chr08	28472500	28477500
O. meridionalis	meridionalis_boundary_1164	meridionalis_chr08	28552500	28557500
O. meridionalis	meridionalis_boundary_1165	meridionalis_chr08	28607500	28612500
O. meridionalis	meridionalis_boundary_1166	meridionalis_chr09	432500	437500
O. meridionalis	meridionalis_boundary_1167	meridionalis_chr09	492500	497500
O. meridionalis	meridionalis_boundary_1168	meridionalis_chr09	827500	832500
O. meridionalis	meridionalis_boundary_1169	meridionalis_chr09	942500	947500
O. meridionalis	meridionalis_boundary_1170	meridionalis_chr09	1137500	1142500
O. meridionalis	meridionalis_boundary_1171	meridionalis_chr09	1232500	1237500
O. meridionalis	meridionalis_boundary_1172	meridionalis_chr09	1907500	1947500
O. meridionalis	meridionalis_boundary_1173	meridionalis_chr09	2007500	2012500
O. meridionalis	meridionalis_boundary_1174	meridionalis_chr09	2077500	2082500
O. meridionalis	meridionalis_boundary_1175	meridionalis_chr09	2157500	2162500
O. meridionalis	meridionalis_boundary_1176	meridionalis_chr09	3102500	3107500

O. meridionalis	meridionalis_boundary_1177	meridionalis_chr09	3252500	3257500
O. meridionalis	meridionalis_boundary_1178	meridionalis_chr09	4027500	4032500
O. meridionalis	meridionalis_boundary_1179	meridionalis_chr09	4082500	4087500
O. meridionalis	meridionalis_boundary_1180	meridionalis_chr09	4487500	4492500
O. meridionalis	meridionalis_boundary_1181	meridionalis_chr09	4562500	4567500
O. meridionalis	meridionalis_boundary_1182	meridionalis_chr09	4812500	4817500
O. meridionalis	meridionalis_boundary_1183	meridionalis_chr09	5337500	5342500
O. meridionalis	meridionalis_boundary_1184	meridionalis_chr09	5447500	5452500
O. meridionalis	meridionalis_boundary_1185	meridionalis_chr09	5517500	5522500
O. meridionalis	meridionalis_boundary_1186	meridionalis_chr09	6017500	6022500
O. meridionalis	meridionalis_boundary_1187	meridionalis_chr09	6062500	6067500
O. meridionalis	meridionalis_boundary_1188	meridionalis_chr09	8732500	8737500
O. meridionalis	meridionalis_boundary_1189	meridionalis_chr09	8782500	8787500
O. meridionalis	meridionalis_boundary_1190	meridionalis_chr09	8852500	8857500
O. meridionalis	meridionalis_boundary_1191	meridionalis_chr09	8932500	8937500
O. meridionalis	meridionalis_boundary_1192	meridionalis_chr09	11972500	11977500
O. meridionalis	meridionalis_boundary_1193	meridionalis_chr09	12032500	12072500
O. meridionalis	meridionalis_boundary_1194	meridionalis_chr09	12132500	12137500
O. meridionalis	meridionalis_boundary_1195	meridionalis_chr09	12222500	12227500
O. meridionalis	meridionalis_boundary_1196	meridionalis_chr09	12302500	12307500
O. meridionalis	meridionalis_boundary_1197	meridionalis_chr09	12557500	12562500
O. meridionalis	meridionalis_boundary_1198	meridionalis_chr09	12652500	12692500
O. meridionalis	meridionalis_boundary_1199	meridionalis_chr09	12837500	12842500
O. meridionalis	meridionalis_boundary_1200	meridionalis_chr09	13002500	13032500
O. meridionalis	meridionalis_boundary_1201	meridionalis_chr09	13077500	13082500
O. meridionalis	meridionalis_boundary_1202	meridionalis_chr09	13222500	13227500
O. meridionalis	meridionalis_boundary_1203	meridionalis_chr09	13352500	13357500
O. meridionalis	meridionalis_boundary_1204	meridionalis_chr09	13422500	13427500
O. meridionalis	meridionalis_boundary_1205	meridionalis_chr09	13752500	13757500
O. meridionalis	meridionalis_boundary_1206	meridionalis_chr09	13807500	13812500
O. meridionalis	meridionalis_boundary_1207	meridionalis_chr09	13907500	13912500
O. meridionalis	meridionalis_boundary_1208	meridionalis_chr09	14017500	14022500

O. meridionalis	meridionalis_boundary_1209	meridionalis_chr09	14077500	14142500
O. meridionalis	meridionalis_boundary_1210	meridionalis_chr09	14457500	14462500
O. meridionalis	meridionalis_boundary_1211	meridionalis_chr09	14537500	14542500
O. meridionalis	meridionalis_boundary_1212	meridionalis_chr09	14722500	14757500
O. meridionalis	meridionalis_boundary_1213	meridionalis_chr09	14937500	14967500
O. meridionalis	meridionalis_boundary_1214	meridionalis_chr09	15012500	15017500
O. meridionalis	meridionalis_boundary_1215	meridionalis_chr09	15327500	15332500
O. meridionalis	meridionalis_boundary_1216	meridionalis_chr09	15402500	15407500
O. meridionalis	meridionalis_boundary_1217	meridionalis_chr09	15577500	15582500
O. meridionalis	meridionalis_boundary_1218	meridionalis_chr09	15647500	15652500
O. meridionalis	meridionalis_boundary_1219	meridionalis_chr09	15702500	15707500
O. meridionalis	meridionalis_boundary_1220	meridionalis_chr09	15817500	15822500
O. meridionalis	meridionalis_boundary_1221	meridionalis_chr09	15877500	15882500
O. meridionalis	meridionalis_boundary_1222	meridionalis_chr09	16132500	16177500
O. meridionalis	meridionalis_boundary_1223	meridionalis_chr09	16247500	16252500
O. meridionalis	meridionalis_boundary_1224	meridionalis_chr09	16332500	16377500
O. meridionalis	meridionalis_boundary_1225	meridionalis_chr09	16472500	16477500
O. meridionalis	meridionalis_boundary_1226	meridionalis_chr09	16542500	16547500
O. meridionalis	meridionalis_boundary_1227	meridionalis_chr09	16702500	16707500
O. meridionalis	meridionalis_boundary_1228	meridionalis_chr09	16767500	16772500
O. meridionalis	meridionalis_boundary_1229	meridionalis_chr09	16847500	16852500
O. meridionalis	meridionalis_boundary_1230	meridionalis_chr09	17172500	17177500
O. meridionalis	meridionalis_boundary_1231	meridionalis_chr09	17227500	17232500
O. meridionalis	meridionalis_boundary_1232	meridionalis_chr09	17287500	17292500
O. meridionalis	meridionalis_boundary_1233	meridionalis_chr09	17332500	17337500
O. meridionalis	meridionalis_boundary_1234	meridionalis_chr09	18032500	18037500
O. meridionalis	meridionalis_boundary_1235	meridionalis_chr09	18147500	18152500
O. meridionalis	meridionalis_boundary_1236	meridionalis_chr09	18202500	18207500
O. meridionalis	meridionalis_boundary_1237	meridionalis_chr09	19252500	19257500
O. meridionalis	meridionalis_boundary_1238	meridionalis_chr09	19362500	19367500
O. meridionalis	meridionalis_boundary_1239	meridionalis_chr09	19842500	19847500
O. meridionalis	meridionalis_boundary_1240	meridionalis_chr09	19972500	20017500

O. meridionalis	meridionalis_boundary_1241	meridionalis_chr09	20072500	20077500
O. meridionalis	meridionalis_boundary_1242	meridionalis_chr09	20122500	20127500
O. meridionalis	meridionalis_boundary_1243	meridionalis_chr09	20397500	20402500
O. meridionalis	meridionalis_boundary_1244	meridionalis_chr09	20507500	20512500
O. meridionalis	meridionalis_boundary_1245	meridionalis_chr09	20570000	20612500
O. meridionalis	meridionalis_boundary_1246	meridionalis_chr09	20667500	20672500
O. meridionalis	meridionalis_boundary_1247	meridionalis_chr09	21017500	21022500
O. meridionalis	meridionalis_boundary_1248	meridionalis_chr09	21072500	21077500
O. meridionalis	meridionalis_boundary_1249	meridionalis_chr09	21227500	21257500
O. meridionalis	meridionalis_boundary_1250	meridionalis_chr09	21312500	21317500
O. meridionalis	meridionalis_boundary_1251	meridionalis_chr09	21532500	21537500
O. meridionalis	meridionalis_boundary_1252	meridionalis_chr09	21577500	21612500
O. meridionalis	meridionalis_boundary_1253	meridionalis_chr09	21677500	21682500
O. meridionalis	meridionalis_boundary_1254	meridionalis_chr09	21732500	21737500
O. meridionalis	meridionalis_boundary_1255	meridionalis_chr09	21777500	21782500
O. meridionalis	meridionalis_boundary_1256	meridionalis_chr09	21977500	21982500
O. meridionalis	meridionalis_boundary_1257	meridionalis_chr09	22197500	22262500
O. meridionalis	meridionalis_boundary_1258	meridionalis_chr09	22307500	22312500
O. meridionalis	meridionalis_boundary_1259	meridionalis_chr09	22407500	22412500
O. meridionalis	meridionalis_boundary_1260	meridionalis_chr09	22452500	22457500
O. meridionalis	meridionalis_boundary_1261	meridionalis_chr09	22542500	22547500
O. meridionalis	meridionalis_boundary_1262	meridionalis_chr09	22587500	22592500
O. meridionalis	meridionalis_boundary_1263	meridionalis_chr09	22642500	22647500
O. meridionalis	meridionalis_boundary_1264	meridionalis_chr10	2052500	2057500
O. meridionalis	meridionalis_boundary_1265	meridionalis_chr10	2107500	2112500
O. meridionalis	meridionalis_boundary_1266	meridionalis_chr10	2252500	2257500
O. meridionalis	meridionalis_boundary_1267	meridionalis_chr10	2357500	2362500
O. meridionalis	meridionalis_boundary_1268	meridionalis_chr10	3247500	3252500
O. meridionalis	meridionalis_boundary_1269	meridionalis_chr10	3322500	3327500
O. meridionalis	meridionalis_boundary_1270	meridionalis_chr10	3442500	3487500
O. meridionalis	meridionalis_boundary_1271	meridionalis_chr10	3587500	3592500
O. meridionalis	meridionalis_boundary_1272	meridionalis_chr10	3692500	3697500

O. meridionalis	meridionalis_boundary_1273	meridionalis_chr10	4827500	4832500
O. meridionalis	meridionalis_boundary_1274	meridionalis_chr10	4927500	4962500
O. meridionalis	meridionalis_boundary_1275	meridionalis_chr10	5037500	5042500
O. meridionalis	meridionalis_boundary_1276	meridionalis_chr10	5127500	5132500
O. meridionalis	meridionalis_boundary_1277	meridionalis_chr10	5182500	5187500
O. meridionalis	meridionalis_boundary_1278	meridionalis_chr10	5647500	5652500
O. meridionalis	meridionalis_boundary_1279	meridionalis_chr10	5722500	5727500
O. meridionalis	meridionalis_boundary_1280	meridionalis_chr10	6652500	6657500
O. meridionalis	meridionalis_boundary_1281	meridionalis_chr10	6737500	6742500
O. meridionalis	meridionalis_boundary_1282	meridionalis_chr10	6805000	6810000
O. meridionalis	meridionalis_boundary_1283	meridionalis_chr10	7947500	7952500
O. meridionalis	meridionalis_boundary_1284	meridionalis_chr10	8067500	8072500
O. meridionalis	meridionalis_boundary_1285	meridionalis_chr10	8392500	8397500
O. meridionalis	meridionalis_boundary_1286	meridionalis_chr10	8477500	8482500
O. meridionalis	meridionalis_boundary_1287	meridionalis_chr10	9002500	9007500
O. meridionalis	meridionalis_boundary_1288	meridionalis_chr10	9107500	9112500
O. meridionalis	meridionalis_boundary_1289	meridionalis_chr10	11092500	11097500
O. meridionalis	meridionalis_boundary_1290	meridionalis_chr10	11232500	11237500
O. meridionalis	meridionalis_boundary_1291	meridionalis_chr10	11367500	11372500
O. meridionalis	meridionalis_boundary_1292	meridionalis_chr10	11867500	11872500
O. meridionalis	meridionalis_boundary_1293	meridionalis_chr10	12082500	12087500
O. meridionalis	meridionalis_boundary_1294	meridionalis_chr10	12357500	12362500
O. meridionalis	meridionalis_boundary_1295	meridionalis_chr10	12457500	12462500
O. meridionalis	meridionalis_boundary_1296	meridionalis_chr10	12812500	12817500
O. meridionalis	meridionalis_boundary_1297	meridionalis_chr10	12867500	12872500
O. meridionalis	meridionalis_boundary_1298	meridionalis_chr10	12912500	12917500
O. meridionalis	meridionalis_boundary_1299	meridionalis_chr10	13832500	13837500
O. meridionalis	meridionalis_boundary_1300	meridionalis_chr10	13892500	13897500
O. meridionalis	meridionalis_boundary_1301	meridionalis_chr10	14297500	14302500
O. meridionalis	meridionalis_boundary_1302	meridionalis_chr10	14377500	14382500
O. meridionalis	meridionalis_boundary_1303	meridionalis_chr10	15247500	15252500
O. meridionalis	meridionalis_boundary_1304	meridionalis_chr10	15322500	15327500

O. meridionalis	meridionalis_boundary_1305	meridionalis_chr10	15447500	15452500
O. meridionalis	meridionalis_boundary_1306	meridionalis_chr10	15507500	15512500
O. meridionalis	meridionalis_boundary_1307	meridionalis_chr10	15642500	15782500
O. meridionalis	meridionalis_boundary_1308	meridionalis_chr10	15857500	15862500
O. meridionalis	meridionalis_boundary_1309	meridionalis_chr10	15992500	15997500
O. meridionalis	meridionalis_boundary_1310	meridionalis_chr10	16217500	16222500
O. meridionalis	meridionalis_boundary_1311	meridionalis_chr10	16332500	16377500
O. meridionalis	meridionalis_boundary_1312	meridionalis_chr10	17052500	17097500
O. meridionalis	meridionalis_boundary_1313	meridionalis_chr10	17167500	17172500
O. meridionalis	meridionalis_boundary_1314	meridionalis_chr10	17232500	17237500
O. meridionalis	meridionalis_boundary_1315	meridionalis_chr10	17377500	17417500
O. meridionalis	meridionalis_boundary_1316	meridionalis_chr10	17602500	17607500
O. meridionalis	meridionalis_boundary_1317	meridionalis_chr10	17657500	17662500
O. meridionalis	meridionalis_boundary_1318	meridionalis_chr10	17747500	17752500
O. meridionalis	meridionalis_boundary_1319	meridionalis_chr10	17837500	17842500
O. meridionalis	meridionalis_boundary_1320	meridionalis_chr10	17922500	17967500
O. meridionalis	meridionalis_boundary_1321	meridionalis_chr10	18517500	18562500
O. meridionalis	meridionalis_boundary_1322	meridionalis_chr10	18612500	18617500
O. meridionalis	meridionalis_boundary_1323	meridionalis_chr10	18697500	18737500
O. meridionalis	meridionalis_boundary_1324	meridionalis_chr10	18807500	18812500
O. meridionalis	meridionalis_boundary_1325	meridionalis_chr10	18857500	18862500
O. meridionalis	meridionalis_boundary_1326	meridionalis_chr10	18917500	18922500
O. meridionalis	meridionalis_boundary_1327	meridionalis_chr10	18972500	19007500
O. meridionalis	meridionalis_boundary_1328	meridionalis_chr10	19437500	19442500
O. meridionalis	meridionalis_boundary_1329	meridionalis_chr10	19507500	19512500
O. meridionalis	meridionalis_boundary_1330	meridionalis_chr10	19562500	19567500
O. meridionalis	meridionalis_boundary_1331	meridionalis_chr10	19612500	19617500
O. meridionalis	meridionalis_boundary_1332	meridionalis_chr10	19670000	19675000
O. meridionalis	meridionalis_boundary_1333	meridionalis_chr10	19837500	19842500
O. meridionalis	meridionalis_boundary_1334	meridionalis_chr10	19927500	19932500
O. meridionalis	meridionalis_boundary_1335	meridionalis_chr10	20062500	20067500
O. meridionalis	meridionalis_boundary_1336	meridionalis_chr10	20147500	20187500

O. meridionalis	meridionalis_boundary_1337	meridionalis_chr10	20267500	20272500
O. meridionalis	meridionalis_boundary_1338	meridionalis_chr10	20517500	20522500
O. meridionalis	meridionalis_boundary_1339	meridionalis_chr10	20597500	20602500
O. meridionalis	meridionalis_boundary_1340	meridionalis_chr10	20692500	20697500
O. meridionalis	meridionalis_boundary_1341	meridionalis_chr10	20737500	20807500
O. meridionalis	meridionalis_boundary_1342	meridionalis_chr10	21517500	21522500
O. meridionalis	meridionalis_boundary_1343	meridionalis_chr10	21577500	21582500
O. meridionalis	meridionalis_boundary_1344	meridionalis_chr10	21912500	21917500
O. meridionalis	meridionalis_boundary_1345	meridionalis_chr10	21997500	22002500
O. meridionalis	meridionalis_boundary_1346	meridionalis_chr10	22072500	22077500
O. meridionalis	meridionalis_boundary_1347	meridionalis_chr10	22122500	22127500
O. meridionalis	meridionalis_boundary_1348	meridionalis_chr10	23097500	23102500
O. meridionalis	meridionalis_boundary_1349	meridionalis_chr10	23162500	23192500
O. meridionalis	meridionalis_boundary_1350	meridionalis_chr10	23262500	23267500
O. meridionalis	meridionalis_boundary_1351	meridionalis_chr10	23422500	23427500
O. meridionalis	meridionalis_boundary_1352	meridionalis_chr10	23502500	23507500
O. meridionalis	meridionalis_boundary_1353	meridionalis_chr10	23842500	23847500
O. meridionalis	meridionalis_boundary_1354	meridionalis_chr10	23887500	23892500
O. meridionalis	meridionalis_boundary_1355	meridionalis_chr11	2167500	2172500
O. meridionalis	meridionalis_boundary_1356	meridionalis_chr11	2232500	2237500
O. meridionalis	meridionalis_boundary_1357	meridionalis_chr11	2557500	2602500
O. meridionalis	meridionalis_boundary_1358	meridionalis_chr11	2647500	2652500
O. meridionalis	meridionalis_boundary_1359	meridionalis_chr11	3037500	3122500
O. meridionalis	meridionalis_boundary_1360	meridionalis_chr11	3217500	3247500
O. meridionalis	meridionalis_boundary_1361	meridionalis_chr11	3332500	3337500
O. meridionalis	meridionalis_boundary_1362	meridionalis_chr11	3457500	3462500
O. meridionalis	meridionalis_boundary_1363	meridionalis_chr11	3517500	3552500
O. meridionalis	meridionalis_boundary_1364	meridionalis_chr11	3717500	3722500
O. meridionalis	meridionalis_boundary_1365	meridionalis_chr11	3822500	3827500
O. meridionalis	meridionalis_boundary_1366	meridionalis_chr11	3892500	3897500
O. meridionalis	meridionalis_boundary_1367	meridionalis_chr11	4052500	4057500
O. meridionalis	meridionalis_boundary_1368	meridionalis_chr11	4122500	4127500

O. meridionalis	meridionalis_boundary_1369	meridionalis_chr11	4182500	4187500
O. meridionalis	meridionalis_boundary_1370	meridionalis_chr11	4322500	4327500
O. meridionalis	meridionalis_boundary_1371	meridionalis_chr11	5037500	5042500
O. meridionalis	meridionalis_boundary_1372	meridionalis_chr11	5107500	5152500
O. meridionalis	meridionalis_boundary_1373	meridionalis_chr11	5192500	5197500
O. meridionalis	meridionalis_boundary_1374	meridionalis_chr11	5472500	5477500
O. meridionalis	meridionalis_boundary_1375	meridionalis_chr11	5527500	5567500
O. meridionalis	meridionalis_boundary_1376	meridionalis_chr11	5812500	5817500
O. meridionalis	meridionalis_boundary_1377	meridionalis_chr11	5932500	5937500
O. meridionalis	meridionalis_boundary_1378	meridionalis_chr11	6137500	6142500
O. meridionalis	meridionalis_boundary_1379	meridionalis_chr11	6682500	6722500
O. meridionalis	meridionalis_boundary_1380	meridionalis_chr11	6842500	6847500
O. meridionalis	meridionalis_boundary_1381	meridionalis_chr11	6895000	6900000
O. meridionalis	meridionalis_boundary_1382	meridionalis_chr11	7467500	7472500
O. meridionalis	meridionalis_boundary_1383	meridionalis_chr11	7552500	7557500
O. meridionalis	meridionalis_boundary_1384	meridionalis_chr11	7612500	7617500
O. meridionalis	meridionalis_boundary_1385	meridionalis_chr11	7727500	7732500
O. meridionalis	meridionalis_boundary_1386	meridionalis_chr11	7852500	7857500
O. meridionalis	meridionalis_boundary_1387	meridionalis_chr11	8197500	8202500
O. meridionalis	meridionalis_boundary_1388	meridionalis_chr11	8257500	8332500
O. meridionalis	meridionalis_boundary_1389	meridionalis_chr11	8992500	8997500
O. meridionalis	meridionalis_boundary_1390	meridionalis_chr11	9102500	9107500
O. meridionalis	meridionalis_boundary_1391	meridionalis_chr11	9272500	9277500
O. meridionalis	meridionalis_boundary_1392	meridionalis_chr11	9327500	9332500
O. meridionalis	meridionalis_boundary_1393	meridionalis_chr11	10802500	10807500
O. meridionalis	meridionalis_boundary_1394	meridionalis_chr11	10887500	10927500
O. meridionalis	meridionalis_boundary_1395	meridionalis_chr11	11587500	11592500
O. meridionalis	meridionalis_boundary_1396	meridionalis_chr11	11752500	11757500
O. meridionalis	meridionalis_boundary_1397	meridionalis_chr11	13540000	13545000
O. meridionalis	meridionalis_boundary_1398	meridionalis_chr11	13612500	13617500
O. meridionalis	meridionalis_boundary_1399	meridionalis_chr11	13962500	13967500
O. meridionalis	meridionalis_boundary_1400	meridionalis_chr11	14017500	14022500

O. meridionalis	meridionalis_boundary_1401	meridionalis_chr11	14082500	14087500
O. meridionalis	meridionalis_boundary_1402	meridionalis_chr11	14357500	14392500
O. meridionalis	meridionalis_boundary_1403	meridionalis_chr11	15737500	15742500
O. meridionalis	meridionalis_boundary_1404	meridionalis_chr11	15872500	15877500
O. meridionalis	meridionalis_boundary_1405	meridionalis_chr11	17487500	17492500
O. meridionalis	meridionalis_boundary_1406	meridionalis_chr11	17552500	17557500
O. meridionalis	meridionalis_boundary_1407	meridionalis_chr11	17637500	17642500
O. meridionalis	meridionalis_boundary_1408	meridionalis_chr11	18407500	18447500
O. meridionalis	meridionalis_boundary_1409	meridionalis_chr11	19197500	19202500
O. meridionalis	meridionalis_boundary_1410	meridionalis_chr11	19277500	19282500
O. meridionalis	meridionalis_boundary_1411	meridionalis_chr11	19402500	19407500
O. meridionalis	meridionalis_boundary_1412	meridionalis_chr11	19447500	19452500
O. meridionalis	meridionalis_boundary_1413	meridionalis_chr11	19517500	19522500
O. meridionalis	meridionalis_boundary_1414	meridionalis_chr11	19592500	19597500
O. meridionalis	meridionalis_boundary_1415	meridionalis_chr11	19697500	19702500
O. meridionalis	meridionalis_boundary_1416	meridionalis_chr11	20115000	20157500
O. meridionalis	meridionalis_boundary_1417	meridionalis_chr11	20207500	20212500
O. meridionalis	meridionalis_boundary_1418	meridionalis_chr11	20562500	20567500
O. meridionalis	meridionalis_boundary_1419	meridionalis_chr11	20637500	20642500
O. meridionalis	meridionalis_boundary_1420	meridionalis_chr11	20922500	20967500
O. meridionalis	meridionalis_boundary_1421	meridionalis_chr11	21197500	21242500
O. meridionalis	meridionalis_boundary_1422	meridionalis_chr11	22832500	22837500
O. meridionalis	meridionalis_boundary_1423	meridionalis_chr11	22927500	22932500
O. meridionalis	meridionalis_boundary_1424	meridionalis_chr11	23047500	23052500
O. meridionalis	meridionalis_boundary_1425	meridionalis_chr11	23097500	23102500
O. meridionalis	meridionalis_boundary_1426	meridionalis_chr11	23187500	23192500
O. meridionalis	meridionalis_boundary_1427	meridionalis_chr11	23657500	23662500
O. meridionalis	meridionalis_boundary_1428	meridionalis_chr11	23707500	23712500
O. meridionalis	meridionalis_boundary_1429	meridionalis_chr11	23997500	24002500
O. meridionalis	meridionalis_boundary_1430	meridionalis_chr11	24042500	24047500
O. meridionalis	meridionalis_boundary_1431	meridionalis_chr11	26417500	26422500
O. meridionalis	meridionalis_boundary_1432	meridionalis_chr11	26527500	26532500

O. meridionalis	meridionalis_boundary_1433	meridionalis_chr11	26847500	26852500
O. meridionalis	meridionalis_boundary_1434	meridionalis_chr11	26907500	26912500
O. meridionalis	meridionalis_boundary_1435	meridionalis_chr11	27222500	27227500
O. meridionalis	meridionalis_boundary_1436	meridionalis_chr11	27337500	27342500
O. meridionalis	meridionalis_boundary_1437	meridionalis_chr11	27837500	27875000
O. meridionalis	meridionalis_boundary_1438	meridionalis_chr11	28087500	28092500
O. meridionalis	meridionalis_boundary_1439	meridionalis_chr11	28157500	28192500
O. meridionalis	meridionalis_boundary_1440	meridionalis_chr11	28257500	28262500
O. meridionalis	meridionalis_boundary_1441	meridionalis_chr11	28312500	28317500
O. meridionalis	meridionalis_boundary_1442	meridionalis_chr11	28372500	28377500
O. meridionalis	meridionalis_boundary_1443	meridionalis_chr11	28422500	28427500
O. meridionalis	meridionalis_boundary_1444	meridionalis_chr11	28487500	28492500
O. meridionalis	meridionalis_boundary_1445	meridionalis_chr12	770000	775000
O. meridionalis	meridionalis_boundary_1446	meridionalis_chr12	822500	827500
O. meridionalis	meridionalis_boundary_1447	meridionalis_chr12	1242500	1247500
O. meridionalis	meridionalis_boundary_1448	meridionalis_chr12	1312500	1317500
O. meridionalis	meridionalis_boundary_1449	meridionalis_chr12	1445000	1492500
O. meridionalis	meridionalis_boundary_1450	meridionalis_chr12	1607500	1612500
O. meridionalis	meridionalis_boundary_1451	meridionalis_chr12	2957500	2997500
O. meridionalis	meridionalis_boundary_1452	meridionalis_chr12	3732500	3737500
O. meridionalis	meridionalis_boundary_1453	meridionalis_chr12	3782500	3787500
O. meridionalis	meridionalis_boundary_1454	meridionalis_chr12	3927500	3932500
O. meridionalis	meridionalis_boundary_1455	meridionalis_chr12	3997500	4002500
O. meridionalis	meridionalis_boundary_1456	meridionalis_chr12	4332500	4337500
O. meridionalis	meridionalis_boundary_1457	meridionalis_chr12	4442500	4447500
O. meridionalis	meridionalis_boundary_1458	meridionalis_chr12	4507500	4512500
O. meridionalis	meridionalis_boundary_1459	meridionalis_chr12	5572500	5577500
O. meridionalis	meridionalis_boundary_1460	meridionalis_chr12	5632500	5637500
O. meridionalis	meridionalis_boundary_1461	meridionalis_chr12	5777500	5782500
O. meridionalis	meridionalis_boundary_1462	meridionalis_chr12	5857500	5862500
O. meridionalis	meridionalis_boundary_1463	meridionalis_chr12	6807500	6812500
O. meridionalis	meridionalis_boundary_1464	meridionalis_chr12	6952500	6957500

O. meridionalis	meridionalis_boundary_1465	meridionalis_chr12	7252500	7300000
O. meridionalis	meridionalis_boundary_1466	meridionalis_chr12	7397500	7437500
O. meridionalis	meridionalis_boundary_1467	meridionalis_chr12	9942500	9947500
O. meridionalis	meridionalis_boundary_1468	meridionalis_chr12	9997500	10002500
O. meridionalis	meridionalis_boundary_1469	meridionalis_chr12	10212500	10217500
O. meridionalis	meridionalis_boundary_1470	meridionalis_chr12	10287500	10292500
O. meridionalis	meridionalis_boundary_1471	meridionalis_chr12	12072500	12117500
O. meridionalis	meridionalis_boundary_1472	meridionalis_chr12	13452500	13457500
O. meridionalis	meridionalis_boundary_1473	meridionalis_chr12	13527500	13532500
O. meridionalis	meridionalis_boundary_1474	meridionalis_chr12	14057500	14092500
O. meridionalis	meridionalis_boundary_1475	meridionalis_chr12	15637500	15642500
O. meridionalis	meridionalis_boundary_1476	meridionalis_chr12	15707500	15712500
O. meridionalis	meridionalis_boundary_1477	meridionalis_chr12	15827500	15832500
O. meridionalis	meridionalis_boundary_1478	meridionalis_chr12	16197500	16202500
O. meridionalis	meridionalis_boundary_1479	meridionalis_chr12	16262500	16267500
O. meridionalis	meridionalis_boundary_1480	meridionalis_chr12	16312500	16317500
O. meridionalis	meridionalis_boundary_1481	meridionalis_chr12	16362500	16367500
O. meridionalis	meridionalis_boundary_1482	meridionalis_chr12	17542500	17547500
O. meridionalis	meridionalis_boundary_1483	meridionalis_chr12	17597500	17602500
O. meridionalis	meridionalis_boundary_1484	meridionalis_chr12	17912500	17917500
O. meridionalis	meridionalis_boundary_1485	meridionalis_chr12	18172500	18177500
O. meridionalis	meridionalis_boundary_1486	meridionalis_chr12	18367500	18372500
O. meridionalis	meridionalis_boundary_1487	meridionalis_chr12	18417500	18452500
O. meridionalis	meridionalis_boundary_1488	meridionalis_chr12	18582500	18587500
O. meridionalis	meridionalis_boundary_1489	meridionalis_chr12	18917500	18922500
O. meridionalis	meridionalis_boundary_1490	meridionalis_chr12	19067500	19072500
O. meridionalis	meridionalis_boundary_1491	meridionalis_chr12	19122500	19127500
O. meridionalis	meridionalis_boundary_1492	meridionalis_chr12	19380000	19385000
O. rufipogon	rufipogon_boundary_0	rufipogon_chr01	87500	132500
O. rufipogon	rufipogon_boundary_1	rufipogon_chr01	187500	192500
O. rufipogon	rufipogon_boundary_2	rufipogon_chr01	262500	267500
O. rufipogon	rufipogon_boundary_3	rufipogon_chr01	482500	487500

O. rufipogon	rufipogon_boundary_4	rufipogon_chr01	537500	542500
O. rufipogon	rufipogon_boundary_5	rufipogon_chr01	1062500	1067500
O. rufipogon	rufipogon_boundary_6	rufipogon_chr01	1117500	1122500
O. rufipogon	rufipogon_boundary_7	rufipogon_chr01	1747500	1752500
O. rufipogon	rufipogon_boundary_8	rufipogon_chr01	1837500	1842500
O. rufipogon	rufipogon_boundary_9	rufipogon_chr01	2267500	2272500
O. rufipogon	rufipogon_boundary_10	rufipogon_chr01	2342500	2377500
O. rufipogon	rufipogon_boundary_11	rufipogon_chr01	2487500	2492500
O. rufipogon	rufipogon_boundary_12	rufipogon_chr01	2977500	2982500
O. rufipogon	rufipogon_boundary_13	rufipogon_chr01	3067500	3072500
O. rufipogon	rufipogon_boundary_14	rufipogon_chr01	3357500	3397500
O. rufipogon	rufipogon_boundary_15	rufipogon_chr01	3712500	3757500
O. rufipogon	rufipogon_boundary_16	rufipogon_chr01	3902500	3907500
O. rufipogon	rufipogon_boundary_17	rufipogon_chr01	3972500	3977500
O. rufipogon	rufipogon_boundary_18	rufipogon_chr01	4037500	4042500
O. rufipogon	rufipogon_boundary_19	rufipogon_chr01	4117500	4122500
O. rufipogon	rufipogon_boundary_20	rufipogon_chr01	4332500	4377500
O. rufipogon	rufipogon_boundary_21	rufipogon_chr01	4452500	4457500
O. rufipogon	rufipogon_boundary_22	rufipogon_chr01	4502500	4507500
O. rufipogon	rufipogon_boundary_23	rufipogon_chr01	4557500	4562500
O. rufipogon	rufipogon_boundary_24	rufipogon_chr01	4602500	4647500
O. rufipogon	rufipogon_boundary_25	rufipogon_chr01	5362500	5367500
O. rufipogon	rufipogon_boundary_26	rufipogon_chr01	5442500	5447500
O. rufipogon	rufipogon_boundary_27	rufipogon_chr01	5562500	5567500
O. rufipogon	rufipogon_boundary_28	rufipogon_chr01	5727500	5732500
O. rufipogon	rufipogon_boundary_29	rufipogon_chr01	5797500	5802500
O. rufipogon	rufipogon_boundary_30	rufipogon_chr01	5842500	5847500
O. rufipogon	rufipogon_boundary_31	rufipogon_chr01	5932500	5937500
O. rufipogon	rufipogon_boundary_32	rufipogon_chr01	6007500	6012500
O. rufipogon	rufipogon_boundary_33	rufipogon_chr01	6087500	6092500
O. rufipogon	rufipogon_boundary_34	rufipogon_chr01	6167500	6172500
O. rufipogon	rufipogon_boundary_35	rufipogon_chr01	6282500	6287500

O. rufipogon	rufipogon_boundary_36	rufipogon_chr01	6332500	6337500
O. rufipogon	rufipogon_boundary_37	rufipogon_chr01	6417500	6422500
O. rufipogon	rufipogon_boundary_38	rufipogon_chr01	6502500	6507500
O. rufipogon	rufipogon_boundary_39	rufipogon_chr01	6592500	6597500
O. rufipogon	rufipogon_boundary_40	rufipogon_chr01	6717500	6757500
O. rufipogon	rufipogon_boundary_41	rufipogon_chr01	6837500	6842500
O. rufipogon	rufipogon_boundary_42	rufipogon_chr01	6882500	6887500
O. rufipogon	rufipogon_boundary_43	rufipogon_chr01	7967500	7972500
O. rufipogon	rufipogon_boundary_44	rufipogon_chr01	8065000	8112500
O. rufipogon	rufipogon_boundary_45	rufipogon_chr01	8162500	8167500
O. rufipogon	rufipogon_boundary_46	rufipogon_chr01	8382500	8387500
O. rufipogon	rufipogon_boundary_47	rufipogon_chr01	8477500	8482500
O. rufipogon	rufipogon_boundary_48	rufipogon_chr01	8542500	8547500
O. rufipogon	rufipogon_boundary_49	rufipogon_chr01	8642500	8647500
O. rufipogon	rufipogon_boundary_50	rufipogon_chr01	8702500	8747500
O. rufipogon	rufipogon_boundary_51	rufipogon_chr01	9612500	9652500
O. rufipogon	rufipogon_boundary_52	rufipogon_chr01	10007500	10012500
O. rufipogon	rufipogon_boundary_53	rufipogon_chr01	10082500	10087500
O. rufipogon	rufipogon_boundary_54	rufipogon_chr01	10192500	10197500
O. rufipogon	rufipogon_boundary_55	rufipogon_chr01	10247500	10252500
O. rufipogon	rufipogon_boundary_56	rufipogon_chr01	10507500	10552500
O. rufipogon	rufipogon_boundary_57	rufipogon_chr01	10627500	10632500
O. rufipogon	rufipogon_boundary_58	rufipogon_chr01	10937500	10977500
O. rufipogon	rufipogon_boundary_59	rufipogon_chr01	11327500	11332500
O. rufipogon	rufipogon_boundary_60	rufipogon_chr01	11382500	11387500
O. rufipogon	rufipogon_boundary_61	rufipogon_chr01	11427500	11467500
O. rufipogon	rufipogon_boundary_62	rufipogon_chr01	11637500	11642500
O. rufipogon	rufipogon_boundary_63	rufipogon_chr01	11737500	11742500
O. rufipogon	rufipogon_boundary_64	rufipogon_chr01	12202500	12207500
O. rufipogon	rufipogon_boundary_65	rufipogon_chr01	12257500	12262500
O. rufipogon	rufipogon_boundary_66	rufipogon_chr01	13282500	13287500
O. rufipogon	rufipogon_boundary_67	rufipogon_chr01	13397500	13402500

O. rufipogon	rufipogon_boundary_68	rufipogon_chr01	13462500	13467500
O. rufipogon	rufipogon_boundary_69	rufipogon_chr01	13607500	13612500
O. rufipogon	rufipogon_boundary_70	rufipogon_chr01	13752500	13757500
O. rufipogon	rufipogon_boundary_71	rufipogon_chr01	13827500	13832500
O. rufipogon	rufipogon_boundary_72	rufipogon_chr01	14717500	14722500
O. rufipogon	rufipogon_boundary_73	rufipogon_chr01	14872500	14877500
O. rufipogon	rufipogon_boundary_74	rufipogon_chr01	17792500	17797500
O. rufipogon	rufipogon_boundary_75	rufipogon_chr01	17837500	17842500
O. rufipogon	rufipogon_boundary_76	rufipogon_chr01	17970000	17975000
O. rufipogon	rufipogon_boundary_77	rufipogon_chr01	18042500	18090000
O. rufipogon	rufipogon_boundary_78	rufipogon_chr01	18142500	18147500
O. rufipogon	rufipogon_boundary_79	rufipogon_chr01	18582500	18587500
O. rufipogon	rufipogon_boundary_80	rufipogon_chr01	18637500	18642500
O. rufipogon	rufipogon_boundary_81	rufipogon_chr01	18732500	18737500
O. rufipogon	rufipogon_boundary_82	rufipogon_chr01	18847500	18852500
O. rufipogon	rufipogon_boundary_83	rufipogon_chr01	18992500	18997500
O. rufipogon	rufipogon_boundary_84	rufipogon_chr01	19070000	19075000
O. rufipogon	rufipogon_boundary_85	rufipogon_chr01	19342500	19347500
O. rufipogon	rufipogon_boundary_86	rufipogon_chr01	19407500	19412500
O. rufipogon	rufipogon_boundary_87	rufipogon_chr01	19757500	19762500
O. rufipogon	rufipogon_boundary_88	rufipogon_chr01	19817500	19822500
O. rufipogon	rufipogon_boundary_89	rufipogon_chr01	19952500	19957500
O. rufipogon	rufipogon_boundary_90	rufipogon_chr01	20037500	20042500
O. rufipogon	rufipogon_boundary_91	rufipogon_chr01	20387500	20472500
O. rufipogon	rufipogon_boundary_92	rufipogon_chr01	20635000	20682500
O. rufipogon	rufipogon_boundary_93	rufipogon_chr01	21027500	21032500
O. rufipogon	rufipogon_boundary_94	rufipogon_chr01	21097500	21132500
O. rufipogon	rufipogon_boundary_95	rufipogon_chr01	21217500	21222500
O. rufipogon	rufipogon_boundary_96	rufipogon_chr01	21322500	21327500
O. rufipogon	rufipogon_boundary_97	rufipogon_chr01	21382500	21387500
O. rufipogon	rufipogon_boundary_98	rufipogon_chr01	21482500	21487500
O. rufipogon	rufipogon_boundary_99	rufipogon_chr01	21532500	21572500

O. rufipogon	rufipogon_boundary_100	rufipogon_chr01	21897500	21902500
O. rufipogon	rufipogon_boundary_101	rufipogon_chr01	21962500	21967500
O. rufipogon	rufipogon_boundary_102	rufipogon_chr01	22037500	22042500
O. rufipogon	rufipogon_boundary_103	rufipogon_chr01	22167500	22207500
O. rufipogon	rufipogon_boundary_104	rufipogon_chr01	22295000	22300000
O. rufipogon	rufipogon_boundary_105	rufipogon_chr01	22722500	22727500
O. rufipogon	rufipogon_boundary_106	rufipogon_chr01	22767500	22772500
O. rufipogon	rufipogon_boundary_107	rufipogon_chr01	22817500	22882500
O. rufipogon	rufipogon_boundary_108	rufipogon_chr01	22932500	22937500
O. rufipogon	rufipogon_boundary_109	rufipogon_chr01	23167500	23172500
O. rufipogon	rufipogon_boundary_110	rufipogon_chr01	23247500	23252500
O. rufipogon	rufipogon_boundary_111	rufipogon_chr01	23627500	23692500
O. rufipogon	rufipogon_boundary_112	rufipogon_chr01	23777500	23782500
O. rufipogon	rufipogon_boundary_113	rufipogon_chr01	23857500	23862500
O. rufipogon	rufipogon_boundary_114	rufipogon_chr01	23902500	23907500
O. rufipogon	rufipogon_boundary_115	rufipogon_chr01	24002500	24007500
O. rufipogon	rufipogon_boundary_116	rufipogon_chr01	24642500	24647500
O. rufipogon	rufipogon_boundary_117	rufipogon_chr01	24732500	24777500
O. rufipogon	rufipogon_boundary_118	rufipogon_chr01	24827500	24872500
O. rufipogon	rufipogon_boundary_119	rufipogon_chr01	25157500	25227500
O. rufipogon	rufipogon_boundary_120	rufipogon_chr01	25287500	25292500
O. rufipogon	rufipogon_boundary_121	rufipogon_chr01	25872500	25877500
O. rufipogon	rufipogon_boundary_122	rufipogon_chr01	25917500	26032500
O. rufipogon	rufipogon_boundary_123	rufipogon_chr01	26082500	26087500
O. rufipogon	rufipogon_boundary_124	rufipogon_chr01	26147500	26152500
O. rufipogon	rufipogon_boundary_125	rufipogon_chr01	26192500	26197500
O. rufipogon	rufipogon_boundary_126	rufipogon_chr01	26277500	26362500
O. rufipogon	rufipogon_boundary_127	rufipogon_chr01	26702500	26707500
O. rufipogon	rufipogon_boundary_128	rufipogon_chr01	26767500	26772500
O. rufipogon	rufipogon_boundary_129	rufipogon_chr01	26822500	26827500
O. rufipogon	rufipogon_boundary_130	rufipogon_chr01	26947500	26952500
O. rufipogon	rufipogon_boundary_131	rufipogon_chr01	27277500	27282500

O. rufipogon	rufipogon_boundary_132	rufipogon_chr01	27347500	27352500
O. rufipogon	rufipogon_boundary_133	rufipogon_chr01	27402500	27407500
O. rufipogon	rufipogon_boundary_134	rufipogon_chr01	27562500	27567500
O. rufipogon	rufipogon_boundary_135	rufipogon_chr01	27647500	27652500
O. rufipogon	rufipogon_boundary_136	rufipogon_chr01	27777500	27812500
O. rufipogon	rufipogon_boundary_137	rufipogon_chr01	28472500	28512500
O. rufipogon	rufipogon_boundary_138	rufipogon_chr01	28672500	28752500
O. rufipogon	rufipogon_boundary_139	rufipogon_chr01	28792500	28797500
O. rufipogon	rufipogon_boundary_140	rufipogon_chr01	29562500	29597500
O. rufipogon	rufipogon_boundary_141	rufipogon_chr01	29672500	29677500
O. rufipogon	rufipogon_boundary_142	rufipogon_chr01	29872500	29877500
O. rufipogon	rufipogon_boundary_143	rufipogon_chr01	29917500	29922500
O. rufipogon	rufipogon_boundary_144	rufipogon_chr01	30012500	30057500
O. rufipogon	rufipogon_boundary_145	rufipogon_chr01	30317500	30322500
O. rufipogon	rufipogon_boundary_146	rufipogon_chr01	30472500	30477500
O. rufipogon	rufipogon_boundary_147	rufipogon_chr01	30542500	30547500
O. rufipogon	rufipogon_boundary_148	rufipogon_chr01	30837500	30872500
O. rufipogon	rufipogon_boundary_149	rufipogon_chr01	31252500	31257500
O. rufipogon	rufipogon_boundary_150	rufipogon_chr01	31312500	31317500
O. rufipogon	rufipogon_boundary_151	rufipogon_chr01	31387500	31392500
O. rufipogon	rufipogon_boundary_152	rufipogon_chr01	31475000	31480000
O. rufipogon	rufipogon_boundary_153	rufipogon_chr01	31532500	31537500
O. rufipogon	rufipogon_boundary_154	rufipogon_chr01	32467500	32472500
O. rufipogon	rufipogon_boundary_155	rufipogon_chr01	32552500	32557500
O. rufipogon	rufipogon_boundary_156	rufipogon_chr01	32607500	32612500
O. rufipogon	rufipogon_boundary_157	rufipogon_chr01	33155000	33160000
O. rufipogon	rufipogon_boundary_158	rufipogon_chr01	33207500	33212500
O. rufipogon	rufipogon_boundary_159	rufipogon_chr01	33277500	33282500
O. rufipogon	rufipogon_boundary_160	rufipogon_chr01	33362500	33397500
O. rufipogon	rufipogon_boundary_161	rufipogon_chr01	33737500	33777500
O. rufipogon	rufipogon_boundary_162	rufipogon_chr01	34372500	34417500
O. rufipogon	rufipogon_boundary_163	rufipogon_chr01	34502500	34507500

O. rufipogon	rufipogon_boundary_164	rufipogon_chr01	34677500	34717500
O. rufipogon	rufipogon_boundary_165	rufipogon_chr01	35157500	35202500
O. rufipogon	rufipogon_boundary_166	rufipogon_chr01	35257500	35262500
O. rufipogon	rufipogon_boundary_167	rufipogon_chr01	35302500	35307500
O. rufipogon	rufipogon_boundary_168	rufipogon_chr01	35412500	35417500
O. rufipogon	rufipogon_boundary_169	rufipogon_chr01	35457500	35462500
O. rufipogon	rufipogon_boundary_170	rufipogon_chr01	36287500	36292500
O. rufipogon	rufipogon_boundary_171	rufipogon_chr01	36337500	36417500
O. rufipogon	rufipogon_boundary_172	rufipogon_chr01	36547500	36552500
O. rufipogon	rufipogon_boundary_173	rufipogon_chr01	36617500	36672500
O. rufipogon	rufipogon_boundary_174	rufipogon_chr01	36792500	36797500
O. rufipogon	rufipogon_boundary_175	rufipogon_chr01	36852500	36857500
O. rufipogon	rufipogon_boundary_176	rufipogon_chr01	36902500	36907500
O. rufipogon	rufipogon_boundary_177	rufipogon_chr01	37107500	37112500
O. rufipogon	rufipogon_boundary_178	rufipogon_chr01	37157500	37162500
O. rufipogon	rufipogon_boundary_179	rufipogon_chr01	37202500	37207500
O. rufipogon	rufipogon_boundary_180	rufipogon_chr01	37272500	37277500
O. rufipogon	rufipogon_boundary_181	rufipogon_chr01	37335000	37340000
O. rufipogon	rufipogon_boundary_182	rufipogon_chr01	37482500	37517500
O. rufipogon	rufipogon_boundary_183	rufipogon_chr01	37597500	37642500
O. rufipogon	rufipogon_boundary_184	rufipogon_chr01	37717500	37722500
O. rufipogon	rufipogon_boundary_185	rufipogon_chr01	37797500	37802500
O. rufipogon	rufipogon_boundary_186	rufipogon_chr01	37872500	37877500
O. rufipogon	rufipogon_boundary_187	rufipogon_chr01	37922500	37927500
O. rufipogon	rufipogon_boundary_188	rufipogon_chr01	38642500	38647500
O. rufipogon	rufipogon_boundary_189	rufipogon_chr01	38747500	38752500
O. rufipogon	rufipogon_boundary_190	rufipogon_chr01	38802500	38807500
O. rufipogon	rufipogon_boundary_191	rufipogon_chr01	38962500	38967500
O. rufipogon	rufipogon_boundary_192	rufipogon_chr01	39032500	39037500
O. rufipogon	rufipogon_boundary_193	rufipogon_chr01	39115000	39120000
O. rufipogon	rufipogon_boundary_194	rufipogon_chr01	39177500	39182500
O. rufipogon	rufipogon_boundary_195	rufipogon_chr01	39272500	39277500

O. rufipogon	rufipogon_boundary_196	rufipogon_chr01	39337500	39342500
O. rufipogon	rufipogon_boundary_197	rufipogon_chr01	39387500	39392500
O. rufipogon	rufipogon_boundary_198	rufipogon_chr01	39482500	39487500
O. rufipogon	rufipogon_boundary_199	rufipogon_chr01	39565000	39570000
O. rufipogon	rufipogon_boundary_200	rufipogon_chr02	177500	182500
O. rufipogon	rufipogon_boundary_201	rufipogon_chr02	267500	272500
O. rufipogon	rufipogon_boundary_202	rufipogon_chr02	342500	347500
O. rufipogon	rufipogon_boundary_203	rufipogon_chr02	472500	477500
O. rufipogon	rufipogon_boundary_204	rufipogon_chr02	652500	657500
O. rufipogon	rufipogon_boundary_205	rufipogon_chr02	697500	702500
O. rufipogon	rufipogon_boundary_206	rufipogon_chr02	1372500	1377500
O. rufipogon	rufipogon_boundary_207	rufipogon_chr02	1427500	1432500
O. rufipogon	rufipogon_boundary_208	rufipogon_chr02	1482500	1527500
O. rufipogon	rufipogon_boundary_209	rufipogon_chr02	1607500	1612500
O. rufipogon	rufipogon_boundary_210	rufipogon_chr02	1742500	1747500
O. rufipogon	rufipogon_boundary_211	rufipogon_chr02	1802500	1807500
O. rufipogon	rufipogon_boundary_212	rufipogon_chr02	1867500	1872500
O. rufipogon	rufipogon_boundary_213	rufipogon_chr02	1927500	1932500
O. rufipogon	rufipogon_boundary_214	rufipogon_chr02	2017500	2022500
O. rufipogon	rufipogon_boundary_215	rufipogon_chr02	2402500	2407500
O. rufipogon	rufipogon_boundary_216	rufipogon_chr02	2452500	2457500
O. rufipogon	rufipogon_boundary_217	rufipogon_chr02	2517500	2522500
O. rufipogon	rufipogon_boundary_218	rufipogon_chr02	2587500	2592500
O. rufipogon	rufipogon_boundary_219	rufipogon_chr02	2847500	2852500
O. rufipogon	rufipogon_boundary_220	rufipogon_chr02	2917500	2952500
O. rufipogon	rufipogon_boundary_221	rufipogon_chr02	3062500	3067500
O. rufipogon	rufipogon_boundary_222	rufipogon_chr02	3182500	3187500
O. rufipogon	rufipogon_boundary_223	rufipogon_chr02	3347500	3352500
O. rufipogon	rufipogon_boundary_224	rufipogon_chr02	3437500	3442500
O. rufipogon	rufipogon_boundary_225	rufipogon_chr02	3662500	3697500
O. rufipogon	rufipogon_boundary_226	rufipogon_chr02	3747500	3752500
O. rufipogon	rufipogon_boundary_227	rufipogon_chr02	3967500	3972500

O. rufipogon	rufipogon_boundary_228	rufipogon_chr02	4072500	4077500
O. rufipogon	rufipogon_boundary_229	rufipogon_chr02	4307500	4312500
O. rufipogon	rufipogon_boundary_230	rufipogon_chr02	4352500	4357500
O. rufipogon	rufipogon_boundary_231	rufipogon_chr02	4512500	4517500
O. rufipogon	rufipogon_boundary_232	rufipogon_chr02	4570000	4575000
O. rufipogon	rufipogon_boundary_233	rufipogon_chr02	4687500	4735000
O. rufipogon	rufipogon_boundary_234	rufipogon_chr02	4877500	4882500
O. rufipogon	rufipogon_boundary_235	rufipogon_chr02	4957500	4962500
O. rufipogon	rufipogon_boundary_236	rufipogon_chr02	5117500	5157500
O. rufipogon	rufipogon_boundary_237	rufipogon_chr02	6012500	6017500
O. rufipogon	rufipogon_boundary_238	rufipogon_chr02	6087500	6092500
O. rufipogon	rufipogon_boundary_239	rufipogon_chr02	6212500	6217500
O. rufipogon	rufipogon_boundary_240	rufipogon_chr02	6257500	6262500
O. rufipogon	rufipogon_boundary_241	rufipogon_chr02	6612500	6617500
O. rufipogon	rufipogon_boundary_242	rufipogon_chr02	6802500	6807500
O. rufipogon	rufipogon_boundary_243	rufipogon_chr02	7027500	7032500
O. rufipogon	rufipogon_boundary_244	rufipogon_chr02	7107500	7112500
O. rufipogon	rufipogon_boundary_245	rufipogon_chr02	7537500	7597500
O. rufipogon	rufipogon_boundary_246	rufipogon_chr02	7652500	7657500
O. rufipogon	rufipogon_boundary_247	rufipogon_chr02	8162500	8167500
O. rufipogon	rufipogon_boundary_248	rufipogon_chr02	8212500	8217500
O. rufipogon	rufipogon_boundary_249	rufipogon_chr02	8302500	8307500
O. rufipogon	rufipogon_boundary_250	rufipogon_chr02	8347500	8352500
O. rufipogon	rufipogon_boundary_251	rufipogon_chr02	8717500	8752500
O. rufipogon	rufipogon_boundary_252	rufipogon_chr02	9147500	9152500
O. rufipogon	rufipogon_boundary_253	rufipogon_chr02	9237500	9242500
O. rufipogon	rufipogon_boundary_254	rufipogon_chr02	9307500	9312500
O. rufipogon	rufipogon_boundary_255	rufipogon_chr02	9362500	9367500
O. rufipogon	rufipogon_boundary_256	rufipogon_chr02	9472500	9477500
O. rufipogon	rufipogon_boundary_257	rufipogon_chr02	10077500	10082500
O. rufipogon	rufipogon_boundary_258	rufipogon_chr02	10142500	10147500
O. rufipogon	rufipogon_boundary_259	rufipogon_chr02	10237500	10307500

O. rufipogon	rufipogon_boundary_260	rufipogon_chr02	10422500	10427500
O. rufipogon	rufipogon_boundary_261	rufipogon_chr02	10512500	10517500
O. rufipogon	rufipogon_boundary_262	rufipogon_chr02	10707500	10712500
O. rufipogon	rufipogon_boundary_263	rufipogon_chr02	10797500	10802500
O. rufipogon	rufipogon_boundary_264	rufipogon_chr02	10852500	10857500
O. rufipogon	rufipogon_boundary_265	rufipogon_chr02	10932500	10937500
O. rufipogon	rufipogon_boundary_266	rufipogon_chr02	11042500	11047500
O. rufipogon	rufipogon_boundary_267	rufipogon_chr02	11105000	11110000
O. rufipogon	rufipogon_boundary_268	rufipogon_chr02	11197500	11202500
O. rufipogon	rufipogon_boundary_269	rufipogon_chr02	11287500	11292500
O. rufipogon	rufipogon_boundary_270	rufipogon_chr02	11372500	11377500
O. rufipogon	rufipogon_boundary_271	rufipogon_chr02	12032500	12037500
O. rufipogon	rufipogon_boundary_272	rufipogon_chr02	12137500	12142500
O. rufipogon	rufipogon_boundary_273	rufipogon_chr02	12242500	12247500
O. rufipogon	rufipogon_boundary_274	rufipogon_chr02	12307500	12312500
O. rufipogon	rufipogon_boundary_275	rufipogon_chr02	12367500	12372500
O. rufipogon	rufipogon_boundary_276	rufipogon_chr02	12427500	12470000
O. rufipogon	rufipogon_boundary_277	rufipogon_chr02	12542500	12547500
O. rufipogon	rufipogon_boundary_278	rufipogon_chr02	12587500	12592500
O. rufipogon	rufipogon_boundary_279	rufipogon_chr02	12727500	12732500
O. rufipogon	rufipogon_boundary_280	rufipogon_chr02	12785000	12790000
O. rufipogon	rufipogon_boundary_281	rufipogon_chr02	12897500	12902500
O. rufipogon	rufipogon_boundary_282	rufipogon_chr02	13162500	13202500
O. rufipogon	rufipogon_boundary_283	rufipogon_chr02	13912500	13917500
O. rufipogon	rufipogon_boundary_284	rufipogon_chr02	14007500	14012500
O. rufipogon	rufipogon_boundary_285	rufipogon_chr02	14247500	14290000
O. rufipogon	rufipogon_boundary_286	rufipogon_chr02	15457500	15462500
O. rufipogon	rufipogon_boundary_287	rufipogon_chr02	15582500	15587500
O. rufipogon	rufipogon_boundary_288	rufipogon_chr02	17392500	17397500
O. rufipogon	rufipogon_boundary_289	rufipogon_chr02	17482500	17487500
O. rufipogon	rufipogon_boundary_290	rufipogon_chr02	17747500	17752500
O. rufipogon	rufipogon_boundary_291	rufipogon_chr02	17807500	17812500

O. rufipogon	rufipogon_boundary_292	rufipogon_chr02	18177500	18182500
O. rufipogon	rufipogon_boundary_293	rufipogon_chr02	18302500	18307500
O. rufipogon	rufipogon_boundary_294	rufipogon_chr02	18517500	18522500
O. rufipogon	rufipogon_boundary_295	rufipogon_chr02	18577500	18582500
O. rufipogon	rufipogon_boundary_296	rufipogon_chr02	18652500	18657500
O. rufipogon	rufipogon_boundary_297	rufipogon_chr02	18707500	18712500
O. rufipogon	rufipogon_boundary_298	rufipogon_chr02	18812500	18817500
O. rufipogon	rufipogon_boundary_299	rufipogon_chr02	19050000	19055000
O. rufipogon	rufipogon_boundary_300	rufipogon_chr02	19142500	19147500
O. rufipogon	rufipogon_boundary_301	rufipogon_chr02	19977500	19982500
O. rufipogon	rufipogon_boundary_302	rufipogon_chr02	20042500	20047500
O. rufipogon	rufipogon_boundary_303	rufipogon_chr02	20632500	20702500
O. rufipogon	rufipogon_boundary_304	rufipogon_chr02	20877500	20882500
O. rufipogon	rufipogon_boundary_305	rufipogon_chr02	20997500	21002500
O. rufipogon	rufipogon_boundary_306	rufipogon_chr02	21747500	21752500
O. rufipogon	rufipogon_boundary_307	rufipogon_chr02	21892500	21897500
O. rufipogon	rufipogon_boundary_308	rufipogon_chr02	22007500	22012500
O. rufipogon	rufipogon_boundary_309	rufipogon_chr02	22062500	22067500
O. rufipogon	rufipogon_boundary_310	rufipogon_chr02	22112500	22117500
O. rufipogon	rufipogon_boundary_311	rufipogon_chr02	22367500	22372500
O. rufipogon	rufipogon_boundary_312	rufipogon_chr02	22432500	22437500
O. rufipogon	rufipogon_boundary_313	rufipogon_chr02	22477500	22512500
O. rufipogon	rufipogon_boundary_314	rufipogon_chr02	22607500	22677500
O. rufipogon	rufipogon_boundary_315	rufipogon_chr02	22757500	22762500
O. rufipogon	rufipogon_boundary_316	rufipogon_chr02	23257500	23297500
O. rufipogon	rufipogon_boundary_317	rufipogon_chr02	23342500	23347500
O. rufipogon	rufipogon_boundary_318	rufipogon_chr02	23442500	23447500
O. rufipogon	rufipogon_boundary_319	rufipogon_chr02	23557500	23602500
O. rufipogon	rufipogon_boundary_320	rufipogon_chr02	23642500	23647500
O. rufipogon	rufipogon_boundary_321	rufipogon_chr02	23687500	23692500
O. rufipogon	rufipogon_boundary_322	rufipogon_chr02	24222500	24227500
O. rufipogon	rufipogon_boundary_323	rufipogon_chr02	24312500	24317500

O. rufipogon	rufipogon_boundary_324	rufipogon_chr02	24642500	24647500
O. rufipogon	rufipogon_boundary_325	rufipogon_chr02	24687500	24692500
O. rufipogon	rufipogon_boundary_326	rufipogon_chr02	24732500	24762500
O. rufipogon	rufipogon_boundary_327	rufipogon_chr02	24812500	24817500
O. rufipogon	rufipogon_boundary_328	rufipogon_chr02	24872500	24877500
O. rufipogon	rufipogon_boundary_329	rufipogon_chr02	24917500	24952500
O. rufipogon	rufipogon_boundary_330	rufipogon_chr02	25047500	25052500
O. rufipogon	rufipogon_boundary_331	rufipogon_chr02	25150000	25155000
O. rufipogon	rufipogon_boundary_332	rufipogon_chr02	25747500	25817500
O. rufipogon	rufipogon_boundary_333	rufipogon_chr02	26112500	26117500
O. rufipogon	rufipogon_boundary_334	rufipogon_chr02	26157500	26197500
O. rufipogon	rufipogon_boundary_335	rufipogon_chr02	26257500	26262500
O. rufipogon	rufipogon_boundary_336	rufipogon_chr02	26737500	26742500
O. rufipogon	rufipogon_boundary_337	rufipogon_chr02	26792500	26797500
O. rufipogon	rufipogon_boundary_338	rufipogon_chr02	26912500	26917500
O. rufipogon	rufipogon_boundary_339	rufipogon_chr02	27082500	27087500
O. rufipogon	rufipogon_boundary_340	rufipogon_chr02	27302500	27307500
O. rufipogon	rufipogon_boundary_341	rufipogon_chr02	27367500	27372500
O. rufipogon	rufipogon_boundary_342	rufipogon_chr02	27832500	27837500
O. rufipogon	rufipogon_boundary_343	rufipogon_chr02	27887500	27892500
O. rufipogon	rufipogon_boundary_344	rufipogon_chr02	28015000	28020000
O. rufipogon	rufipogon_boundary_345	rufipogon_chr02	28137500	28142500
O. rufipogon	rufipogon_boundary_346	rufipogon_chr02	28332500	28337500
O. rufipogon	rufipogon_boundary_347	rufipogon_chr02	28407500	28412500
O. rufipogon	rufipogon_boundary_348	rufipogon_chr02	28502500	28507500
O. rufipogon	rufipogon_boundary_349	rufipogon_chr02	28627500	28632500
O. rufipogon	rufipogon_boundary_350	rufipogon_chr02	28702500	28707500
O. rufipogon	rufipogon_boundary_351	rufipogon_chr02	28747500	28752500
O. rufipogon	rufipogon_boundary_352	rufipogon_chr02	28812500	28847500
O. rufipogon	rufipogon_boundary_353	rufipogon_chr02	28907500	28912500
O. rufipogon	rufipogon_boundary_354	rufipogon_chr02	28957500	28962500
O. rufipogon	rufipogon_boundary_355	rufipogon_chr02	29040000	29045000

O. rufipogon	rufipogon_boundary_356	rufipogon_chr02	29462500	29467500
O. rufipogon	rufipogon_boundary_357	rufipogon_chr02	29527500	29532500
O. rufipogon	rufipogon_boundary_358	rufipogon_chr02	29597500	29602500
O. rufipogon	rufipogon_boundary_359	rufipogon_chr02	30047500	30052500
O. rufipogon	rufipogon_boundary_360	rufipogon_chr02	30132500	30137500
O. rufipogon	rufipogon_boundary_361	rufipogon_chr02	30432500	30437500
O. rufipogon	rufipogon_boundary_362	rufipogon_chr02	30542500	30547500
O. rufipogon	rufipogon_boundary_363	rufipogon_chr02	31277500	31282500
O. rufipogon	rufipogon_boundary_364	rufipogon_chr02	31332500	31337500
O. rufipogon	rufipogon_boundary_365	rufipogon_chr02	31417500	31422500
O. rufipogon	rufipogon_boundary_366	rufipogon_chr02	31467500	31547500
O. rufipogon	rufipogon_boundary_367	rufipogon_chr02	31747500	31752500
O. rufipogon	rufipogon_boundary_368	rufipogon_chr02	31817500	31822500
O. rufipogon	rufipogon_boundary_369	rufipogon_chr02	31882500	31887500
O. rufipogon	rufipogon_boundary_370	rufipogon_chr02	31957500	31962500
O. rufipogon	rufipogon_boundary_371	rufipogon_chr02	32227500	32377500
O. rufipogon	rufipogon_boundary_372	rufipogon_chr02	33302500	33307500
O. rufipogon	rufipogon_boundary_373	rufipogon_chr02	33412500	33417500
O. rufipogon	rufipogon_boundary_374	rufipogon_chr02	33612500	33617500
O. rufipogon	rufipogon_boundary_375	rufipogon_chr02	33712500	33717500
O. rufipogon	rufipogon_boundary_376	rufipogon_chr02	33767500	33772500
O. rufipogon	rufipogon_boundary_377	rufipogon_chr03	262500	267500
O. rufipogon	rufipogon_boundary_378	rufipogon_chr03	337500	342500
O. rufipogon	rufipogon_boundary_379	rufipogon_chr03	402500	407500
O. rufipogon	rufipogon_boundary_380	rufipogon_chr03	482500	487500
O. rufipogon	rufipogon_boundary_381	rufipogon_chr03	537500	542500
O. rufipogon	rufipogon_boundary_382	rufipogon_chr03	587500	592500
O. rufipogon	rufipogon_boundary_383	rufipogon_chr03	637500	642500
O. rufipogon	rufipogon_boundary_384	rufipogon_chr03	692500	697500
O. rufipogon	rufipogon_boundary_385	rufipogon_chr03	1142500	1147500
O. rufipogon	rufipogon_boundary_386	rufipogon_chr03	1217500	1222500
O. rufipogon	rufipogon_boundary_387	rufipogon_chr03	1267500	1272500

O. rufipogon	rufipogon_boundary_388	rufipogon_chr03	1322500	1327500
O. rufipogon	rufipogon_boundary_389	rufipogon_chr03	1627500	1632500
O. rufipogon	rufipogon_boundary_390	rufipogon_chr03	1692500	1697500
O. rufipogon	rufipogon_boundary_391	rufipogon_chr03	2087500	2092500
O. rufipogon	rufipogon_boundary_392	rufipogon_chr03	2192500	2197500
O. rufipogon	rufipogon_boundary_393	rufipogon_chr03	2482500	2487500
O. rufipogon	rufipogon_boundary_394	rufipogon_chr03	2557500	2562500
O. rufipogon	rufipogon_boundary_395	rufipogon_chr03	2602500	2607500
O. rufipogon	rufipogon_boundary_396	rufipogon_chr03	2652500	2657500
O. rufipogon	rufipogon_boundary_397	rufipogon_chr03	2782500	2787500
O. rufipogon	rufipogon_boundary_398	rufipogon_chr03	2842500	2882500
O. rufipogon	rufipogon_boundary_399	rufipogon_chr03	2957500	2997500
O. rufipogon	rufipogon_boundary_400	rufipogon_chr03	3682500	3687500
O. rufipogon	rufipogon_boundary_401	rufipogon_chr03	3747500	3752500
O. rufipogon	rufipogon_boundary_402	rufipogon_chr03	4082500	4087500
O. rufipogon	rufipogon_boundary_403	rufipogon_chr03	4182500	4187500
O. rufipogon	rufipogon_boundary_404	rufipogon_chr03	4227500	4232500
O. rufipogon	rufipogon_boundary_405	rufipogon_chr03	4277500	4282500
O. rufipogon	rufipogon_boundary_406	rufipogon_chr03	4357500	4362500
O. rufipogon	rufipogon_boundary_407	rufipogon_chr03	4447500	4452500
O. rufipogon	rufipogon_boundary_408	rufipogon_chr03	4492500	4497500
O. rufipogon	rufipogon_boundary_409	rufipogon_chr03	4592500	4597500
O. rufipogon	rufipogon_boundary_410	rufipogon_chr03	4662500	4727500
O. rufipogon	rufipogon_boundary_411	rufipogon_chr03	4777500	4782500
O. rufipogon	rufipogon_boundary_412	rufipogon_chr03	5232500	5237500
O. rufipogon	rufipogon_boundary_413	rufipogon_chr03	5322500	5327500
O. rufipogon	rufipogon_boundary_414	rufipogon_chr03	5382500	5387500
O. rufipogon	rufipogon_boundary_415	rufipogon_chr03	5857500	5892500
O. rufipogon	rufipogon_boundary_416	rufipogon_chr03	5982500	5987500
O. rufipogon	rufipogon_boundary_417	rufipogon_chr03	6027500	6032500
O. rufipogon	rufipogon_boundary_418	rufipogon_chr03	6112500	6157500
O. rufipogon	rufipogon_boundary_419	rufipogon_chr03	6202500	6207500

O. rufipogon	rufipogon_boundary_420	rufipogon_chr03	6312500	6352500
O. rufipogon	rufipogon_boundary_421	rufipogon_chr03	6407500	6412500
O. rufipogon	rufipogon_boundary_422	rufipogon_chr03	7172500	7177500
O. rufipogon	rufipogon_boundary_423	rufipogon_chr03	7252500	7257500
O. rufipogon	rufipogon_boundary_424	rufipogon_chr03	7302500	7307500
O. rufipogon	rufipogon_boundary_425	rufipogon_chr03	8562500	8567500
O. rufipogon	rufipogon_boundary_426	rufipogon_chr03	8752500	8757500
O. rufipogon	rufipogon_boundary_427	rufipogon_chr03	8852500	8932500
O. rufipogon	rufipogon_boundary_428	rufipogon_chr03	9162500	9167500
O. rufipogon	rufipogon_boundary_429	rufipogon_chr03	9227500	9232500
O. rufipogon	rufipogon_boundary_430	rufipogon_chr03	9782500	9822500
O. rufipogon	rufipogon_boundary_431	rufipogon_chr03	10072500	10107500
O. rufipogon	rufipogon_boundary_432	rufipogon_chr03	10237500	10277500
O. rufipogon	rufipogon_boundary_433	rufipogon_chr03	10482500	10487500
O. rufipogon	rufipogon_boundary_434	rufipogon_chr03	10577500	10582500
O. rufipogon	rufipogon_boundary_435	rufipogon_chr03	10712500	10717500
O. rufipogon	rufipogon_boundary_436	rufipogon_chr03	10757500	10802500
O. rufipogon	rufipogon_boundary_437	rufipogon_chr03	10842500	10887500
O. rufipogon	rufipogon_boundary_438	rufipogon_chr03	11097500	11102500
O. rufipogon	rufipogon_boundary_439	rufipogon_chr03	11147500	11152500
O. rufipogon	rufipogon_boundary_440	rufipogon_chr03	11387500	11392500
O. rufipogon	rufipogon_boundary_441	rufipogon_chr03	11432500	11467500
O. rufipogon	rufipogon_boundary_442	rufipogon_chr03	11522500	11527500
O. rufipogon	rufipogon_boundary_443	rufipogon_chr03	11572500	11577500
O. rufipogon	rufipogon_boundary_444	rufipogon_chr03	11647500	11652500
O. rufipogon	rufipogon_boundary_445	rufipogon_chr03	11712500	11757500
O. rufipogon	rufipogon_boundary_446	rufipogon_chr03	11817500	11822500
O. rufipogon	rufipogon_boundary_447	rufipogon_chr03	11877500	11912500
O. rufipogon	rufipogon_boundary_448	rufipogon_chr03	12032500	12037500
O. rufipogon	rufipogon_boundary_449	rufipogon_chr03	12627500	12632500
O. rufipogon	rufipogon_boundary_450	rufipogon_chr03	12727500	12732500
O. rufipogon	rufipogon_boundary_451	rufipogon_chr03	13222500	13227500

O. rufipogon	rufipogon_boundary_452	rufipogon_chr03	13292500	13297500
O. rufipogon	rufipogon_boundary_453	rufipogon_chr03	13362500	13367500
O. rufipogon	rufipogon_boundary_454	rufipogon_chr03	13522500	13527500
O. rufipogon	rufipogon_boundary_455	rufipogon_chr03	13567500	13572500
O. rufipogon	rufipogon_boundary_456	rufipogon_chr03	13867500	13907500
O. rufipogon	rufipogon_boundary_457	rufipogon_chr03	13957500	13962500
O. rufipogon	rufipogon_boundary_458	rufipogon_chr03	14002500	14007500
O. rufipogon	rufipogon_boundary_459	rufipogon_chr03	14087500	14092500
O. rufipogon	rufipogon_boundary_460	rufipogon_chr03	14167500	14172500
O. rufipogon	rufipogon_boundary_461	rufipogon_chr03	14412500	14417500
O. rufipogon	rufipogon_boundary_462	rufipogon_chr03	14467500	14472500
O. rufipogon	rufipogon_boundary_463	rufipogon_chr03	14627500	14632500
O. rufipogon	rufipogon_boundary_464	rufipogon_chr03	14677500	14682500
O. rufipogon	rufipogon_boundary_465	rufipogon_chr03	14737500	14742500
O. rufipogon	rufipogon_boundary_466	rufipogon_chr03	14787500	14792500
O. rufipogon	rufipogon_boundary_467	rufipogon_chr03	14842500	14847500
O. rufipogon	rufipogon_boundary_468	rufipogon_chr03	14902500	14907500
O. rufipogon	rufipogon_boundary_469	rufipogon_chr03	15102500	15107500
O. rufipogon	rufipogon_boundary_470	rufipogon_chr03	15157500	15162500
O. rufipogon	rufipogon_boundary_471	rufipogon_chr03	15232500	15237500
O. rufipogon	rufipogon_boundary_472	rufipogon_chr03	16872500	16877500
O. rufipogon	rufipogon_boundary_473	rufipogon_chr03	16927500	16932500
O. rufipogon	rufipogon_boundary_474	rufipogon_chr03	18127500	18132500
O. rufipogon	rufipogon_boundary_475	rufipogon_chr03	18205000	18210000
O. rufipogon	rufipogon_boundary_476	rufipogon_chr03	18267500	18272500
O. rufipogon	rufipogon_boundary_477	rufipogon_chr03	18317500	18322500
O. rufipogon	rufipogon_boundary_478	rufipogon_chr03	18400000	18442500
O. rufipogon	rufipogon_boundary_479	rufipogon_chr03	18582500	18587500
O. rufipogon	rufipogon_boundary_480	rufipogon_chr03	18662500	18667500
O. rufipogon	rufipogon_boundary_481	rufipogon_chr03	19297500	19302500
O. rufipogon	rufipogon_boundary_482	rufipogon_chr03	19422500	19427500
O. rufipogon	rufipogon_boundary_483	rufipogon_chr03	19957500	19962500

O. rufipogon	rufipogon_boundary_484	rufipogon_chr03	20022500	20027500
O. rufipogon	rufipogon_boundary_485	rufipogon_chr03	20080000	20085000
O. rufipogon	rufipogon_boundary_486	rufipogon_chr03	20127500	20132500
O. rufipogon	rufipogon_boundary_487	rufipogon_chr03	20207500	20242500
O. rufipogon	rufipogon_boundary_488	rufipogon_chr03	20312500	20317500
O. rufipogon	rufipogon_boundary_489	rufipogon_chr03	20357500	20362500
O. rufipogon	rufipogon_boundary_490	rufipogon_chr03	20467500	20472500
O. rufipogon	rufipogon_boundary_491	rufipogon_chr03	20512500	20517500
O. rufipogon	rufipogon_boundary_492	rufipogon_chr03	20642500	20647500
O. rufipogon	rufipogon_boundary_493	rufipogon_chr03	20795000	20800000
O. rufipogon	rufipogon_boundary_494	rufipogon_chr03	20927500	20967500
O. rufipogon	rufipogon_boundary_495	rufipogon_chr03	21042500	21047500
O. rufipogon	rufipogon_boundary_496	rufipogon_chr03	21122500	21127500
O. rufipogon	rufipogon_boundary_497	rufipogon_chr03	21172500	21177500
O. rufipogon	rufipogon_boundary_498	rufipogon_chr03	22292500	22297500
O. rufipogon	rufipogon_boundary_499	rufipogon_chr03	22397500	22402500
O. rufipogon	rufipogon_boundary_500	rufipogon_chr03	22537500	22542500
O. rufipogon	rufipogon_boundary_501	rufipogon_chr03	22632500	22637500
O. rufipogon	rufipogon_boundary_502	rufipogon_chr03	22687500	22732500
O. rufipogon	rufipogon_boundary_503	rufipogon_chr03	22807500	22812500
O. rufipogon	rufipogon_boundary_504	rufipogon_chr03	22857500	22902500
O. rufipogon	rufipogon_boundary_505	rufipogon_chr03	23652500	23687500
O. rufipogon	rufipogon_boundary_506	rufipogon_chr03	24682500	24687500
O. rufipogon	rufipogon_boundary_507	rufipogon_chr03	24742500	24747500
O. rufipogon	rufipogon_boundary_508	rufipogon_chr03	24787500	24792500
O. rufipogon	rufipogon_boundary_509	rufipogon_chr03	26627500	26662500
O. rufipogon	rufipogon_boundary_510	rufipogon_chr03	26712500	26717500
O. rufipogon	rufipogon_boundary_511	rufipogon_chr03	26777500	26782500
O. rufipogon	rufipogon_boundary_512	rufipogon_chr03	26837500	26872500
O. rufipogon	rufipogon_boundary_513	rufipogon_chr03	26947500	26952500
O. rufipogon	rufipogon_boundary_514	rufipogon_chr03	27022500	27052500
O. rufipogon	rufipogon_boundary_515	rufipogon_chr03	27207500	27212500

O. rufipogon	rufipogon_boundary_516	rufipogon_chr03	27302500	27307500
O. rufipogon	rufipogon_boundary_517	rufipogon_chr03	27372500	27377500
O. rufipogon	rufipogon_boundary_518	rufipogon_chr03	27567500	27572500
O. rufipogon	rufipogon_boundary_519	rufipogon_chr03	27642500	27647500
O. rufipogon	rufipogon_boundary_520	rufipogon_chr03	27952500	27997500
O. rufipogon	rufipogon_boundary_521	rufipogon_chr03	28237500	28242500
O. rufipogon	rufipogon_boundary_522	rufipogon_chr03	28292500	28297500
O. rufipogon	rufipogon_boundary_523	rufipogon_chr03	28557500	28562500
O. rufipogon	rufipogon_boundary_524	rufipogon_chr03	28637500	28677500
O. rufipogon	rufipogon_boundary_525	rufipogon_chr03	28732500	28737500
O. rufipogon	rufipogon_boundary_526	rufipogon_chr03	28977500	29022500
O. rufipogon	rufipogon_boundary_527	rufipogon_chr03	29927500	29972500
O. rufipogon	rufipogon_boundary_528	rufipogon_chr03	30032500	30037500
O. rufipogon	rufipogon_boundary_529	rufipogon_chr03	30082500	30087500
O. rufipogon	rufipogon_boundary_530	rufipogon_chr03	30392500	30432500
O. rufipogon	rufipogon_boundary_531	rufipogon_chr03	30512500	30517500
O. rufipogon	rufipogon_boundary_532	rufipogon_chr03	31352500	31357500
O. rufipogon	rufipogon_boundary_533	rufipogon_chr03	31407500	31412500
O. rufipogon	rufipogon_boundary_534	rufipogon_chr03	32022500	32057500
O. rufipogon	rufipogon_boundary_535	rufipogon_chr03	32562500	32607500
O. rufipogon	rufipogon_boundary_536	rufipogon_chr03	32747500	32752500
O. rufipogon	rufipogon_boundary_537	rufipogon_chr03	32827500	32832500
O. rufipogon	rufipogon_boundary_538	rufipogon_chr03	32887500	32892500
O. rufipogon	rufipogon_boundary_539	rufipogon_chr03	32937500	32942500
O. rufipogon	rufipogon_boundary_540	rufipogon_chr03	33297500	33302500
O. rufipogon	rufipogon_boundary_541	rufipogon_chr03	33372500	33377500
O. rufipogon	rufipogon_boundary_542	rufipogon_chr03	33495000	33500000
O. rufipogon	rufipogon_boundary_543	rufipogon_chr03	33982500	33987500
O. rufipogon	rufipogon_boundary_544	rufipogon_chr03	34037500	34042500
O. rufipogon	rufipogon_boundary_545	rufipogon_chr03	34122500	34162500
O. rufipogon	rufipogon_boundary_546	rufipogon_chr03	34227500	34232500
O. rufipogon	rufipogon_boundary_547	rufipogon_chr04	317500	352500

O. rufipogon	rufipogon_boundary_548	rufipogon_chr04	587500	592500
O. rufipogon	rufipogon_boundary_549	rufipogon_chr04	657500	662500
O. rufipogon	rufipogon_boundary_550	rufipogon_chr04	3417500	3422500
O. rufipogon	rufipogon_boundary_551	rufipogon_chr04	3517500	3522500
O. rufipogon	rufipogon_boundary_552	rufipogon_chr04	3652500	3657500
O. rufipogon	rufipogon_boundary_553	rufipogon_chr04	3717500	3722500
O. rufipogon	rufipogon_boundary_554	rufipogon_chr04	3832500	3877500
O. rufipogon	rufipogon_boundary_555	rufipogon_chr04	4520000	4525000
O. rufipogon	rufipogon_boundary_556	rufipogon_chr04	4582500	4587500
O. rufipogon	rufipogon_boundary_557	rufipogon_chr04	4675000	4680000
O. rufipogon	rufipogon_boundary_558	rufipogon_chr04	4917500	4922500
O. rufipogon	rufipogon_boundary_559	rufipogon_chr04	4997500	5002500
O. rufipogon	rufipogon_boundary_560	rufipogon_chr04	5172500	5177500
O. rufipogon	rufipogon_boundary_561	rufipogon_chr04	5255000	5260000
O. rufipogon	rufipogon_boundary_562	rufipogon_chr04	5315000	5320000
O. rufipogon	rufipogon_boundary_563	rufipogon_chr04	5397500	5402500
O. rufipogon	rufipogon_boundary_564	rufipogon_chr04	5467500	5512500
O. rufipogon	rufipogon_boundary_565	rufipogon_chr04	5952500	5957500
O. rufipogon	rufipogon_boundary_566	rufipogon_chr04	6037500	6042500
O. rufipogon	rufipogon_boundary_567	rufipogon_chr04	6852500	6857500
O. rufipogon	rufipogon_boundary_568	rufipogon_chr04	6955000	6960000
O. rufipogon	rufipogon_boundary_569	rufipogon_chr04	7702500	7707500
O. rufipogon	rufipogon_boundary_570	rufipogon_chr04	7765000	7770000
O. rufipogon	rufipogon_boundary_571	rufipogon_chr04	10242500	10287500
O. rufipogon	rufipogon_boundary_572	rufipogon_chr04	10372500	10420000
O. rufipogon	rufipogon_boundary_573	rufipogon_chr04	10462500	10467500
O. rufipogon	rufipogon_boundary_574	rufipogon_chr04	10612500	10685000
O. rufipogon	rufipogon_boundary_575	rufipogon_chr04	10767500	10772500
O. rufipogon	rufipogon_boundary_576	rufipogon_chr04	11087500	11092500
O. rufipogon	rufipogon_boundary_577	rufipogon_chr04	11142500	11147500
O. rufipogon	rufipogon_boundary_578	rufipogon_chr04	11492500	11497500
O. rufipogon	rufipogon_boundary_579	rufipogon_chr04	11617500	11622500

O. rufipogon	rufipogon_boundary_580	rufipogon_chr04	12115000	12120000
O. rufipogon	rufipogon_boundary_581	rufipogon_chr04	12182500	12187500
O. rufipogon	rufipogon_boundary_582	rufipogon_chr04	12327500	12332500
O. rufipogon	rufipogon_boundary_583	rufipogon_chr04	12387500	12392500
O. rufipogon	rufipogon_boundary_584	rufipogon_chr04	13057500	13062500
O. rufipogon	rufipogon_boundary_585	rufipogon_chr04	13112500	13117500
O. rufipogon	rufipogon_boundary_586	rufipogon_chr04	13167500	13172500
O. rufipogon	rufipogon_boundary_587	rufipogon_chr04	13217500	13222500
O. rufipogon	rufipogon_boundary_588	rufipogon_chr04	13272500	13277500
O. rufipogon	rufipogon_boundary_589	rufipogon_chr04	13330000	13335000
O. rufipogon	rufipogon_boundary_590	rufipogon_chr04	14782500	14817500
O. rufipogon	rufipogon_boundary_591	rufipogon_chr04	15572500	15577500
O. rufipogon	rufipogon_boundary_592	rufipogon_chr04	15657500	15662500
O. rufipogon	rufipogon_boundary_593	rufipogon_chr04	15977500	15982500
O. rufipogon	rufipogon_boundary_594	rufipogon_chr04	16047500	16052500
O. rufipogon	rufipogon_boundary_595	rufipogon_chr04	16522500	16527500
O. rufipogon	rufipogon_boundary_596	rufipogon_chr04	16632500	16637500
O. rufipogon	rufipogon_boundary_597	rufipogon_chr04	17042500	17087500
O. rufipogon	rufipogon_boundary_598	rufipogon_chr04	17170000	17175000
O. rufipogon	rufipogon_boundary_599	rufipogon_chr04	17297500	17302500
O. rufipogon	rufipogon_boundary_600	rufipogon_chr04	17352500	17357500
O. rufipogon	rufipogon_boundary_601	rufipogon_chr04	17802500	17807500
O. rufipogon	rufipogon_boundary_602	rufipogon_chr04	17932500	17937500
O. rufipogon	rufipogon_boundary_603	rufipogon_chr04	18042500	18047500
O. rufipogon	rufipogon_boundary_604	rufipogon_chr04	20042500	20047500
O. rufipogon	rufipogon_boundary_605	rufipogon_chr04	20097500	20102500
O. rufipogon	rufipogon_boundary_606	rufipogon_chr04	20207500	20212500
O. rufipogon	rufipogon_boundary_607	rufipogon_chr04	20307500	20352500
O. rufipogon	rufipogon_boundary_608	rufipogon_chr04	20397500	20402500
O. rufipogon	rufipogon_boundary_609	rufipogon_chr04	20537500	20542500
O. rufipogon	rufipogon_boundary_610	rufipogon_chr04	20652500	20657500
O. rufipogon	rufipogon_boundary_611	rufipogon_chr04	20717500	20722500

O. rufipogon	rufipogon_boundary_612	rufipogon_chr04	20887500	20892500
O. rufipogon	rufipogon_boundary_613	rufipogon_chr04	20957500	20962500
O. rufipogon	rufipogon_boundary_614	rufipogon_chr04	21527500	21532500
O. rufipogon	rufipogon_boundary_615	rufipogon_chr04	21622500	21627500
O. rufipogon	rufipogon_boundary_616	rufipogon_chr04	21957500	21962500
O. rufipogon	rufipogon_boundary_617	rufipogon_chr04	22087500	22092500
O. rufipogon	rufipogon_boundary_618	rufipogon_chr04	22162500	22167500
O. rufipogon	rufipogon_boundary_619	rufipogon_chr04	22637500	22642500
O. rufipogon	rufipogon_boundary_620	rufipogon_chr04	22682500	22687500
O. rufipogon	rufipogon_boundary_621	rufipogon_chr04	22737500	22742500
O. rufipogon	rufipogon_boundary_622	rufipogon_chr04	23157500	23162500
O. rufipogon	rufipogon_boundary_623	rufipogon_chr04	23250000	23255000
O. rufipogon	rufipogon_boundary_624	rufipogon_chr04	23307500	23312500
O. rufipogon	rufipogon_boundary_625	rufipogon_chr04	23432500	23437500
O. rufipogon	rufipogon_boundary_626	rufipogon_chr04	23487500	23492500
O. rufipogon	rufipogon_boundary_627	rufipogon_chr04	23612500	23617500
O. rufipogon	rufipogon_boundary_628	rufipogon_chr04	24102500	24107500
O. rufipogon	rufipogon_boundary_629	rufipogon_chr04	24157500	24162500
O. rufipogon	rufipogon_boundary_630	rufipogon_chr04	24227500	24232500
O. rufipogon	rufipogon_boundary_631	rufipogon_chr04	24637500	24642500
O. rufipogon	rufipogon_boundary_632	rufipogon_chr04	24687500	24692500
O. rufipogon	rufipogon_boundary_633	rufipogon_chr04	24822500	24857500
O. rufipogon	rufipogon_boundary_634	rufipogon_chr04	24907500	24912500
O. rufipogon	rufipogon_boundary_635	rufipogon_chr04	24962500	24967500
O. rufipogon	rufipogon_boundary_636	rufipogon_chr04	25127500	25172500
O. rufipogon	rufipogon_boundary_637	rufipogon_chr04	26567500	26572500
O. rufipogon	rufipogon_boundary_638	rufipogon_chr04	26622500	26667500
O. rufipogon	rufipogon_boundary_639	rufipogon_chr04	27027500	27032500
O. rufipogon	rufipogon_boundary_640	rufipogon_chr04	27142500	27147500
O. rufipogon	rufipogon_boundary_641	rufipogon_chr04	28027500	28032500
O. rufipogon	rufipogon_boundary_642	rufipogon_chr04	28102500	28107500
O. rufipogon	rufipogon_boundary_643	rufipogon_chr04	29092500	29097500

O. rufipogon	rufipogon_boundary_644	rufipogon_chr04	29202500	29207500
O. rufipogon	rufipogon_boundary_645	rufipogon_chr04	29247500	29252500
O. rufipogon	rufipogon_boundary_646	rufipogon_chr04	29542500	29547500
O. rufipogon	rufipogon_boundary_647	rufipogon_chr04	29617500	29622500
O. rufipogon	rufipogon_boundary_648	rufipogon_chr04	29797500	29802500
O. rufipogon	rufipogon_boundary_649	rufipogon_chr04	29907500	29912500
O. rufipogon	rufipogon_boundary_650	rufipogon_chr05	92500	97500
O. rufipogon	rufipogon_boundary_651	rufipogon_chr05	142500	147500
O. rufipogon	rufipogon_boundary_652	rufipogon_chr05	227500	232500
O. rufipogon	rufipogon_boundary_653	rufipogon_chr05	597500	627500
O. rufipogon	rufipogon_boundary_654	rufipogon_chr05	702500	707500
O. rufipogon	rufipogon_boundary_655	rufipogon_chr05	807500	812500
O. rufipogon	rufipogon_boundary_656	rufipogon_chr05	2332500	2337500
O. rufipogon	rufipogon_boundary_657	rufipogon_chr05	2392500	2397500
O. rufipogon	rufipogon_boundary_658	rufipogon_chr05	2627500	2632500
O. rufipogon	rufipogon_boundary_659	rufipogon_chr05	2695000	2700000
O. rufipogon	rufipogon_boundary_660	rufipogon_chr05	2957500	2962500
O. rufipogon	rufipogon_boundary_661	rufipogon_chr05	3052500	3057500
O. rufipogon	rufipogon_boundary_662	rufipogon_chr05	3152500	3157500
O. rufipogon	rufipogon_boundary_663	rufipogon_chr05	3207500	3212500
O. rufipogon	rufipogon_boundary_664	rufipogon_chr05	3272500	3277500
O. rufipogon	rufipogon_boundary_665	rufipogon_chr05	3317500	3322500
O. rufipogon	rufipogon_boundary_666	rufipogon_chr05	3377500	3417500
O. rufipogon	rufipogon_boundary_667	rufipogon_chr05	3477500	3482500
O. rufipogon	rufipogon_boundary_668	rufipogon_chr05	4342500	4347500
O. rufipogon	rufipogon_boundary_669	rufipogon_chr05	4442500	4447500
O. rufipogon	rufipogon_boundary_670	rufipogon_chr05	4832500	4837500
O. rufipogon	rufipogon_boundary_671	rufipogon_chr05	4887500	4892500
O. rufipogon	rufipogon_boundary_672	rufipogon_chr05	5267500	5272500
O. rufipogon	rufipogon_boundary_673	rufipogon_chr05	5372500	5377500
O. rufipogon	rufipogon_boundary_674	rufipogon_chr05	6022500	6027500
O. rufipogon	rufipogon_boundary_675	rufipogon_chr05	6092500	6097500

O. rufipogon	rufipogon_boundary_676	rufipogon_chr05	6152500	6157500
O. rufipogon	rufipogon_boundary_677	rufipogon_chr05	6232500	6237500
O. rufipogon	rufipogon_boundary_678	rufipogon_chr05	7372500	7377500
O. rufipogon	rufipogon_boundary_679	rufipogon_chr05	7492500	7497500
O. rufipogon	rufipogon_boundary_680	rufipogon_chr05	7590000	7595000
O. rufipogon	rufipogon_boundary_681	rufipogon_chr05	7687500	7692500
O. rufipogon	rufipogon_boundary_682	rufipogon_chr05	7732500	7737500
O. rufipogon	rufipogon_boundary_683	rufipogon_chr05	7842500	7847500
O. rufipogon	rufipogon_boundary_684	rufipogon_chr05	9475000	9480000
O. rufipogon	rufipogon_boundary_685	rufipogon_chr05	9525000	9530000
O. rufipogon	rufipogon_boundary_686	rufipogon_chr05	9672500	9712500
O. rufipogon	rufipogon_boundary_687	rufipogon_chr05	9817500	9822500
O. rufipogon	rufipogon_boundary_688	rufipogon_chr05	9867500	9872500
O. rufipogon	rufipogon_boundary_689	rufipogon_chr05	10692500	10697500
O. rufipogon	rufipogon_boundary_690	rufipogon_chr05	10775000	10780000
O. rufipogon	rufipogon_boundary_691	rufipogon_chr05	10880000	10885000
O. rufipogon	rufipogon_boundary_692	rufipogon_chr05	10942500	10947500
O. rufipogon	rufipogon_boundary_693	rufipogon_chr05	11052500	11057500
O. rufipogon	rufipogon_boundary_694	rufipogon_chr05	11307500	11312500
O. rufipogon	rufipogon_boundary_695	rufipogon_chr05	11367500	11372500
O. rufipogon	rufipogon_boundary_696	rufipogon_chr05	11482500	11487500
O. rufipogon	rufipogon_boundary_697	rufipogon_chr05	11572500	11577500
O. rufipogon	rufipogon_boundary_698	rufipogon_chr05	11647500	11652500
O. rufipogon	rufipogon_boundary_699	rufipogon_chr05	11742500	11747500
O. rufipogon	rufipogon_boundary_700	rufipogon_chr05	11787500	11792500
O. rufipogon	rufipogon_boundary_701	rufipogon_chr05	13332500	13337500
O. rufipogon	rufipogon_boundary_702	rufipogon_chr05	13435000	13440000
O. rufipogon	rufipogon_boundary_703	rufipogon_chr05	13517500	13522500
O. rufipogon	rufipogon_boundary_704	rufipogon_chr05	13572500	13577500
O. rufipogon	rufipogon_boundary_705	rufipogon_chr05	13710000	13715000
O. rufipogon	rufipogon_boundary_706	rufipogon_chr05	13787500	13792500
O. rufipogon	rufipogon_boundary_707	rufipogon_chr05	13857500	13862500

O. rufipogon	rufipogon_boundary_708	rufipogon_chr05	13977500	13982500
O. rufipogon	rufipogon_boundary_709	rufipogon_chr05	14367500	14372500
O. rufipogon	rufipogon_boundary_710	rufipogon_chr05	14467500	14472500
O. rufipogon	rufipogon_boundary_711	rufipogon_chr05	14532500	14537500
O. rufipogon	rufipogon_boundary_712	rufipogon_chr05	14857500	14862500
O. rufipogon	rufipogon_boundary_713	rufipogon_chr05	14902500	14907500
O. rufipogon	rufipogon_boundary_714	rufipogon_chr05	15417500	15422500
O. rufipogon	rufipogon_boundary_715	rufipogon_chr05	15492500	15497500
O. rufipogon	rufipogon_boundary_716	rufipogon_chr05	16142500	16147500
O. rufipogon	rufipogon_boundary_717	rufipogon_chr05	16197500	16202500
O. rufipogon	rufipogon_boundary_718	rufipogon_chr05	16472500	16517500
O. rufipogon	rufipogon_boundary_719	rufipogon_chr05	16707500	16712500
O. rufipogon	rufipogon_boundary_720	rufipogon_chr05	16812500	16817500
O. rufipogon	rufipogon_boundary_721	rufipogon_chr05	16992500	16997500
O. rufipogon	rufipogon_boundary_722	rufipogon_chr05	17152500	17157500
O. rufipogon	rufipogon_boundary_723	rufipogon_chr05	17220000	17225000
O. rufipogon	rufipogon_boundary_724	rufipogon_chr05	17302500	17307500
O. rufipogon	rufipogon_boundary_725	rufipogon_chr05	17402500	17407500
O. rufipogon	rufipogon_boundary_726	rufipogon_chr05	17452500	17457500
O. rufipogon	rufipogon_boundary_727	rufipogon_chr05	17517500	17522500
O. rufipogon	rufipogon_boundary_728	rufipogon_chr05	18242500	18247500
O. rufipogon	rufipogon_boundary_729	rufipogon_chr05	18352500	18387500
O. rufipogon	rufipogon_boundary_730	rufipogon_chr05	18447500	18452500
O. rufipogon	rufipogon_boundary_731	rufipogon_chr05	18532500	18537500
O. rufipogon	rufipogon_boundary_732	rufipogon_chr05	18697500	18702500
O. rufipogon	rufipogon_boundary_733	rufipogon_chr05	18747500	18752500
O. rufipogon	rufipogon_boundary_734	rufipogon_chr05	19492500	19532500
O. rufipogon	rufipogon_boundary_735	rufipogon_chr05	19712500	19717500
O. rufipogon	rufipogon_boundary_736	rufipogon_chr05	19757500	19762500
O. rufipogon	rufipogon_boundary_737	rufipogon_chr05	19817500	19822500
O. rufipogon	rufipogon_boundary_738	rufipogon_chr05	19912500	19917500
O. rufipogon	rufipogon_boundary_739	rufipogon_chr05	20107500	20147500

O. rufipogon	rufipogon_boundary_740	rufipogon_chr05	20317500	20322500
O. rufipogon	rufipogon_boundary_741	rufipogon_chr05	20382500	20457500
O. rufipogon	rufipogon_boundary_742	rufipogon_chr05	20597500	20602500
O. rufipogon	rufipogon_boundary_743	rufipogon_chr05	20682500	20687500
O. rufipogon	rufipogon_boundary_744	rufipogon_chr05	20747500	20752500
O. rufipogon	rufipogon_boundary_745	rufipogon_chr05	21032500	21037500
O. rufipogon	rufipogon_boundary_746	rufipogon_chr05	21087500	21092500
O. rufipogon	rufipogon_boundary_747	rufipogon_chr05	21132500	21137500
O. rufipogon	rufipogon_boundary_748	rufipogon_chr05	21192500	21197500
O. rufipogon	rufipogon_boundary_749	rufipogon_chr05	21527500	21532500
O. rufipogon	rufipogon_boundary_750	rufipogon_chr05	21617500	21622500
O. rufipogon	rufipogon_boundary_751	rufipogon_chr05	22152500	22157500
O. rufipogon	rufipogon_boundary_752	rufipogon_chr05	22212500	22217500
O. rufipogon	rufipogon_boundary_753	rufipogon_chr05	22277500	22322500
O. rufipogon	rufipogon_boundary_754	rufipogon_chr05	22522500	22527500
O. rufipogon	rufipogon_boundary_755	rufipogon_chr05	22612500	22617500
O. rufipogon	rufipogon_boundary_756	rufipogon_chr05	23552500	23557500
O. rufipogon	rufipogon_boundary_757	rufipogon_chr05	23657500	23662500
O. rufipogon	rufipogon_boundary_758	rufipogon_chr05	24012500	24017500
O. rufipogon	rufipogon_boundary_759	rufipogon_chr05	24082500	24087500
O. rufipogon	rufipogon_boundary_760	rufipogon_chr05	24367500	24372500
O. rufipogon	rufipogon_boundary_761	rufipogon_chr05	24422500	24462500
O. rufipogon	rufipogon_boundary_762	rufipogon_chr05	25012500	25057500
O. rufipogon	rufipogon_boundary_763	rufipogon_chr05	25117500	25122500
O. rufipogon	rufipogon_boundary_764	rufipogon_chr05	25222500	25267500
O. rufipogon	rufipogon_boundary_765	rufipogon_chr05	25357500	25362500
O. rufipogon	rufipogon_boundary_766	rufipogon_chr05	25432500	25437500
O. rufipogon	rufipogon_boundary_767	rufipogon_chr05	25482500	25487500
O. rufipogon	rufipogon_boundary_768	rufipogon_chr05	25932500	25937500
O. rufipogon	rufipogon_boundary_769	rufipogon_chr05	25977500	25982500
O. rufipogon	rufipogon_boundary_770	rufipogon_chr05	26117500	26122500
O. rufipogon	rufipogon_boundary_771	rufipogon_chr05	26357500	26362500

O. rufipogon	rufipogon_boundary_772	rufipogon_chr05	26422500	26427500
O. rufipogon	rufipogon_boundary_773	rufipogon_chr06	302500	347500
O. rufipogon	rufipogon_boundary_774	rufipogon_chr06	422500	427500
O. rufipogon	rufipogon_boundary_775	rufipogon_chr06	477500	482500
O. rufipogon	rufipogon_boundary_776	rufipogon_chr06	797500	802500
O. rufipogon	rufipogon_boundary_777	rufipogon_chr06	917500	922500
O. rufipogon	rufipogon_boundary_778	rufipogon_chr06	1987500	1992500
O. rufipogon	rufipogon_boundary_779	rufipogon_chr06	2042500	2047500
O. rufipogon	rufipogon_boundary_780	rufipogon_chr06	2167500	2172500
O. rufipogon	rufipogon_boundary_781	rufipogon_chr06	2297500	2332500
O. rufipogon	rufipogon_boundary_782	rufipogon_chr06	2407500	2412500
O. rufipogon	rufipogon_boundary_783	rufipogon_chr06	2497500	2502500
O. rufipogon	rufipogon_boundary_784	rufipogon_chr06	3132500	3167500
O. rufipogon	rufipogon_boundary_785	rufipogon_chr06	3232500	3237500
O. rufipogon	rufipogon_boundary_786	rufipogon_chr06	3292500	3297500
O. rufipogon	rufipogon_boundary_787	rufipogon_chr06	4232500	4237500
O. rufipogon	rufipogon_boundary_788	rufipogon_chr06	4297500	4302500
O. rufipogon	rufipogon_boundary_789	rufipogon_chr06	4477500	4482500
O. rufipogon	rufipogon_boundary_790	rufipogon_chr06	5502500	5507500
O. rufipogon	rufipogon_boundary_791	rufipogon_chr06	5582500	5587500
O. rufipogon	rufipogon_boundary_792	rufipogon_chr06	6562500	6567500
O. rufipogon	rufipogon_boundary_793	rufipogon_chr06	6622500	6627500
O. rufipogon	rufipogon_boundary_794	rufipogon_chr06	6957500	6962500
O. rufipogon	rufipogon_boundary_795	rufipogon_chr06	7017500	7022500
O. rufipogon	rufipogon_boundary_796	rufipogon_chr06	7072500	7077500
O. rufipogon	rufipogon_boundary_797	rufipogon_chr06	7117500	7162500
O. rufipogon	rufipogon_boundary_798	rufipogon_chr06	7252500	7257500
O. rufipogon	rufipogon_boundary_799	rufipogon_chr06	7307500	7347500
O. rufipogon	rufipogon_boundary_800	rufipogon_chr06	7387500	7392500
O. rufipogon	rufipogon_boundary_801	rufipogon_chr06	7527500	7532500
O. rufipogon	rufipogon_boundary_802	rufipogon_chr06	7602500	7607500
O. rufipogon	rufipogon_boundary_803	rufipogon_chr06	8612500	8617500

O. rufipogon	rufipogon_boundary_804	rufipogon_chr06	8720000	8725000
O. rufipogon	rufipogon_boundary_805	rufipogon_chr06	8777500	8782500
O. rufipogon	rufipogon_boundary_806	rufipogon_chr06	8977500	8982500
O. rufipogon	rufipogon_boundary_807	rufipogon_chr06	9062500	9067500
O. rufipogon	rufipogon_boundary_808	rufipogon_chr06	9207500	9212500
O. rufipogon	rufipogon_boundary_809	rufipogon_chr06	9275000	9280000
O. rufipogon	rufipogon_boundary_810	rufipogon_chr06	9417500	9422500
O. rufipogon	rufipogon_boundary_811	rufipogon_chr06	9505000	9510000
O. rufipogon	rufipogon_boundary_812	rufipogon_chr06	9600000	9605000
O. rufipogon	rufipogon_boundary_813	rufipogon_chr06	9772500	9777500
O. rufipogon	rufipogon_boundary_814	rufipogon_chr06	9817500	9822500
O. rufipogon	rufipogon_boundary_815	rufipogon_chr06	9862500	9867500
O. rufipogon	rufipogon_boundary_816	rufipogon_chr06	9937500	9942500
O. rufipogon	rufipogon_boundary_817	rufipogon_chr06	10057500	10062500
O. rufipogon	rufipogon_boundary_818	rufipogon_chr06	10132500	10172500
O. rufipogon	rufipogon_boundary_819	rufipogon_chr06	11512500	11517500
O. rufipogon	rufipogon_boundary_820	rufipogon_chr06	11577500	11582500
O. rufipogon	rufipogon_boundary_821	rufipogon_chr06	11885000	11890000
O. rufipogon	rufipogon_boundary_822	rufipogon_chr06	11947500	11952500
O. rufipogon	rufipogon_boundary_823	rufipogon_chr06	14080000	14085000
O. rufipogon	rufipogon_boundary_824	rufipogon_chr06	14232500	14237500
O. rufipogon	rufipogon_boundary_825	rufipogon_chr06	14322500	14327500
O. rufipogon	rufipogon_boundary_826	rufipogon_chr06	14495000	14500000
O. rufipogon	rufipogon_boundary_827	rufipogon_chr06	14807500	14812500
O. rufipogon	rufipogon_boundary_828	rufipogon_chr06	14912500	14917500
O. rufipogon	rufipogon_boundary_829	rufipogon_chr06	15467500	15472500
O. rufipogon	rufipogon_boundary_830	rufipogon_chr06	15540000	15545000
O. rufipogon	rufipogon_boundary_831	rufipogon_chr06	15627500	15632500
O. rufipogon	rufipogon_boundary_832	rufipogon_chr06	15727500	15732500
O. rufipogon	rufipogon_boundary_833	rufipogon_chr06	16830000	16835000
O. rufipogon	rufipogon_boundary_834	rufipogon_chr06	16907500	16912500
O. rufipogon	rufipogon_boundary_835	rufipogon_chr06	17317500	17322500

O. rufipogon	rufipogon_boundary_836	rufipogon_chr06	17387500	17392500
O. rufipogon	rufipogon_boundary_837	rufipogon_chr06	17567500	17572500
O. rufipogon	rufipogon_boundary_838	rufipogon_chr06	17647500	17687500
O. rufipogon	rufipogon_boundary_839	rufipogon_chr06	17832500	17837500
O. rufipogon	rufipogon_boundary_840	rufipogon_chr06	17982500	18017500
O. rufipogon	rufipogon_boundary_841	rufipogon_chr06	18587500	18592500
O. rufipogon	rufipogon_boundary_842	rufipogon_chr06	18672500	18677500
O. rufipogon	rufipogon_boundary_843	rufipogon_chr06	18717500	18722500
O. rufipogon	rufipogon_boundary_844	rufipogon_chr06	18817500	18847500
O. rufipogon	rufipogon_boundary_845	rufipogon_chr06	18902500	18907500
O. rufipogon	rufipogon_boundary_846	rufipogon_chr06	18947500	18952500
O. rufipogon	rufipogon_boundary_847	rufipogon_chr06	18992500	18997500
O. rufipogon	rufipogon_boundary_848	rufipogon_chr06	19057500	19062500
O. rufipogon	rufipogon_boundary_849	rufipogon_chr06	19312500	19317500
O. rufipogon	rufipogon_boundary_850	rufipogon_chr06	19402500	19407500
O. rufipogon	rufipogon_boundary_851	rufipogon_chr06	19467500	19472500
O. rufipogon	rufipogon_boundary_852	rufipogon_chr06	19572500	19577500
O. rufipogon	rufipogon_boundary_853	rufipogon_chr06	19632500	19637500
O. rufipogon	rufipogon_boundary_854	rufipogon_chr06	19697500	19702500
O. rufipogon	rufipogon_boundary_855	rufipogon_chr06	19742500	19747500
O. rufipogon	rufipogon_boundary_856	rufipogon_chr06	19812500	19817500
O. rufipogon	rufipogon_boundary_857	rufipogon_chr06	20027500	20032500
O. rufipogon	rufipogon_boundary_858	rufipogon_chr06	20077500	20082500
O. rufipogon	rufipogon_boundary_859	rufipogon_chr06	20157500	20162500
O. rufipogon	rufipogon_boundary_860	rufipogon_chr06	20232500	20277500
O. rufipogon	rufipogon_boundary_861	rufipogon_chr06	20802500	20842500
O. rufipogon	rufipogon_boundary_862	rufipogon_chr06	20902500	20907500
O. rufipogon	rufipogon_boundary_863	rufipogon_chr06	20957500	20962500
O. rufipogon	rufipogon_boundary_864	rufipogon_chr06	21372500	21417500
O. rufipogon	rufipogon_boundary_865	rufipogon_chr06	21497500	21502500
O. rufipogon	rufipogon_boundary_866	rufipogon_chr06	21702500	21707500
O. rufipogon	rufipogon_boundary_867	rufipogon_chr06	21757500	21762500

O. rufipogon	rufipogon_boundary_868	rufipogon_chr06	23562500	23567500
O. rufipogon	rufipogon_boundary_869	rufipogon_chr06	23622500	23667500
O. rufipogon	rufipogon_boundary_870	rufipogon_chr06	24822500	24827500
O. rufipogon	rufipogon_boundary_871	rufipogon_chr06	24892500	24897500
O. rufipogon	rufipogon_boundary_872	rufipogon_chr06	24977500	24982500
O. rufipogon	rufipogon_boundary_873	rufipogon_chr06	25237500	25242500
O. rufipogon	rufipogon_boundary_874	rufipogon_chr06	25327500	25332500
O. rufipogon	rufipogon_boundary_875	rufipogon_chr06	25437500	25442500
O. rufipogon	rufipogon_boundary_876	rufipogon_chr06	25517500	25522500
O. rufipogon	rufipogon_boundary_877	rufipogon_chr06	25587500	25592500
O. rufipogon	rufipogon_boundary_878	rufipogon_chr06	25652500	25657500
O. rufipogon	rufipogon_boundary_879	rufipogon_chr06	25737500	25742500
O. rufipogon	rufipogon_boundary_880	rufipogon_chr06	26017500	26022500
O. rufipogon	rufipogon_boundary_881	rufipogon_chr06	26080000	26085000
O. rufipogon	rufipogon_boundary_882	rufipogon_chr06	26402500	26407500
O. rufipogon	rufipogon_boundary_883	rufipogon_chr06	26477500	26482500
O. rufipogon	rufipogon_boundary_884	rufipogon_chr06	26607500	26612500
O. rufipogon	rufipogon_boundary_885	rufipogon_chr06	26667500	26672500
O. rufipogon	rufipogon_boundary_886	rufipogon_chr06	26797500	26802500
O. rufipogon	rufipogon_boundary_887	rufipogon_chr06	26847500	26852500
O. rufipogon	rufipogon_boundary_888	rufipogon_chr06	27292500	27297500
O. rufipogon	rufipogon_boundary_889	rufipogon_chr06	27387500	27392500
O. rufipogon	rufipogon_boundary_890	rufipogon_chr07	482500	487500
O. rufipogon	rufipogon_boundary_891	rufipogon_chr07	537500	542500
O. rufipogon	rufipogon_boundary_892	rufipogon_chr07	662500	667500
O. rufipogon	rufipogon_boundary_893	rufipogon_chr07	717500	722500
O. rufipogon	rufipogon_boundary_894	rufipogon_chr07	772500	777500
O. rufipogon	rufipogon_boundary_895	rufipogon_chr07	822500	827500
O. rufipogon	rufipogon_boundary_896	rufipogon_chr07	887500	892500
O. rufipogon	rufipogon_boundary_897	rufipogon_chr07	1645000	1650000
O. rufipogon	rufipogon_boundary_898	rufipogon_chr07	1702500	1707500
O. rufipogon	rufipogon_boundary_899	rufipogon_chr07	1747500	1752500

O. rufipogon	rufipogon_boundary_900	rufipogon_chr07	1817500	1822500
O. rufipogon	rufipogon_boundary_901	rufipogon_chr07	1892500	1897500
O. rufipogon	rufipogon_boundary_902	rufipogon_chr07	2322500	2377500
O. rufipogon	rufipogon_boundary_903	rufipogon_chr07	2747500	2752500
O. rufipogon	rufipogon_boundary_904	rufipogon_chr07	2792500	2797500
O. rufipogon	rufipogon_boundary_905	rufipogon_chr07	3232500	3237500
O. rufipogon	rufipogon_boundary_906	rufipogon_chr07	3337500	3342500
O. rufipogon	rufipogon_boundary_907	rufipogon_chr07	3382500	3387500
O. rufipogon	rufipogon_boundary_908	rufipogon_chr07	3467500	3472500
O. rufipogon	rufipogon_boundary_909	rufipogon_chr07	3522500	3527500
O. rufipogon	rufipogon_boundary_910	rufipogon_chr07	3567500	3572500
O. rufipogon	rufipogon_boundary_911	rufipogon_chr07	3622500	3662500
O. rufipogon	rufipogon_boundary_912	rufipogon_chr07	3747500	3752500
O. rufipogon	rufipogon_boundary_913	rufipogon_chr07	4087500	4092500
O. rufipogon	rufipogon_boundary_914	rufipogon_chr07	4177500	4182500
O. rufipogon	rufipogon_boundary_915	rufipogon_chr07	4272500	4277500
O. rufipogon	rufipogon_boundary_916	rufipogon_chr07	4332500	4337500
O. rufipogon	rufipogon_boundary_917	rufipogon_chr07	4407500	4442500
O. rufipogon	rufipogon_boundary_918	rufipogon_chr07	4487500	4492500
O. rufipogon	rufipogon_boundary_919	rufipogon_chr07	4532500	4580000
O. rufipogon	rufipogon_boundary_920	rufipogon_chr07	4662500	4667500
O. rufipogon	rufipogon_boundary_921	rufipogon_chr07	5217500	5222500
O. rufipogon	rufipogon_boundary_922	rufipogon_chr07	5307500	5312500
O. rufipogon	rufipogon_boundary_923	rufipogon_chr07	5982500	5987500
O. rufipogon	rufipogon_boundary_924	rufipogon_chr07	6057500	6062500
O. rufipogon	rufipogon_boundary_925	rufipogon_chr07	6727500	6732500
O. rufipogon	rufipogon_boundary_926	rufipogon_chr07	6847500	6852500
O. rufipogon	rufipogon_boundary_927	rufipogon_chr07	6987500	6992500
O. rufipogon	rufipogon_boundary_928	rufipogon_chr07	7727500	7762500
O. rufipogon	rufipogon_boundary_929	rufipogon_chr07	8087500	8092500
O. rufipogon	rufipogon_boundary_930	rufipogon_chr07	8132500	8137500
O. rufipogon	rufipogon_boundary_931	rufipogon_chr07	9047500	9092500

O. rufipogon	rufipogon_boundary_932	rufipogon_chr07	9907500	9912500
O. rufipogon	rufipogon_boundary_933	rufipogon_chr07	9997500	10002500
O. rufipogon	rufipogon_boundary_934	rufipogon_chr07	10177500	10182500
O. rufipogon	rufipogon_boundary_935	rufipogon_chr07	10227500	10262500
O. rufipogon	rufipogon_boundary_936	rufipogon_chr07	10352500	10397500
O. rufipogon	rufipogon_boundary_937	rufipogon_chr07	10697500	10702500
O. rufipogon	rufipogon_boundary_938	rufipogon_chr07	10747500	10752500
O. rufipogon	rufipogon_boundary_939	rufipogon_chr07	11292500	11297500
O. rufipogon	rufipogon_boundary_940	rufipogon_chr07	11402500	11407500
O. rufipogon	rufipogon_boundary_941	rufipogon_chr07	11715000	11720000
O. rufipogon	rufipogon_boundary_942	rufipogon_chr07	11850000	11855000
O. rufipogon	rufipogon_boundary_943	rufipogon_chr07	12320000	12325000
O. rufipogon	rufipogon_boundary_944	rufipogon_chr07	12442500	12447500
O. rufipogon	rufipogon_boundary_945	rufipogon_chr07	12547500	12552500
O. rufipogon	rufipogon_boundary_946	rufipogon_chr07	12617500	12622500
O. rufipogon	rufipogon_boundary_947	rufipogon_chr07	13707500	13712500
O. rufipogon	rufipogon_boundary_948	rufipogon_chr07	13802500	13807500
O. rufipogon	rufipogon_boundary_949	rufipogon_chr07	13860000	13865000
O. rufipogon	rufipogon_boundary_950	rufipogon_chr07	13952500	13957500
O. rufipogon	rufipogon_boundary_951	rufipogon_chr07	14062500	14067500
O. rufipogon	rufipogon_boundary_952	rufipogon_chr07	14147500	14152500
O. rufipogon	rufipogon_boundary_953	rufipogon_chr07	14272500	14277500
O. rufipogon	rufipogon_boundary_954	rufipogon_chr07	14332500	14337500
O. rufipogon	rufipogon_boundary_955	rufipogon_chr07	14392500	14397500
O. rufipogon	rufipogon_boundary_956	rufipogon_chr07	14497500	14502500
O. rufipogon	rufipogon_boundary_957	rufipogon_chr07	14640000	14687500
O. rufipogon	rufipogon_boundary_958	rufipogon_chr07	14737500	14742500
O. rufipogon	rufipogon_boundary_959	rufipogon_chr07	14812500	14817500
O. rufipogon	rufipogon_boundary_960	rufipogon_chr07	15637500	15642500
O. rufipogon	rufipogon_boundary_961	rufipogon_chr07	15707500	15712500
O. rufipogon	rufipogon_boundary_962	rufipogon_chr07	15837500	15842500
O. rufipogon	rufipogon_boundary_963	rufipogon_chr07	15957500	15962500

O. rufipogon	rufipogon_boundary_964	rufipogon_chr07	16242500	16247500
O. rufipogon	rufipogon_boundary_965	rufipogon_chr07	16342500	16347500
O. rufipogon	rufipogon_boundary_966	rufipogon_chr07	16582500	16587500
O. rufipogon	rufipogon_boundary_967	rufipogon_chr07	16667500	16672500
O. rufipogon	rufipogon_boundary_968	rufipogon_chr07	16782500	16787500
O. rufipogon	rufipogon_boundary_969	rufipogon_chr07	16877500	16882500
O. rufipogon	rufipogon_boundary_970	rufipogon_chr07	16982500	16987500
O. rufipogon	rufipogon_boundary_971	rufipogon_chr07	17337500	17342500
O. rufipogon	rufipogon_boundary_972	rufipogon_chr07	17392500	17397500
O. rufipogon	rufipogon_boundary_973	rufipogon_chr07	17462500	17467500
O. rufipogon	rufipogon_boundary_974	rufipogon_chr07	17567500	17572500
O. rufipogon	rufipogon_boundary_975	rufipogon_chr07	17962500	17997500
O. rufipogon	rufipogon_boundary_976	rufipogon_chr07	18602500	18607500
O. rufipogon	rufipogon_boundary_977	rufipogon_chr07	18647500	18652500
O. rufipogon	rufipogon_boundary_978	rufipogon_chr07	18872500	18917500
O. rufipogon	rufipogon_boundary_979	rufipogon_chr07	18987500	18992500
O. rufipogon	rufipogon_boundary_980	rufipogon_chr07	19057500	19062500
O. rufipogon	rufipogon_boundary_981	rufipogon_chr07	19157500	19162500
O. rufipogon	rufipogon_boundary_982	rufipogon_chr07	19202500	19207500
O. rufipogon	rufipogon_boundary_983	rufipogon_chr07	19262500	19267500
O. rufipogon	rufipogon_boundary_984	rufipogon_chr07	19462500	19467500
O. rufipogon	rufipogon_boundary_985	rufipogon_chr07	19537500	19542500
O. rufipogon	rufipogon_boundary_986	rufipogon_chr07	19592500	19597500
O. rufipogon	rufipogon_boundary_987	rufipogon_chr07	19787500	19792500
O. rufipogon	rufipogon_boundary_988	rufipogon_chr07	19842500	19882500
O. rufipogon	rufipogon_boundary_989	rufipogon_chr07	19952500	19957500
O. rufipogon	rufipogon_boundary_990	rufipogon_chr07	20312500	20317500
O. rufipogon	rufipogon_boundary_991	rufipogon_chr07	20367500	20372500
O. rufipogon	rufipogon_boundary_992	rufipogon_chr07	21027500	21032500
O. rufipogon	rufipogon_boundary_993	rufipogon_chr07	21092500	21097500
O. rufipogon	rufipogon_boundary_994	rufipogon_chr07	21552500	21557500
O. rufipogon	rufipogon_boundary_995	rufipogon_chr07	21612500	21617500

O. rufipogon	rufipogon_boundary_996	rufipogon_chr07	21702500	21707500
O. rufipogon	rufipogon_boundary_997	rufipogon_chr07	21767500	21772500
O. rufipogon	rufipogon_boundary_998	rufipogon_chr07	21832500	21837500
O. rufipogon	rufipogon_boundary_999	rufipogon_chr07	22072500	22107500
O. rufipogon	rufipogon_boundary_1000	rufipogon_chr07	22517500	22522500
O. rufipogon	rufipogon_boundary_1001	rufipogon_chr07	22577500	22582500
O. rufipogon	rufipogon_boundary_1002	rufipogon_chr07	24217500	24222500
O. rufipogon	rufipogon_boundary_1003	rufipogon_chr07	24267500	24272500
O. rufipogon	rufipogon_boundary_1004	rufipogon_chr07	24327500	24332500
O. rufipogon	rufipogon_boundary_1005	rufipogon_chr07	24392500	24397500
O. rufipogon	rufipogon_boundary_1006	rufipogon_chr07	24467500	24472500
O. rufipogon	rufipogon_boundary_1007	rufipogon_chr07	24942500	24977500
O. rufipogon	rufipogon_boundary_1008	rufipogon_chr07	25632500	25637500
O. rufipogon	rufipogon_boundary_1009	rufipogon_chr07	25682500	25687500
O. rufipogon	rufipogon_boundary_1010	rufipogon_chr08	142500	147500
O. rufipogon	rufipogon_boundary_1011	rufipogon_chr08	192500	197500
O. rufipogon	rufipogon_boundary_1012	rufipogon_chr08	267500	272500
O. rufipogon	rufipogon_boundary_1013	rufipogon_chr08	347500	352500
O. rufipogon	rufipogon_boundary_1014	rufipogon_chr08	622500	627500
O. rufipogon	rufipogon_boundary_1015	rufipogon_chr08	707500	712500
O. rufipogon	rufipogon_boundary_1016	rufipogon_chr08	852500	857500
O. rufipogon	rufipogon_boundary_1017	rufipogon_chr08	922500	927500
O. rufipogon	rufipogon_boundary_1018	rufipogon_chr08	2852500	2857500
O. rufipogon	rufipogon_boundary_1019	rufipogon_chr08	2902500	2907500
O. rufipogon	rufipogon_boundary_1020	rufipogon_chr08	2947500	2952500
O. rufipogon	rufipogon_boundary_1021	rufipogon_chr08	4497500	4502500
O. rufipogon	rufipogon_boundary_1022	rufipogon_chr08	4542500	4547500
O. rufipogon	rufipogon_boundary_1023	rufipogon_chr08	4600000	4605000
O. rufipogon	rufipogon_boundary_1024	rufipogon_chr08	7047500	7092500
O. rufipogon	rufipogon_boundary_1025	rufipogon_chr08	7252500	7300000
O. rufipogon	rufipogon_boundary_1026	rufipogon_chr08	7372500	7377500
O. rufipogon	rufipogon_boundary_1027	rufipogon_chr08	7462500	7467500

O. rufipogon	rufipogon_boundary_1028	rufipogon_chr08	7822500	7827500
O. rufipogon	rufipogon_boundary_1029	rufipogon_chr08	7887500	7892500
O. rufipogon	rufipogon_boundary_1030	rufipogon_chr08	7942500	7987500
O. rufipogon	rufipogon_boundary_1031	rufipogon_chr08	9010000	9015000
O. rufipogon	rufipogon_boundary_1032	rufipogon_chr08	9057500	9062500
O. rufipogon	rufipogon_boundary_1033	rufipogon_chr08	9322500	9327500
O. rufipogon	rufipogon_boundary_1034	rufipogon_chr08	9407500	9412500
O. rufipogon	rufipogon_boundary_1035	rufipogon_chr08	9472500	9477500
O. rufipogon	rufipogon_boundary_1036	rufipogon_chr08	9542500	9582500
O. rufipogon	rufipogon_boundary_1037	rufipogon_chr08	9712500	9717500
O. rufipogon	rufipogon_boundary_1038	rufipogon_chr08	10455000	10460000
O. rufipogon	rufipogon_boundary_1039	rufipogon_chr08	10527500	10642500
O. rufipogon	rufipogon_boundary_1040	rufipogon_chr08	10710000	10715000
O. rufipogon	rufipogon_boundary_1041	rufipogon_chr08	10775000	10780000
O. rufipogon	rufipogon_boundary_1042	rufipogon_chr08	10842500	10847500
O. rufipogon	rufipogon_boundary_1043	rufipogon_chr08	10977500	10982500
O. rufipogon	rufipogon_boundary_1044	rufipogon_chr08	11052500	11057500
O. rufipogon	rufipogon_boundary_1045	rufipogon_chr08	11517500	11562500
O. rufipogon	rufipogon_boundary_1046	rufipogon_chr08	11725000	11730000
O. rufipogon	rufipogon_boundary_1047	rufipogon_chr08	12167500	12172500
O. rufipogon	rufipogon_boundary_1048	rufipogon_chr08	12312500	12317500
O. rufipogon	rufipogon_boundary_1049	rufipogon_chr08	12670000	12675000
O. rufipogon	rufipogon_boundary_1050	rufipogon_chr08	12812500	12817500
O. rufipogon	rufipogon_boundary_1051	rufipogon_chr08	12860000	12865000
O. rufipogon	rufipogon_boundary_1052	rufipogon_chr08	12955000	12960000
O. rufipogon	rufipogon_boundary_1053	rufipogon_chr08	13095000	13100000
O. rufipogon	rufipogon_boundary_1054	rufipogon_chr08	13197500	13202500
O. rufipogon	rufipogon_boundary_1055	rufipogon_chr08	13392500	13397500
O. rufipogon	rufipogon_boundary_1056	rufipogon_chr08	13462500	13467500
O. rufipogon	rufipogon_boundary_1057	rufipogon_chr08	13532500	13537500
O. rufipogon	rufipogon_boundary_1058	rufipogon_chr08	13897500	13902500
O. rufipogon	rufipogon_boundary_1059	rufipogon_chr08	13952500	13957500

O. rufipogon	rufipogon_boundary_1060	rufipogon_chr08	14042500	14047500
O. rufipogon	rufipogon_boundary_1061	rufipogon_chr08	14392500	14397500
O. rufipogon	rufipogon_boundary_1062	rufipogon_chr08	14457500	14462500
O. rufipogon	rufipogon_boundary_1063	rufipogon_chr08	14567500	14597500
O. rufipogon	rufipogon_boundary_1064	rufipogon_chr08	14645000	14650000
O. rufipogon	rufipogon_boundary_1065	rufipogon_chr08	15057500	15062500
O. rufipogon	rufipogon_boundary_1066	rufipogon_chr08	15247500	15252500
O. rufipogon	rufipogon_boundary_1067	rufipogon_chr08	15337500	15342500
O. rufipogon	rufipogon_boundary_1068	rufipogon_chr08	15427500	15432500
O. rufipogon	rufipogon_boundary_1069	rufipogon_chr08	15552500	15557500
O. rufipogon	rufipogon_boundary_1070	rufipogon_chr08	15632500	15637500
O. rufipogon	rufipogon_boundary_1071	rufipogon_chr08	15817500	15822500
O. rufipogon	rufipogon_boundary_1072	rufipogon_chr08	15917500	15922500
O. rufipogon	rufipogon_boundary_1073	rufipogon_chr08	15962500	15967500
O. rufipogon	rufipogon_boundary_1074	rufipogon_chr08	16145000	16150000
O. rufipogon	rufipogon_boundary_1075	rufipogon_chr08	16237500	16242500
O. rufipogon	rufipogon_boundary_1076	rufipogon_chr08	16342500	16347500
O. rufipogon	rufipogon_boundary_1077	rufipogon_chr08	17502500	17507500
O. rufipogon	rufipogon_boundary_1078	rufipogon_chr08	17552500	17592500
O. rufipogon	rufipogon_boundary_1079	rufipogon_chr08	17777500	17782500
O. rufipogon	rufipogon_boundary_1080	rufipogon_chr08	17857500	17862500
O. rufipogon	rufipogon_boundary_1081	rufipogon_chr08	17902500	17932500
O. rufipogon	rufipogon_boundary_1082	rufipogon_chr08	17982500	17987500
O. rufipogon	rufipogon_boundary_1083	rufipogon_chr08	18697500	18732500
O. rufipogon	rufipogon_boundary_1084	rufipogon_chr08	18792500	18797500
O. rufipogon	rufipogon_boundary_1085	rufipogon_chr08	18887500	18892500
O. rufipogon	rufipogon_boundary_1086	rufipogon_chr08	18932500	18937500
O. rufipogon	rufipogon_boundary_1087	rufipogon_chr08	18977500	18982500
O. rufipogon	rufipogon_boundary_1088	rufipogon_chr08	19047500	19052500
O. rufipogon	rufipogon_boundary_1089	rufipogon_chr08	19097500	19102500
O. rufipogon	rufipogon_boundary_1090	rufipogon_chr08	19162500	19202500
O. rufipogon	rufipogon_boundary_1091	rufipogon_chr08	19687500	19692500

O. rufipogon	rufipogon_boundary_1092	rufipogon_chr08	19742500	19747500
O. rufipogon	rufipogon_boundary_1093	rufipogon_chr08	19842500	19847500
O. rufipogon	rufipogon_boundary_1094	rufipogon_chr08	19902500	19907500
O. rufipogon	rufipogon_boundary_1095	rufipogon_chr08	20087500	20122500
O. rufipogon	rufipogon_boundary_1096	rufipogon_chr08	20357500	20362500
O. rufipogon	rufipogon_boundary_1097	rufipogon_chr08	20422500	20427500
O. rufipogon	rufipogon_boundary_1098	rufipogon_chr08	20682500	20727500
O. rufipogon	rufipogon_boundary_1099	rufipogon_chr08	20767500	20772500
O. rufipogon	rufipogon_boundary_1100	rufipogon_chr08	21077500	21082500
O. rufipogon	rufipogon_boundary_1101	rufipogon_chr08	21152500	21157500
O. rufipogon	rufipogon_boundary_1102	rufipogon_chr08	21427500	21432500
O. rufipogon	rufipogon_boundary_1103	rufipogon_chr08	21567500	21572500
O. rufipogon	rufipogon_boundary_1104	rufipogon_chr08	21902500	21907500
O. rufipogon	rufipogon_boundary_1105	rufipogon_chr08	21952500	21957500
O. rufipogon	rufipogon_boundary_1106	rufipogon_chr08	22092500	22097500
O. rufipogon	rufipogon_boundary_1107	rufipogon_chr08	22182500	22212500
O. rufipogon	rufipogon_boundary_1108	rufipogon_chr08	22377500	22412500
O. rufipogon	rufipogon_boundary_1109	rufipogon_chr08	22477500	22482500
O. rufipogon	rufipogon_boundary_1110	rufipogon_chr08	22522500	22527500
O. rufipogon	rufipogon_boundary_1111	rufipogon_chr08	22937500	22942500
O. rufipogon	rufipogon_boundary_1112	rufipogon_chr08	23027500	23032500
O. rufipogon	rufipogon_boundary_1113	rufipogon_chr08	23087500	23092500
O. rufipogon	rufipogon_boundary_1114	rufipogon_chr08	23147500	23152500
O. rufipogon	rufipogon_boundary_1115	rufipogon_chr08	23297500	23302500
O. rufipogon	rufipogon_boundary_1116	rufipogon_chr08	23352500	23357500
O. rufipogon	rufipogon_boundary_1117	rufipogon_chr08	23417500	23452500
O. rufipogon	rufipogon_boundary_1118	rufipogon_chr08	23902500	23907500
O. rufipogon	rufipogon_boundary_1119	rufipogon_chr08	23987500	23992500
O. rufipogon	rufipogon_boundary_1120	rufipogon_chr08	24397500	24402500
O. rufipogon	rufipogon_boundary_1121	rufipogon_chr08	24477500	24482500
O. rufipogon	rufipogon_boundary_1122	rufipogon_chr08	24567500	24572500
O. rufipogon	rufipogon_boundary_1123	rufipogon_chr08	24837500	24862500

O. rufipogon	rufipogon_boundary_1124	rufipogon_chr08	25002500	25007500
O. rufipogon	rufipogon_boundary_1125	rufipogon_chr08	25067500	25107500
O. rufipogon	rufipogon_boundary_1126	rufipogon_chr08	25337500	25342500
O. rufipogon	rufipogon_boundary_1127	rufipogon_chr08	25477500	25507500
O. rufipogon	rufipogon_boundary_1128	rufipogon_chr09	97500	102500
O. rufipogon	rufipogon_boundary_1129	rufipogon_chr09	170000	175000
O. rufipogon	rufipogon_boundary_1130	rufipogon_chr09	280000	285000
O. rufipogon	rufipogon_boundary_1131	rufipogon_chr09	2107500	2112500
O. rufipogon	rufipogon_boundary_1132	rufipogon_chr09	2197500	2202500
O. rufipogon	rufipogon_boundary_1133	rufipogon_chr09	2532500	2537500
O. rufipogon	rufipogon_boundary_1134	rufipogon_chr09	2657500	2662500
O. rufipogon	rufipogon_boundary_1135	rufipogon_chr09	2737500	2742500
O. rufipogon	rufipogon_boundary_1136	rufipogon_chr09	3592500	3597500
O. rufipogon	rufipogon_boundary_1137	rufipogon_chr09	3777500	3782500
O. rufipogon	rufipogon_boundary_1138	rufipogon_chr09	4722500	4727500
O. rufipogon	rufipogon_boundary_1139	rufipogon_chr09	4772500	4777500
O. rufipogon	rufipogon_boundary_1140	rufipogon_chr09	4847500	4852500
O. rufipogon	rufipogon_boundary_1141	rufipogon_chr09	6380000	6385000
O. rufipogon	rufipogon_boundary_1142	rufipogon_chr09	6537500	6542500
O. rufipogon	rufipogon_boundary_1143	rufipogon_chr09	6672500	6677500
O. rufipogon	rufipogon_boundary_1144	rufipogon_chr09	7132500	7137500
O. rufipogon	rufipogon_boundary_1145	rufipogon_chr09	7182500	7187500
O. rufipogon	rufipogon_boundary_1146	rufipogon_chr09	7257500	7262500
O. rufipogon	rufipogon_boundary_1147	rufipogon_chr09	7947500	7992500
O. rufipogon	rufipogon_boundary_1148	rufipogon_chr09	8112500	8117500
O. rufipogon	rufipogon_boundary_1149	rufipogon_chr09	8275000	8280000
O. rufipogon	rufipogon_boundary_1150	rufipogon_chr09	8660000	8665000
O. rufipogon	rufipogon_boundary_1151	rufipogon_chr09	8747500	8752500
O. rufipogon	rufipogon_boundary_1152	rufipogon_chr09	9382500	9462500
O. rufipogon	rufipogon_boundary_1153	rufipogon_chr09	9742500	9747500
O. rufipogon	rufipogon_boundary_1154	rufipogon_chr09	9840000	9845000
O. rufipogon	rufipogon_boundary_1155	rufipogon_chr09	10712500	10752500

O. rufipogon	rufipogon_boundary_1156	rufipogon_chr09	10812500	10817500
O. rufipogon	rufipogon_boundary_1157	rufipogon_chr09	11002500	11007500
O. rufipogon	rufipogon_boundary_1158	rufipogon_chr09	11080000	11085000
O. rufipogon	rufipogon_boundary_1159	rufipogon_chr09	11127500	11132500
O. rufipogon	rufipogon_boundary_1160	rufipogon_chr09	11507500	11512500
O. rufipogon	rufipogon_boundary_1161	rufipogon_chr09	11592500	11597500
O. rufipogon	rufipogon_boundary_1162	rufipogon_chr09	11697500	11702500
O. rufipogon	rufipogon_boundary_1163	rufipogon_chr09	11762500	11767500
O. rufipogon	rufipogon_boundary_1164	rufipogon_chr09	11822500	11827500
O. rufipogon	rufipogon_boundary_1165	rufipogon_chr09	11882500	11887500
O. rufipogon	rufipogon_boundary_1166	rufipogon_chr09	11947500	11952500
O. rufipogon	rufipogon_boundary_1167	rufipogon_chr09	11992500	12057500
O. rufipogon	rufipogon_boundary_1168	rufipogon_chr09	12472500	12477500
O. rufipogon	rufipogon_boundary_1169	rufipogon_chr09	12532500	12572500
O. rufipogon	rufipogon_boundary_1170	rufipogon_chr09	12627500	12707500
O. rufipogon	rufipogon_boundary_1171	rufipogon_chr09	12767500	12867500
O. rufipogon	rufipogon_boundary_1172	rufipogon_chr09	13167500	13202500
O. rufipogon	rufipogon_boundary_1173	rufipogon_chr09	13762500	13807500
O. rufipogon	rufipogon_boundary_1174	rufipogon_chr09	14447500	14487500
O. rufipogon	rufipogon_boundary_1175	rufipogon_chr09	14572500	14577500
O. rufipogon	rufipogon_boundary_1176	rufipogon_chr09	14637500	14642500
O. rufipogon	rufipogon_boundary_1177	rufipogon_chr09	14722500	14727500
O. rufipogon	rufipogon_boundary_1178	rufipogon_chr09	15112500	15117500
O. rufipogon	rufipogon_boundary_1179	rufipogon_chr09	15192500	15197500
O. rufipogon	rufipogon_boundary_1180	rufipogon_chr09	15467500	15472500
O. rufipogon	rufipogon_boundary_1181	rufipogon_chr09	15517500	15522500
O. rufipogon	rufipogon_boundary_1182	rufipogon_chr09	15602500	15607500
O. rufipogon	rufipogon_boundary_1183	rufipogon_chr09	15672500	15677500
O. rufipogon	rufipogon_boundary_1184	rufipogon_chr09	15957500	15997500
O. rufipogon	rufipogon_boundary_1185	rufipogon_chr09	16037500	16042500
O. rufipogon	rufipogon_boundary_1186	rufipogon_chr09	16087500	16092500
O. rufipogon	rufipogon_boundary_1187	rufipogon_chr09	16217500	16222500

O. rufipogon	rufipogon_boundary_1188	rufipogon_chr09	16282500	16287500
O. rufipogon	rufipogon_boundary_1189	rufipogon_chr09	16552500	16597500
O. rufipogon	rufipogon_boundary_1190	rufipogon_chr09	17097500	17102500
O. rufipogon	rufipogon_boundary_1191	rufipogon_chr09	17182500	17187500
O. rufipogon	rufipogon_boundary_1192	rufipogon_chr09	17267500	17272500
O. rufipogon	rufipogon_boundary_1193	rufipogon_chr09	17377500	17382500
O. rufipogon	rufipogon_boundary_1194	rufipogon_chr09	18267500	18272500
O. rufipogon	rufipogon_boundary_1195	rufipogon_chr09	18352500	18357500
O. rufipogon	rufipogon_boundary_1196	rufipogon_chr09	18407500	18412500
O. rufipogon	rufipogon_boundary_1197	rufipogon_chr09	18467500	18472500
O. rufipogon	rufipogon_boundary_1198	rufipogon_chr09	18595000	18637500
O. rufipogon	rufipogon_boundary_1199	rufipogon_chr09	18967500	18972500
O. rufipogon	rufipogon_boundary_1200	rufipogon_chr09	19012500	19017500
O. rufipogon	rufipogon_boundary_1201	rufipogon_chr09	19102500	19107500
O. rufipogon	rufipogon_boundary_1202	rufipogon_chr09	19187500	19192500
O. rufipogon	rufipogon_boundary_1203	rufipogon_chr09	19267500	19272500
O. rufipogon	rufipogon_boundary_1204	rufipogon_chr09	19372500	19377500
O. rufipogon	rufipogon_boundary_1205	rufipogon_chr09	19432500	19437500
O. rufipogon	rufipogon_boundary_1206	rufipogon_chr09	19492500	19497500
O. rufipogon	rufipogon_boundary_1207	rufipogon_chr09	19542500	19547500
O. rufipogon	rufipogon_boundary_1208	rufipogon_chr09	19607500	19612500
O. rufipogon	rufipogon_boundary_1209	rufipogon_chr09	19667500	19672500
O. rufipogon	rufipogon_boundary_1210	rufipogon_chr09	19767500	19772500
O. rufipogon	rufipogon_boundary_1211	rufipogon_chr09	19862500	19867500
O. rufipogon	rufipogon_boundary_1212	rufipogon_chr09	19912500	19917500
O. rufipogon	rufipogon_boundary_1213	rufipogon_chr09	20232500	20237500
O. rufipogon	rufipogon_boundary_1214	rufipogon_chr09	20352500	20357500
O. rufipogon	rufipogon_boundary_1215	rufipogon_chr10	497500	502500
O. rufipogon	rufipogon_boundary_1216	rufipogon_chr10	627500	632500
O. rufipogon	rufipogon_boundary_1217	rufipogon_chr10	697500	742500
O. rufipogon	rufipogon_boundary_1218	rufipogon_chr10	800000	805000
O. rufipogon	rufipogon_boundary_1219	rufipogon_chr10	937500	942500

O. rufipogon	rufipogon_boundary_1220	rufipogon_chr10	1010000	1015000
O. rufipogon	rufipogon_boundary_1221	rufipogon_chr10	1472500	1477500
O. rufipogon	rufipogon_boundary_1222	rufipogon_chr10	1537500	1542500
O. rufipogon	rufipogon_boundary_1223	rufipogon_chr10	1732500	1737500
O. rufipogon	rufipogon_boundary_1224	rufipogon_chr10	1867500	1872500
O. rufipogon	rufipogon_boundary_1225	rufipogon_chr10	2002500	2007500
O. rufipogon	rufipogon_boundary_1226	rufipogon_chr10	2052500	2057500
O. rufipogon	rufipogon_boundary_1227	rufipogon_chr10	2247500	2252500
O. rufipogon	rufipogon_boundary_1228	rufipogon_chr10	3562500	3567500
O. rufipogon	rufipogon_boundary_1229	rufipogon_chr10	3642500	3647500
O. rufipogon	rufipogon_boundary_1230	rufipogon_chr10	3702500	3707500
O. rufipogon	rufipogon_boundary_1231	rufipogon_chr10	3792500	3797500
O. rufipogon	rufipogon_boundary_1232	rufipogon_chr10	3837500	3842500
O. rufipogon	rufipogon_boundary_1233	rufipogon_chr10	3912500	3917500
O. rufipogon	rufipogon_boundary_1234	rufipogon_chr10	3967500	3972500
O. rufipogon	rufipogon_boundary_1235	rufipogon_chr10	4402500	4407500
O. rufipogon	rufipogon_boundary_1236	rufipogon_chr10	4482500	4527500
O. rufipogon	rufipogon_boundary_1237	rufipogon_chr10	5037500	5042500
O. rufipogon	rufipogon_boundary_1238	rufipogon_chr10	5192500	5197500
O. rufipogon	rufipogon_boundary_1239	rufipogon_chr10	6477500	6482500
O. rufipogon	rufipogon_boundary_1240	rufipogon_chr10	6562500	6567500
O. rufipogon	rufipogon_boundary_1241	rufipogon_chr10	6962500	6967500
O. rufipogon	rufipogon_boundary_1242	rufipogon_chr10	7047500	7052500
O. rufipogon	rufipogon_boundary_1243	rufipogon_chr10	7212500	7217500
O. rufipogon	rufipogon_boundary_1244	rufipogon_chr10	7772500	7812500
O. rufipogon	rufipogon_boundary_1245	rufipogon_chr10	7872500	7877500
O. rufipogon	rufipogon_boundary_1246	rufipogon_chr10	8595000	8600000
O. rufipogon	rufipogon_boundary_1247	rufipogon_chr10	8672500	8677500
O. rufipogon	rufipogon_boundary_1248	rufipogon_chr10	9382500	9387500
O. rufipogon	rufipogon_boundary_1249	rufipogon_chr10	9452500	9457500
O. rufipogon	rufipogon_boundary_1250	rufipogon_chr10	9537500	9542500
O. rufipogon	rufipogon_boundary_1251	rufipogon_chr10	9827500	9832500

O. rufipogon	rufipogon_boundary_1252	rufipogon_chr10	9892500	9897500
O. rufipogon	rufipogon_boundary_1253	rufipogon_chr10	10347500	10352500
O. rufipogon	rufipogon_boundary_1254	rufipogon_chr10	10522500	10527500
O. rufipogon	rufipogon_boundary_1255	rufipogon_chr10	10712500	10717500
O. rufipogon	rufipogon_boundary_1256	rufipogon_chr10	10832500	10837500
O. rufipogon	rufipogon_boundary_1257	rufipogon_chr10	11312500	11317500
O. rufipogon	rufipogon_boundary_1258	rufipogon_chr10	11367500	11372500
O. rufipogon	rufipogon_boundary_1259	rufipogon_chr10	11467500	11472500
O. rufipogon	rufipogon_boundary_1260	rufipogon_chr10	13062500	13067500
O. rufipogon	rufipogon_boundary_1261	rufipogon_chr10	13230000	13235000
O. rufipogon	rufipogon_boundary_1262	rufipogon_chr10	14942500	14947500
O. rufipogon	rufipogon_boundary_1263	rufipogon_chr10	15042500	15047500
O. rufipogon	rufipogon_boundary_1264	rufipogon_chr10	15422500	15492500
O. rufipogon	rufipogon_boundary_1265	rufipogon_chr10	16342500	16347500
O. rufipogon	rufipogon_boundary_1266	rufipogon_chr10	16392500	16397500
O. rufipogon	rufipogon_boundary_1267	rufipogon_chr10	16612500	16647500
O. rufipogon	rufipogon_boundary_1268	rufipogon_chr10	16897500	16902500
O. rufipogon	rufipogon_boundary_1269	rufipogon_chr10	16942500	16947500
O. rufipogon	rufipogon_boundary_1270	rufipogon_chr10	17217500	17222500
O. rufipogon	rufipogon_boundary_1271	rufipogon_chr10	17312500	17317500
O. rufipogon	rufipogon_boundary_1272	rufipogon_chr10	17417500	17422500
O. rufipogon	rufipogon_boundary_1273	rufipogon_chr10	17467500	17472500
O. rufipogon	rufipogon_boundary_1274	rufipogon_chr10	17562500	17597500
O. rufipogon	rufipogon_boundary_1275	rufipogon_chr10	17652500	17657500
O. rufipogon	rufipogon_boundary_1276	rufipogon_chr10	17807500	17847500
O. rufipogon	rufipogon_boundary_1277	rufipogon_chr10	17932500	17937500
O. rufipogon	rufipogon_boundary_1278	rufipogon_chr10	17982500	18042500
O. rufipogon	rufipogon_boundary_1279	rufipogon_chr10	18092500	18127500
O. rufipogon	rufipogon_boundary_1280	rufipogon_chr10	18272500	18277500
O. rufipogon	rufipogon_boundary_1281	rufipogon_chr10	18397500	18402500
O. rufipogon	rufipogon_boundary_1282	rufipogon_chr10	18467500	18472500
O. rufipogon	rufipogon_boundary_1283	rufipogon_chr10	18527500	18532500

O. rufipogon	rufipogon_boundary_1284	rufipogon_chr10	18572500	18612500
O. rufipogon	rufipogon_boundary_1285	rufipogon_chr10	18872500	18877500
O. rufipogon	rufipogon_boundary_1286	rufipogon_chr10	18952500	18957500
O. rufipogon	rufipogon_boundary_1287	rufipogon_chr10	19012500	19017500
O. rufipogon	rufipogon_boundary_1288	rufipogon_chr10	19082500	19102500
O. rufipogon	rufipogon_boundary_1289	rufipogon_chr10	19222500	19227500
O. rufipogon	rufipogon_boundary_1290	rufipogon_chr10	19307500	19312500
O. rufipogon	rufipogon_boundary_1291	rufipogon_chr10	19897500	19902500
O. rufipogon	rufipogon_boundary_1292	rufipogon_chr10	19957500	20002500
O. rufipogon	rufipogon_boundary_1293	rufipogon_chr10	20062500	20102500
O. rufipogon	rufipogon_boundary_1294	rufipogon_chr10	20512500	20547500
O. rufipogon	rufipogon_boundary_1295	rufipogon_chr10	20622500	20627500
O. rufipogon	rufipogon_boundary_1296	rufipogon_chr11	167500	172500
O. rufipogon	rufipogon_boundary_1297	rufipogon_chr11	212500	217500
O. rufipogon	rufipogon_boundary_1298	rufipogon_chr11	287500	292500
O. rufipogon	rufipogon_boundary_1299	rufipogon_chr11	447500	452500
O. rufipogon	rufipogon_boundary_1300	rufipogon_chr11	502500	507500
O. rufipogon	rufipogon_boundary_1301	rufipogon_chr11	762500	767500
O. rufipogon	rufipogon_boundary_1302	rufipogon_chr11	847500	852500
O. rufipogon	rufipogon_boundary_1303	rufipogon_chr11	2547500	2552500
O. rufipogon	rufipogon_boundary_1304	rufipogon_chr11	2642500	2687500
O. rufipogon	rufipogon_boundary_1305	rufipogon_chr11	2787500	2792500
O. rufipogon	rufipogon_boundary_1306	rufipogon_chr11	2902500	2907500
O. rufipogon	rufipogon_boundary_1307	rufipogon_chr11	2952500	2957500
O. rufipogon	rufipogon_boundary_1308	rufipogon_chr11	3517500	3522500
O. rufipogon	rufipogon_boundary_1309	rufipogon_chr11	3572500	3577500
O. rufipogon	rufipogon_boundary_1310	rufipogon_chr11	3677500	3682500
O. rufipogon	rufipogon_boundary_1311	rufipogon_chr11	3737500	3742500
O. rufipogon	rufipogon_boundary_1312	rufipogon_chr11	3887500	3892500
O. rufipogon	rufipogon_boundary_1313	rufipogon_chr11	3942500	3947500
O. rufipogon	rufipogon_boundary_1314	rufipogon_chr11	4027500	4067500
O. rufipogon	rufipogon_boundary_1315	rufipogon_chr11	4452500	4457500

O. rufipogon	rufipogon_boundary_1316	rufipogon_chr11	4522500	4527500
O. rufipogon	rufipogon_boundary_1317	rufipogon_chr11	4942500	4947500
O. rufipogon	rufipogon_boundary_1318	rufipogon_chr11	5002500	5047500
O. rufipogon	rufipogon_boundary_1319	rufipogon_chr11	5167500	5172500
O. rufipogon	rufipogon_boundary_1320	rufipogon_chr11	5222500	5227500
O. rufipogon	rufipogon_boundary_1321	rufipogon_chr11	5267500	5272500
O. rufipogon	rufipogon_boundary_1322	rufipogon_chr11	5342500	5347500
O. rufipogon	rufipogon_boundary_1323	rufipogon_chr11	5687500	5692500
O. rufipogon	rufipogon_boundary_1324	rufipogon_chr11	5742500	5747500
O. rufipogon	rufipogon_boundary_1325	rufipogon_chr11	6132500	6137500
O. rufipogon	rufipogon_boundary_1326	rufipogon_chr11	6177500	6182500
O. rufipogon	rufipogon_boundary_1327	rufipogon_chr11	6595000	6600000
O. rufipogon	rufipogon_boundary_1328	rufipogon_chr11	6687500	6692500
O. rufipogon	rufipogon_boundary_1329	rufipogon_chr11	6782500	6787500
O. rufipogon	rufipogon_boundary_1330	rufipogon_chr11	6862500	6867500
O. rufipogon	rufipogon_boundary_1331	rufipogon_chr11	11437500	11442500
O. rufipogon	rufipogon_boundary_1332	rufipogon_chr11	11507500	11512500
O. rufipogon	rufipogon_boundary_1333	rufipogon_chr11	11982500	12027500
O. rufipogon	rufipogon_boundary_1334	rufipogon_chr11	12345000	12350000
O. rufipogon	rufipogon_boundary_1335	rufipogon_chr11	12425000	12430000
O. rufipogon	rufipogon_boundary_1336	rufipogon_chr11	13575000	13622500
O. rufipogon	rufipogon_boundary_1337	rufipogon_chr11	14505000	14510000
O. rufipogon	rufipogon_boundary_1338	rufipogon_chr11	14607500	14612500
O. rufipogon	rufipogon_boundary_1339	rufipogon_chr11	14685000	14690000
O. rufipogon	rufipogon_boundary_1340	rufipogon_chr11	15837500	15842500
O. rufipogon	rufipogon_boundary_1341	rufipogon_chr11	16062500	16102500
O. rufipogon	rufipogon_boundary_1342	rufipogon_chr11	17177500	17182500
O. rufipogon	rufipogon_boundary_1343	rufipogon_chr11	17300000	17305000
O. rufipogon	rufipogon_boundary_1344	rufipogon_chr11	18092500	18097500
O. rufipogon	rufipogon_boundary_1345	rufipogon_chr11	18142500	18147500
O. rufipogon	rufipogon_boundary_1346	rufipogon_chr11	18247500	18252500
O. rufipogon	rufipogon_boundary_1347	rufipogon_chr11	18352500	18357500

O. rufipogon	rufipogon_boundary_1348	rufipogon_chr11	19027500	19032500
O. rufipogon	rufipogon_boundary_1349	rufipogon_chr11	19095000	19100000
O. rufipogon	rufipogon_boundary_1350	rufipogon_chr11	19157500	19162500
O. rufipogon	rufipogon_boundary_1351	rufipogon_chr11	19232500	19237500
O. rufipogon	rufipogon_boundary_1352	rufipogon_chr11	19387500	19392500
O. rufipogon	rufipogon_boundary_1353	rufipogon_chr11	19462500	19467500
O. rufipogon	rufipogon_boundary_1354	rufipogon_chr11	19892500	19897500
O. rufipogon	rufipogon_boundary_1355	rufipogon_chr11	19937500	19942500
O. rufipogon	rufipogon_boundary_1356	rufipogon_chr11	20052500	20057500
O. rufipogon	rufipogon_boundary_1357	rufipogon_chr11	20682500	20687500
O. rufipogon	rufipogon_boundary_1358	rufipogon_chr11	20792500	20797500
O. rufipogon	rufipogon_boundary_1359	rufipogon_chr11	20877500	20882500
O. rufipogon	rufipogon_boundary_1360	rufipogon_chr11	21087500	21117500
O. rufipogon	rufipogon_boundary_1361	rufipogon_chr11	21222500	21227500
O. rufipogon	rufipogon_boundary_1362	rufipogon_chr11	22992500	22997500
O. rufipogon	rufipogon_boundary_1363	rufipogon_chr11	23047500	23052500
O. rufipogon	rufipogon_boundary_1364	rufipogon_chr11	23107500	23152500
O. rufipogon	rufipogon_boundary_1365	rufipogon_chr11	23277500	23282500
O. rufipogon	rufipogon_boundary_1366	rufipogon_chr11	23507500	23512500
O. rufipogon	rufipogon_boundary_1367	rufipogon_chr11	23602500	23607500
O. rufipogon	rufipogon_boundary_1368	rufipogon_chr11	23762500	23767500
O. rufipogon	rufipogon_boundary_1369	rufipogon_chr11	23832500	23837500
O. rufipogon	rufipogon_boundary_1370	rufipogon_chr11	25642500	25647500
O. rufipogon	rufipogon_boundary_1371	rufipogon_chr11	25712500	25717500
O. rufipogon	rufipogon_boundary_1372	rufipogon_chr11	25807500	25812500
O. rufipogon	rufipogon_boundary_1373	rufipogon_chr11	26032500	26037500
O. rufipogon	rufipogon_boundary_1374	rufipogon_chr11	26087500	26092500
O. rufipogon	rufipogon_boundary_1375	rufipogon_chr11	26687500	26692500
O. rufipogon	rufipogon_boundary_1376	rufipogon_chr11	26752500	26757500
O. rufipogon	rufipogon_boundary_1377	rufipogon_chr11	26937500	26942500
O. rufipogon	rufipogon_boundary_1378	rufipogon_chr11	27002500	27007500
O. rufipogon	rufipogon_boundary_1379	rufipogon_chr12	582500	587500

O. rufipogon	rufipogon_boundary_1380	rufipogon_chr12	682500	687500
O. rufipogon	rufipogon_boundary_1381	rufipogon_chr12	1082500	1087500
O. rufipogon	rufipogon_boundary_1382	rufipogon_chr12	1172500	1177500
O. rufipogon	rufipogon_boundary_1383	rufipogon_chr12	1307500	1312500
O. rufipogon	rufipogon_boundary_1384	rufipogon_chr12	1442500	1447500
O. rufipogon	rufipogon_boundary_1385	rufipogon_chr12	1547500	1552500
O. rufipogon	rufipogon_boundary_1386	rufipogon_chr12	1602500	1607500
O. rufipogon	rufipogon_boundary_1387	rufipogon_chr12	1907500	1912500
O. rufipogon	rufipogon_boundary_1388	rufipogon_chr12	1975000	1980000
O. rufipogon	rufipogon_boundary_1389	rufipogon_chr12	2477500	2482500
O. rufipogon	rufipogon_boundary_1390	rufipogon_chr12	2537500	2542500
O. rufipogon	rufipogon_boundary_1391	rufipogon_chr12	3202500	3207500
O. rufipogon	rufipogon_boundary_1392	rufipogon_chr12	3267500	3312500
O. rufipogon	rufipogon_boundary_1393	rufipogon_chr12	3387500	3392500
O. rufipogon	rufipogon_boundary_1394	rufipogon_chr12	3482500	3487500
O. rufipogon	rufipogon_boundary_1395	rufipogon_chr12	3532500	3537500
O. rufipogon	rufipogon_boundary_1396	rufipogon_chr12	3727500	3767500
O. rufipogon	rufipogon_boundary_1397	rufipogon_chr12	3902500	3907500
O. rufipogon	rufipogon_boundary_1398	rufipogon_chr12	4037500	4042500
O. rufipogon	rufipogon_boundary_1399	rufipogon_chr12	4152500	4157500
O. rufipogon	rufipogon_boundary_1400	rufipogon_chr12	4217500	4252500
O. rufipogon	rufipogon_boundary_1401	rufipogon_chr12	4322500	4327500
O. rufipogon	rufipogon_boundary_1402	rufipogon_chr12	4522500	4527500
O. rufipogon	rufipogon_boundary_1403	rufipogon_chr12	4622500	4627500
O. rufipogon	rufipogon_boundary_1404	rufipogon_chr12	4707500	4712500
O. rufipogon	rufipogon_boundary_1405	rufipogon_chr12	4847500	4852500
O. rufipogon	rufipogon_boundary_1406	rufipogon_chr12	4942500	4977500
O. rufipogon	rufipogon_boundary_1407	rufipogon_chr12	5087500	5092500
O. rufipogon	rufipogon_boundary_1408	rufipogon_chr12	5190000	5195000
O. rufipogon	rufipogon_boundary_1409	rufipogon_chr12	5292500	5297500
O. rufipogon	rufipogon_boundary_1410	rufipogon_chr12	5340000	5345000
O. rufipogon	rufipogon_boundary_1411	rufipogon_chr12	6077500	6082500

O. rufipogon	rufipogon_boundary_1412	rufipogon_chr12	6147500	6152500
O. rufipogon	rufipogon_boundary_1413	rufipogon_chr12	7602500	7607500
O. rufipogon	rufipogon_boundary_1414	rufipogon_chr12	7782500	7787500
O. rufipogon	rufipogon_boundary_1415	rufipogon_chr12	7962500	8002500
O. rufipogon	rufipogon_boundary_1416	rufipogon_chr12	8657500	8662500
O. rufipogon	rufipogon_boundary_1417	rufipogon_chr12	8727500	8732500
O. rufipogon	rufipogon_boundary_1418	rufipogon_chr12	9632500	9637500
O. rufipogon	rufipogon_boundary_1419	rufipogon_chr12	9695000	9700000
O. rufipogon	rufipogon_boundary_1420	rufipogon_chr12	10132500	10137500
O. rufipogon	rufipogon_boundary_1421	rufipogon_chr12	10192500	10197500
O. rufipogon	rufipogon_boundary_1422	rufipogon_chr12	10297500	10332500
O. rufipogon	rufipogon_boundary_1423	rufipogon_chr12	10397500	10402500
O. rufipogon	rufipogon_boundary_1424	rufipogon_chr12	11072500	11077500
O. rufipogon	rufipogon_boundary_1425	rufipogon_chr12	11157500	11162500
O. rufipogon	rufipogon_boundary_1426	rufipogon_chr12	11207500	11212500
O. rufipogon	rufipogon_boundary_1427	rufipogon_chr12	11315000	11320000
O. rufipogon	rufipogon_boundary_1428	rufipogon_chr12	11772500	11817500
O. rufipogon	rufipogon_boundary_1429	rufipogon_chr12	11887500	11892500
O. rufipogon	rufipogon_boundary_1430	rufipogon_chr12	11972500	11977500
O. rufipogon	rufipogon_boundary_1431	rufipogon_chr12	12222500	12227500
O. rufipogon	rufipogon_boundary_1432	rufipogon_chr12	12285000	12290000
O. rufipogon	rufipogon_boundary_1433	rufipogon_chr12	12362500	12367500
O. rufipogon	rufipogon_boundary_1434	rufipogon_chr12	12450000	12455000
O. rufipogon	rufipogon_boundary_1435	rufipogon_chr12	13622500	13627500
O. rufipogon	rufipogon_boundary_1436	rufipogon_chr12	13737500	13742500
O. rufipogon	rufipogon_boundary_1437	rufipogon_chr12	13802500	13807500
O. rufipogon	rufipogon_boundary_1438	rufipogon_chr12	13877500	13882500
O. rufipogon	rufipogon_boundary_1439	rufipogon_chr12	14177500	14182500
O. rufipogon	rufipogon_boundary_1440	rufipogon_chr12	14257500	14262500
O. rufipogon	rufipogon_boundary_1441	rufipogon_chr12	14692500	14697500
O. rufipogon	rufipogon_boundary_1442	rufipogon_chr12	14742500	14747500
O. rufipogon	rufipogon_boundary_1443	rufipogon_chr12	15512500	15517500

O. rufipogon	rufipogon_boundary_1444	rufipogon_chr12	15587500	15632500
O. rufipogon	rufipogon_boundary_1445	rufipogon_chr12	16242500	16247500
O. rufipogon	rufipogon_boundary_1446	rufipogon_chr12	16302500	16347500
O. rufipogon	rufipogon_boundary_1447	rufipogon_chr12	16717500	16722500
O. rufipogon	rufipogon_boundary_1448	rufipogon_chr12	16777500	16782500
O. rufipogon	rufipogon_boundary_1449	rufipogon_chr12	17522500	17527500
O. rufipogon	rufipogon_boundary_1450	rufipogon_chr12	17582500	17587500
O. rufipogon	rufipogon_boundary_1451	rufipogon_chr12	18842500	18847500
O. rufipogon	rufipogon_boundary_1452	rufipogon_chr12	18922500	18927500
O. rufipogon	rufipogon_boundary_1453	rufipogon_chr12	19642500	19682500
O. rufipogon	rufipogon_boundary_1454	rufipogon_chr12	19762500	19767500
O. rufipogon	rufipogon_boundary_1455	rufipogon_chr12	19867500	19872500
O. rufipogon	rufipogon_boundary_1456	rufipogon_chr12	19987500	19992500
O. rufipogon	rufipogon_boundary_1457	rufipogon_chr12	20127500	20132500
O. rufipogon	rufipogon_boundary_1458	rufipogon_chr12	20192500	20197500
O. rufipogon	rufipogon_boundary_1459	rufipogon_chr12	21187500	21222500
O. rufipogon	rufipogon_boundary_1460	rufipogon_chr12	21597500	21602500
O. rufipogon	rufipogon_boundary_1461	rufipogon_chr12	21757500	21762500
O. rufipogon	rufipogon_boundary_1462	rufipogon_chr12	22297500	22302500
O. rufipogon	rufipogon_boundary_1463	rufipogon_chr12	22367500	22372500
O. rufipogon	rufipogon_boundary_1464	rufipogon_chr12	22447500	22517500
O. rufipogon	rufipogon_boundary_1465	rufipogon_chr12	22582500	22587500
O. rufipogon	rufipogon_boundary_1466	rufipogon_chr12	22662500	22667500
