# Supplementary material for: Evolutionary and Functional Conservation of Topologically Associating Domain Boundaries in Rice Genomes
Source: Int J Mol Sci. 2026 Oct 10;27(20):8979. doi: 10.3390/ijms27208979 (PMC13704375; doi:10.3390/ijms27208979)
Supplement: Supplementary file 1 [file ijms-27-08979-s001.zip › Sup_Table_S3.pdf]

Supplemental Table S3: Unfiltered enriched gene ontology (GO) terms using PANTHER. Cellular Component (CC) analyses were excluded due to functional relevancy.

Term	# in REF	# in QUERY	Expected	over/ under	Fold enrichment	Raw P-value	FDR	BP/MF	Conservation
mRNA 3'-end processing (GO:0031124)	21	4	0.21	+	19.16	0.0000662	0.0165	BP	HC
RNA 3'-end processing (GO:0031123)	65	6	0.65	+	9.29	0.0000567	0.015	BP	HC
RNA processing (GO:0006396)	783	24	7.78	+	3.08	0.00000175	0.00057	BP	HC
nucleobase-containing compound biosynthetic process (GO:0034654)	1299	38	12.91	+	2.94	0.00000000551	0.00000582	BP	HC
RNA biosynthetic process (GO:0032774)	1060	31	10.54	+	2.94	0.000000148	0.0000698	BP	HC
nucleic acid biosynthetic process (GO:0141187)	1098	32	10.92	+	2.93	0.0000001	0.0000708	BP	HC
RNA metabolic process	1469	38	14.6	+	2.6	0.000000128	0.0000674	BP	HC

(GO:0016070)									
nucleobase-containing compound metabolic process (GO:0006139)	2318	53	23.04	+	2.3	0.0000000215	0.0000182	BP	HC
nucleic acid metabolic process (GO:0090304)	1908	43	18.97	+	2.27	0.000000744	0.000286	BP	HC
gene expression (GO:0010467)	1951	40	19.39	+	2.06	0.0000166	0.00468	BP	HC
macromolecule biosynthetic process (GO:0009059)	2300	45	22.86	+	1.97	0.0000152	0.0046	BP	HC
biosynthetic process (GO:0009058)	3731	68	37.09	+	1.83	0.000000995	0.000351	BP	HC
primary metabolic process (GO:0044238)	6856	109	68.15	+	1.6	0.000000292	0.000124	BP	HC
metabolic process (GO:0008152)	8151	126	81.03	+	1.56	0.00000011	0.0000667	BP	HC
cellular process (GO:0009987)	12700	189	126.25	+	1.5	0.000000000113	0.000000478	BP	HC

catalytic activity	10207	138	101.47	+	1.36	0.0000385	0.0324	MF	HC
branched-chain amino acid metabolic process	37	7	0.97	7.18	+	0.0000701	0.0135	BP	C
small molecule metabolic process	1309	63	34.48	1.83	+	0.0000058	0.00163	BP	C
metabolic process	8151	322	214.71	1.5	+	0	0	BP	C
cellular process	12700	458	334.53	1.37	+	0	0	BP	C
primary metabolic process	6856	283	180.59	1.57	+	0	0	BP	C
regulation of translation	136	12	3.58	3.35	+	0.000345	0.0456	BP	C
intracellular protein transport	303	20	7.98	2.51	+	0.000231	0.0349	BP	C
nitrogen compound transport	814	39	21.44	1.82	+	0.000363	0.0465	BP	C
protein-containing complex assembly	391	25	10.3	2.43	+	0.0000675	0.0136	BP	C
cellular component assembly	579	32	15.25	2.1	+	0.000107	0.0181	BP	C
cellular component organization	2031	85	53.5	1.59	+	0.0000272	0.00639	BP	C
cellular component organizatio	2314	101	60.95	1.66	+	0.000000793	0.000279	BP	C

n or biogenesis									
cellular component biogenesis	983	49	25.89	1.89	+	0.0000264	0.00656	BP	C
protein-containing complex organization	455	28	11.99	2.34	+	0.000049	0.0104	BP	C
RNA modification	404	25	10.64	2.35	+	0.000111	0.0181	BP	C
macromolecule metabolic process	4801	202	126.46	1.6	+	0	0.0000000154	BP	C
RNA metabolic process	1469	68	38.7	1.76	+	0.0000087	0.0023	BP	C
nucleic acid metabolic process	1908	87	50.26	1.73	+	0.000000878	0.000286	BP	C
nucleobase-containing compound metabolic process	2318	111	61.06	1.82	+	0.00000000193	0.000000906	BP	C
nucleobase-containing small molecule metabolic process	404	24	10.64	2.26	+	0.000271	0.0396	BP	C
translation	577	32	15.2	2.11	+	0.000101	0.0185	BP	C
macromolecule biosynthetic process	2300	108	60.58	1.78	+	0.00000000896	0.00000379	BP	C

biosynthetic process	3731	162	98.28	1.65	+	0.0000000000348	0.000000184	BP	C
gene expression	1951	101	51.39	1.97	+	0.0000000000206	0.000000124	BP	C
protein metabolic process	2518	108	66.33	1.63	+	0.000000721	0.000277	BP	C
phosphate-containing compound metabolic process	668	34	17.6	1.93	+	0.000297	0.0419	BP	C
phosphorus metabolic process	669	34	17.62	1.93	+	0.000305	0.0416	BP	C
RNA processing	783	39	20.63	1.89	+	0.000171	0.0268	BP	C
RNA biosynthetic process	1060	51	27.92	1.83	+	0.0000444	0.00987	BP	C
nucleic acid biosynthetic process	1098	51	28.92	1.76	+	0.000104	0.0184	BP	C
nucleobase-containing compound biosynthetic process	1299	65	34.22	1.9	+	0.00000119	0.000358	BP	C
structural constituent of ribosome	357	23	9.4	2.45	+	0.000116	0.0326	MF	C
structural molecule activity	469	34	12.35	2.75	+	0.000000242	0.000153	MF	C
RNA binding	1611	78	42.44	1.84	+	0.00000036	0.000182	MF	C

nucleic acid binding	3985	151	104.97	1.44	+	0.00000499	0.0018	MF	C
binding	12105	408	318.86	1.28	+	0.00000000599	0.00000505	MF	C
hydrolase activity	3335	133	87.85	1.51	+	0.00000164	0.000692	MF	C
catalytic activity	10207	325	268.86	1.21	+	0.0000729	0.023	MF	C
Group II intron splicing	22	5	0.5	10.08	+	0.000164	0.0409	BP	MC
gene expression	1951	80	43.97	1.82	+	0.00000034	0.000206	BP	MC
macromolecule biosynthetic process	2300	90	51.84	1.74	+	0.000000444	0.000234	BP	MC
macromolecule metabolic process	4801	172	108.21	1.59	+	0.000000000887	0.00000075	BP	MC
metabolic process	8151	261	183.71	1.42	+	0.000000000947	0.000000667	BP	MC
cellular process	12700	384	286.24	1.34	+	0	0.0000000217	BP	MC
biosynthetic process	3731	128	84.09	1.52	+	0.0000017	0.000798	BP	MC
RNA biosynthetic process	1060	45	23.89	1.88	+	0.0000595	0.0168	BP	MC
RNA metabolic process	1469	61	33.11	1.84	+	0.00000598	0.00253	BP	MC
nucleic acid metabolic process	1908	74	43	1.72	+	0.00000659	0.00232	BP	MC

nucleobase-containing compound metabolic process	2318	86	52.24	1.65	+	0.00000624	0.0024	BP	MC
primary metabolic process	6856	230	154.53	1.49	+	0.000000000246	0.00000026	BP	MC
nucleic acid biosynthetic process	1098	47	24.75	1.9	+	0.0000338	0.011	BP	MC
nucleobase-containing compound biosynthetic process	1299	53	29.28	1.81	+	0.0000381	0.0115	BP	MC
protein metabolic process	2518	86	56.75	1.52	+	0.000112	0.0296	BP	MC
RNA helicase activity	80	11	1.8	6.1	+	0.00000305	0.00193	MF	MC
ATP-dependent activity, acting on RNA	80	11	1.8	6.1	+	0.00000305	0.00154	MF	MC
RNA binding	1611	71	36.31	1.96	+	0.000000121	0.000102	MF	MC
binding	12105	326	272.83	1.19		0.000113	0.0475	MF	MC
endomembrane system organization	90	8	1.3	6.14	+	0.0000633	0.0535	BP	LC
cellular process	12700	253	183.85	1.38	+	0.00000000263	0.0000111	BP	LC
protein transport	590	22	8.54	2.58	+	0.0000738	0.052	BP	LC

protein localization	724	25	10.48	2.39	+	0.0000818	0.0494	BP	LC
cellular macromolecule localization	726	25	10.51	2.38	+	0.0000854	0.0451	BP	LC
metabolic process	8151	165	118	1.4	+	0.00000264	0.00279	BP	LC
