# Supplementary material for: Evolutionary and Functional Conservation of Topologically Associating Domain Boundaries in Rice Genomes
Source: Int J Mol Sci. 2026 Oct 10;27(20):8979. doi: 10.3390/ijms27208979 (PMC13704375; doi:10.3390/ijms27208979)
Supplement: Supplementary file 1 [file ijms-27-08979-s001.zip › Sup_Table_S4.pdf]

LIFTOVER → TCBF ↓	HC (%)	C (%)	MC (%)	LC (%)
**HC (%)**	11.84	22.37	32.89	40.13
**C (%)**	4.06	19.19	30.93	41.99
**MC (%)**	0.22	4.48	24.89	44.84
**LC (%)**	0.60	0.90	12.57	36.53

Supplementary Table S4: Percentage overlap of TAD boundaries assigned to each conservation category (HC, C, MC, and LC) by TCBF and the LiftOver-based conservation classification approach adapted from Kurbidaeva et al. 2025 [19]. Each cell represents the percentage of TCBF boundaries in the corresponding row category that overlap by at least 1 bp with a LiftOver-derived boundary in the indicated column category. Orange cells denote the expected highest-overlap comparisons, corresponding to matching conservation categories along the diagonal.
