# Supplementary material for: Evolutionary and Functional Conservation of Topologically Associating Domain Boundaries in Rice Genomes
Source: Int J Mol Sci. 2026 Oct 10;27(20):8979. doi: 10.3390/ijms27208979 (PMC13704375; doi:10.3390/ijms27208979)
Supplement: Supplementary file 1 [file ijms-27-08979-s001.zip › Sup_Table_S5.pdf]

LIFTOVER	Azucena	*O. rufipogon*	IR64	*O. meridionalis*
**Mapped (%)**	99.04	96.21	95.75	80.18
**Unmapped (%)**	01.00	03.79	04.24	19.81

Supplemental Table S5: Percentages of successfully mapped and unmapped TAD boundaries for each rice cultivar using the LiftOver tool [47]. All boundaries were mapped to the Nipponbare reference genome using a minimum matching threshold of 20% (-minMatch = 0.20).
